# Platelet lysate-sodium hyaluronate gel promotes diabetic foot wound healing by regulating oxidative stress and autophagy

**DOI:** 10.1371/journal.pone.0324264

**Published:** 2025-06-06

**Authors:** Chunyu Wang, Yangli Zhang, Hailong Yang, Li Zhou, Zhiwei Xu, Chaojie Jin, Liang Zhou, Letian Shan, Hui Wang

**Affiliations:** 1 School of Pharmaceutical Sciences, Zhejiang Chinese Medical University, Hangzhou, Zhejiang, China; 2 The First Affiliated Hospital, Zhejiang Chinese Medical University, Hangzhou, Zhejiang, China; 3 The Second Affiliated Hospital, Zhejiang Chinese Medical University, Hangzhou, Zhejiang, China; 4 Jinhua Academy, Zhejiang Chinese Medical University, Jinhua, Zhejiang, China; 5 The First People’s Hospital of Linping District, Hangzhou, Zhejiang, China; 6 Hangzhou Lin’an District Hospital of Traditional Chinese Medicine, Hangzhou, Zhejiang, China; 7 Fuyang Academy of Research, Zhejiang Chinese Medical University, Hangzhou, Zhejiang, China; 8 Cell Resource Bank and Integrated Cell Preparation Center of Xiaoshan District, Hangzhou Regional Cell Preparation Center (Shangyu Biotechnology Co., Ltd), Hangzhou, Zhejiang, China; Advanced Materials Technology Research Institute, National Research Centre, EGYPT

## Abstract

Diabetic foot ulcer (DFU) is a major complication of diabetes which is difficult to heal and has high recurrence rates. It often requires amputation and even causes death, imposing significant economic burden on the society. Moreover, the current treatment options are not sufficiently effective, necessitating the search for novel treatments. In this study, therapeutic effect and mechanisms of platelet lysate-sodium hyaluronate gel (PL-HA gel) in the treatment of DFU were studied. Initially, the PL-HA gel was examined for quality control, and then the expression of growth factors (GFs) content in PL was determined. The results demonstrated high expression of GFs in PL and the gel exhibited a porous microstructure, which allowed continuous release of PL to achieve its therapeutic effects. *In vivo* model of type II diabetic rats was established to clarify the therapeutic effect of PL-HA gel and the mechanism by which the PL exerted therapeutic effects was further explored in HUVECs and HSFs. *In vivo* experiments indicated that PL-HA gel promoted wound epithelialization, collagen deposition, Cytokeratin and angiogenesis, as well as suppressed the expression of Beclin-1 and LC3 levels, while up-regulating that of P62. It also increased the SOD and MDA levels, promoted antioxidant effects, to accelerate the recovery of wounds. *In vitro* experiments revealed that PL could promoted wound healing by modulating oxidative stress-related parameters (ROS, SOD) and autophagy-related parameters (ULK1, P62, LC3, Beclin-1). In summary, treatment with the PL-HA gel improved diabetic wounds healing by suppressing oxidative stress and autophagy. The PL dissolved in the gel not only increased its efficacy but also ensured its safety and reliability, demonstrating the promising potential of PL-HA gel to treat DFU.

## Introduction

Diabetic foot ulcer (DFU) is one of the complications of diabetes mellitus (DM), with an increasing prevalence over the past decade, placing a heavy economic burden on the society [1–3]. Patients with DM may develop hyperglycaemia, focal neuropathy and peripheral vasculopathy in the distal lower limbs, which increases foot wound infection and ulceration [4]. Clinically, the wound healing process classified into four stages: hemostatic, inflammatory, proliferative and remodeling [5]. The most frequently used treatments are surgical debridement, growth factors, wound dressings and infection treatment, each with its own strengths and weaknesses [6,7].

Autophagy and oxidative stress have implicated in the wound healing and tissue repair [8]. ULK1, Beclin-1, and LC3 are potential biomarkers for evaluating intracellular autophagic activity. The expression level of P62 (SQSTM1) is inversely related with autophagy, and the degradation of p62 indicates autophagy activation [9]. Drugs that modulate autophagy and prevent bacterial infection, inflammatory response, oxidative stress and cell proliferation are potential treatments for wounds. It has been shown that excess ROS levels induce oxidative stress in cells, thereby triggering autophagy [10].

Growth factors (GFs) can promote the healing process, however, the presence of proteolytic enzymes in the wound environment can degrade GFs, limiting their capacity to accelerate the healing process [11,12]. At each stage of wound healing, different GFs interact with their receptors and extracellular matrix components to accelerate wound healing [13]. Platelet derivatives are rich in GFs, such as PDGF, EGF, bFGF, TGF-β, VEGF, and IGF-1, which have been implicated in keratinocyte proliferation, fibroblast migration, and collagen expression, and vascularization in wounded tissues [14–17]. Following their lysis, PL release GFs, which promotes wound healing [18,19]. Research has shown that autologous sources have been shown to be safe and effective treatments for DFU [20]. For instance, PL can be injected locally to accelerate angiogenesis [21] and cell proliferation [22]. However, its optimal mode of application has not been clarified.

Studies have demonstrated that topical gel biomaterials can protect ulcers from the external factors and help to prevent infection and promote wound healing [23,24]. Hydrogel dressings (e.g., hyaluronic acid) possess antibacterial, hemostatic, anti-inflammatory, antioxidant, and wound healing effects [5,25]. Topical application of hyaluronic acid gel may enhance antioxidant activity, stimulate granulation tissue genesis and vascularization, as well as stabilize the structure of GFs, thus prevent their degradation [26–29]. When used in combination, PL and gel may better leverage the therapeutic benefits of PL in promoting wound healing while providing a safer and more reliable environment.

In this study, the results indicate that the HA gel achieved sustained slow release of PL and maintained a moist environment. Moreover, the PL-HA gel enhanced epithelialization, angiogenesis, and collagen deposition at the wound site, as well as improved oxidative stress and autophagy, thereby accelerating the wound healing process. These data indicate that the PL-HA gel can promote wound healing by regulating oxidative stress and autophagy.

## Materials and methods

### Rat PL extraction and platelet count

Rats were anesthetized with isoflurane, and whole blood was collected from the heart into anticoagulant tube containing 4% sodium citrate and allowed to stand for 2 h at 4°C. The whole blood was centrifuged at 185 g for 5 min and the supernatant (platelet-containing plasma, PCP, before freezing and thawing) was transferred into a centrifuge tube. The collected supernatant was subjected to a freeze-thaw process: it was cryopreserved at −80°C overnight, and thawed at 37°C in a water bath for 10 min until completely melted. This cycle was repeated three times and after the final thawing, high-temperature inactivation was performed at 56°C for 30 min. The sample was centrifuged (Centrifuge 5804 R, Eppendorf) at 514 g for 5 min, and the supernatant was collected and stored in aliquots for further experimental use.

Platelet count: Platelets were conducted on both whole blood and PCP as previously described. The sodium citrate vacuum collection tube was gently shaken to ensure proper mixing of the anticoagulant with the blood. The sample was then injected into Cell Counting Plates and left undisturbed for 10–15 min to allow platelets to settle. Platelet counts were determined using an inverted microscope (Carl Zeiss, Gottingen, Germany).

### Determination of growth factors content in PL

Following PL extraction, the concentration of GFs (HGF, PDGF-BB, PDGF-AB, PDGF-AA) was quantified to verify quality control. The reagents, samples in the ELISA kit (MULTI SCIENCES) were equivalent to room temperature (25°C ± 3°C) before further experiments. PL was diluted to the appropriate concentration and then added to the sample wells, into which the detection antibody was added. After incubation, the wells were washed and the substrate color development. Finally, OD values were measured at a wavelength of 450 nm and 570 nm using a microplate photometer (Multiskan™ FC, ThermoFisher Scientific Inc., Waltham, MA, USA). The concentration of the samples was calculated using a standard curve.

### Preparation of HA gel and PL-HA gel

Medical sodium hyaluronate gel was purchased from HANGZHOU SINGCLEAN MEDICAL PRODUCTS CO.,LTD (Singclean®). Briefly, 1 ml of PBS was mixed with 2 ml of the gel to prepare the HA gel (PBS content is approximately 33% v/v). To prepare the PL-HA gel (PL content is approximately 33% v/v), 1 ml of PL was mixed with 2 ml of the Medical sodium hyaluronate gel.

### HA gel and PL-HA gel quality control

Electron microscopy observation: Following gel preparation in 15 ml centrifuge tubes, the PL and gels were shaken thoroughly and dried using vacuum freezing technology (Labogene 100−9, Denmark). They were sliced to the thickness required for field emission scanning electron microscopy (< 1 mm). The internal morphology of the gels was recorded using a field emission scanning electron microscope (SU8010, Hitachi, Japan).

Gel swelling rate: To determine the swelling rate, the gels were dried, the dry matter was weighed(Wd), and then added to the PBS. The weight was measured at 0 h, 0.25 h, 0.5 h, 1 h, 2 h, 4 h, 8 h, 12 h, 24 h, 36 h, and 48 h as wet weight (Ww), and the swelling ratio formula was calculated using the following formula: swelling ratio = (Ww – Wd)/ Wd.

Gel degradation rate (%):To calculate a gel degradation rate (%): The gel (Ww), tested using triple the amount of PBS for the degradation rate. The remaining gel weight (Wr) was recorded at different times (0 h, 1h, 2 h, 4 h, 8 h, 12 h, 24 h, 36 h, 48 h, 60 h, 72 h, 84 h, 96 h), and the degradation rate was calculated using the following formular: (%) = (Ww-Wr)/ Ww × 100%.

Examination of the release profile (%) of PL-loaded hydrogels: PL contain high concentration of PDGF-BB, and thus, the PDGF-BB content in PL was measured using an ELISA kit. PL-HA gels were prepared as detailed in the manuscript. A total of 300 μL of PL-HA gel (containing 251.75 pg of PDGF-BB) was placed into a 24-well plate, followed by the addition of 900 μL of PBS. The plate was then incubated at 37°C with 5% CO₂. Supernatants were collected at specified time points (4, 12, 24, 48, 72, and 96 hours), and after each collection, the remaining gel was resuspended in fresh PBS. The concentration of PDGF-BB in the supernatant collected at each time point was determined using an ELISA kit (MULTI SCIENCES). The cumulative release was calculated as follows: Total Cumulative Release (%) = [Σ(C_n_ × 900 μL)]/ Total_PDGF-BB_ × 100%, where C_n_ represents the concentration of PDGF-BB measured at each time point (n).

Cytotoxicity assays: Briefly, HSFs were seeded in 96-well plates at a density of 3x10^3^ cells/well. The cells were allowed to attach and then 20 μL of gel was added to each well, followed by addition of 180 μL of medium. For the control group, the wells were filled with PBS. The cells were incubated for 24 and 48 hours and the toxicity of the hydrogel was examined using the CCK-8 assay.

### Establishment of DFU model

Wistar and Goto-Kakizaki (GK) male rats were purchased from SLAC Laboratory Animal Co. Ltd. The Wistar rats served as the control, whereas the GK rats (260-300g) were used to establish the type II diabetes model. All rats were fed under standard environmental conditions in a specific pathogen-free (SPF) room with free access to food and water. The laboratory environment was controlled to a 12-hour light/dark cycle. All animal experiments were approved by the Animal Ethical and Welfare Committee of Zhejiang Chinese Medical University, Hangzhou, China and were conducted according to the guidelines of the National Institutes of Health Animal for the care and use of animals (Ethics No: IACUC-20230501–05).

The rats were grouped according to their fasting blood glucose (FBG) levels. Specifically, GK rats were included if their FBG was at least 50% higher than that of Wistar rats and their FBG levels were ≥7.0 mM. A total of 26 rats were randomly assigned to four groups (each with n ≥ 6): the Control group (Wistar rats, n = 7), the Model group (GK rats, n = 6), the HA group (GK rats, n = 7), and the PL-HA group (GK rats, n = 6).

After the induction of diabetes, the rats were anesthetized with Zoletil 50, and a full-thickness skin wound measuring 5 × 10 mm was created on the rat’s foot. PBS was For rats in the control and model groups, PBS was applied externally, whereas for the HA and PL-HA groups, HA gel and PL-HA gel were applied externally, respectively. The gel was prepared fresh for immediate use and was administered twice weekly. The wound healing rate was processed with Image J using the following formula: Wound healing rate (%) = (Aday0-AdayX)/ Aday0 × 100%. Aday0 was the original wound area on day0, and AdayX was the wound area on dayX. After 18 days of modeling, the rats were euthanized using carbon dioxide. Foot skin samples were collected and divided into two halves: one half was cryoprotected for preservation, and the other half was fixed in 4% paraformaldehyde for further analysis in subsequent experiments.

### H&E staining and masson staining

To determine the therapeutic effect of PL-HA gel on wounds, hematoxylin-eosin (H&E) staining and Masson staining were performed to characterize morphological and collagen deposition. The collected skin samples were dehydrated, fixed and embedded followed by sectioning into 4 μm thick skin slices. H&E staining was using an Auto-stainer (ST5010, Leica, Wetzlar, Germany), and Masson staining was carried out using the Masson’s Trichrome Stain Kit (G1340, Solarbio, China). Masson staining was performed to visualize tissue components: collagen fibers appeared blue, while cytoplasm, muscle fibers, cellulose, and red blood cells were stained red. Nuclei were distinctly colored blue-black. The stained tissue sections were examined under an inverted microscope, and the resulting images were captured and quantitatively analyzed using ImageJ software for further evaluation.

### Immunohistochemistry staining

The production of cytokeratin, angiogenesis, proliferation and collagen deposition in wounds after PL-HA gel intervention was examined through immunohistochemical staining. The sections were deparaffinized, rehydrated and subjected to high-pressure thermal repair for 20 min. Then, 3% hydrogen peroxide was added for incubation with samples to inhibit endogenous peroxidase. Serum closure was carried out and primary antibodies: (anti-pan-cytokeratin (1 µg/ml; Abcam), anti-CD31 (1:2000; Abcam), anti-ki67 (1:200; Abcam), Collagen I (1:400; Abclonal)) were added for incubation overnight. Next, the samples were incubated secondary antibody. Finally, 3,3′-diaminobenzidine tetrahydrochloride (DAB) was added for color development, with a brown color showing positive results. Finally, the images were captured using an inverted microscope and analyzed using Image J software.

### Determination of SOD and MDA levels in rat skin samples

Skin wound samples were weighed and diluted at a ratio of 1:9 for skin to normal saline. The sample was homogenized using a homogenizer (MagNA Lyser, ROCHE, Germany) at 6,000 r/min, once every 30 s for 3–5 cycles. The sample was centrifuged at 382 g for 15 min using a centrifuge (CT15RE, HITACHI). The supernatant was collected and used to determine the protein concentration with the BCA Protein Assay Kit. The levels of superoxide dismutase (SOD) and malondialdehyde (MDA) in the skin were measured using the Superoxide Dismutase (SOD) Assay Kit (A001-3–2) and the Malondialdehyde (MDA) Assay Kit (A003-1–2), respectively.

### *In vitro* experiments

#### Cell culture.

The HUVECs and HSFs were purchased from the Chinese Academy of Sciences (Beijing, China) and cultured at 37°C, 5% CO_2_in normal high-glucose DMEM medium (25 mM glucose) containing 10% FBS.

#### High-glucose modeling condition.

The HUVECs and HSFs were incubated with different concentration of glucose(35, 40, 45, 50, 55, 60, 65, 70, 75 mM). Cells in the control group were incubated with glucose concentration of 25 Mm. The cells were incubated for 24h, 48h, and 72h and then subjected to the CCK-8 assay. Finally, the OD values of the cell culture was recorded at 450 nm using a microplate reader (ThermoFisher Scientific Inc., Waltham, MA, USA) to examine the cell viability. Based on the OD values measured in the CCK-8 assay, the optimal high glucose modeling concentration and modeling time were selected for formal grouping.

### PL concentration conditions

HUVECs and HSFs were incubated with different concentrations of PL: 10X (10% v/v), 20X (5% v/v), 50X (2% v/v). The PL concentration for the control group was 0%, and the incubation was performed for 48h. The cells were subjected to the CCK-8 assay and Cell Scratch Assay. Subsequently, the OD value was recorded at a wavelength of 450 nm using a microplate reader. The images are acquired with an inverted microscope for wound healing rate analysis. Based on the analysis of OD values and wound healing, the optimal PL concentration was selected for formal grouping.

### CCK-8 assay

The viability of HUVECs and HSFs was determined using the CCK-8 assay. The assay was conducted for formal grouping: Control group, Model group, and PL group. HUVECs (2 × 10^3^ cells/well) and HSFs (2 × 10^3^ cells/well) were seeded into 96-well plates and treated as described above. Following the intervention, 100 μl of CCK-8 solution (Beyotime, C0037) was added to the HUVECs and HSFs, which were then incubated at 37°C with 5% CO₂ for 2 hours. After incubation, the optical density (OD) at 450 nm was measured using a microplate reader. Cell viability was calculated and compared to assess the effect of PL on cell proliferation.

### Cell scratch assay

The cell scratch assay was used to evaluate the horizontal migration ability of HUVECs and HSFs under different conditions. Briefly, the HUVECs (3 × 10⁴ cells/well) and HSFs (4 × 10⁴ cells/well) were seeded in 6-well plates, and treated as described above. The cells were incubated until they reached a confluence of 95%. The surface of cell-free area was vertically scratched using a sterile 10 μl pipette tip, followed by washing twice with PBS to remove cell debris. The cells were then cultured with fresh DMEM medium. Next, the scratched areas were photographed using an inverted microscope (Carl Zeiss, Göttingen, Germany) at 0 h, 12 h, and 24 h to assess the cell migration ability. The wound area was calculated using Image-J software. The wound closure rate (%) = (A₀ - A_t_)/ A₀ × 100%, where A₀ is the wound area at 0 h and A_t_ is the remaining area at the specified time.

### Tube formation assay

The effect of PL on angiogenesis of HUVECs was determined using the tube formation assay. Briefly, the HUVECs were treated as described above.. After treatment, 50 μl of Matrigel® Matrix (Corning, 354234) was added to the 96-well plate and cultured at 37°C for 30 min. HUVECs were then digested with 0.25% trypsinization and seeded into pre-coated Matrigel-coated 96-well plates (4 × 104 cells/well) for 4 hours at 37°C. Finally, the cells were examined using an inverted microscope to examine the formation of a pipe network and analyzed using Image-J software.

### Measurement of reactive oxygen species (ROS) production

the effect of PL on intracellular ROS generation in HSFs was assessed using the Reactive Oxygen Species Assay Kit (Beyotime, S0033S). Briefly, the HSFs cells (3 × 10³ cells/well) were seeded into 24 – well plates and treated as described above. The cells were incubated with fresh medium added containing 10 μM DCFH-DA at 37°C for 30 min following the manufacturer’s instructions. The cells were washed three times with serum-free DMEM medium, followed by measurement of fluorescence intensity in randomly selected regions using a Zen software (Carl Zeiss, Göttingen, Germany) and quantified using Image-J software.

### RNA extraction and quantitative real-time polymerase chain reaction (qPCR)

Total RNA was extracted from HUVECs and HSFs using the Trizol reagent and quantified with NanoDrop 2000 (Thermo Fisher Scientific, Inc., USA). It was then subjected to qPCR on the Applied Biosystems StepOnePlus Real-Time PCR System (Thermo Fisher Scientific, Inc., MA, USA). The reaction was performed under the following three-step procedure: hold phase (one cycle at 95°C for 30 s), cycling phase (40 cycles at 95°C for 15 s, 40 cycles at 60°C for 30 s, and 40 cycles at 72°C for 30 s), and melting curve phase (one cycle at 95°C for 15 s, one cycle at 60°C for 60 s, and one cycle at 95°C for 15 s). The expression of ET1 and VEGF in HUVECs as well as the concentration of SOD1, SOD2, Beclin-1, ATG5, ATG7, P62, and LC3B in HSFs was analyzed. In this test, the mRNA of GAPDH served as the reference gene. The relative mRNA expression was calculated using the ^ΔΔ^Ct method, and the primer sequences of all genes are shown in Table 1.

**Table 1:** Primer sequences used for qPCR analysis.

Gene	Forward Primer	Reverse Primer
Beclin-1	5’-TCAAGATCCTGGACCGAGTGACC-3’	5’-CTCCTCTCCTGAGTTAGCCTCTTCC-3’
LC3B	5’-GAGCGAGTTGGTCAAGATCATCCG-3’	5’-GATGTCAGCGATGGGTGTGGATAC-3’
ATG5	5’-AAGCAACTCTGGATGGGATT-3’	5’-GCAGCCACAGGACGAAAC-3’
ATG7	5’-AAGCCCGCAGAGATGTGGAG-3’	5’-GCAGCAATGACGGCAGGAAG-3’
P62	5’-GGTGTCTGTGAGAGGACGAGGAG-3’	5’-TCTGGTGATGGAGCCTCTTACTGG-3’
SOD1	5’-GATGACTTGGGCAAAGGTGGAAATG-3’	5’-CCAATTACACCACAAGCCAAACGAC-3’
SOD2	5’-CGCCCTGGAACCTCACATCAAC-3’	5’-AACGCCTCCTGGTACTTCTCCTC-3’
ET-1	5’-TAGAGTGTGTCTACTTCTGCCA-3’	5’-TTCTTCCTCTCACTAACTGCTG-3’
VEGF	5’-GCCTTGCCTTGCTGCTCTAC-3’	5’-ATGATTCTGCCCTCCTCCTTCTG-3’
GAPDH	5’-CACCCACTCCTCCACCTTTG-3’	5’-CCACCACCCTGTTGCTGTAG-3’

### Western Blot (WB) analysis

Total protein was extracted from the lysate and then separated by sodium dodecyl sulphate-polyacrylamide gel electrophoresis. The protein was transferred to the polyvinylidene fluoride (PVDF) membrane, blocked with serum, incubated overnight with the following primary antibodies: LC3 (1:1000; CST), and P62 (1:1000; CST), ULK1(1:5000; HUABIO). The membrane was washed with TBST, incubated with secondary antibodies and finally scanned using the ChemiDoc imaging system.

### Statistical analysis

The data were analyzed and presented as mean ± standard deviation (SD). Statistical analyses were performed using GraphPad Prism 8.0.2.263 (GraphPad Software Inc. USA). First, the data were normalized based on the control group. Then, each group was tested for normality. Since the data conformed to a normal distribution, One-Way ANOVA was used to compare various groups. Homogeneity of variance was then tested. The least Significant Difference (LSD) test was performed if the variance was homogeneous and the Games-Howell (G) test was performed if the variance was heterogeneous. P < 0.05 and P < 0.01 were considered statistically significant. P < 0.05 and P < 0.01 were considered statistically significant.

## Results

### Measurement of the various GFs in platelet lysate and platelet count

The expression level of GFs was conducted to determine extraction efficiency and quality [30]. The result showed that the platelet lysate contained 95 pg/ ml HGF, 6536.19 pg/ ml PDGF-BB, 3043.75 pg/ ml PDGF-AB and 1515 pg/ ml PDGF-AA (Table 2). Data are expressed as mean ± SD.

**Table 2:** Content of various GFs.

Source	Growth factors	Content (pg/ ml)
PL (Rat)	HGF	95 ± 25
PL (Rat)	PDGF-BB	6536.19 ± 29.74
PL (Rat)	PDGF-AB	3043.75 ± 62.5
PL (Rat)	PDGF-AA	1515 ± 25

### Characterization of HA gel and PL-HA gel

**Fig 1:**
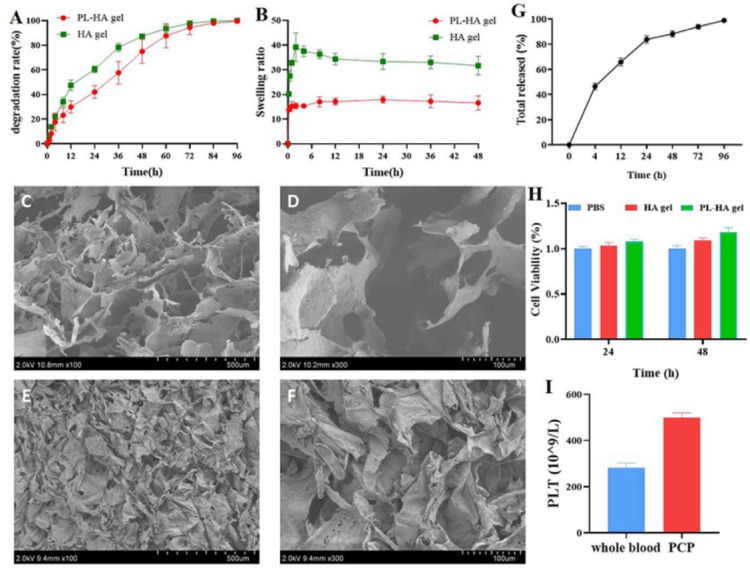
Physical and biological characterization of two gels. **(A-B)**. Degradation rate and expansion ratio of two gels. **(C-D)**. Examination of the microstructure of PL-HA gel through the scanning electron microscope; **(E-F)**. Analysis of the microstructure of HA gel using the scanning electron microscope; **(G)**. The sustained release ability of PL-HA gel as determined by ELISA kits at 4, 12, 24, 48, 72 and 96 h; cumulative amount of released PDGF-BB is shown. **(H)**. Cytotoxicity of gels was detected by CCK-8 assay. **(I)**. Platelet count in whole blood and platelet-containing plasma (PCP). Data are expressed as means ± SD.

To examine the microstructure and assess the cytotoxicity of the gels, the degradation behavior of HA gel and PL-HA gel was evaluated. Both gels underwent rapid degradation within the first 48 h, with the HA gel degrading by 87.27% and the PL-HA gel by 74.93%. After 48 h, the degradation rate slowed significantly. By 84 h, the HA gel had degraded by 99.65%, while the PL-HA gel reached 99.60% degradation after 96 h (Fig 1A). HA gel and PL-HA gel were rapidly absorbed and expanded within 0.5 h. The swelling rate of HA gel reached 27.50% and that of PL-HA gel reached 14.33%. The highest value of HA gel was 39.17%, while that of PL-HA gel was 17.89% (Fig 1B). Electron microscopes revealed that HA gel and PL-HA gel exhibited relatively uniform multilayer structures, and showing typical loose and porous structures. These suggested that gels could effective deliver PL (Fig 1C, 1D, 1E, and 1F). Platelet count (PLT) in whole blood was 282 ± 20.211 × 10^9^/L, platelet-containing plasma was 499.6 ± 19.781 × 10^9^/L (Fig 1I). The results of PL release in gel showed that PL was released slowly in gel (Fig 1G). Cytotoxicity experiments demonstrated that the gels exhibited no significant toxicity to cells and displayed good biocompatibility (Fig 1H).

### Fasting blood glucose levels and body weight of rats during study

**Fig 2:**
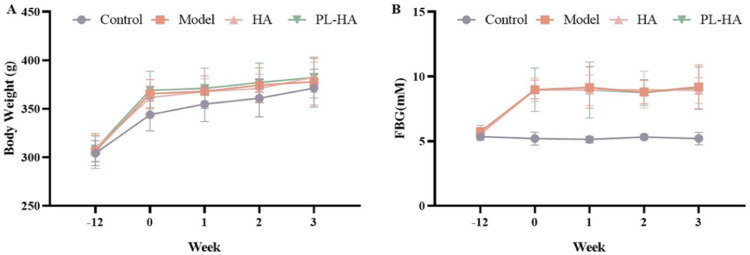
The effect of HA gel and PL-HA gel on body weight and fasting blood glucose in rats. **(A)** Rats’ body weight during the trial; **(B)**. Rats’ Fasting blood glucose (FBG) level during the trial. Data are expressed as the mean ± SD.

The results showed that HA gel treatment and PL-HA gel treatment had no effect on the body weight of GK rats (Fig 2A). The GK rats spontaneously developed diabetes (FBG_(GK)_ ≥ 150% FBG_(Wistar)_, and FBG_(GK)_ ≥ 7.0 mM) and HA and PL-HA did not induce hypoglycemia (Fig 2B).

### Evaluation of wound healing *in vivo*

**Fig. 3:**
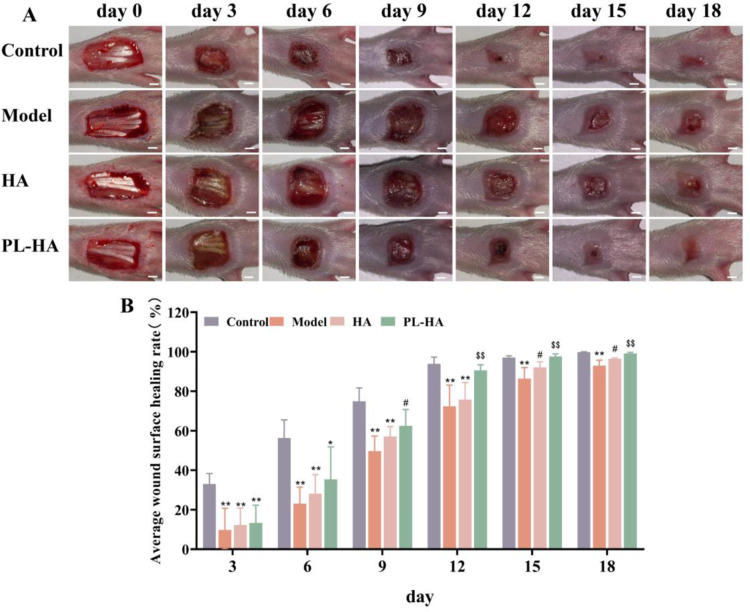
Therapeutic effects of the PL-HA gel on foot wounds healing in rats. **(A)**. Representative images of full-thickness skin treated during the study. Scale bar: 2 mm; **(B)**. Wound healing rate in different groups. Data are expressed as the mean ± SD, ^**^P < 0.01, ^*^P < 0.05 vs control; ^*#*^*P < 0.05 vs model*; ^*$$*^*P < 0.01 vs HA*.

At 3 days, the wound areas of non-diabetic rats were significantly smaller compared with those of diabetic rats. In comparison, at 9 days, the wound area of rats treated with PL-HA gel was significantly reduced. Further analysis showed that the wound area of PL-HA group was significantly smaller than that of HA group at 12 days. At 15 days, the ulcer area of HA group was significantly different from that of model group. However, there was no significant in the ulcer area between PL-HA group and the control group at 18 day. The results showed that treatment with HA gel and PL-HA gel promoted wound healing, with the PL-HA gel achieving better results compared with HA gel (Fig 3A and 3B).

### Histologic analyses of wound healing

**Fig. 4:**
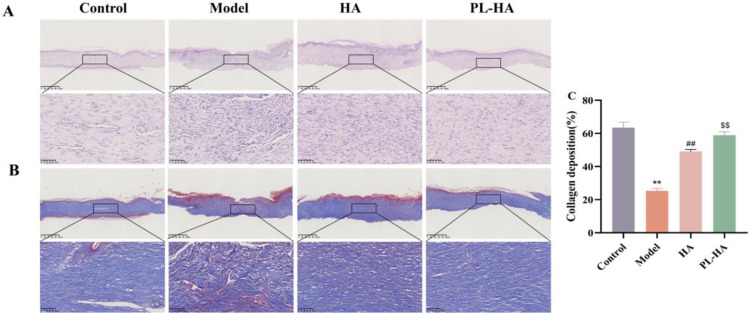
Histologic analyses of wound healing in rats on day18. **(A** and **B)** H&E and Masson staining images of wounds on day 18, bar scale: 500 μm and 50 μm; **(C)** Collagen deposition (blue) showed by Masson staining on day 18. Data are expressed as the mean ± SD, ^**^P < 0.01 vs control; ^*##*^*P < 0.01* vs model; ^*$$*^*P < 0.01* vs HA.

H&E and Masson’s staining were used to examine the pathological changes in wounds treated with PL-HA gel. The control group exhibited enhanced regeneration of blood vessels, hair follicles, and sebaceous glands. In contrast, the model group showed increased inflammation, decreased fibroblasts formation, and inhibited epithelialization. HA gel and PL-HA gel treatments alleviated inflammation, promoted epithelialization, angiogenesis, and the regeneration of hair follicles and sebaceous glands, with groups treated with PL-HA gel showing better results than the HA gel group (Fig 4A). Collagen deposition promoted wound healing, which significantly increased after treatment, with the PL-HA gel treatment achieving better results than the HA gel (Fig 4B and 4C).

Immunohistochemistry analysis of collagen I, Cytokeratin, ki67 and CD31 expression in wounds

Cytokeratin is an important biomarker associated with epidermal differentiation and re-epithelialization [31], and Cytokeratin has been used to assess the extent of re-epithelialization during healing. Blood vessels and cell proliferation are important factors regulating tissue regeneration, providing nutrients and oxygen to cells and enhancing cell regeneration. Type I collagen is a major component of the dermal ECM that contributes to wound healing [32].

**Fig. 5:**
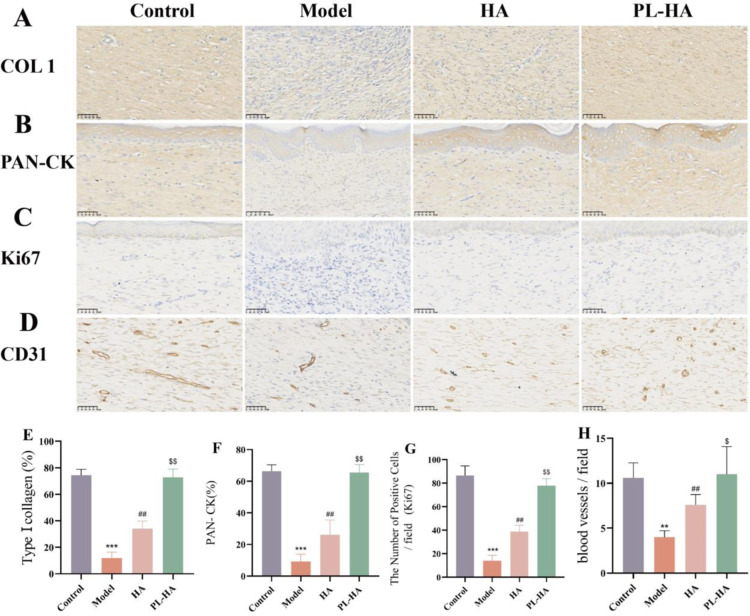
Immunohistochemical analysis of collagen I, Cytokeratin, ki67 and CD31 expression in rat wound healing on day 18. **(A-D)** Representative images showing collagen I, Pan-Cytokeratin, Ki67 and CD31 expression (brown) and nuclei counterstained with hematoxylin (blue) on day 18, bar scale: 50 μm; **(E)** Quantitative analysis of the relative density of collagen I on day 18 after surgery; **(F)** Quantitative analysis of the relative density of Pan-Cytokeratin on day 18; **(G)** Quantification of the number of Ki67 positive cells on day 18; **(H)** The number of blood vessels on day 18 after treatment with HA gel and PL-HA gel. Data are expressed as the mean ± SD, ^***^P < 0.001, ^**^P < 0.01 vs control; ^*##*^*P < 0.01* vs model; ^*$$*^*P < 0.01*, ^*$*^*P < 0.05* vs HA.

We found a significant decrease in the positive rate of Col1 following modeling. However, treatment with HA gel and PL-HA gel led to a notable increase in the positive rate of Col1. Notably, the PL-HA group exhibited a significantly higher intensity of Col1 expression compared to the other groups after treatment, highlighting its enhanced efficacy in promoting collagen production. There was no significant difference between PL-HA group and control group (Fig 5A and 5E). The expression of Pan-Cytokeratin (Pan-ck) was significantly higher in HA and PL-HA groups than in the model group. Furthermore, the intensity of PAN-CK was significantly higher in PL-HA group than in other groups after treatment (Fig 5B and 5F). It was observed that the proliferation of cells was inhibited after modelling. The expression of Ki67 in HA and PL-HA groups was significantly higher relative to the model group, indicating that the cell proliferation was enhanced, with the highest effect detected in the PL-HA group (Fig 5C and 5G). Neovascularization was significantly increased in HA and PL-HA groups compared to model group. In addition, the number of neovascularization in PL-HA group was significantly higher than in other groups after treatment (Fig 5D and 5H).

### Effect of Application of HA gel and PL-HA gel on SOD and MDA levels

**Fig 6:**
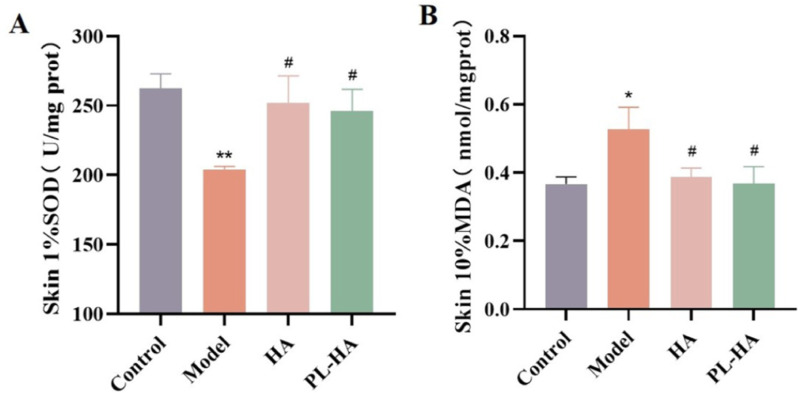
The concentration of SOD and MDA in wound healing tissue on day 18 post-wounding. **(A)** Levels of SOD in wound healing tissue on day 18; **(B)** Levels of MDA in wound healing tissue on day 18. Data are repressed as the mean ± SD, ^**^P < 0.01, ^*^P < 0.05 vs control; ^#^*P* < 0.05 vs model.

Treatment with the gel increased the SOD levels and decreased the MDA levels. In addition, the level of antioxidants was reduced at the wound site (Fig 6A and 6B).

### Levels of autophagy in wounds

**Fig 7:**
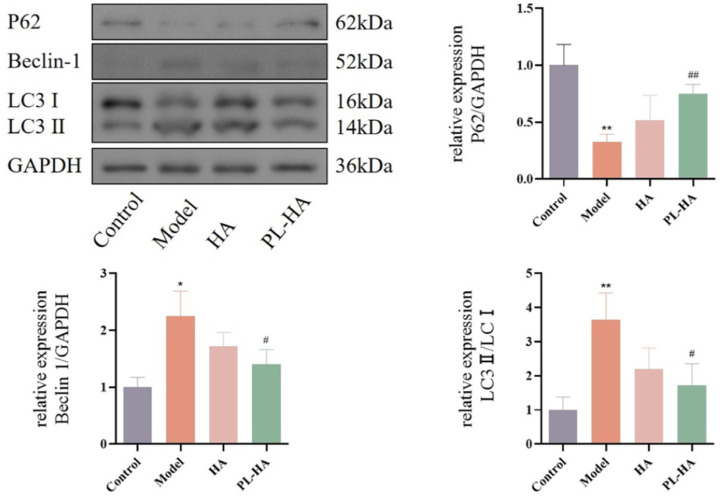
Expression of autophagy-related proteins in wound healing tissue on day 18. Data are expressed as the mean ± SD, ^**^P < 0.01, ^*^P < 0.05 vs control; ^##^*P* < 0.01; ^#^*P* < 0.05 vs model.

The expression level of autophagy-related proteins (LC-3, P62) at the wound was measured to analyze the relationship between diabetic wounds and autophagy. IT was found that the drug decreased LC3II/ I and Beclin-1 levels, increased P62, and inhibited autophagy (Fig 7).

### Exploration of cell high-glucose modeling and drug administration conditions

**Fig 8:**
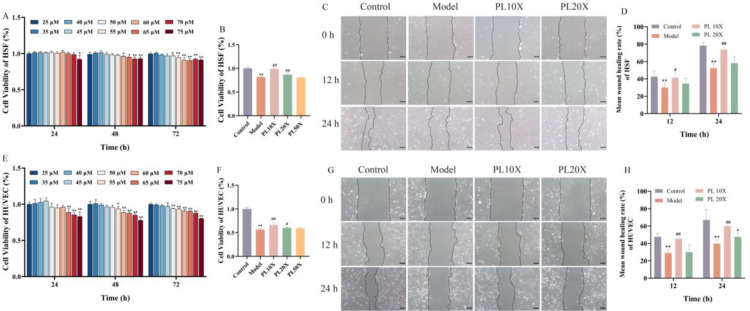
Changes in viability and cell migration of HUVECs and HSFs (each n = 5). **(A** and **B)** Cell viability of HSFs was detected by CCK-8 assay; **(C** and **D)** Cell Scratch Assay showing results of cell migration of HSFs, Scale bar: 100 μm; **(E** and **F)** Cell viability of HUVECs was detected by CCK-8 assay; **(G** and **H)** Cell migration of HUVECs was detected by Cell Scratch Assay, Scale bar: 100 μm. Data are presented as the mean ± SD, ^**^P < 0.01 vs control; ^##^*P* < 0.01; ^#^*P* < 0.05 vs model.

To determine the optimal conditions for cell modeling and drug treatment, we performed the CCK-8 and cell scratch assays. Data demonstrated that the 60 mM of glucose for 48 h was ideal for establishing the HSFs cell model (Fig 8A). Similarly, HUVECs treated with 55 mM glucose for 48 h showed the best results (Fig 8E). PL administered at a concentration of 10X in HSFs and HUVECs achieved the optimal results (Fig 8B, 8C, 8D, 8F, 8G and 8H).

PL promotes cell proliferation, angiogenesis and improves oxidative stress at different time points

**Fig 9:**
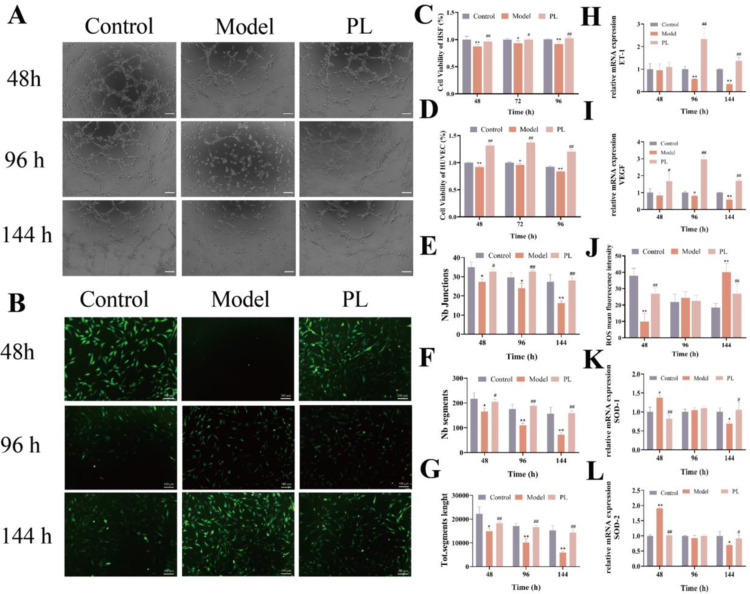
Effects on cell proliferation, angiogenesis and oxidative stress at 48, 96, 144 h after PL administration. **(A, E, F** and **G)** Representative images and quantitative estimation of the tube formation assay in HUVECs calculated by Image-J software (n = 3), Scale bar: 100 μm; **(B** and **J)** Representative images and intracellular ROS measurement of HSFs (n = 5), Scale bar: 100 μm. **(C)** Cell viability of HSFs was detected by CCK-8 assay (n = 5); **(D)** Cell viability of HUVECs was detected by CCK-8 assay (n = 5); **(H** and **I)** mRNA levels of genes associated with tube formation (*ET-1 and VEGF*) in HUVECs (n = 3); **(K** and **L)** mRNA expressions of oxidative stress (*SOD1* and *SOD2*) related genes in HSFs (n = 3). Values were repressed as means ± SD, ^**^P < 0.01, ^*^P < 0.05 vs control; ^##^*P* < 0.01; ^#^*P* < 0.05 vs model.

Data shown in the figures show that the PL promoted the proliferation of both HSFs and HUVECs (Fig 9C and 9D). Additionally, PL enhanced angiogenesis (Fig 9A, 9E, 9F, 9G, 9H, and 9I) and ameliorated oxidative stress (Fig 9B,9J,9K, and 9L). The experimental results revealed that high glucose modeling significantly inhibited cell proliferation and angiogenesis at 48, 96, and 144 h. However, these inhibitory effects were markedly alleviated following the administration of PL, highlighting its potential therapeutic benefits. At 48 h after high glucose exposure, the ROS production was significantly decreased, whereas that of SOD1 and SOD2 was significantly increased. However, at 144 h, ROS level was significantly increased, and that of SOD1 and SOD2 was significantly decreased. However, treatment with the drug improved the pathological changes. These data demonstrate that during different stages of wound healing, PL treatment can suppress oxidative stress.

### PL improves autophagy at different time points

**Fig 10:**
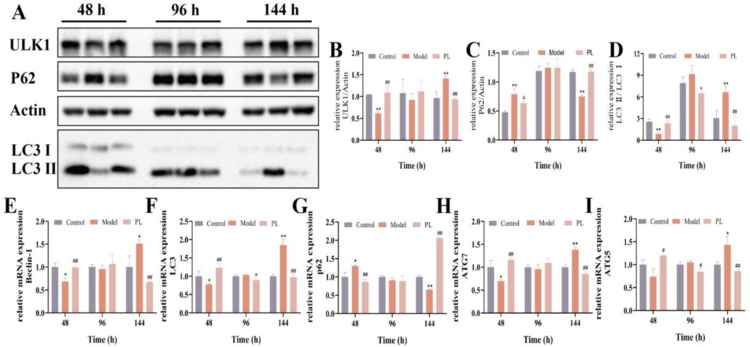
Effects on autophagy at 48, 96, 144 h after PL administration. **(A)** Representative WB images showing the expression of ULK1, P62 and LC3 in HSFs (n = 3); **(B, C** and **D)** Relative protein levels of ULK1, P62 and LC3 in HSFs (n = 3); **(E, F, G, H** and **I)** Relative mRNA expressions levels of Beclin-1, LC3, P62, ATG5 and ATG7 in HSFs (n = 3). Data are shown as the mean ± SD, ^**^P < 0.01, ^*^P < 0.05 vs control; ^##^*P *< 0.01; ^#^*P* < 0.05 vs model.

To examine the effect of PL on autophagy, Western blot and PCR experiments were conducted. After 48 h of high-glucose exposure, the protein levels of ULK1 and LC3 showed a significant decrease (Fig 10A,10B and 10D), accompanied by a marked reduction in the gene expression of Beclin-1, LC3, ATG5, and ATG7 (Fig 10E,10F,10H and 10I). In contrast, the level of P62 significantly increased (Fig 10C and 10G). However, after 144 h of high-glucose exposure, the protein levels of ULK1 and LC3 significantly increased (Fig 10A,10B and 10D), along with an upregulation in the gene expression of Beclin-1, LC3, ATG5, and ATG7 (Fig 10E,10F,10H and 10I). Conversely, the level of P62 showed a significant decrease (Fig 10C and 10G). Notably, the pathological changes were abolished by drug administration. This indicated that PL treatment regulated autophagy in different wound-healing stages.

## Discussion

Research show that DFU is a major complication of in type 1 and type 2 diabetes, with the reported prevalence similar in both diseases [33,34], causing high mortality and amputation rates. DFU often do not heal spontaneously, with about 68% of amputees dying within five years [35–37]. Epidemiological studies have projected that the number of people diagnosed with diabetes is expected to exceed 360 million by 2030, with approximately 15% of patients with diabetes developing DFU [38]. The recurrence of ulcers is influenced by several factors such as neuropathy-related sequelae and location of the ulcer [39]. Wound infection can delay wound healing by causing collagen degradation and localized pressure [40]. Several studies have attempted to develop topical gel-based treatments for DFU. However, there is still no effective treatments, with only one topical gel currently FDA-approved for treating DFU. Considering the increasing incidence of DFU, researchers are increasingly developing new regimens for the treatment of DFU [5]. In this study, we established *in vivo* skin injury model of type Ⅱ diabetic rats to investigate the therapeutic effect of PL-HA gel on DFU. The results showed that PL-HA gel promoted DFU skin healing, angiogenesis and collagen deposition. Moreover, the treatment was safe, without adverse effects. *In vitro* experiments showed that PL influenced oxidative stress and autophagy to accelerate wound healing.

Previous studies have shown that platelet-rich plasma can stimulate angiogenesis [41–45]. Notably, PL releases GFs which stimulate wound healing, shorten the time to achieve complete healing, enhance wound keratinocyte proliferation, fibroblast migration, wound collagen expression, and increased vascularization [18–22]. Moreover, hPL injection therapy was reported to promote keratinocyte migration and foot ulcer healing [46]. Topical application of hyaluronic acid gel stimulates wound healing, enhances antioxidant activity, synthesis of the granulation tissue and vascularization, accelerates collagen deposition and inhibits inflammatory reactions. It also stabilizes the structure of GFs, thereby preventing their degradation and improving their capacity to accelerate wound healing [26–29]. However, the impact of topical platelet lysate on ulcer healing has not been investigated sufficiently. Notably, GFs is highly expressed in PL, which play crucial roles in the wound healing process [47]. GFs promote angiogenesis and cell proliferation. Moreover, autologous sources have been reported to be safe, simple and easy to obtain, making the ideal treatments for DFU [20]. A previous study performed quality for HGF and PDGF using rat and human PL [30]. To ensure the quality control of PL, ELISA kits were utilized to measure HGF, PDGF-AA, PDGF-BB, and PDGF-AB levels. To address the limitations associated with systemic or local PL injections, this study employed the topical application of PL-HA gel.

In this study, we tested the efficacy of direct topical administration of PL-HA gel on the DFU site. Gel dressings provided a moist environment around the wound area, promoted cell migration, collagen formation, angiogenesis, and reduced the wound scarring degree. Currently, few treatment are available for DFU management, which necessitates further research [48,49]. The hyaluronate gel is often used as a carrier of PL because it is highly biocompatible and degradable, and it maintains the stability of GFs, preventing infections to promote healing [2 23,24]. In this study, a multi-layered, loose, and porous sodium hyaluronate gel was applied to maintain the semi-solid state of PL. It was observed that more than 90% of PL-HA gel had degraded at 72 hours, continuously releasing PL within the gel to stimulate wound healing and restore vascularization.

In the rat model of type II diabetes, we found that PL-HA gel was more effective in promoting the formation of granulation tissue, re-epithelialization, hair follicle and sebaceous gland production, collagen deposition, and angiogenesis compared with the HA gel. Moreover, the PL-HA gel improved the antioxidant indexes, SOD and MDA. Results of western blot revealed that the expression of endogenous LC3II/I, Beclin-1, and P62 was significantly altered, suggesting their effect on autophagy. Notably, treatment with PL-HA gel improved the rate of foot wound healing in type II diabetic rats. In addition, direct topical application of PL-HA gel enhanced the wound healing process through sustained drug release, while preventing infection and injection-related injury [50].

In this study, we established *in vivo* high-glucose models of HUVECs and HSFs. The results indicated that the levels of oxidative stress and autophagy were significantly affected in the high-glucose environment, which delayed wound healing process. However, after PL treatment, the changes in oxidative stress and autophagy were significantly reversed. Experimental results (ROS, SOD-1, SOD-2) demonstrated that, compared to the high-glucose model group, oxidative stress-related indicators gradually restored to the levels in the control group at 48-, 96-, and 144-hours post-administration. These findings suggest that PL enhances wound healing, potentially through its effects on oxidative stress regulation. In addition, compared with the high-glucose model group, the levels of autophagy-related indicators (ULK1, P62, Beclin – 1, LC3) at 48, 96, and 144 hours after administration were similar to those of the control group, suggesting that PL promoted wound healing by modulating autophagy. In other words, in vitro experiments demonstrated that platelet lysate (PL), an active substance in PL-HA gel, ameliorated high glucose-induced cellular dysfunction by dynamically modulating ROS levels and autophagy markers(e.g., LC3II/I, p62 and ULK1). This mechanism was confirmed in an in vivo diabetic wound model: PL-HA gel treatment significantly increased the MDA level and decreased SOD level, indicating attenuated oxidative stress, while the dynamics of autophagic flux exhibited stage-dependent correlations with healing progression. These findings suggest that PL promotes diabetic wound healing through phase-specific regulation of the oxidative stress-autophagy axis.

Overall, the PL-HA gel proposed in this study is a safe and straightforward topical treatment for DFU. PL was integrated into sodium hyaluronate gel, to achieve controlled release of GFs, provide an external moist environment, maintain PL activity and reduces the loss of GFs, all of which enhanced the wound healing. The gel also promoted wound healing by regulating oxidative stress and autophagy.

## Conclusions

This study demonstrates that PL-HA gel can stimulate the growth of granulation tissue and the formation of new blood vessels, as well as promote cell proliferation in DFU. Moreover, PL stimulates oxidative stress levels and autophagy levels, thereby enhancing the wound healing process. Moreover, the combination of platelet lysate and the gel achieved sustained slow release of GFs to improve the therapeutic effect, providing a convenient and safe topical treatment of the wound. These results show that the PL-HA gel has great potential for clinical application in the management of DFU.

## Supporting information

S1 File Supplement.material-1. (ZIP)

S2 File Supplement.material-2. (ZIP)

S3 File Supplement.material-3. (ZIP)

S4 File Supplement.material-4. (ZIP)

S5 File Supplement.material-5. (ZIP)

S6 File Supplement.material-6. (ZIP)

S7 File Supplement.material-7. (ZIP)

S8 File Supplement.material-8. (ZIP)

S9 File Supplement.material-9. (ZIP)

## Abbreviations

DM: Diabetes mellitus
DFU: Diabetic foot ulcer
PL: Platelet lysate
SOD: Superoxide dismutase
MDA: Malondialdehyde
GFs: Growth factors
TGF-β: Transforming growth factor β
PDGF: Platelet derived growth factor
EGF: Epidermal growth factor
HGF: Hepatocyte growth factor
bFGF: Basic fibroblast growth factor
VEGF: Vascular endothelial growth factor
PAN-CK: Pan-Cytokeratin
IGF-1: Insulin-like growth factor 1
qPCR: Quantitative real-time polymerase chain reaction
WB: Western Blot
HUVECs: Human umbilical vein endothelial cells
HSFs: human skin fibroblasts;
SPF: Specific pathogen Free
CCK-8: Cell Counting Kit-8
ROS: Reactive Oxygen Species
DCFH-DA: Dichloro-dihydro-fluorescein Diacetate
FBG: Fasting Blood Glucose
PBS: Phosphate Buffered Saline
FBS: Fetal Bovine Serum
