# Supplementary material for: Platelet lysate-sodium hyaluronate gel promotes diabetic foot wound healing by regulating oxidative stress and autophagy
Source: PLoS One. 2025 Jun 6;20(6):e0324264. doi: 10.1371/journal.pone.0324264 (PMC12143543; doi:10.1371/journal.pone.0324264)
Supplement: S1 File — (ZIP) [file pone.0324264.s001.zip › supplement.material-1/Supplement.Statistical and wound/wound.pdf]

Day0

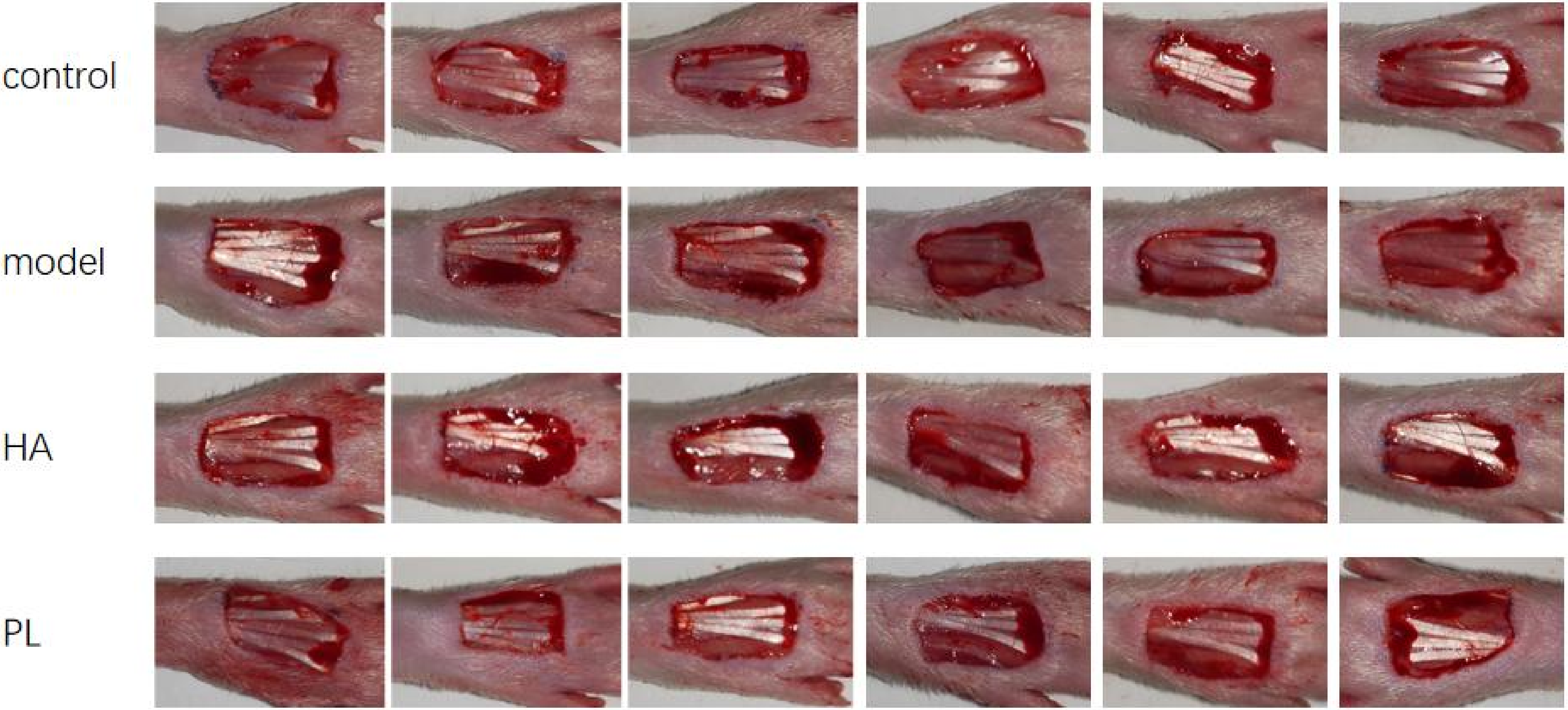

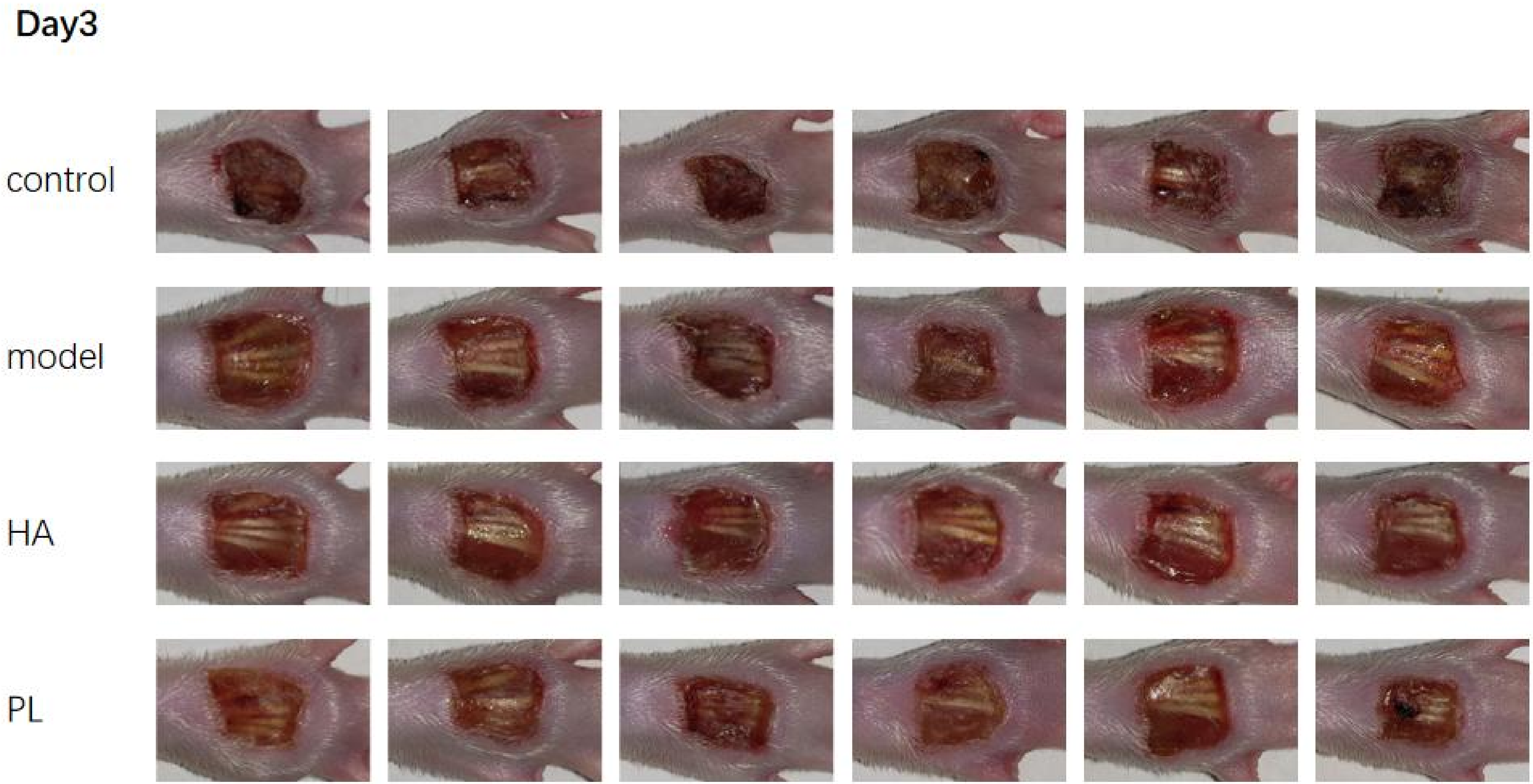
Day3
control
model
HA
PL

Day6

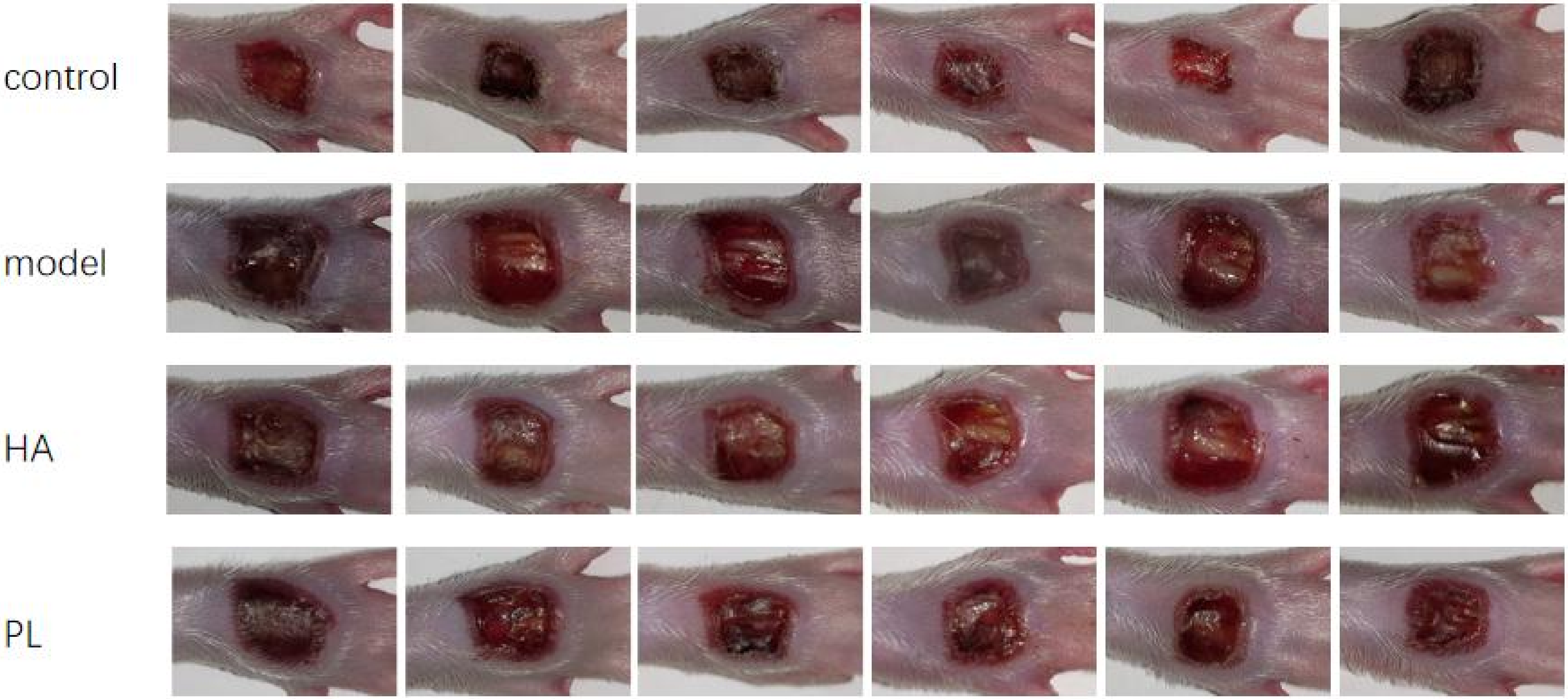

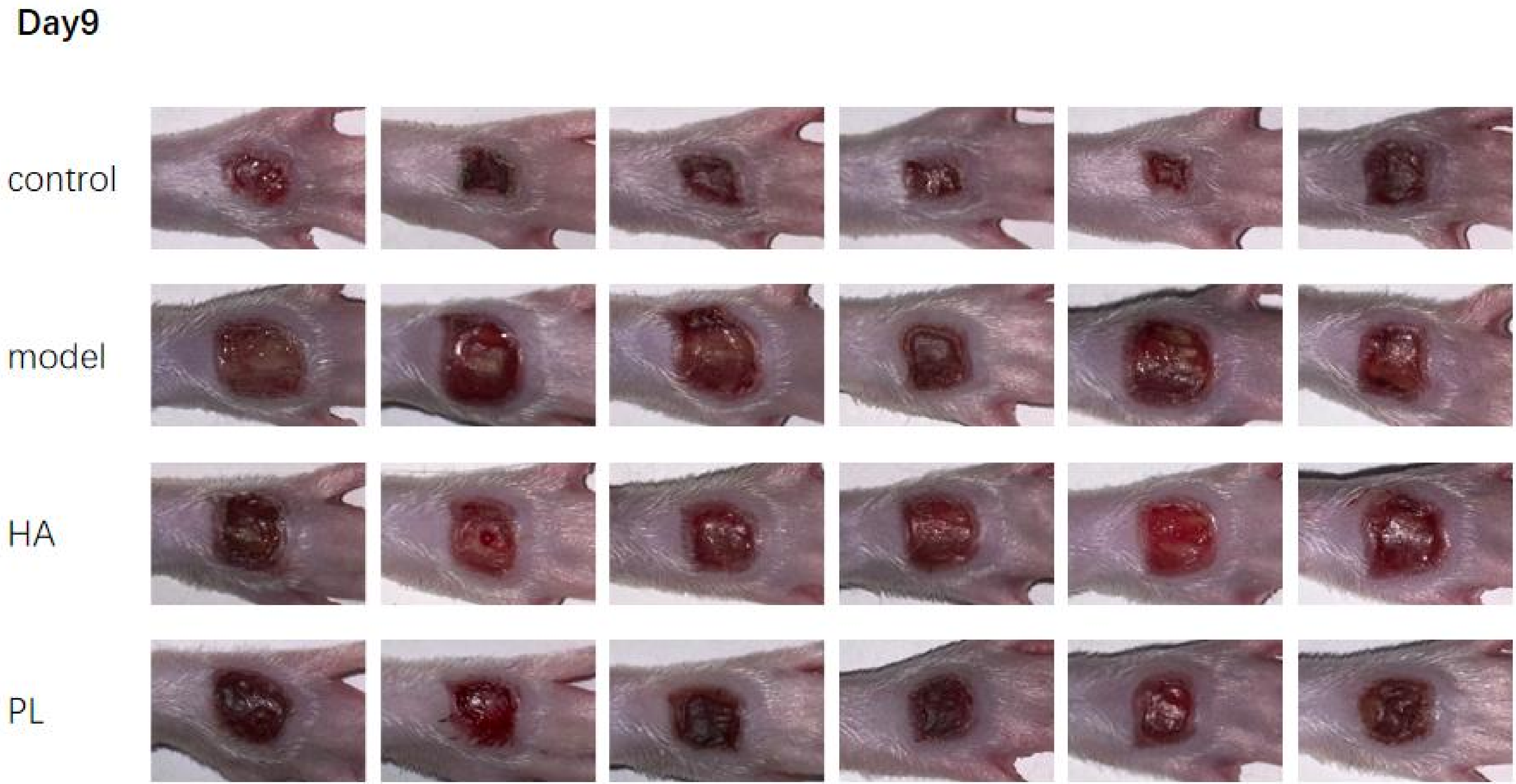
Day9
control
model
HA
PL

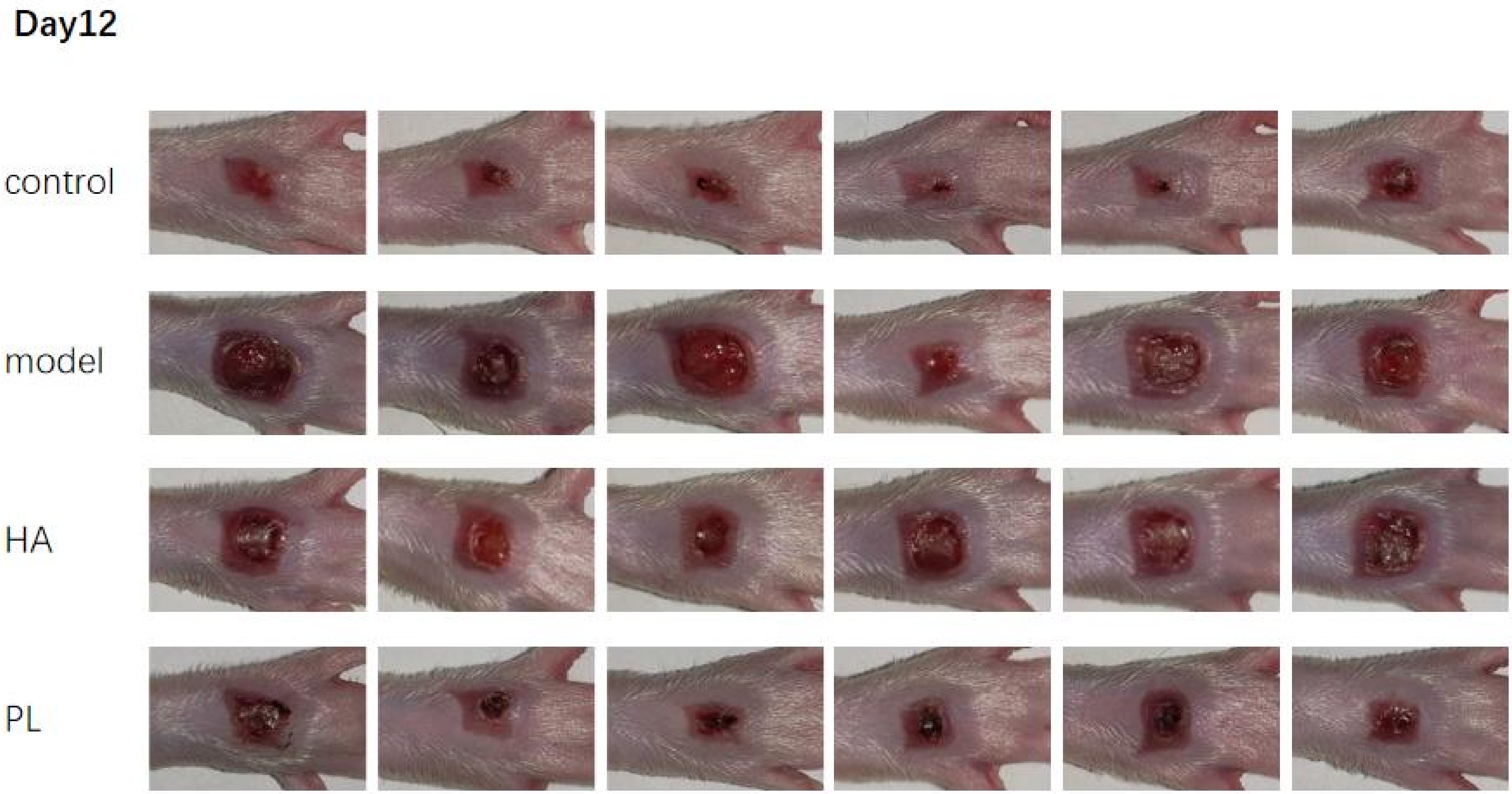
Day12
control
model
HA
PL

Day15

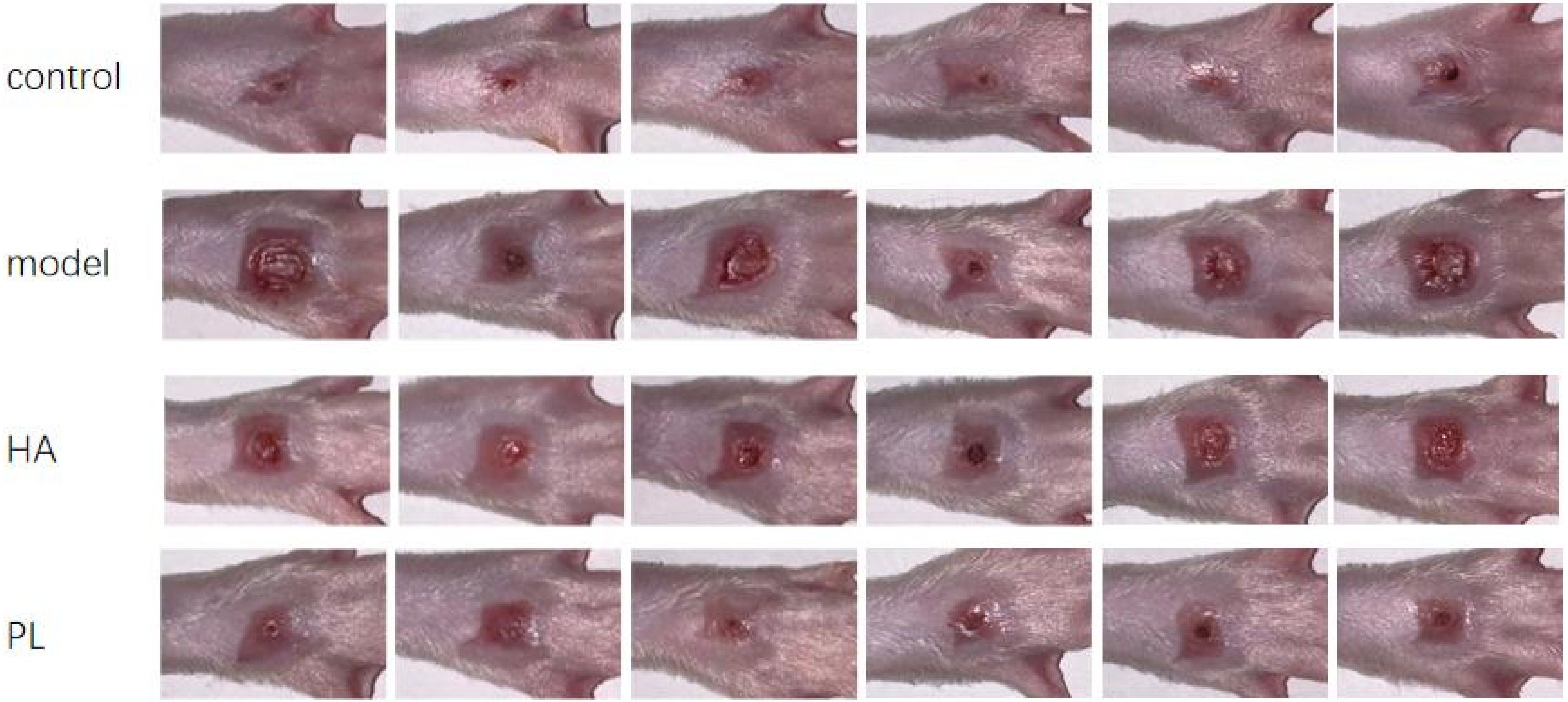

Day18

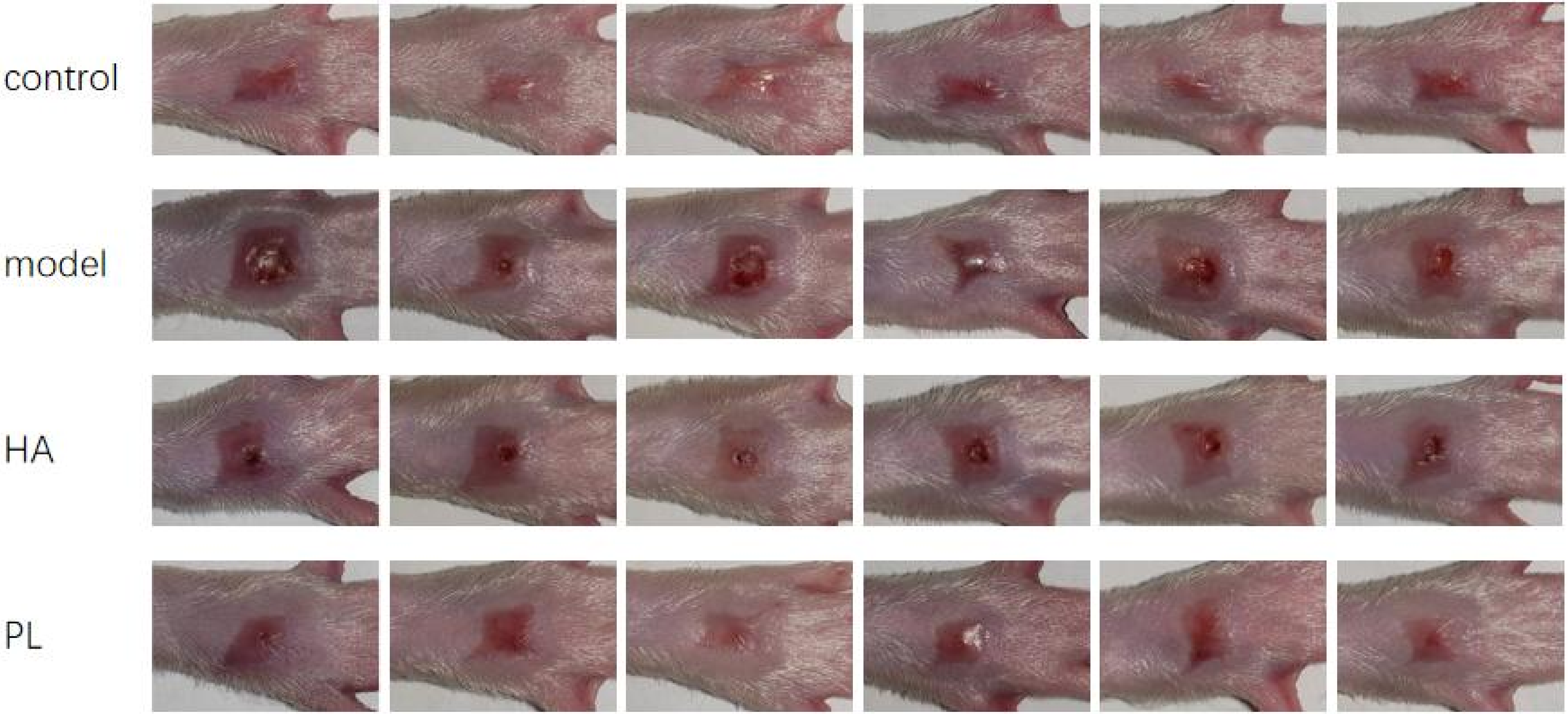

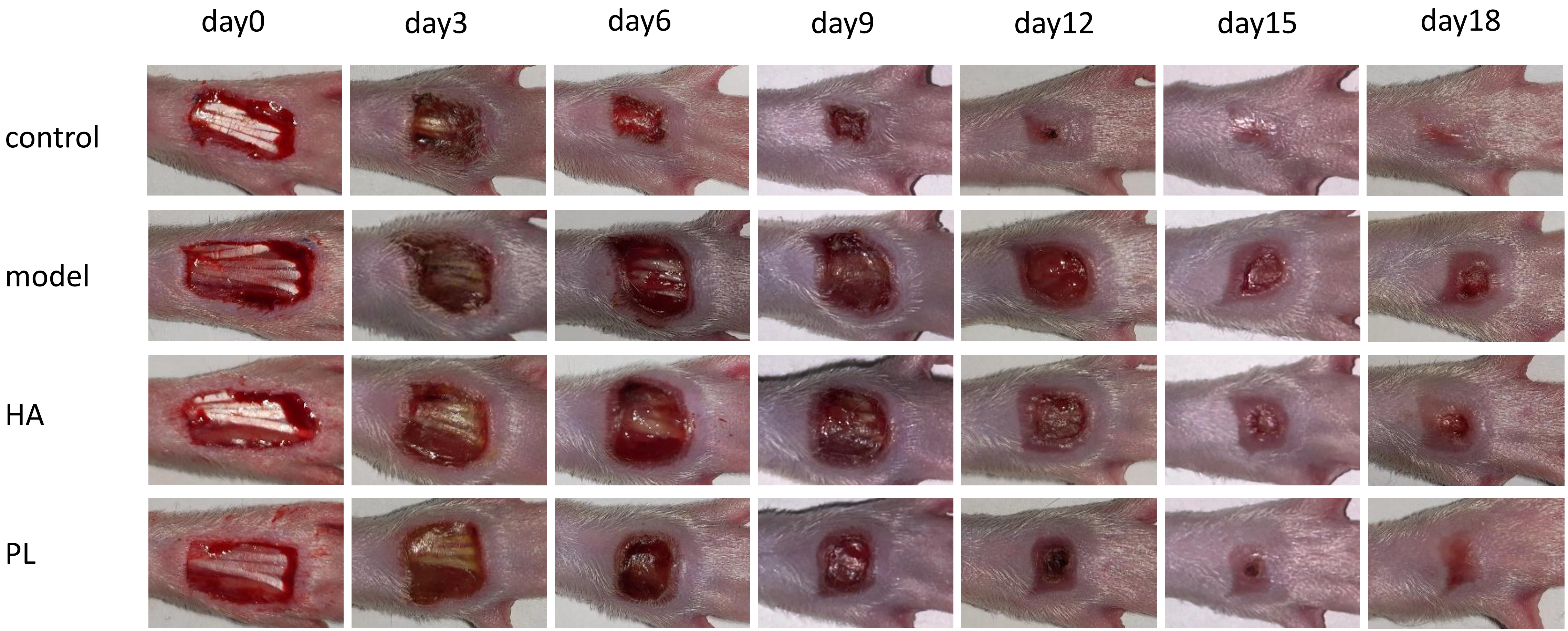
day0
day3
day6
day9
day12
day15
day18
control
model
HA
PL
