# Supplementary figures and images for: Platelet lysate-sodium hyaluronate gel promotes diabetic foot wound healing by regulating oxidative stress and autophagy (part 2 of 4)
Source: PLoS One. 2025 Jun 6;20(6):e0324264. doi: 10.1371/journal.pone.0324264 (PMC12143543; doi:10.1371/journal.pone.0324264)

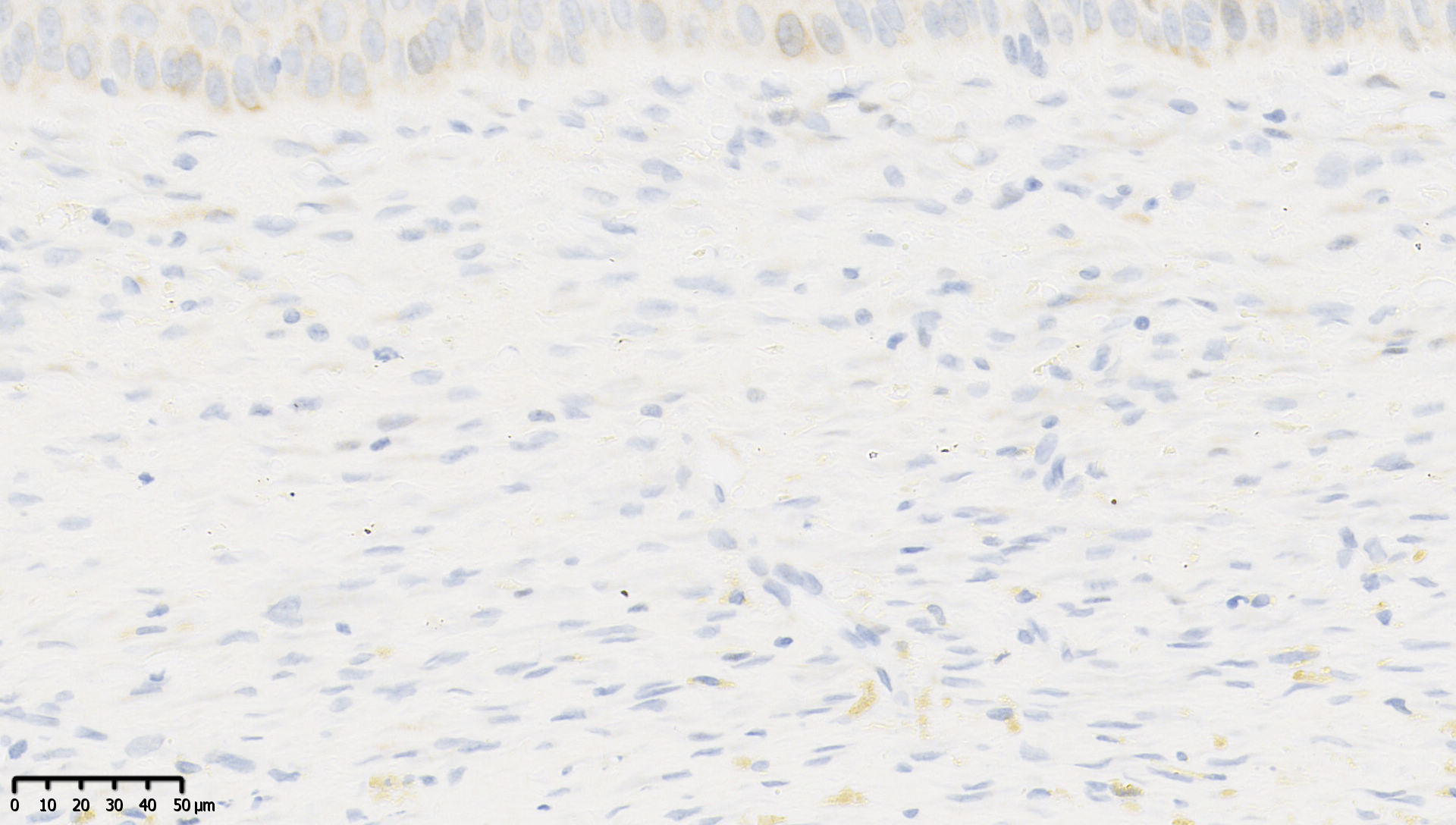

Supplement: S1 File — (ZIP) [file pone.0324264.s001.zip › supplement.material-1/Immunohistochemistry image/KI67/PL-HA-116.jpg]

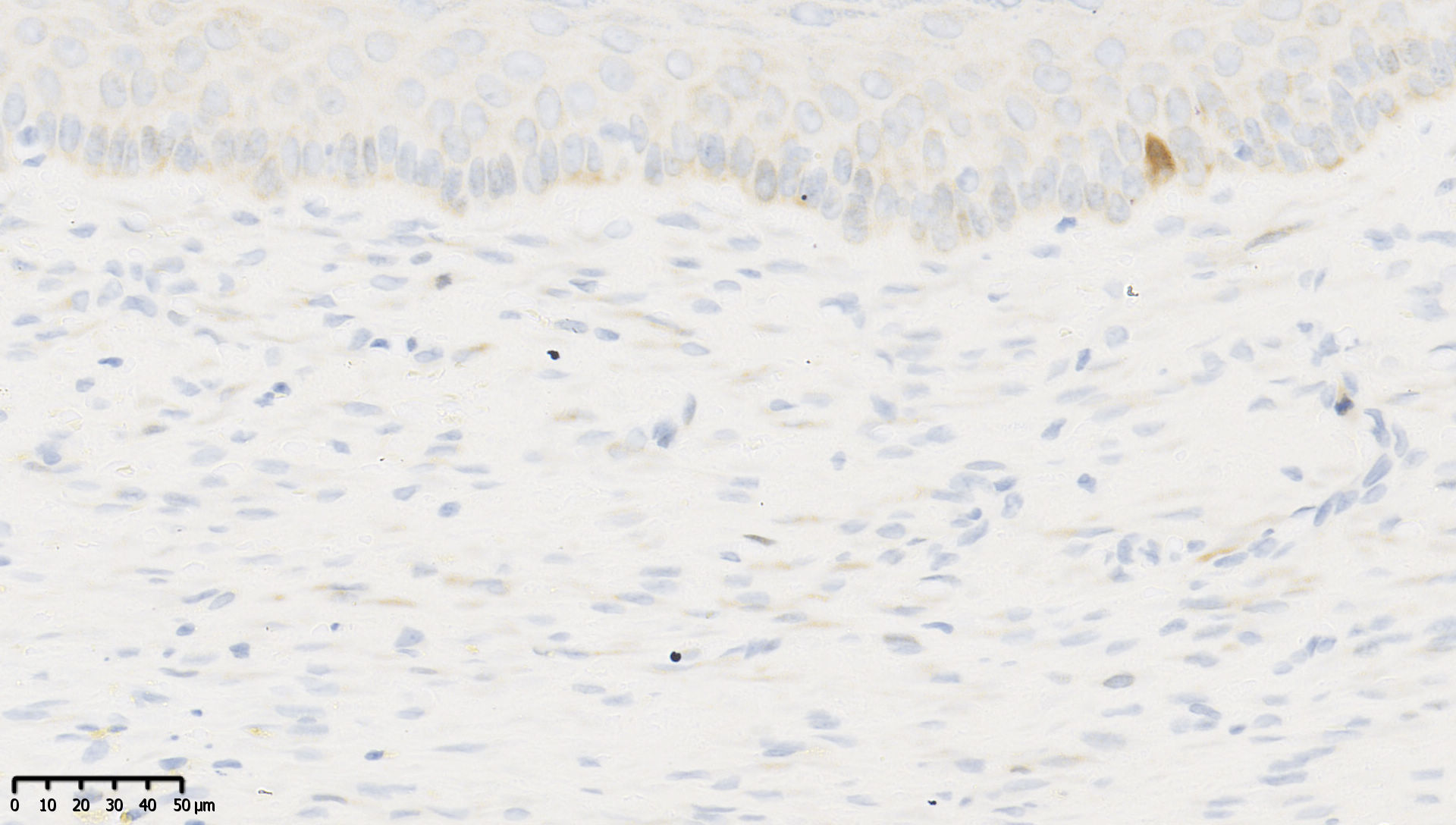

Supplement: S1 File — (ZIP) [file pone.0324264.s001.zip › supplement.material-1/Immunohistochemistry image/KI67/PL-HA-211.jpg]

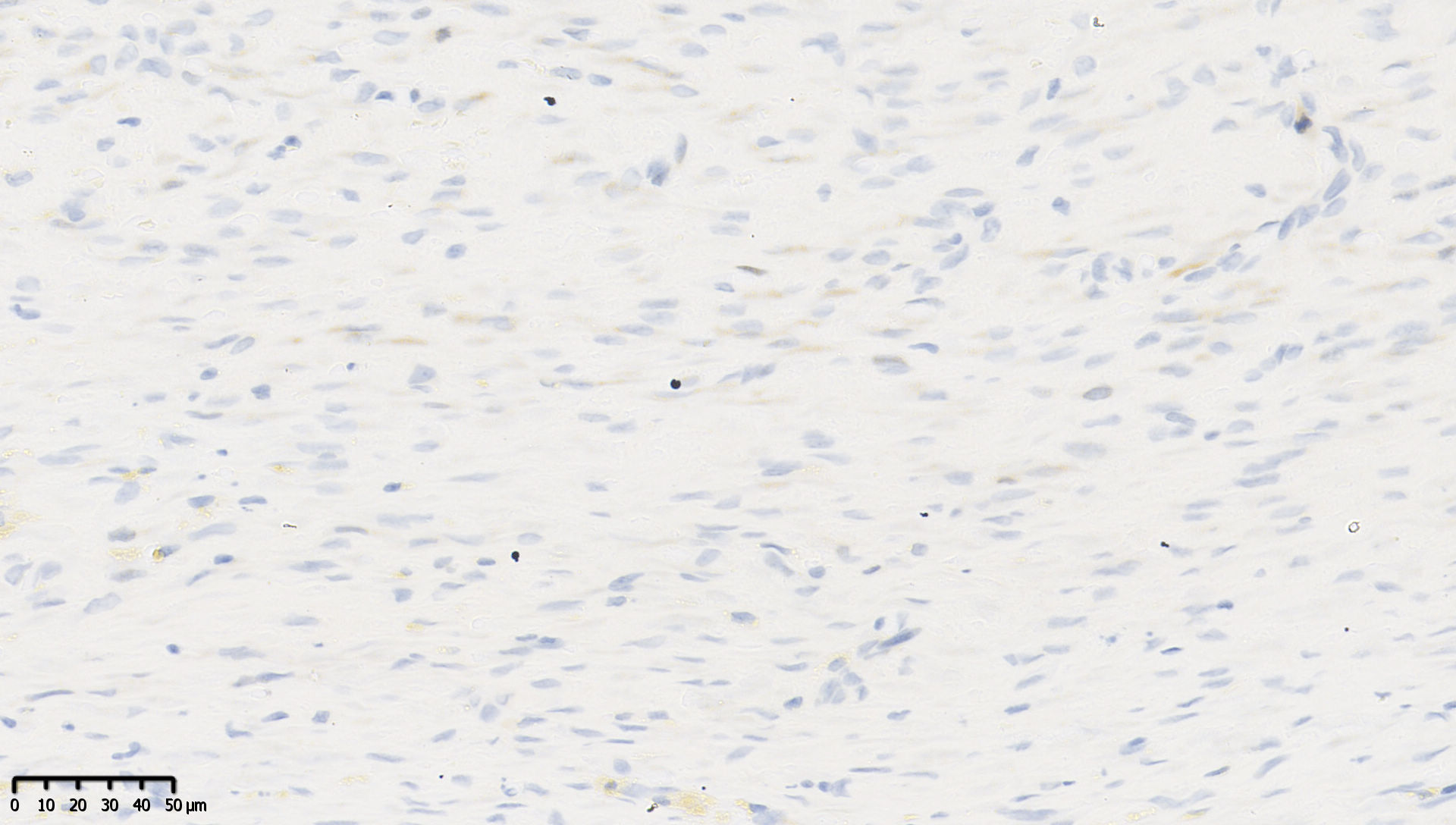

Supplement: S1 File — (ZIP) [file pone.0324264.s001.zip › supplement.material-1/Immunohistochemistry image/KI67/PL-HA-7.jpg]

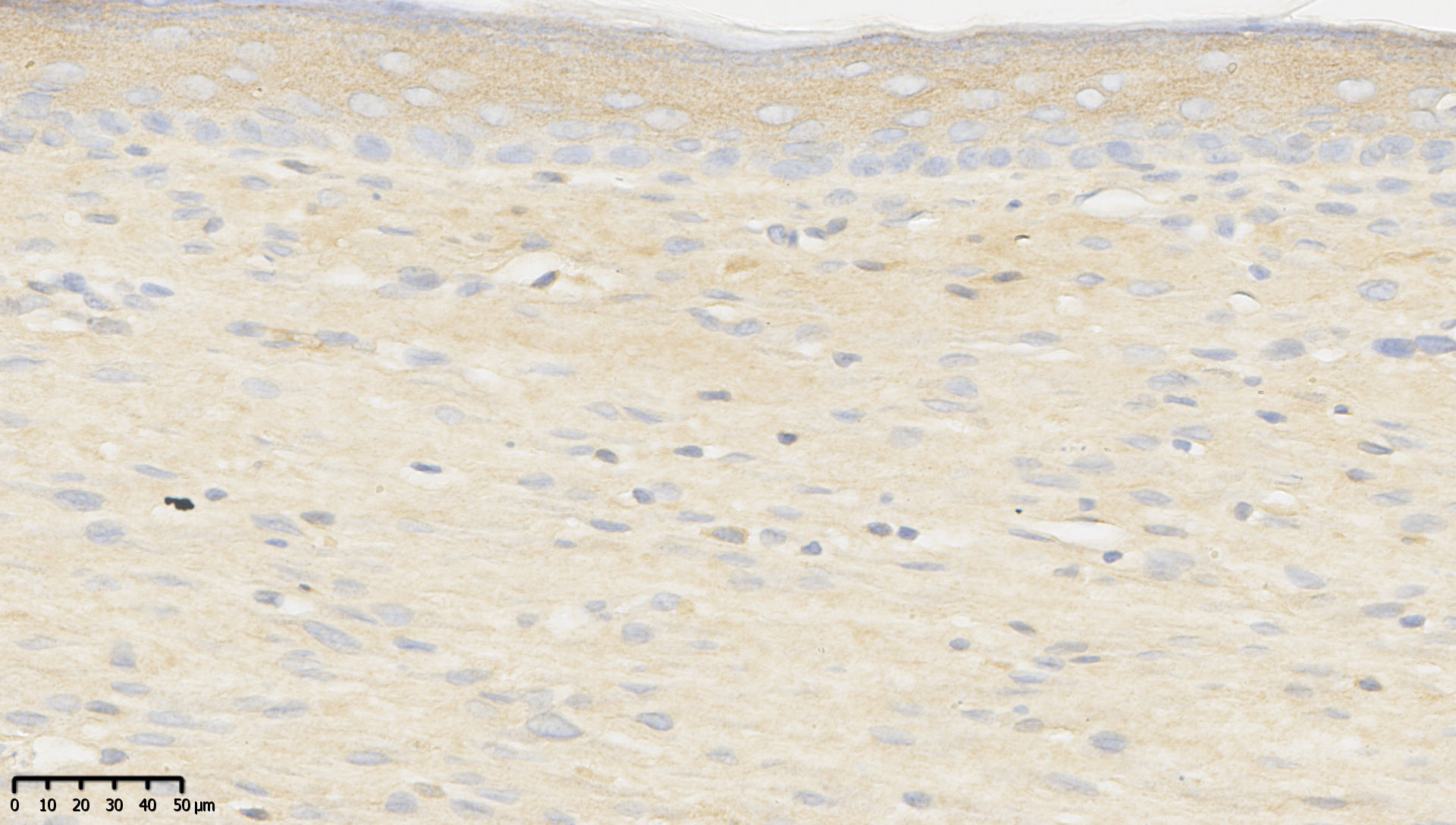

Supplement: S1 File — (ZIP) [file pone.0324264.s001.zip › supplement.material-1/Immunohistochemistry image/pan-cytokeratin/control-1111.jpg]

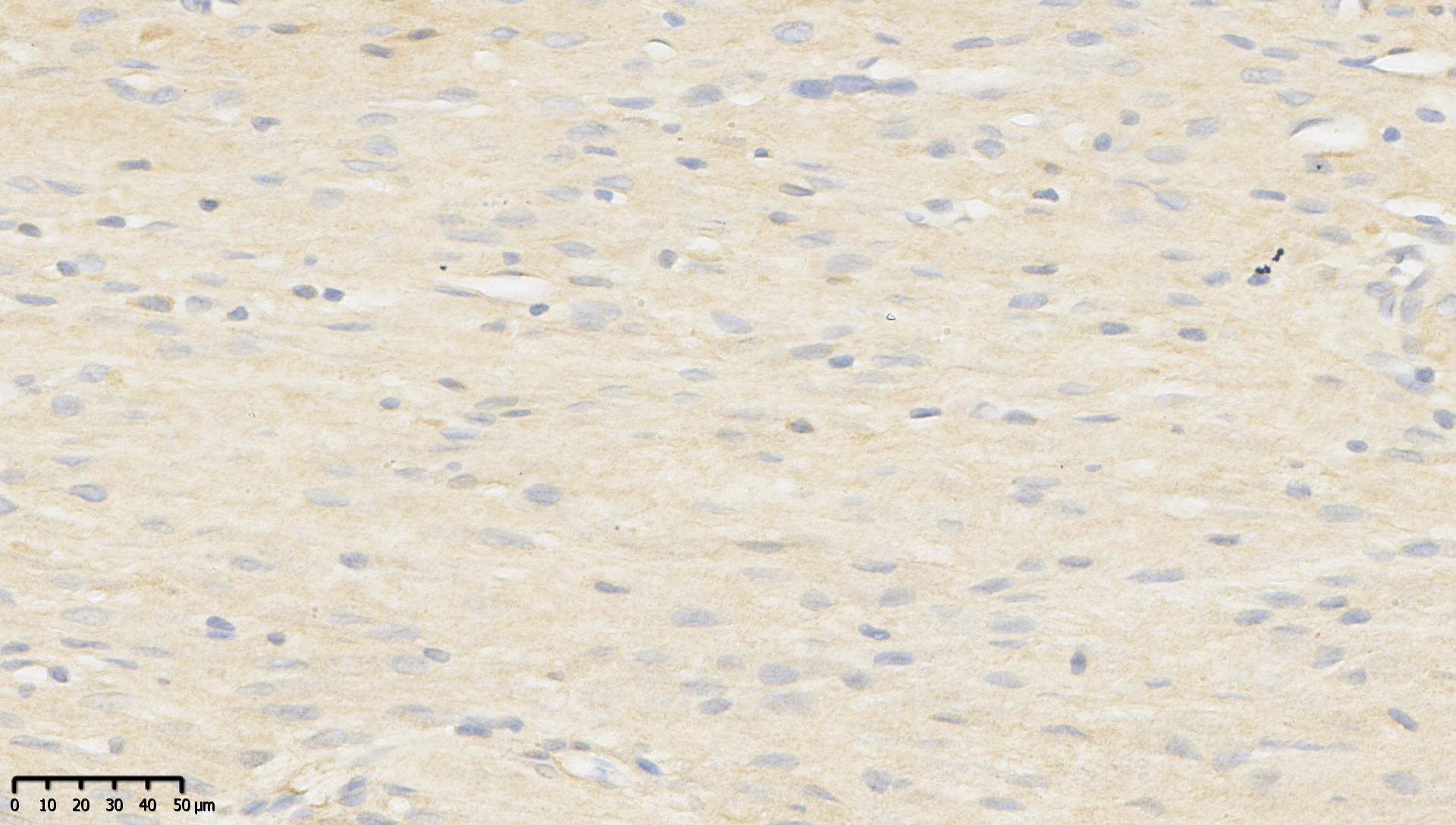

Supplement: S1 File — (ZIP) [file pone.0324264.s001.zip › supplement.material-1/Immunohistochemistry image/pan-cytokeratin/control-1112.jpg]

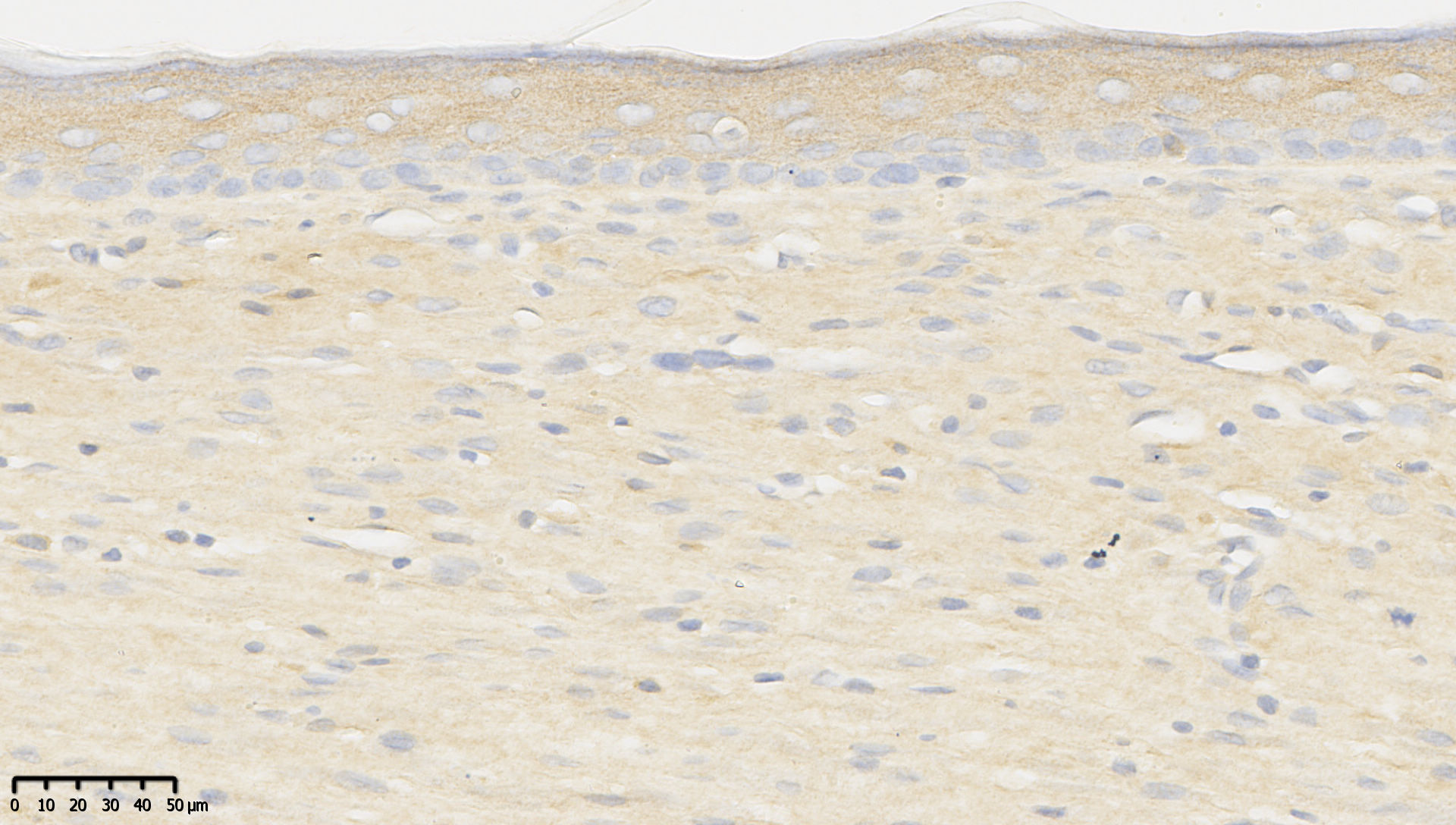

Supplement: S1 File — (ZIP) [file pone.0324264.s001.zip › supplement.material-1/Immunohistochemistry image/pan-cytokeratin/control-1113.jpg]

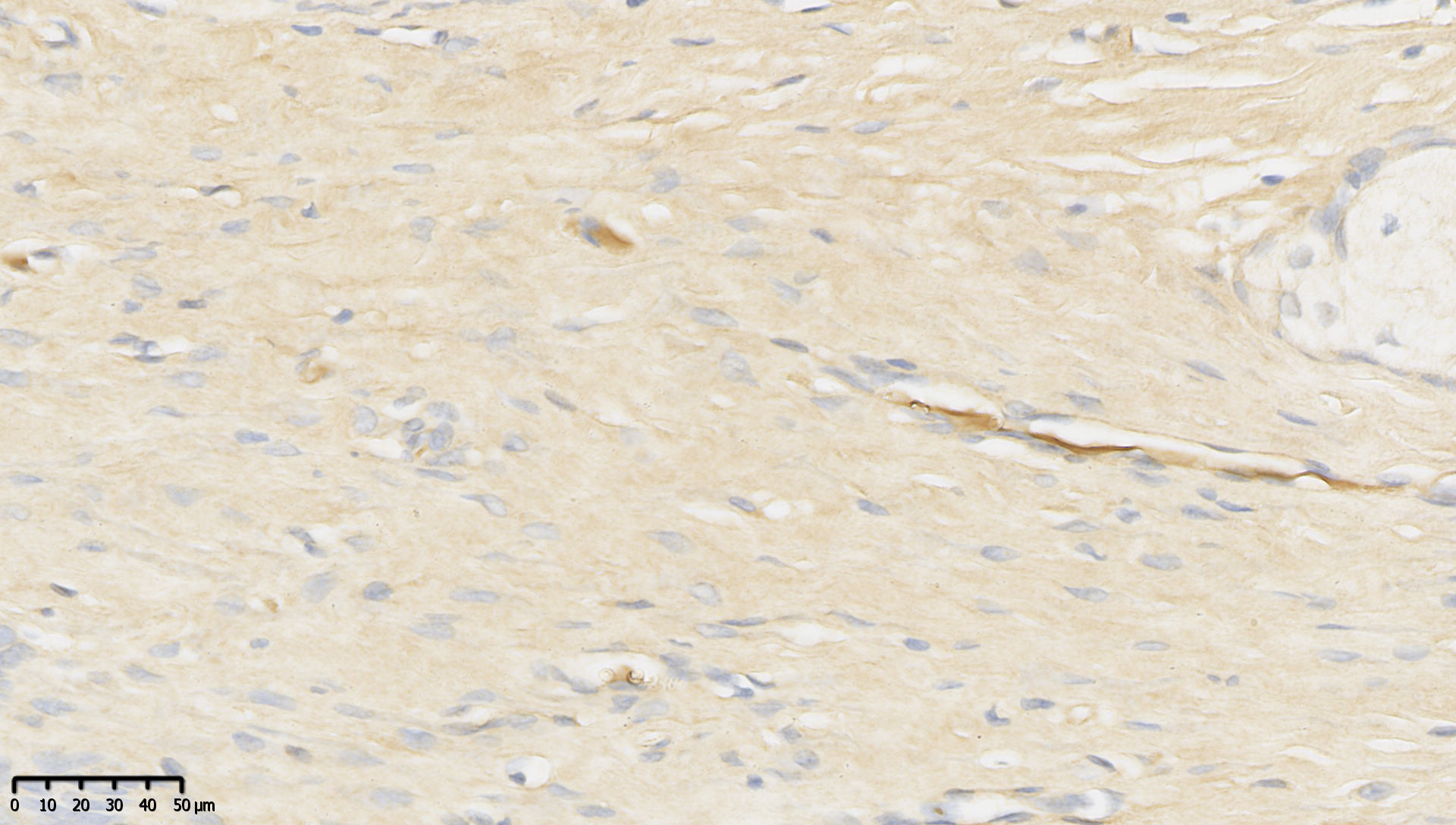

Supplement: S1 File — (ZIP) [file pone.0324264.s001.zip › supplement.material-1/Immunohistochemistry image/pan-cytokeratin/control-1114.jpg]

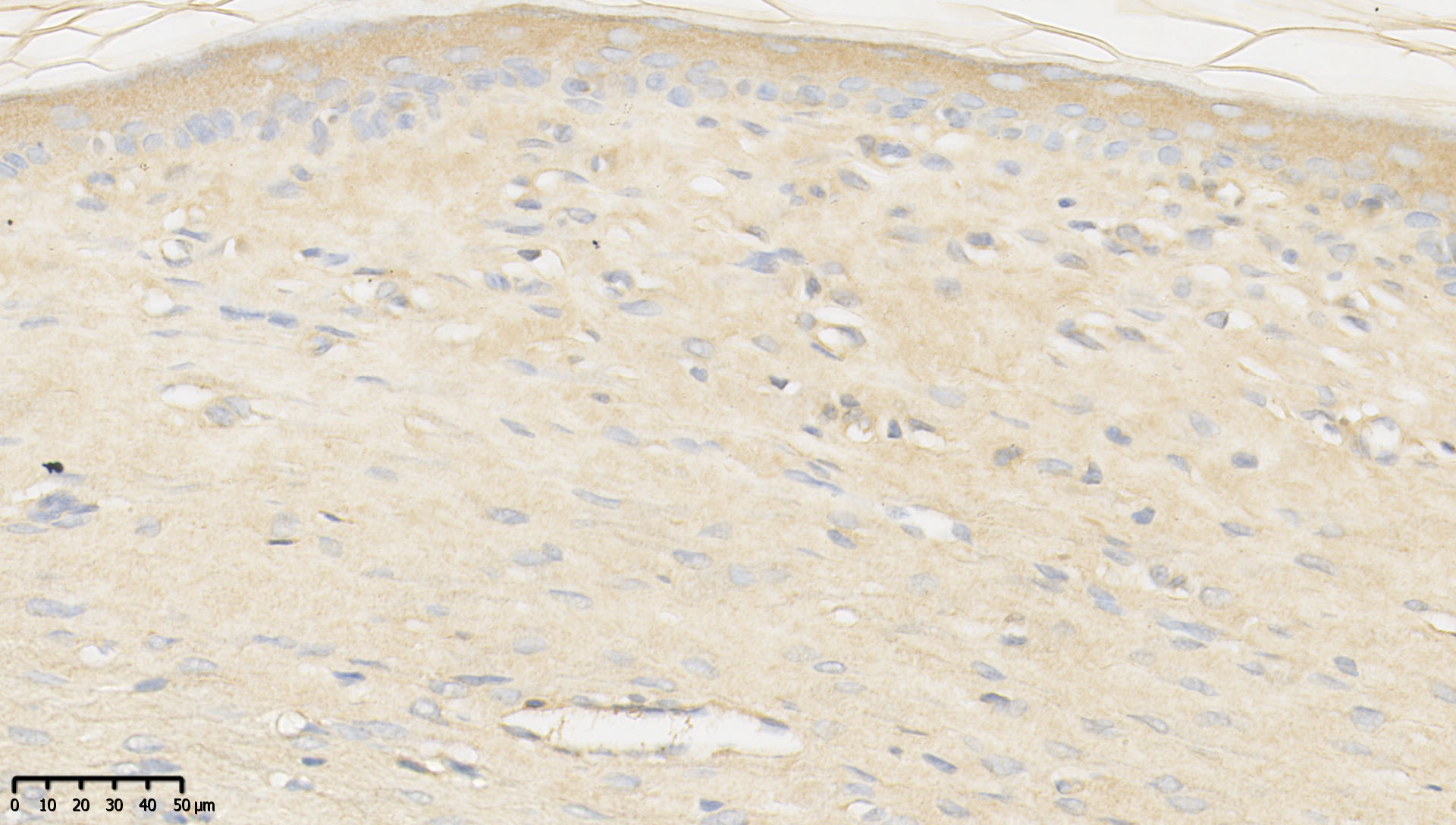

Supplement: S1 File — (ZIP) [file pone.0324264.s001.zip › supplement.material-1/Immunohistochemistry image/pan-cytokeratin/control-1115.jpg]

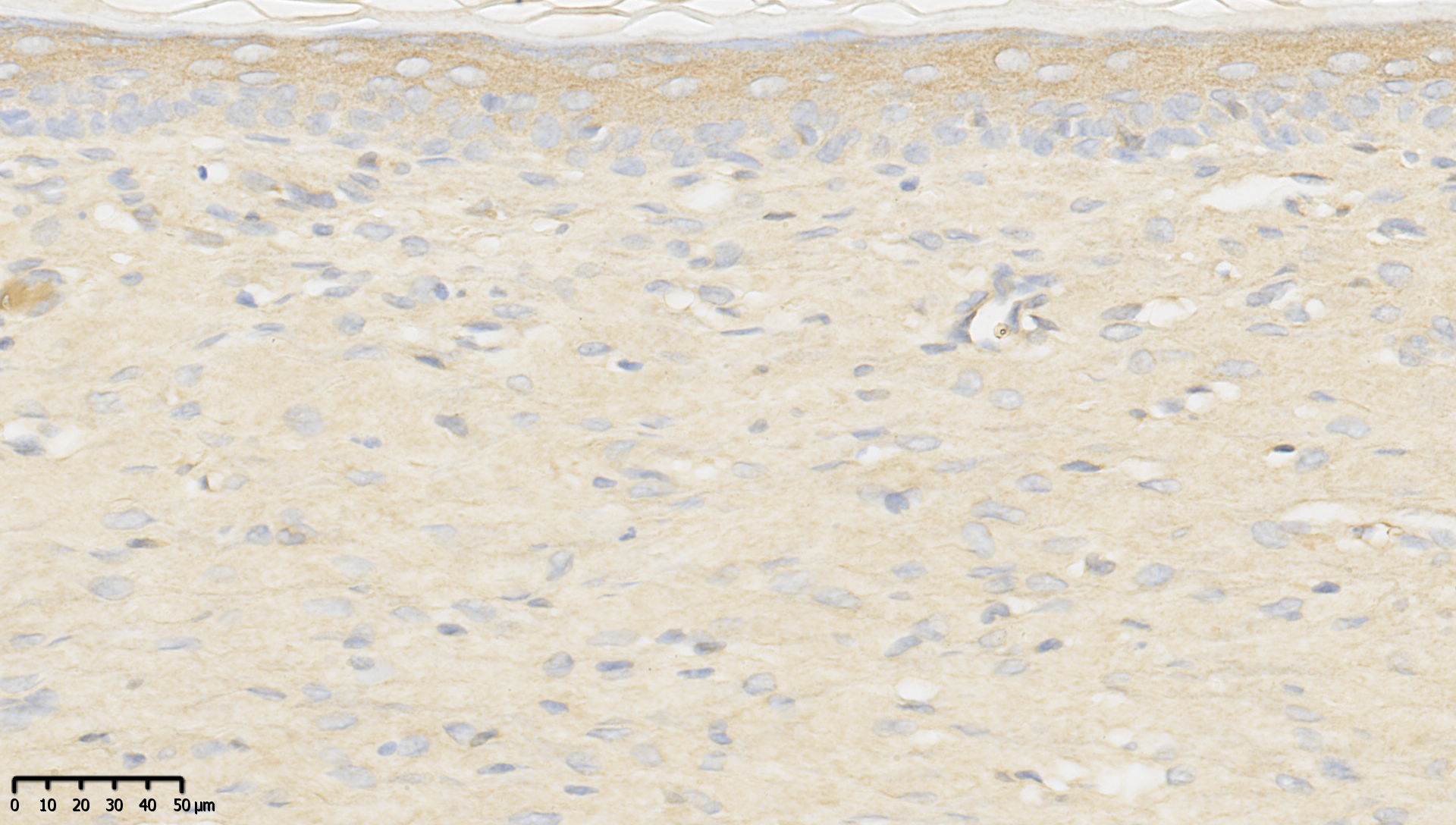

Supplement: S1 File — (ZIP) [file pone.0324264.s001.zip › supplement.material-1/Immunohistochemistry image/pan-cytokeratin/control-1116.jpg]

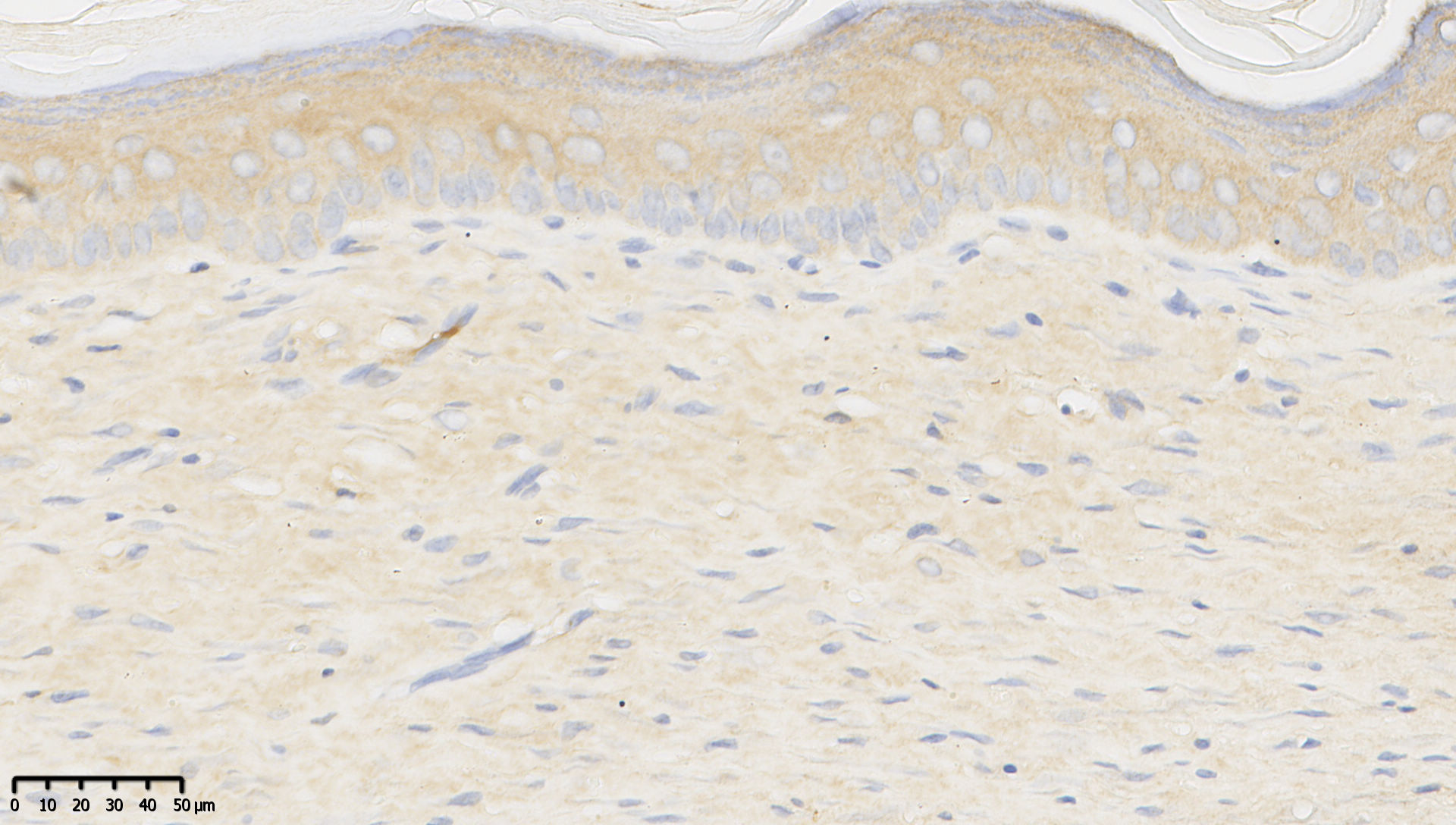

Supplement: S1 File — (ZIP) [file pone.0324264.s001.zip › supplement.material-1/Immunohistochemistry image/pan-cytokeratin/HA-1111.jpg]

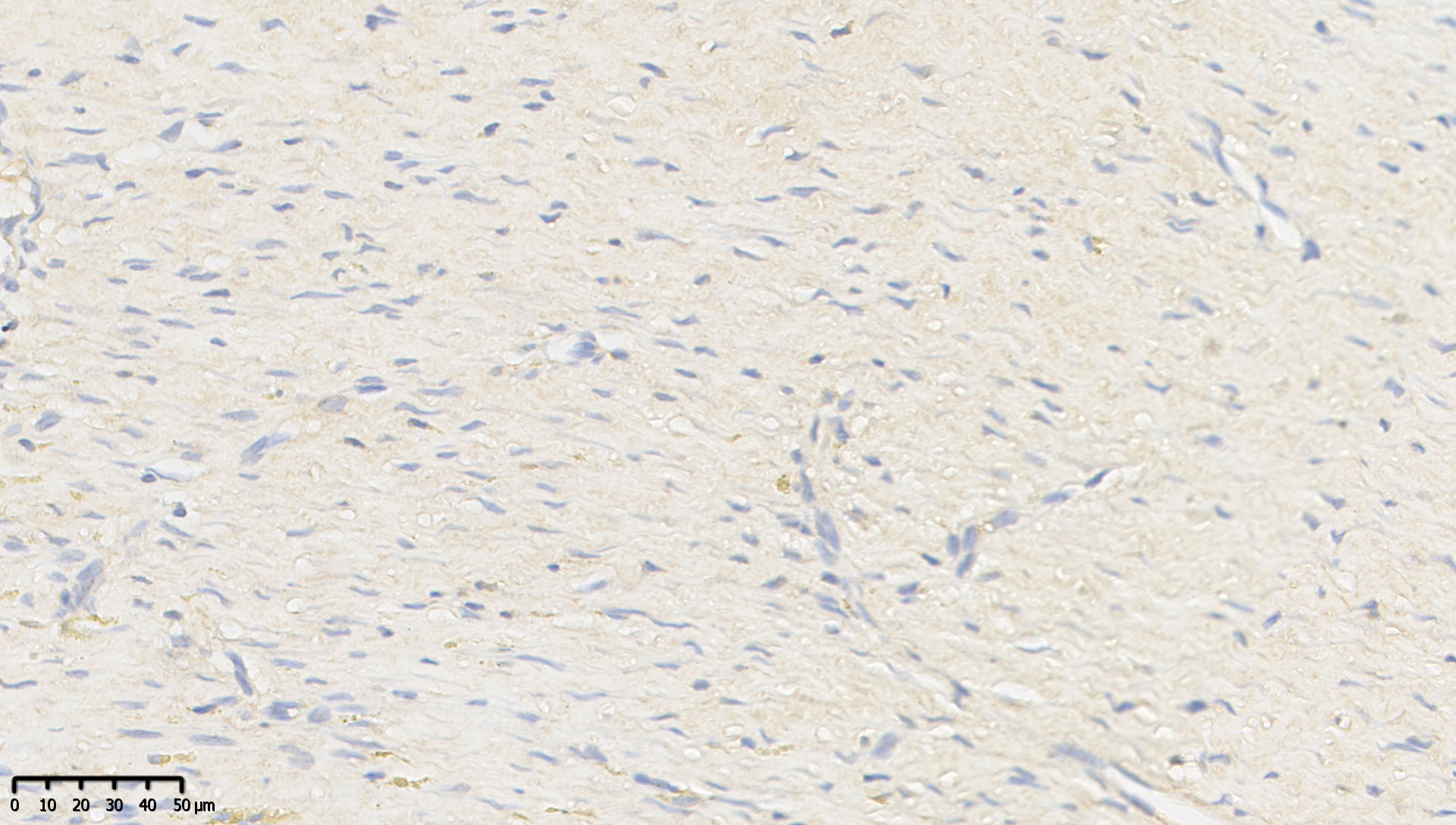

Supplement: S1 File — (ZIP) [file pone.0324264.s001.zip › supplement.material-1/Immunohistochemistry image/pan-cytokeratin/HA-1112.jpg]

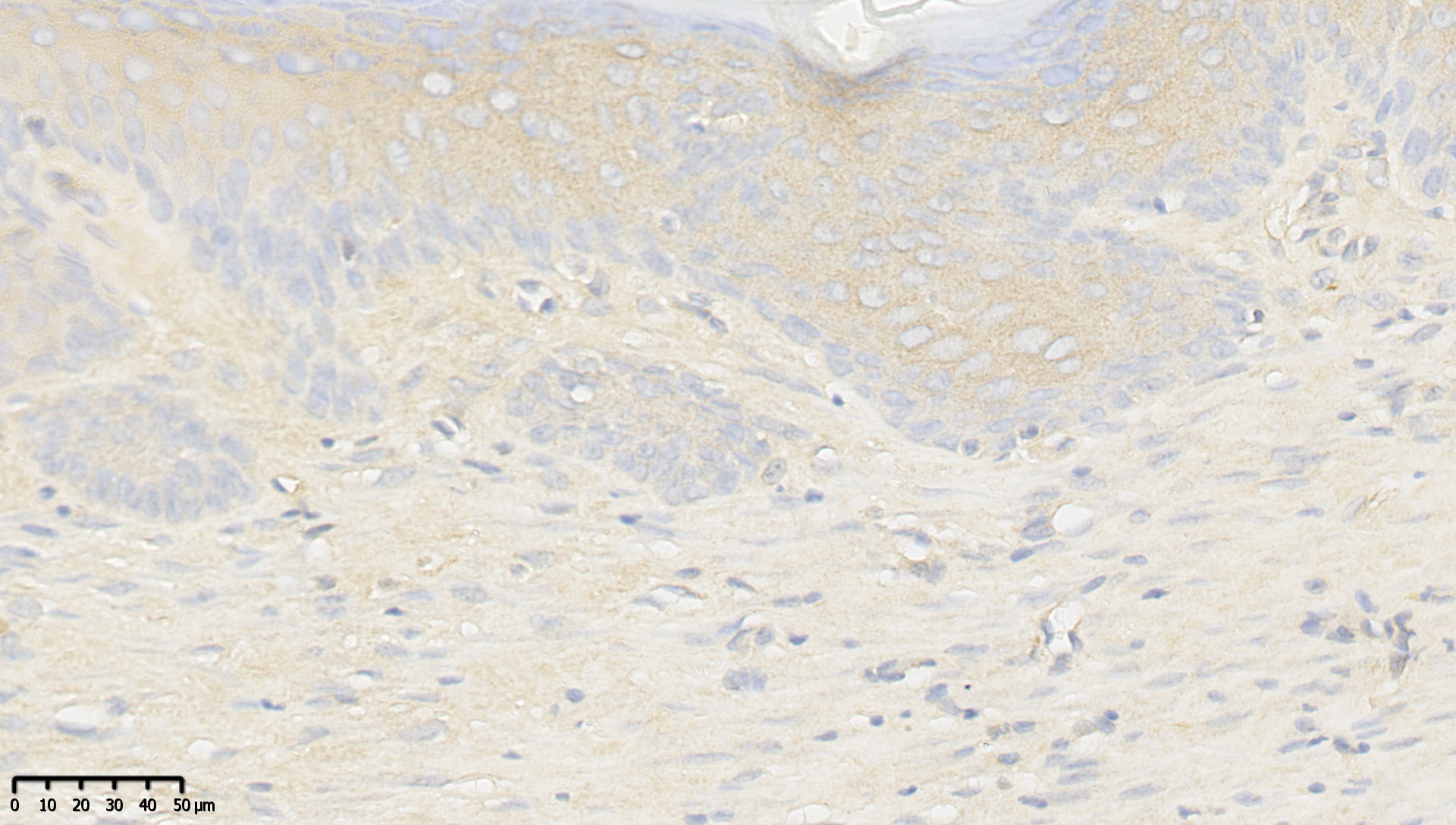

Supplement: S1 File — (ZIP) [file pone.0324264.s001.zip › supplement.material-1/Immunohistochemistry image/pan-cytokeratin/HA-1113.jpg]

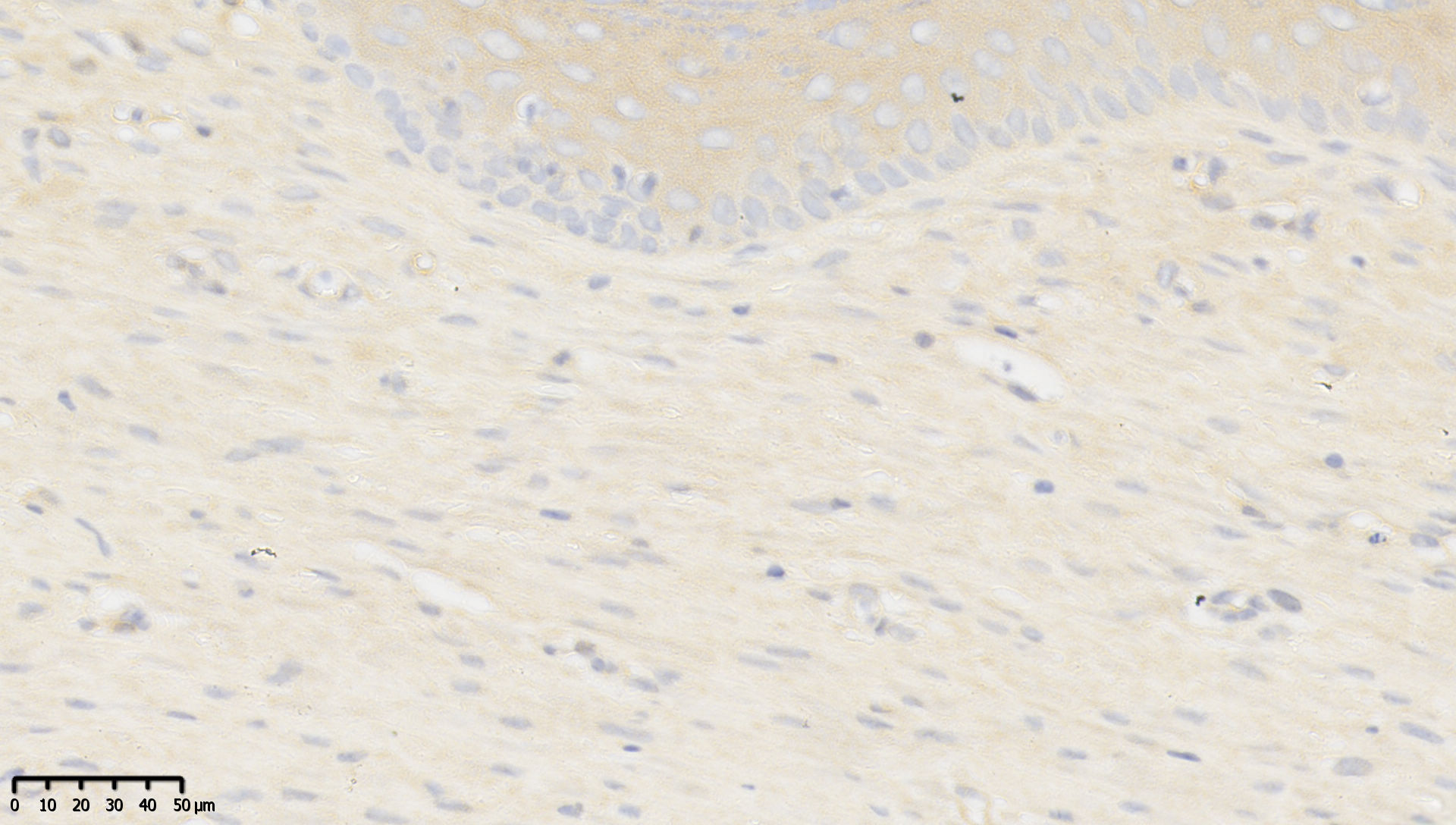

Supplement: S1 File — (ZIP) [file pone.0324264.s001.zip › supplement.material-1/Immunohistochemistry image/pan-cytokeratin/HA-1114.jpg]

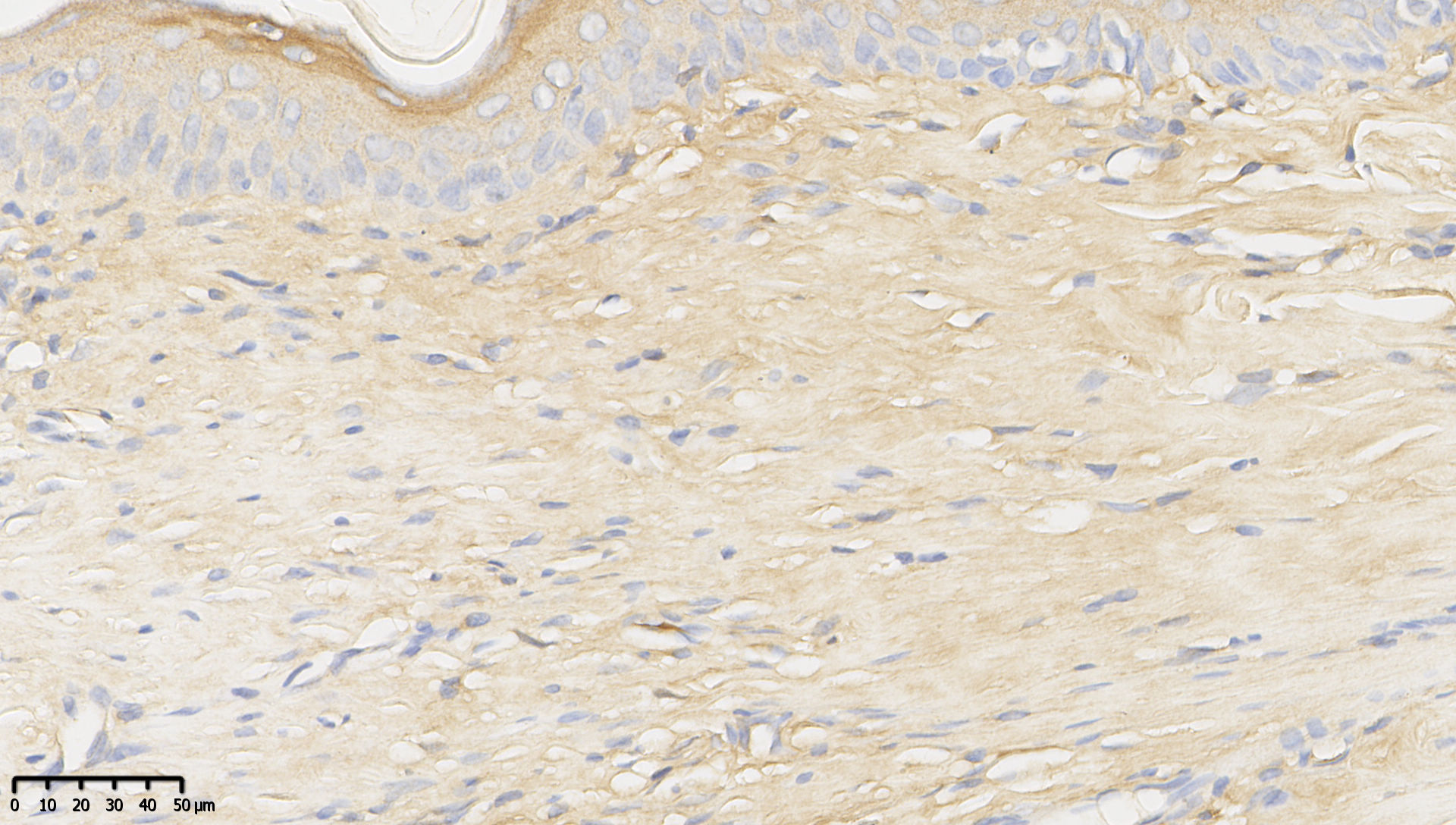

Supplement: S1 File — (ZIP) [file pone.0324264.s001.zip › supplement.material-1/Immunohistochemistry image/pan-cytokeratin/HA-1115.jpg]

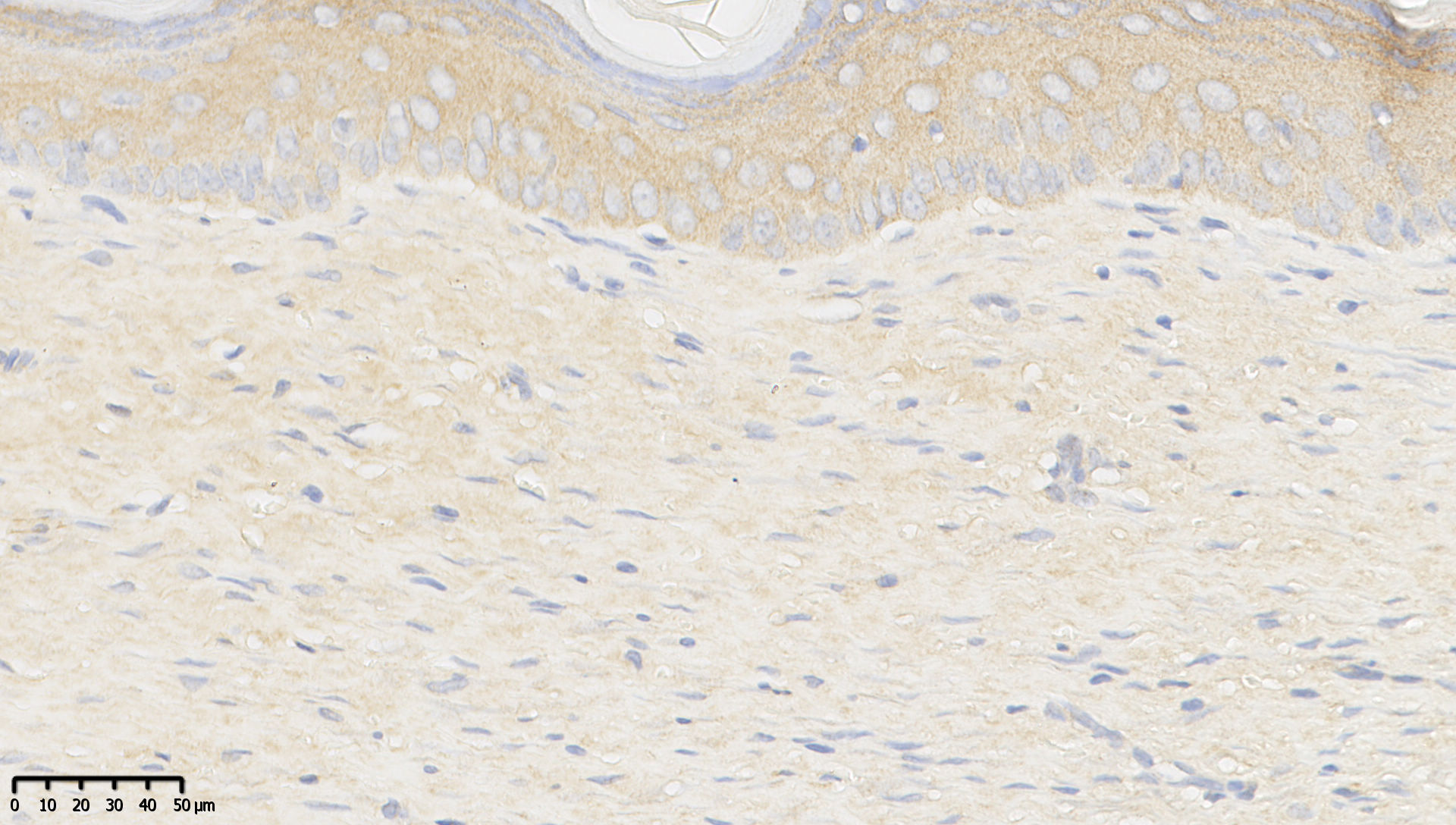

Supplement: S1 File — (ZIP) [file pone.0324264.s001.zip › supplement.material-1/Immunohistochemistry image/pan-cytokeratin/HA-1116.jpg]

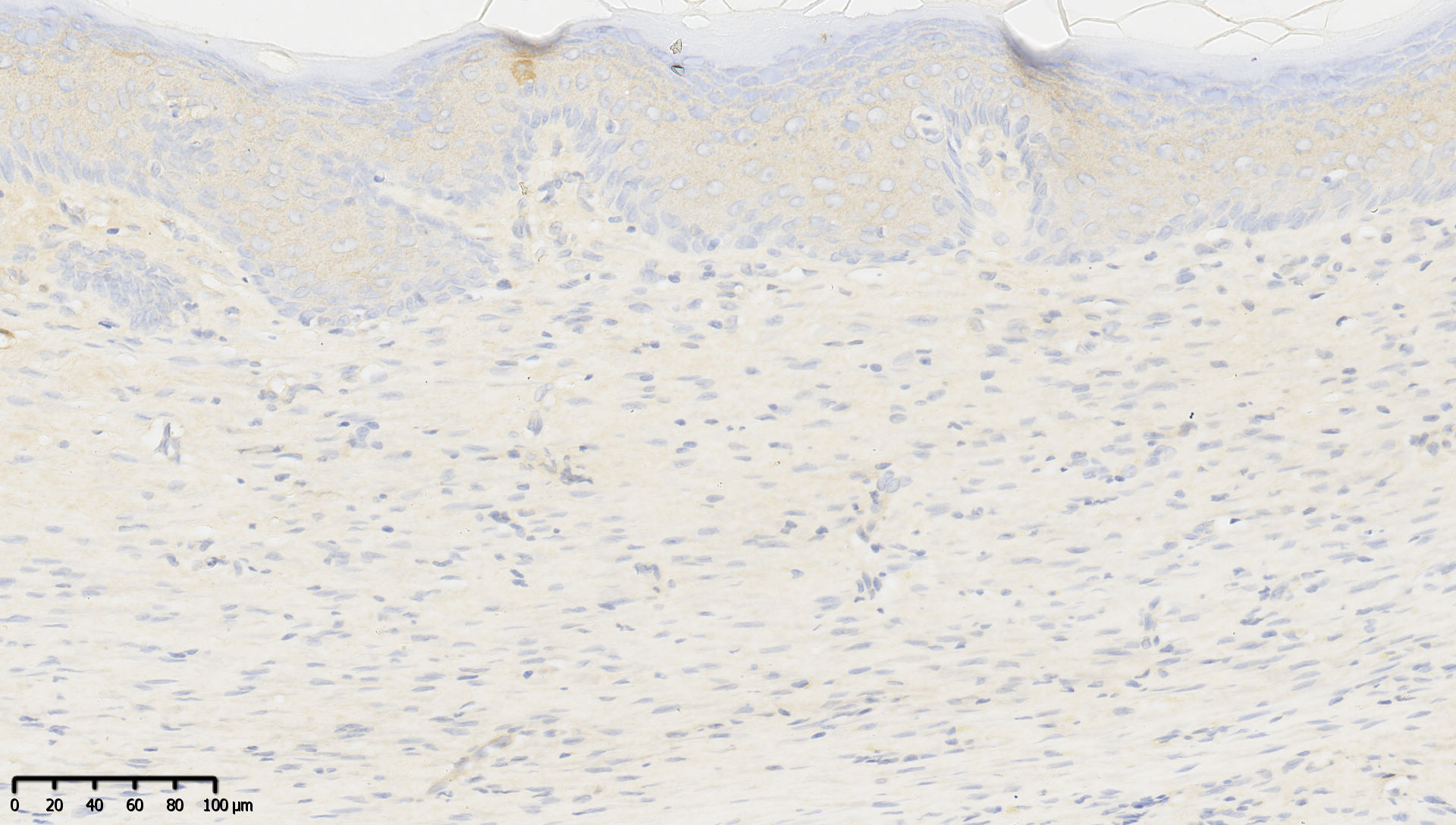

Supplement: S1 File — (ZIP) [file pone.0324264.s001.zip › supplement.material-1/Immunohistochemistry image/pan-cytokeratin/model-1111.jpg]

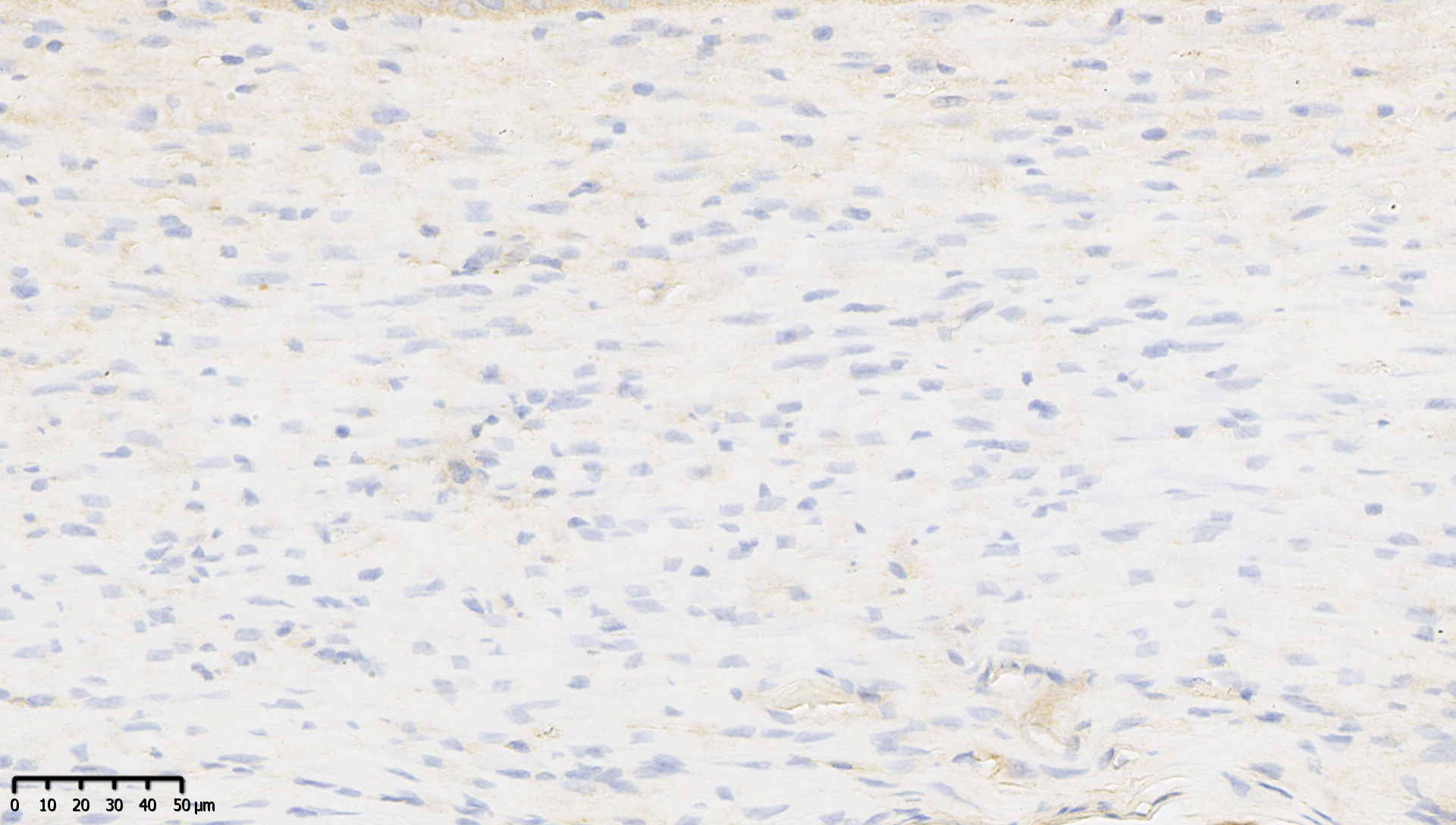

Supplement: S1 File — (ZIP) [file pone.0324264.s001.zip › supplement.material-1/Immunohistochemistry image/pan-cytokeratin/model-1112.jpg]

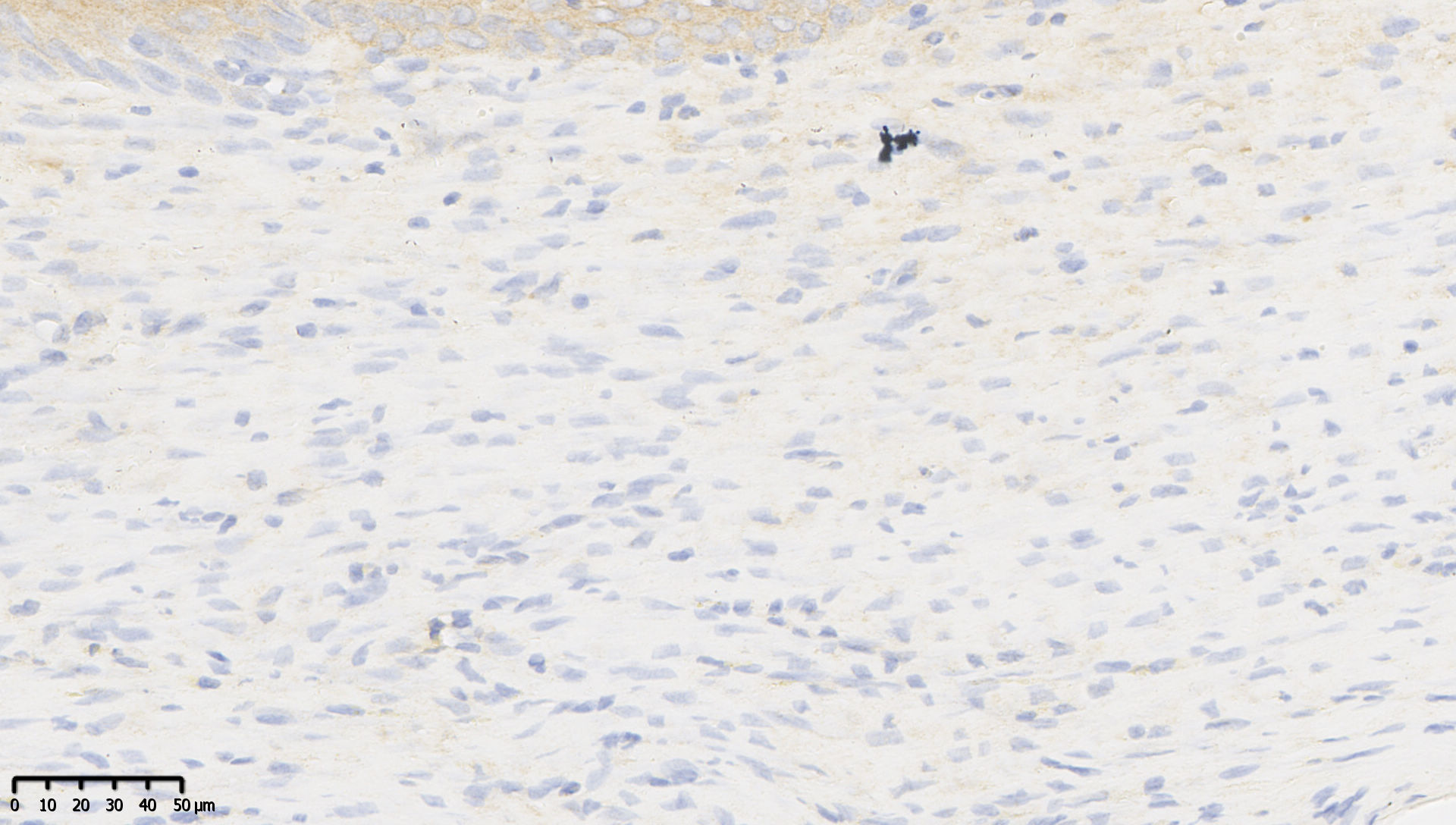

Supplement: S1 File — (ZIP) [file pone.0324264.s001.zip › supplement.material-1/Immunohistochemistry image/pan-cytokeratin/model-1113.jpg]

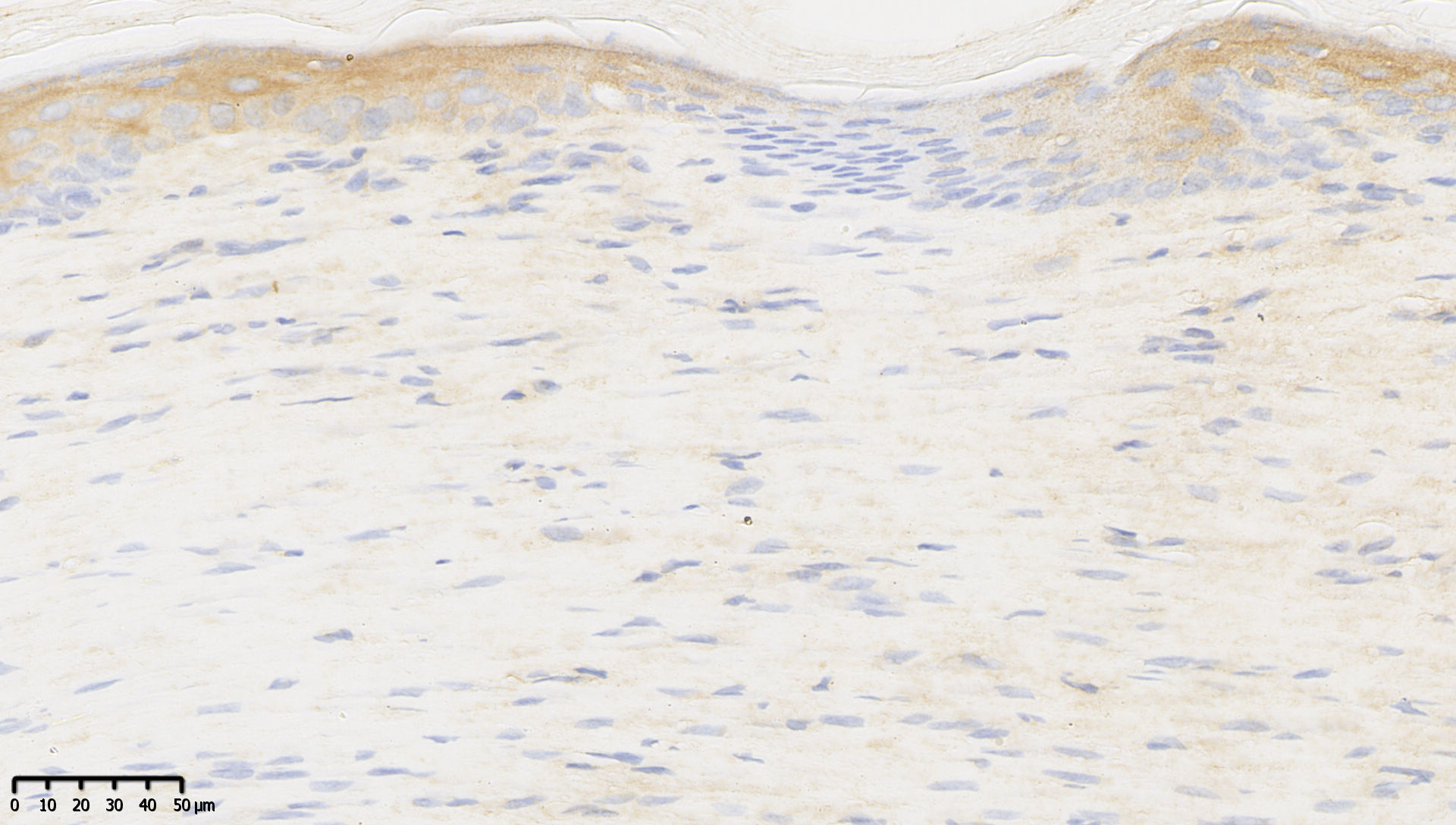

Supplement: S1 File — (ZIP) [file pone.0324264.s001.zip › supplement.material-1/Immunohistochemistry image/pan-cytokeratin/model-1114.jpg]

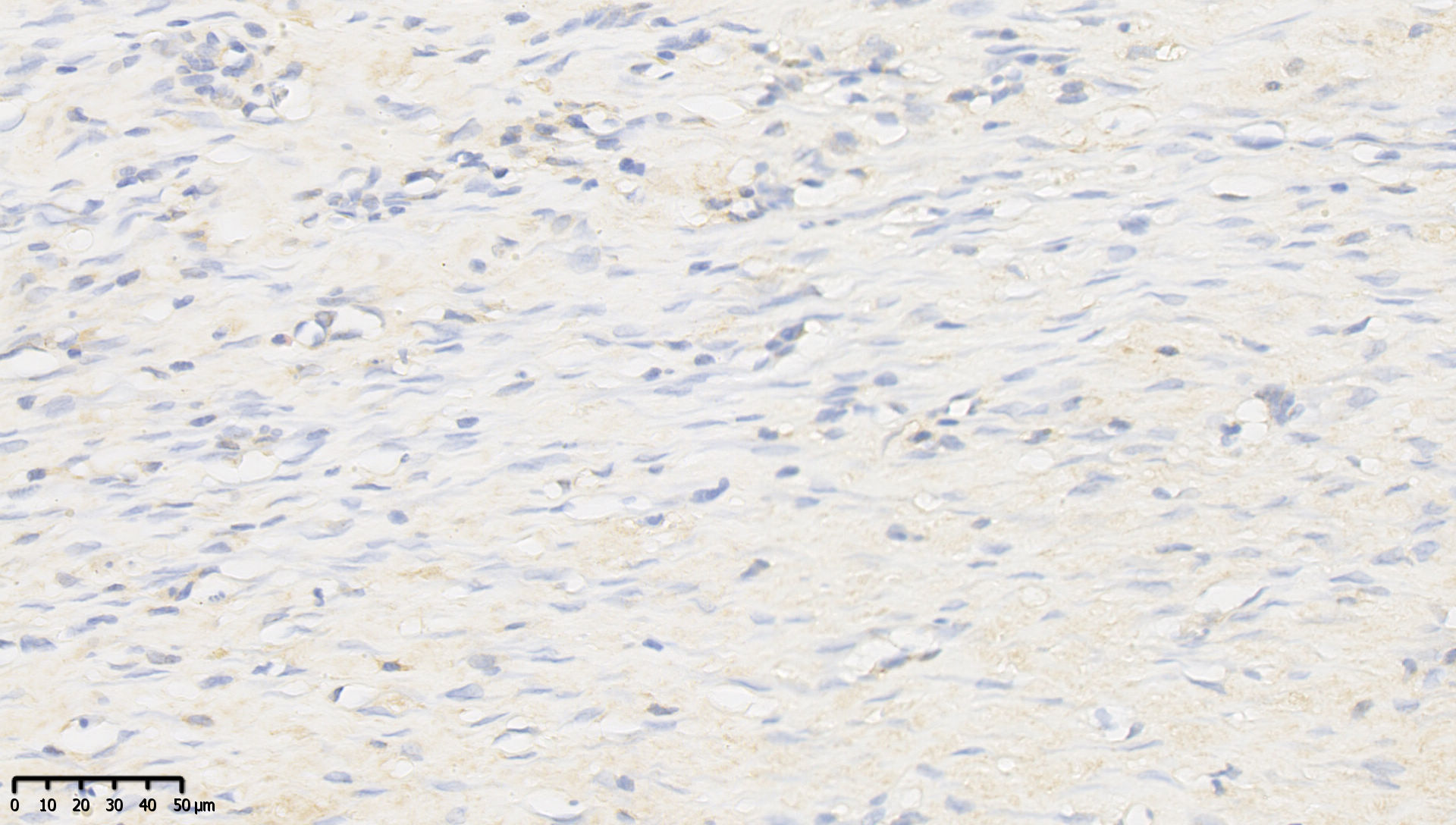

Supplement: S1 File — (ZIP) [file pone.0324264.s001.zip › supplement.material-1/Immunohistochemistry image/pan-cytokeratin/model-1115.jpg]

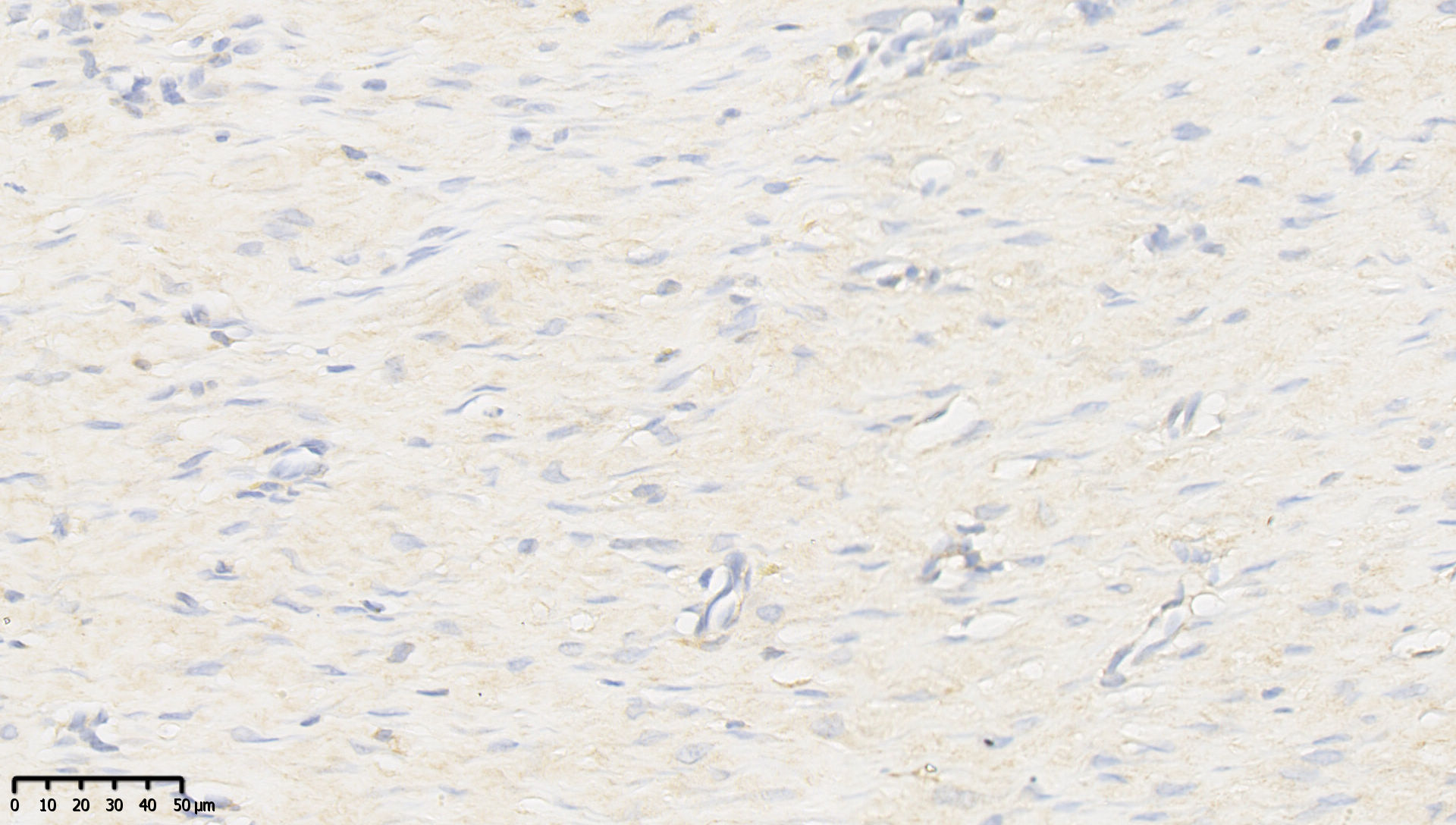

Supplement: S1 File — (ZIP) [file pone.0324264.s001.zip › supplement.material-1/Immunohistochemistry image/pan-cytokeratin/model-1116.jpg]

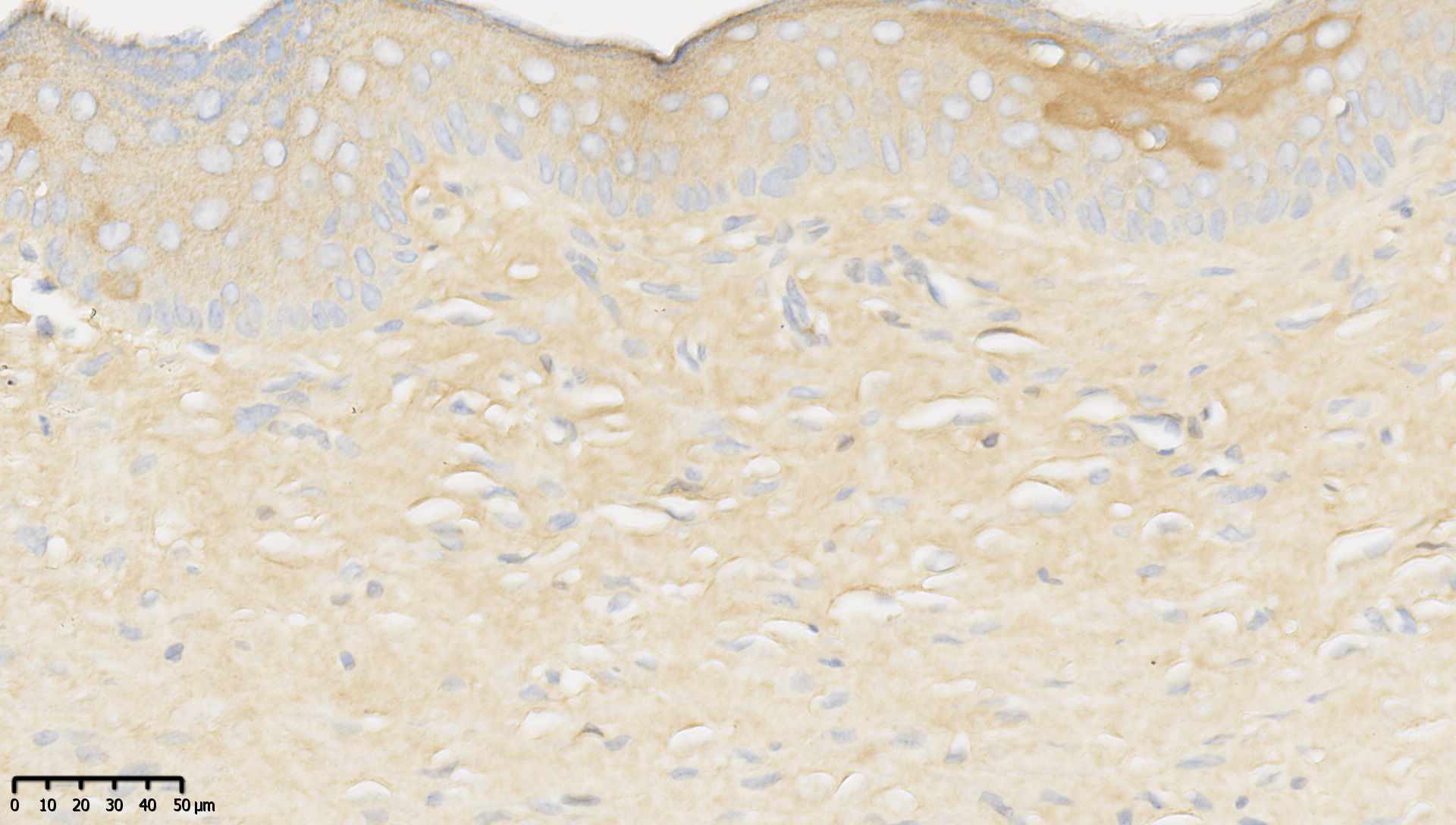

Supplement: S1 File — (ZIP) [file pone.0324264.s001.zip › supplement.material-1/Immunohistochemistry image/pan-cytokeratin/PL-HA-1111.jpg]

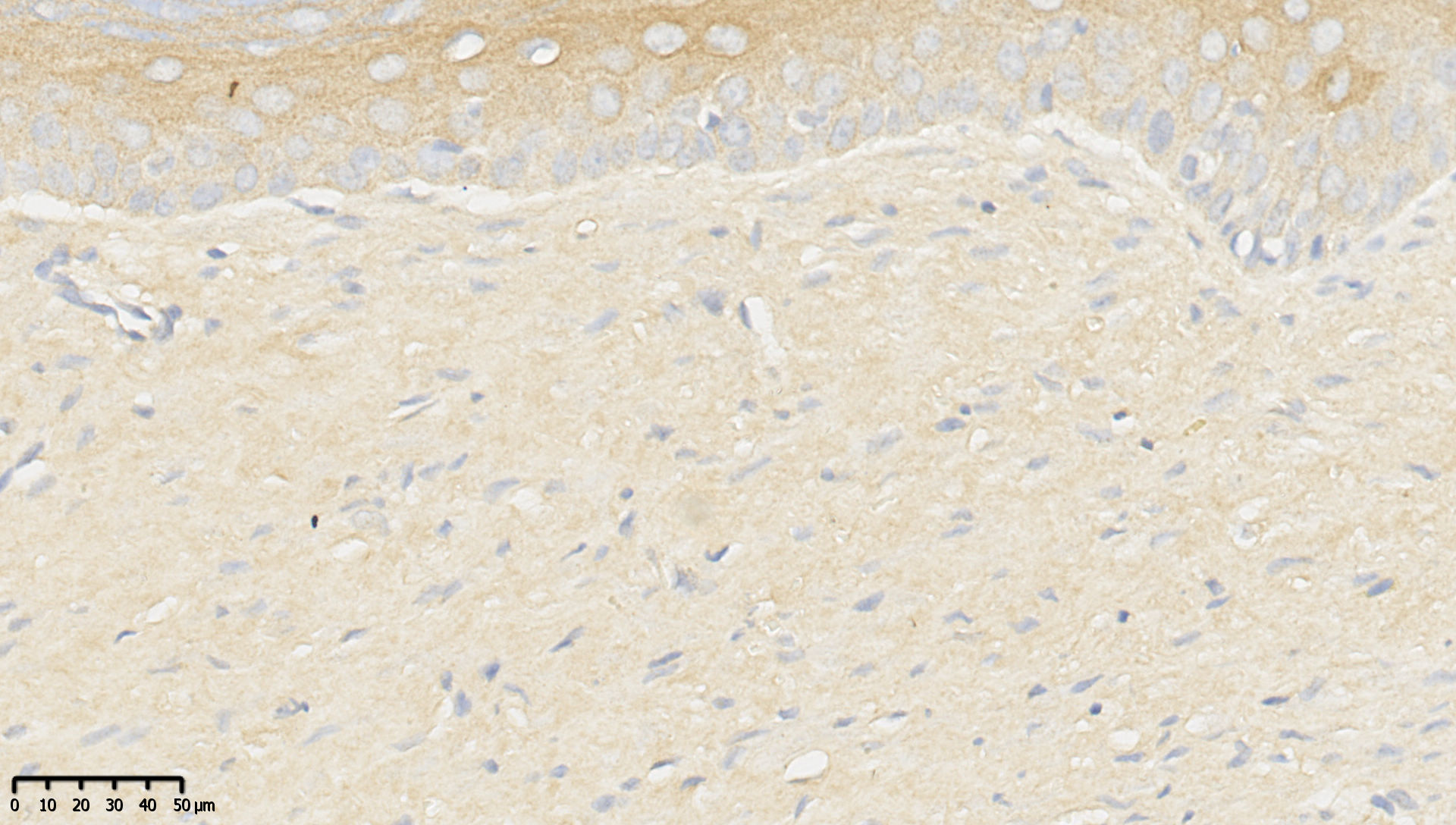

Supplement: S1 File — (ZIP) [file pone.0324264.s001.zip › supplement.material-1/Immunohistochemistry image/pan-cytokeratin/PL-HA-1112.jpg]

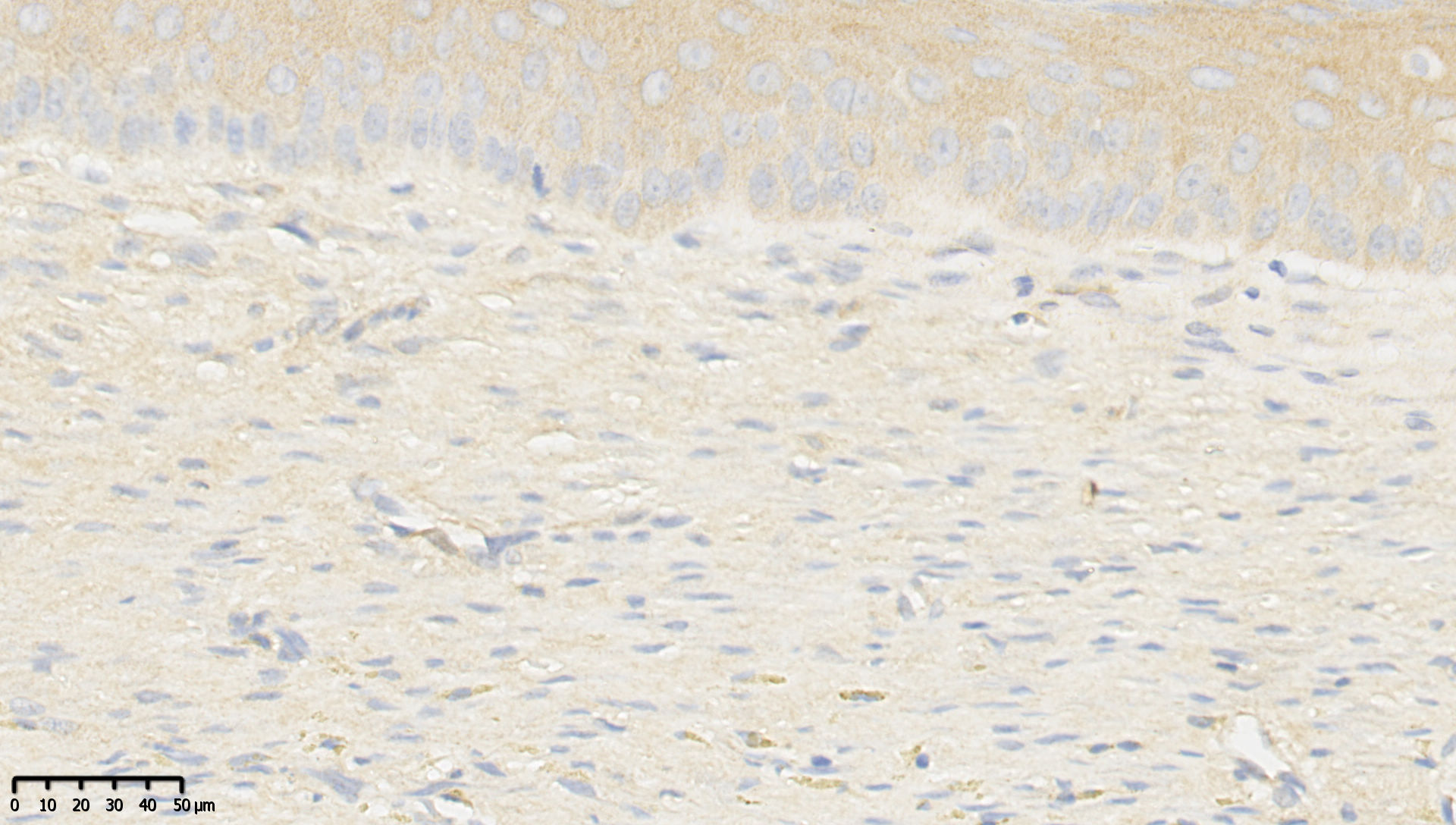

Supplement: S1 File — (ZIP) [file pone.0324264.s001.zip › supplement.material-1/Immunohistochemistry image/pan-cytokeratin/PL-HA-1113.jpg]

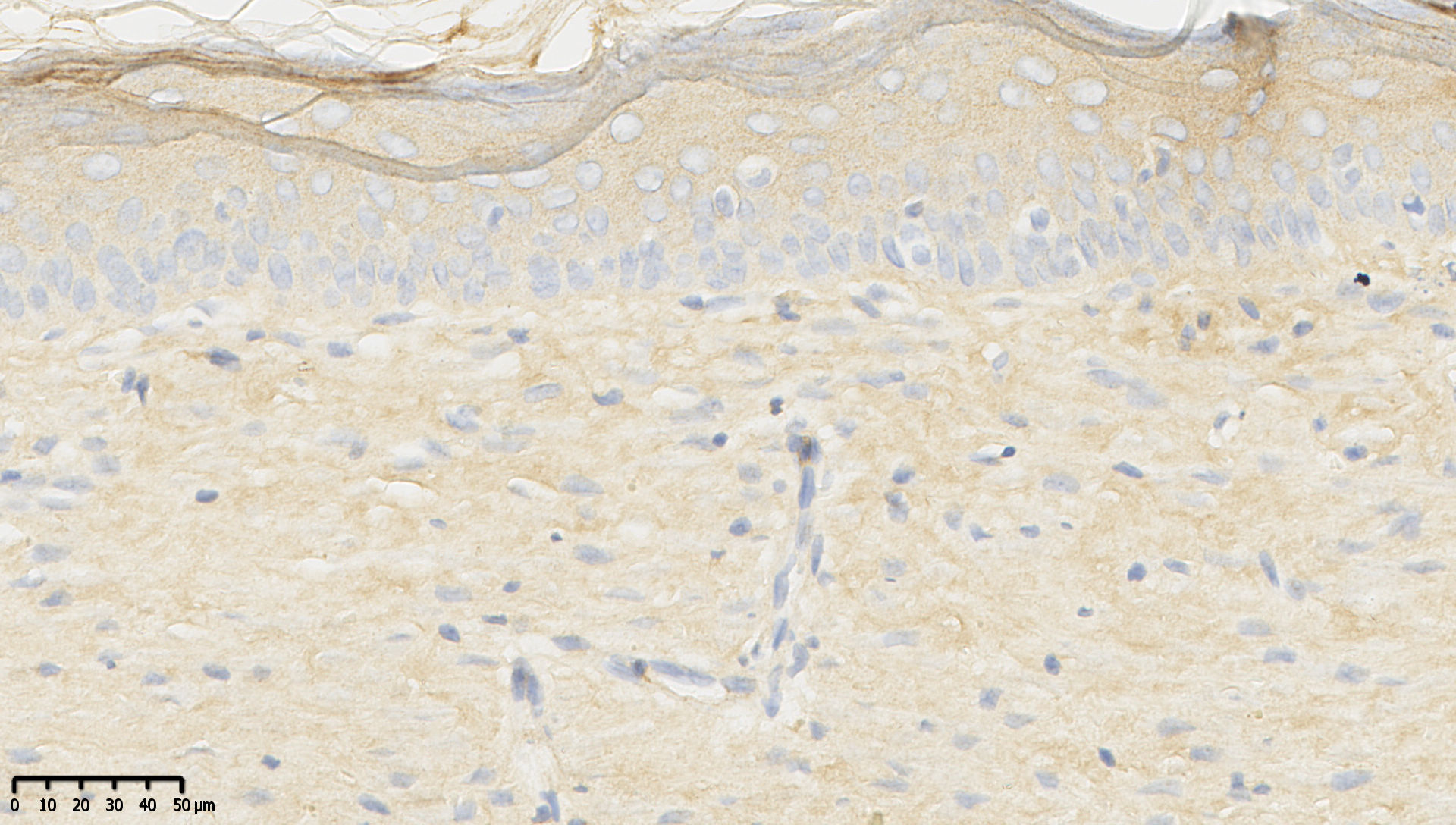

Supplement: S1 File — (ZIP) [file pone.0324264.s001.zip › supplement.material-1/Immunohistochemistry image/pan-cytokeratin/PL-HA-1114.jpg]

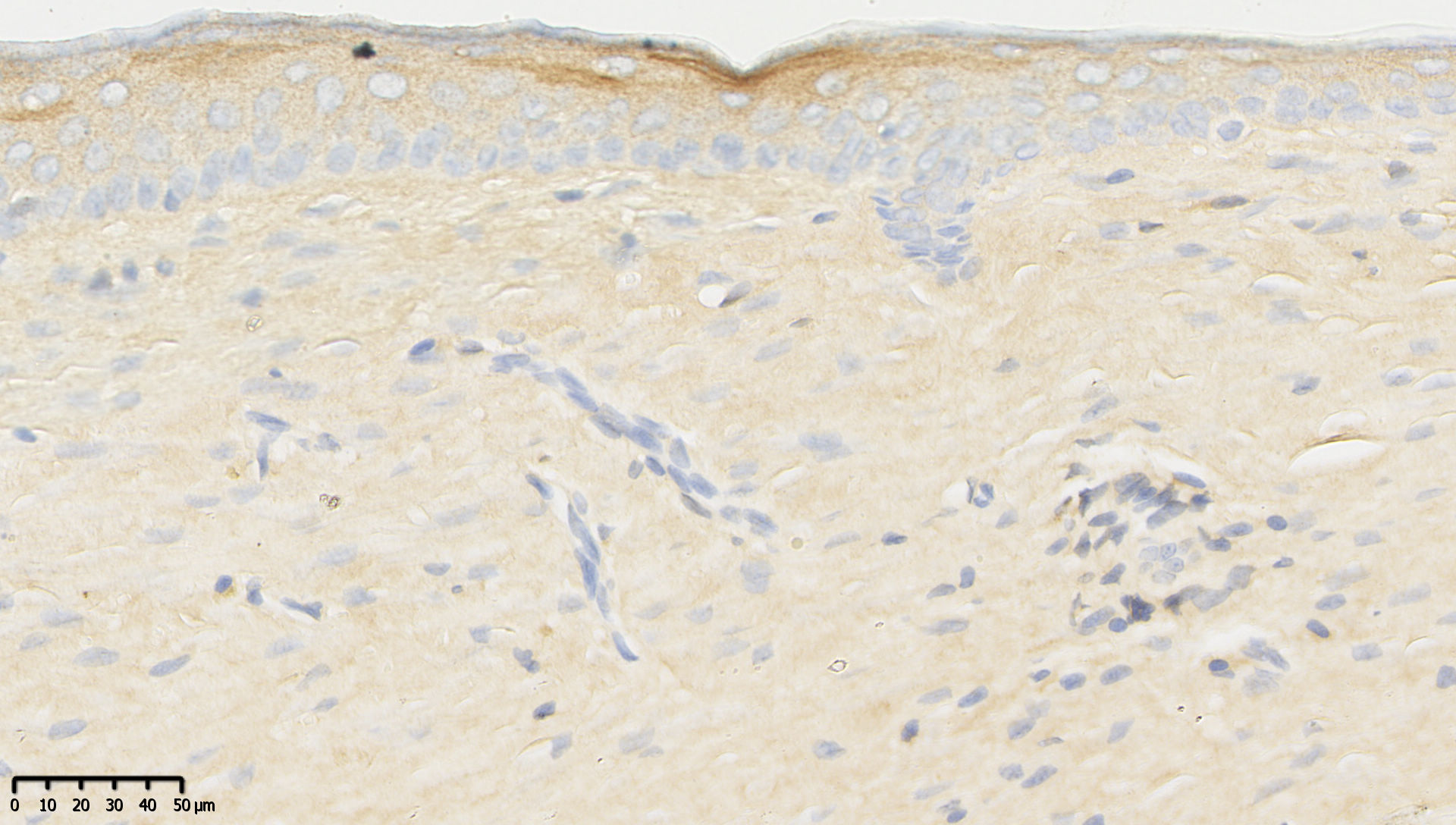

Supplement: S1 File — (ZIP) [file pone.0324264.s001.zip › supplement.material-1/Immunohistochemistry image/pan-cytokeratin/PL-HA-1115.jpg]

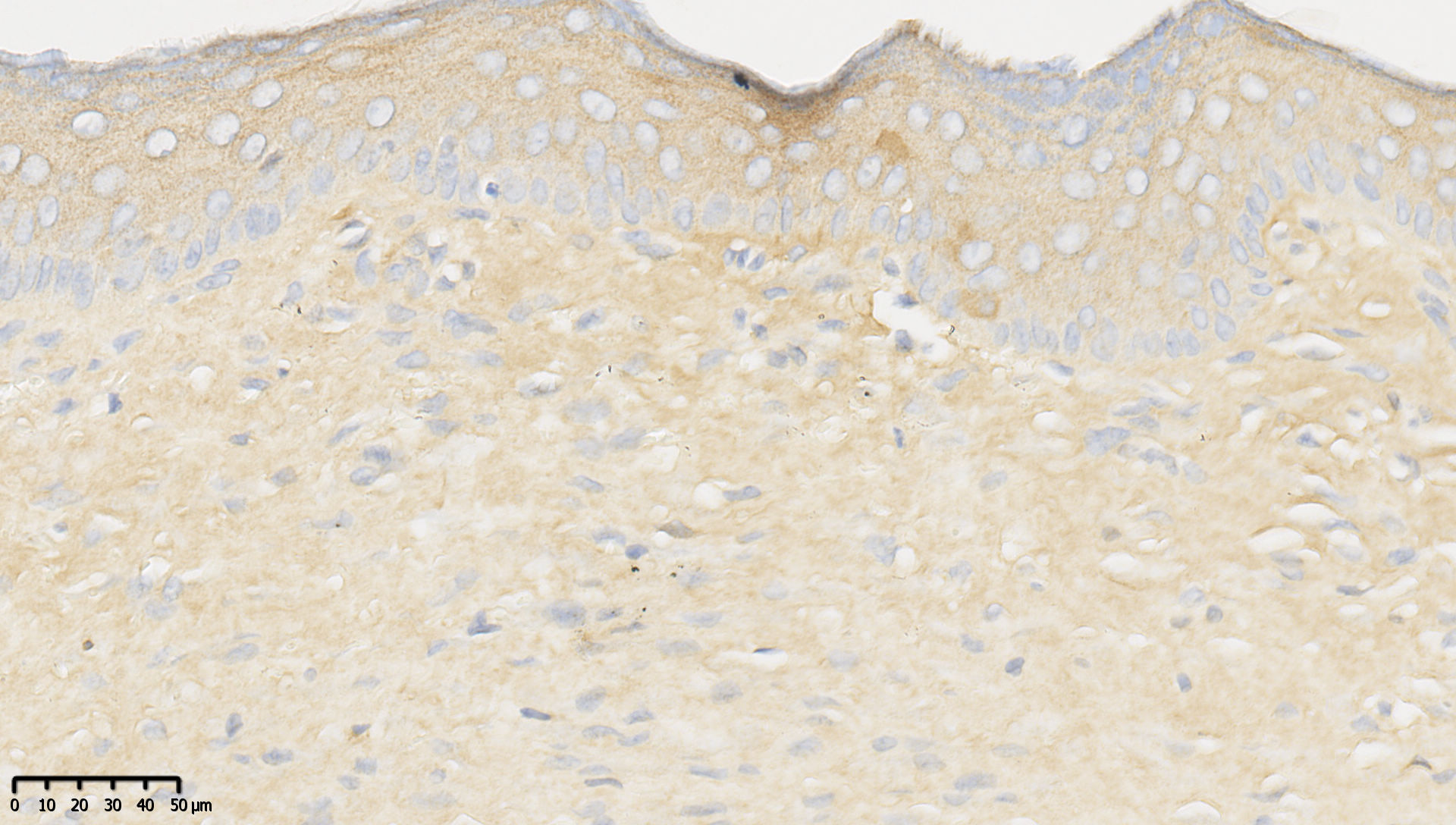

Supplement: S1 File — (ZIP) [file pone.0324264.s001.zip › supplement.material-1/Immunohistochemistry image/pan-cytokeratin/PL-HA-1116.jpg]

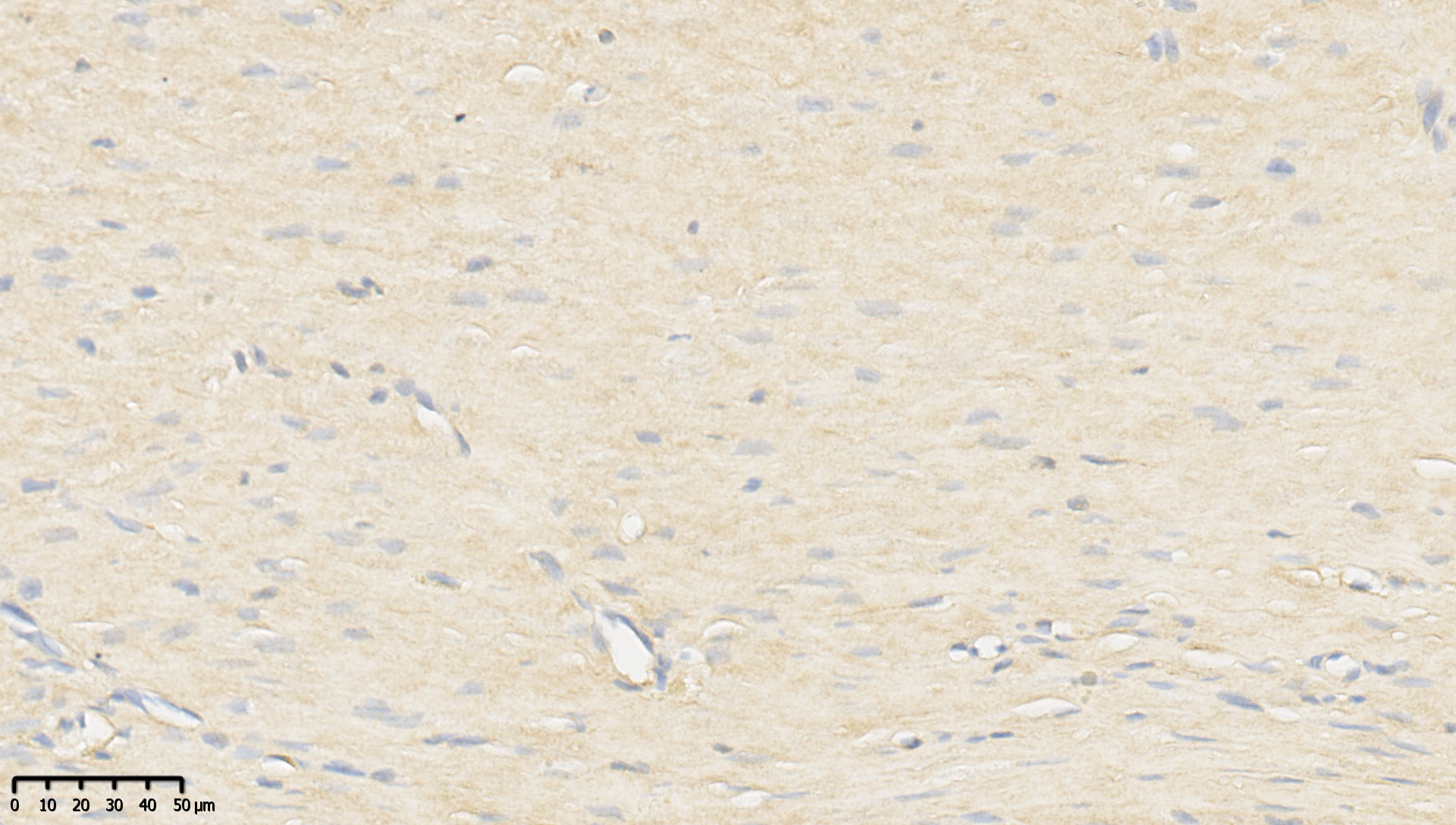

Supplement: S1 File — (ZIP) [file pone.0324264.s001.zip › supplement.material-1/Immunohistochemistry image/pan-cytokeratin/PL-HA-1117.jpg]

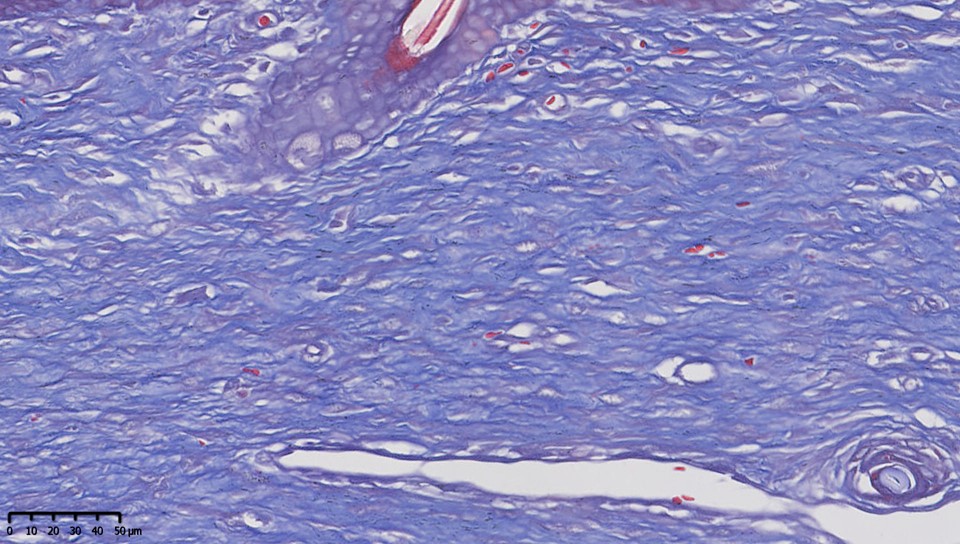

Supplement: S1 File — (ZIP) [file pone.0324264.s001.zip › supplement.material-1/Masson triple section image/control-11 400x.jpg]

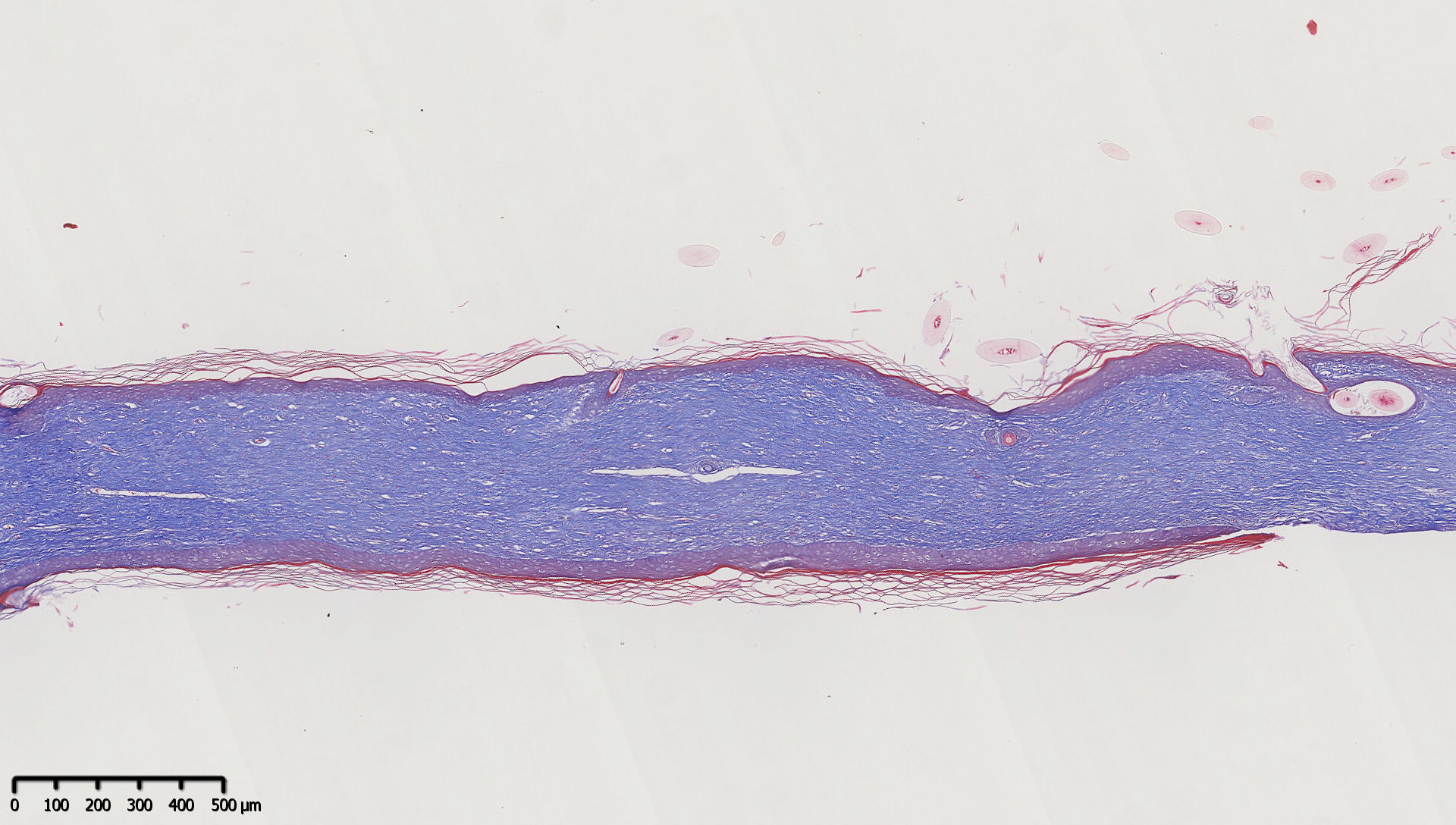

Supplement: S1 File — (ZIP) [file pone.0324264.s001.zip › supplement.material-1/Masson triple section image/control-11 50x.jpg]

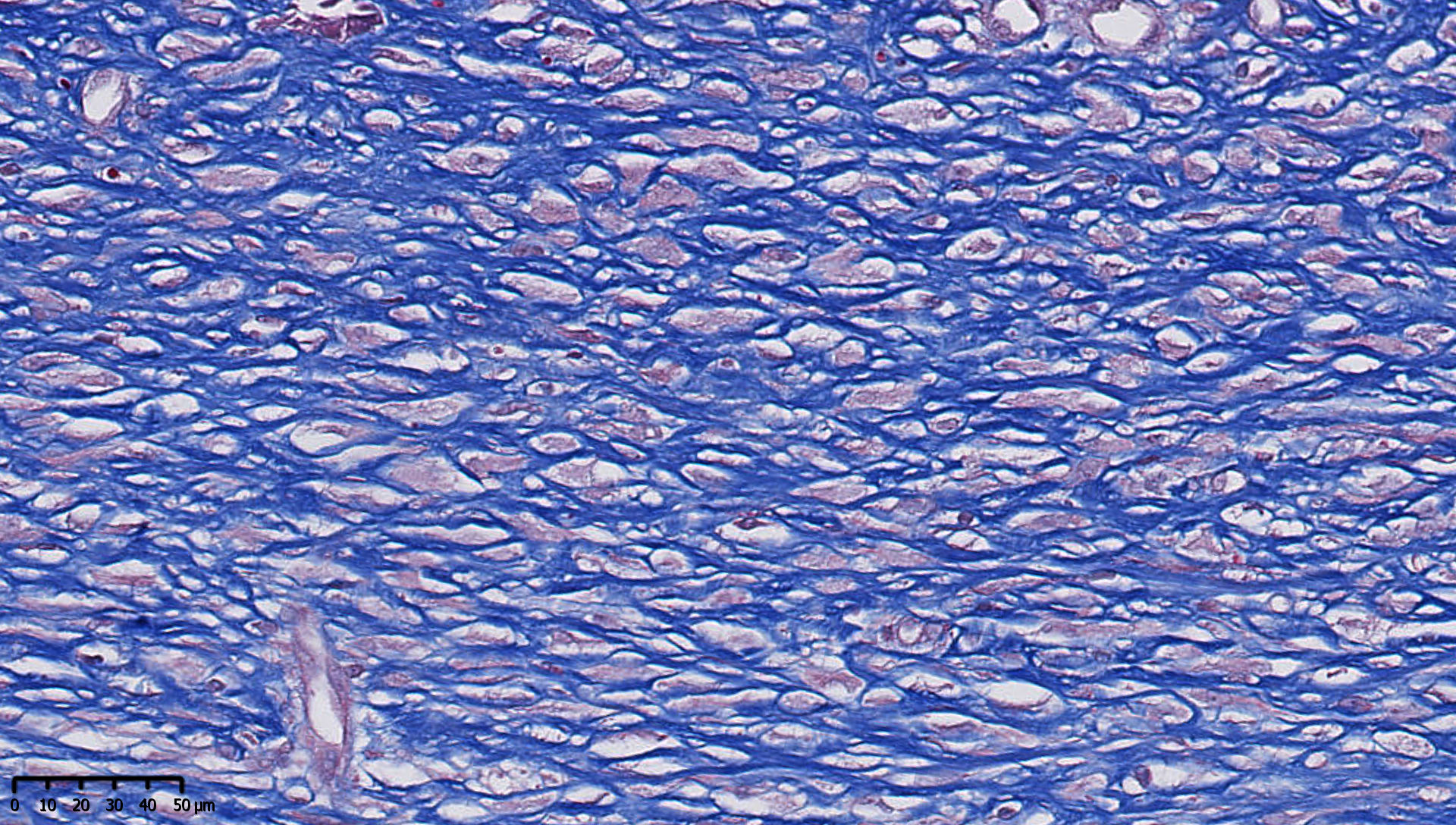

Supplement: S1 File — (ZIP) [file pone.0324264.s001.zip › supplement.material-1/Masson triple section image/control-12 400x.jpg]

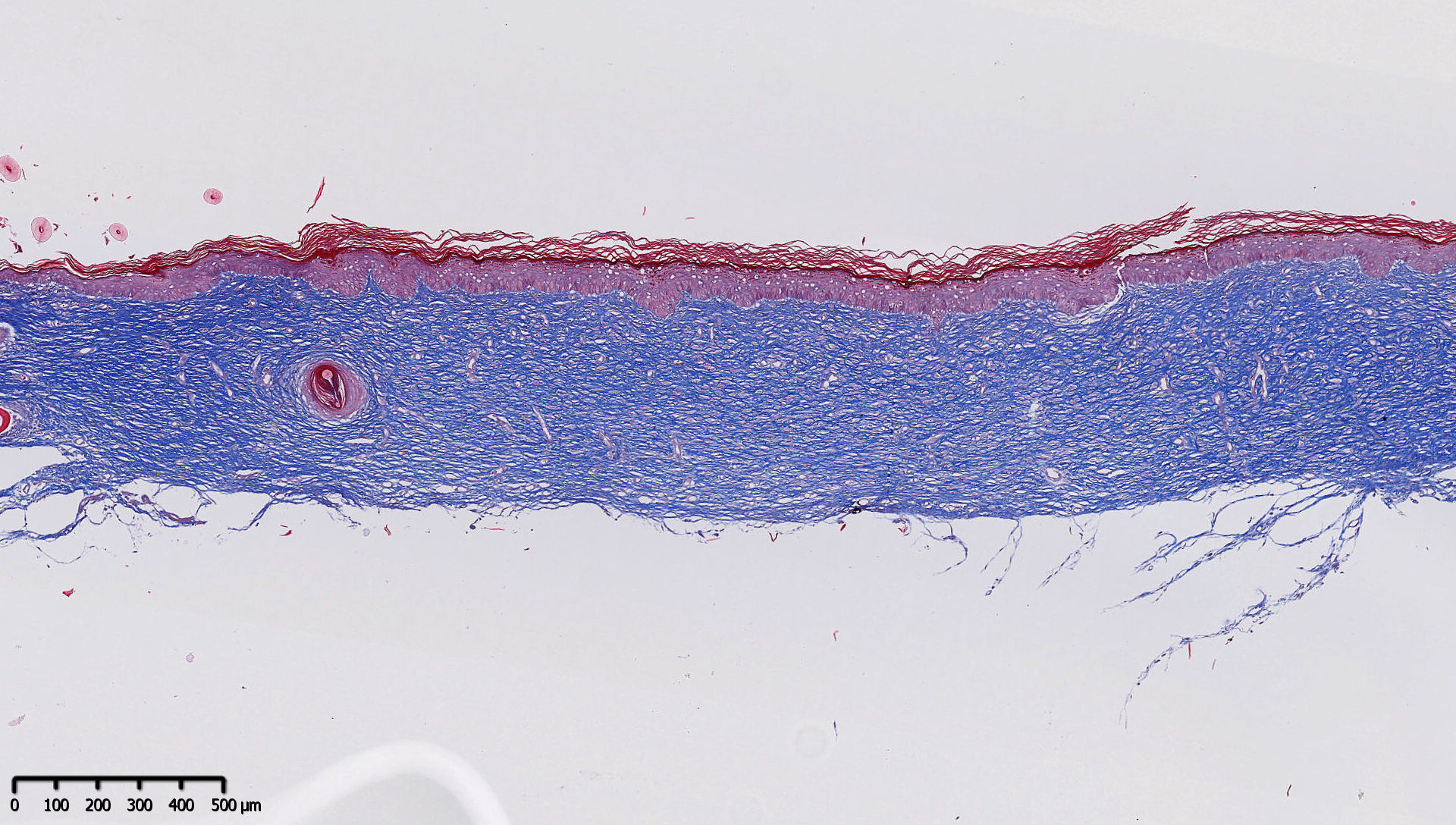

Supplement: S1 File — (ZIP) [file pone.0324264.s001.zip › supplement.material-1/Masson triple section image/control-12 50x.jpg]

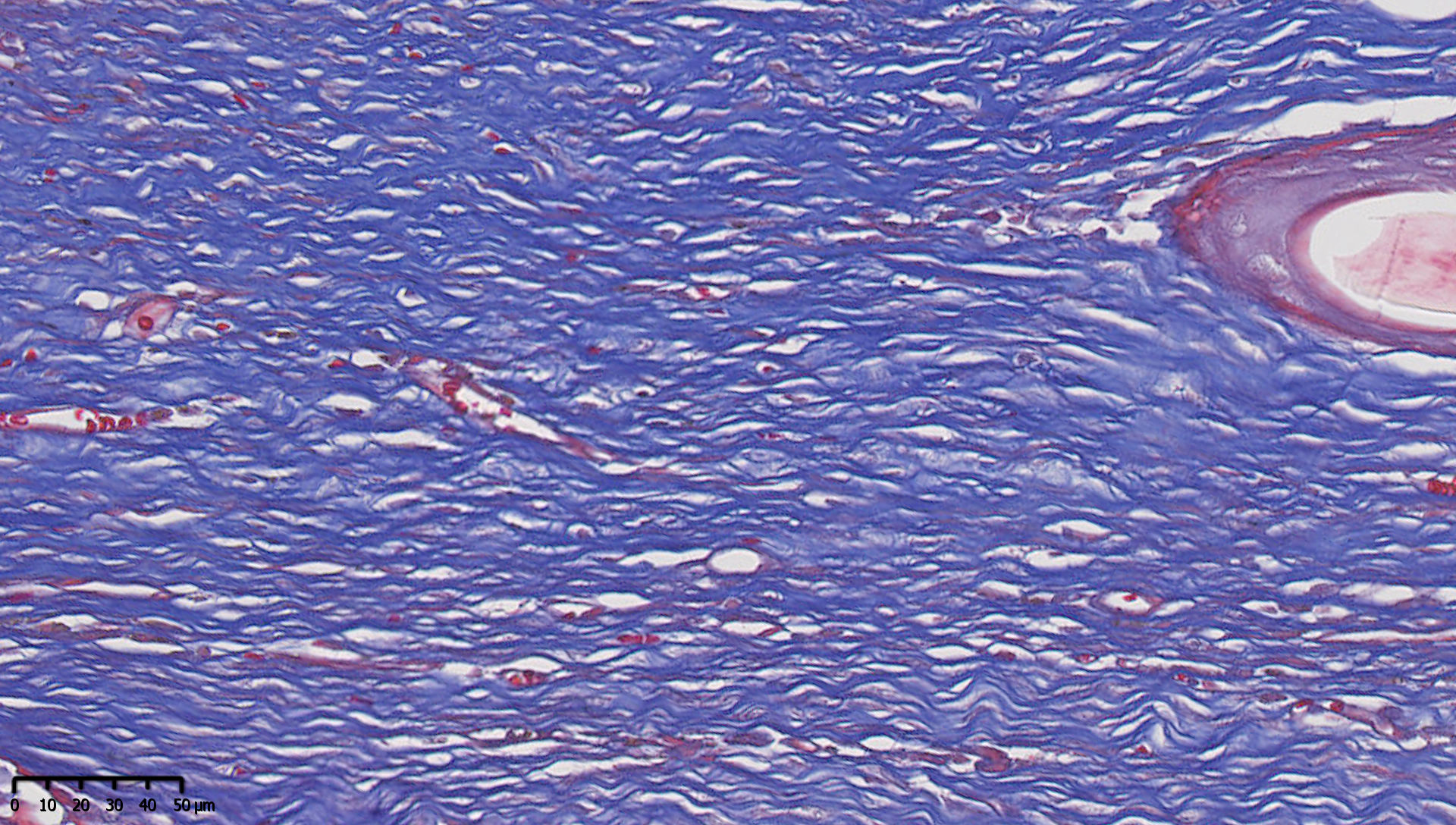

Supplement: S1 File — (ZIP) [file pone.0324264.s001.zip › supplement.material-1/Masson triple section image/control-13 400x.jpg]

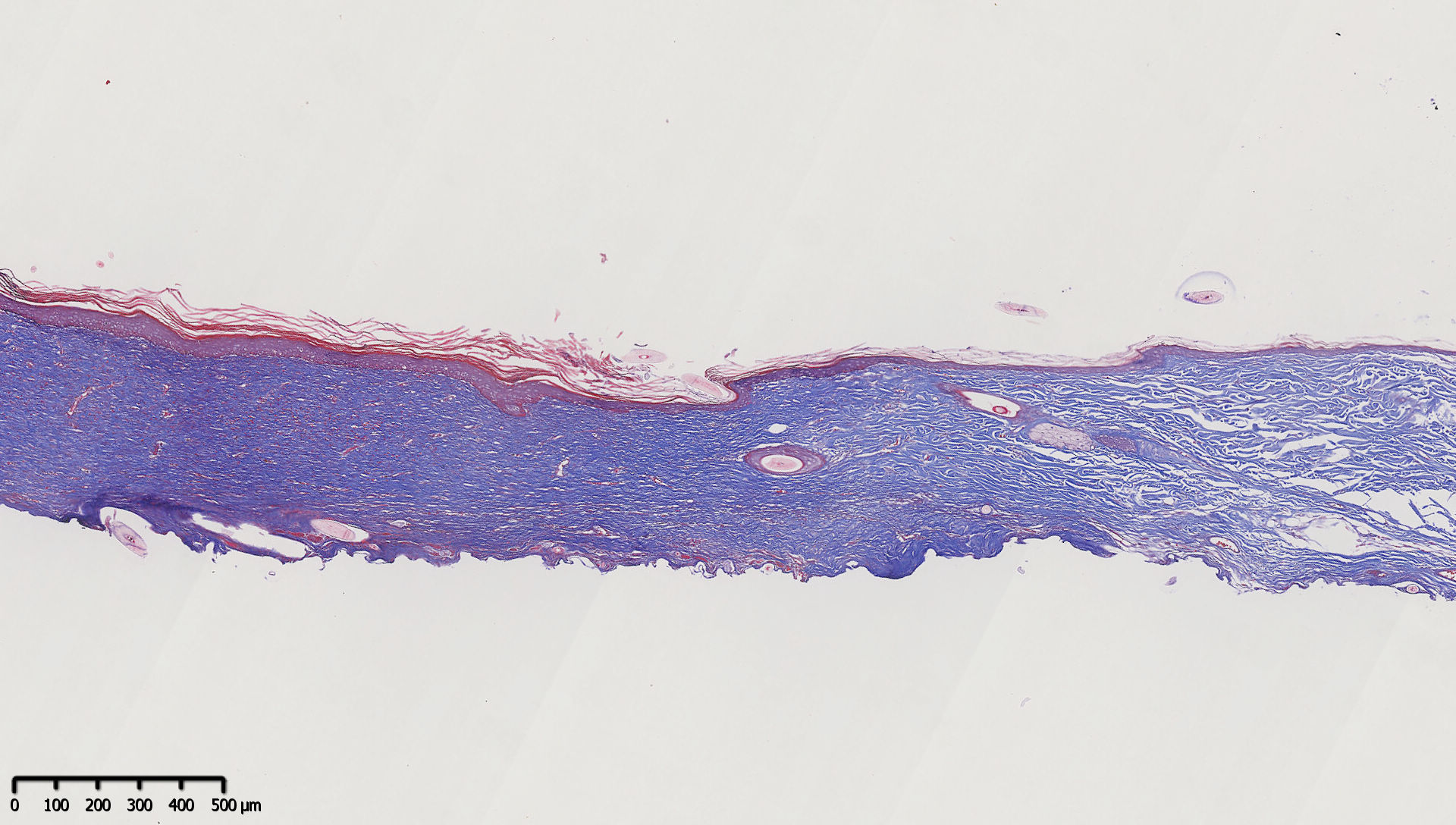

Supplement: S1 File — (ZIP) [file pone.0324264.s001.zip › supplement.material-1/Masson triple section image/control-13 50x.jpg]

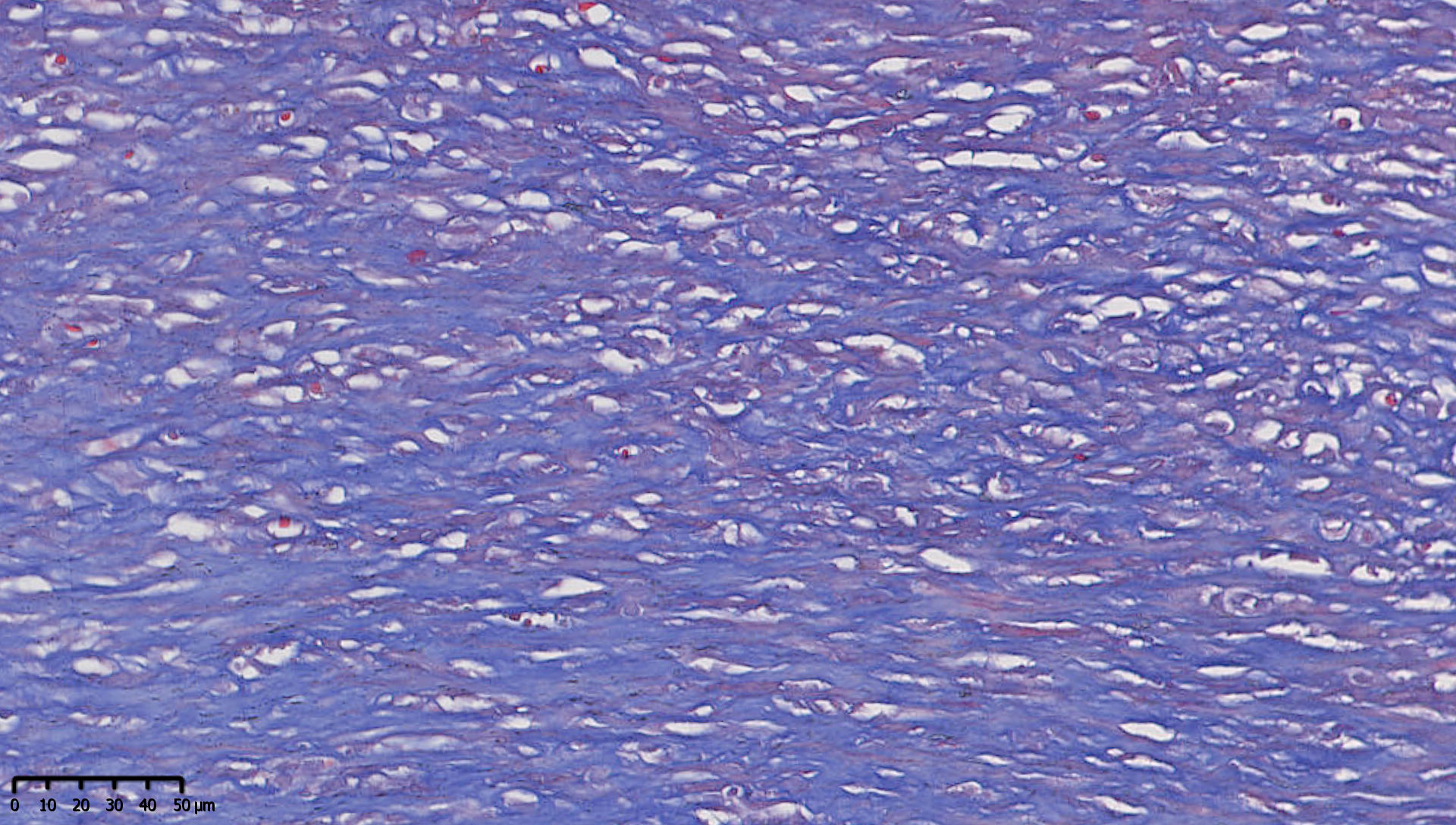

Supplement: S1 File — (ZIP) [file pone.0324264.s001.zip › supplement.material-1/Masson triple section image/HA-11 400x.jpg]

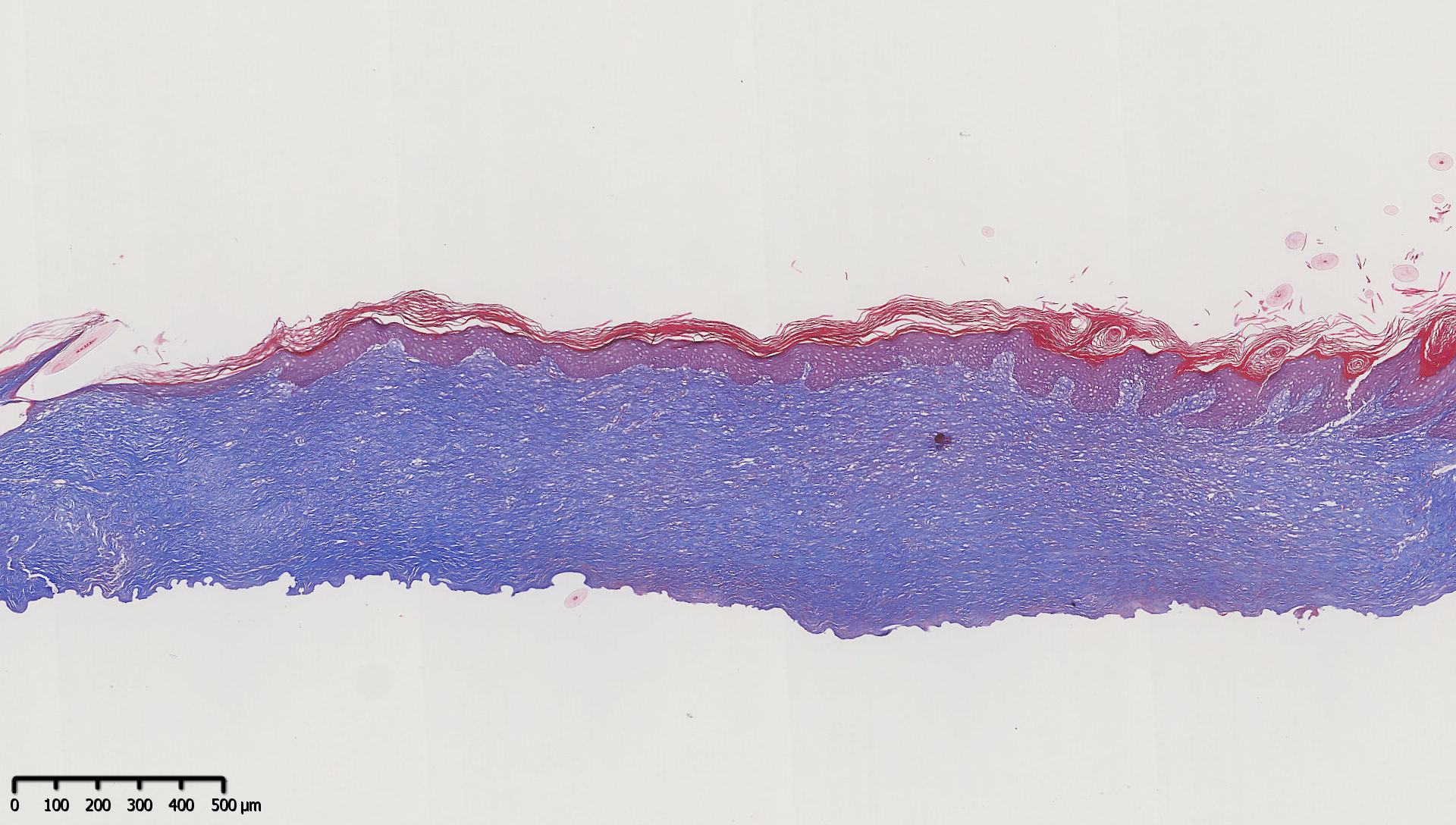

Supplement: S1 File — (ZIP) [file pone.0324264.s001.zip › supplement.material-1/Masson triple section image/HA-11 50x.jpg]

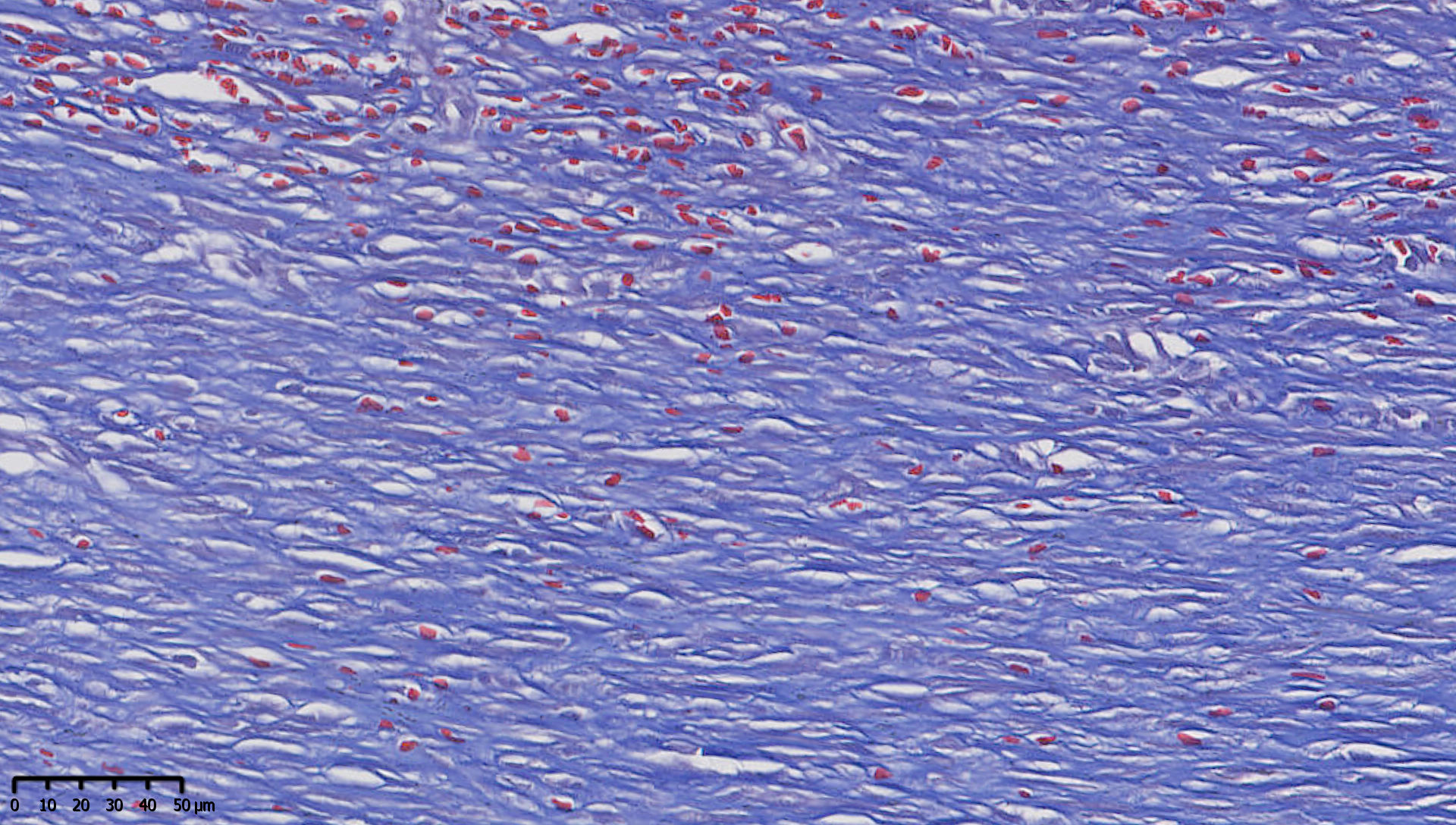

Supplement: S1 File — (ZIP) [file pone.0324264.s001.zip › supplement.material-1/Masson triple section image/HA-12 400x.jpg]

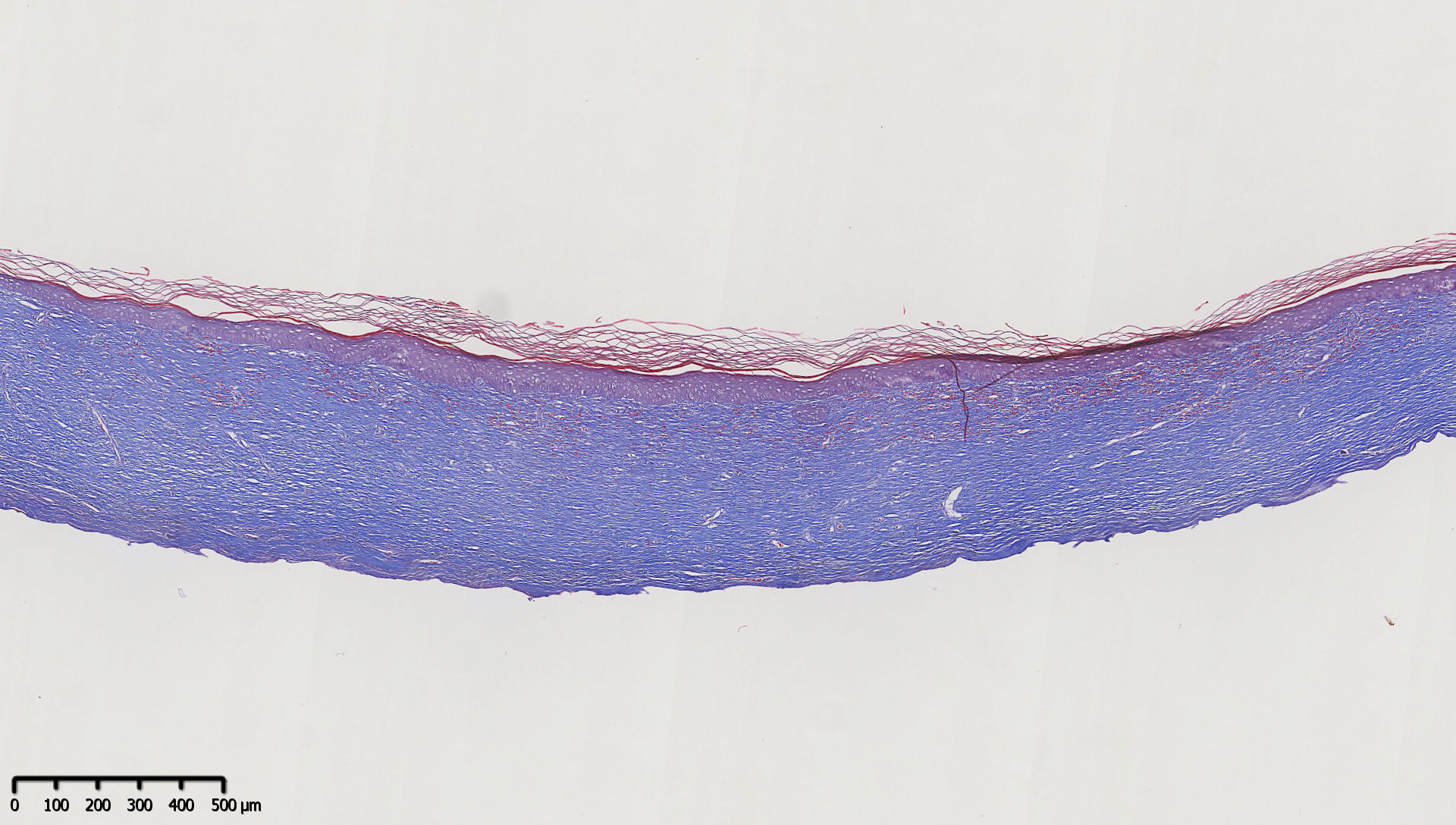

Supplement: S1 File — (ZIP) [file pone.0324264.s001.zip › supplement.material-1/Masson triple section image/HA-12 50x.jpg]

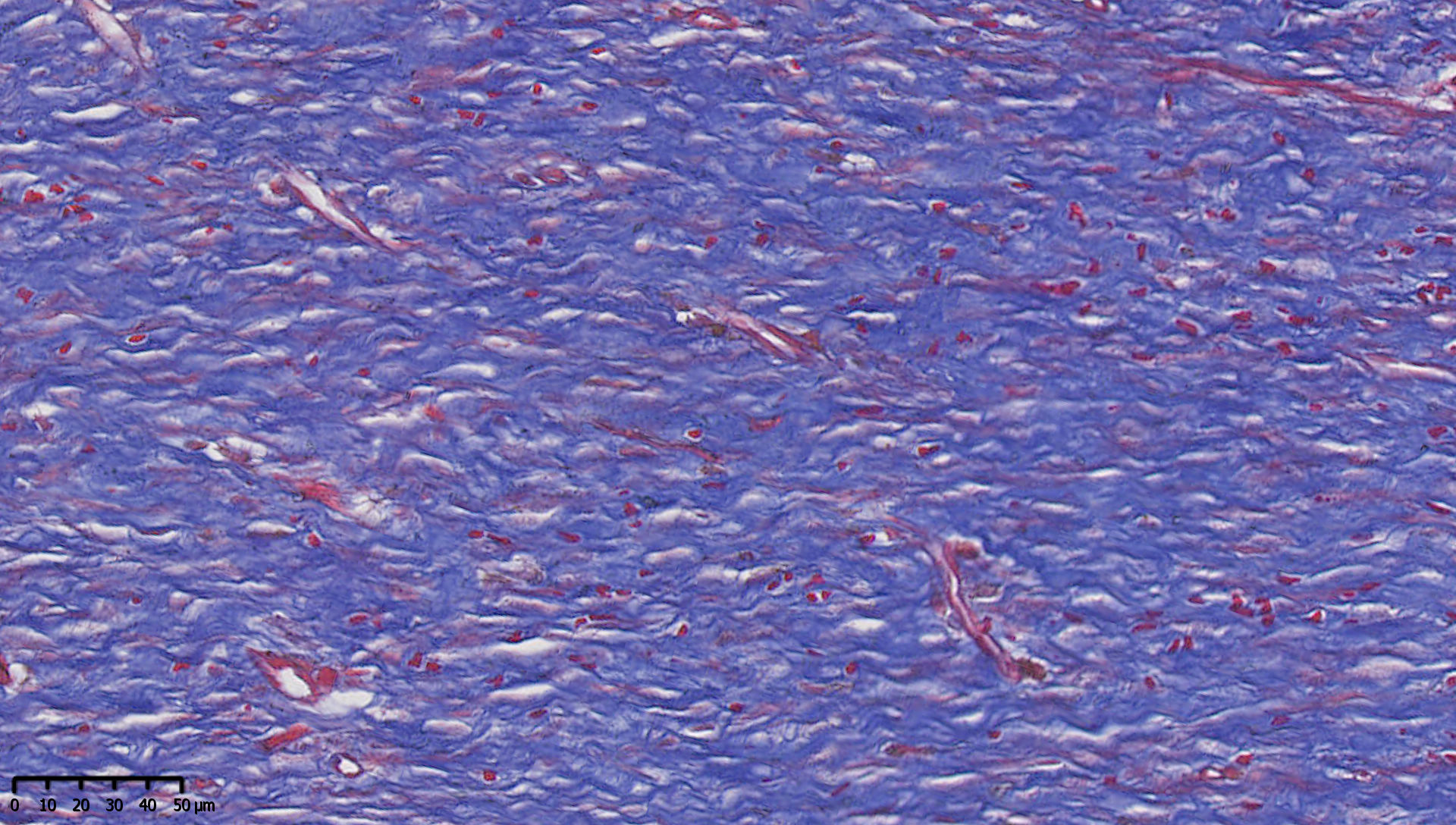

Supplement: S1 File — (ZIP) [file pone.0324264.s001.zip › supplement.material-1/Masson triple section image/HA-13 400x.jpg]

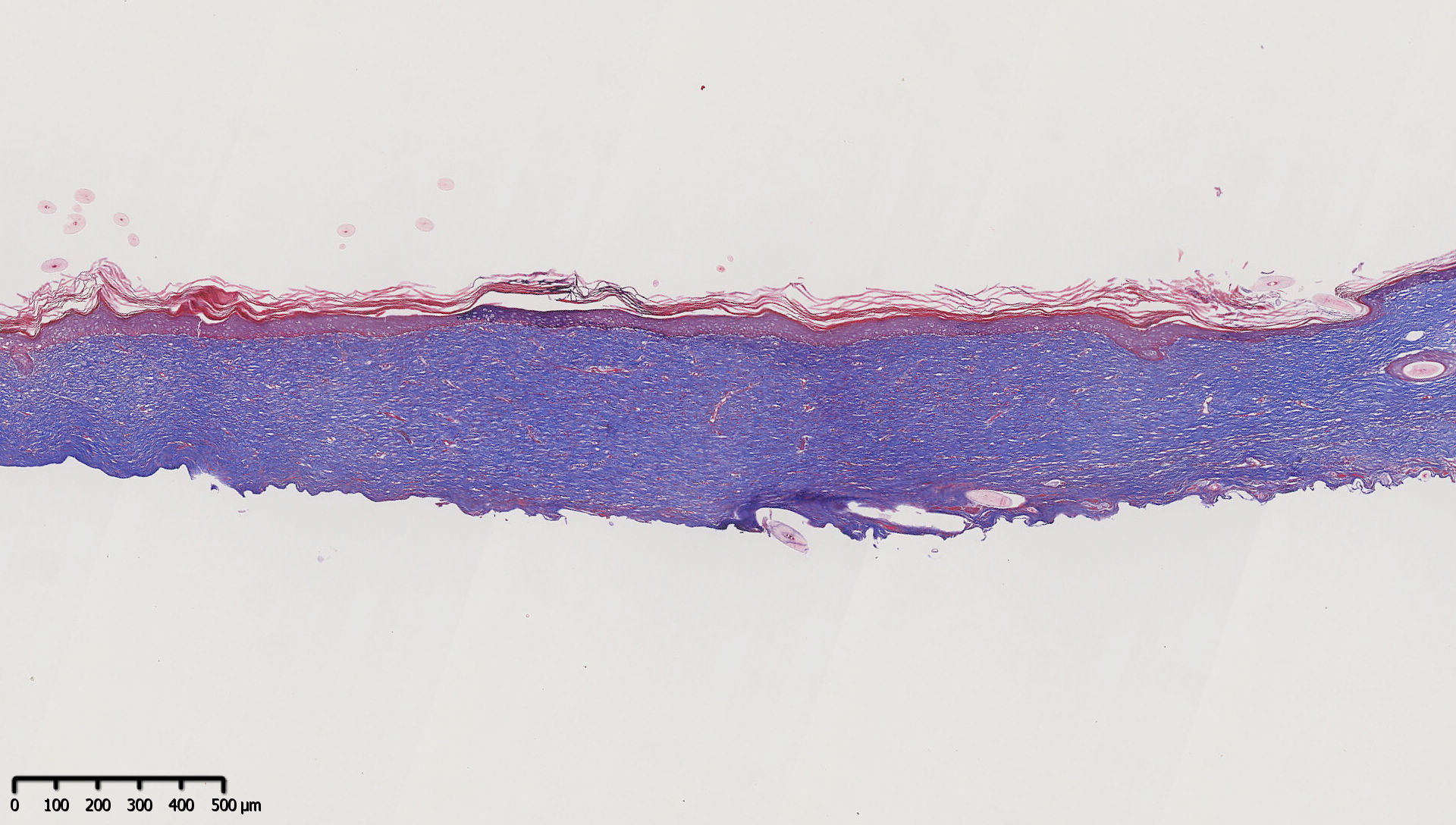

Supplement: S1 File — (ZIP) [file pone.0324264.s001.zip › supplement.material-1/Masson triple section image/HA-13 50x.jpg]

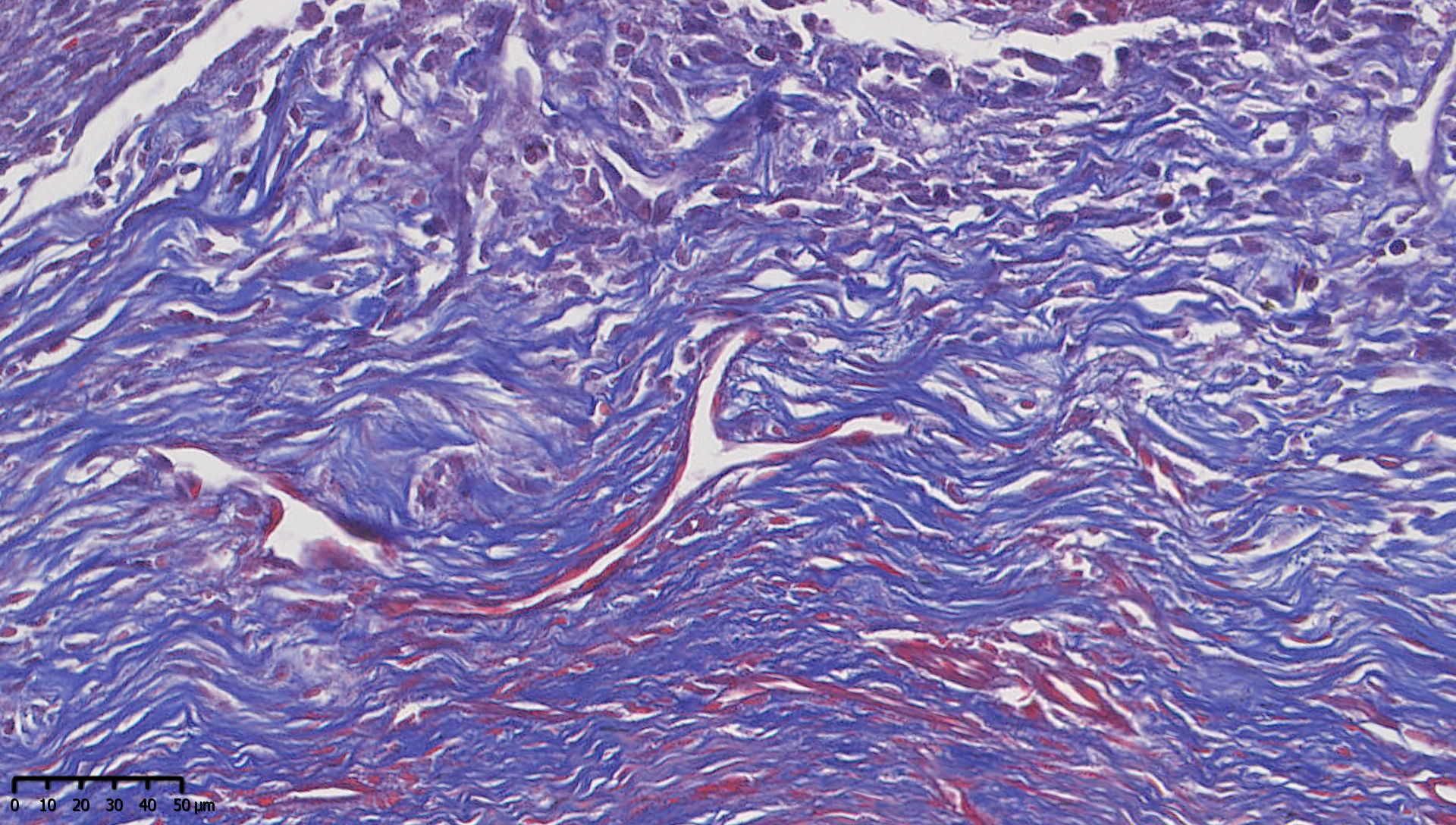

Supplement: S1 File — (ZIP) [file pone.0324264.s001.zip › supplement.material-1/Masson triple section image/model-11 400x.jpg]

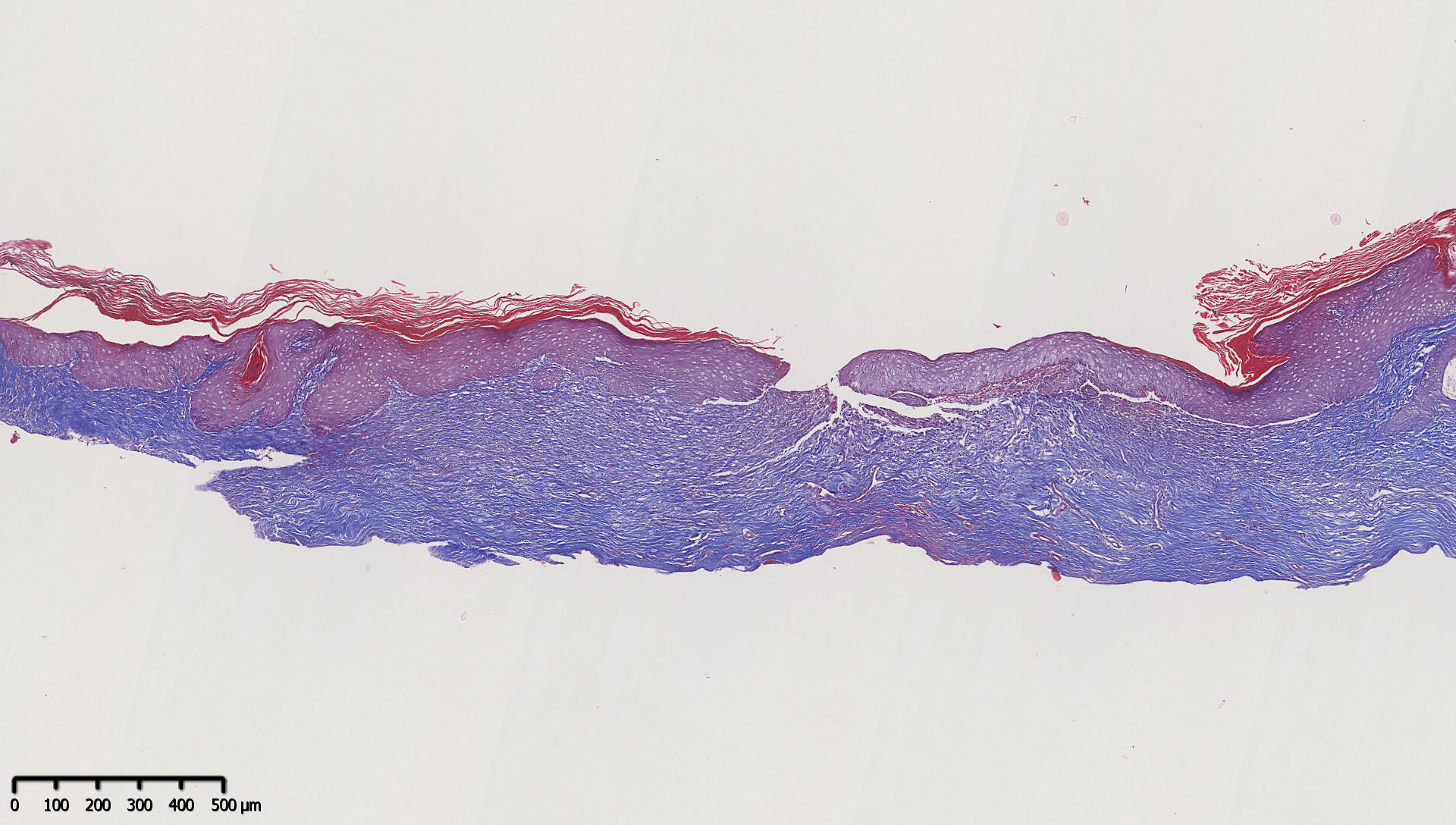

Supplement: S1 File — (ZIP) [file pone.0324264.s001.zip › supplement.material-1/Masson triple section image/model-11 50x.jpg]

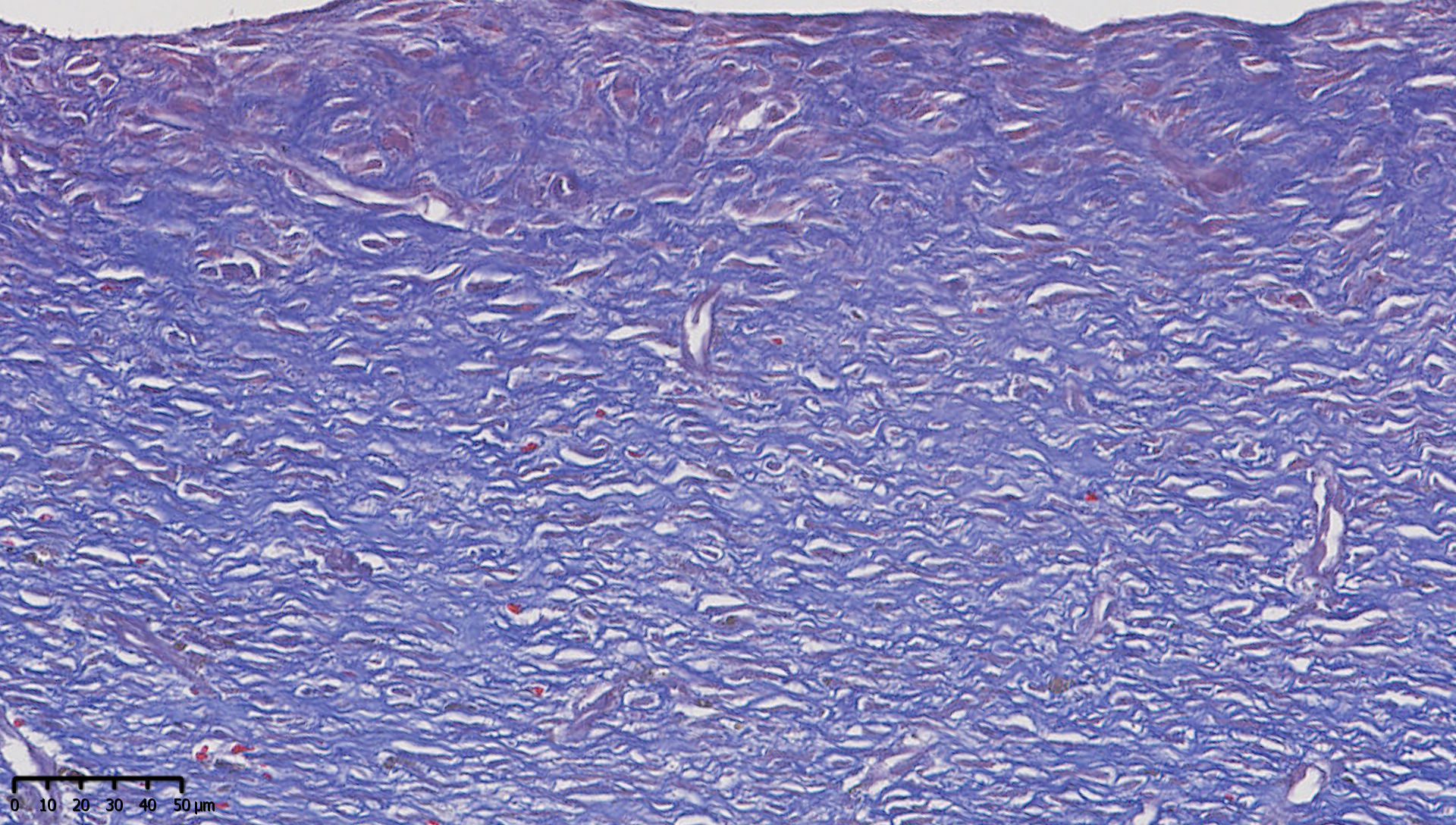

Supplement: S1 File — (ZIP) [file pone.0324264.s001.zip › supplement.material-1/Masson triple section image/model-12 400x.jpg]

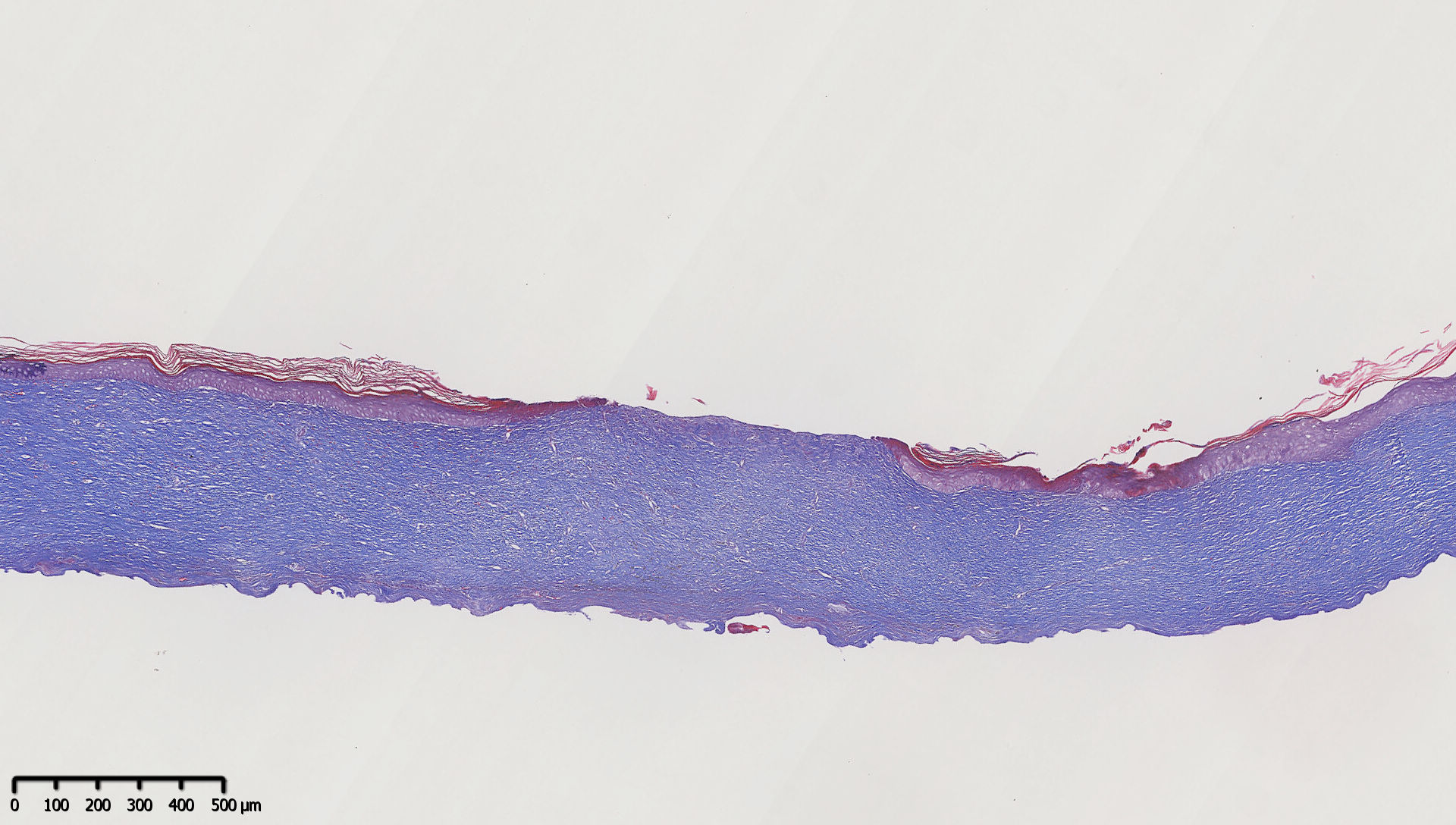

Supplement: S1 File — (ZIP) [file pone.0324264.s001.zip › supplement.material-1/Masson triple section image/model-12 50x.jpg]

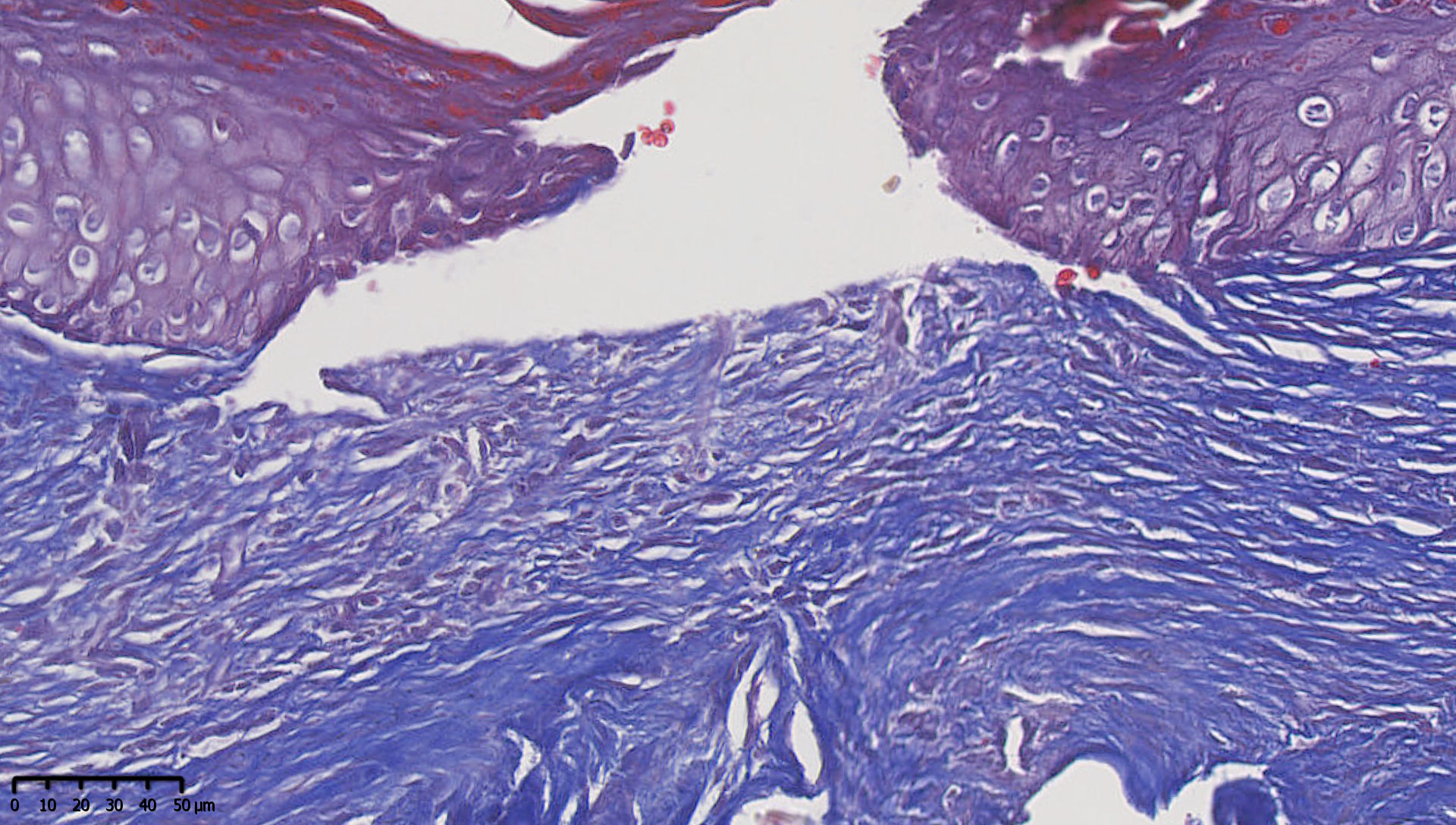

Supplement: S1 File — (ZIP) [file pone.0324264.s001.zip › supplement.material-1/Masson triple section image/model-13 400x.jpg]

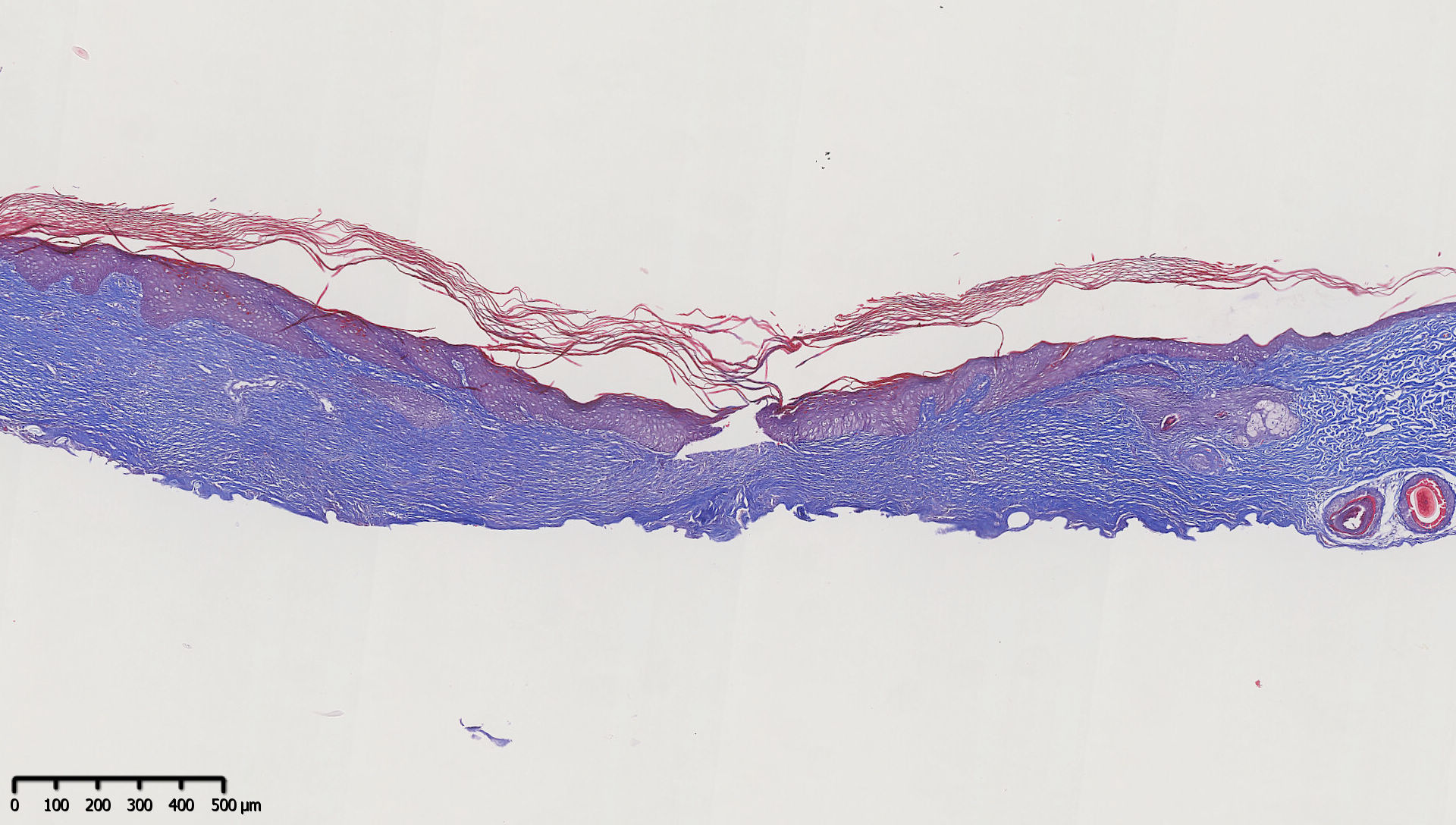

Supplement: S1 File — (ZIP) [file pone.0324264.s001.zip › supplement.material-1/Masson triple section image/model-13 50x.jpg]

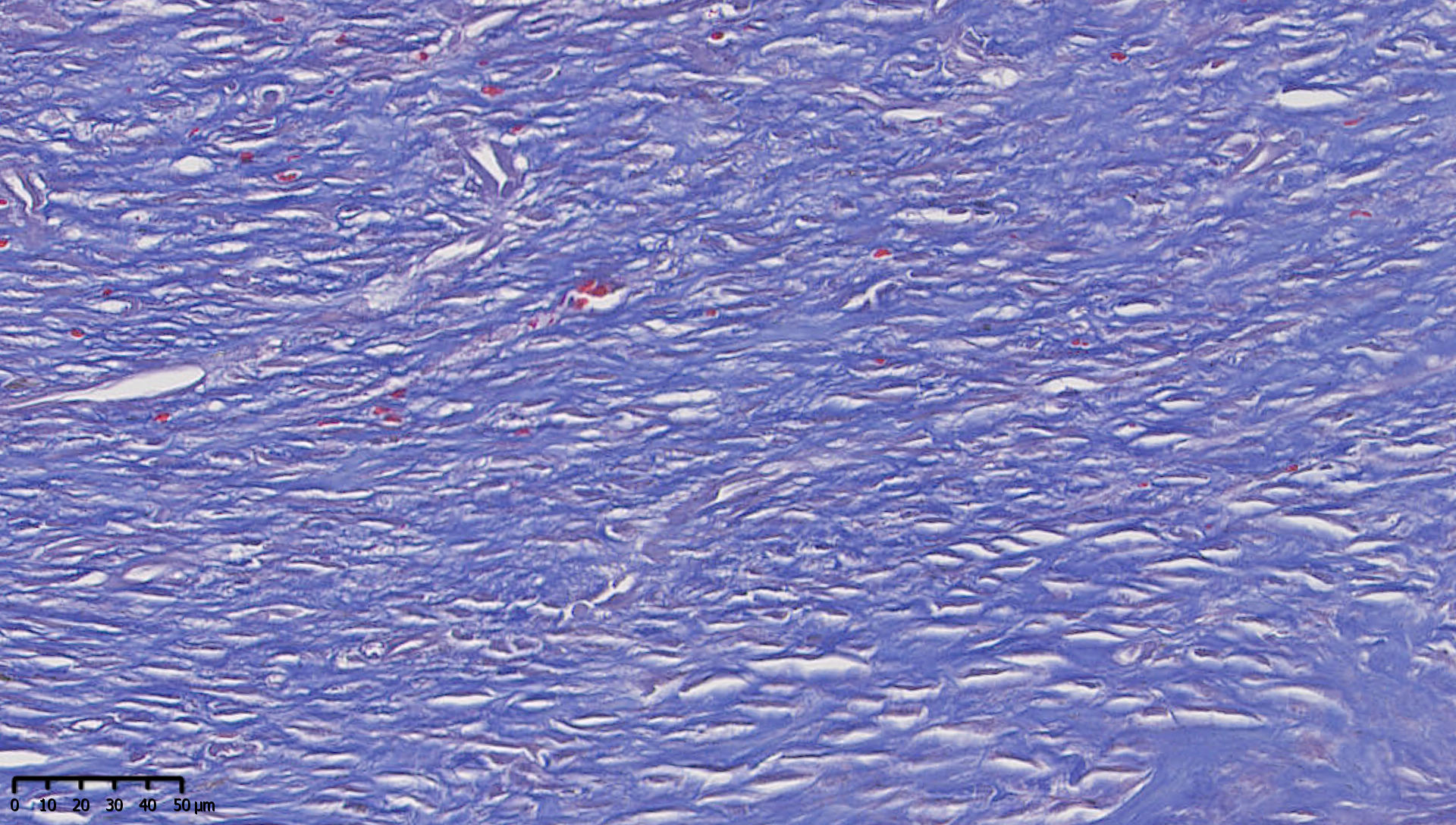

Supplement: S1 File — (ZIP) [file pone.0324264.s001.zip › supplement.material-1/Masson triple section image/PL-HA-11 400x.jpg]

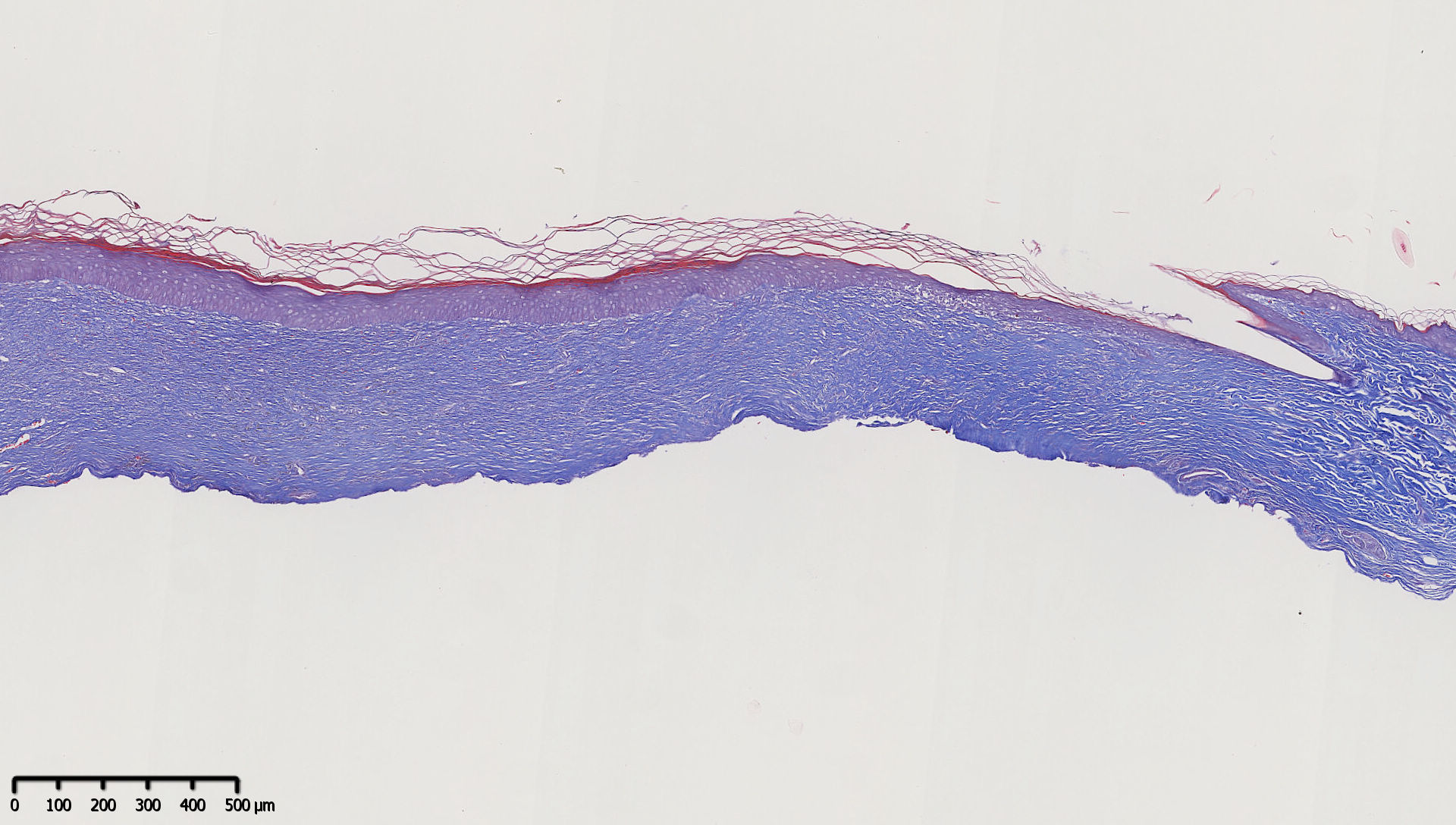

Supplement: S1 File — (ZIP) [file pone.0324264.s001.zip › supplement.material-1/Masson triple section image/PL-HA-11 50x.jpg]

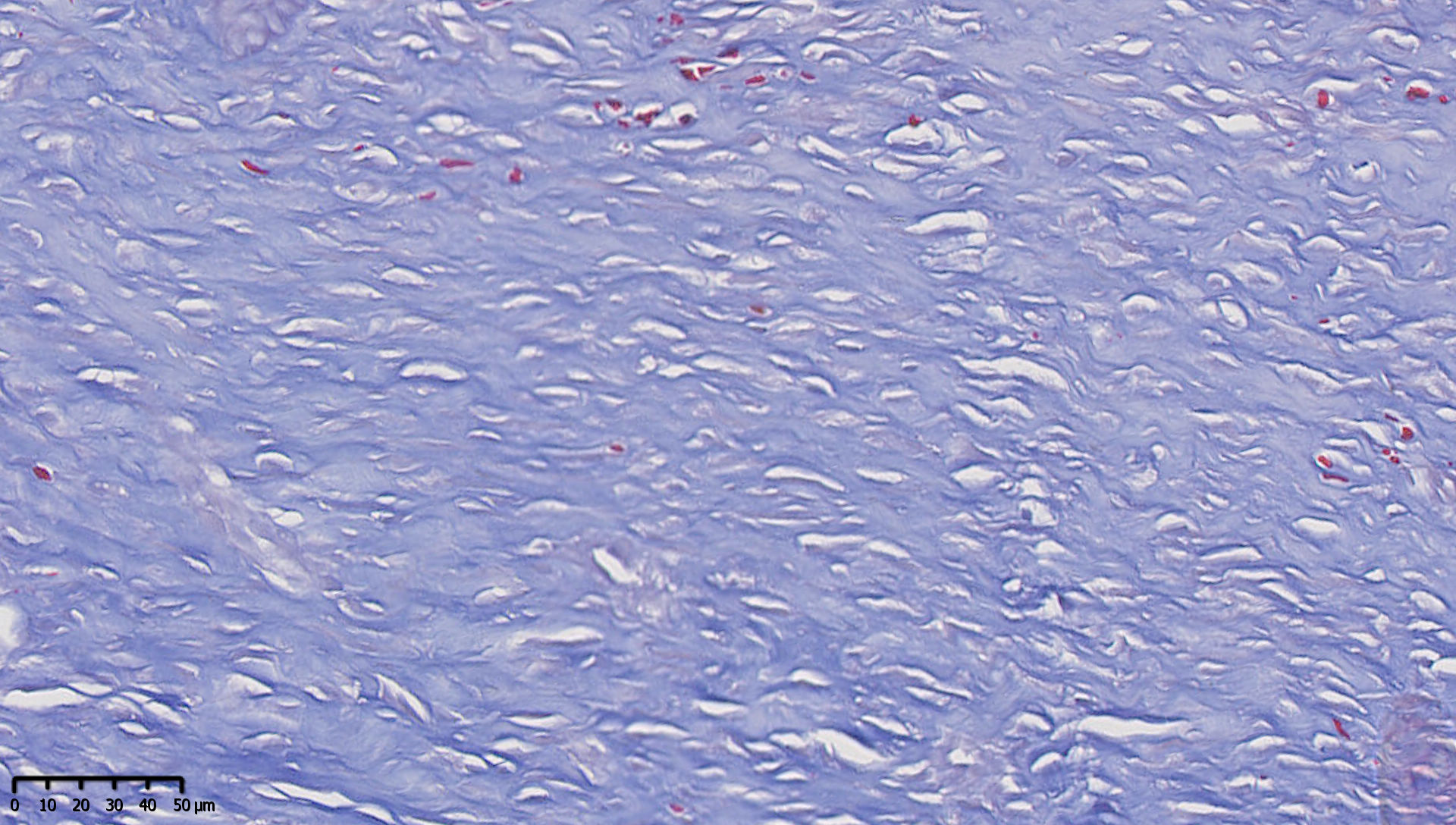

Supplement: S1 File — (ZIP) [file pone.0324264.s001.zip › supplement.material-1/Masson triple section image/PL-HA-12 400x.jpg]

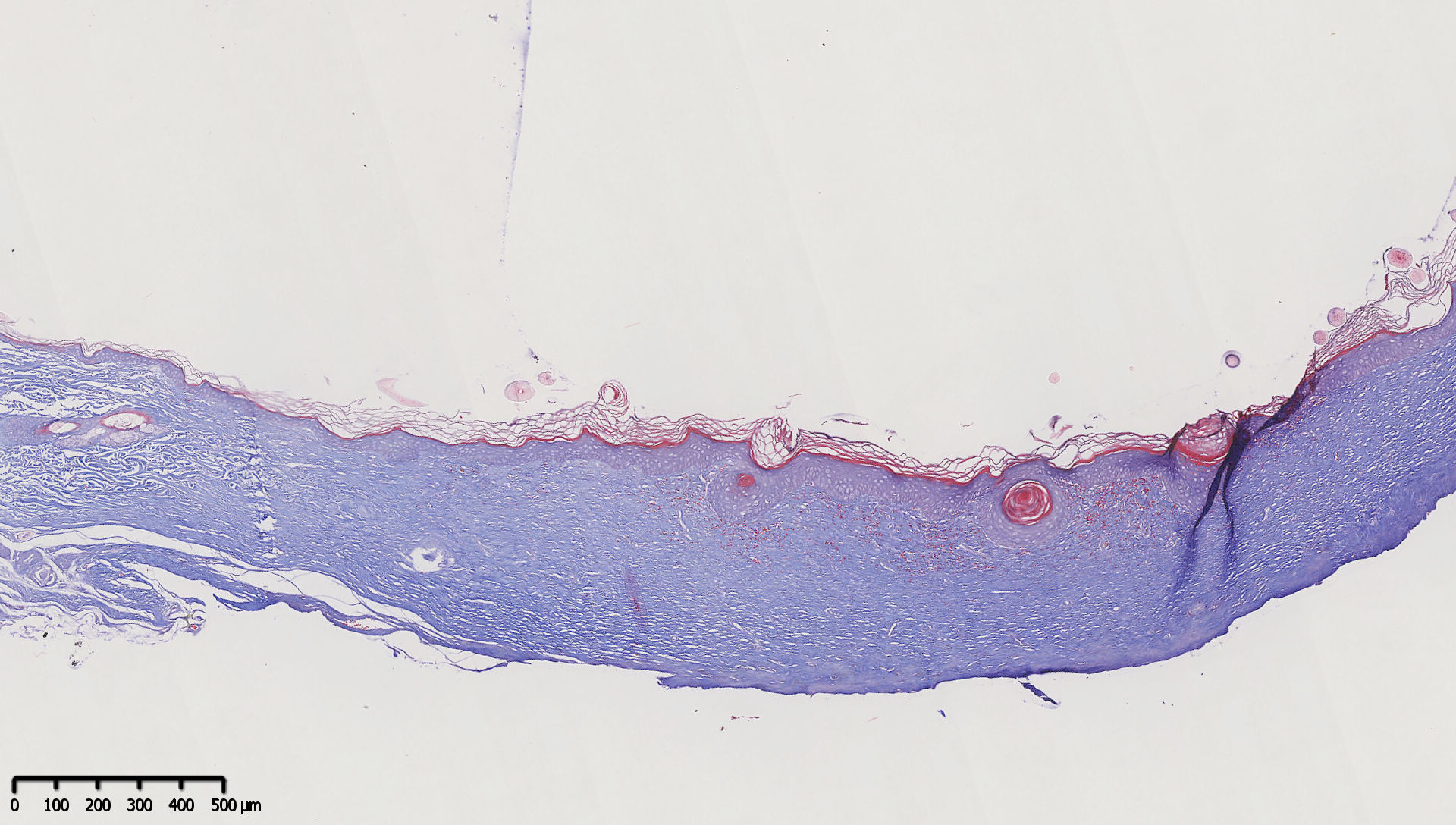

Supplement: S1 File — (ZIP) [file pone.0324264.s001.zip › supplement.material-1/Masson triple section image/PL-HA-12 50x.jpg]

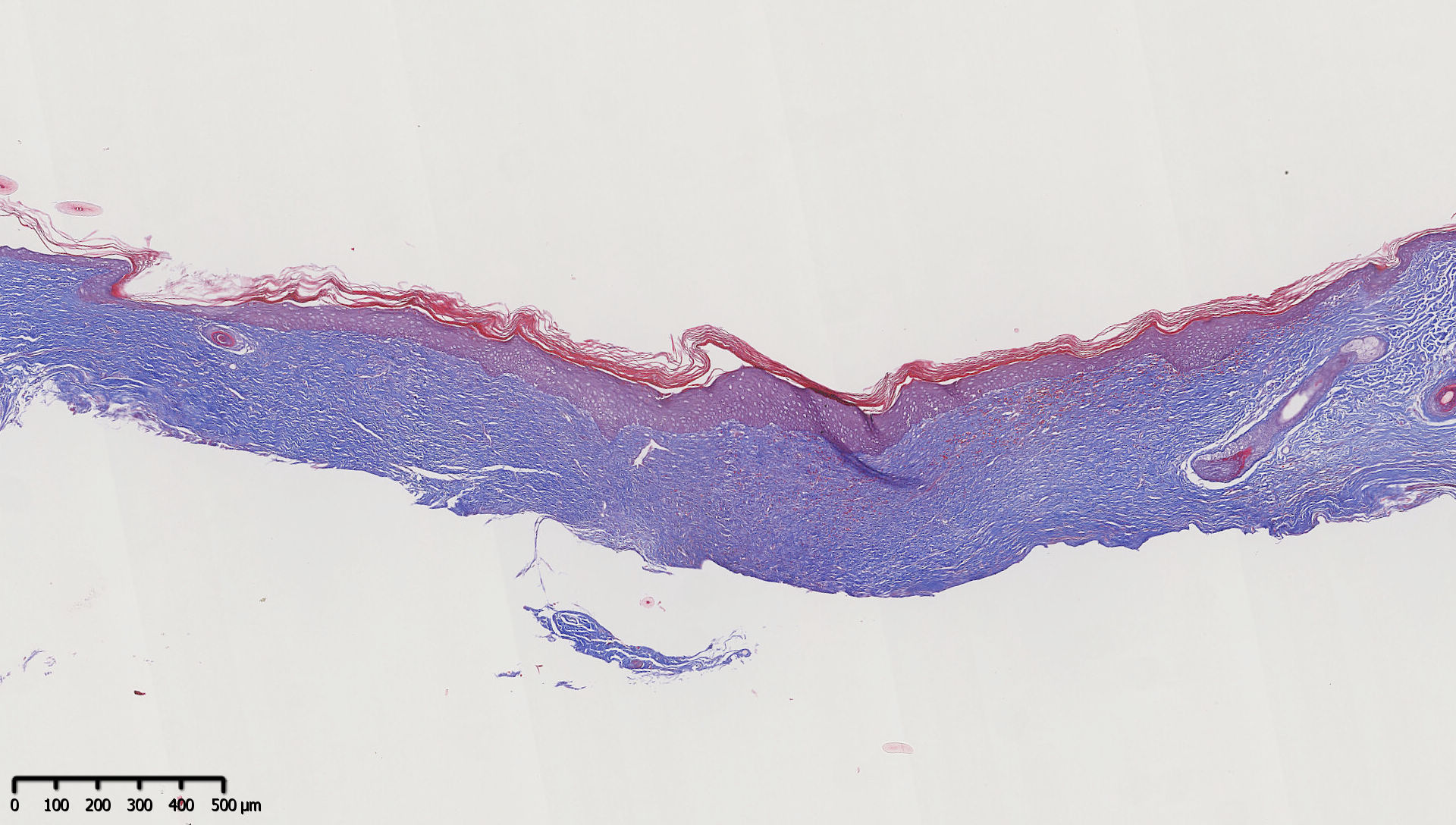

Supplement: S1 File — (ZIP) [file pone.0324264.s001.zip › supplement.material-1/Masson triple section image/PL-HA-13 50x.jpg]

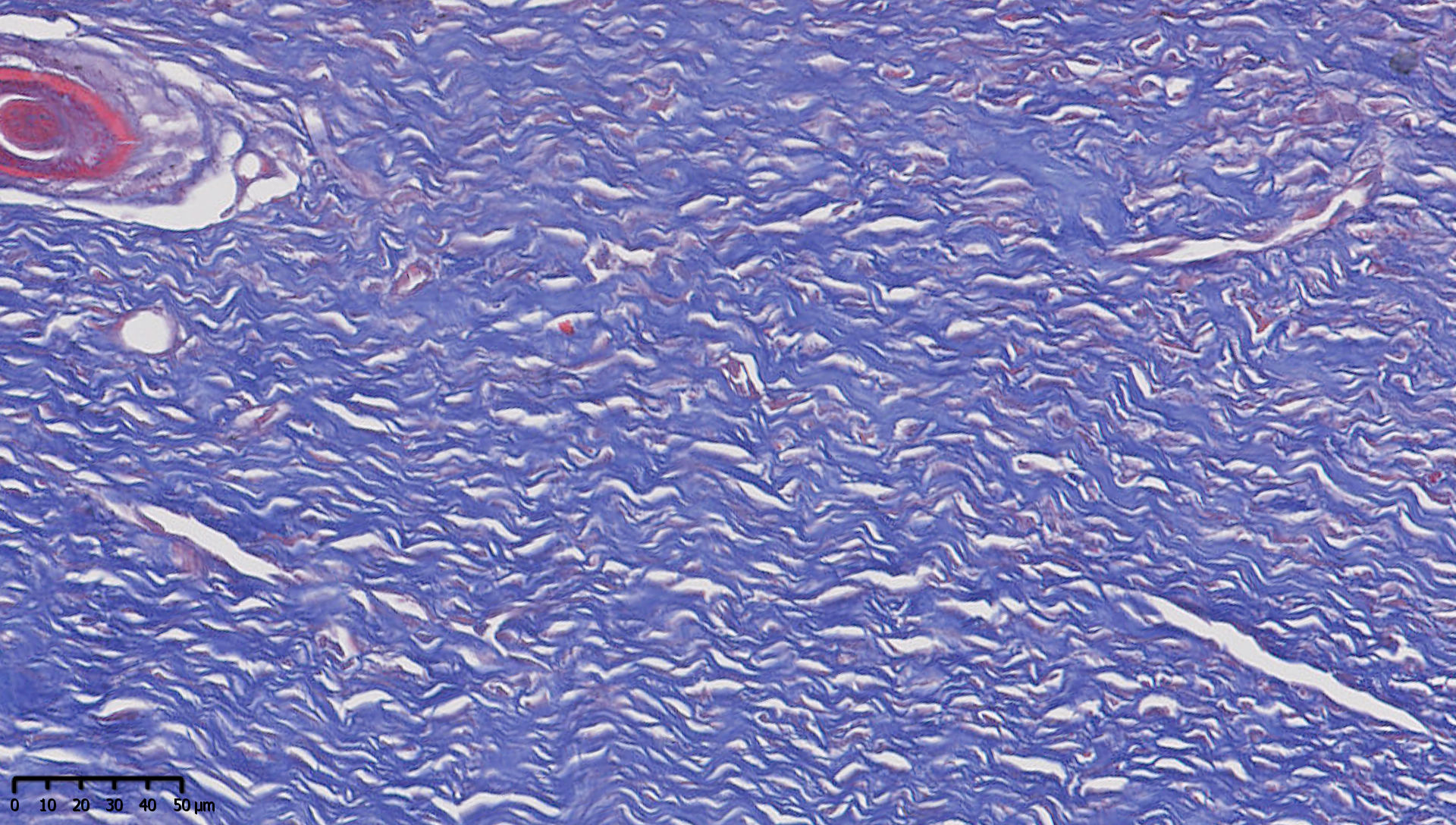

Supplement: S1 File — (ZIP) [file pone.0324264.s001.zip › supplement.material-1/Masson triple section image/PL-HA-3 400x.jpg]

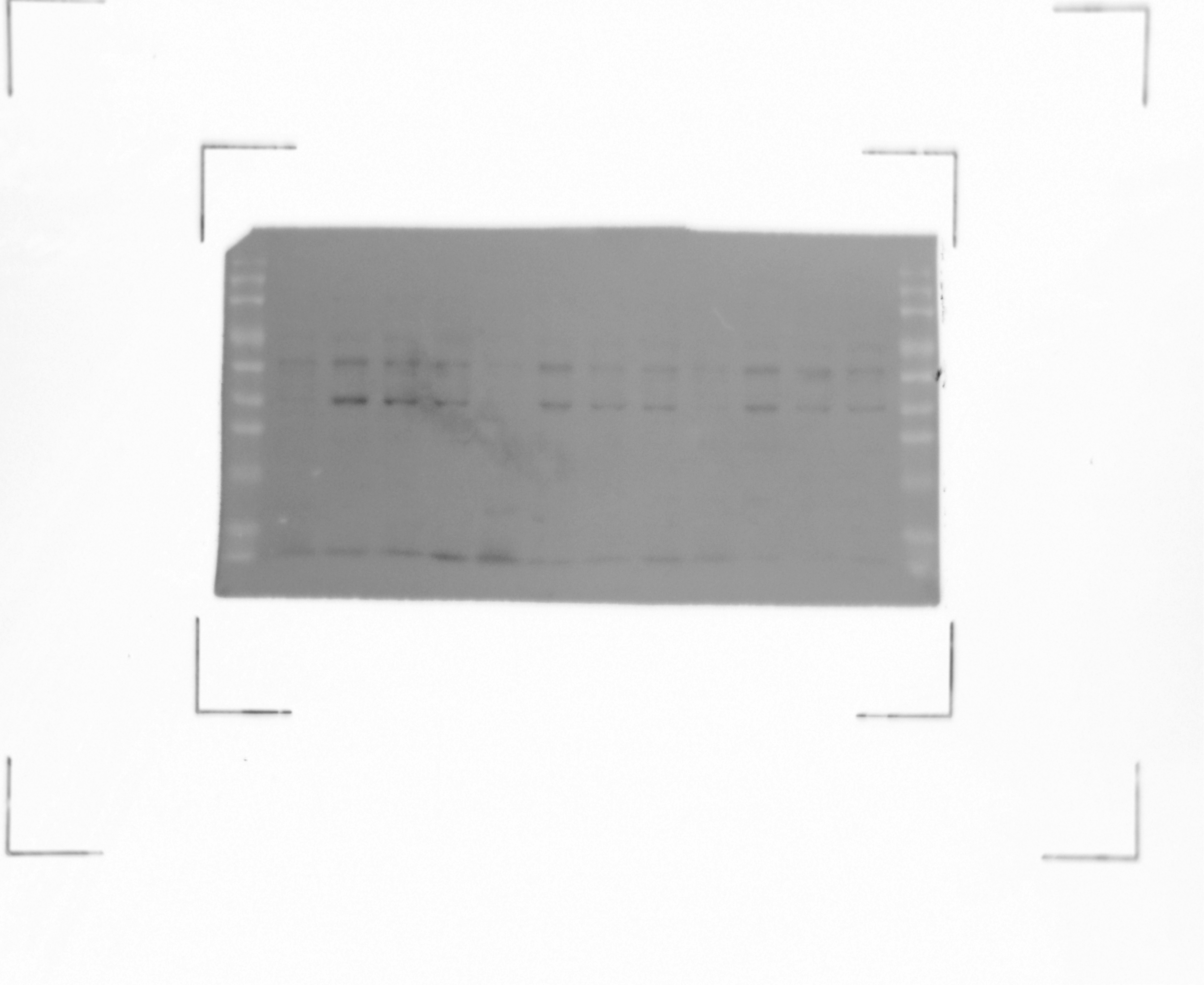

Supplement: S1 File — (ZIP) [file pone.0324264.s001.zip › supplement.material-1/western blot/animal tissues/WB Original strips/Beclin1+M.tif]

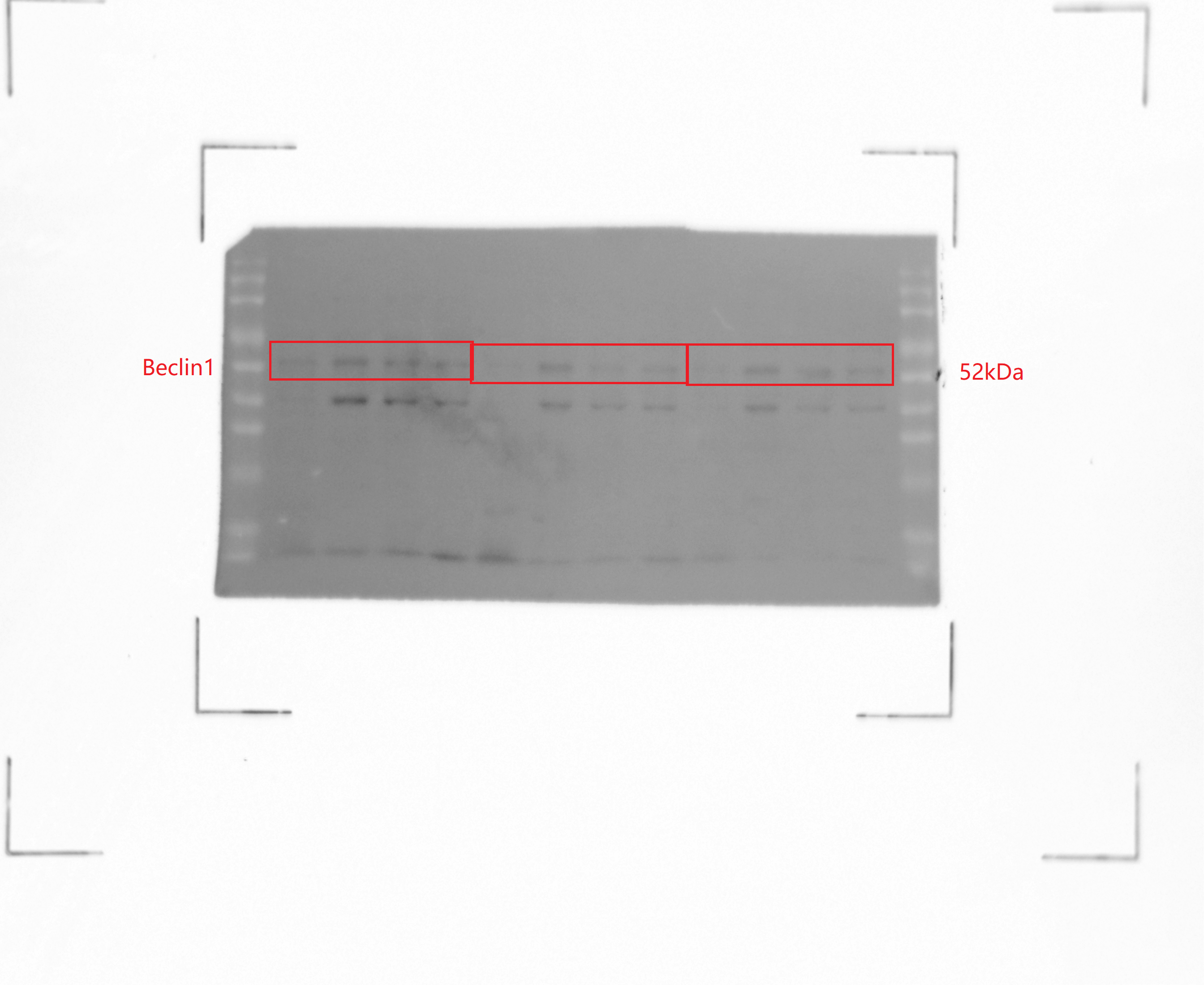

Supplement: S1 File — (ZIP) [file pone.0324264.s001.zip › supplement.material-1/western blot/animal tissues/WB Original strips/Beclin1.tif]

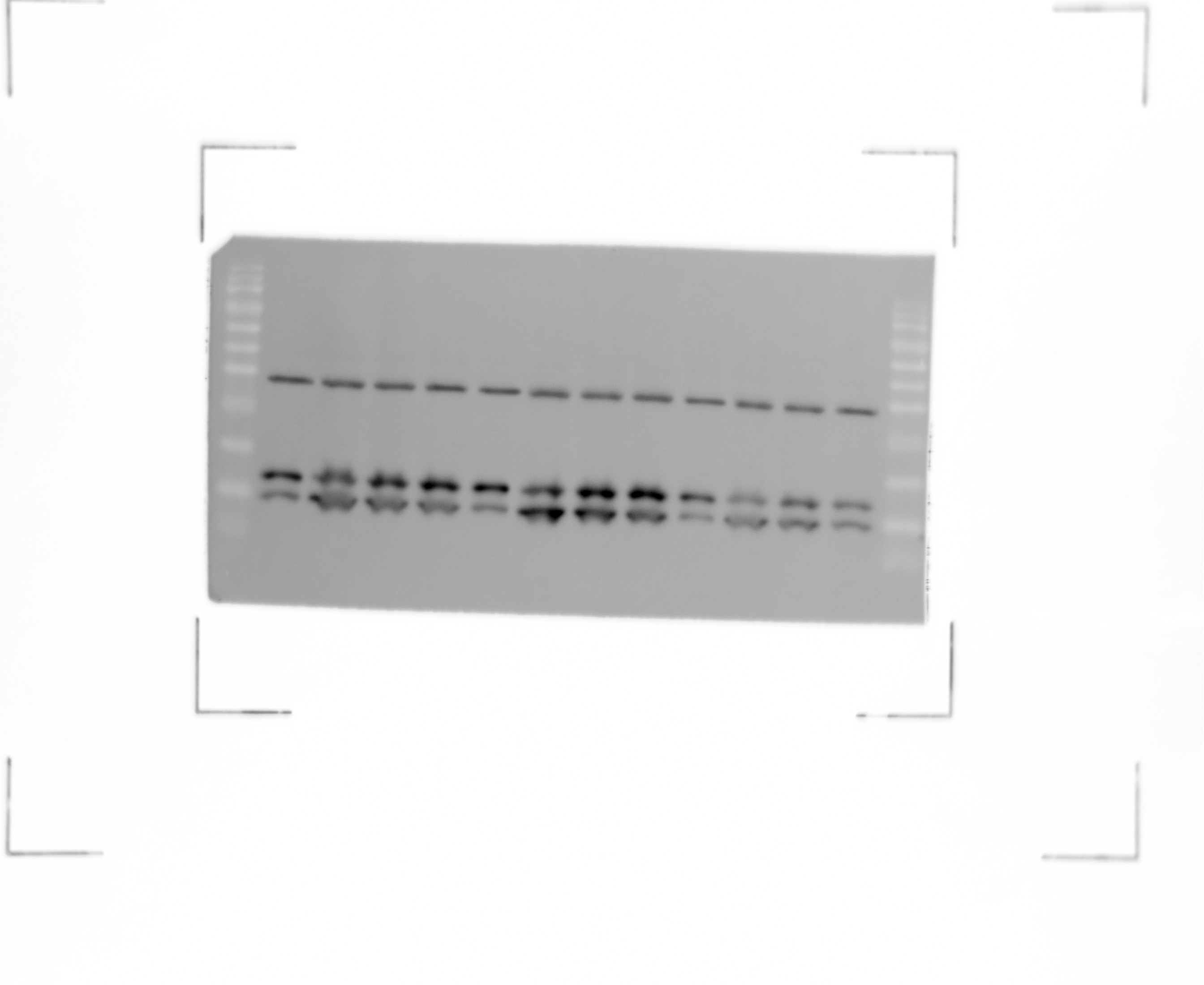

Supplement: S1 File — (ZIP) [file pone.0324264.s001.zip › supplement.material-1/western blot/animal tissues/WB Original strips/LC3+GAPDH+M.tif]

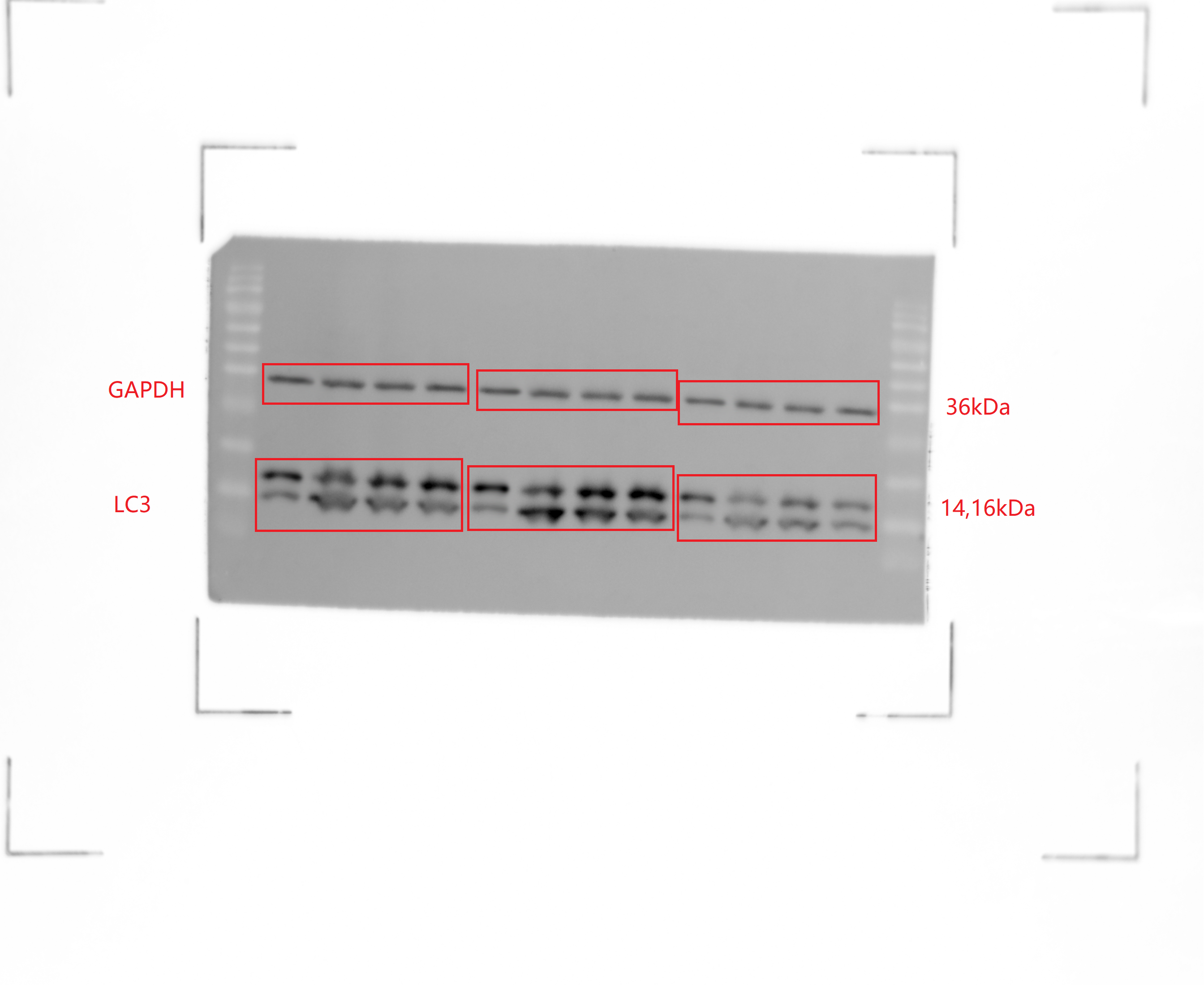

Supplement: S1 File — (ZIP) [file pone.0324264.s001.zip › supplement.material-1/western blot/animal tissues/WB Original strips/LC3+GAPDH.tif]

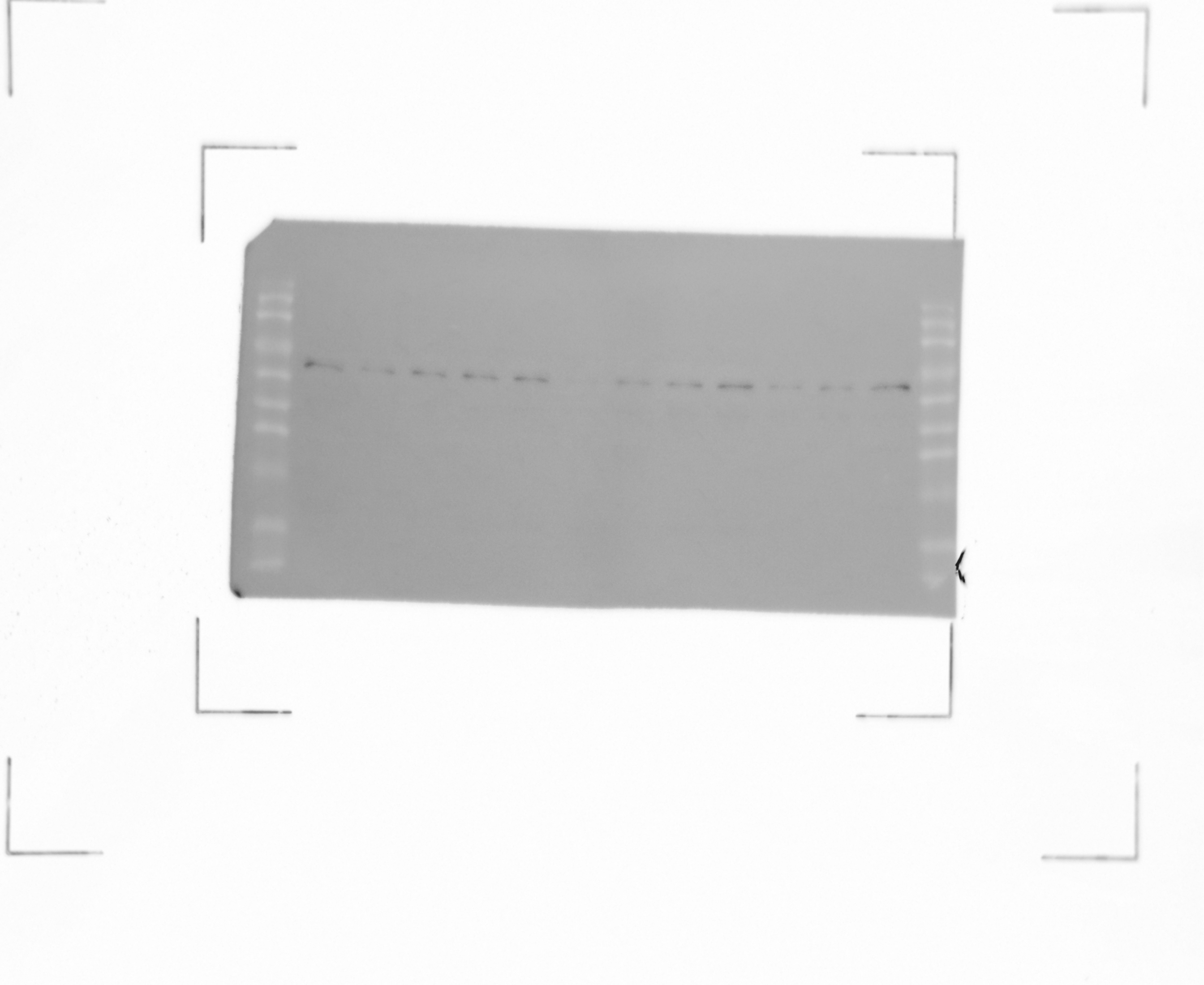

Supplement: S1 File — (ZIP) [file pone.0324264.s001.zip › supplement.material-1/western blot/animal tissues/WB Original strips/p62+M.tif]

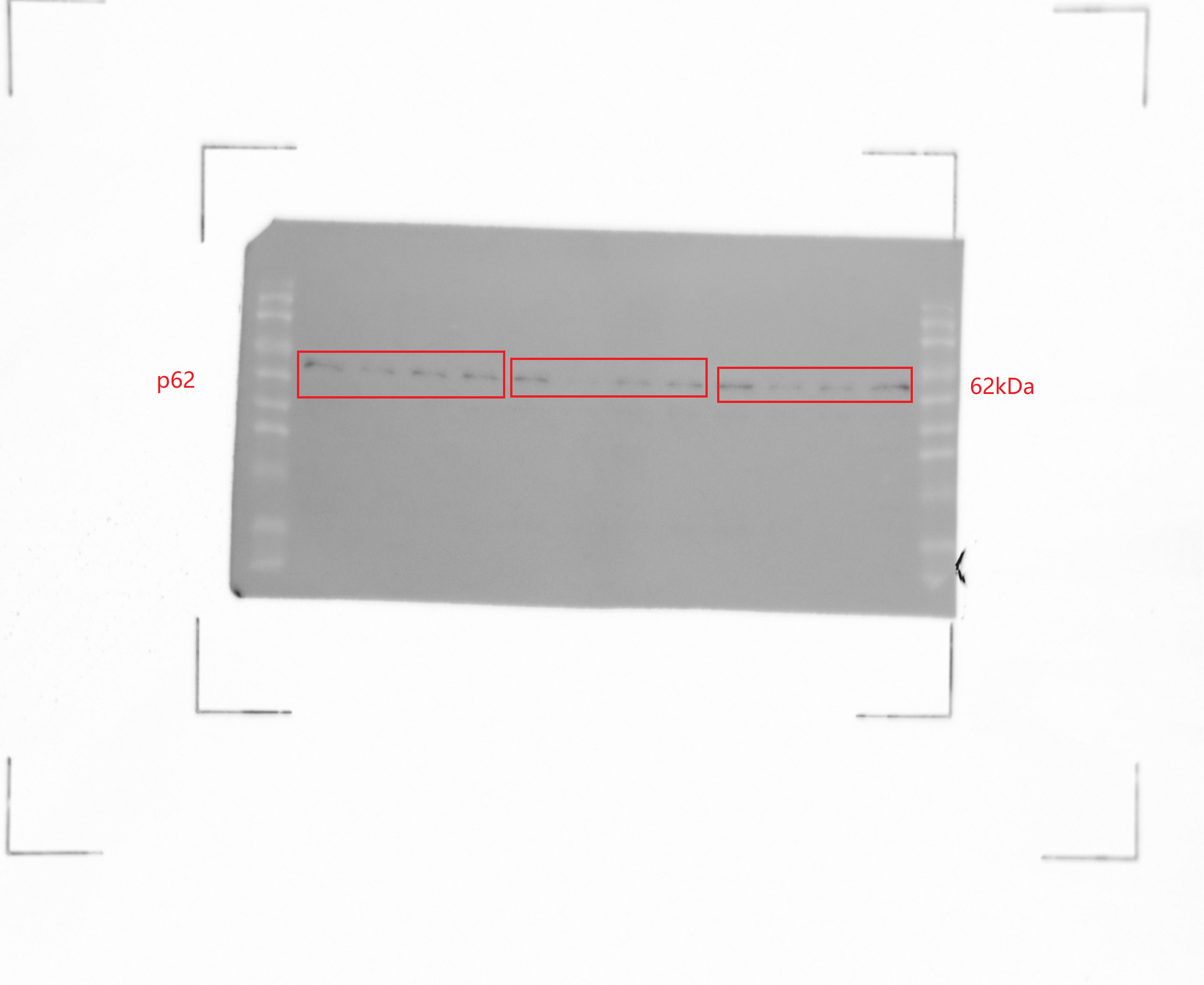

Supplement: S1 File — (ZIP) [file pone.0324264.s001.zip › supplement.material-1/western blot/animal tissues/WB Original strips/p62.tif]

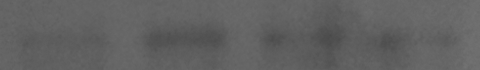

Supplement: S1 File — (ZIP) [file pone.0324264.s001.zip › supplement.material-1/western blot/animal tissues/WB Processing strips/Beclin1-1.tif]

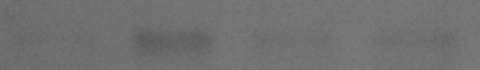

Supplement: S1 File — (ZIP) [file pone.0324264.s001.zip › supplement.material-1/western blot/animal tissues/WB Processing strips/Beclin1-2.tif]

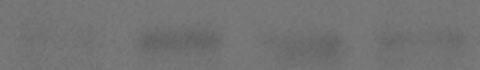

Supplement: S1 File — (ZIP) [file pone.0324264.s001.zip › supplement.material-1/western blot/animal tissues/WB Processing strips/Beclin1-3.tif]

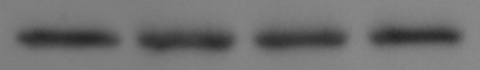

Supplement: S1 File — (ZIP) [file pone.0324264.s001.zip › supplement.material-1/western blot/animal tissues/WB Processing strips/GAPDH-1.tif]

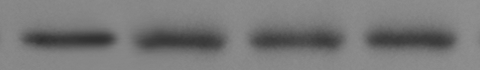

Supplement: S1 File — (ZIP) [file pone.0324264.s001.zip › supplement.material-1/western blot/animal tissues/WB Processing strips/GAPDH-2.tif]

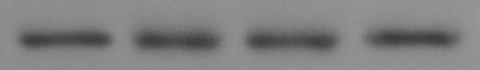

Supplement: S1 File — (ZIP) [file pone.0324264.s001.zip › supplement.material-1/western blot/animal tissues/WB Processing strips/GAPDH-3.tif]

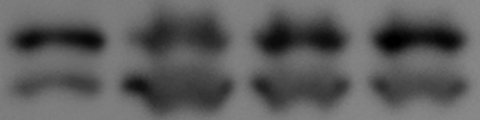

Supplement: S1 File — (ZIP) [file pone.0324264.s001.zip › supplement.material-1/western blot/animal tissues/WB Processing strips/LC3-1.tif]

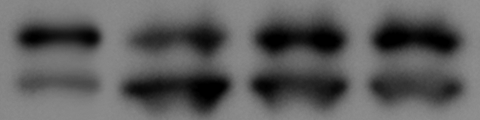

Supplement: S1 File — (ZIP) [file pone.0324264.s001.zip › supplement.material-1/western blot/animal tissues/WB Processing strips/LC3-2.tif]

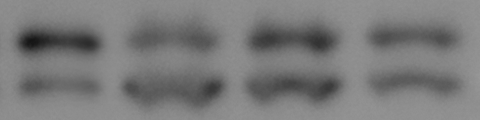

Supplement: S1 File — (ZIP) [file pone.0324264.s001.zip › supplement.material-1/western blot/animal tissues/WB Processing strips/LC3-3.tif]

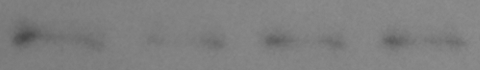

Supplement: S1 File — (ZIP) [file pone.0324264.s001.zip › supplement.material-1/western blot/animal tissues/WB Processing strips/P62-1.tif]

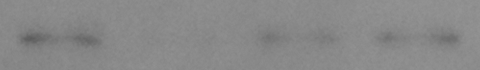

Supplement: S1 File — (ZIP) [file pone.0324264.s001.zip › supplement.material-1/western blot/animal tissues/WB Processing strips/P62-2.tif]

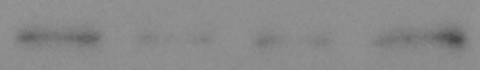

Supplement: S1 File — (ZIP) [file pone.0324264.s001.zip › supplement.material-1/western blot/animal tissues/WB Processing strips/P62-3.tif]

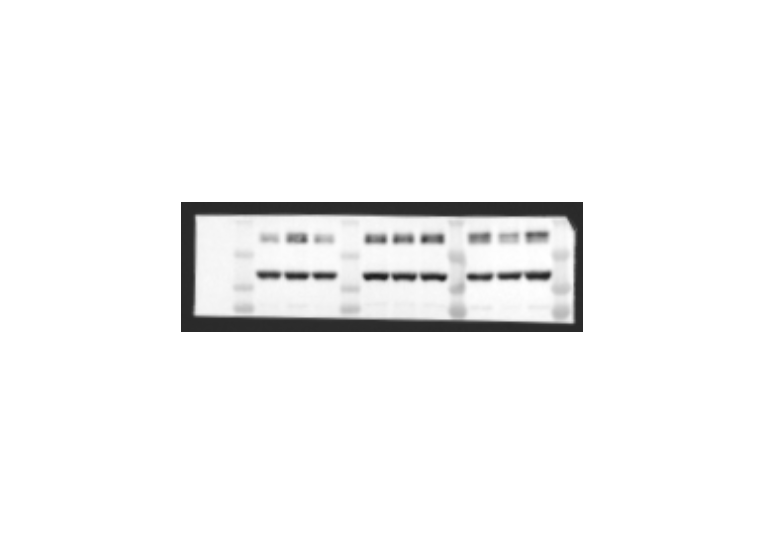

Supplement: S1 File — (ZIP) [file pone.0324264.s001.zip › supplement.material-1/western blot/cell/WB Original strips/1-ACTIN+ACTIN-M.tif]

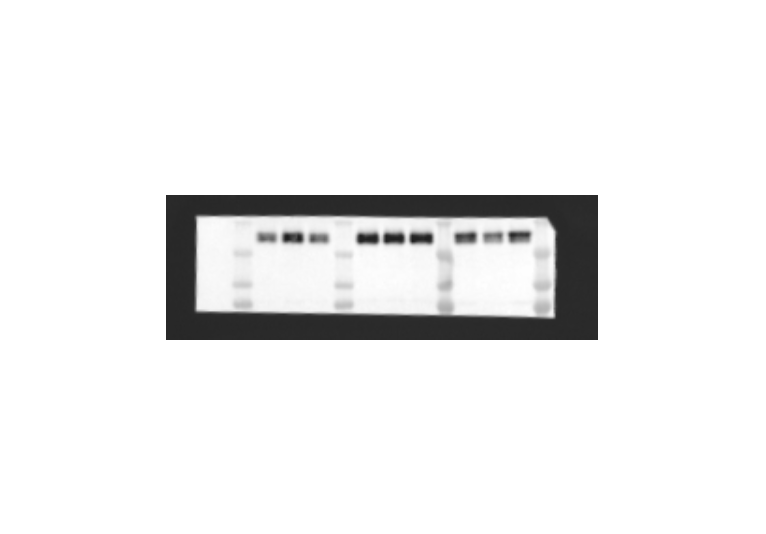

Supplement: S1 File — (ZIP) [file pone.0324264.s001.zip › supplement.material-1/western blot/cell/WB Original strips/1-P62-M+P62.tif]

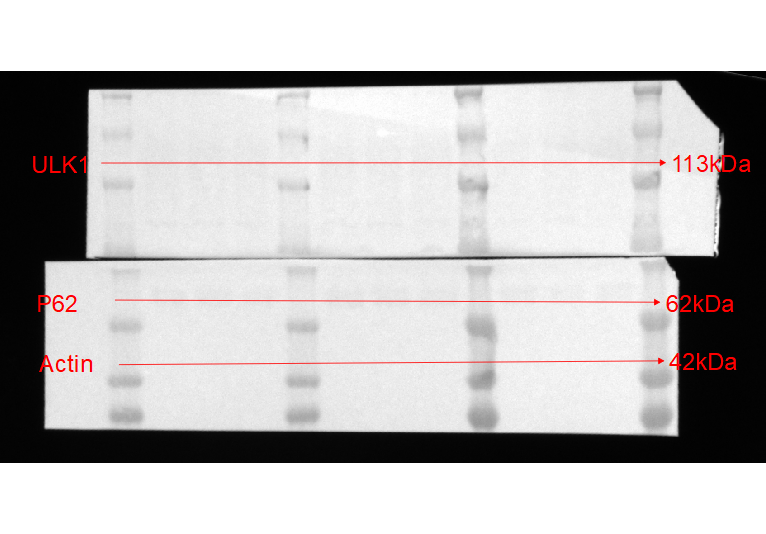

Supplement: S1 File — (ZIP) [file pone.0324264.s001.zip › supplement.material-1/western blot/cell/WB Original strips/1-total maker.tif]

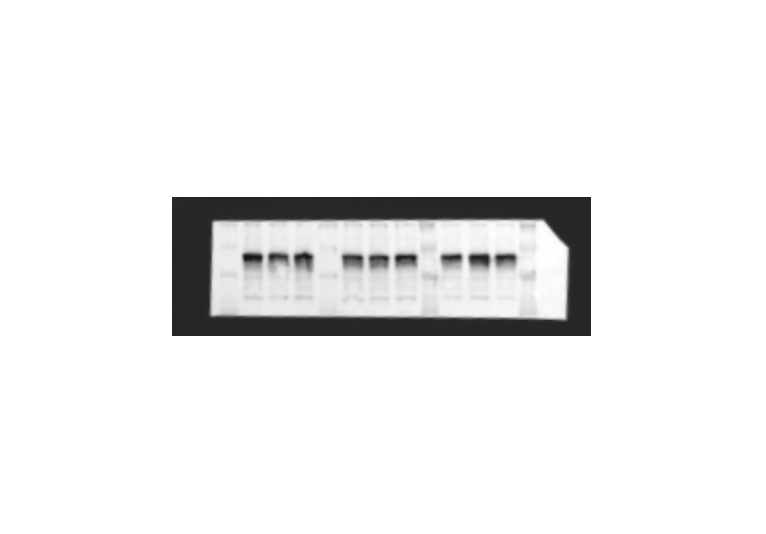

Supplement: S1 File — (ZIP) [file pone.0324264.s001.zip › supplement.material-1/western blot/cell/WB Original strips/1-ULK1-M+ULK1.tif]

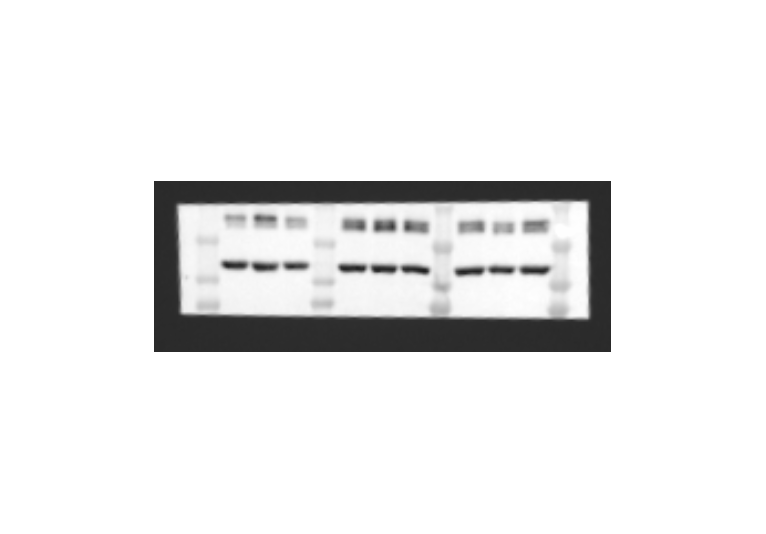

Supplement: S1 File — (ZIP) [file pone.0324264.s001.zip › supplement.material-1/western blot/cell/WB Original strips/2-ACTIN+ACTIN-M.tif]

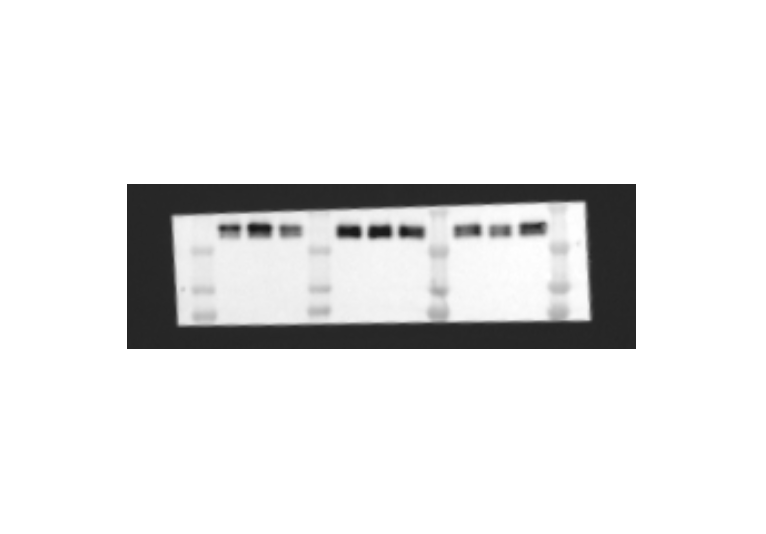

Supplement: S1 File — (ZIP) [file pone.0324264.s001.zip › supplement.material-1/western blot/cell/WB Original strips/2-P62+P62-M.tif]

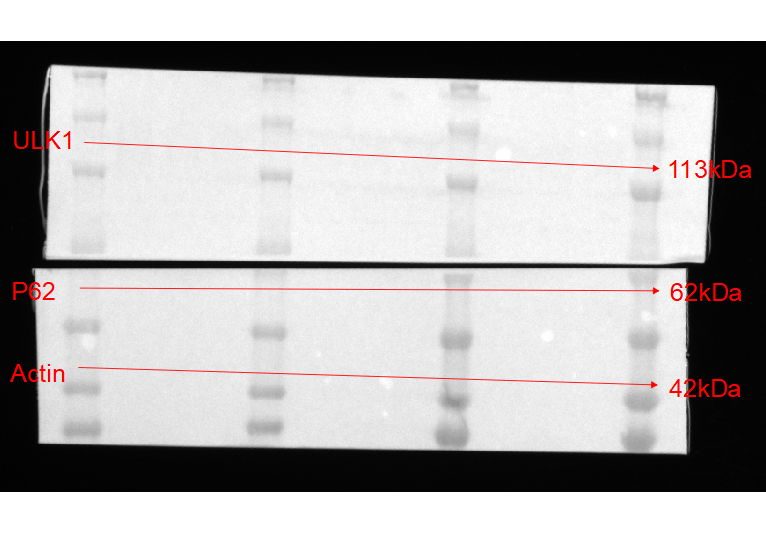

Supplement: S1 File — (ZIP) [file pone.0324264.s001.zip › supplement.material-1/western blot/cell/WB Original strips/2-total maker.tif]

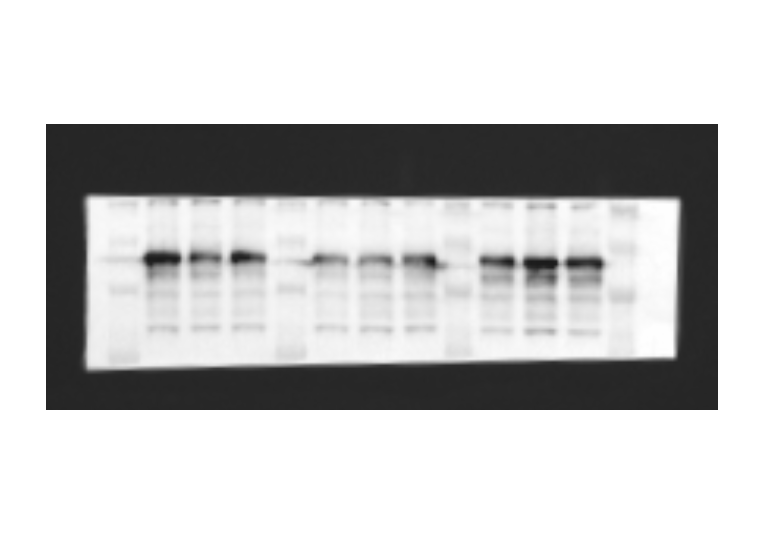

Supplement: S1 File — (ZIP) [file pone.0324264.s001.zip › supplement.material-1/western blot/cell/WB Original strips/2-ULK1+ULK1-M.tif]

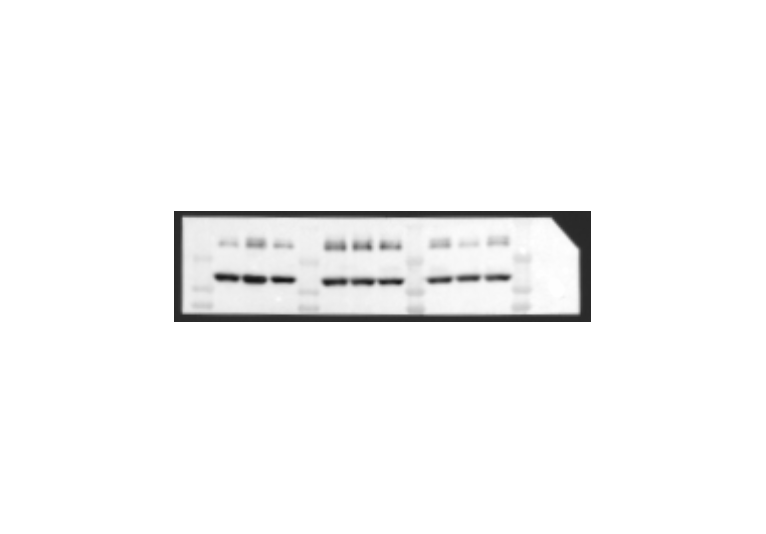

Supplement: S1 File — (ZIP) [file pone.0324264.s001.zip › supplement.material-1/western blot/cell/WB Original strips/3-ACTIN-M+ACTIN.tif]

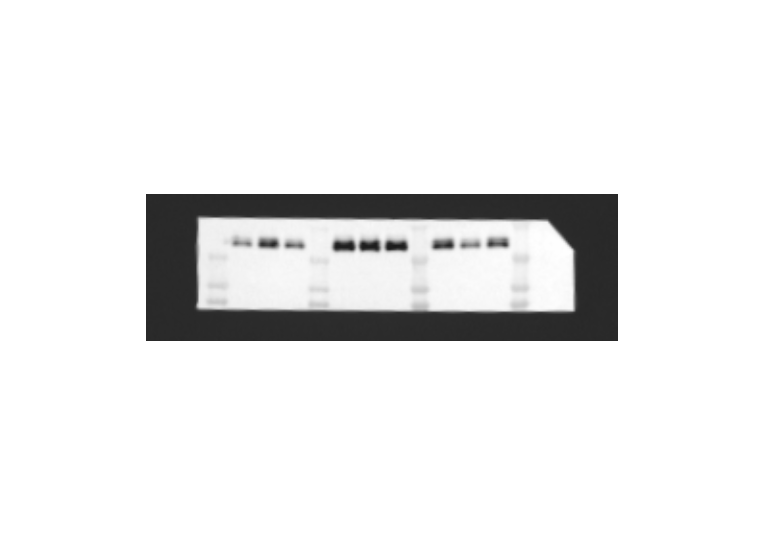

Supplement: S1 File — (ZIP) [file pone.0324264.s001.zip › supplement.material-1/western blot/cell/WB Original strips/3-P62-M+P62.tif]

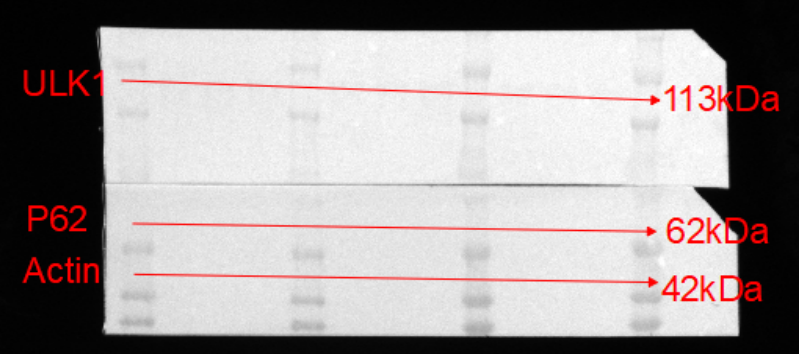

Supplement: S1 File — (ZIP) [file pone.0324264.s001.zip › supplement.material-1/western blot/cell/WB Original strips/3-total maker.jpg]

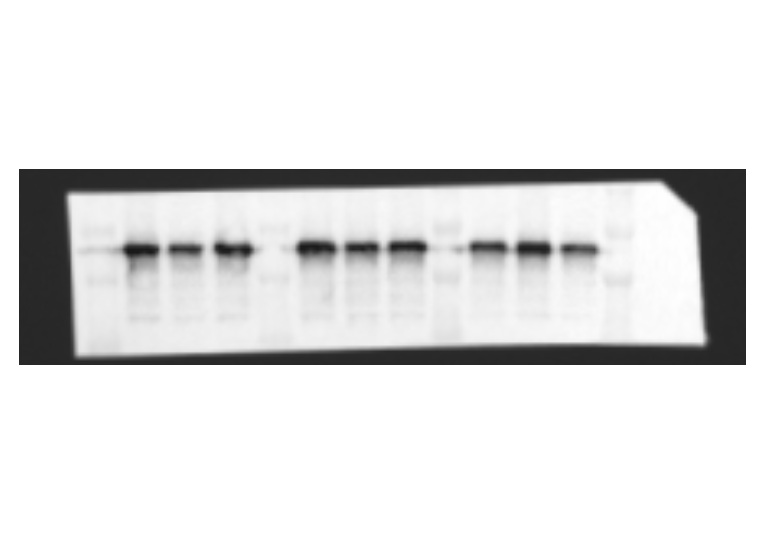

Supplement: S1 File — (ZIP) [file pone.0324264.s001.zip › supplement.material-1/western blot/cell/WB Original strips/3-ULK1-M+ULK1.tif]

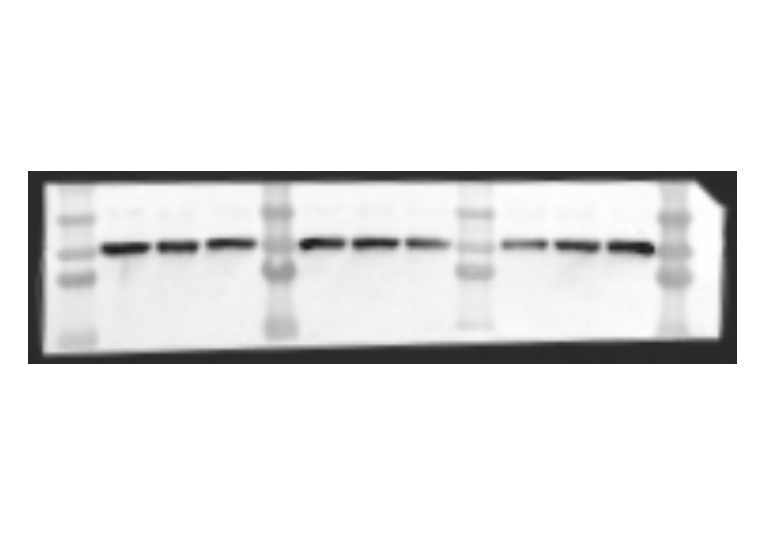

Supplement: S1 File — (ZIP) [file pone.0324264.s001.zip › supplement.material-1/western blot/cell/WB Original strips/4-actin-M+actin.tif]

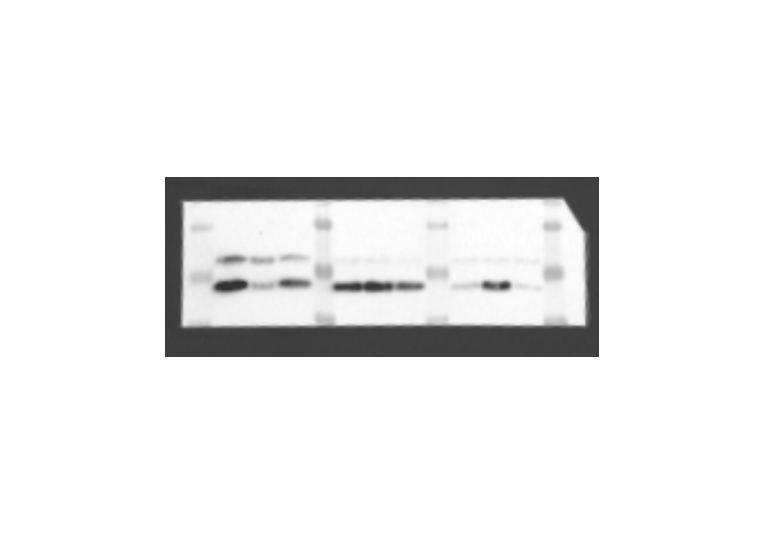

Supplement: S1 File — (ZIP) [file pone.0324264.s001.zip › supplement.material-1/western blot/cell/WB Original strips/4-LC3B-M+LC3B.tif]

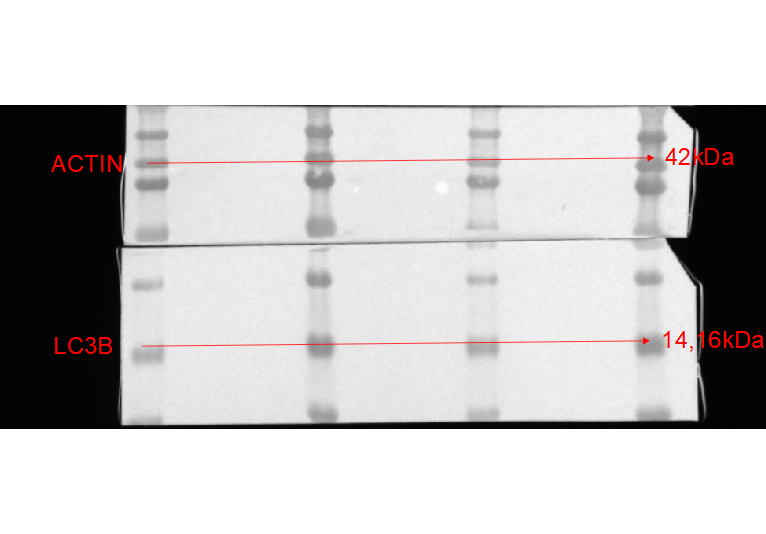

Supplement: S1 File — (ZIP) [file pone.0324264.s001.zip › supplement.material-1/western blot/cell/WB Original strips/4-total maker.tif]

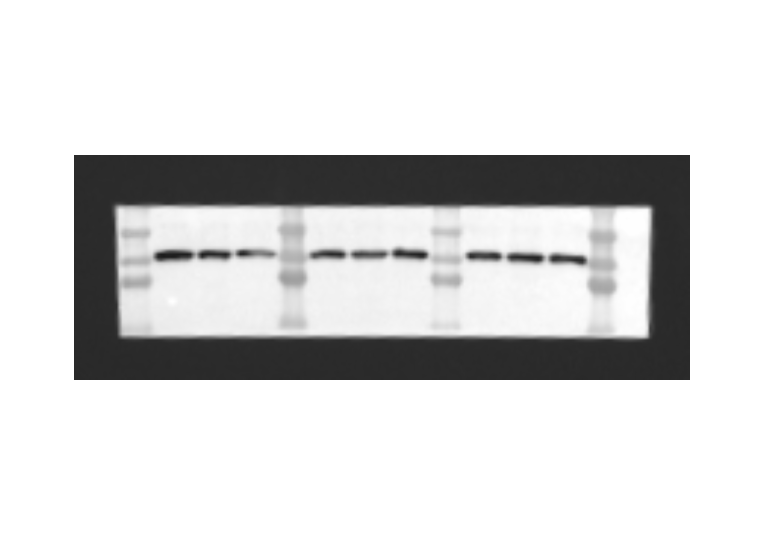

Supplement: S1 File — (ZIP) [file pone.0324264.s001.zip › supplement.material-1/western blot/cell/WB Original strips/5-actin+actin-M.tif]

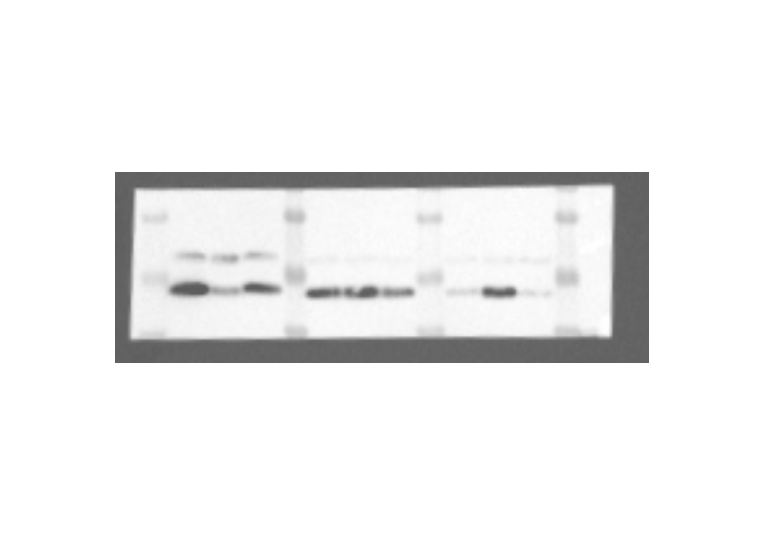

Supplement: S1 File — (ZIP) [file pone.0324264.s001.zip › supplement.material-1/western blot/cell/WB Original strips/5-LC3B-M+LC3B.tif]

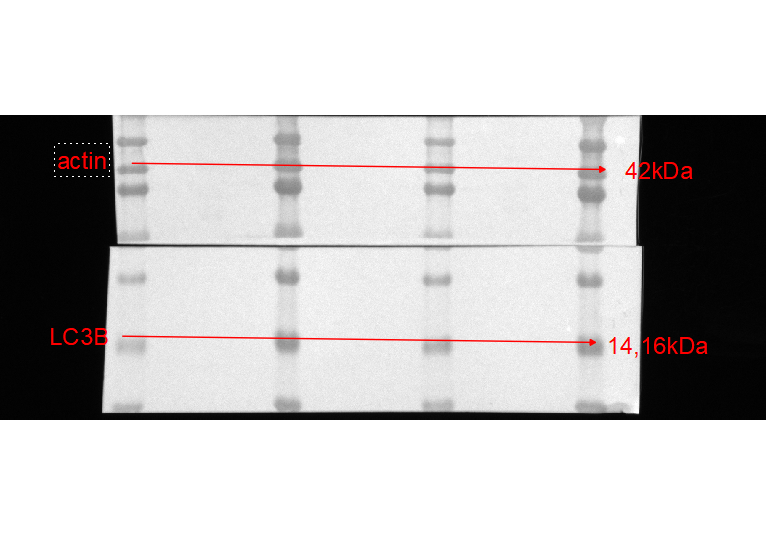

Supplement: S1 File — (ZIP) [file pone.0324264.s001.zip › supplement.material-1/western blot/cell/WB Original strips/5-total maker.tif]

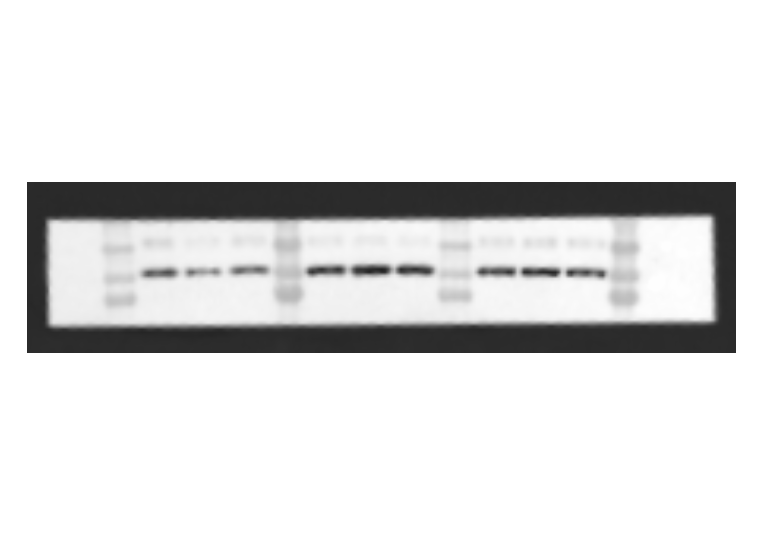

Supplement: S1 File — (ZIP) [file pone.0324264.s001.zip › supplement.material-1/western blot/cell/WB Original strips/6-ACTIN+ACTIN-M.tif]

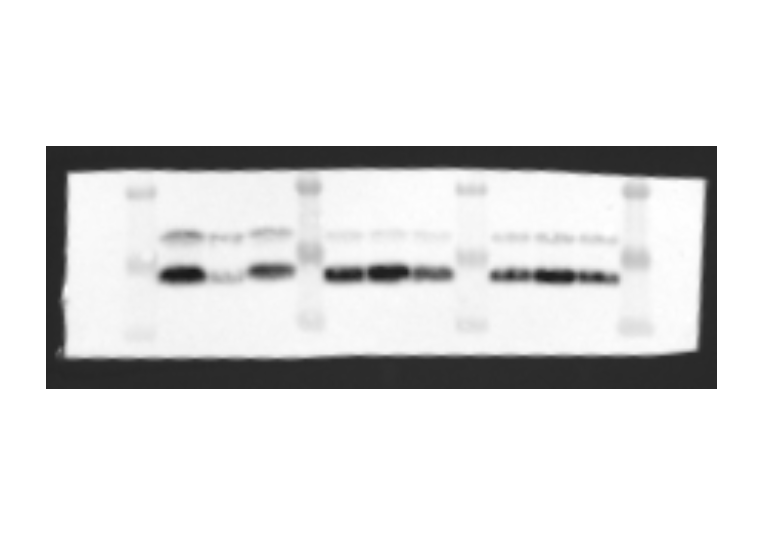

Supplement: S1 File — (ZIP) [file pone.0324264.s001.zip › supplement.material-1/western blot/cell/WB Original strips/6-LC3B-M+LC3B.tif]

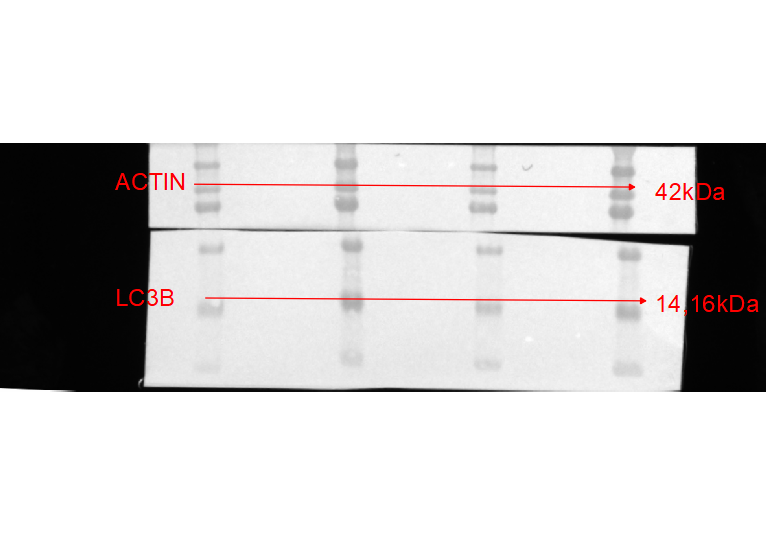

Supplement: S1 File — (ZIP) [file pone.0324264.s001.zip › supplement.material-1/western blot/cell/WB Original strips/6-total maker.tif]

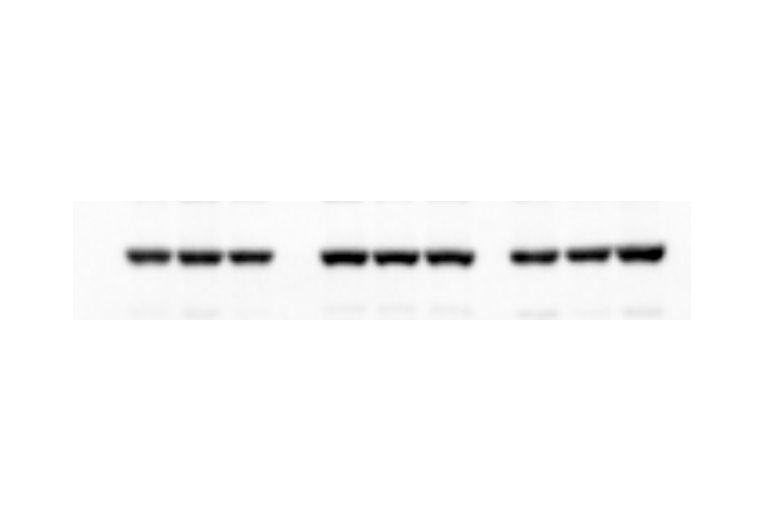

Supplement: S1 File — (ZIP) [file pone.0324264.s001.zip › supplement.material-1/western blot/cell/WB processing strips/1-ACTIN.tif]

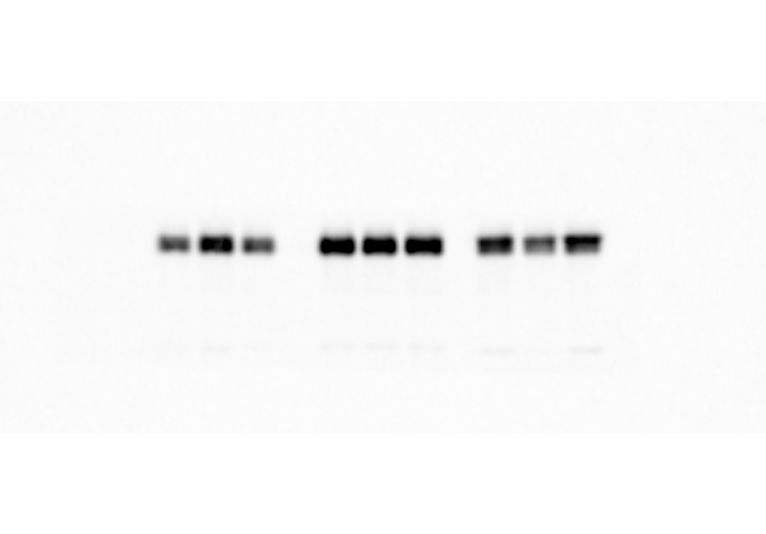

Supplement: S1 File — (ZIP) [file pone.0324264.s001.zip › supplement.material-1/western blot/cell/WB processing strips/1-P62.tif]

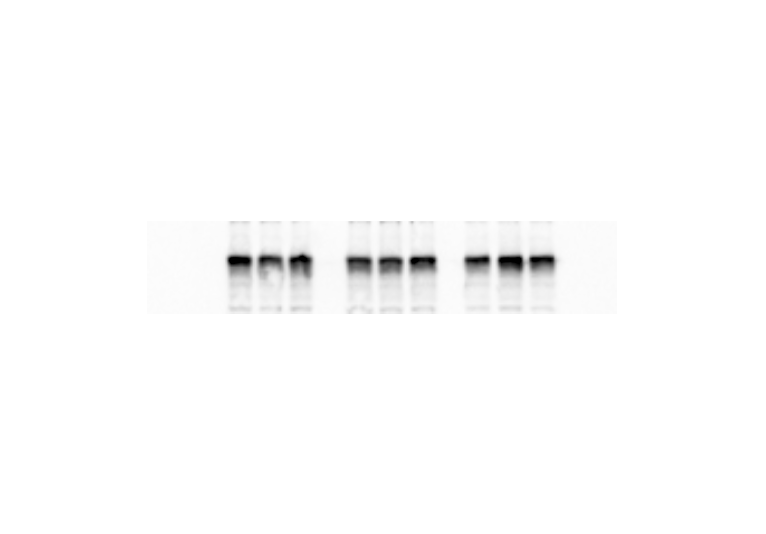

Supplement: S1 File — (ZIP) [file pone.0324264.s001.zip › supplement.material-1/western blot/cell/WB processing strips/1-ULK1.tif]

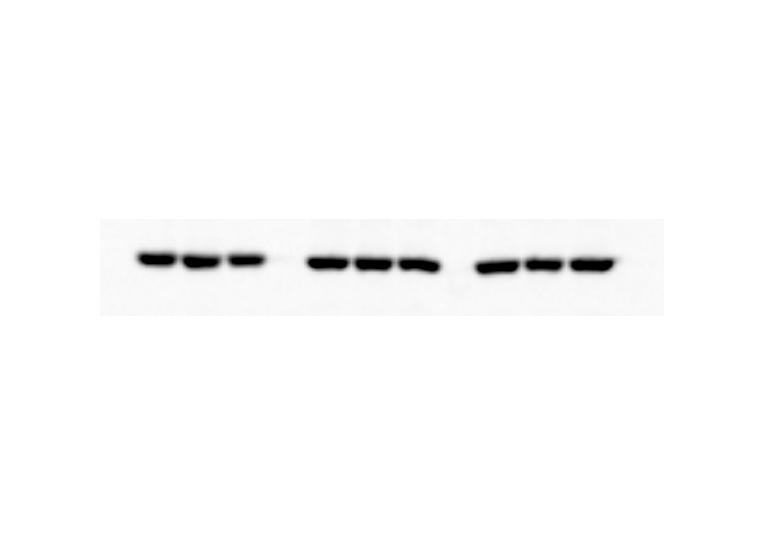

Supplement: S1 File — (ZIP) [file pone.0324264.s001.zip › supplement.material-1/western blot/cell/WB processing strips/2-ACTIN.tif]

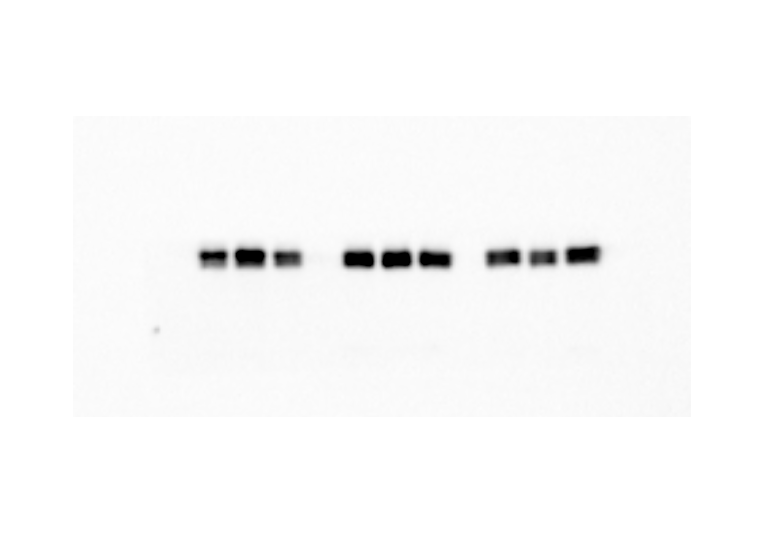

Supplement: S1 File — (ZIP) [file pone.0324264.s001.zip › supplement.material-1/western blot/cell/WB processing strips/2-P62.tif]

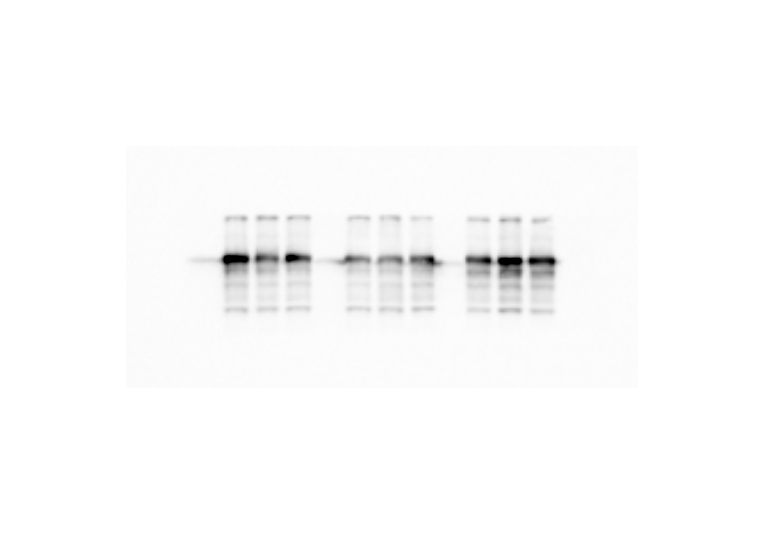

Supplement: S1 File — (ZIP) [file pone.0324264.s001.zip › supplement.material-1/western blot/cell/WB processing strips/2-ULK1.tif]

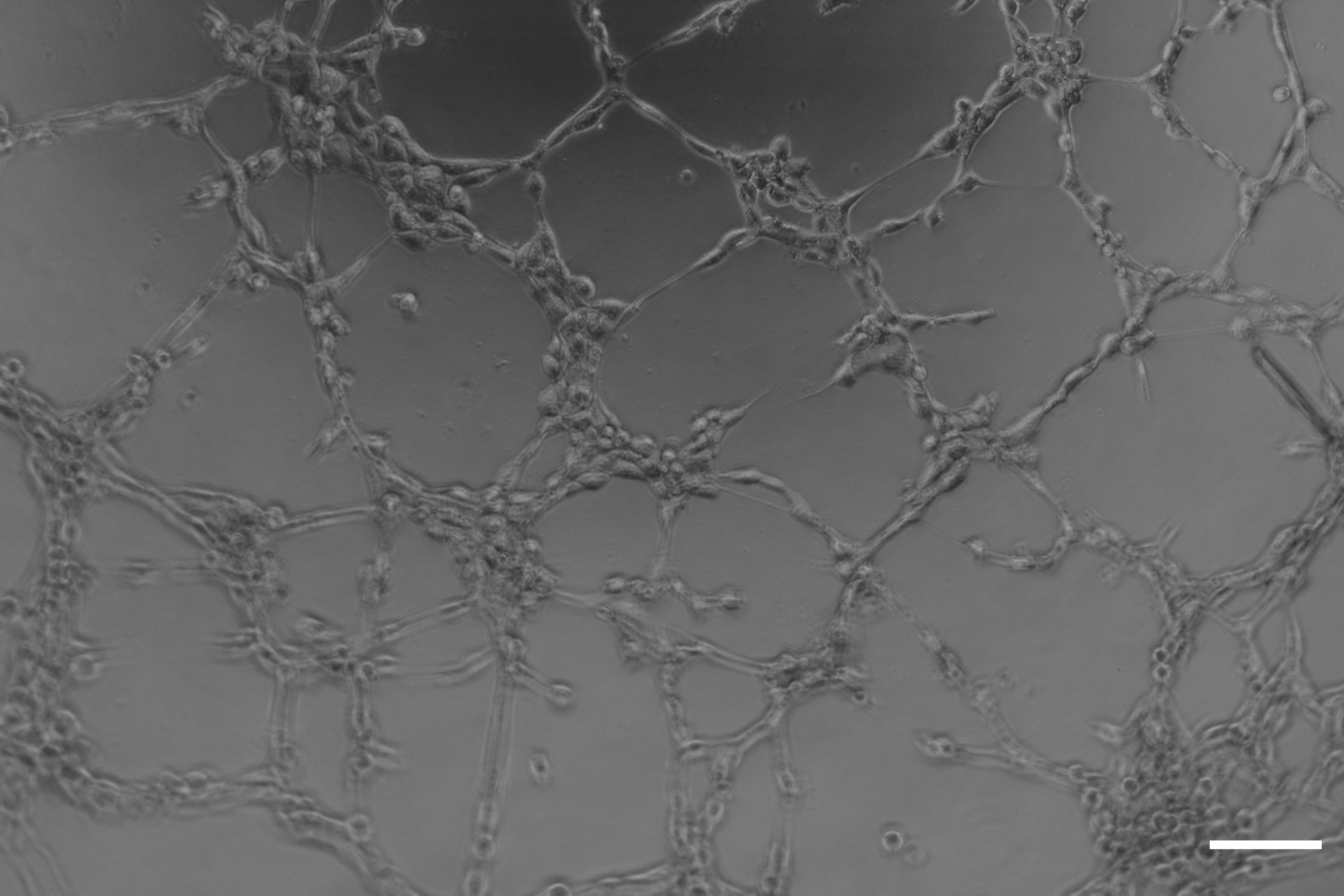

Supplement: S2 File — (ZIP) [file pone.0324264.s002.zip › supplement.material-2/images(tube formation assay)/144-control1.jpg]

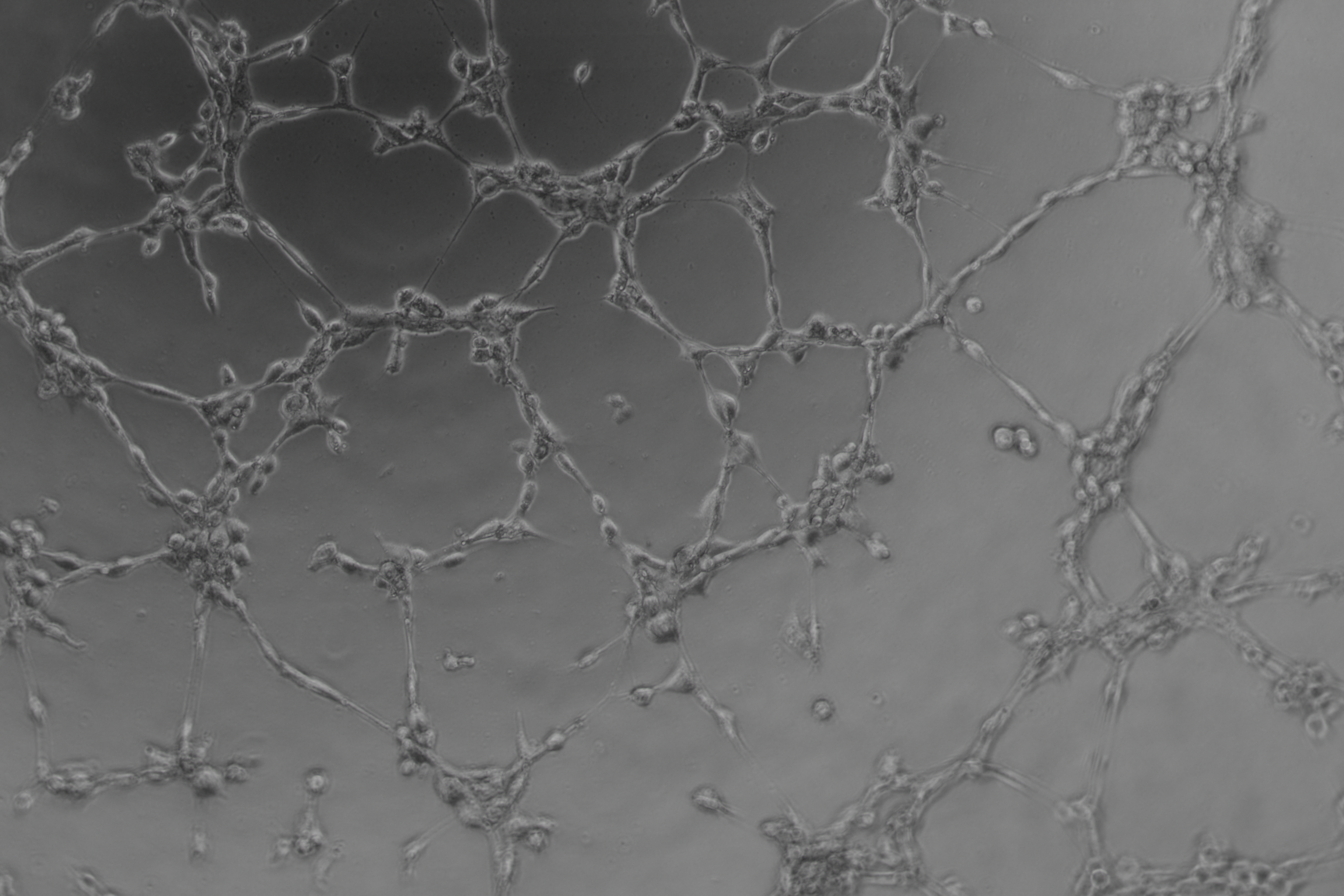

Supplement: S2 File — (ZIP) [file pone.0324264.s002.zip › supplement.material-2/images(tube formation assay)/144-control2.tif]

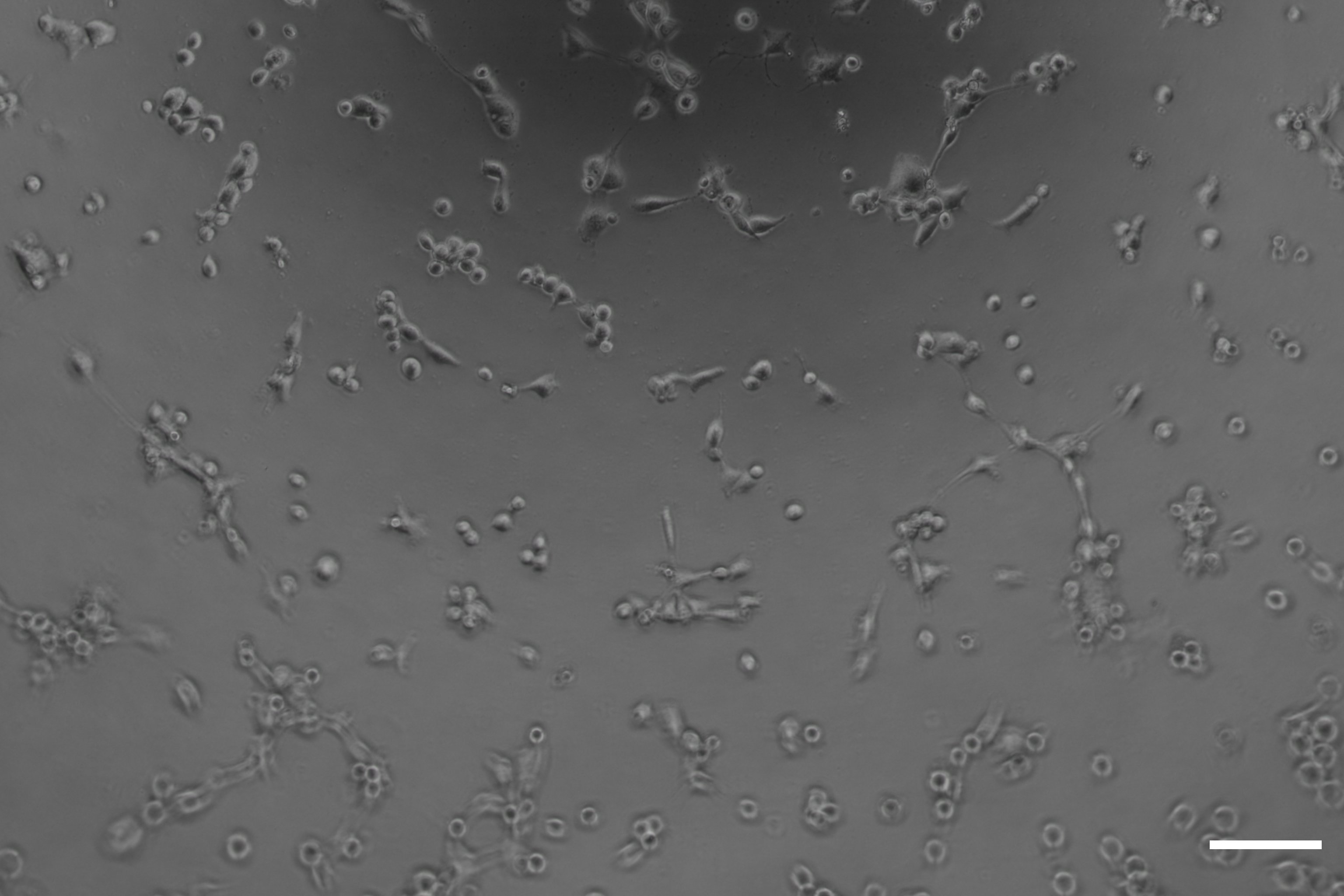

Supplement: S2 File — (ZIP) [file pone.0324264.s002.zip › supplement.material-2/images(tube formation assay)/144-model1.jpg]
