# Supplementary figures and images for: Platelet lysate-sodium hyaluronate gel promotes diabetic foot wound healing by regulating oxidative stress and autophagy (part 1 of 4)
Source: PLoS One. 2025 Jun 6;20(6):e0324264. doi: 10.1371/journal.pone.0324264 (PMC12143543; doi:10.1371/journal.pone.0324264)

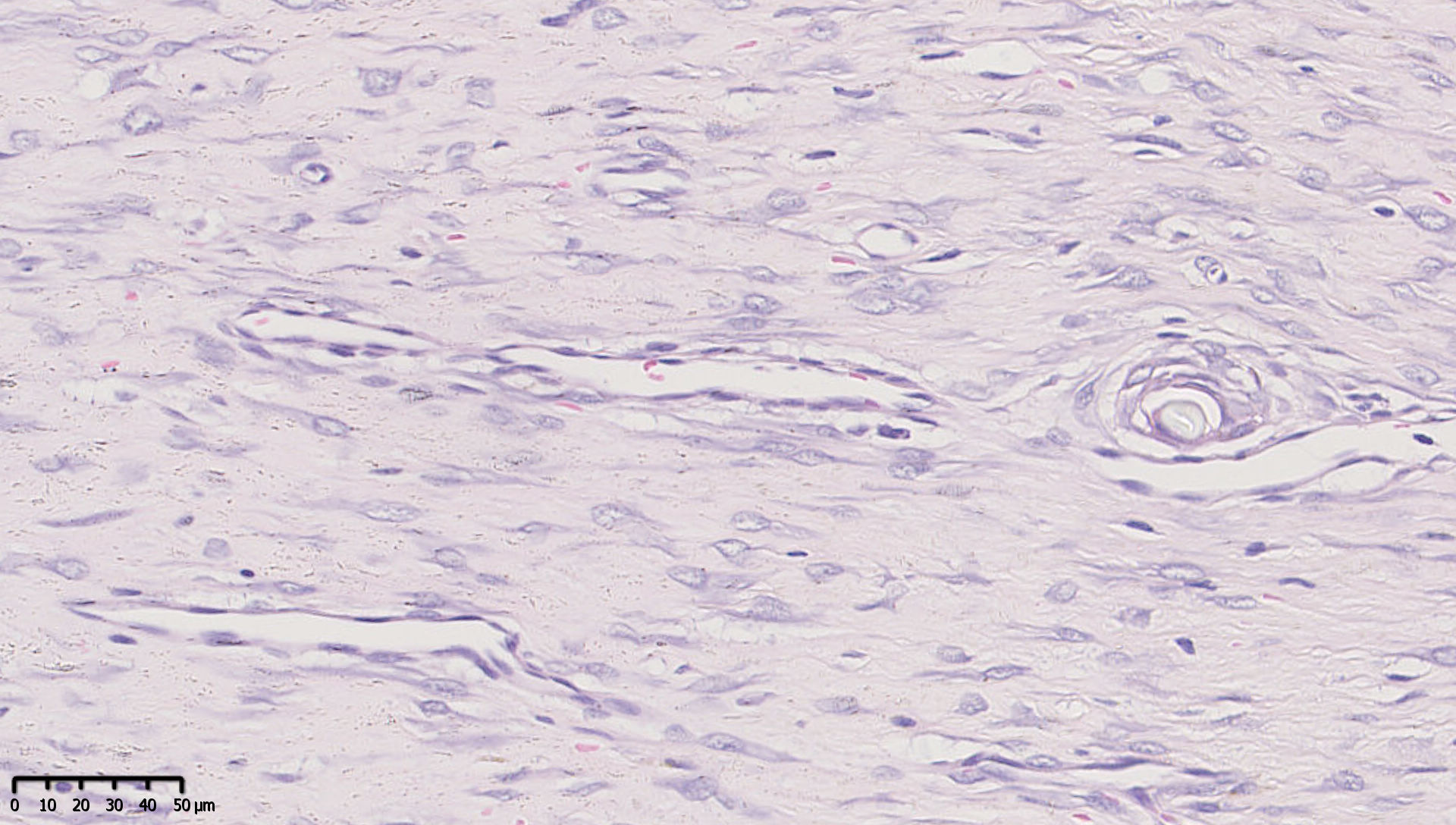

Supplement: S1 File — (ZIP) [file pone.0324264.s001.zip › supplement.material-1/HE triple section image/control-1 400x.jpg]

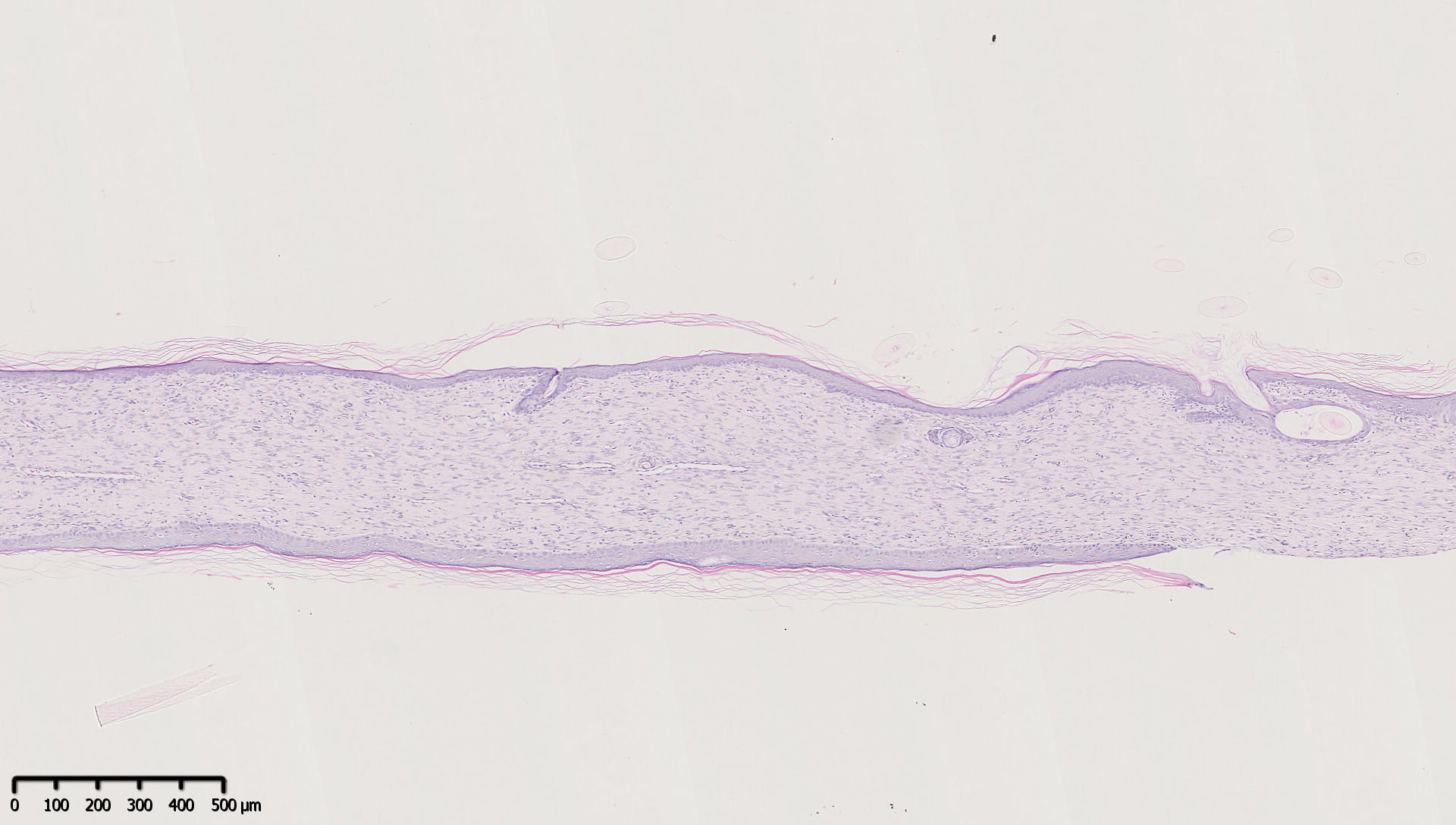

Supplement: S1 File — (ZIP) [file pone.0324264.s001.zip › supplement.material-1/HE triple section image/control-1 50x.jpg]

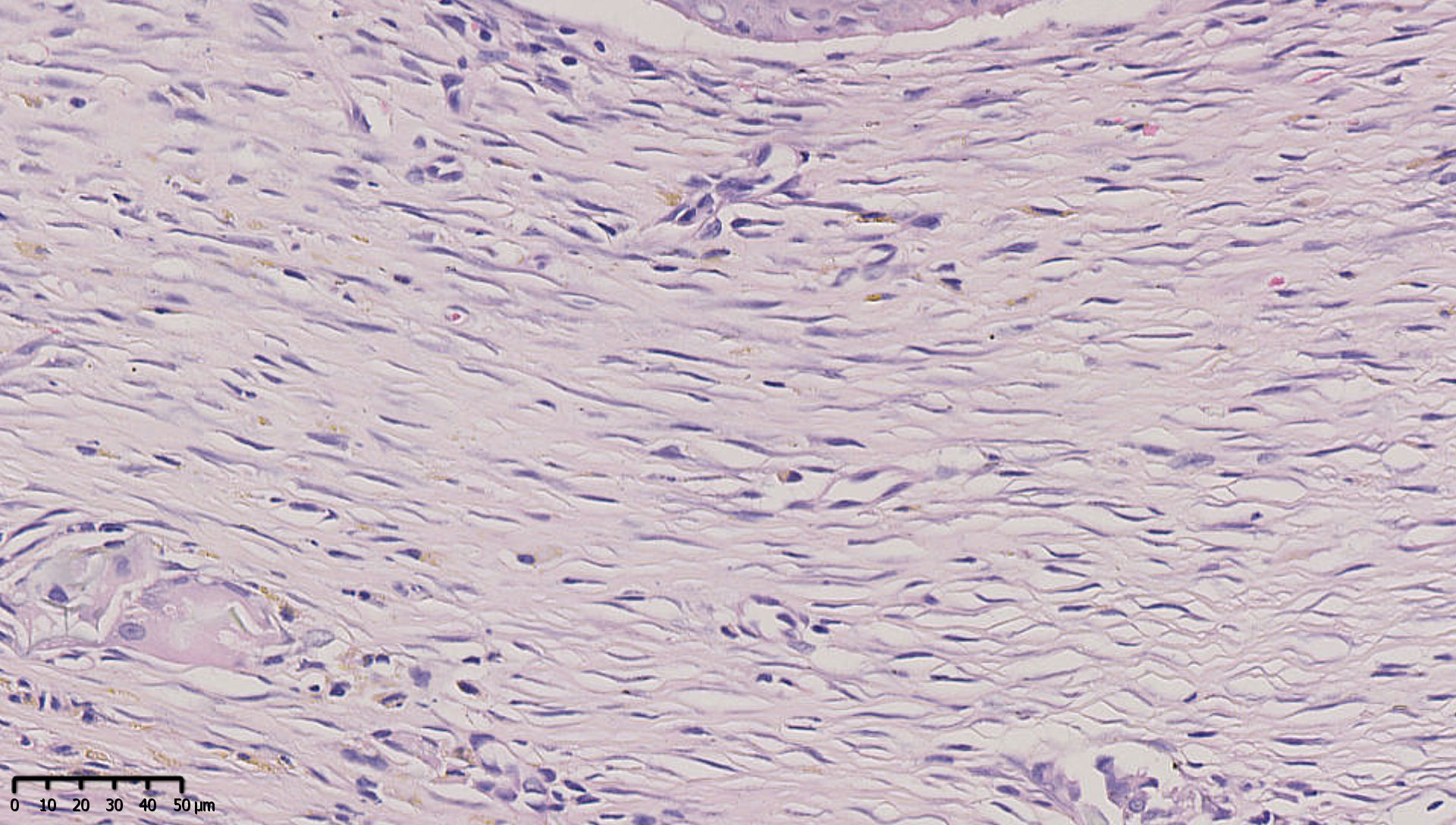

Supplement: S1 File — (ZIP) [file pone.0324264.s001.zip › supplement.material-1/HE triple section image/control-2 400X.jpg]

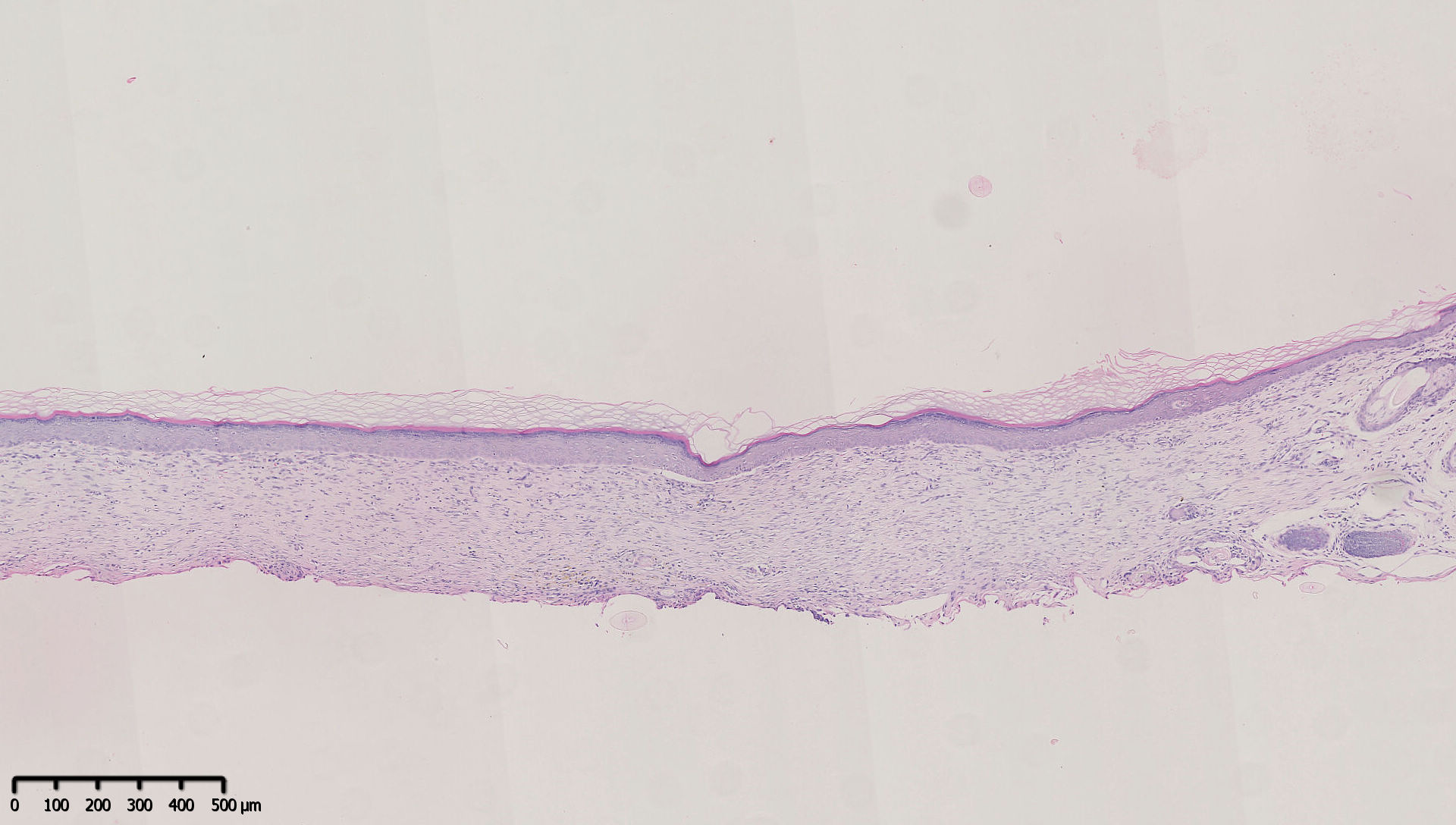

Supplement: S1 File — (ZIP) [file pone.0324264.s001.zip › supplement.material-1/HE triple section image/control-2 50X.jpg]

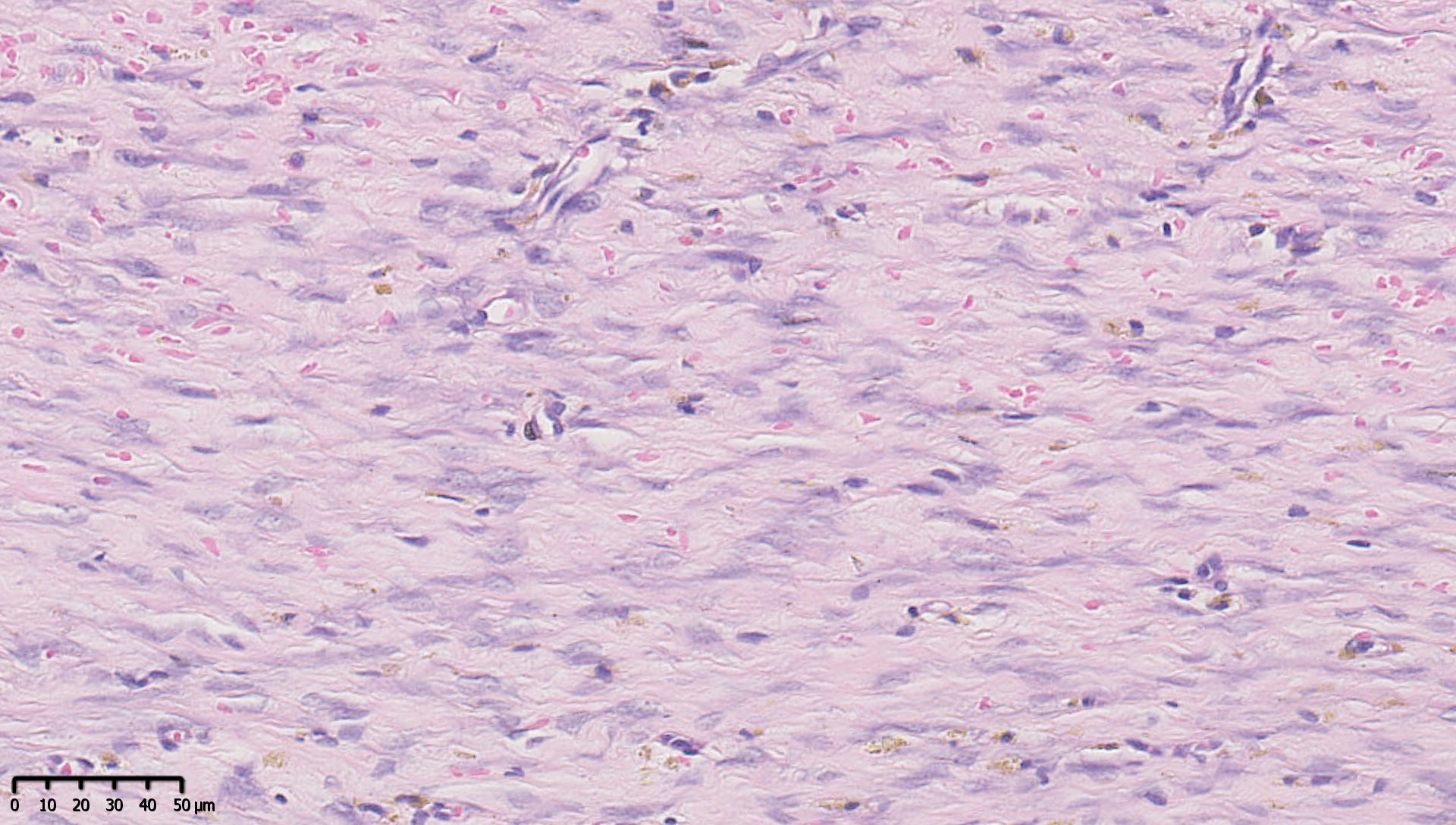

Supplement: S1 File — (ZIP) [file pone.0324264.s001.zip › supplement.material-1/HE triple section image/control-3 400x.jpg]

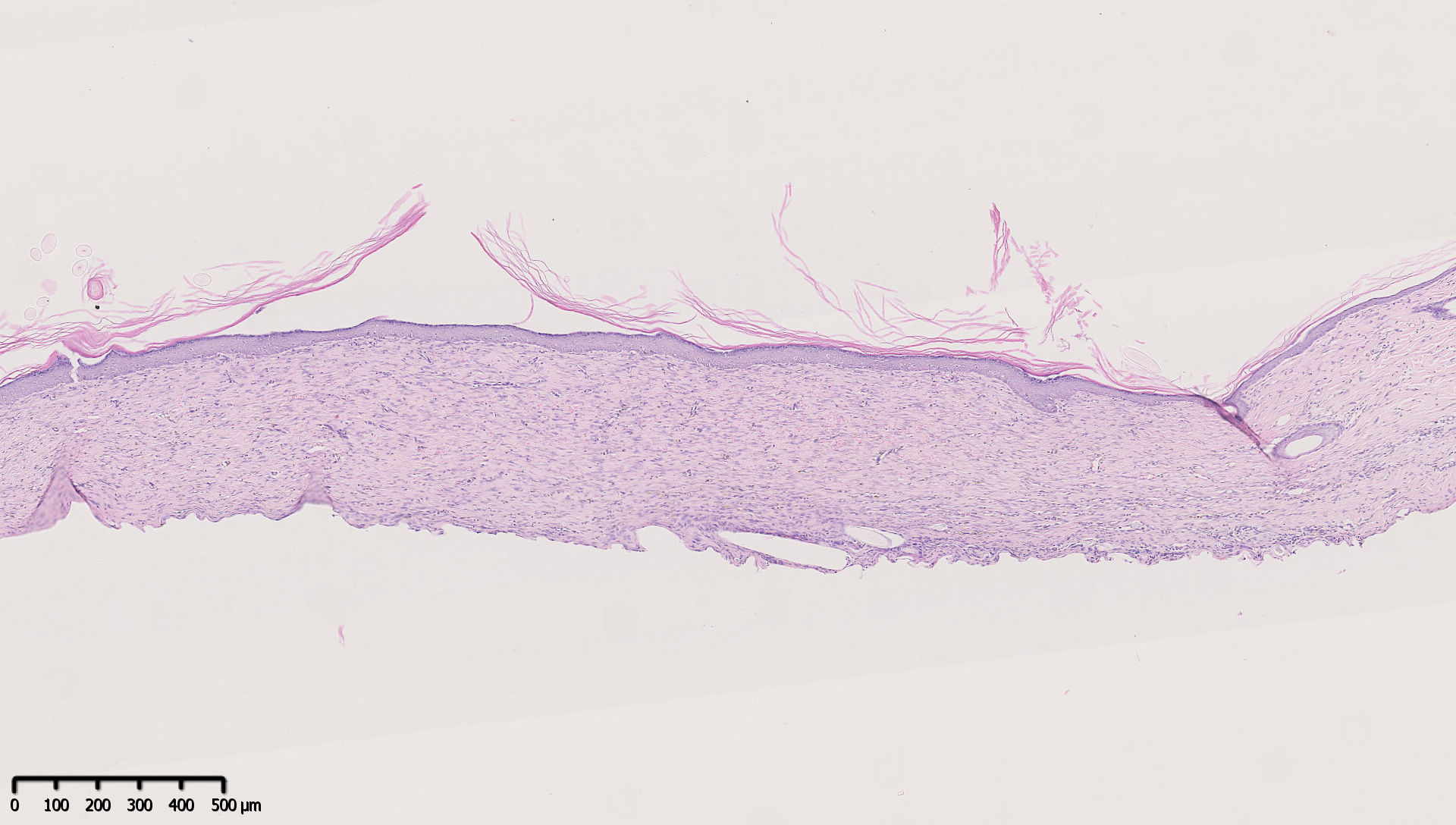

Supplement: S1 File — (ZIP) [file pone.0324264.s001.zip › supplement.material-1/HE triple section image/Control-3 50x.jpg]

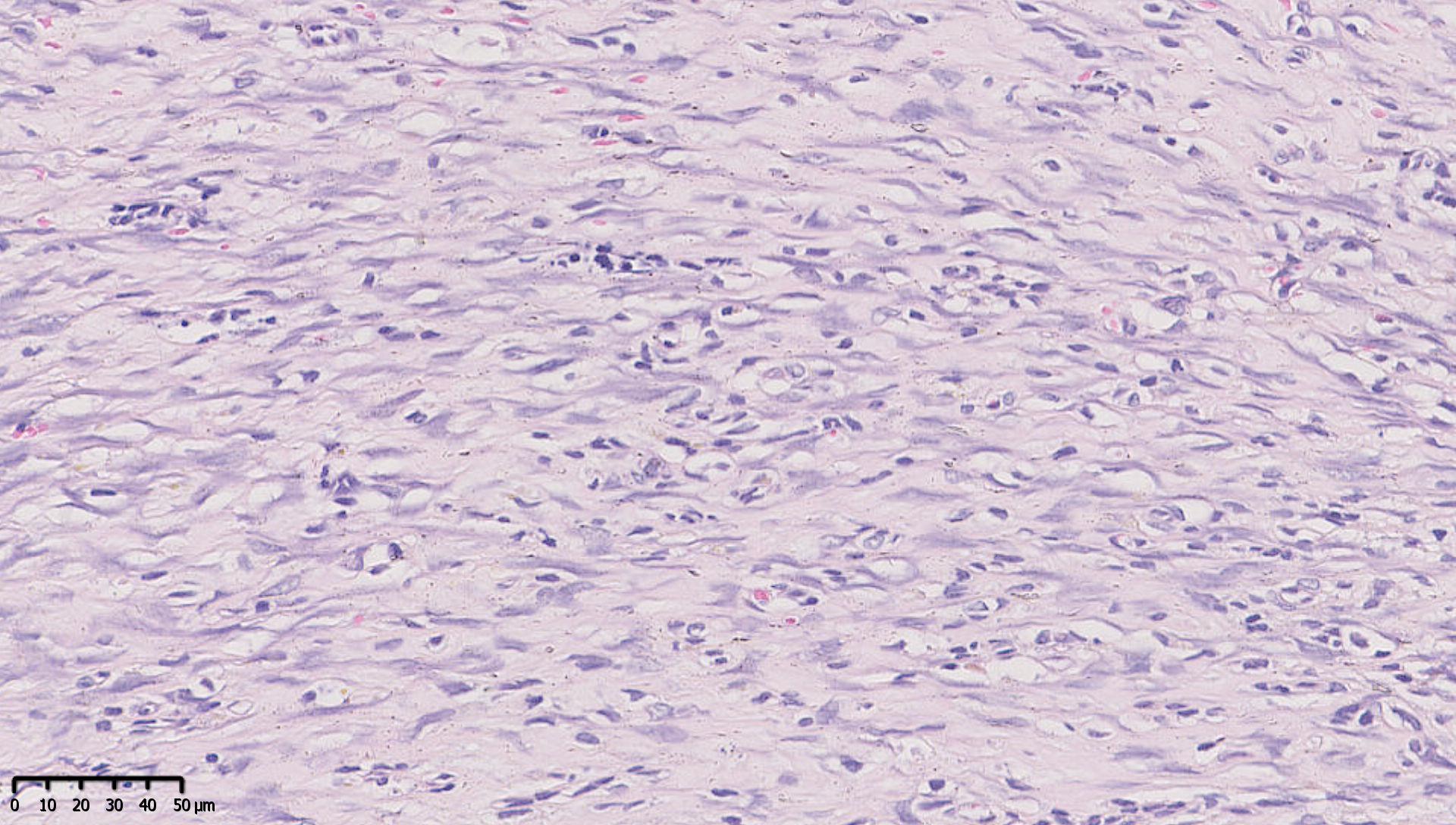

Supplement: S1 File — (ZIP) [file pone.0324264.s001.zip › supplement.material-1/HE triple section image/HA-1 400X.jpg]

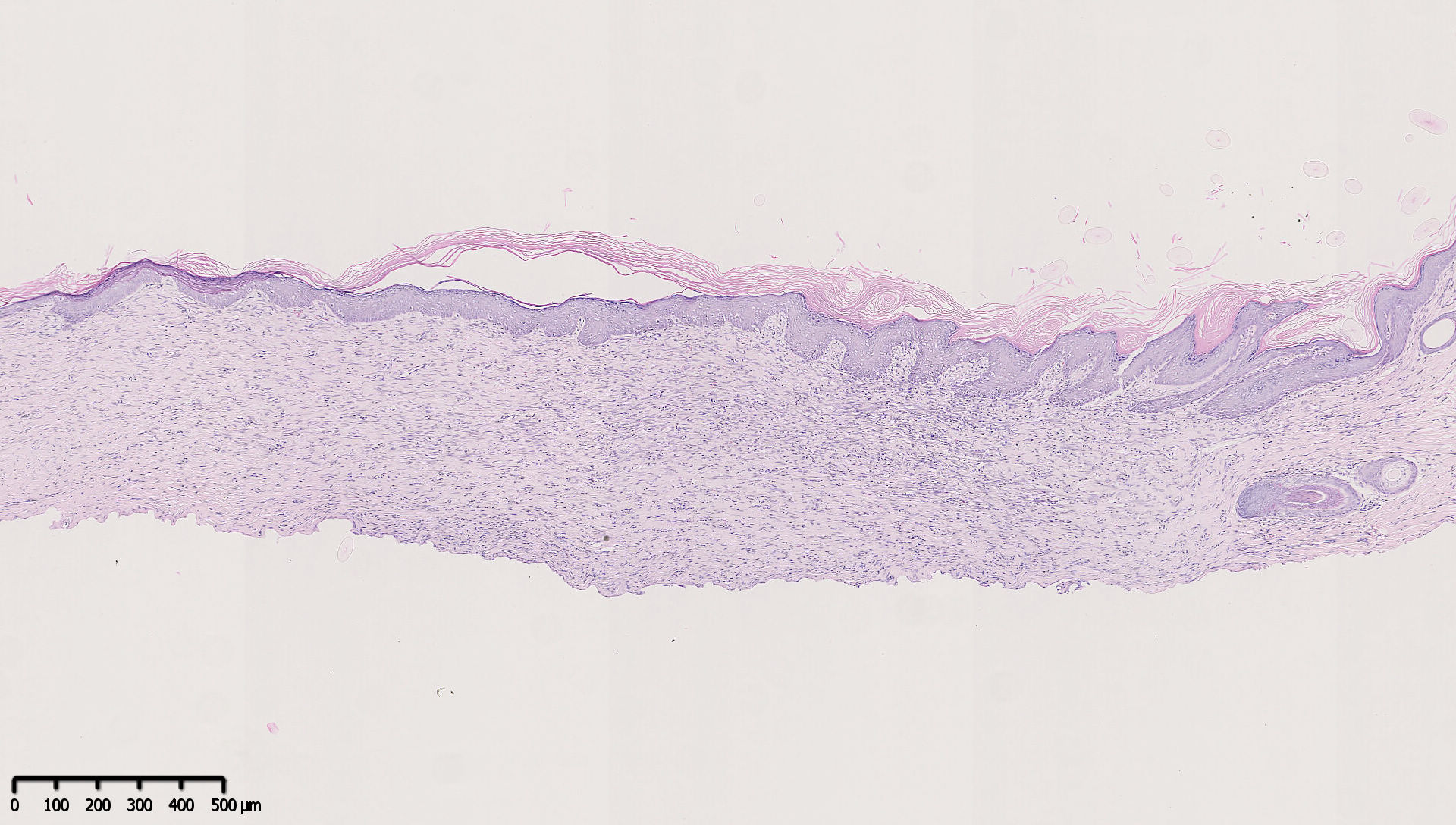

Supplement: S1 File — (ZIP) [file pone.0324264.s001.zip › supplement.material-1/HE triple section image/HA-1 50X.jpg]

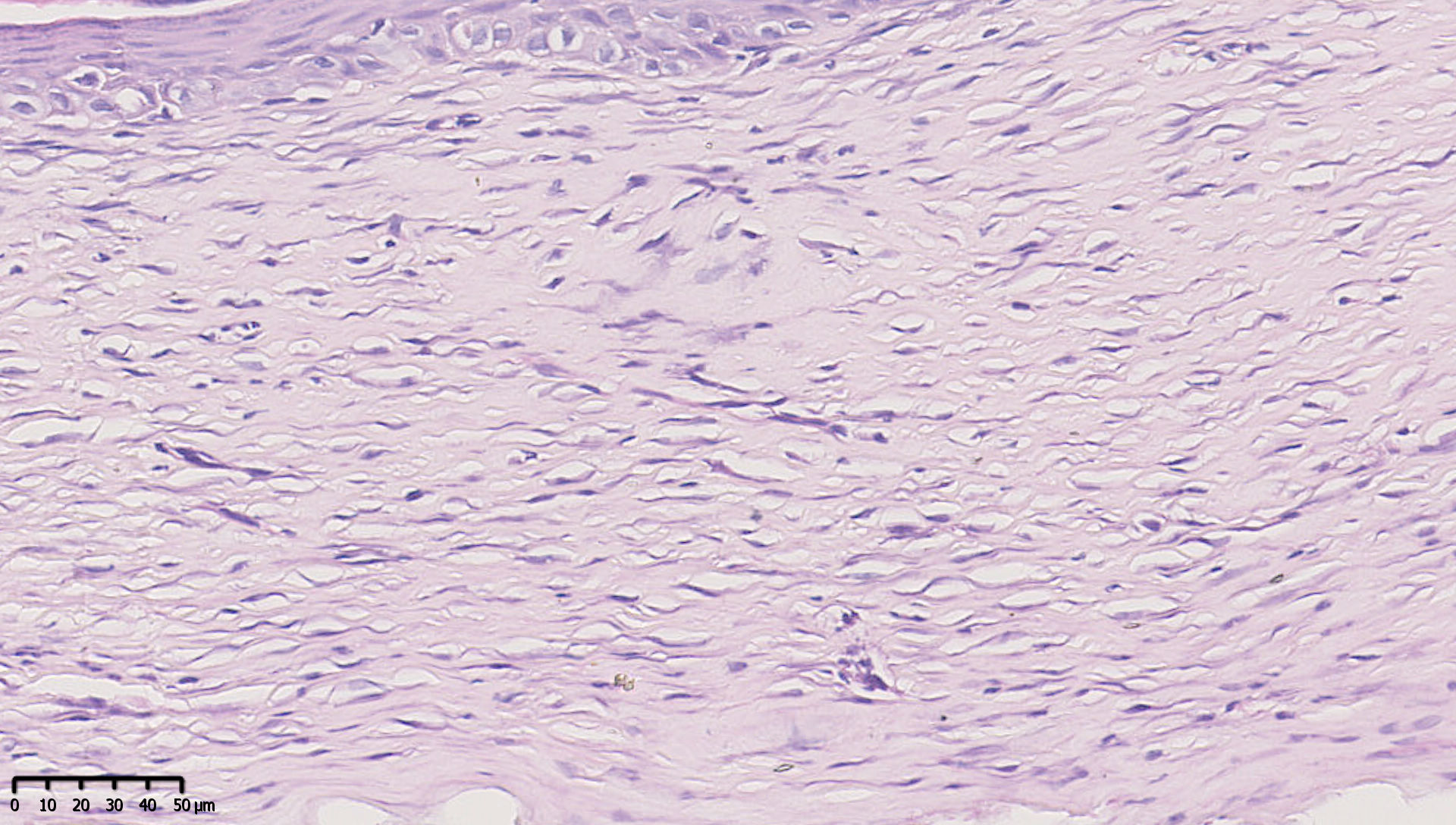

Supplement: S1 File — (ZIP) [file pone.0324264.s001.zip › supplement.material-1/HE triple section image/HA-2 400x.jpg]

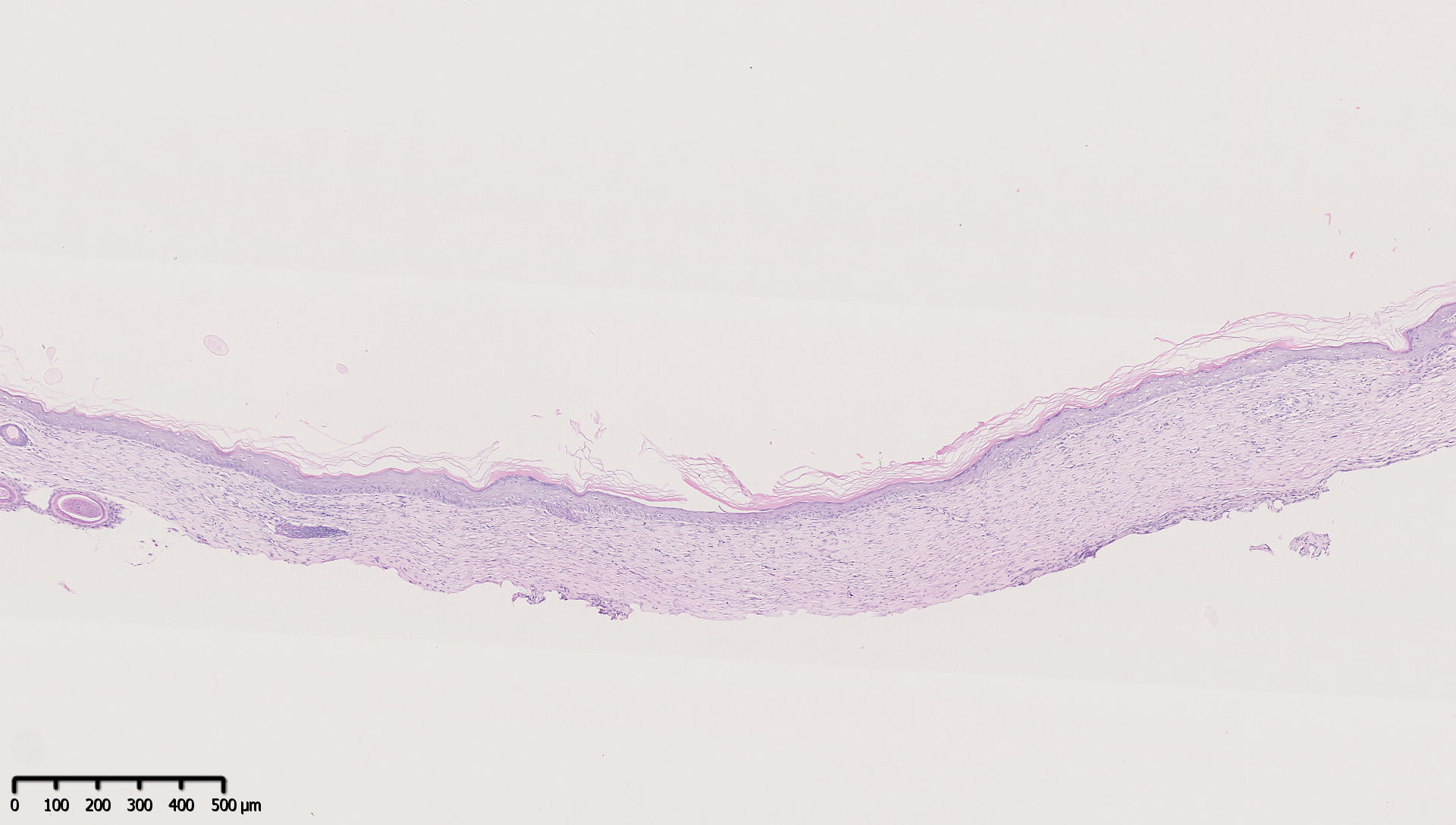

Supplement: S1 File — (ZIP) [file pone.0324264.s001.zip › supplement.material-1/HE triple section image/HA-2 50x.jpg]

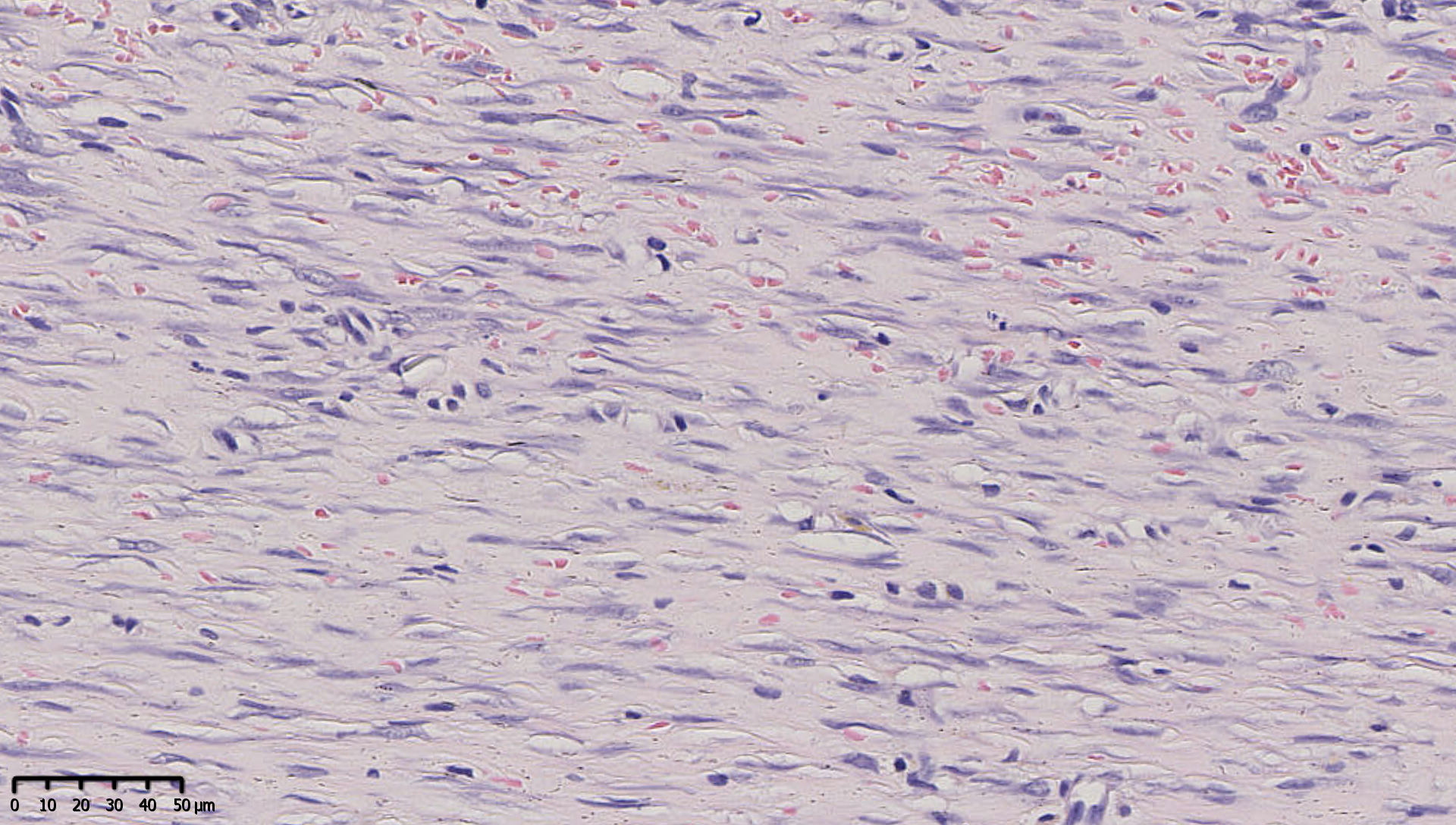

Supplement: S1 File — (ZIP) [file pone.0324264.s001.zip › supplement.material-1/HE triple section image/HA-3 400x.jpg]

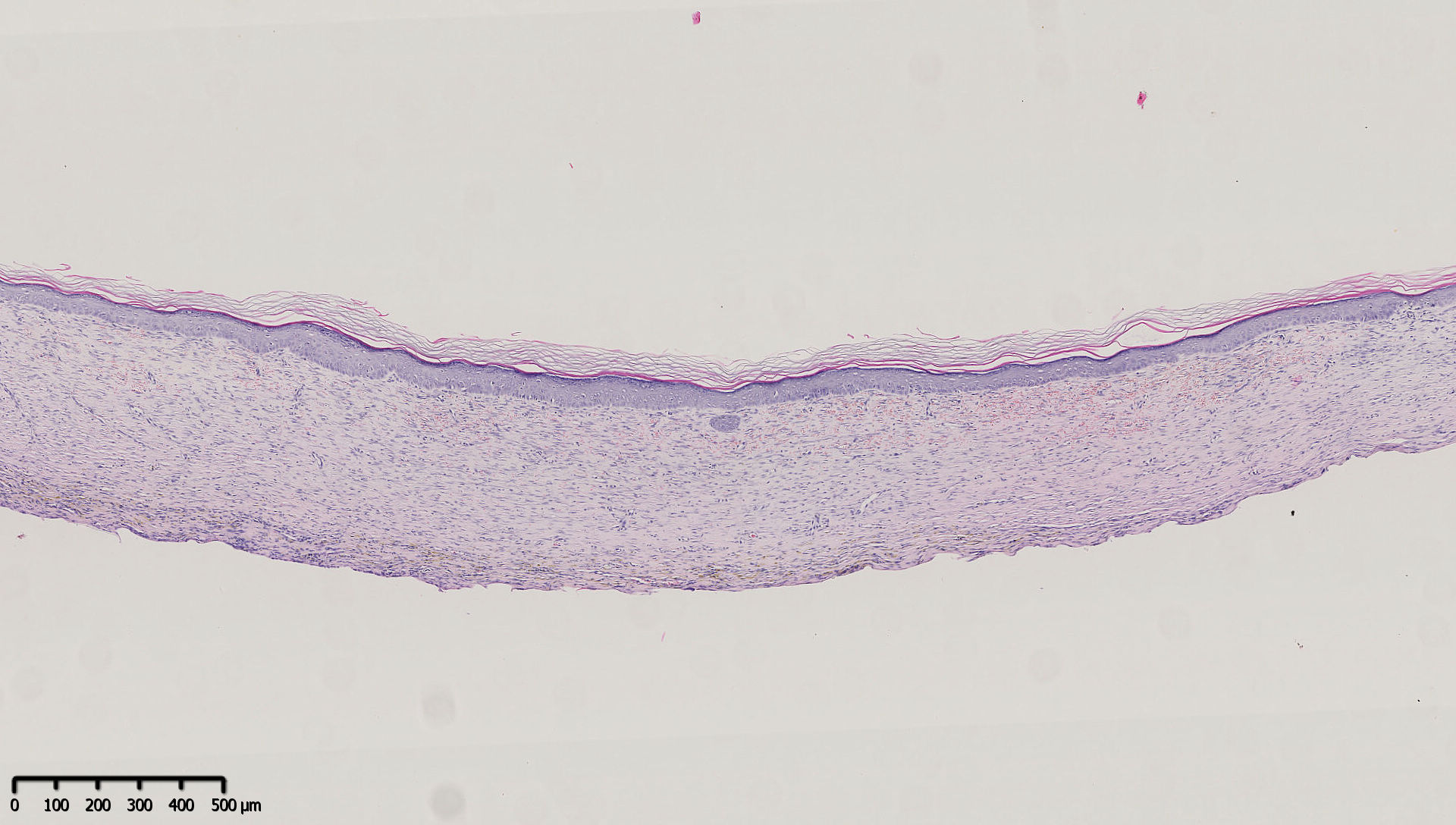

Supplement: S1 File — (ZIP) [file pone.0324264.s001.zip › supplement.material-1/HE triple section image/HA-3 50x.jpg]

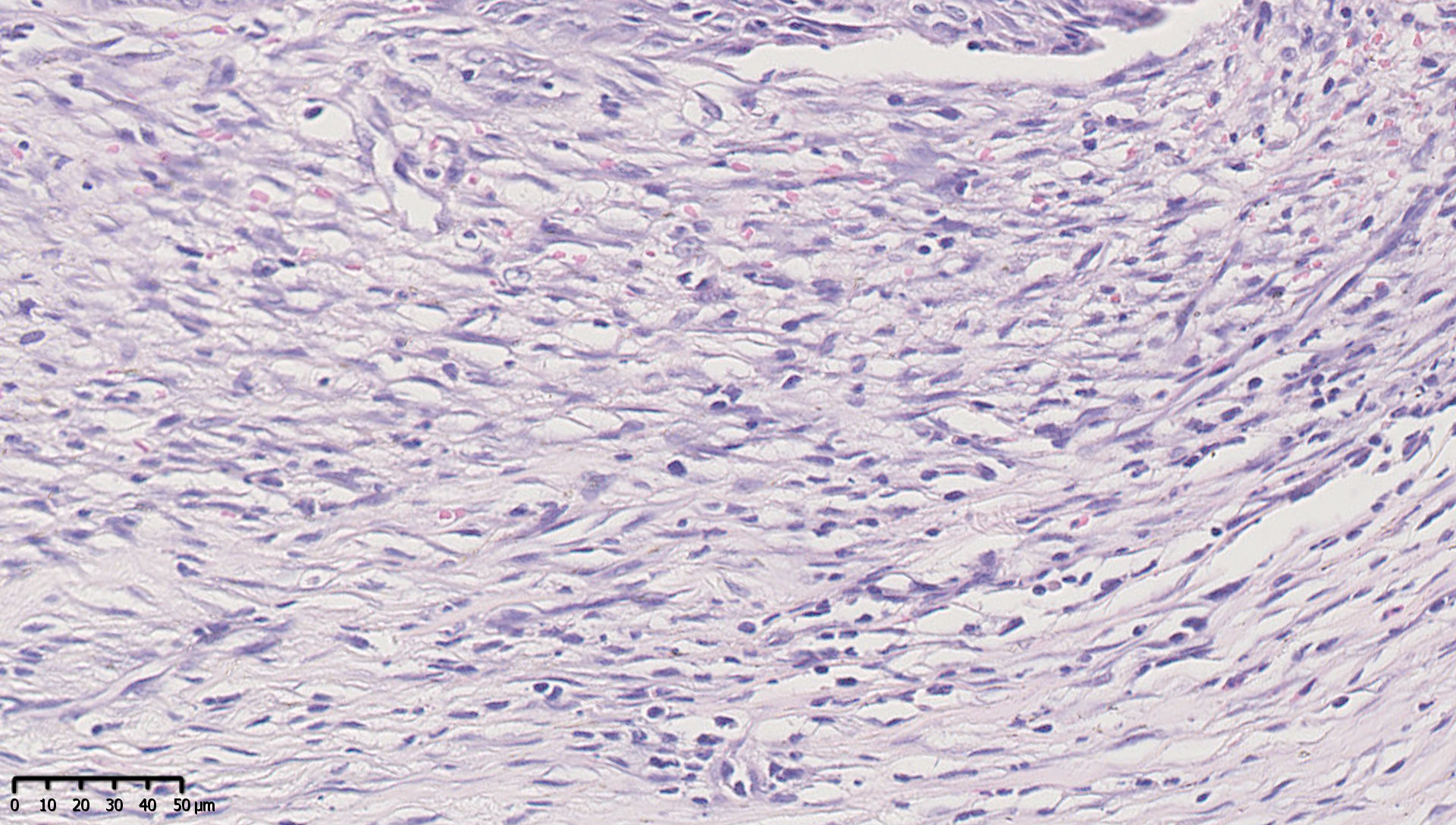

Supplement: S1 File — (ZIP) [file pone.0324264.s001.zip › supplement.material-1/HE triple section image/model-1 400x.jpg]

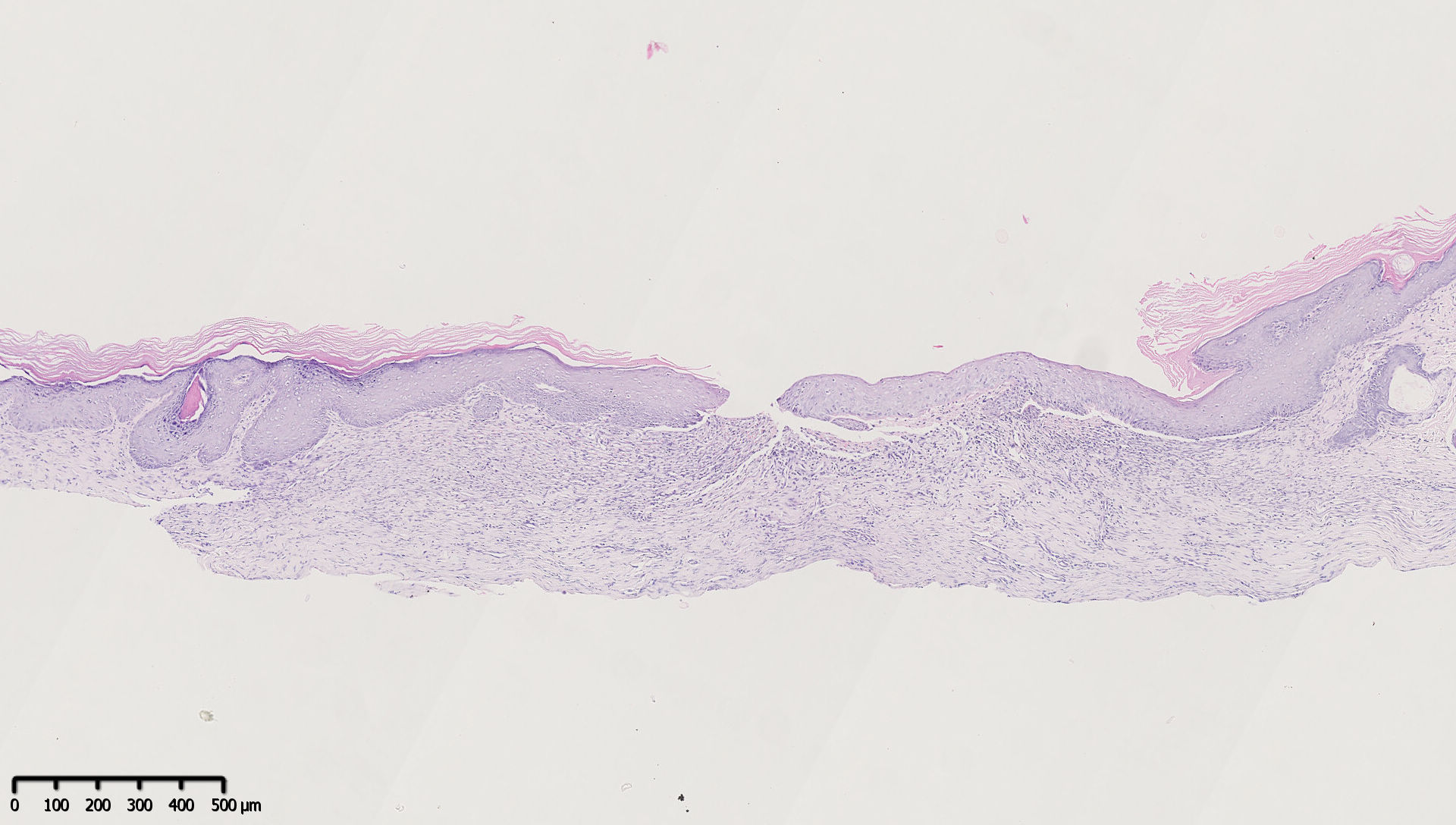

Supplement: S1 File — (ZIP) [file pone.0324264.s001.zip › supplement.material-1/HE triple section image/model-1 50x.jpg]

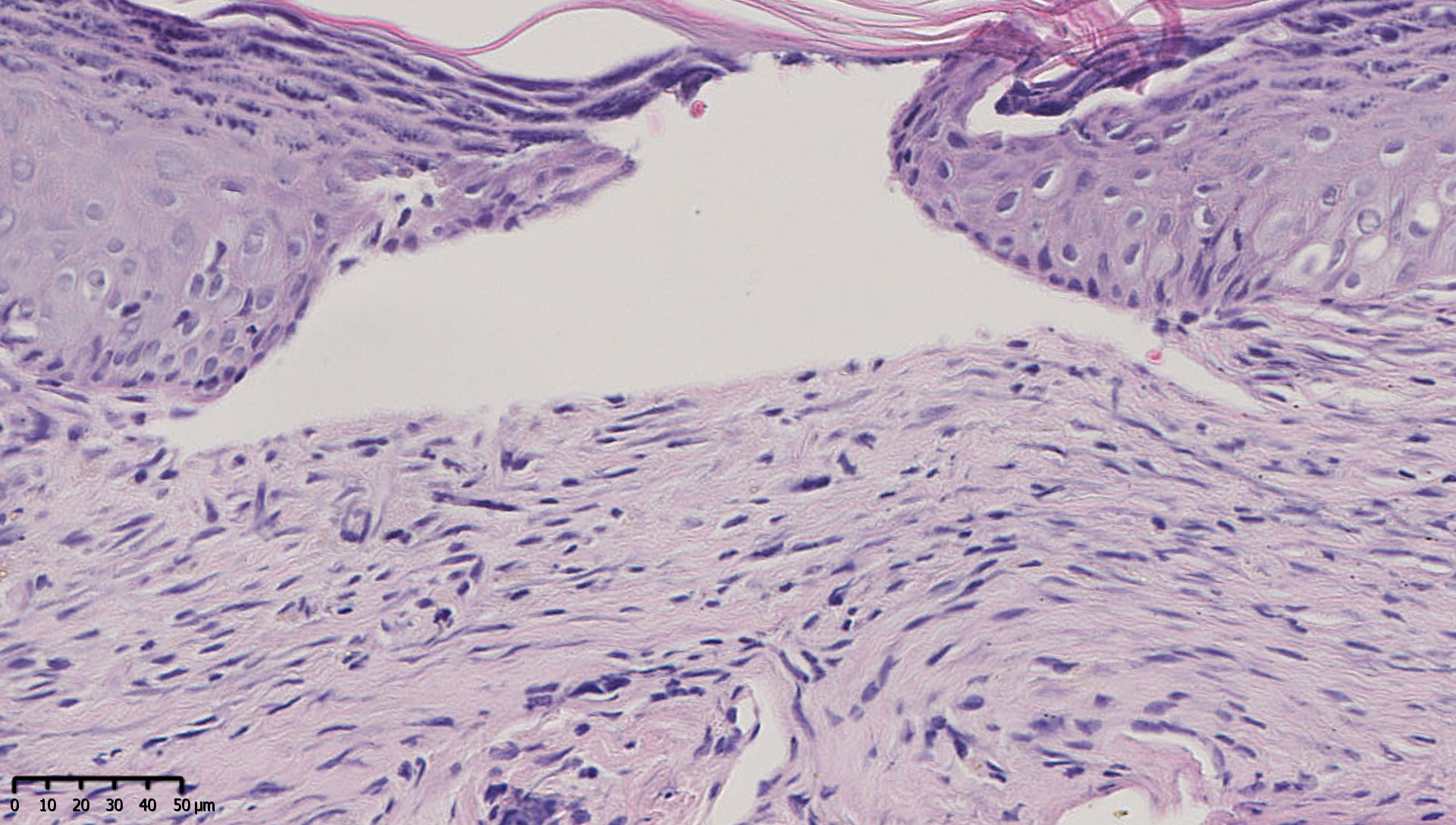

Supplement: S1 File — (ZIP) [file pone.0324264.s001.zip › supplement.material-1/HE triple section image/model-2 400x.jpg]

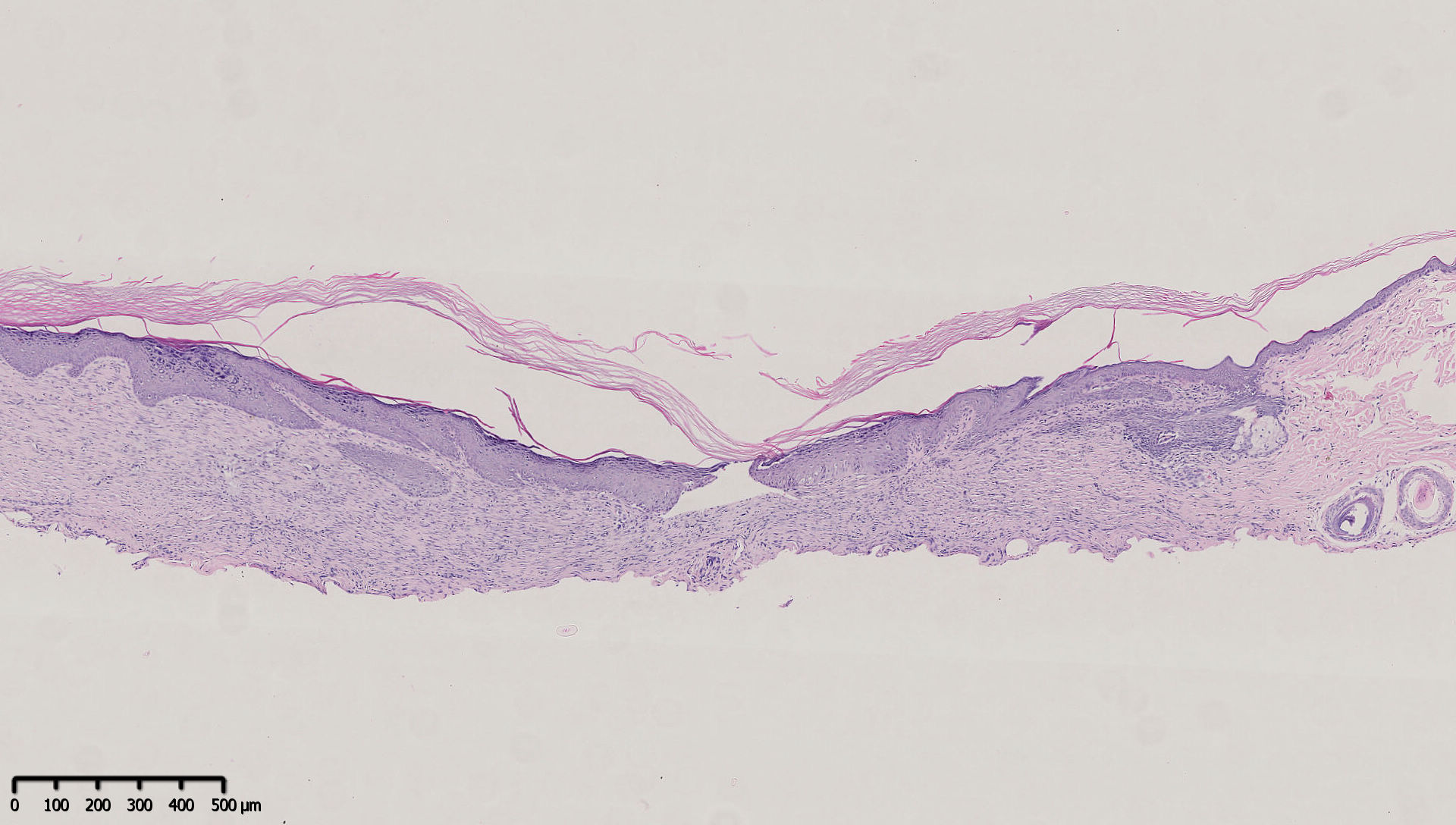

Supplement: S1 File — (ZIP) [file pone.0324264.s001.zip › supplement.material-1/HE triple section image/model-2 50x.jpg]

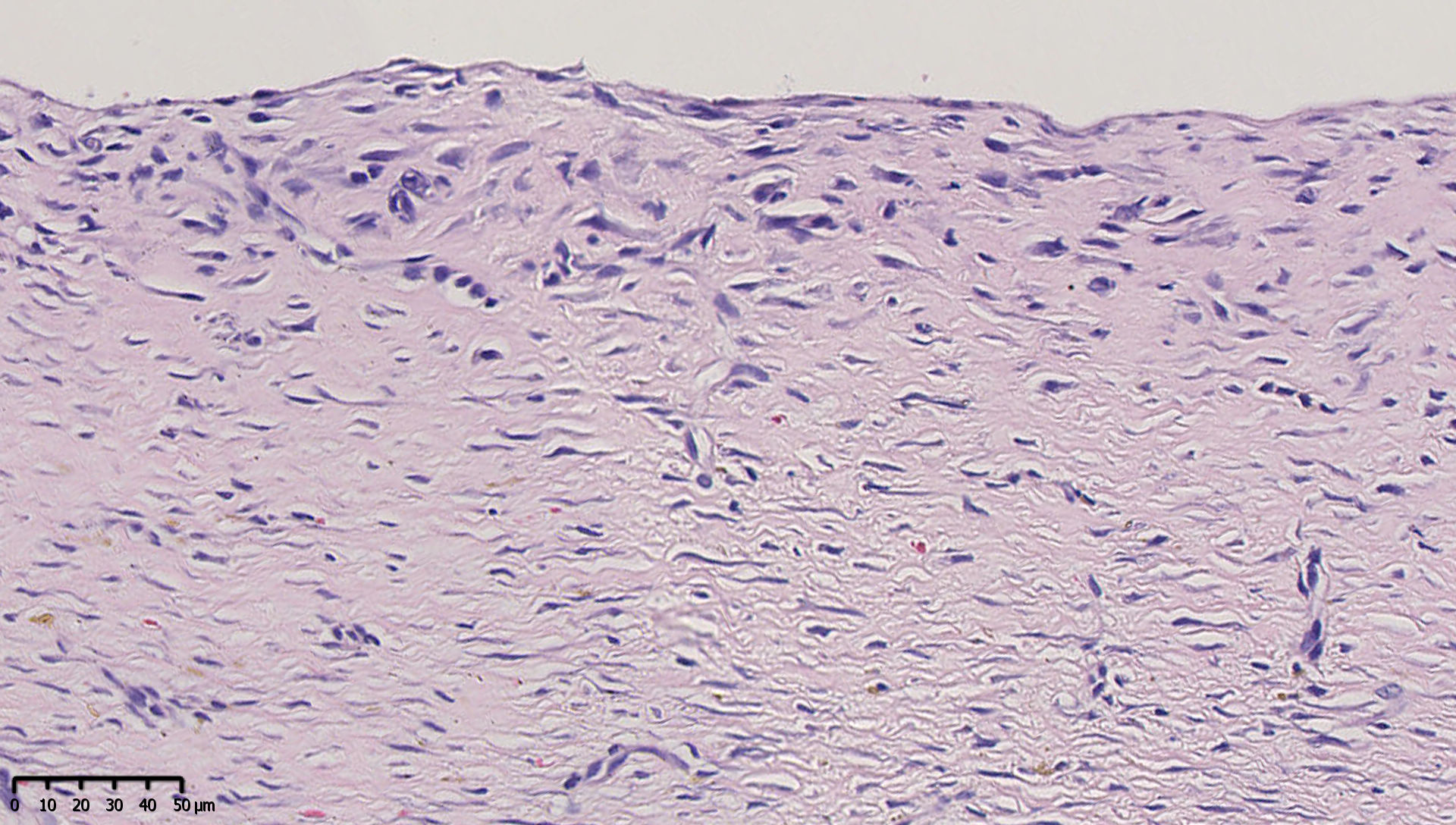

Supplement: S1 File — (ZIP) [file pone.0324264.s001.zip › supplement.material-1/HE triple section image/model-3 400x.jpg]

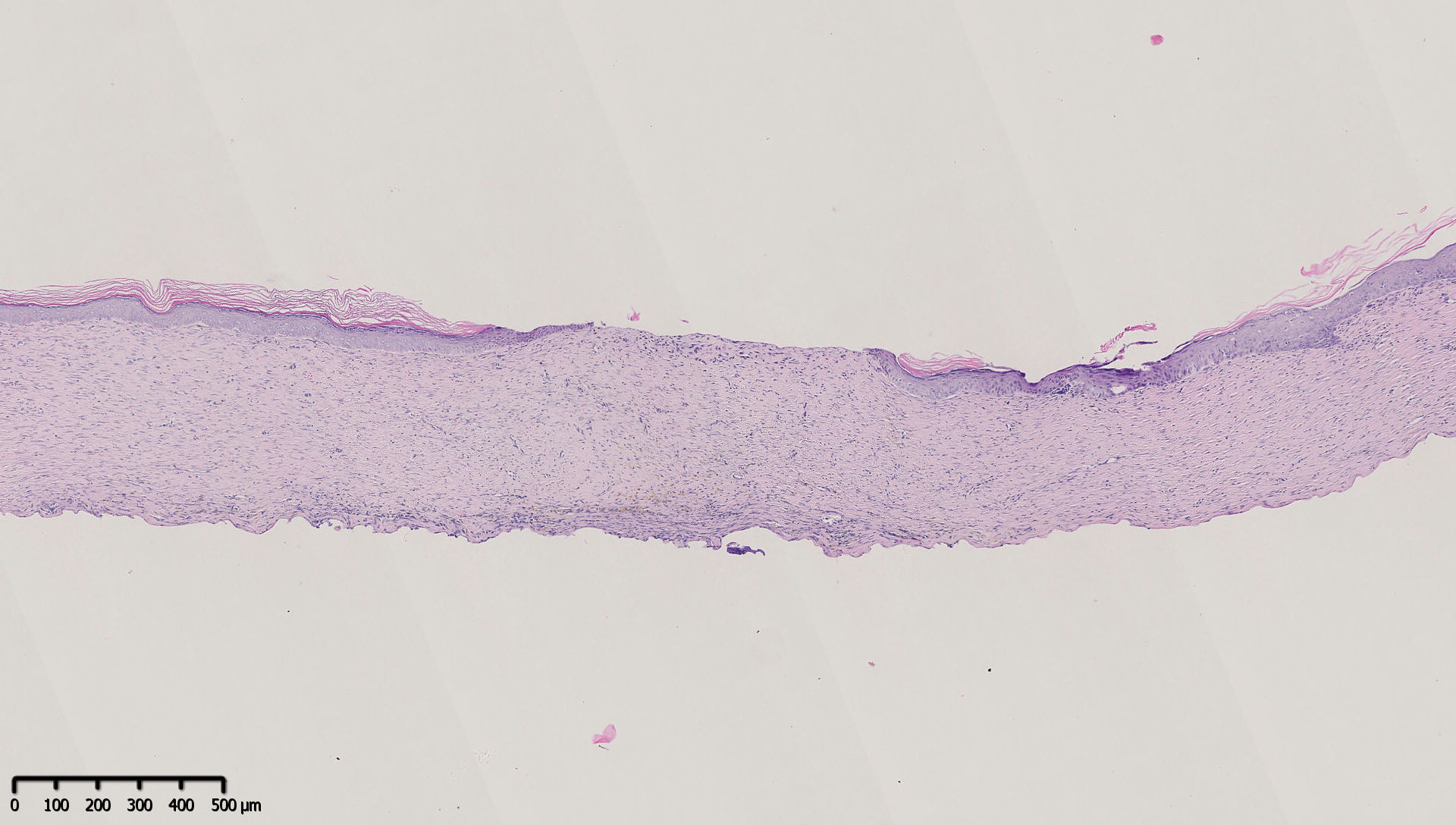

Supplement: S1 File — (ZIP) [file pone.0324264.s001.zip › supplement.material-1/HE triple section image/model-3 50x.jpg]

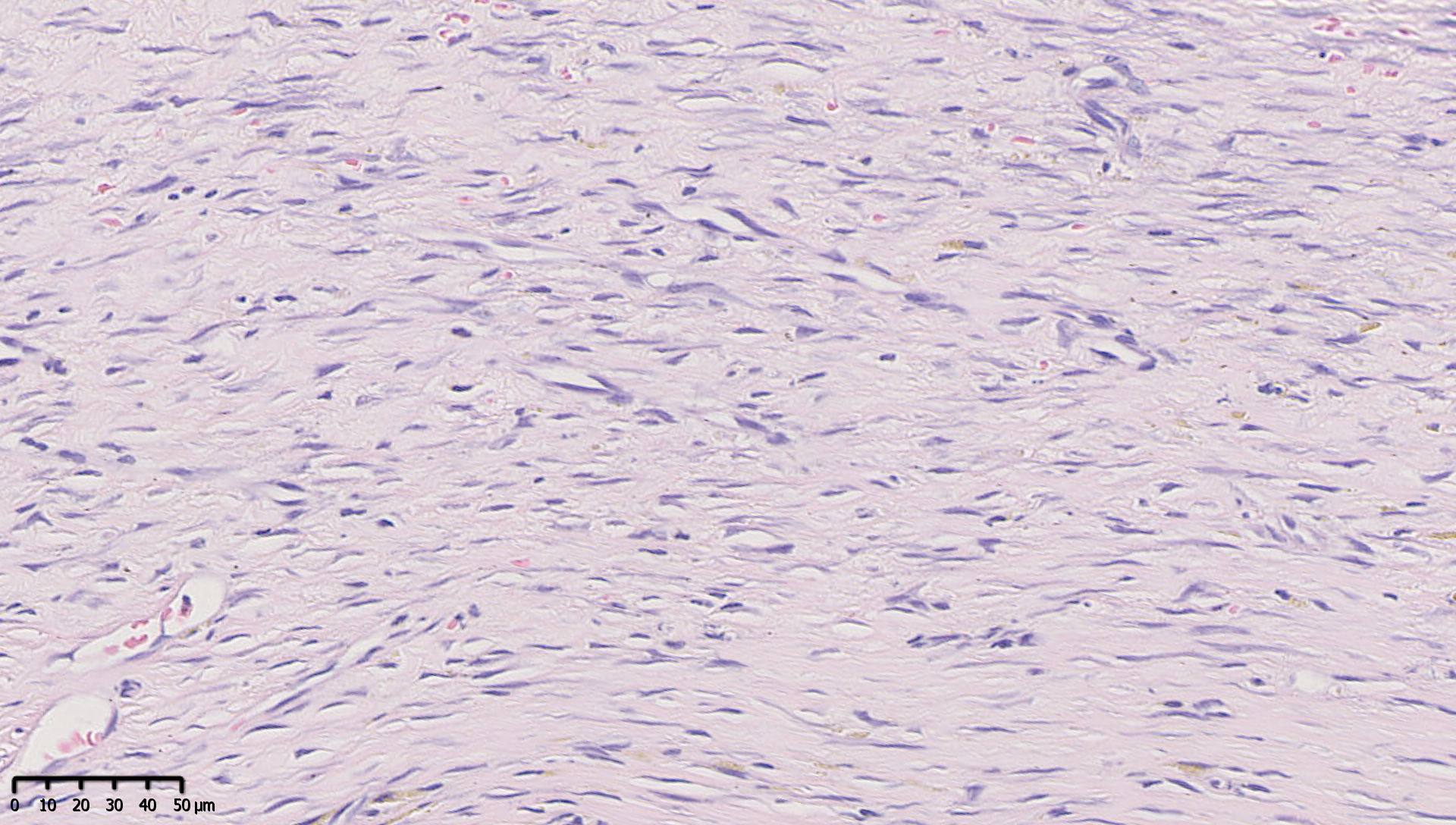

Supplement: S1 File — (ZIP) [file pone.0324264.s001.zip › supplement.material-1/HE triple section image/pl-ha-1 400x.jpg]

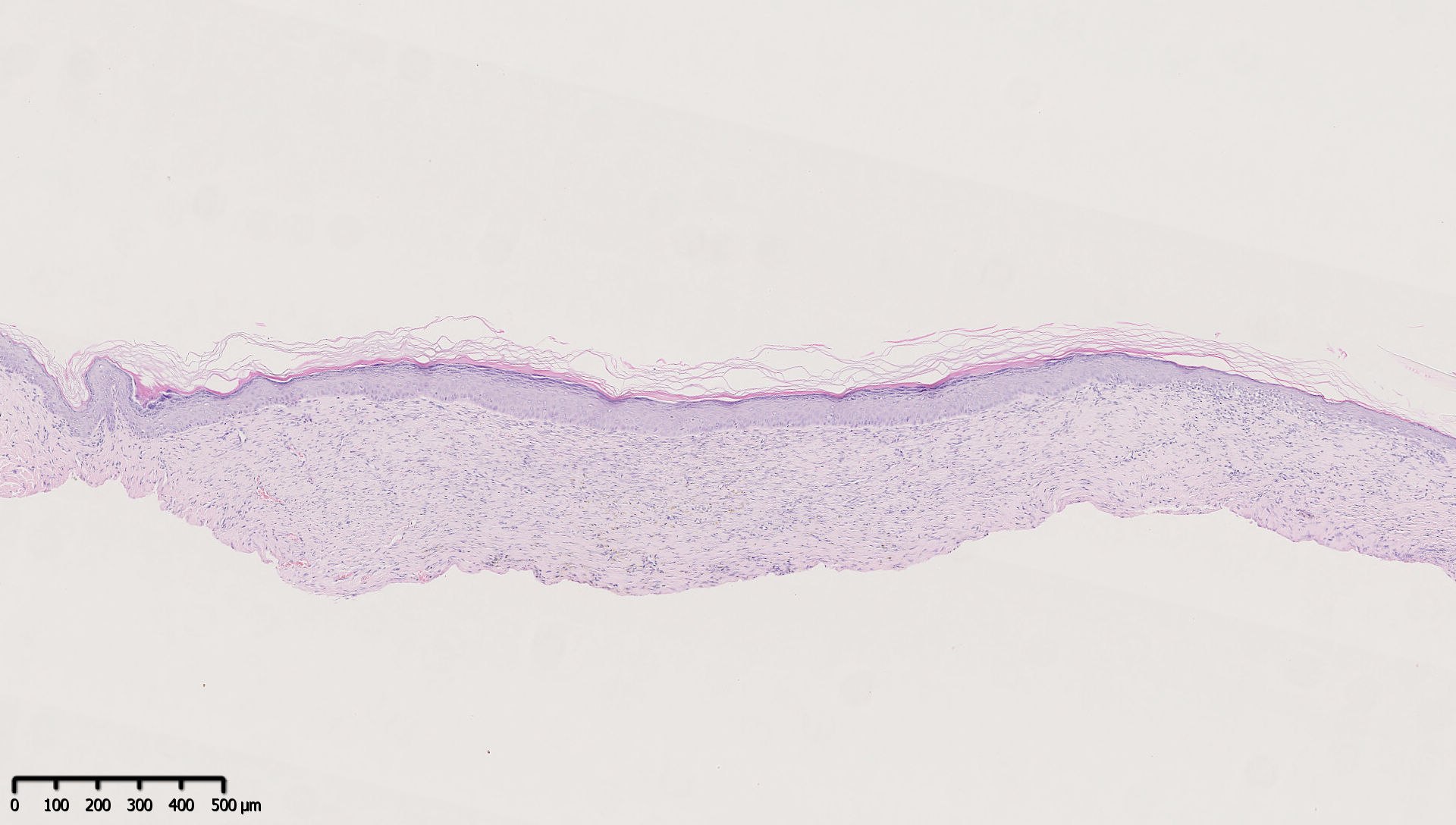

Supplement: S1 File — (ZIP) [file pone.0324264.s001.zip › supplement.material-1/HE triple section image/pl-ha-1 50x.jpg]

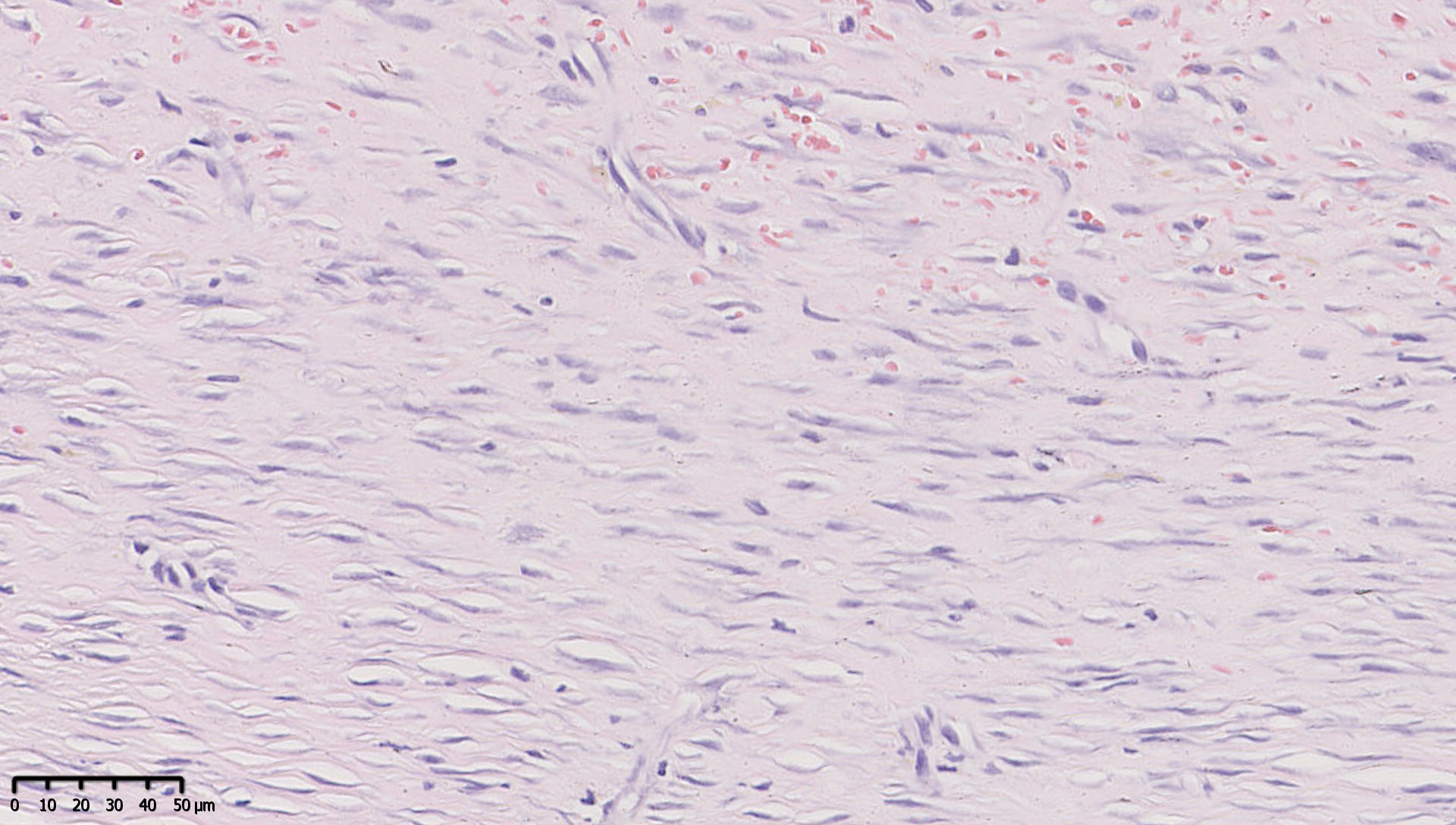

Supplement: S1 File — (ZIP) [file pone.0324264.s001.zip › supplement.material-1/HE triple section image/pl-ha-2 400x.jpg]

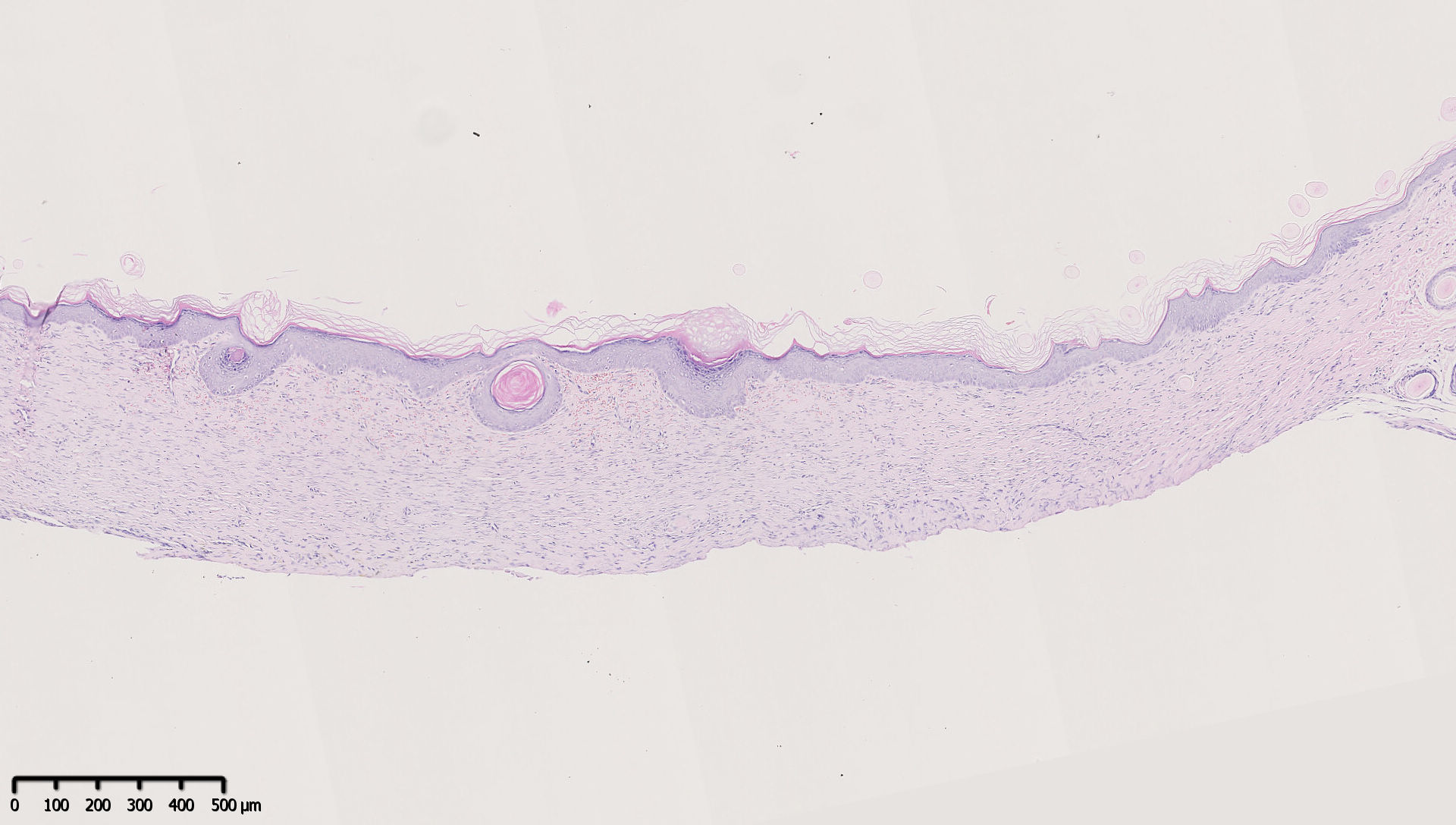

Supplement: S1 File — (ZIP) [file pone.0324264.s001.zip › supplement.material-1/HE triple section image/pl-ha-2 50x.jpg]

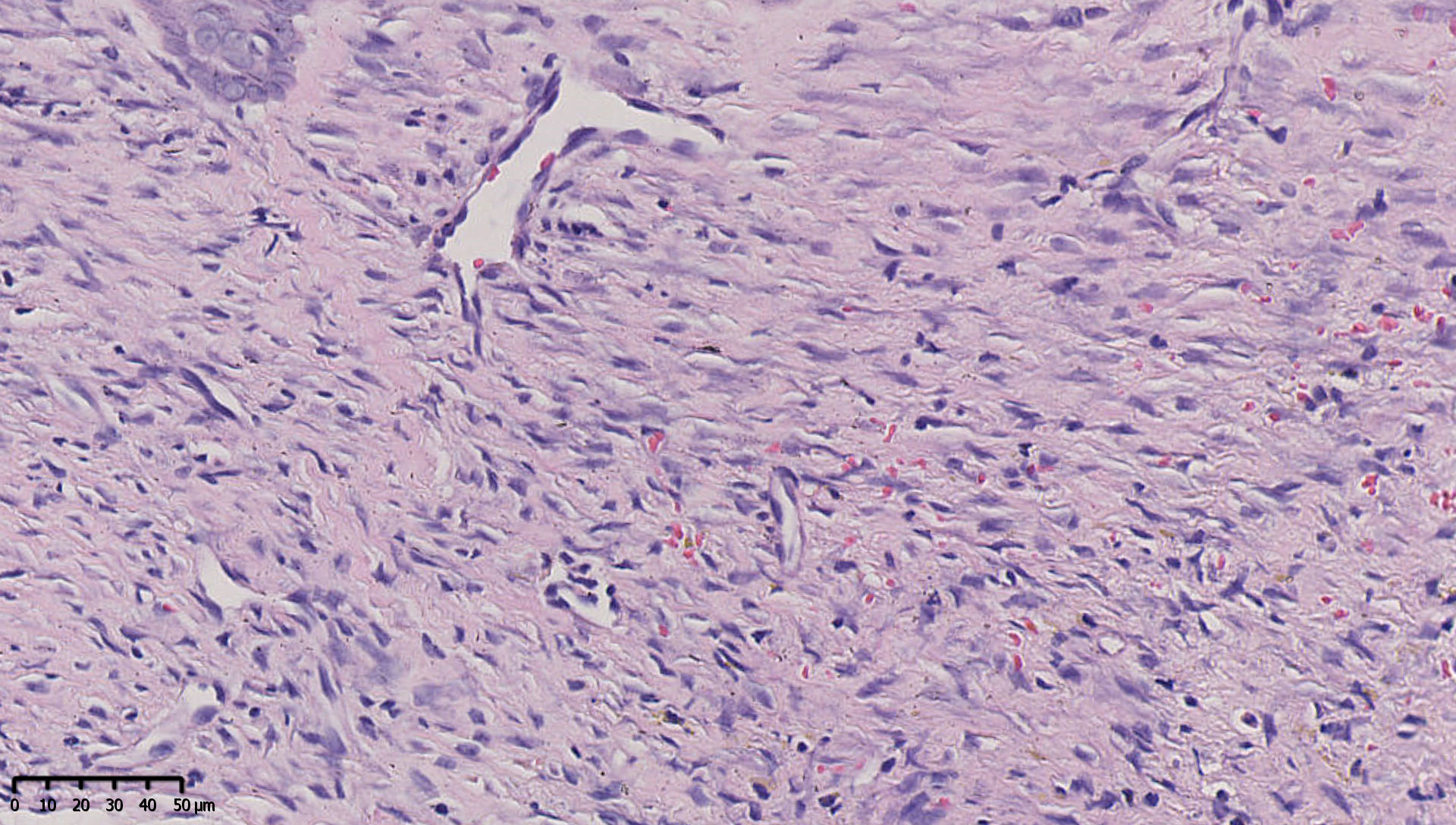

Supplement: S1 File — (ZIP) [file pone.0324264.s001.zip › supplement.material-1/HE triple section image/pl-ha-3 400x.jpg]

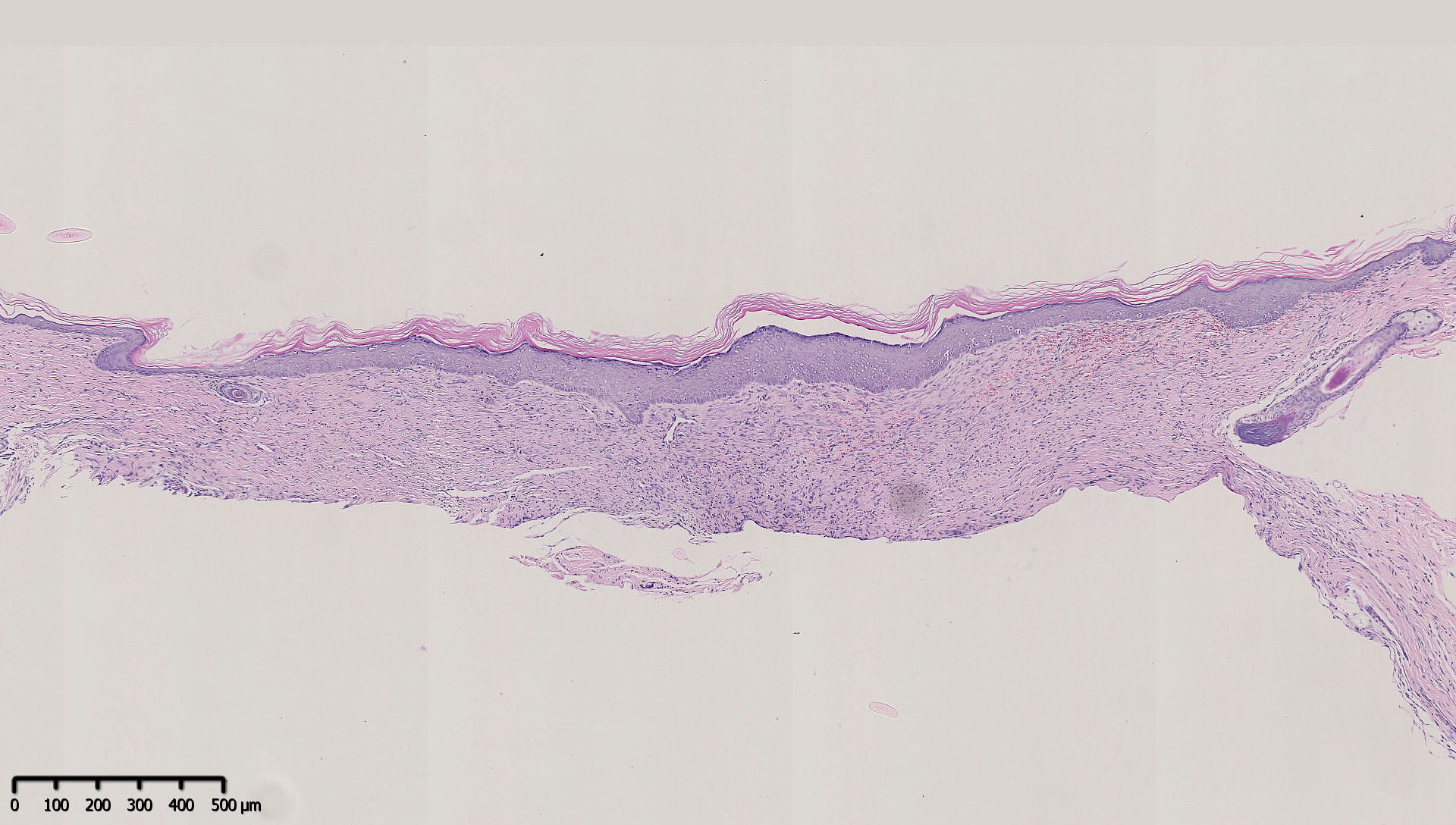

Supplement: S1 File — (ZIP) [file pone.0324264.s001.zip › supplement.material-1/HE triple section image/pl-ha-3 50x.jpg]

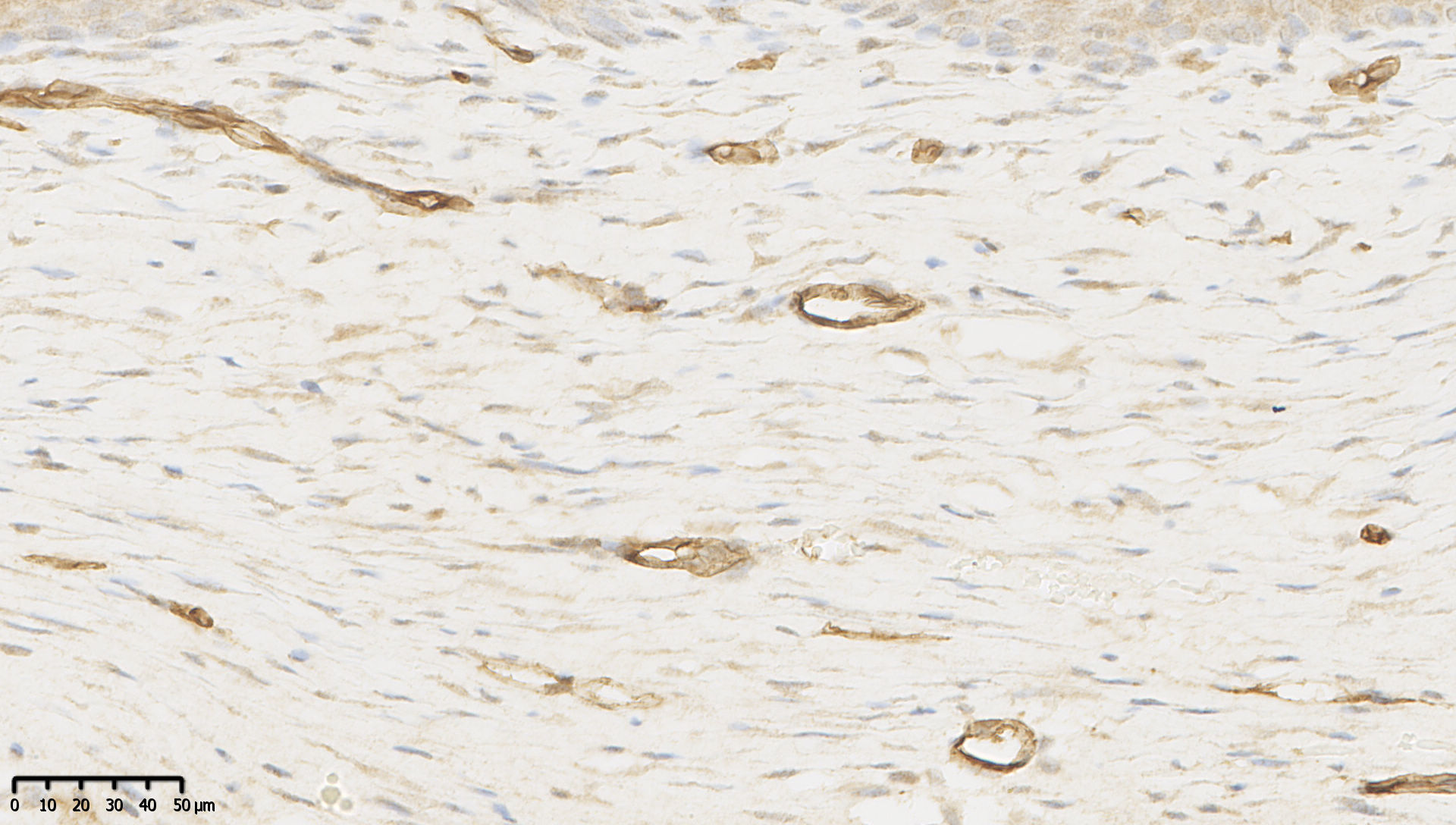

Supplement: S1 File — (ZIP) [file pone.0324264.s001.zip › supplement.material-1/Immunohistochemistry image/CD31/control-1.jpg]

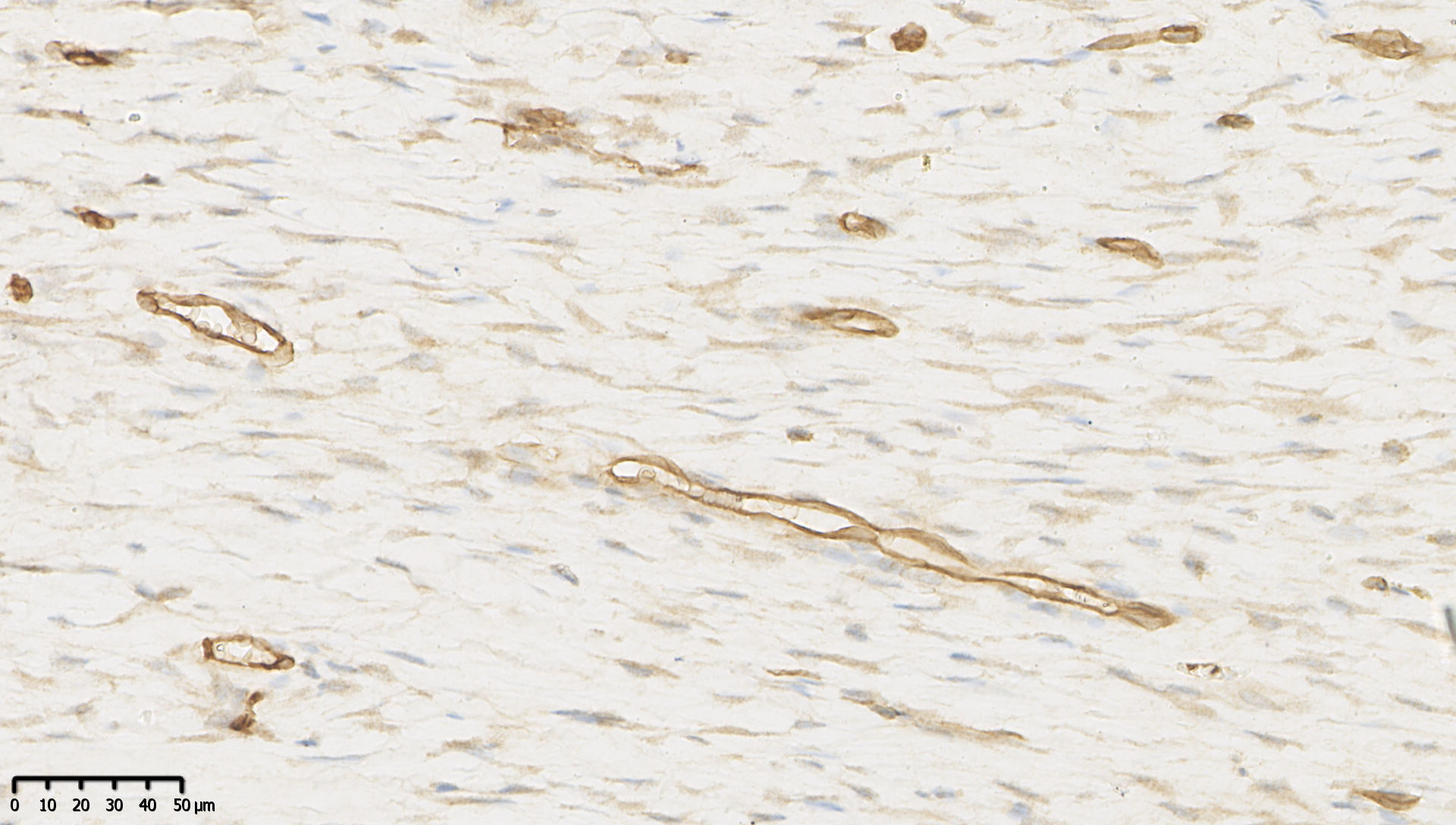

Supplement: S1 File — (ZIP) [file pone.0324264.s001.zip › supplement.material-1/Immunohistochemistry image/CD31/control-2.jpg]

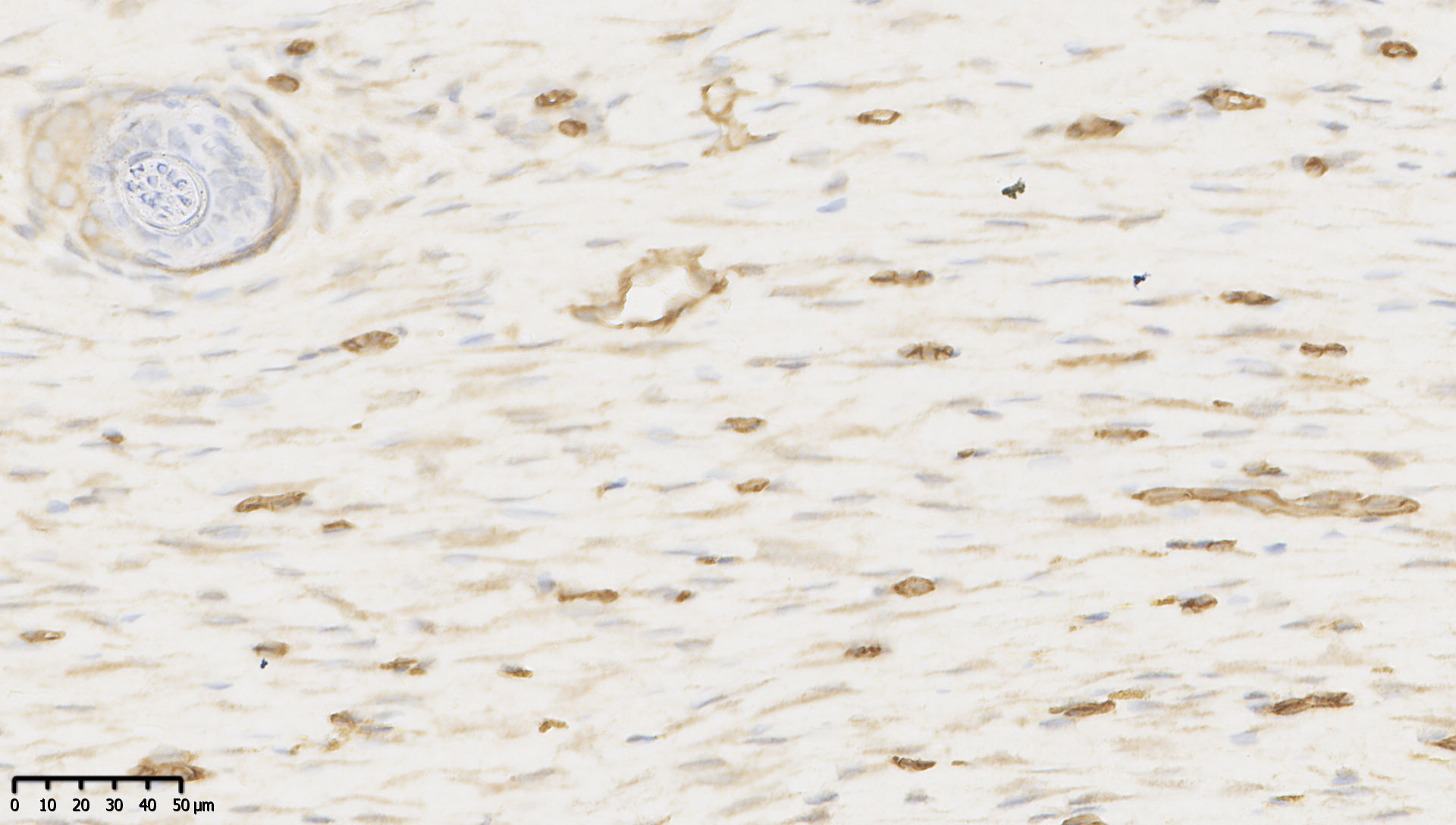

Supplement: S1 File — (ZIP) [file pone.0324264.s001.zip › supplement.material-1/Immunohistochemistry image/CD31/control-3.jpg]

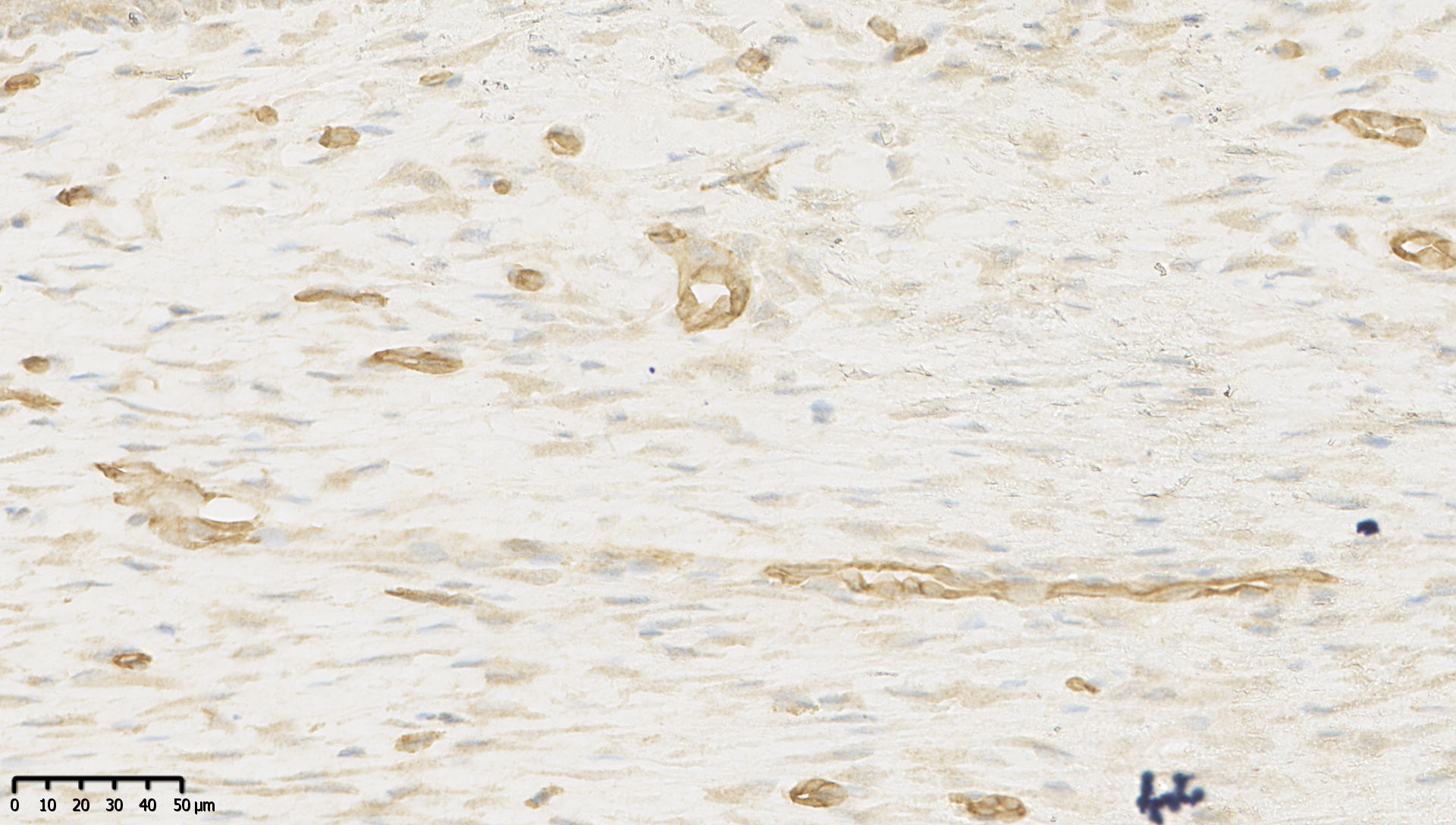

Supplement: S1 File — (ZIP) [file pone.0324264.s001.zip › supplement.material-1/Immunohistochemistry image/CD31/control-4.jpg]

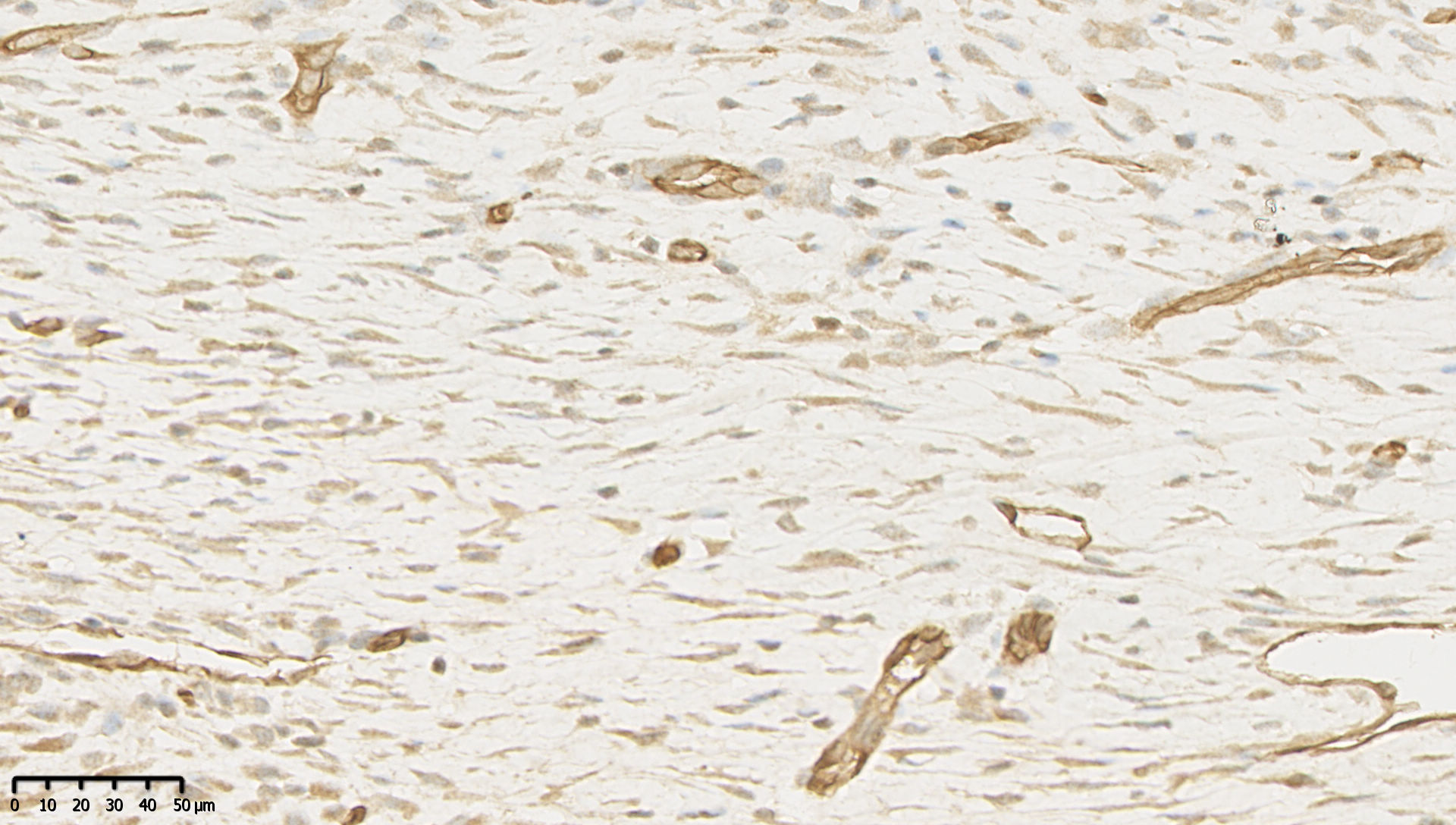

Supplement: S1 File — (ZIP) [file pone.0324264.s001.zip › supplement.material-1/Immunohistochemistry image/CD31/control-5.jpg]

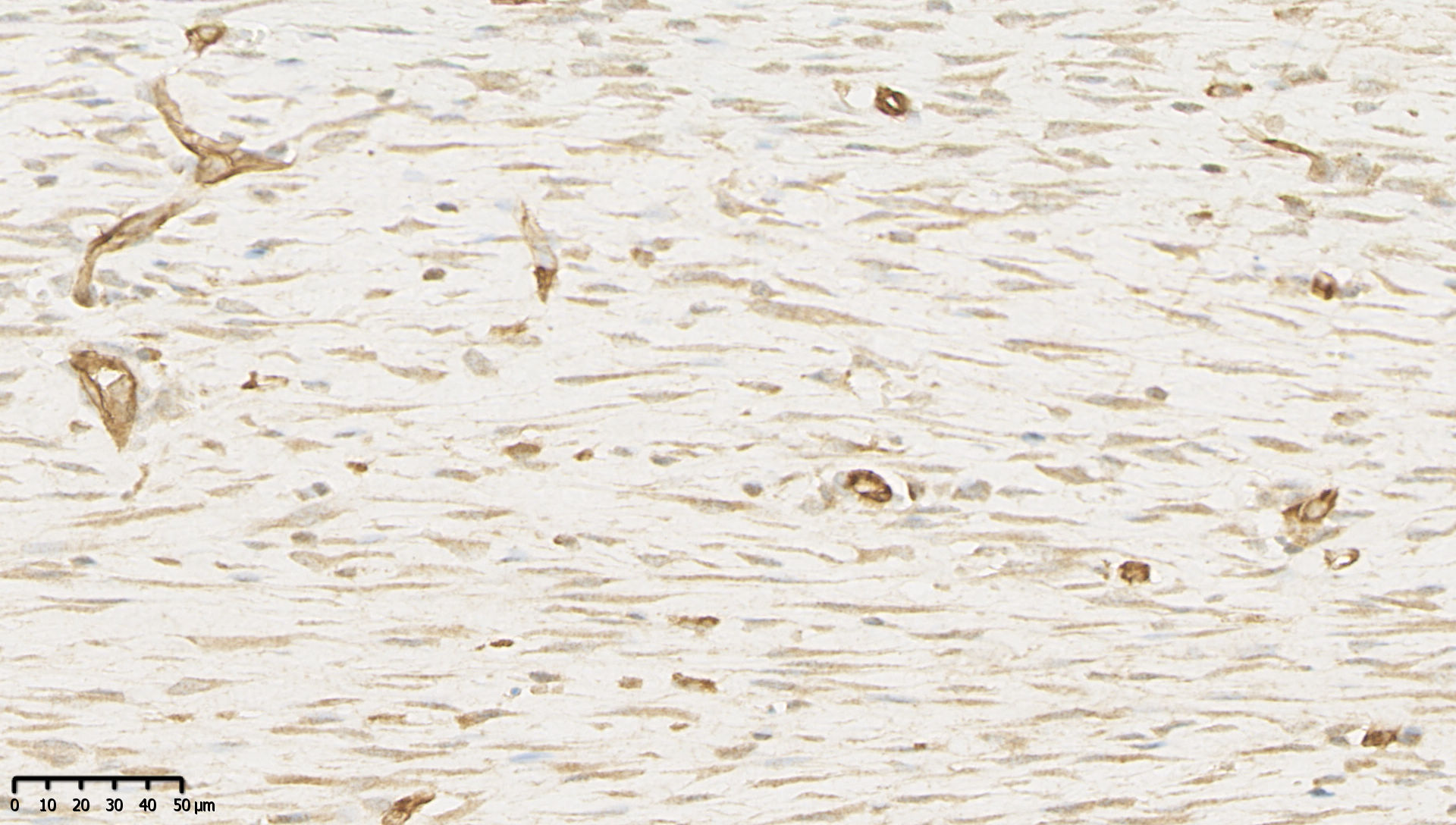

Supplement: S1 File — (ZIP) [file pone.0324264.s001.zip › supplement.material-1/Immunohistochemistry image/CD31/control-6.jpg]

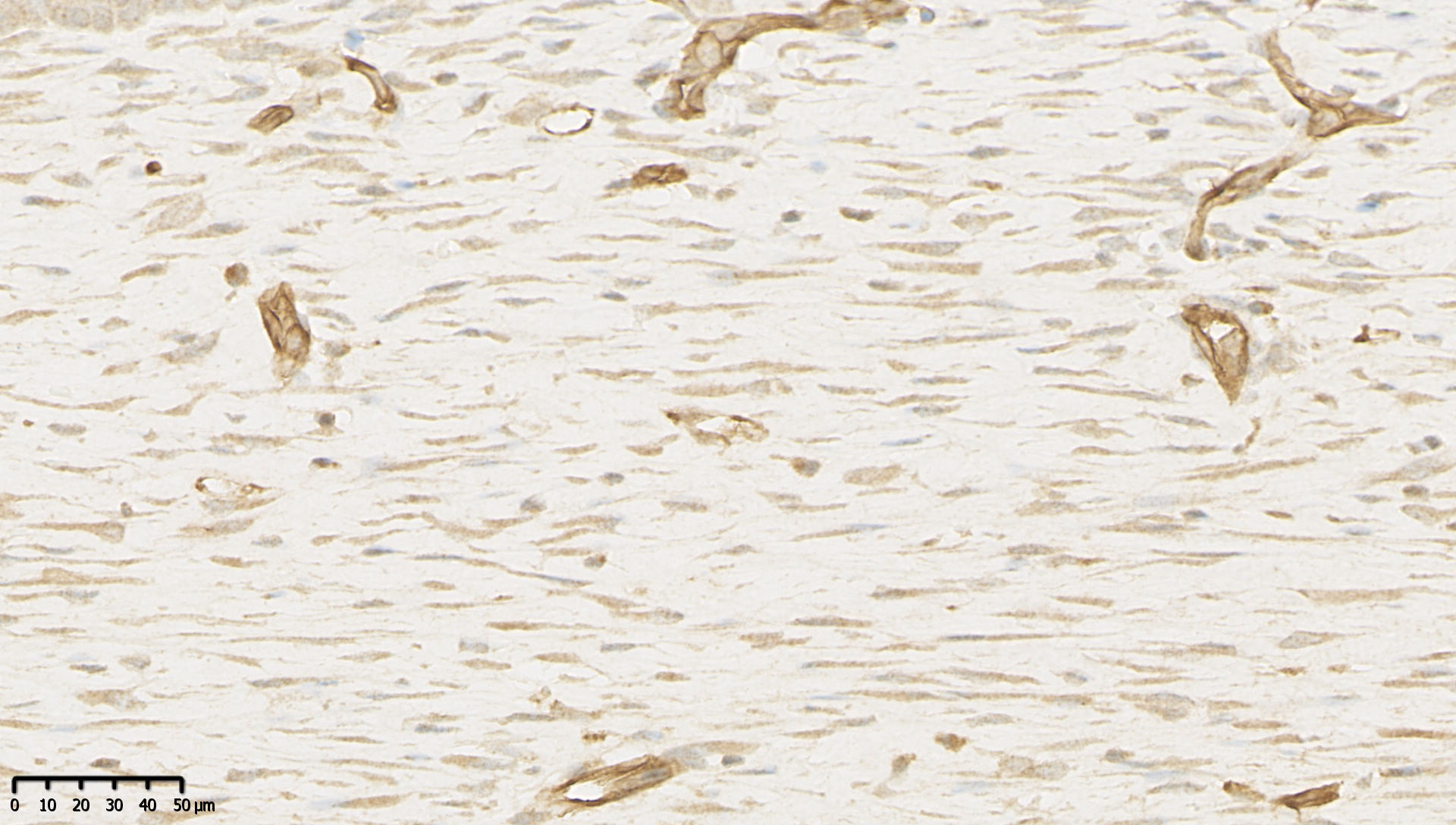

Supplement: S1 File — (ZIP) [file pone.0324264.s001.zip › supplement.material-1/Immunohistochemistry image/CD31/control-7.jpg]

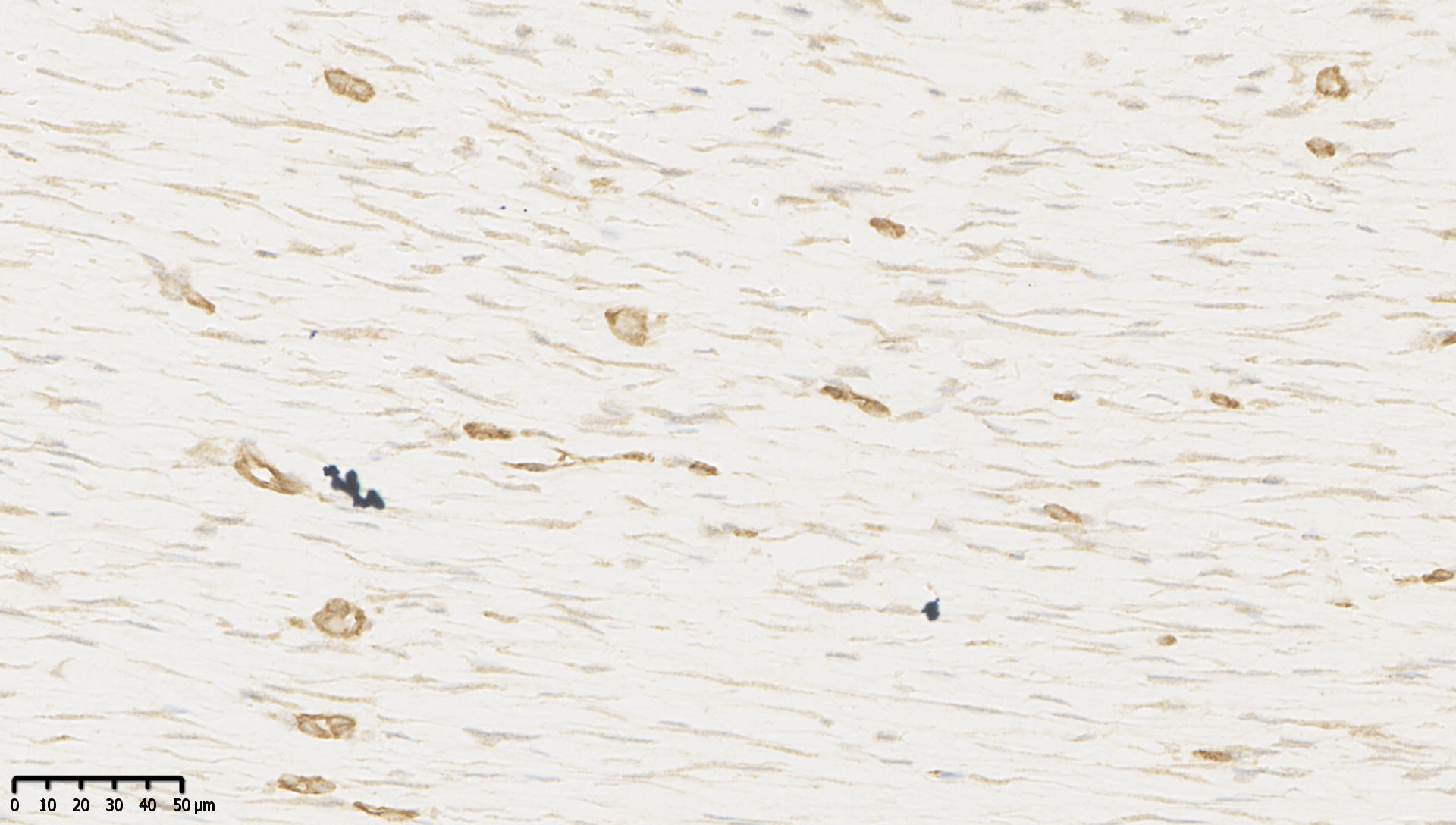

Supplement: S1 File — (ZIP) [file pone.0324264.s001.zip › supplement.material-1/Immunohistochemistry image/CD31/HA-1.jpg]

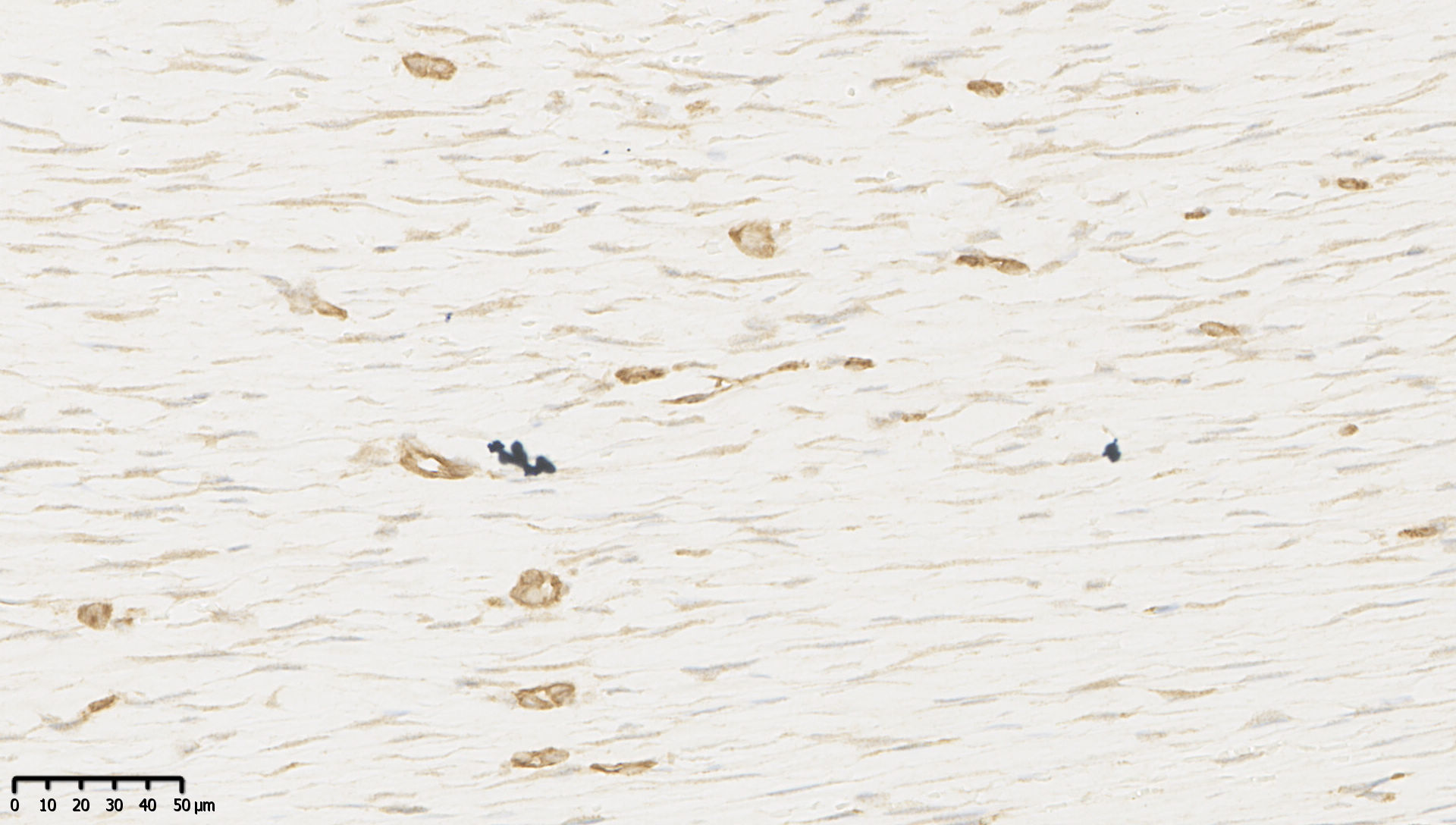

Supplement: S1 File — (ZIP) [file pone.0324264.s001.zip › supplement.material-1/Immunohistochemistry image/CD31/HA-2.jpg]

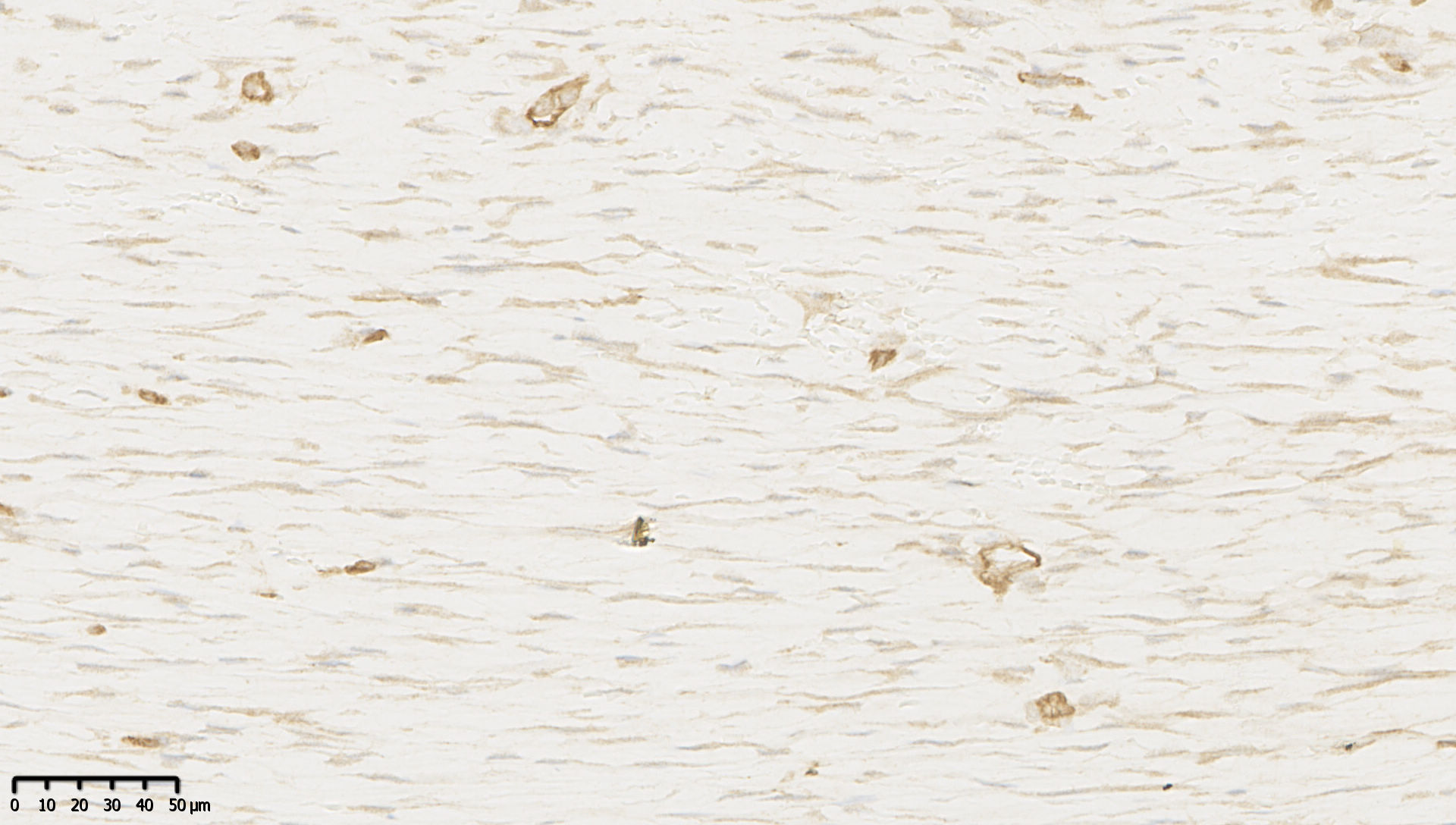

Supplement: S1 File — (ZIP) [file pone.0324264.s001.zip › supplement.material-1/Immunohistochemistry image/CD31/HA-3.jpg]

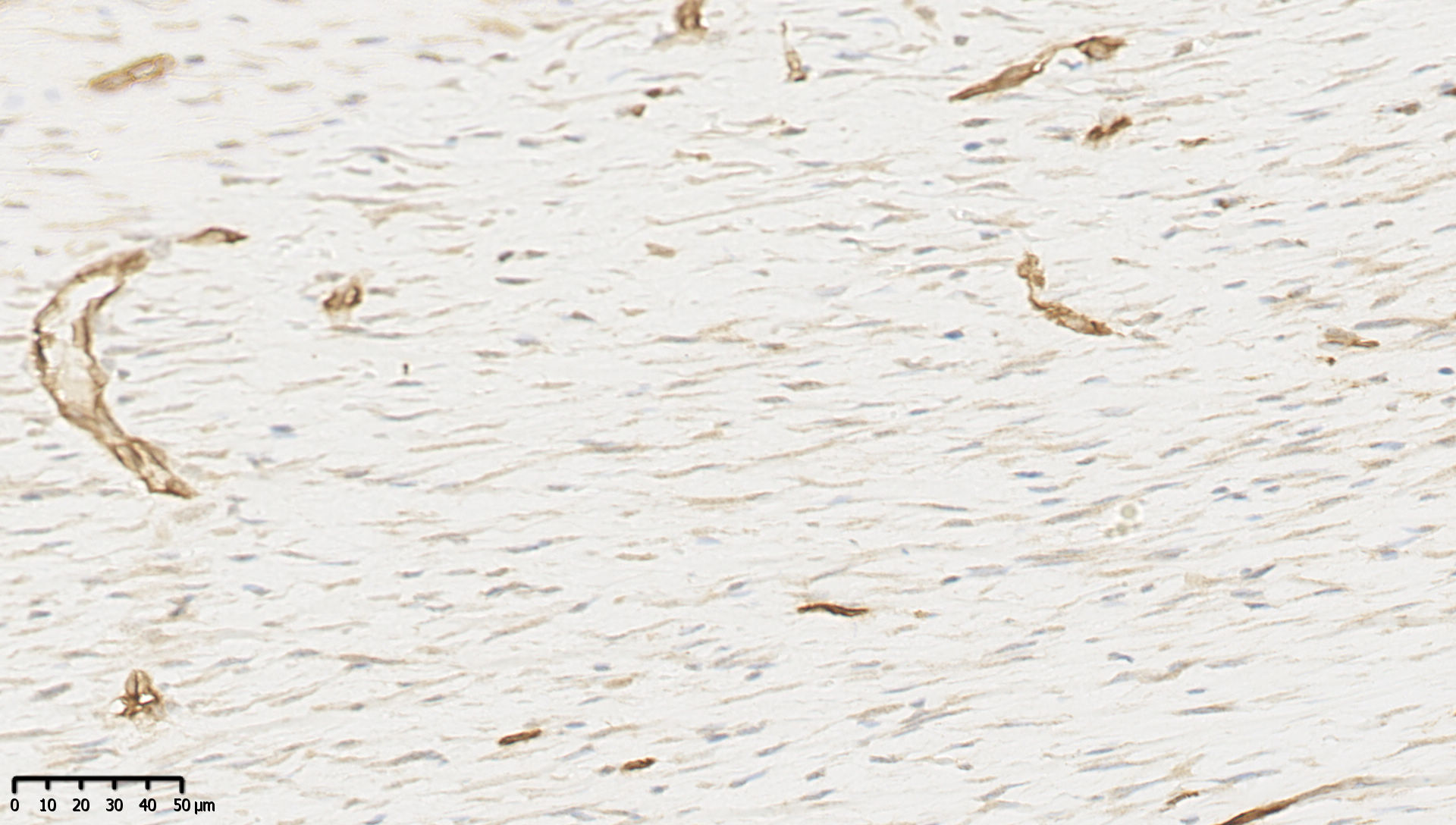

Supplement: S1 File — (ZIP) [file pone.0324264.s001.zip › supplement.material-1/Immunohistochemistry image/CD31/HA-4.jpg]

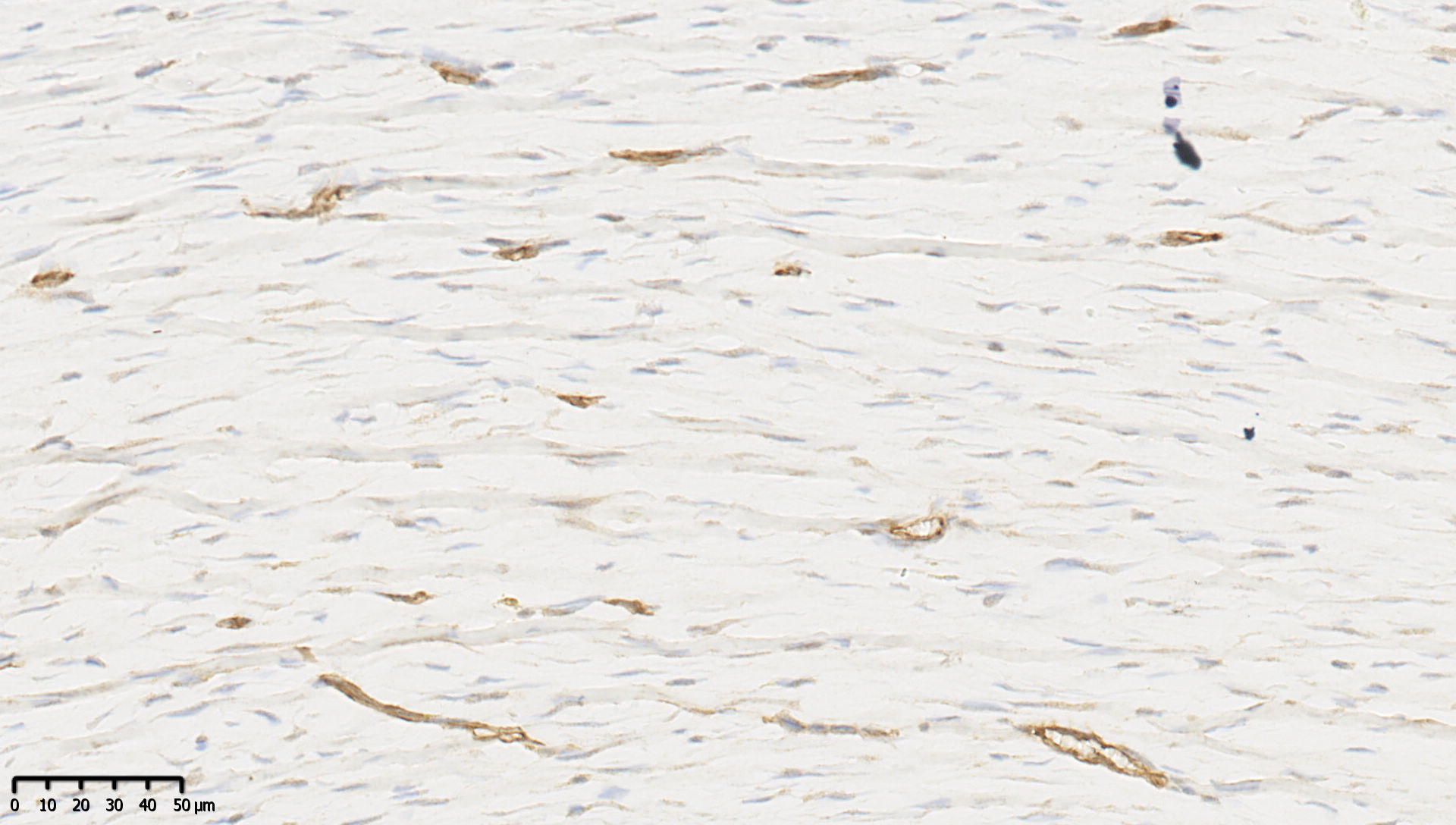

Supplement: S1 File — (ZIP) [file pone.0324264.s001.zip › supplement.material-1/Immunohistochemistry image/CD31/HA-5.jpg]

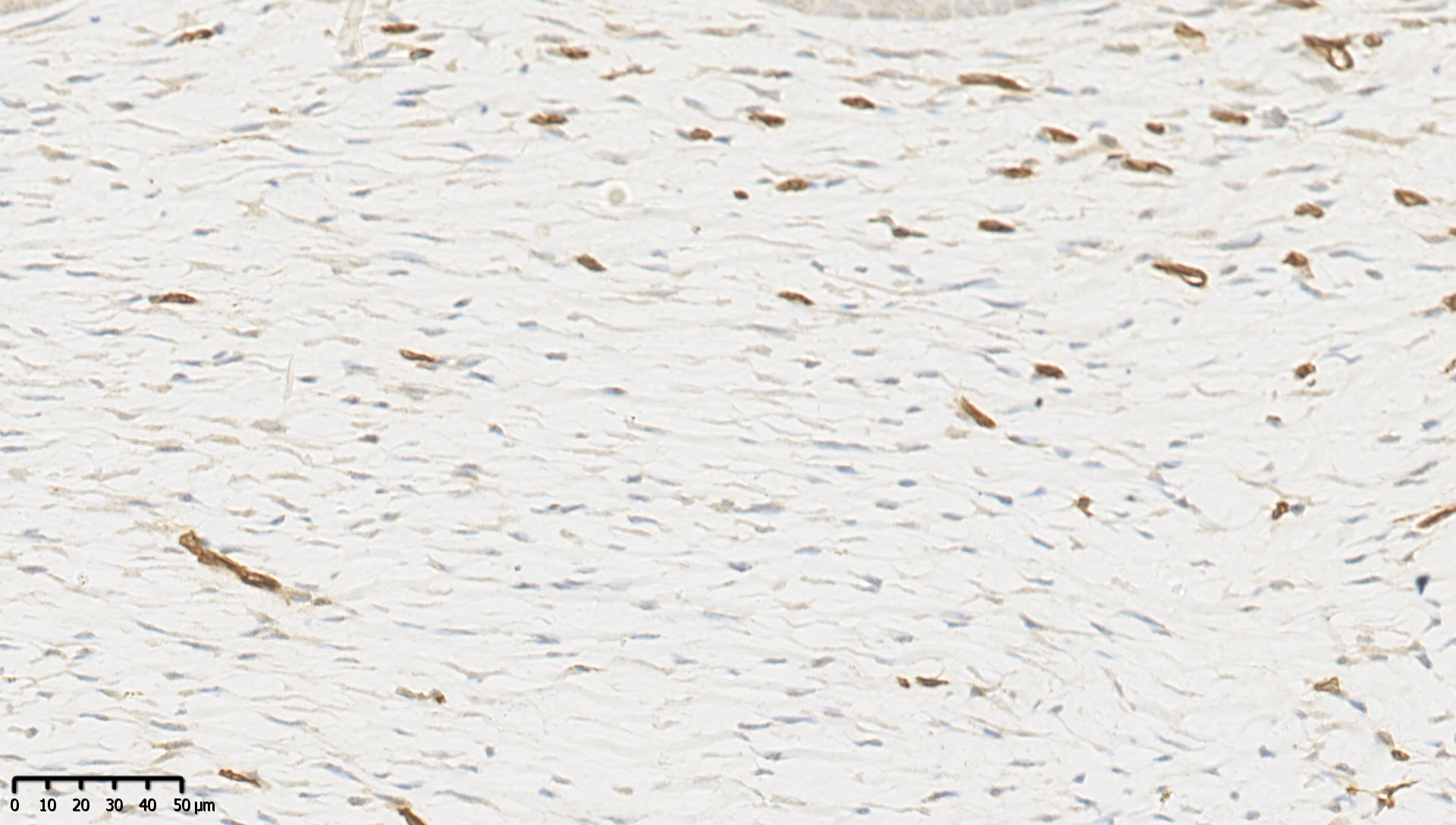

Supplement: S1 File — (ZIP) [file pone.0324264.s001.zip › supplement.material-1/Immunohistochemistry image/CD31/HA-6.jpg]

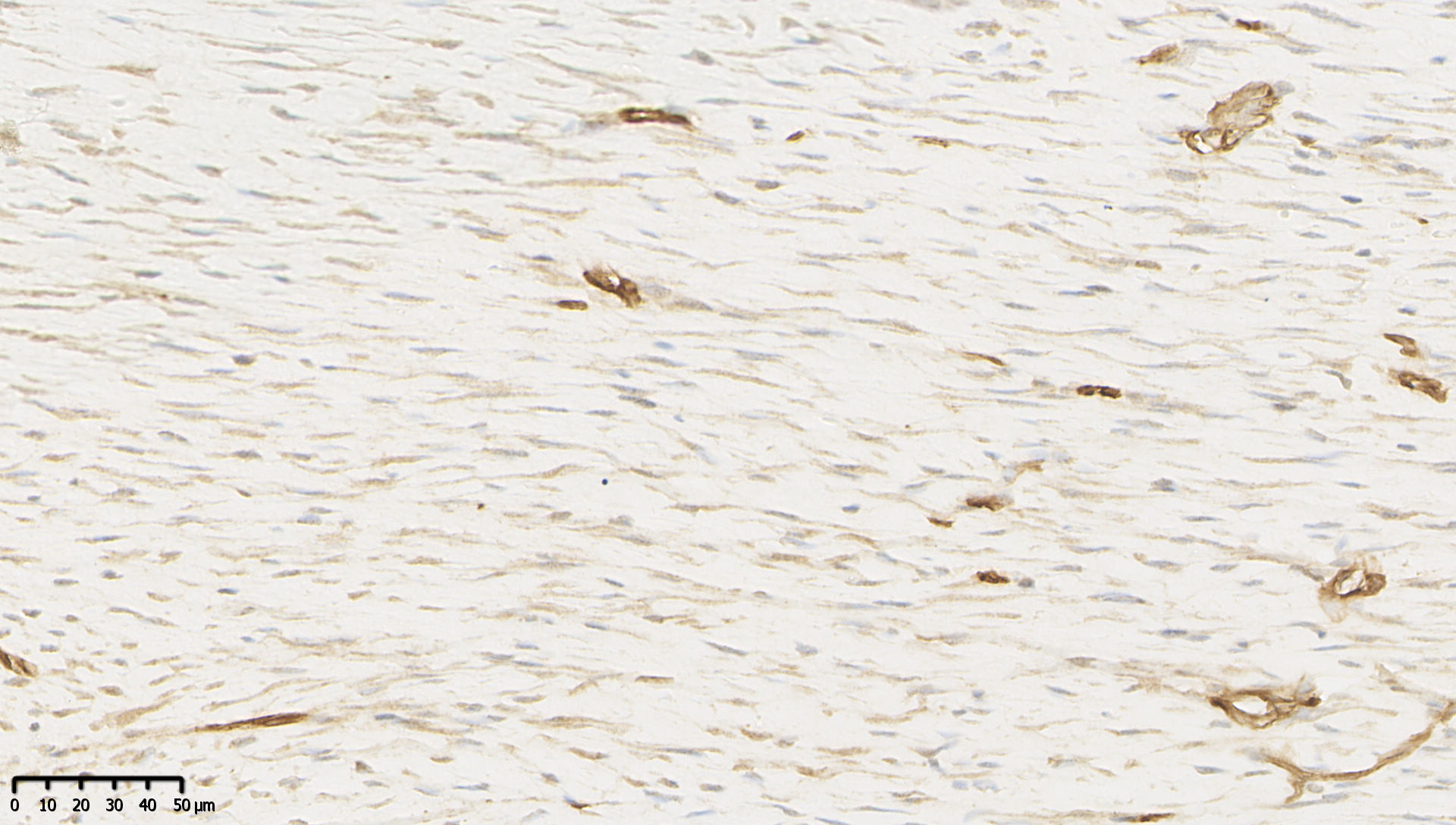

Supplement: S1 File — (ZIP) [file pone.0324264.s001.zip › supplement.material-1/Immunohistochemistry image/CD31/model-1.jpg]

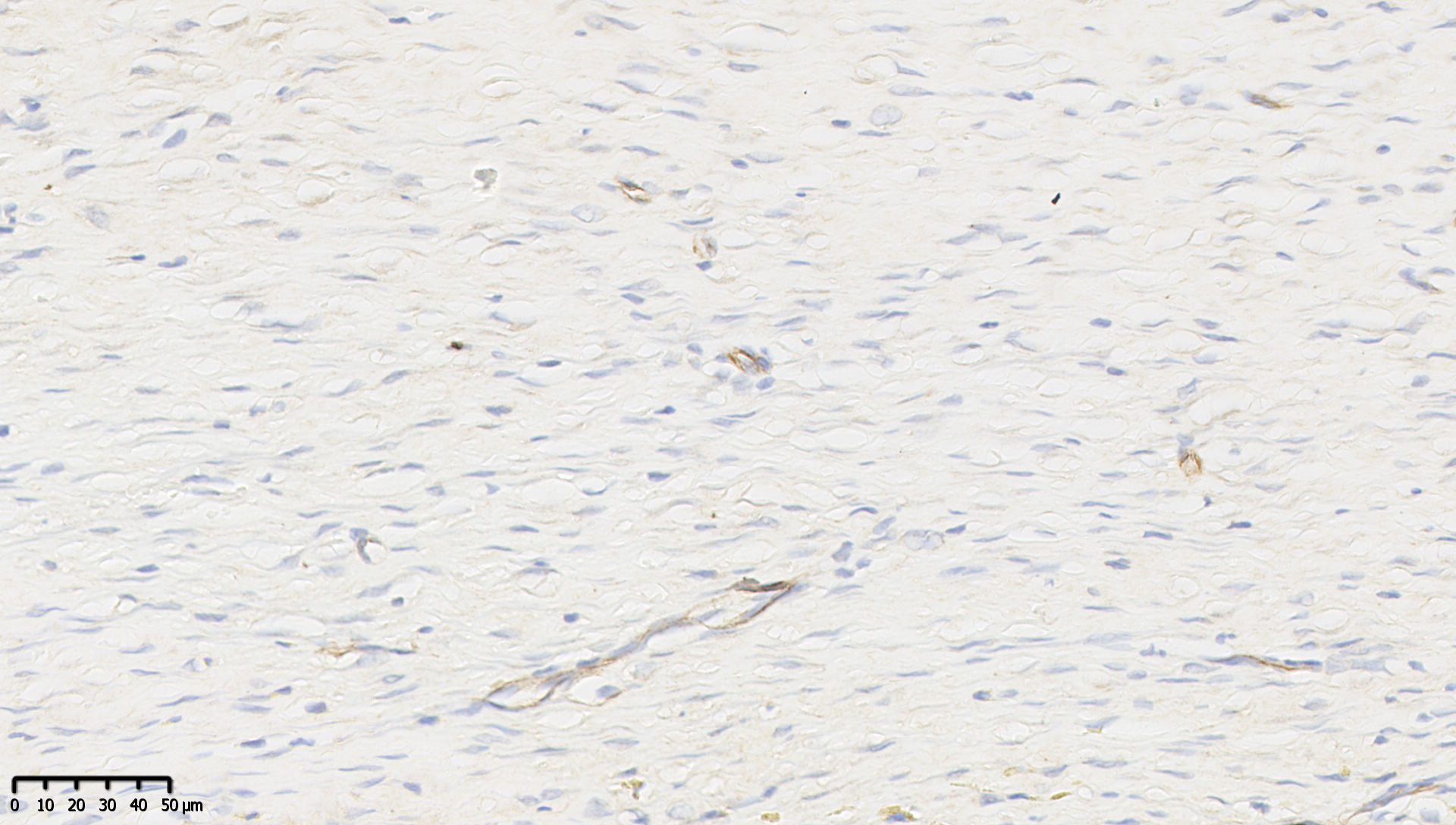

Supplement: S1 File — (ZIP) [file pone.0324264.s001.zip › supplement.material-1/Immunohistochemistry image/CD31/model-2.jpg]

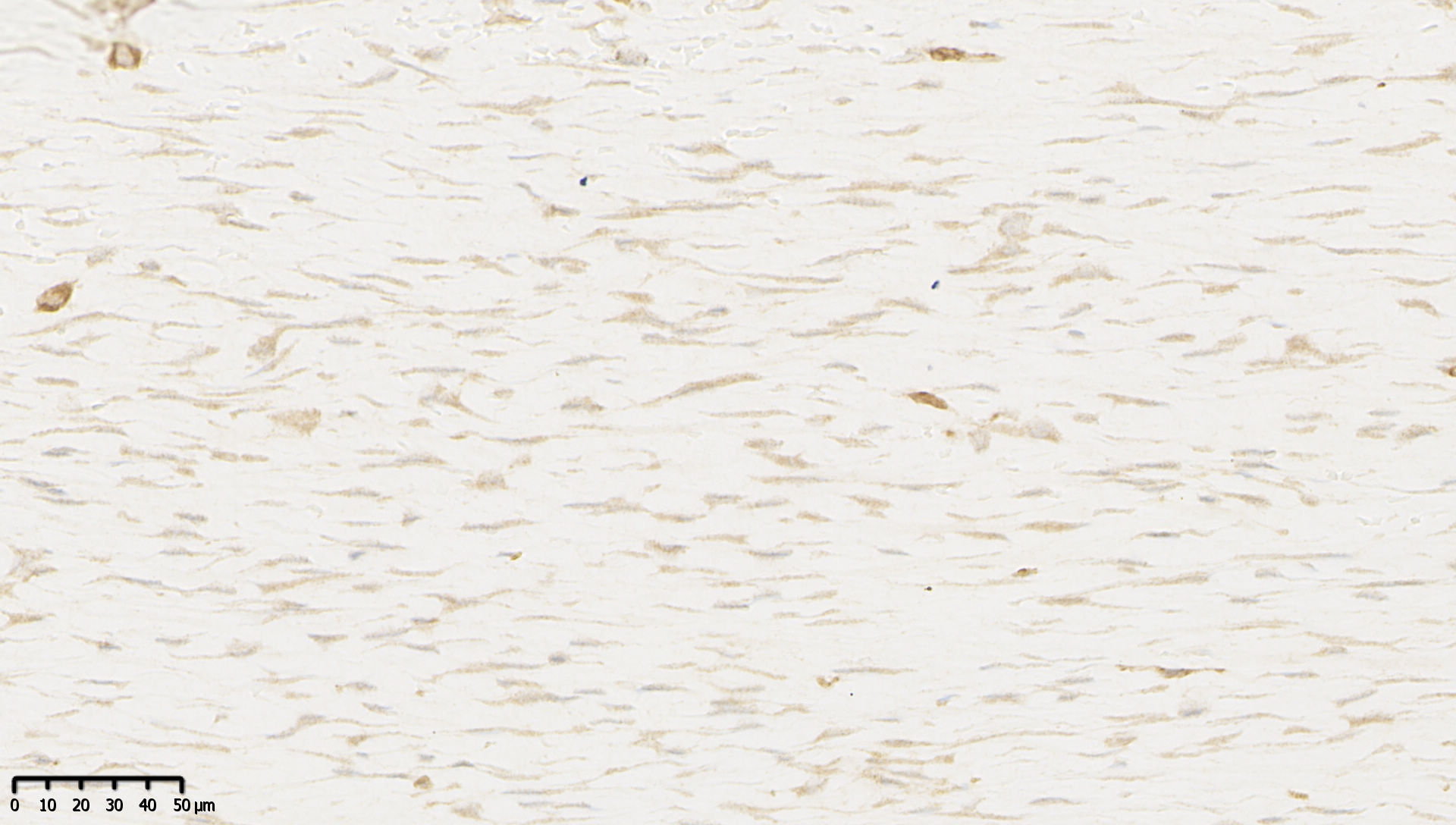

Supplement: S1 File — (ZIP) [file pone.0324264.s001.zip › supplement.material-1/Immunohistochemistry image/CD31/model-3.jpg]

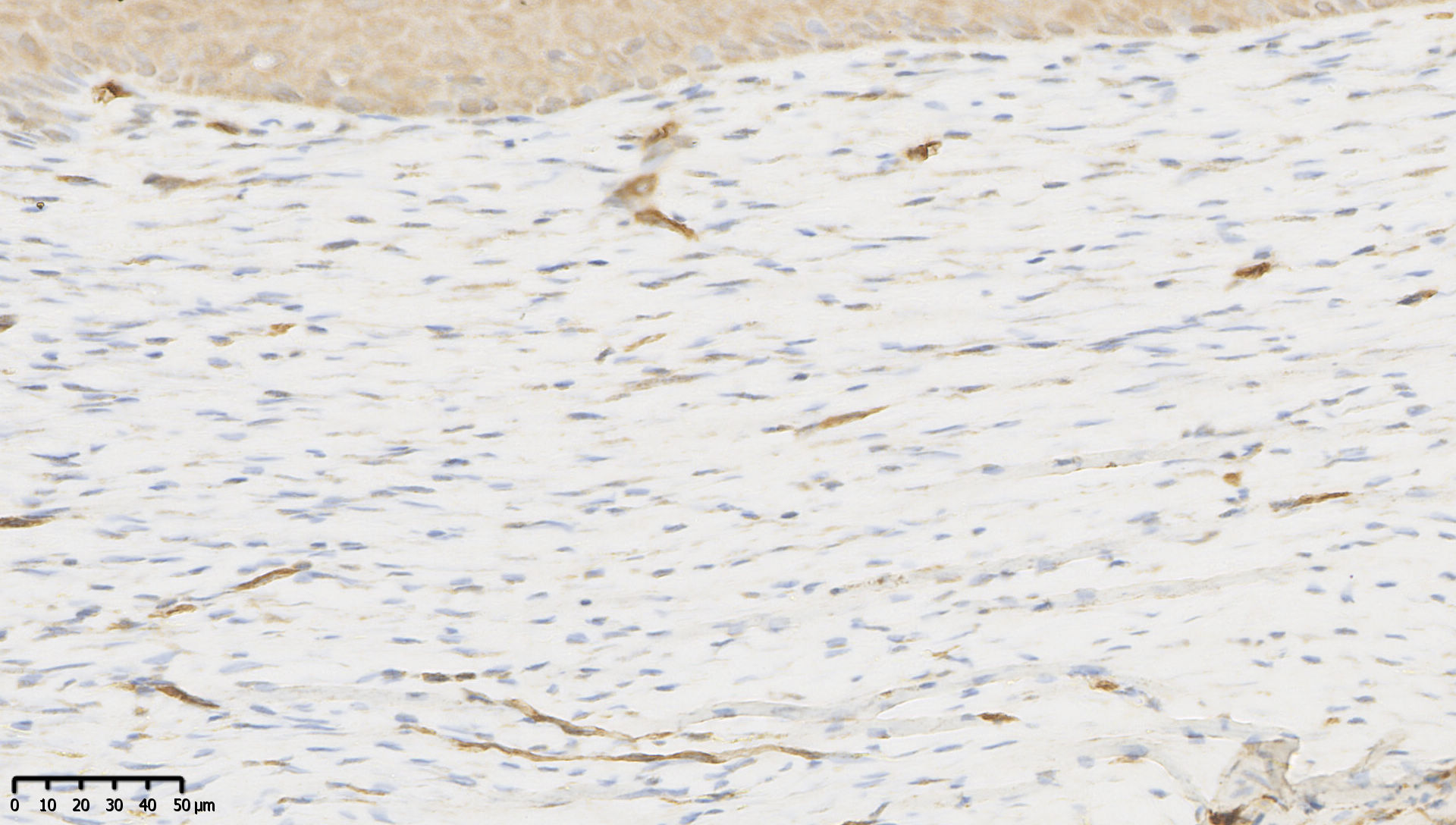

Supplement: S1 File — (ZIP) [file pone.0324264.s001.zip › supplement.material-1/Immunohistochemistry image/CD31/model-4.jpg]

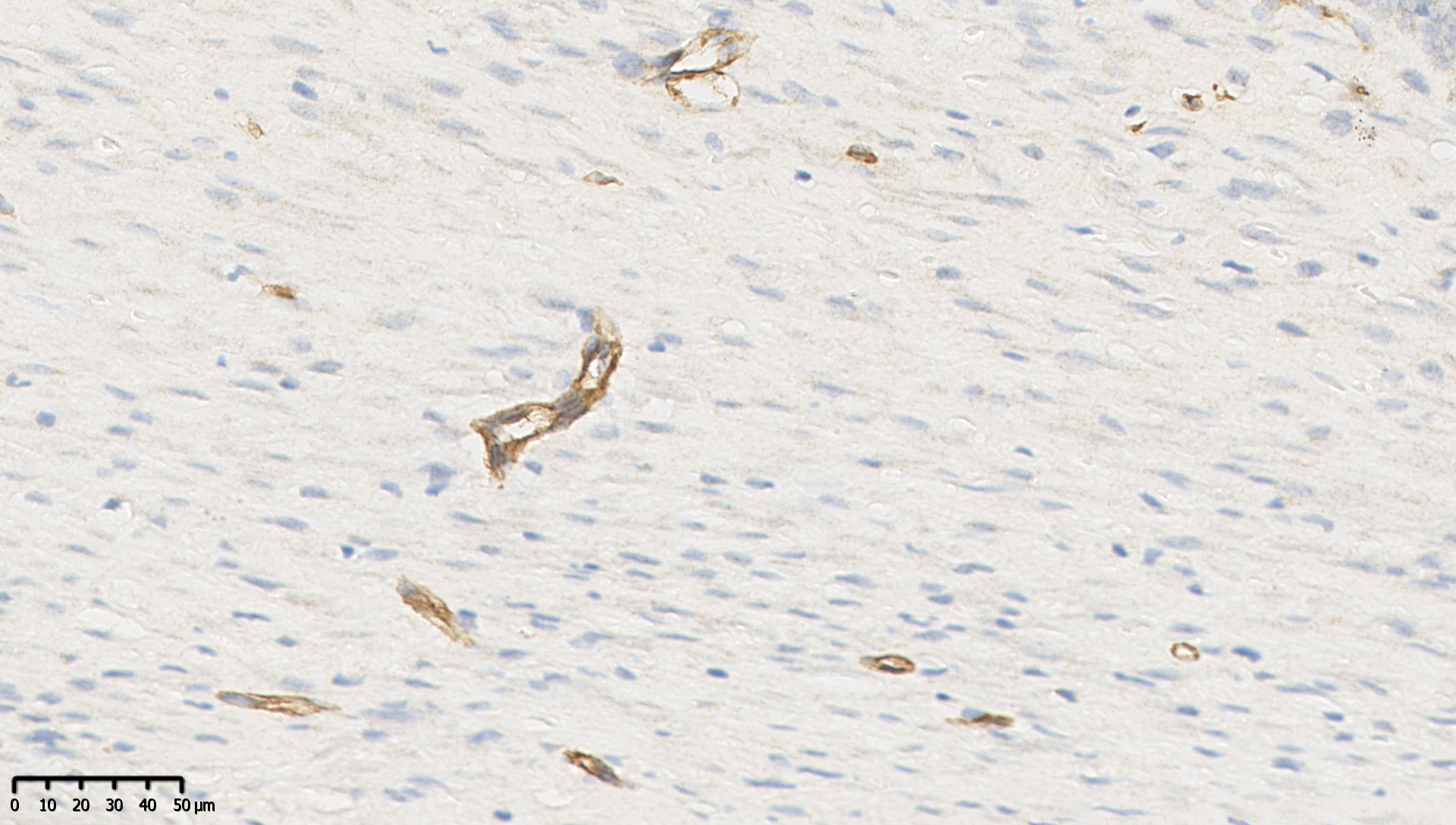

Supplement: S1 File — (ZIP) [file pone.0324264.s001.zip › supplement.material-1/Immunohistochemistry image/CD31/model-4.tif]

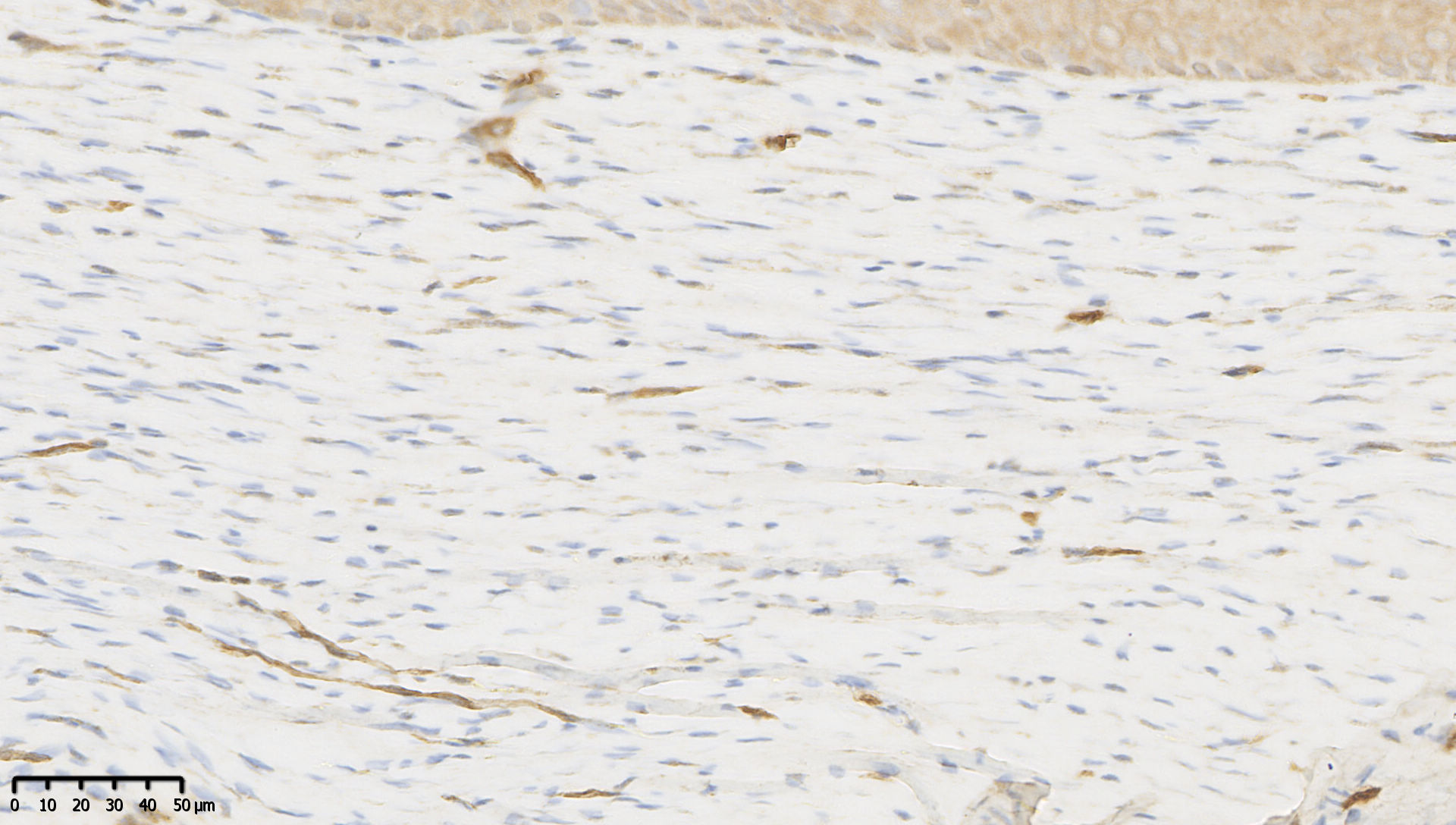

Supplement: S1 File — (ZIP) [file pone.0324264.s001.zip › supplement.material-1/Immunohistochemistry image/CD31/model-5.jpg]

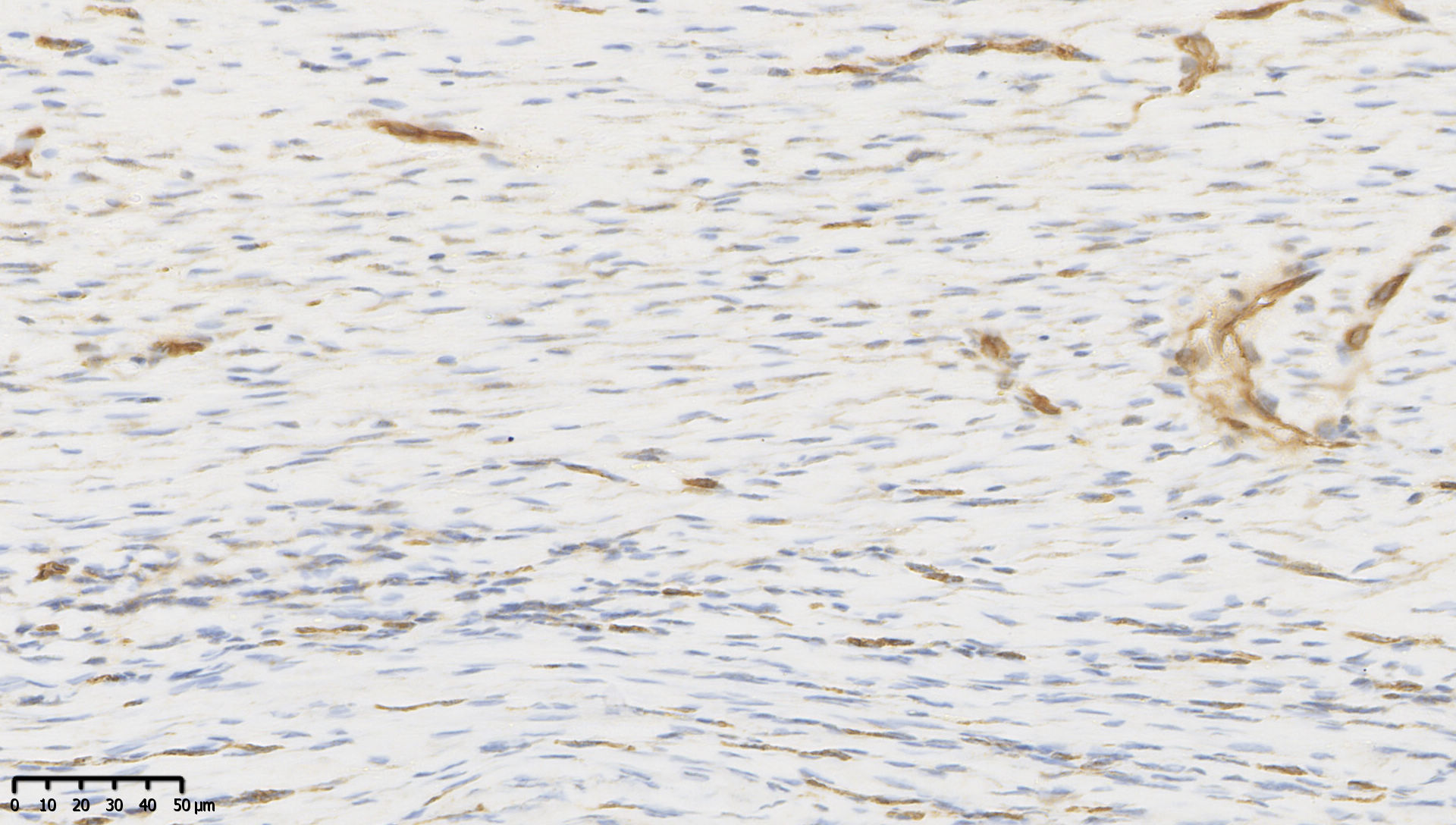

Supplement: S1 File — (ZIP) [file pone.0324264.s001.zip › supplement.material-1/Immunohistochemistry image/CD31/model-6.jpg]

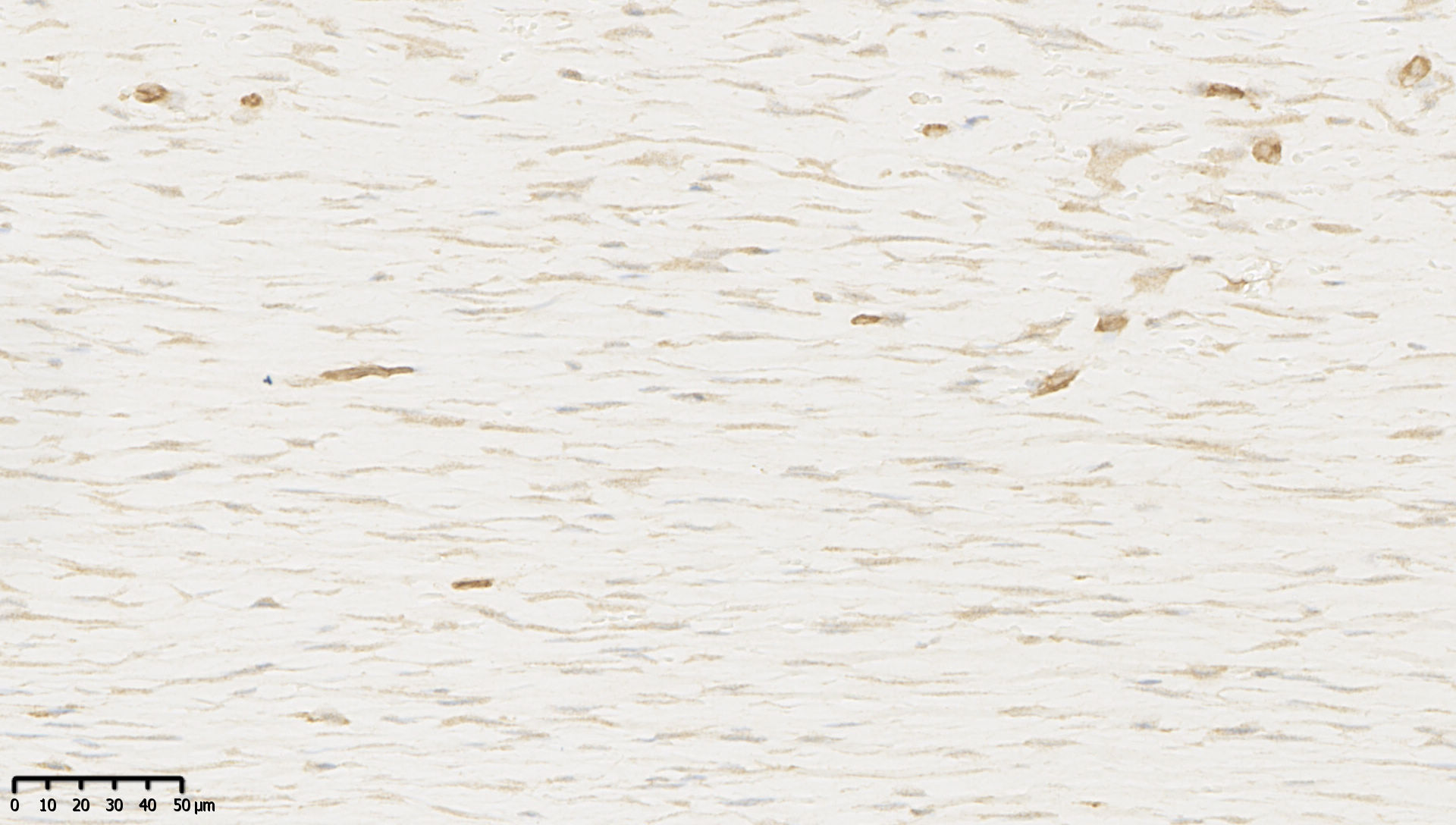

Supplement: S1 File — (ZIP) [file pone.0324264.s001.zip › supplement.material-1/Immunohistochemistry image/CD31/model-7.jpg]

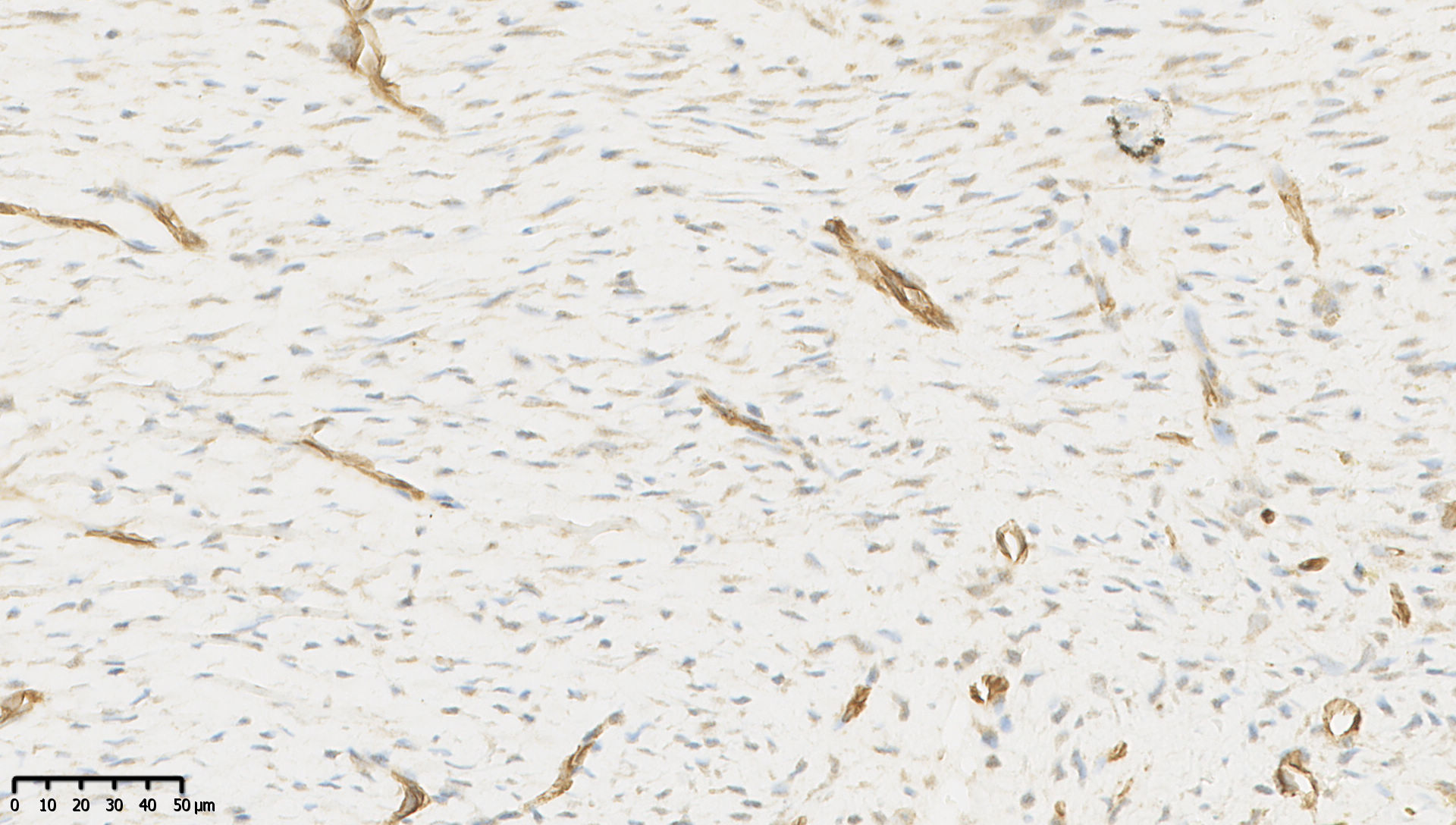

Supplement: S1 File — (ZIP) [file pone.0324264.s001.zip › supplement.material-1/Immunohistochemistry image/CD31/PL-HA-1.jpg]

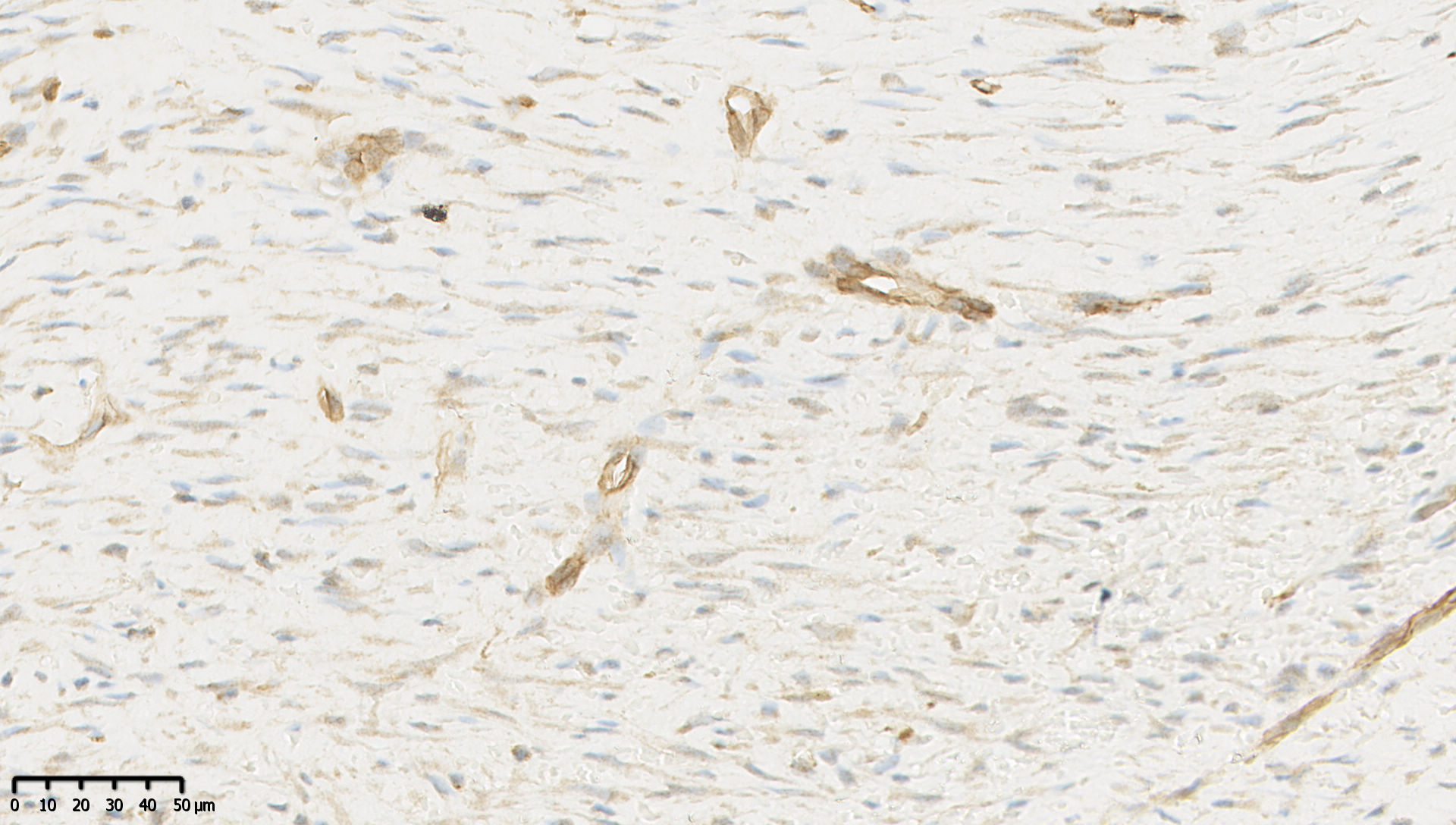

Supplement: S1 File — (ZIP) [file pone.0324264.s001.zip › supplement.material-1/Immunohistochemistry image/CD31/PL-HA-2.jpg]

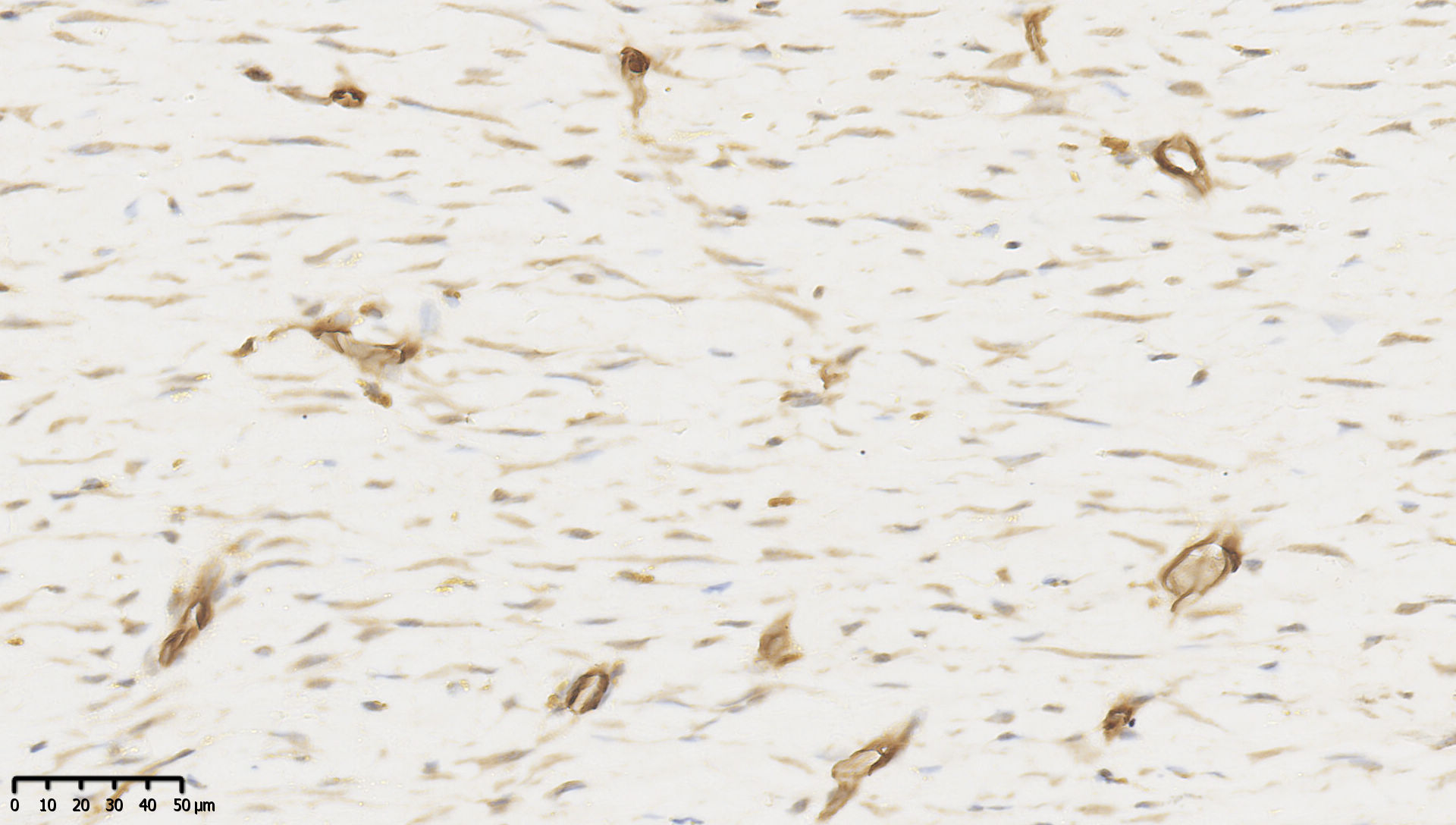

Supplement: S1 File — (ZIP) [file pone.0324264.s001.zip › supplement.material-1/Immunohistochemistry image/CD31/PL-HA-3.jpg]

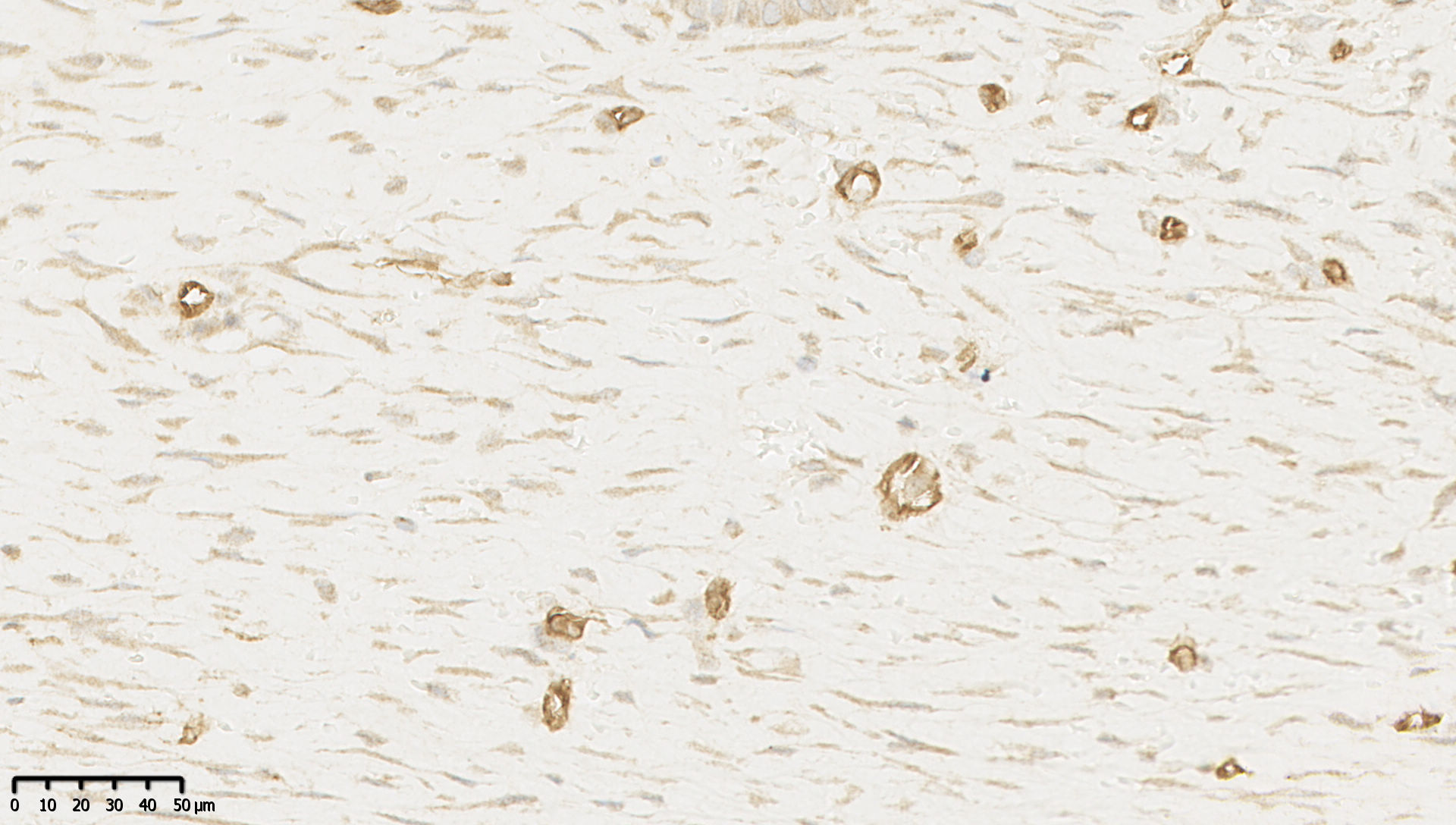

Supplement: S1 File — (ZIP) [file pone.0324264.s001.zip › supplement.material-1/Immunohistochemistry image/CD31/PL-HA-4.jpg]

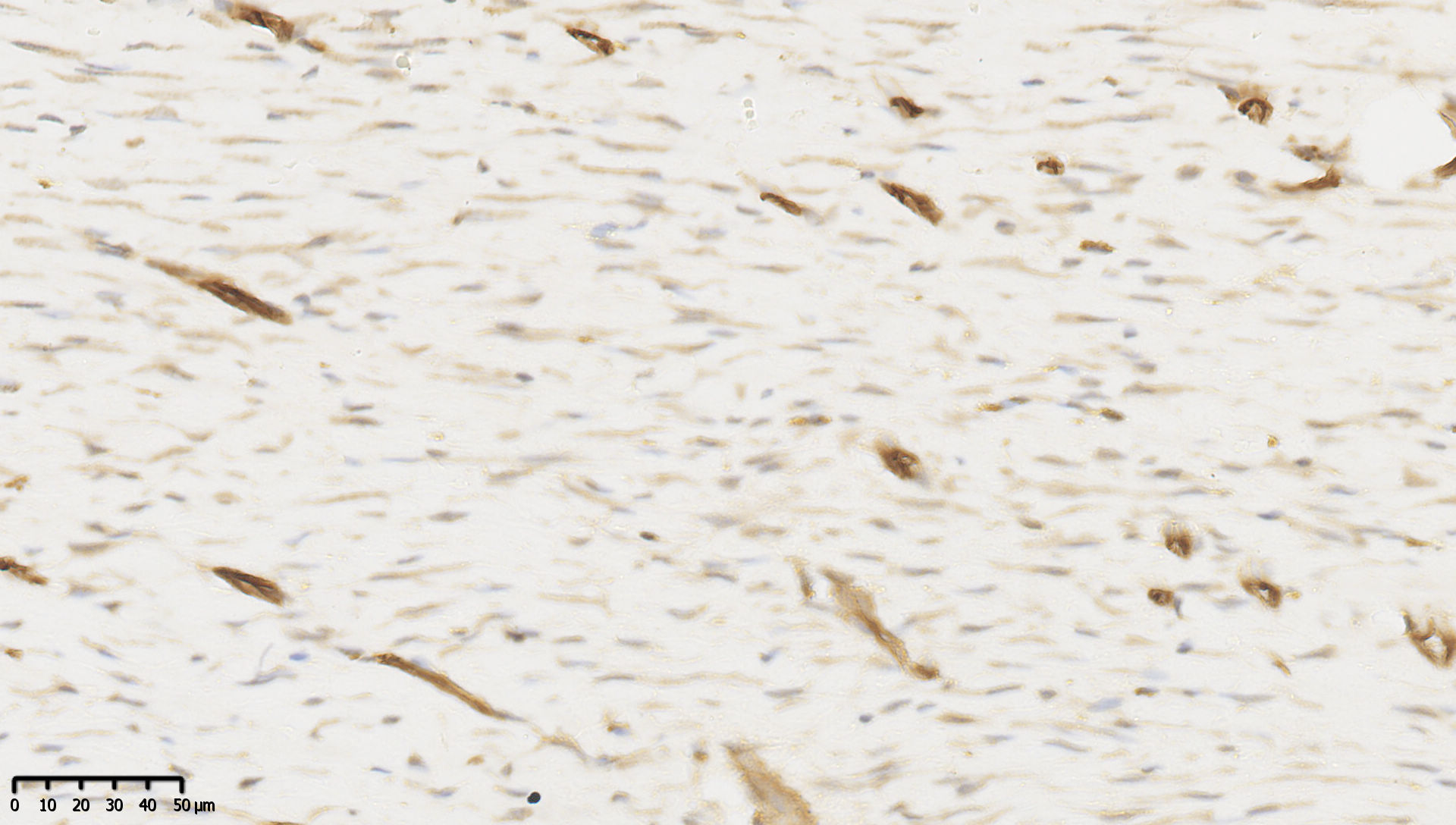

Supplement: S1 File — (ZIP) [file pone.0324264.s001.zip › supplement.material-1/Immunohistochemistry image/CD31/PL-HA-5.jpg]

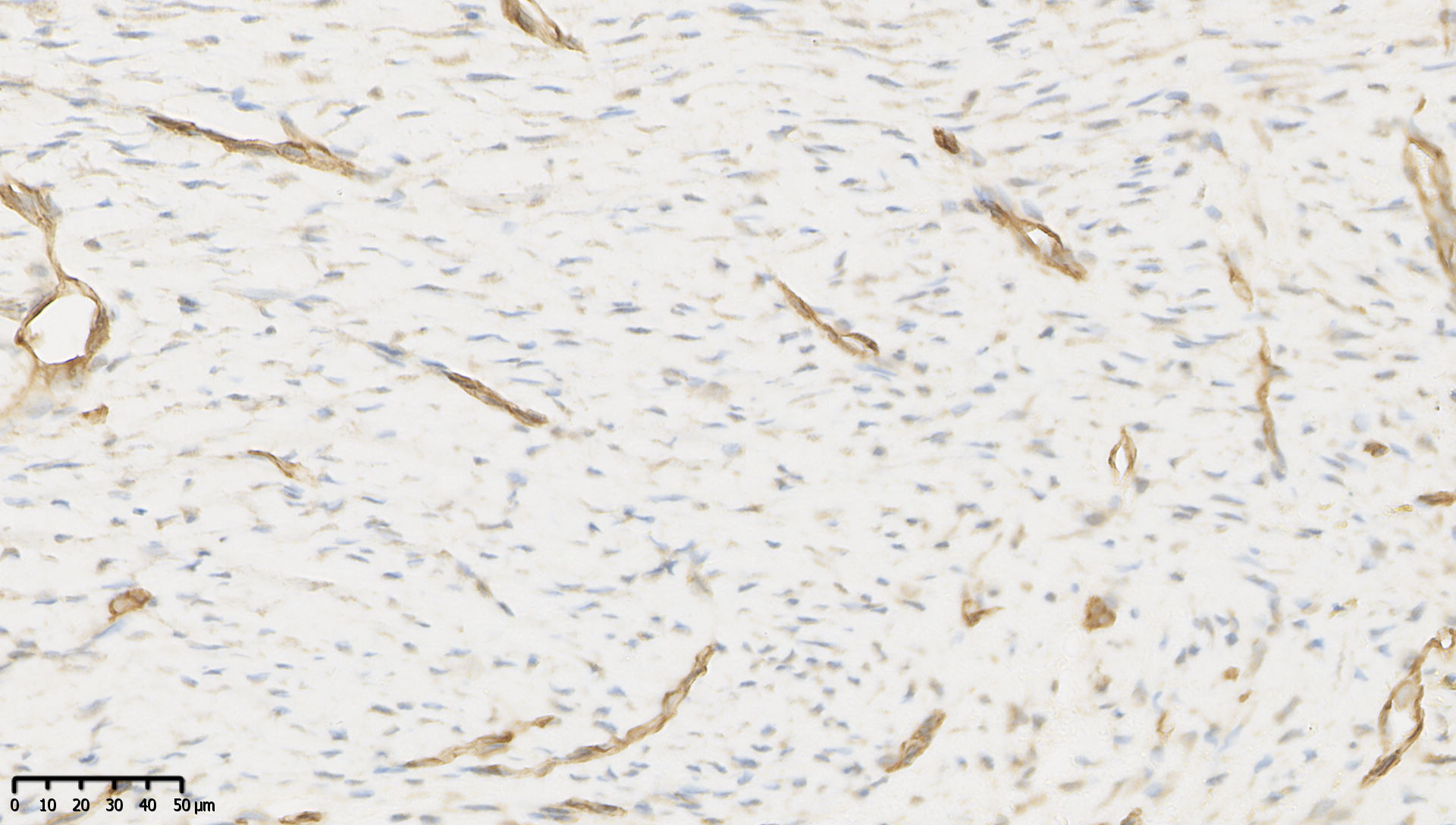

Supplement: S1 File — (ZIP) [file pone.0324264.s001.zip › supplement.material-1/Immunohistochemistry image/CD31/PL-HA-6.jpg]

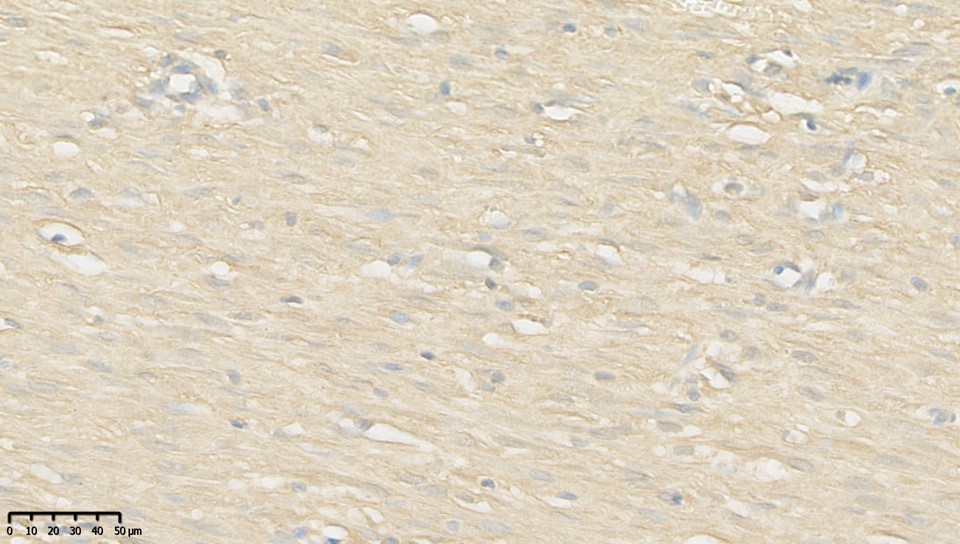

Supplement: S1 File — (ZIP) [file pone.0324264.s001.zip › supplement.material-1/Immunohistochemistry image/COL1/control-11.jpg]

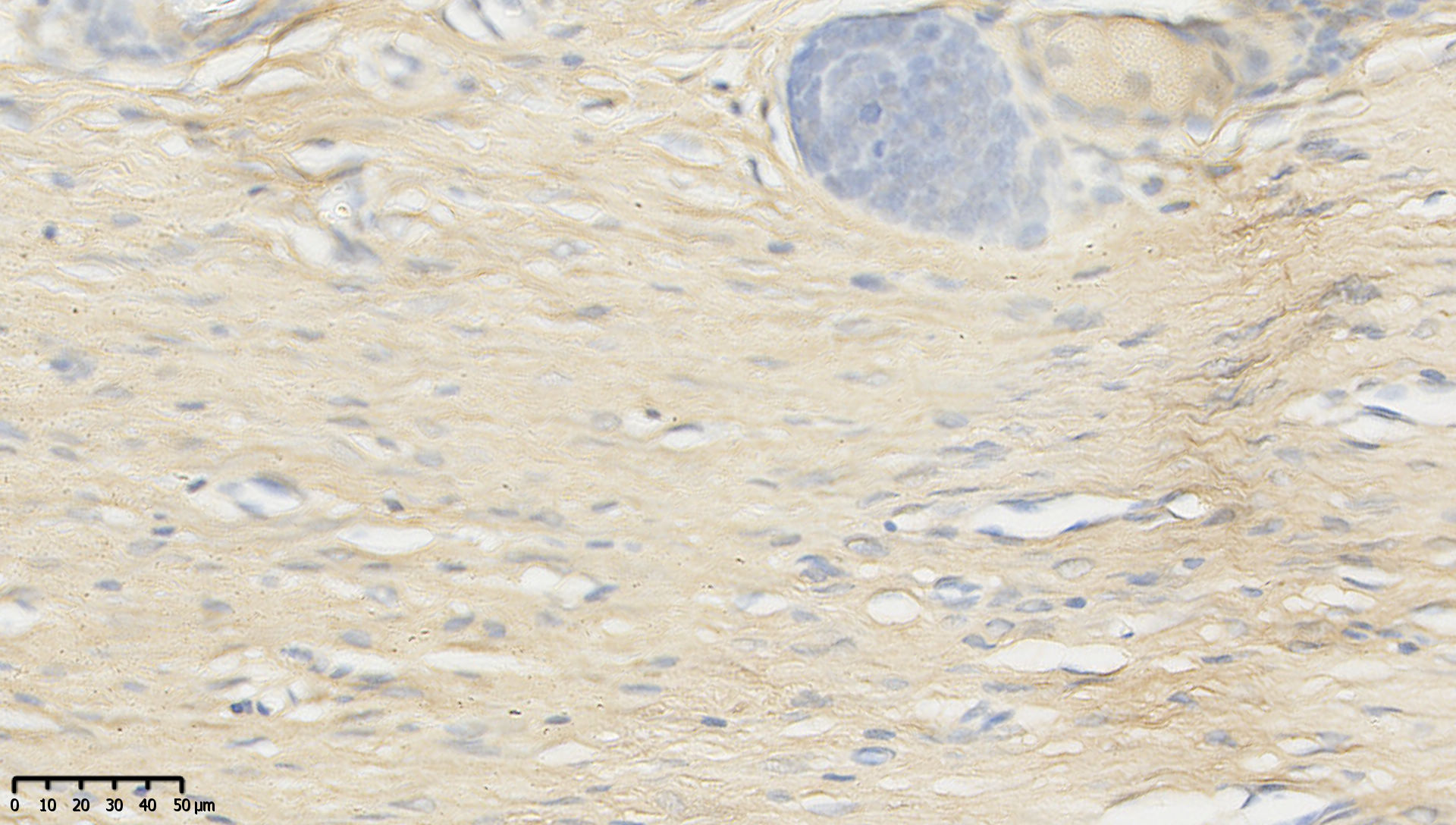

Supplement: S1 File — (ZIP) [file pone.0324264.s001.zip › supplement.material-1/Immunohistochemistry image/COL1/control-12.jpg]

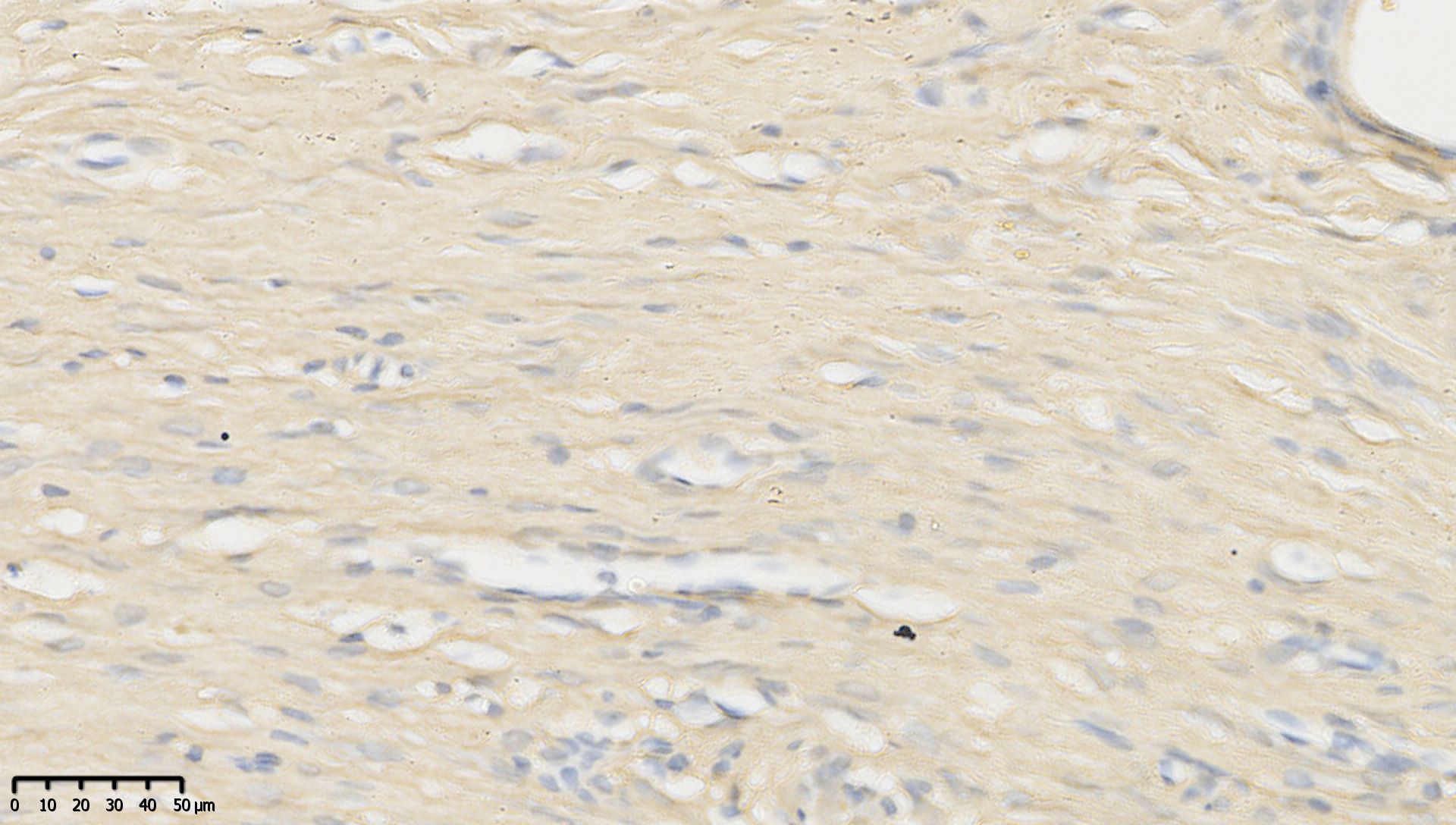

Supplement: S1 File — (ZIP) [file pone.0324264.s001.zip › supplement.material-1/Immunohistochemistry image/COL1/control-13.jpg]

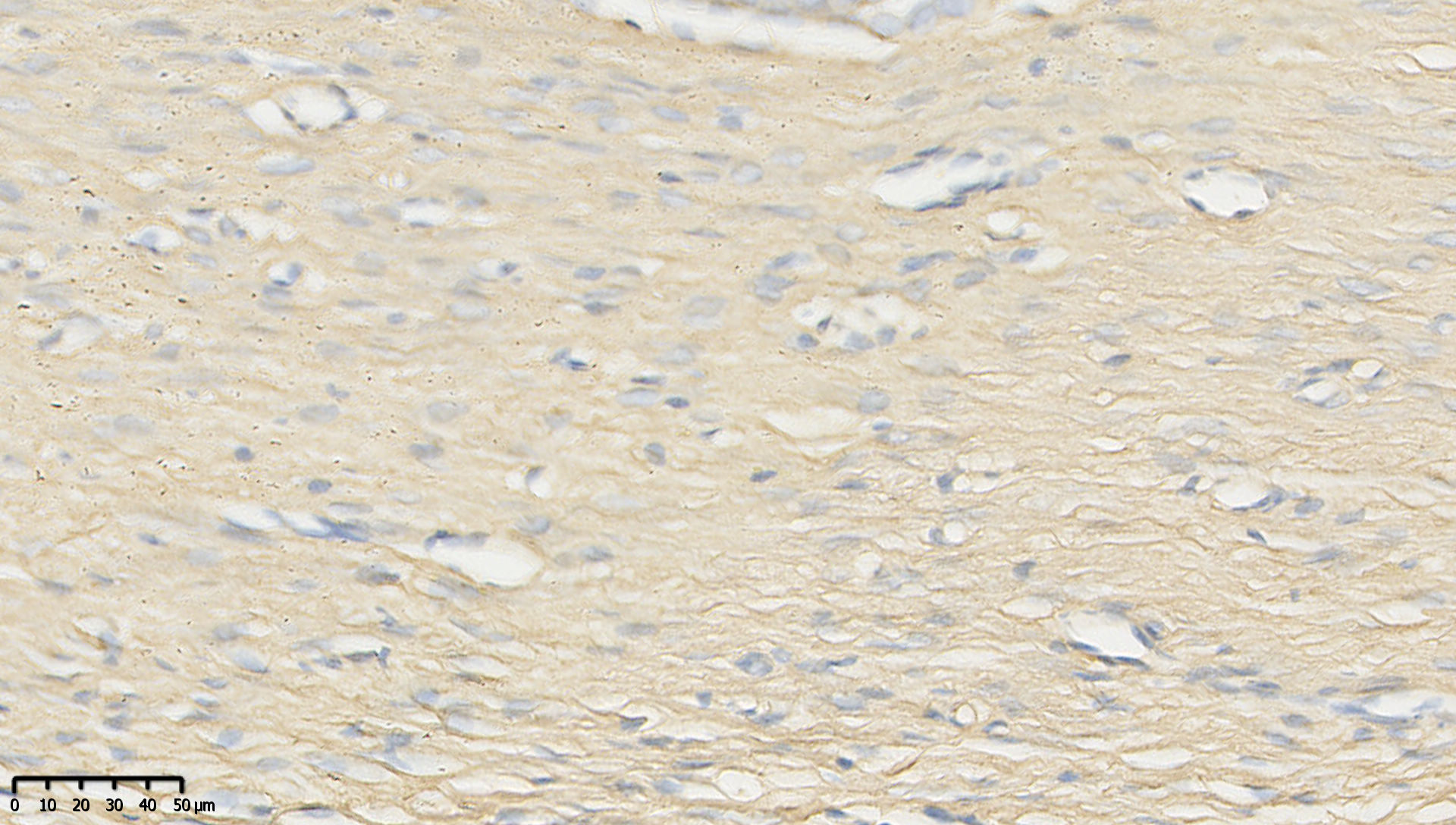

Supplement: S1 File — (ZIP) [file pone.0324264.s001.zip › supplement.material-1/Immunohistochemistry image/COL1/control-14.jpg]

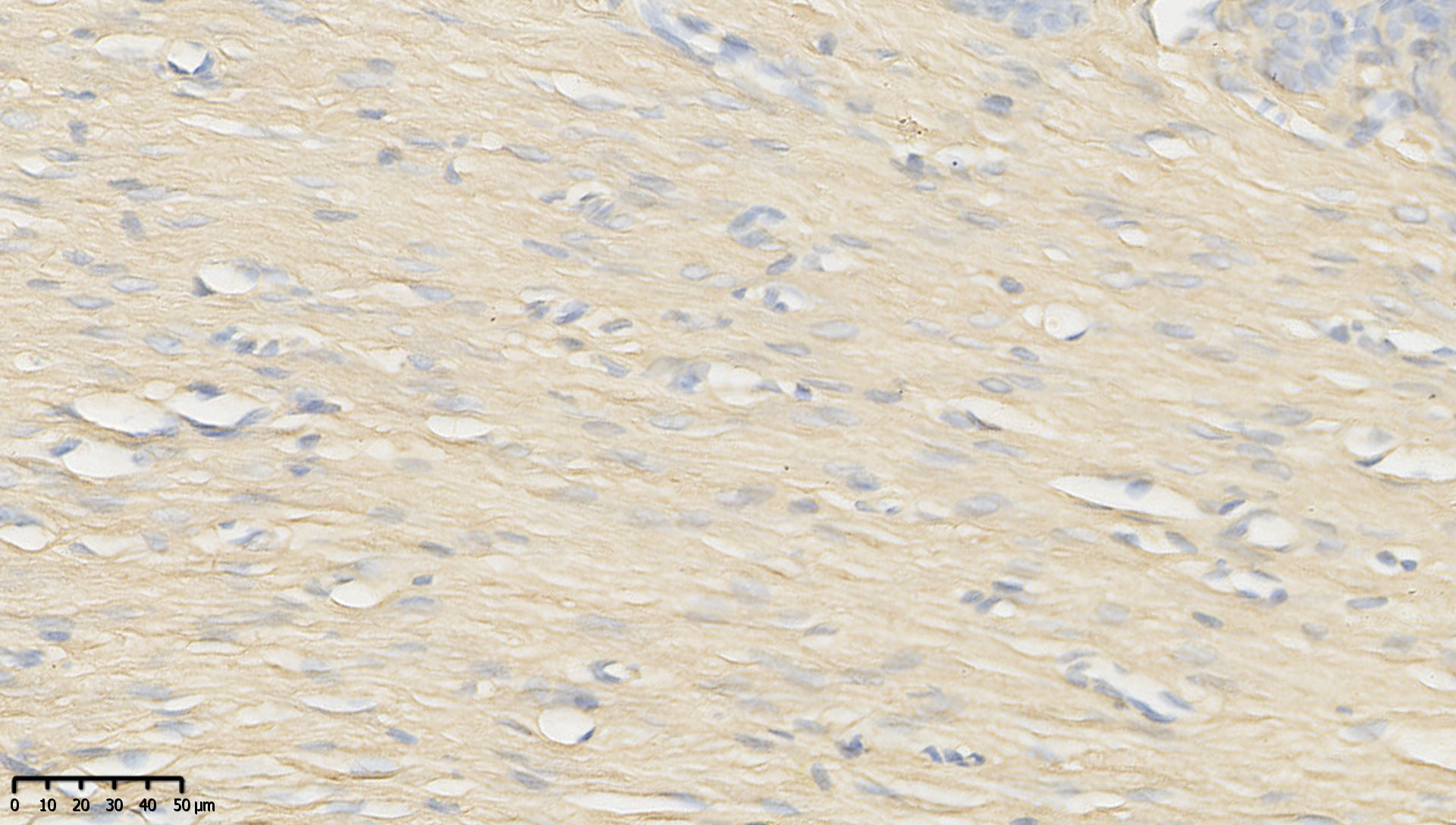

Supplement: S1 File — (ZIP) [file pone.0324264.s001.zip › supplement.material-1/Immunohistochemistry image/COL1/control-15.jpg]

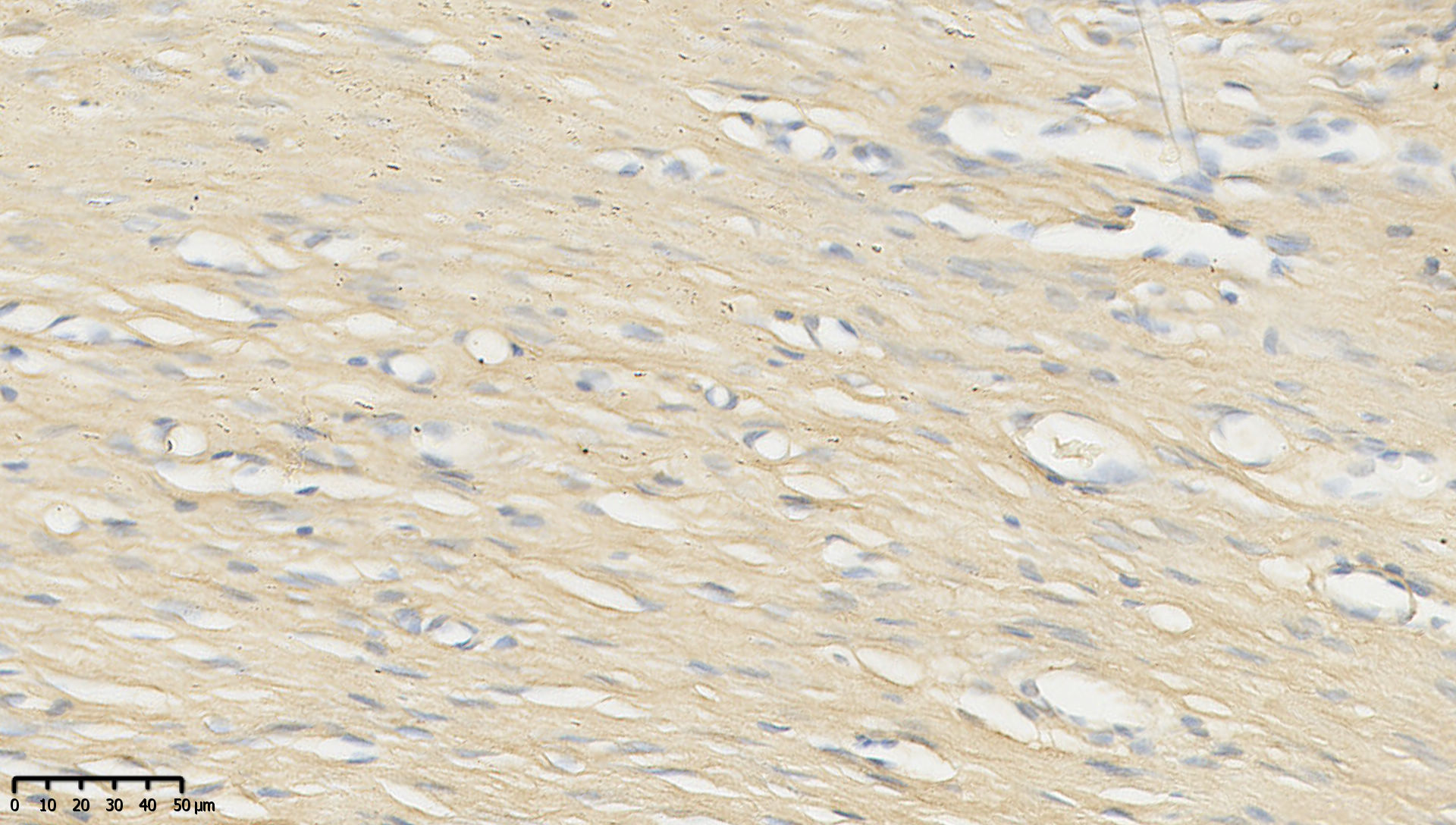

Supplement: S1 File — (ZIP) [file pone.0324264.s001.zip › supplement.material-1/Immunohistochemistry image/COL1/control-16.jpg]

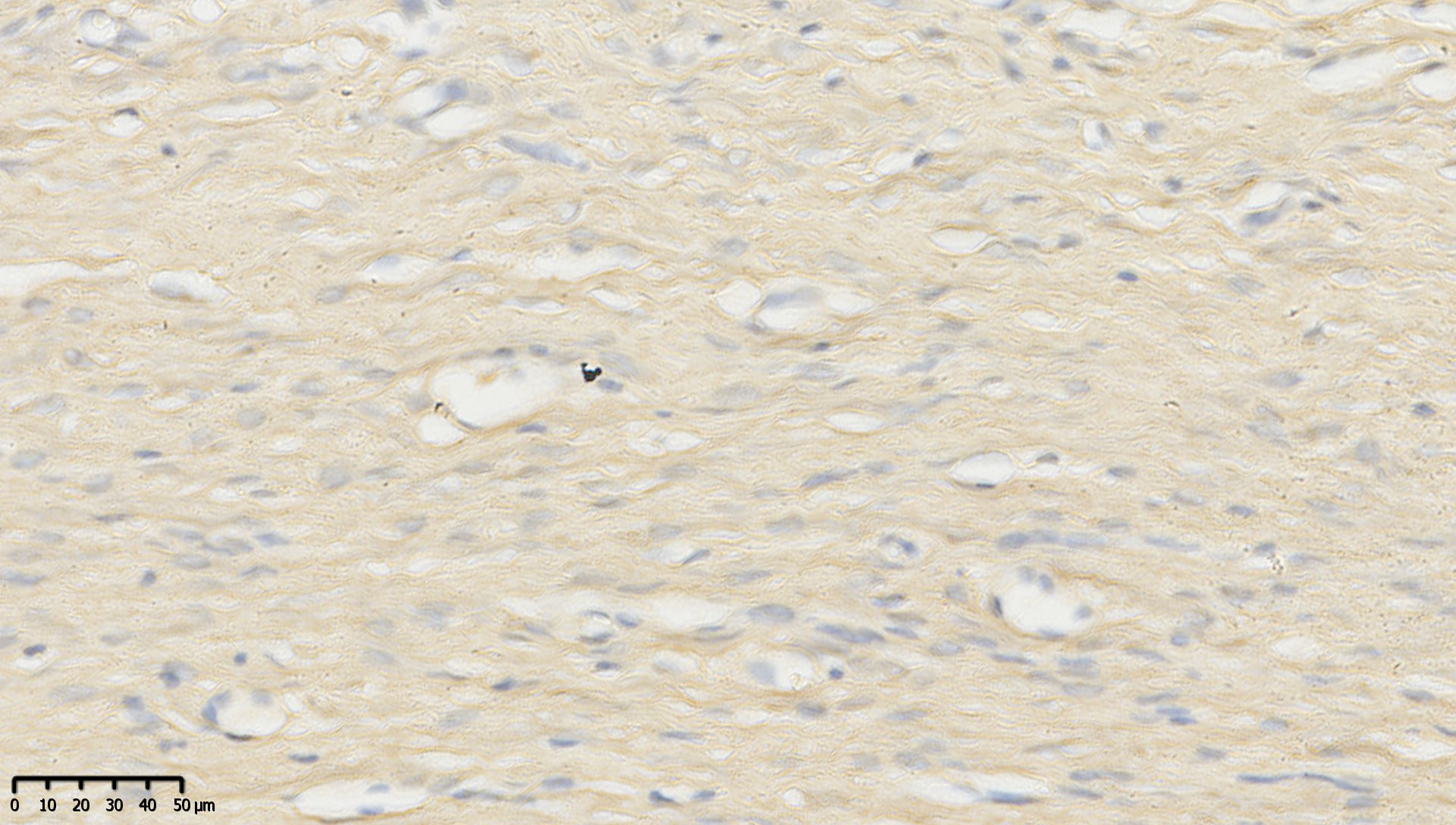

Supplement: S1 File — (ZIP) [file pone.0324264.s001.zip › supplement.material-1/Immunohistochemistry image/COL1/control-17.jpg]

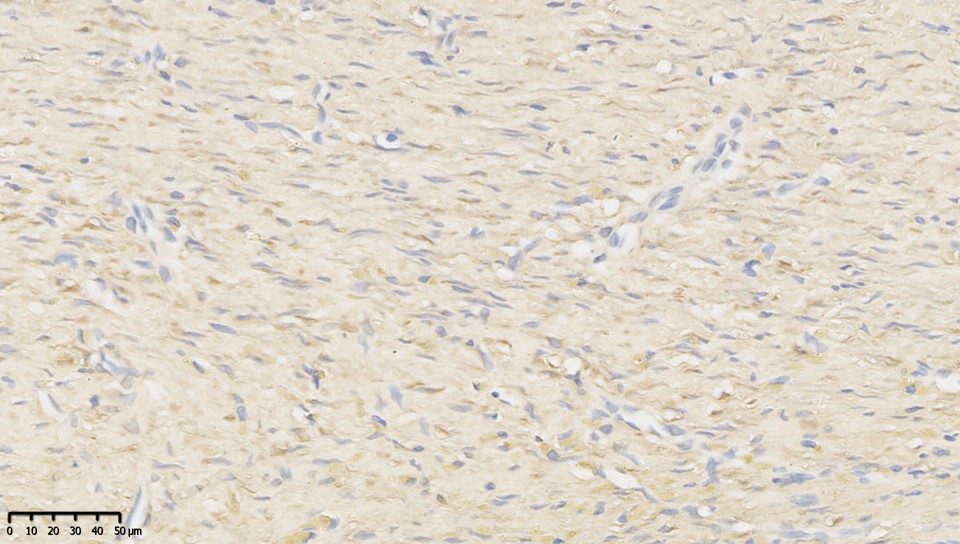

Supplement: S1 File — (ZIP) [file pone.0324264.s001.zip › supplement.material-1/Immunohistochemistry image/COL1/HA-11.jpg]

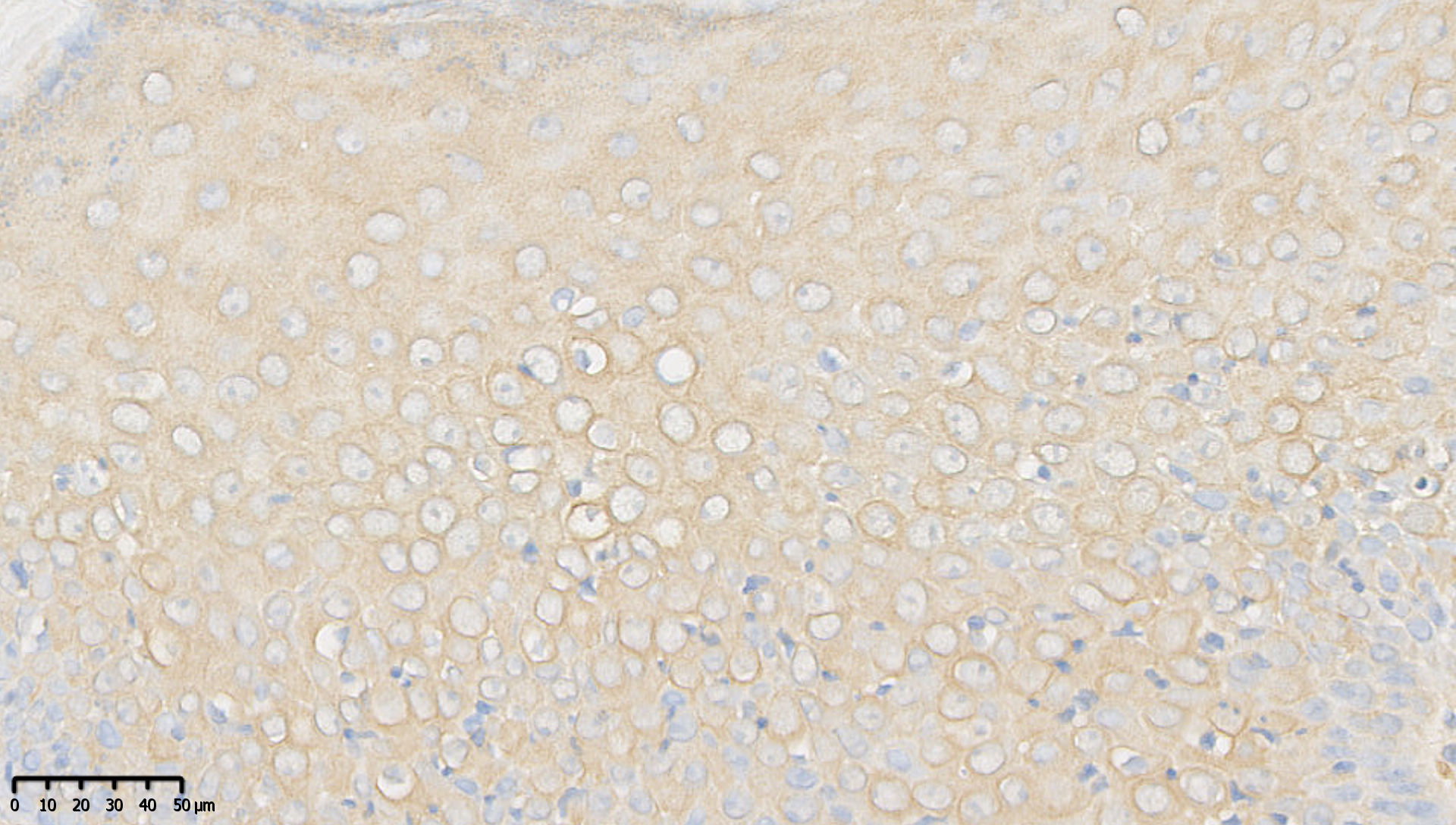

Supplement: S1 File — (ZIP) [file pone.0324264.s001.zip › supplement.material-1/Immunohistochemistry image/COL1/HA-12.jpg]

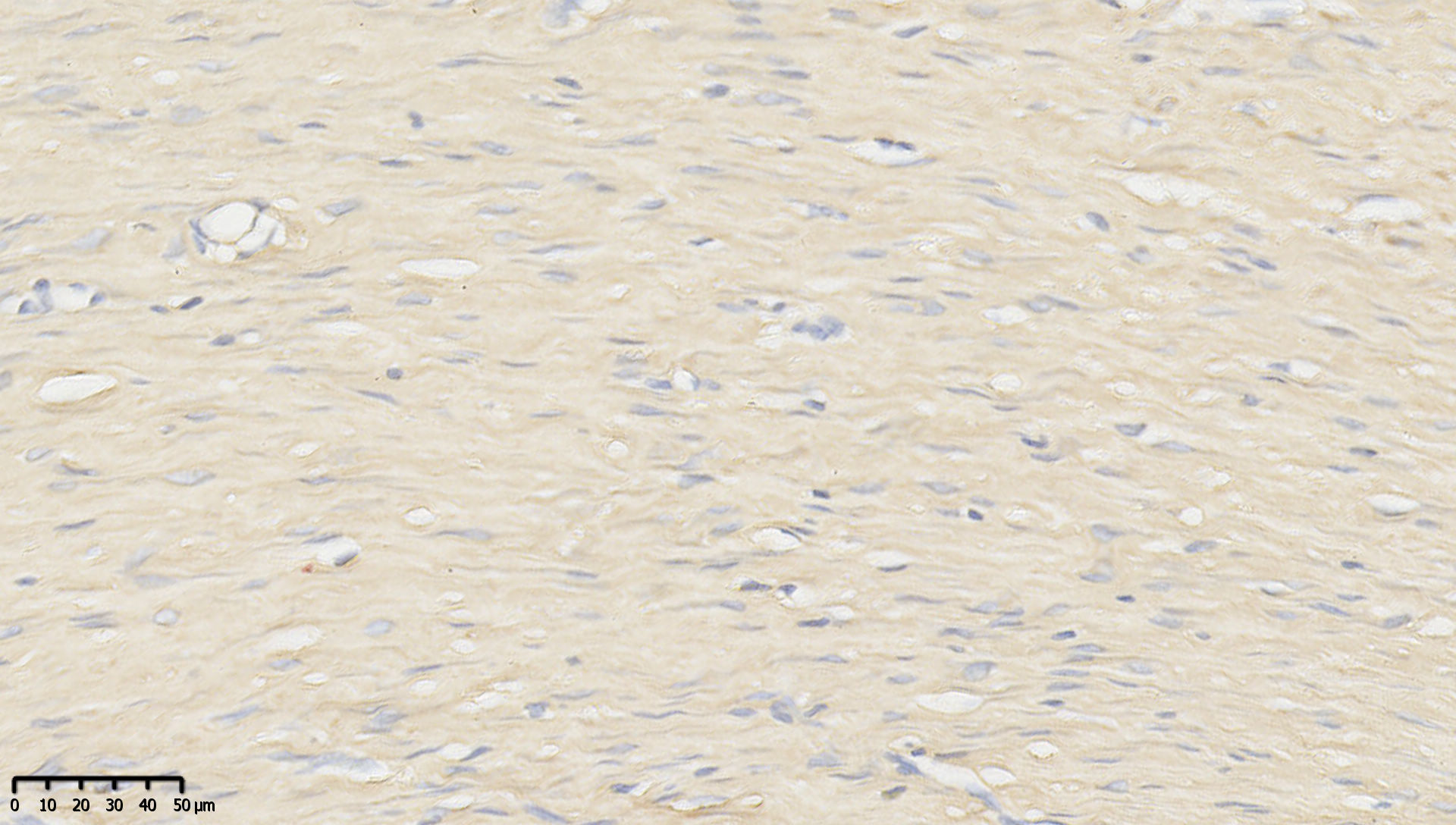

Supplement: S1 File — (ZIP) [file pone.0324264.s001.zip › supplement.material-1/Immunohistochemistry image/COL1/HA-13.jpg]

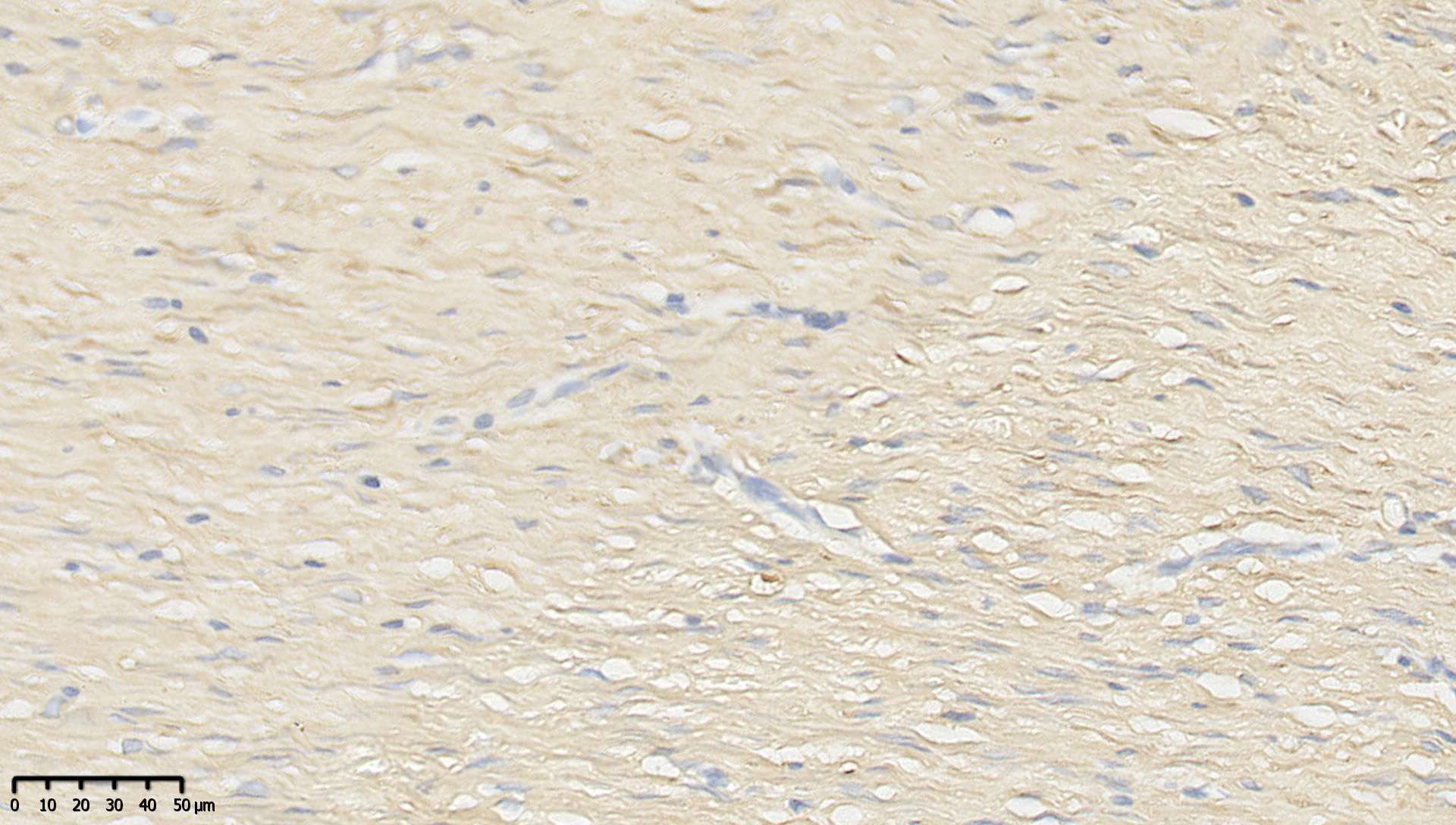

Supplement: S1 File — (ZIP) [file pone.0324264.s001.zip › supplement.material-1/Immunohistochemistry image/COL1/HA-14.jpg]

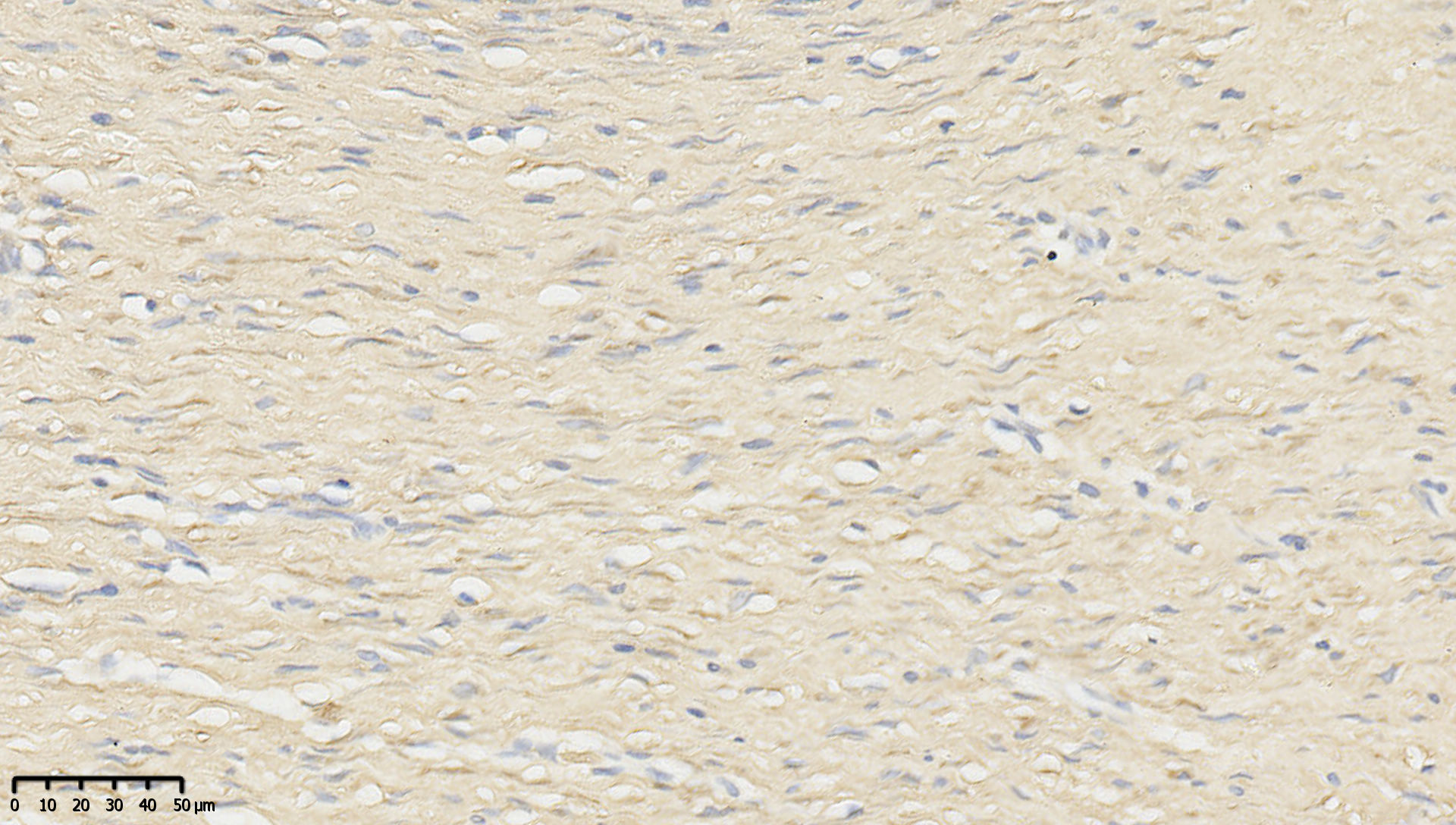

Supplement: S1 File — (ZIP) [file pone.0324264.s001.zip › supplement.material-1/Immunohistochemistry image/COL1/HA-15.jpg]

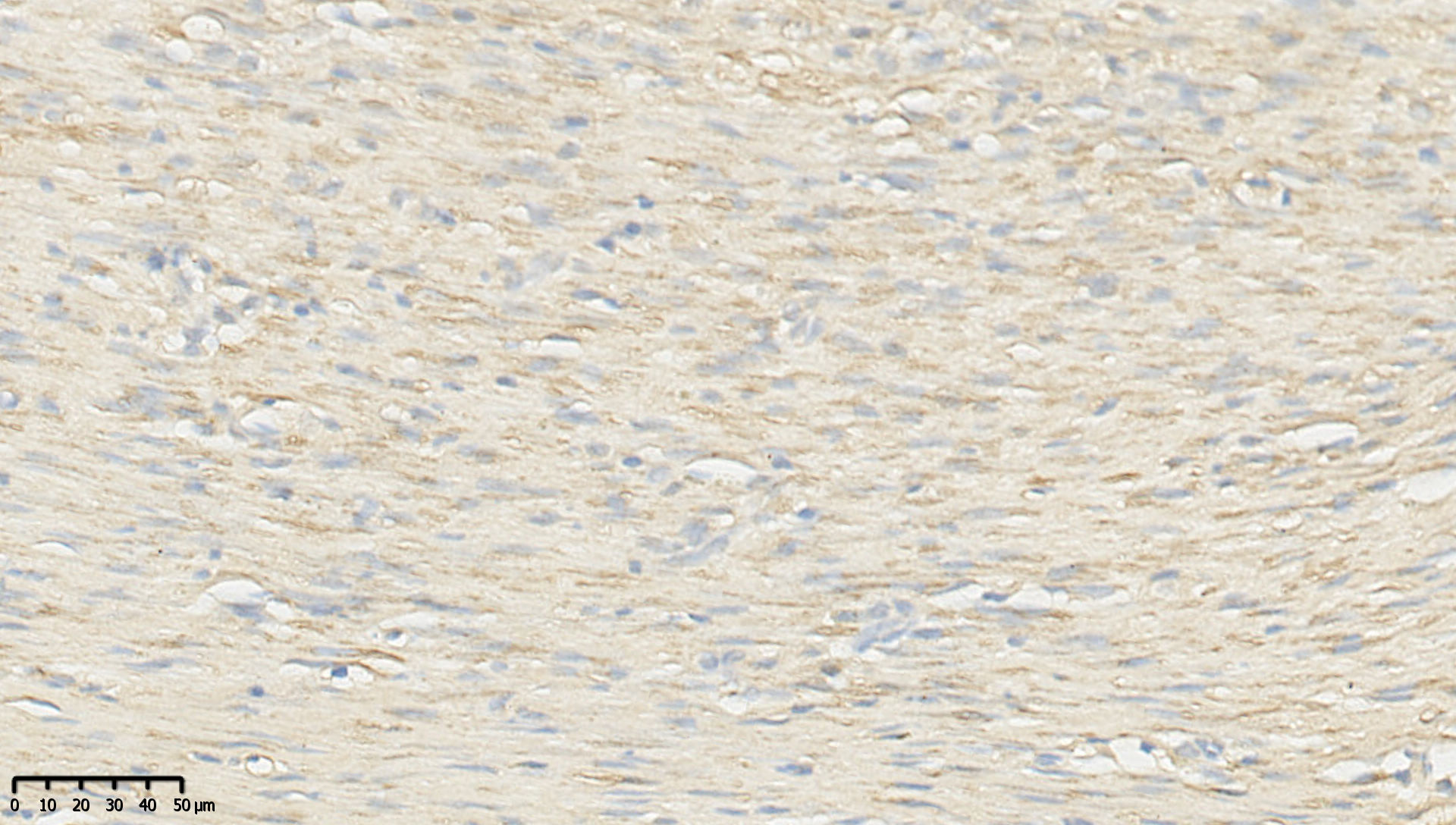

Supplement: S1 File — (ZIP) [file pone.0324264.s001.zip › supplement.material-1/Immunohistochemistry image/COL1/HA-16.jpg]

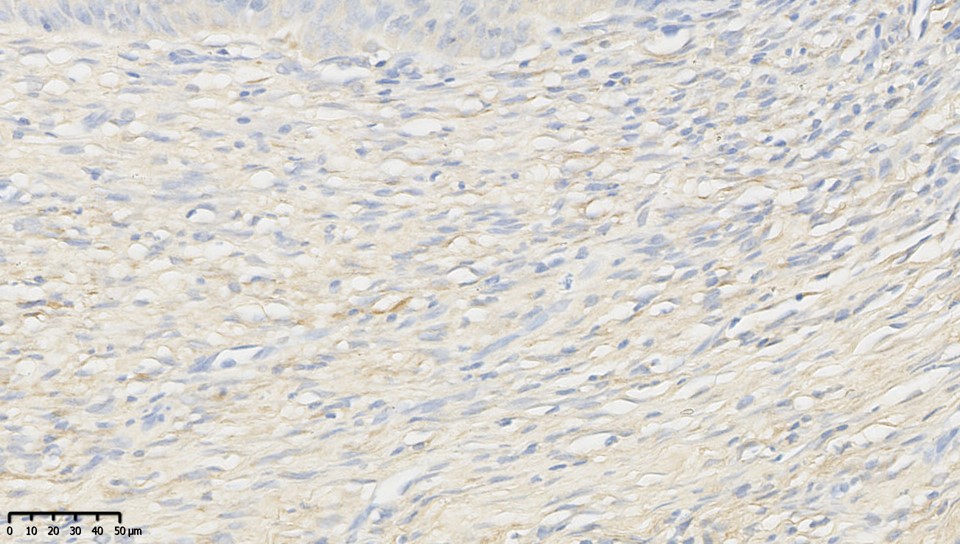

Supplement: S1 File — (ZIP) [file pone.0324264.s001.zip › supplement.material-1/Immunohistochemistry image/COL1/model-11.jpg]

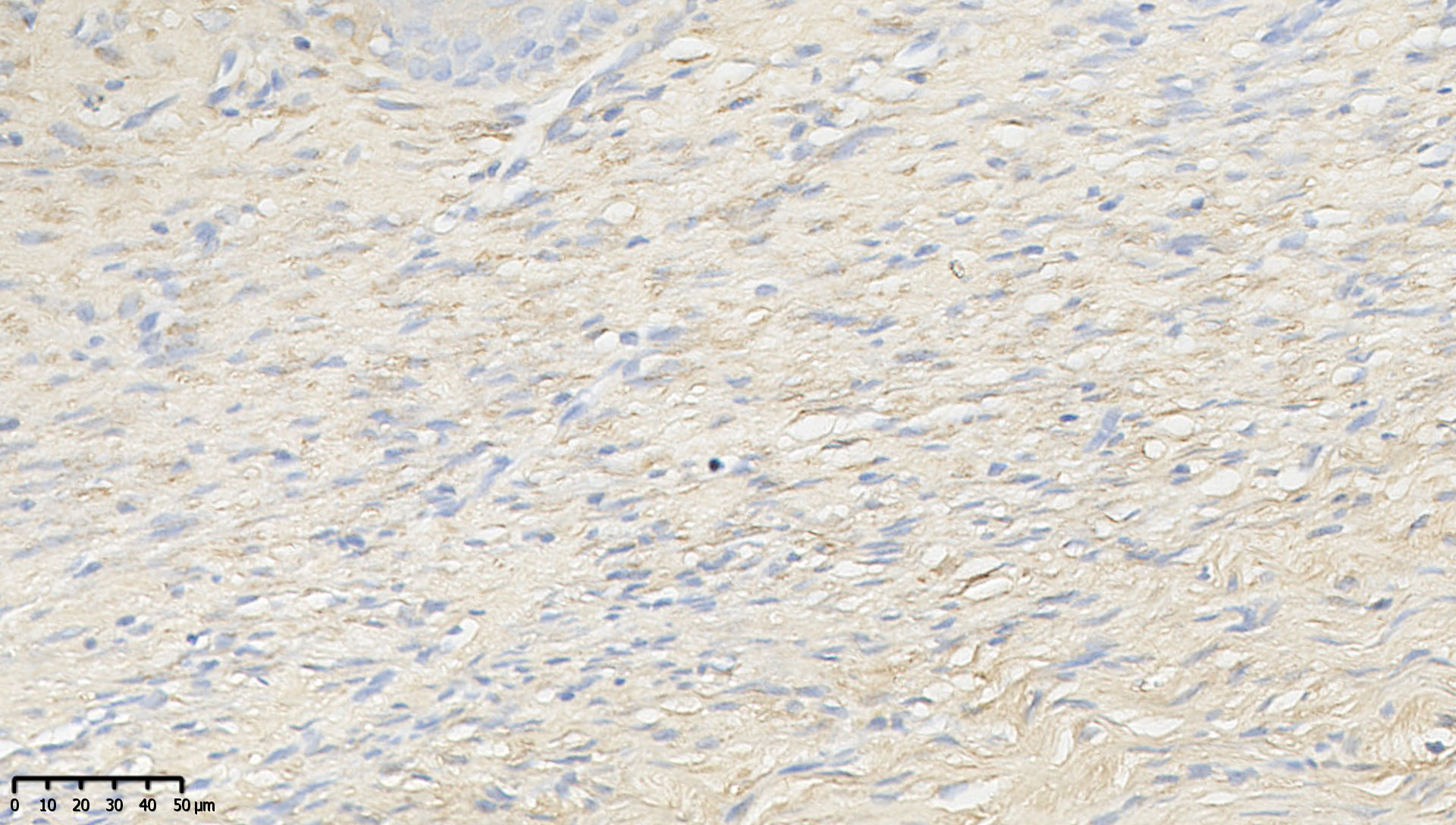

Supplement: S1 File — (ZIP) [file pone.0324264.s001.zip › supplement.material-1/Immunohistochemistry image/COL1/model-12.jpg]

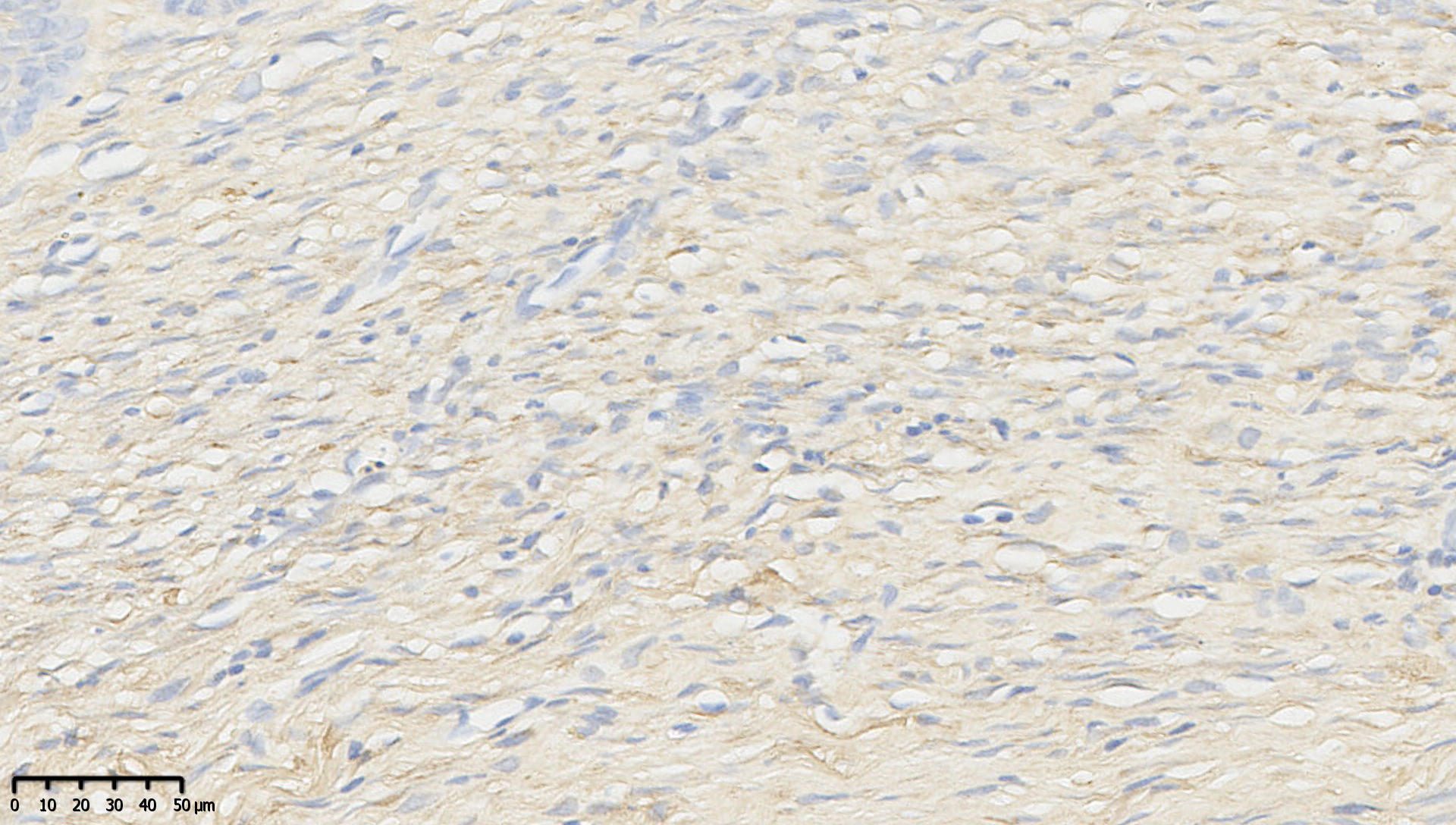

Supplement: S1 File — (ZIP) [file pone.0324264.s001.zip › supplement.material-1/Immunohistochemistry image/COL1/model-13.jpg]

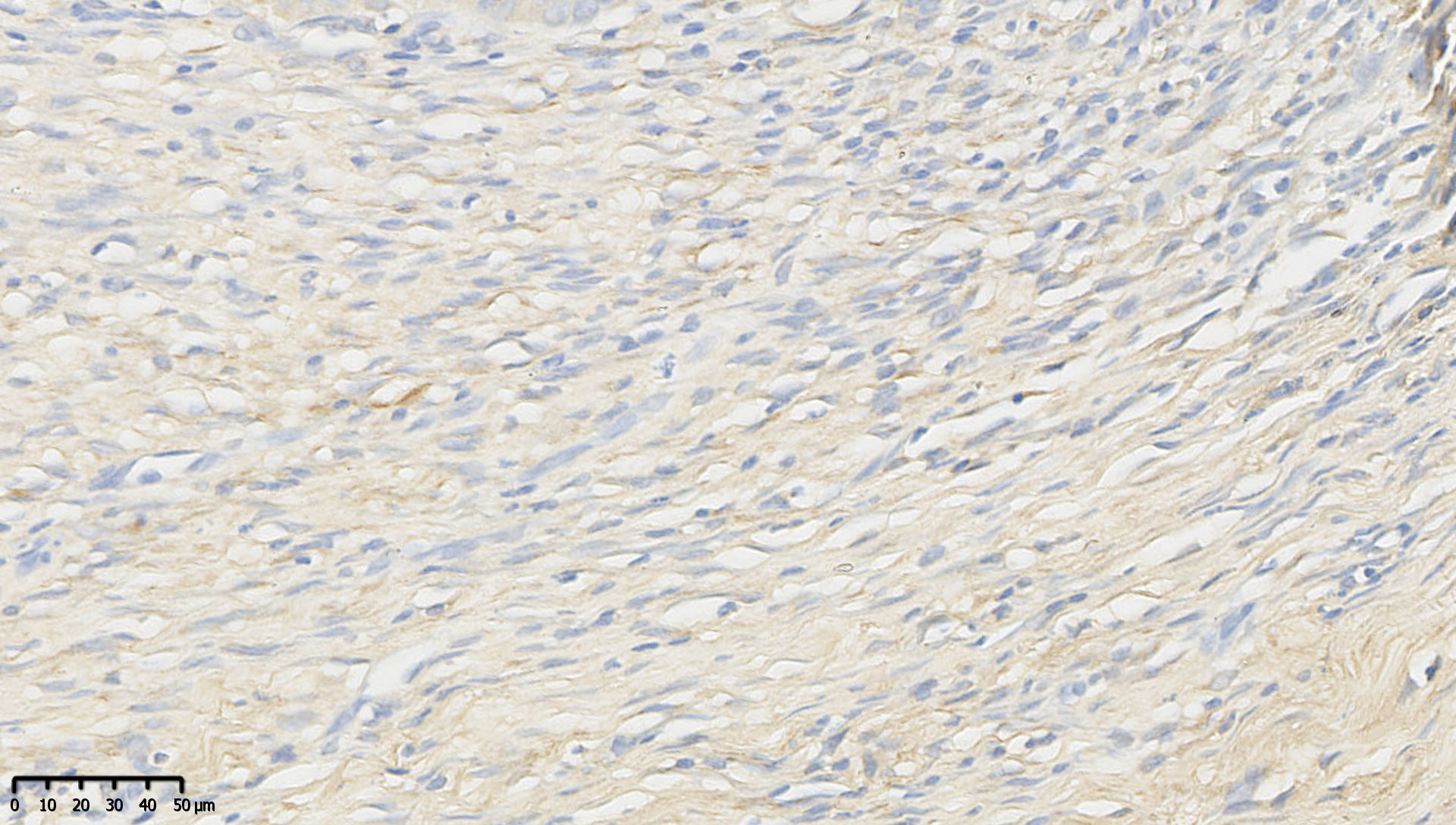

Supplement: S1 File — (ZIP) [file pone.0324264.s001.zip › supplement.material-1/Immunohistochemistry image/COL1/model-14.jpg]

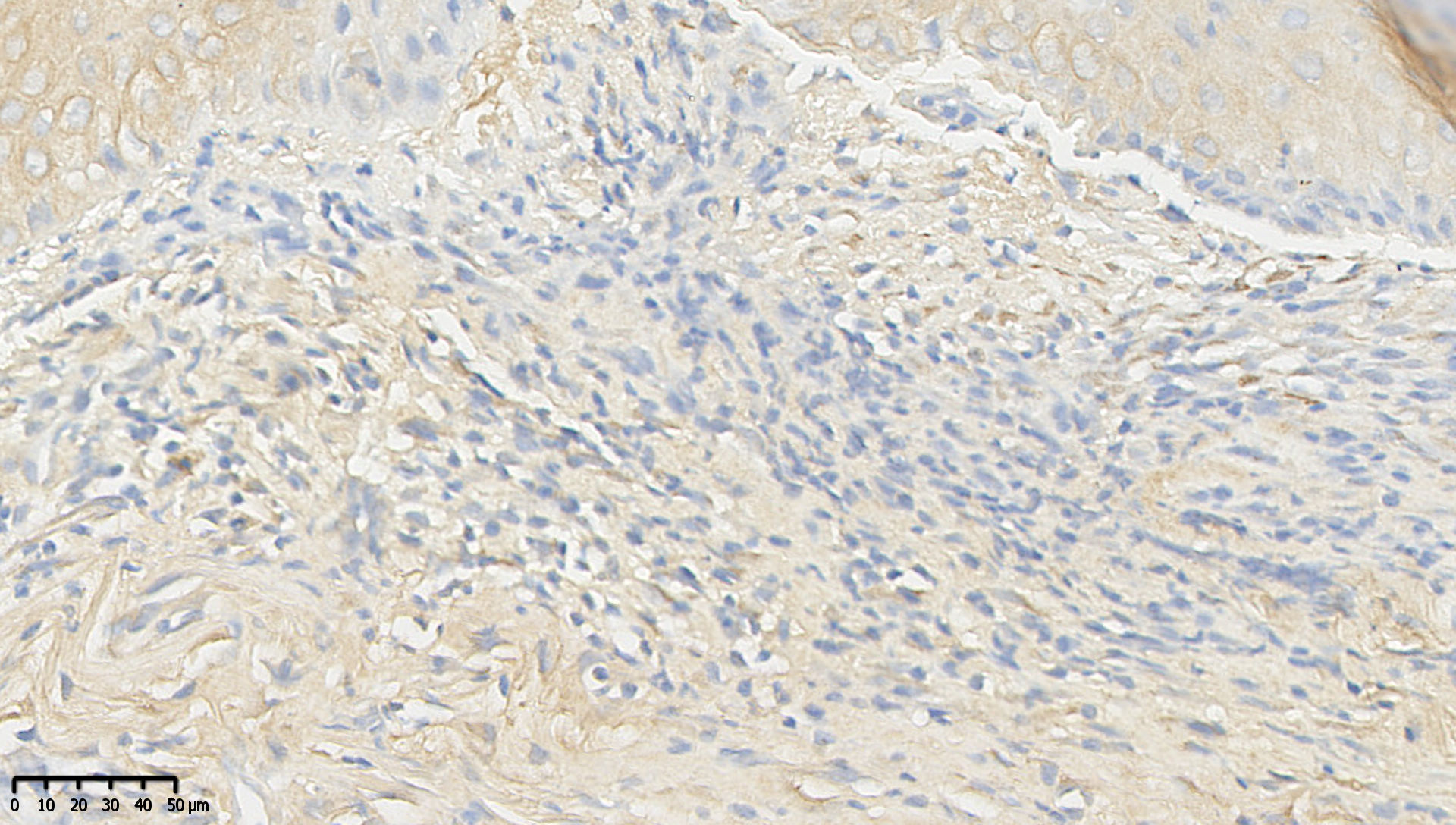

Supplement: S1 File — (ZIP) [file pone.0324264.s001.zip › supplement.material-1/Immunohistochemistry image/COL1/model-15.jpg]

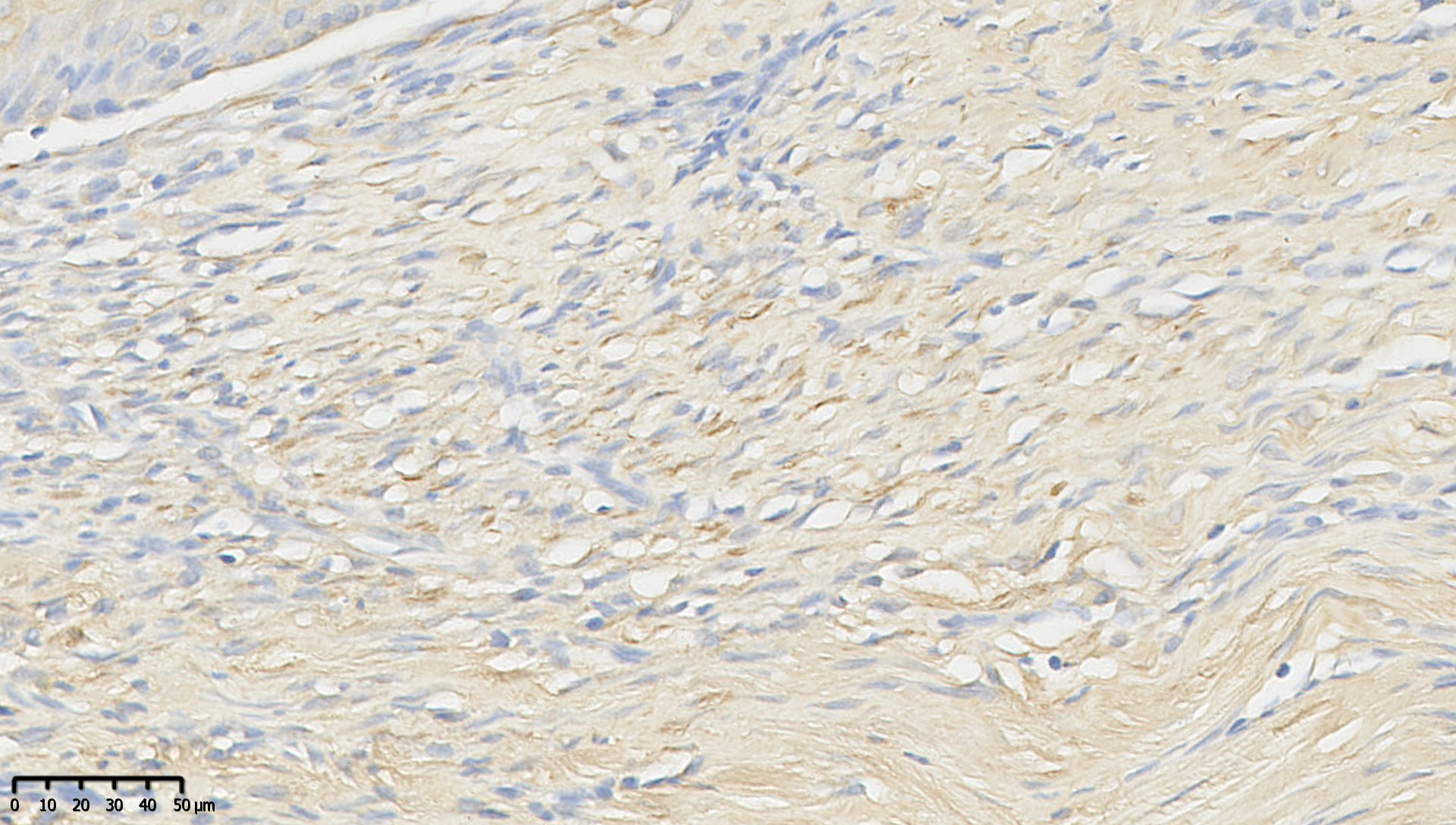

Supplement: S1 File — (ZIP) [file pone.0324264.s001.zip › supplement.material-1/Immunohistochemistry image/COL1/model-16.jpg]

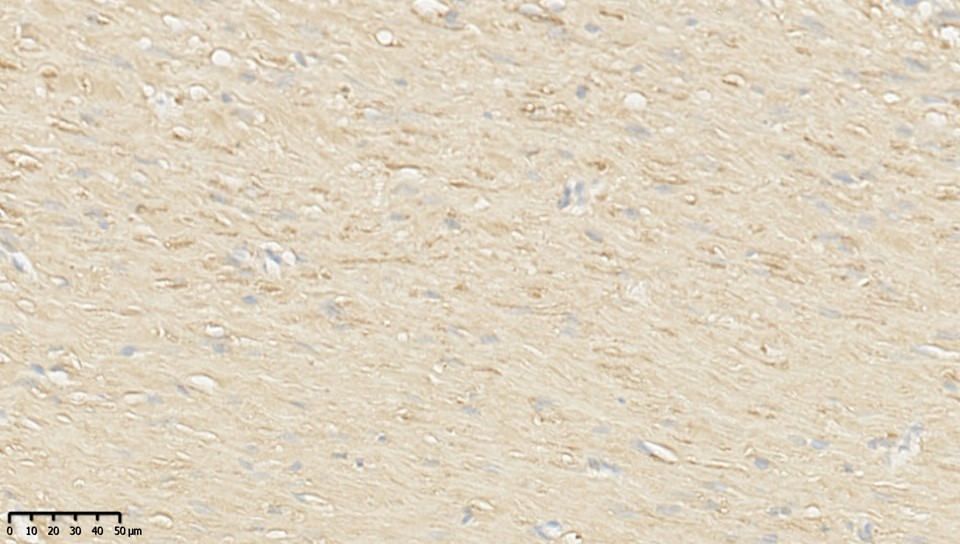

Supplement: S1 File — (ZIP) [file pone.0324264.s001.zip › supplement.material-1/Immunohistochemistry image/COL1/PL-HA-11.jpg]

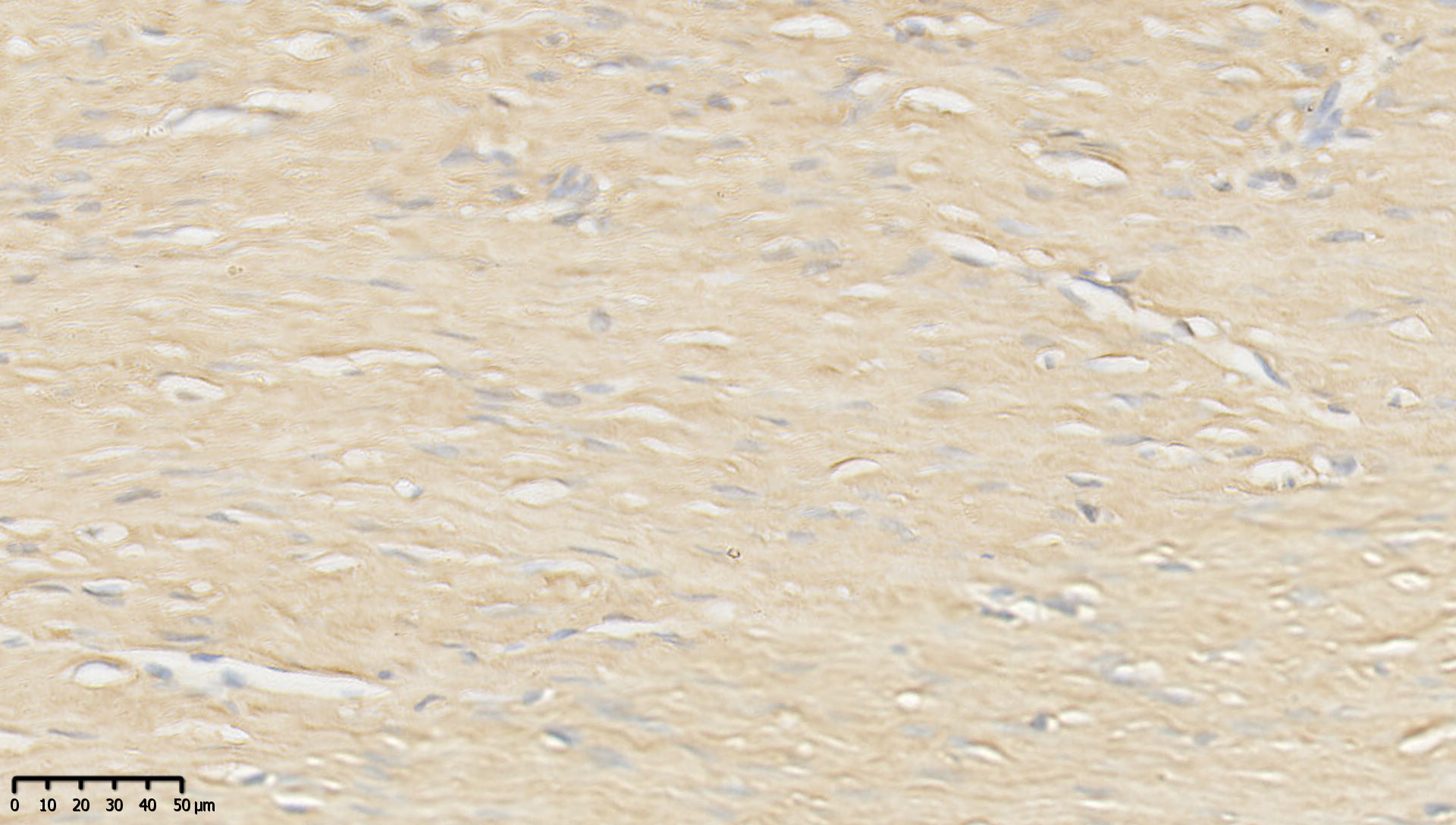

Supplement: S1 File — (ZIP) [file pone.0324264.s001.zip › supplement.material-1/Immunohistochemistry image/COL1/PL-HA-12.jpg]

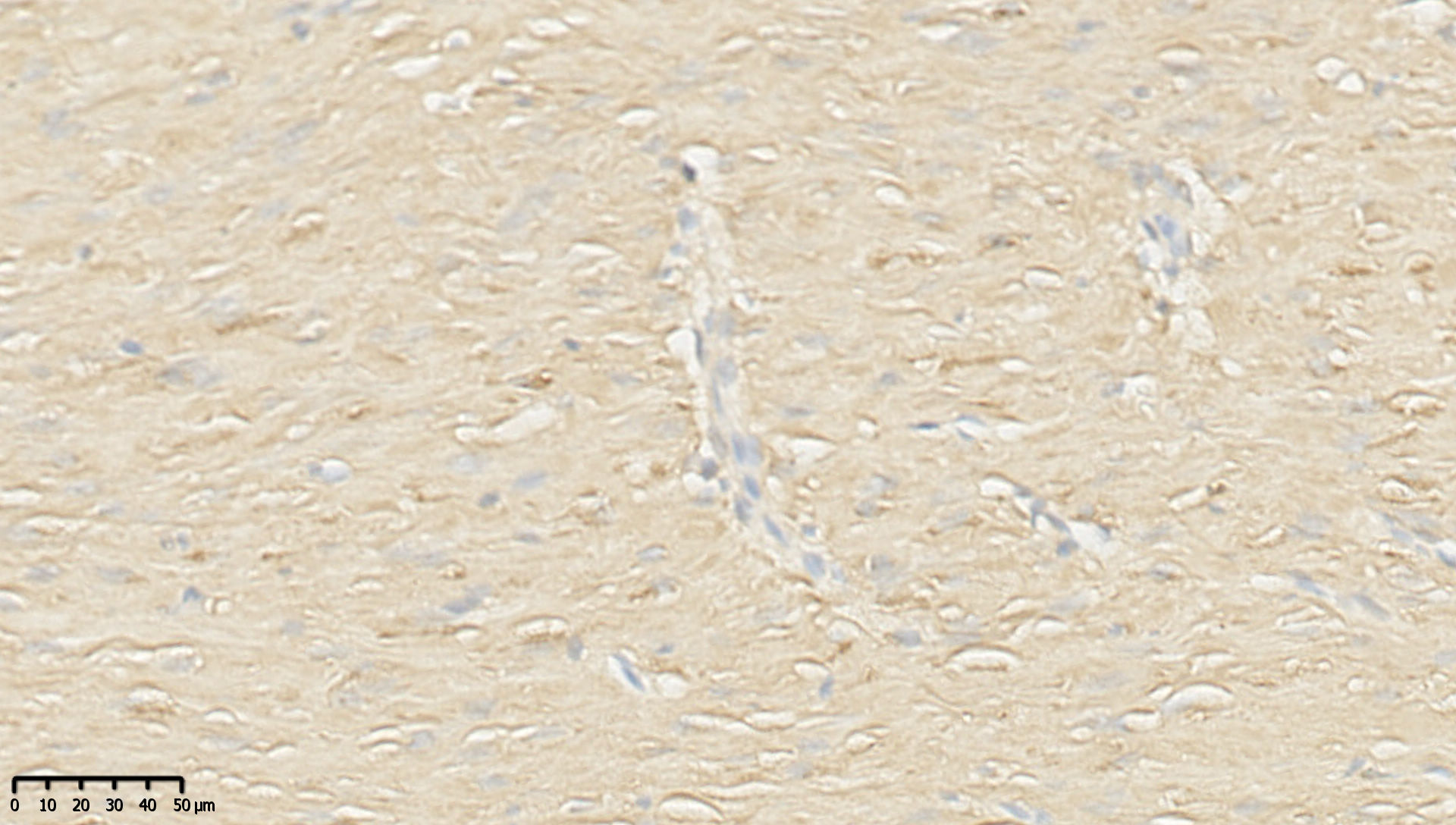

Supplement: S1 File — (ZIP) [file pone.0324264.s001.zip › supplement.material-1/Immunohistochemistry image/COL1/PL-HA-13.jpg]

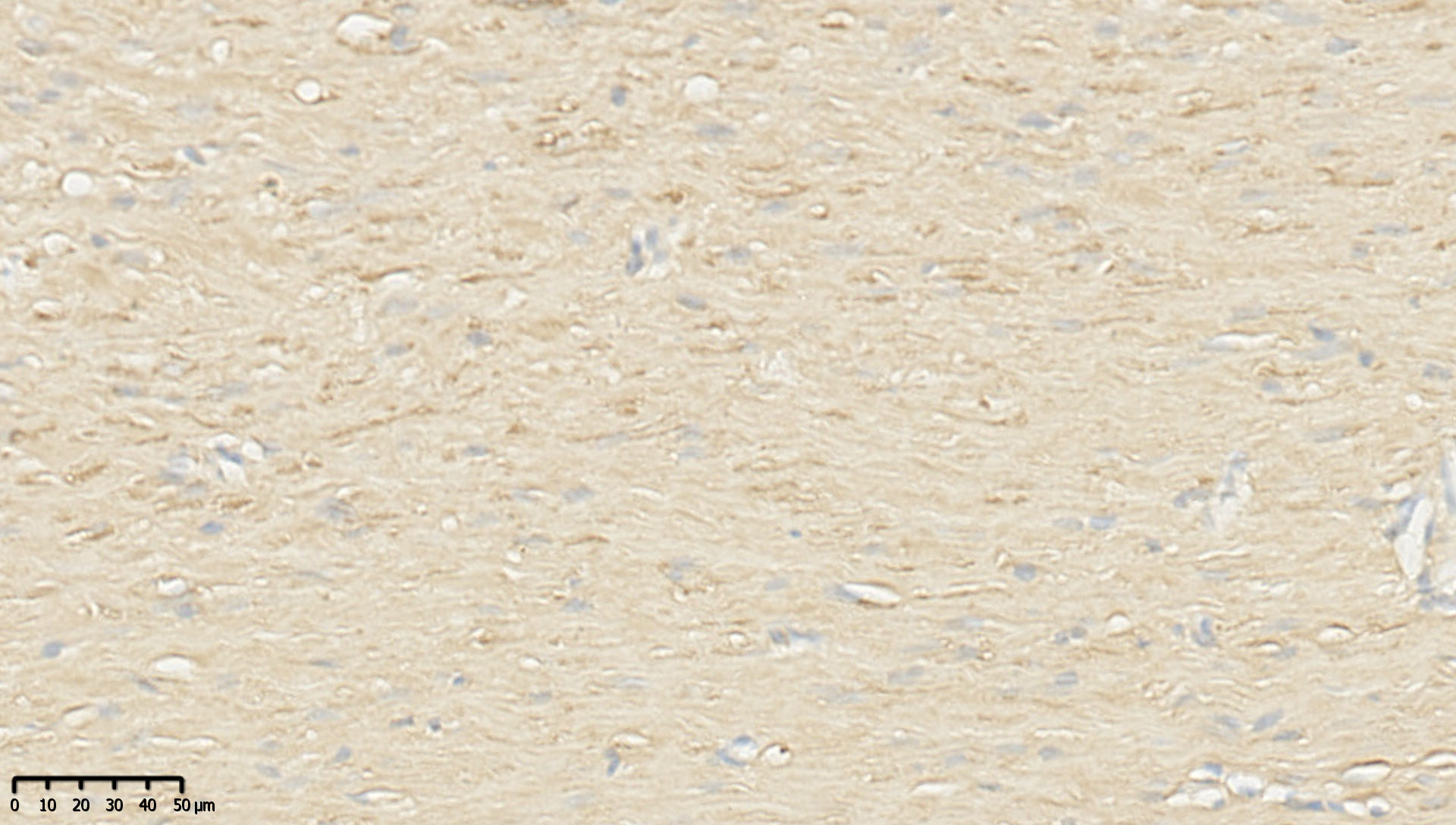

Supplement: S1 File — (ZIP) [file pone.0324264.s001.zip › supplement.material-1/Immunohistochemistry image/COL1/PL-HA-14.jpg]

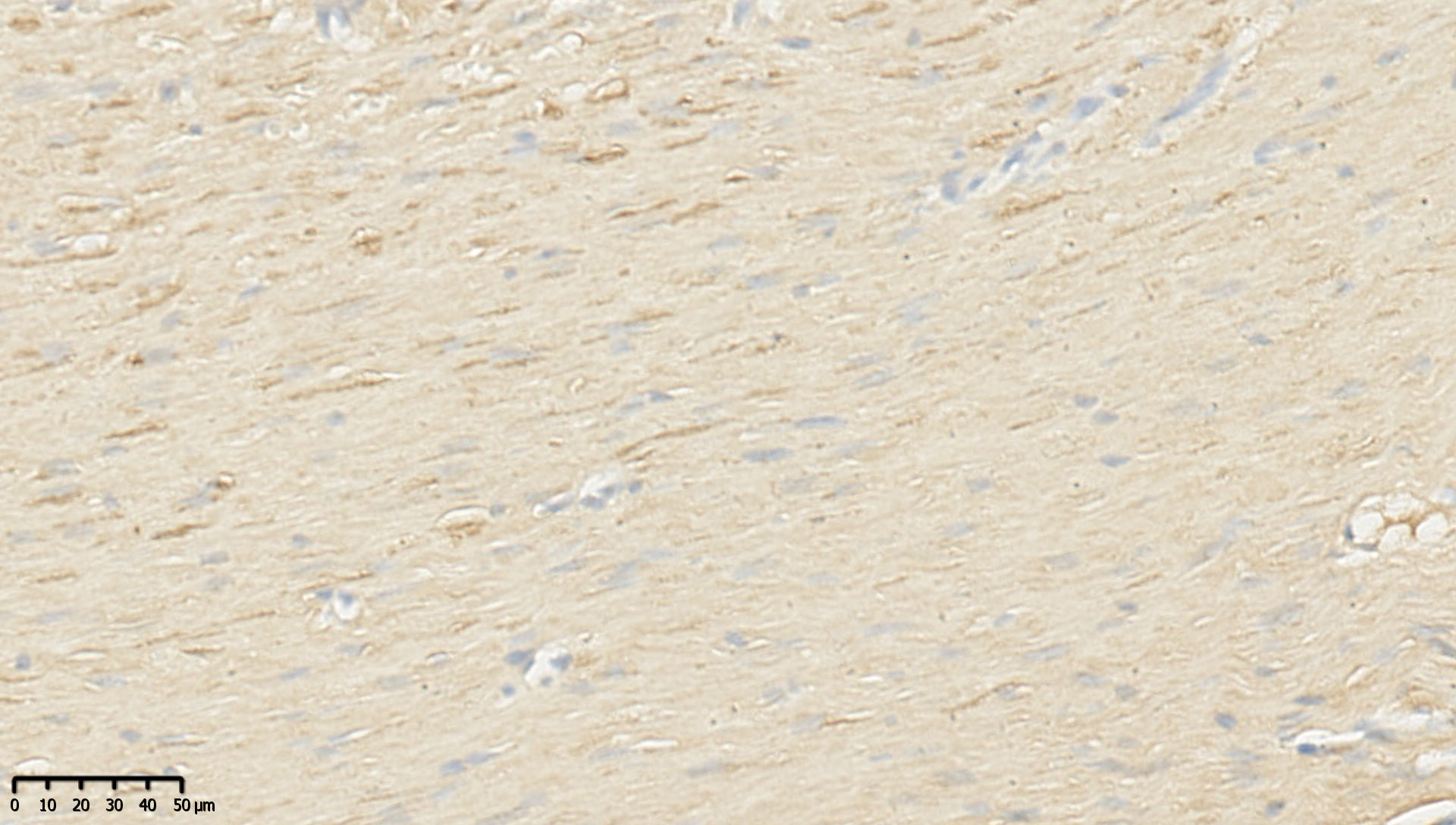

Supplement: S1 File — (ZIP) [file pone.0324264.s001.zip › supplement.material-1/Immunohistochemistry image/COL1/PL-HA-15.jpg]

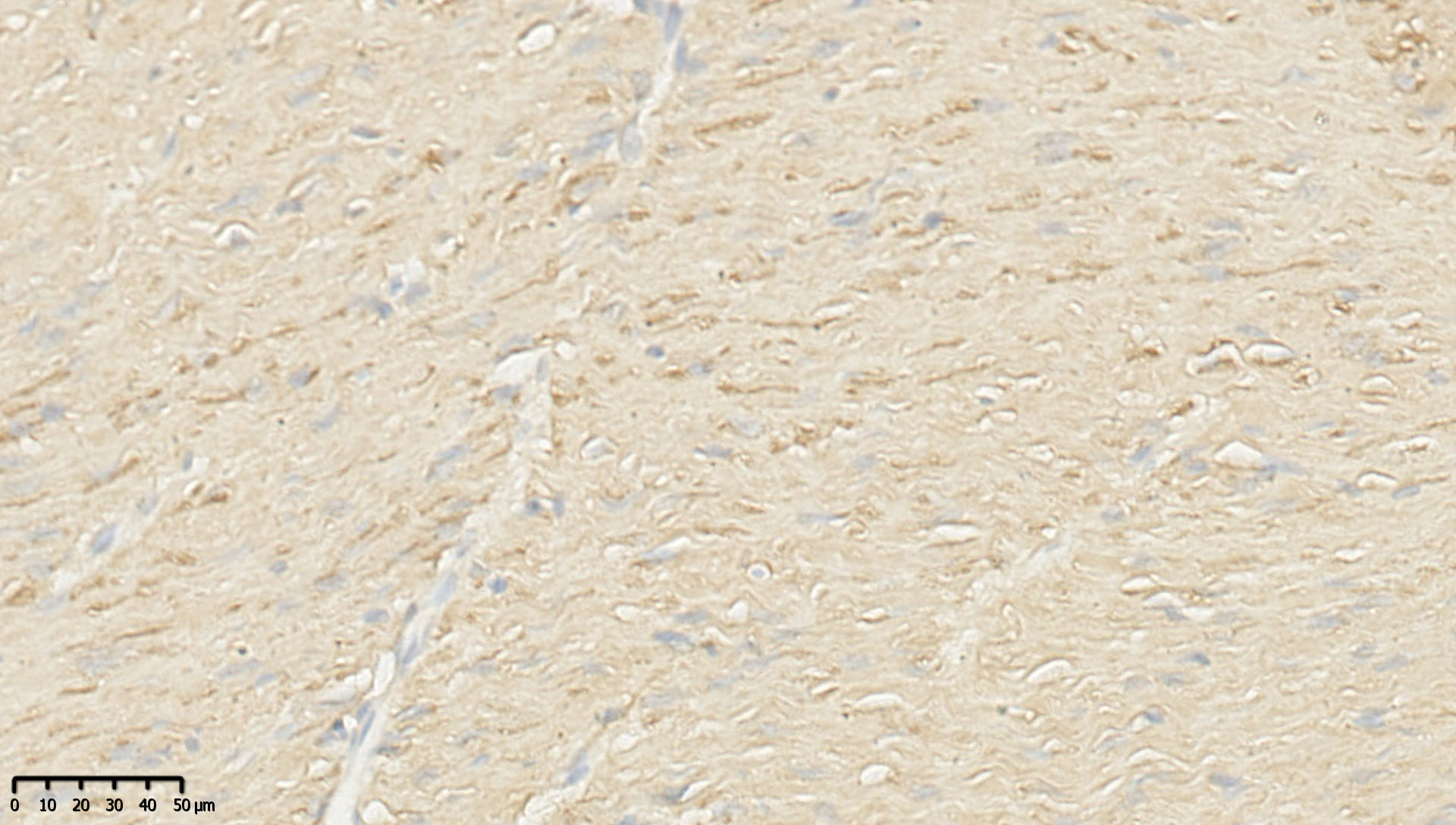

Supplement: S1 File — (ZIP) [file pone.0324264.s001.zip › supplement.material-1/Immunohistochemistry image/COL1/PL-HA-16.jpg]

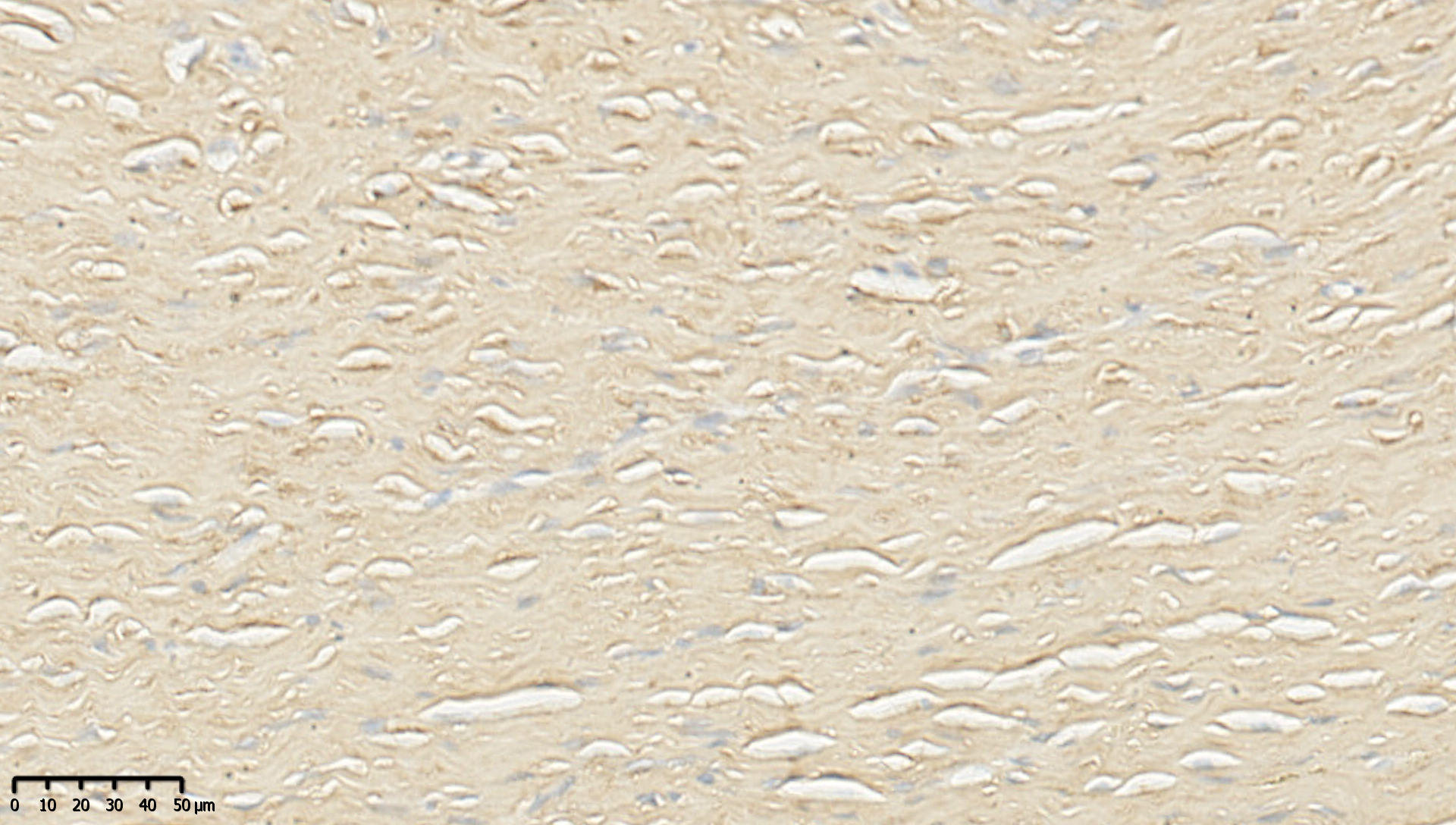

Supplement: S1 File — (ZIP) [file pone.0324264.s001.zip › supplement.material-1/Immunohistochemistry image/COL1/PL-HA-7.jpg]

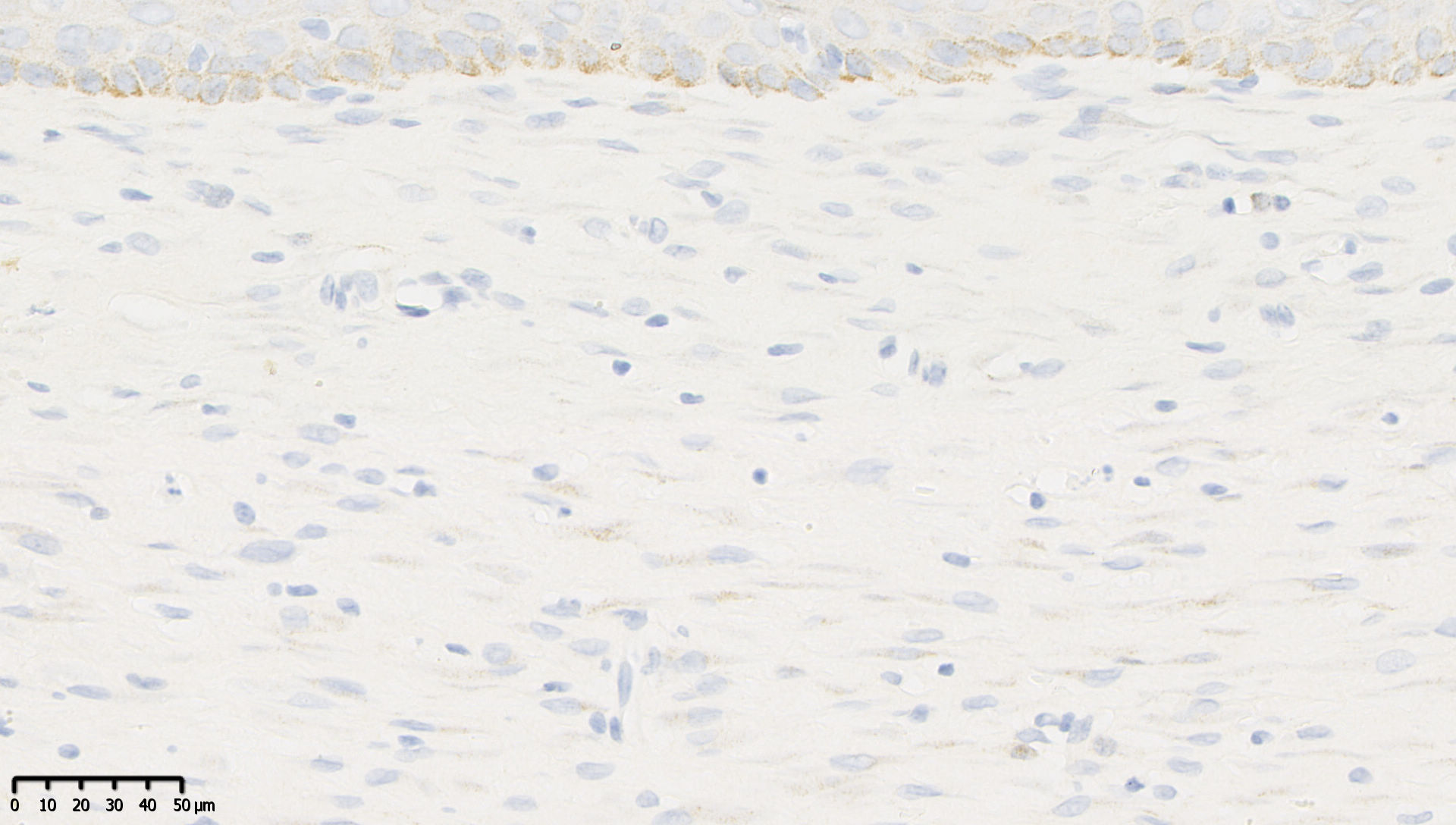

Supplement: S1 File — (ZIP) [file pone.0324264.s001.zip › supplement.material-1/Immunohistochemistry image/KI67/control-111.jpg]

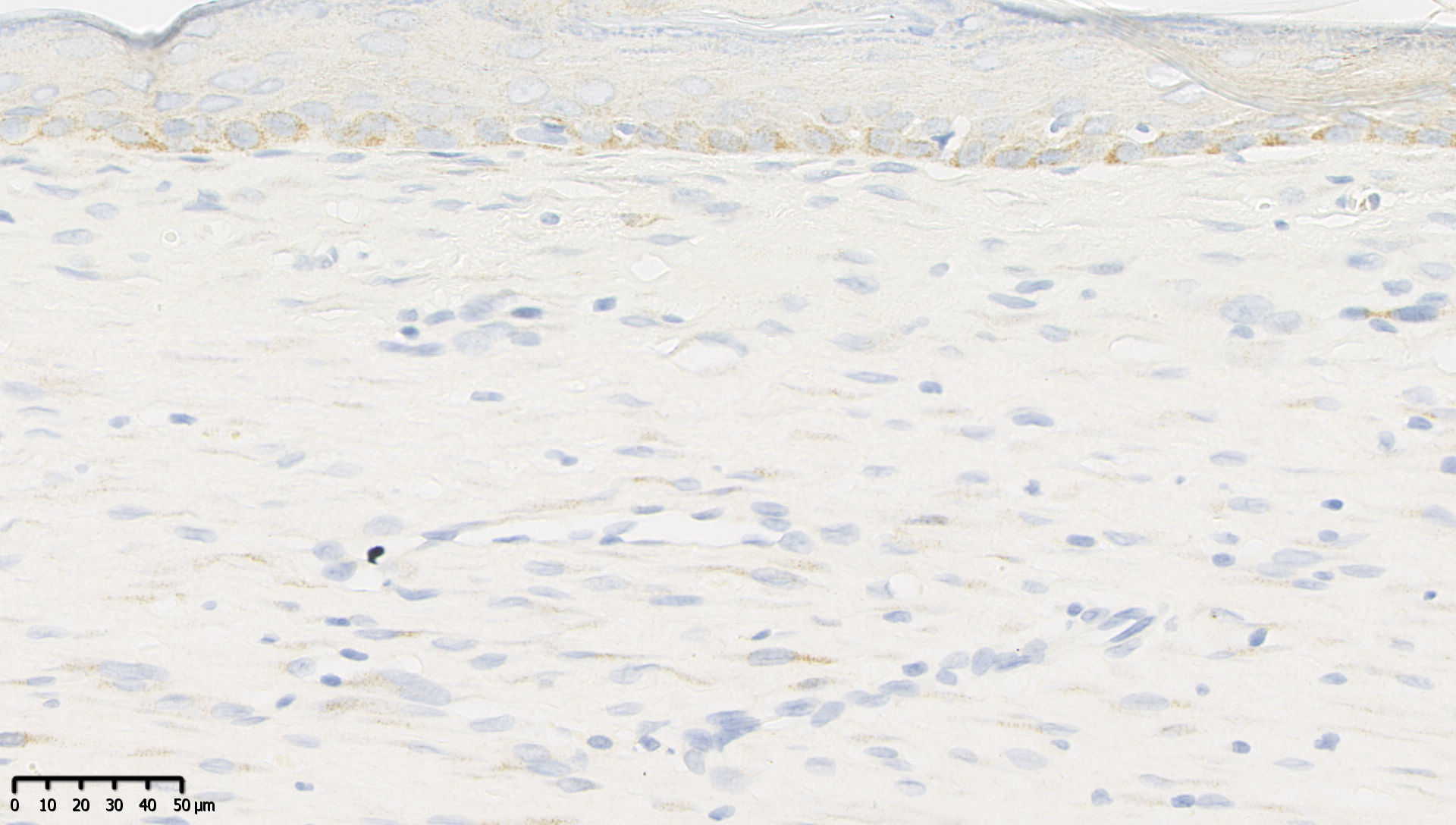

Supplement: S1 File — (ZIP) [file pone.0324264.s001.zip › supplement.material-1/Immunohistochemistry image/KI67/control-112.jpg]

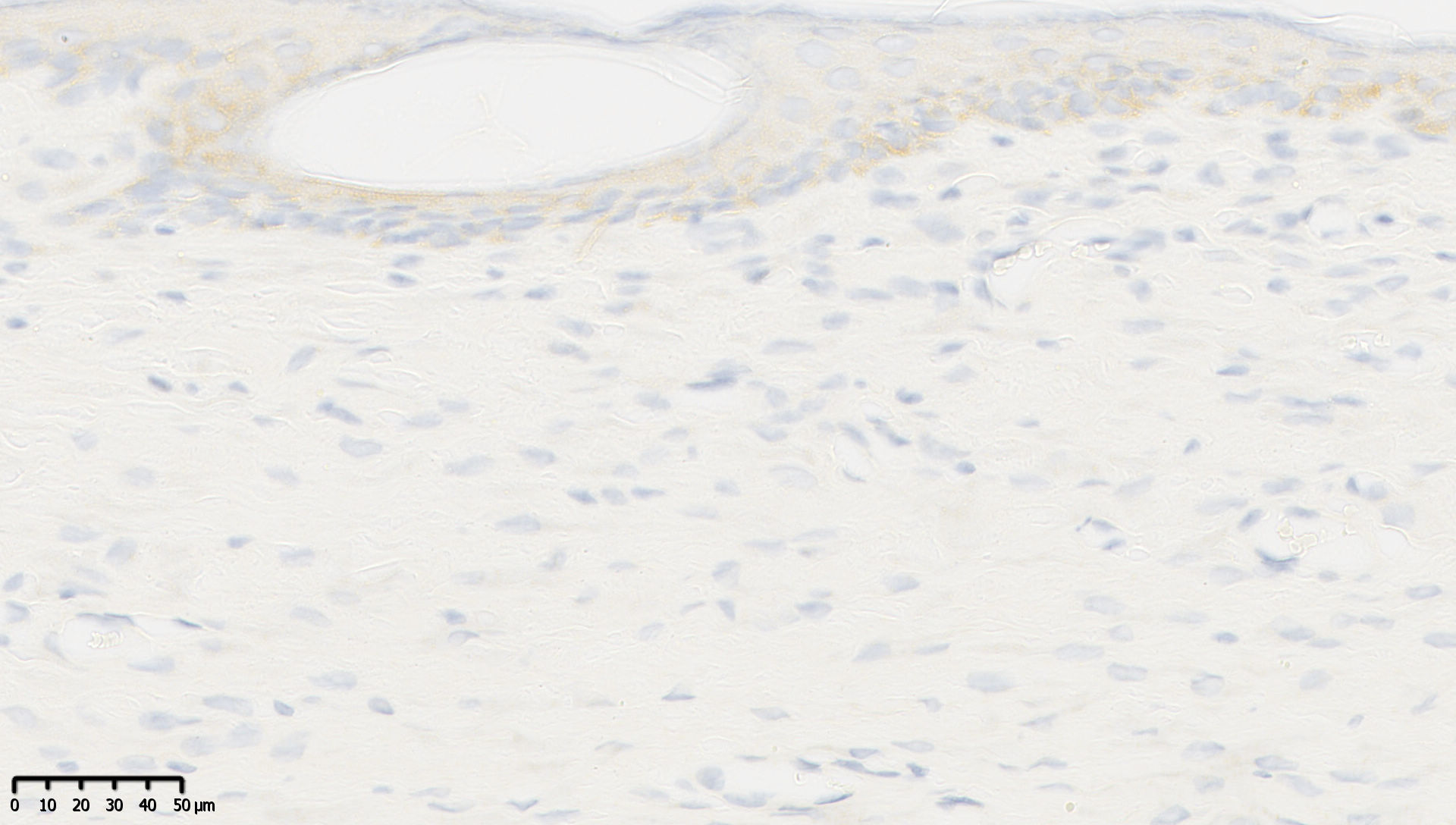

Supplement: S1 File — (ZIP) [file pone.0324264.s001.zip › supplement.material-1/Immunohistochemistry image/KI67/control-113.jpg]

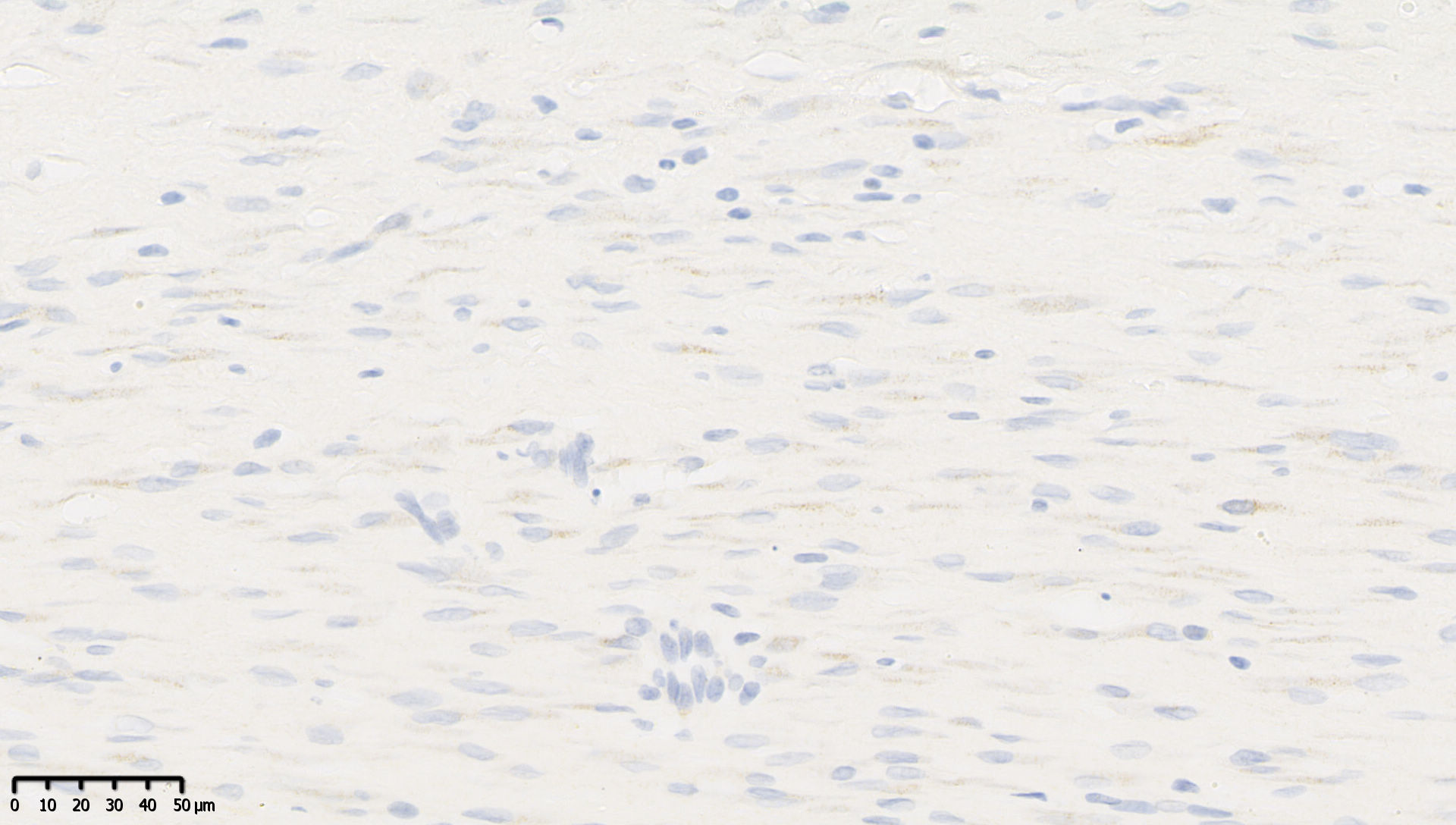

Supplement: S1 File — (ZIP) [file pone.0324264.s001.zip › supplement.material-1/Immunohistochemistry image/KI67/control-114.jpg]

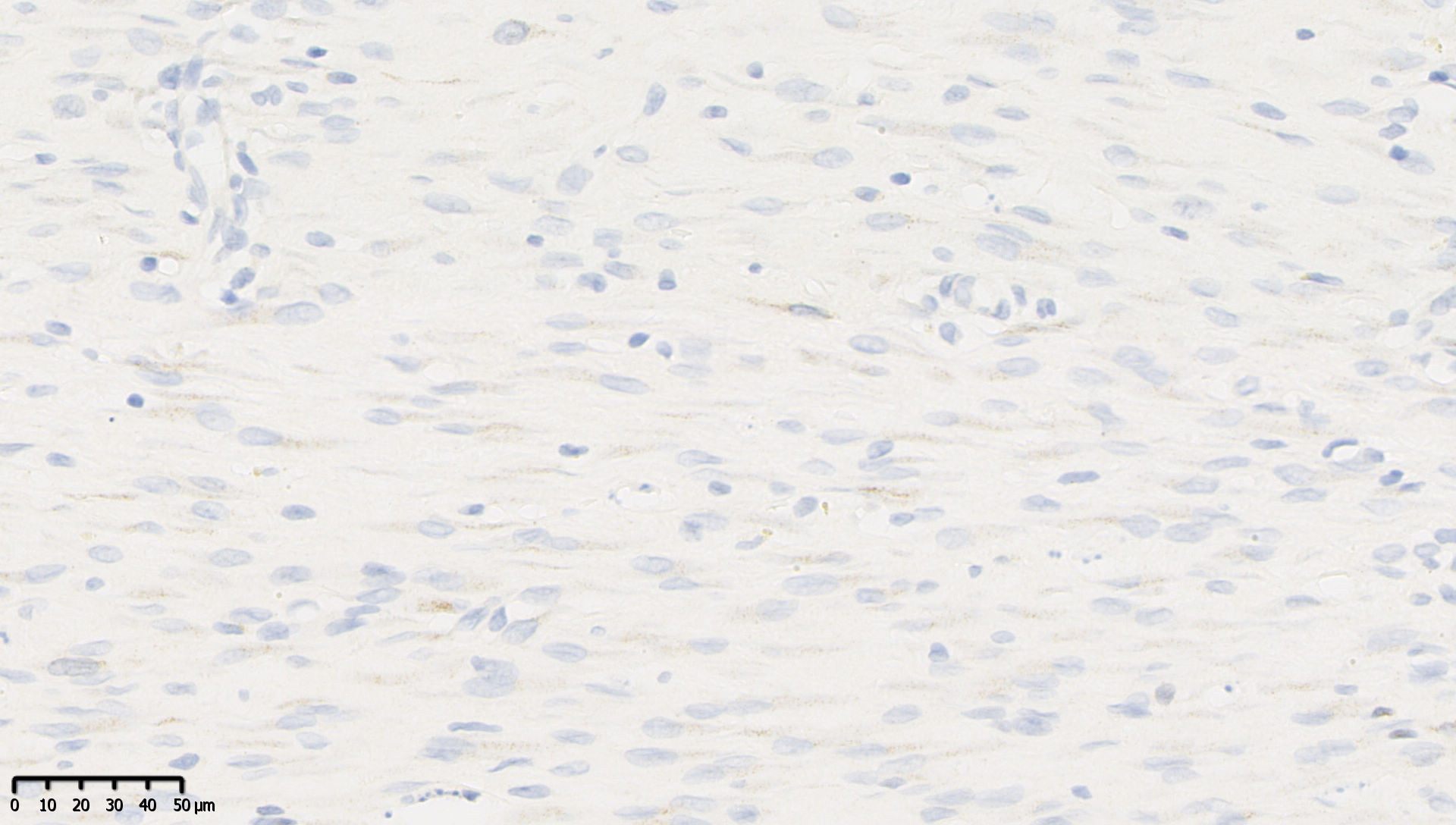

Supplement: S1 File — (ZIP) [file pone.0324264.s001.zip › supplement.material-1/Immunohistochemistry image/KI67/control-115.jpg]

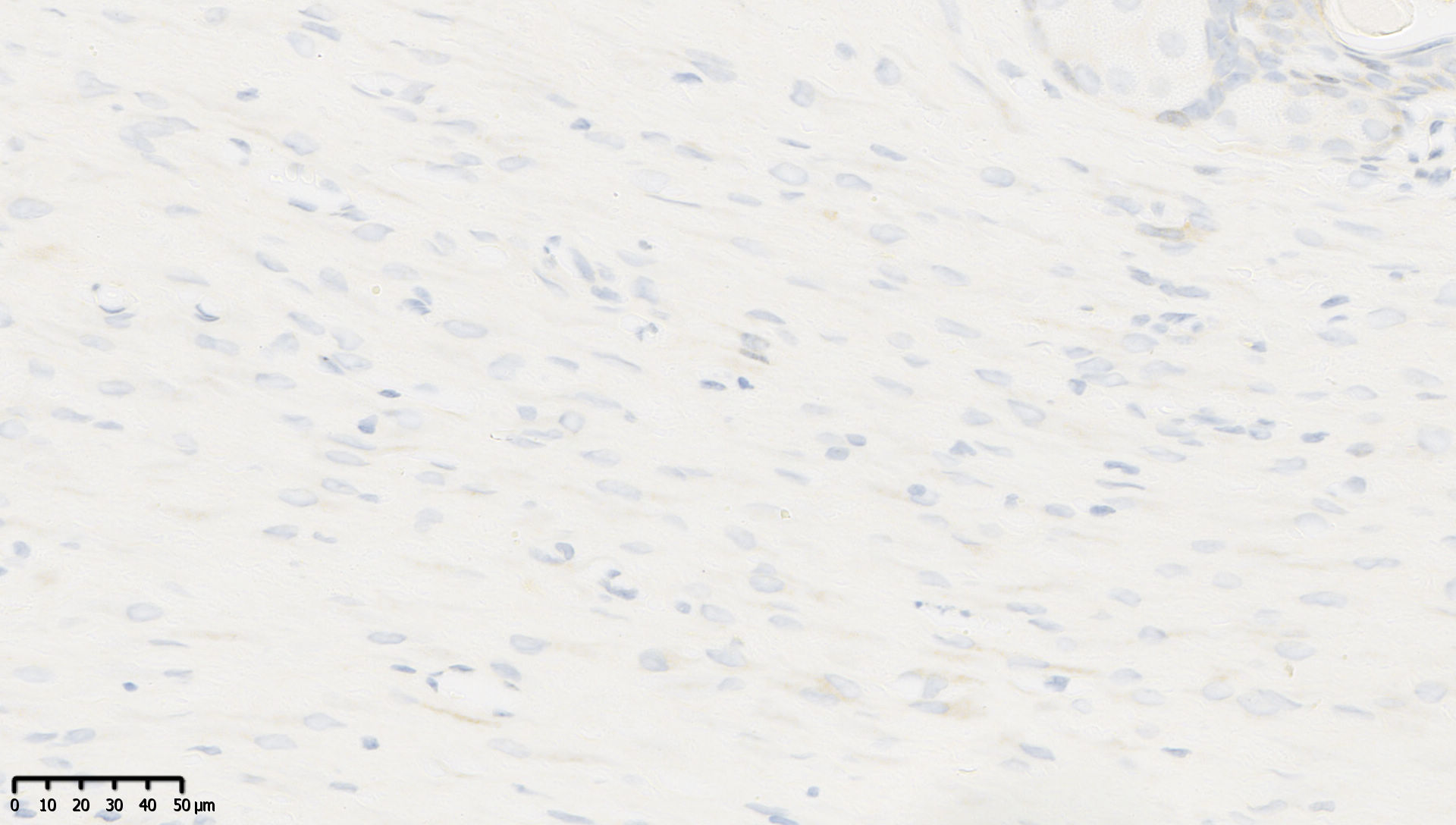

Supplement: S1 File — (ZIP) [file pone.0324264.s001.zip › supplement.material-1/Immunohistochemistry image/KI67/control-116.jpg]

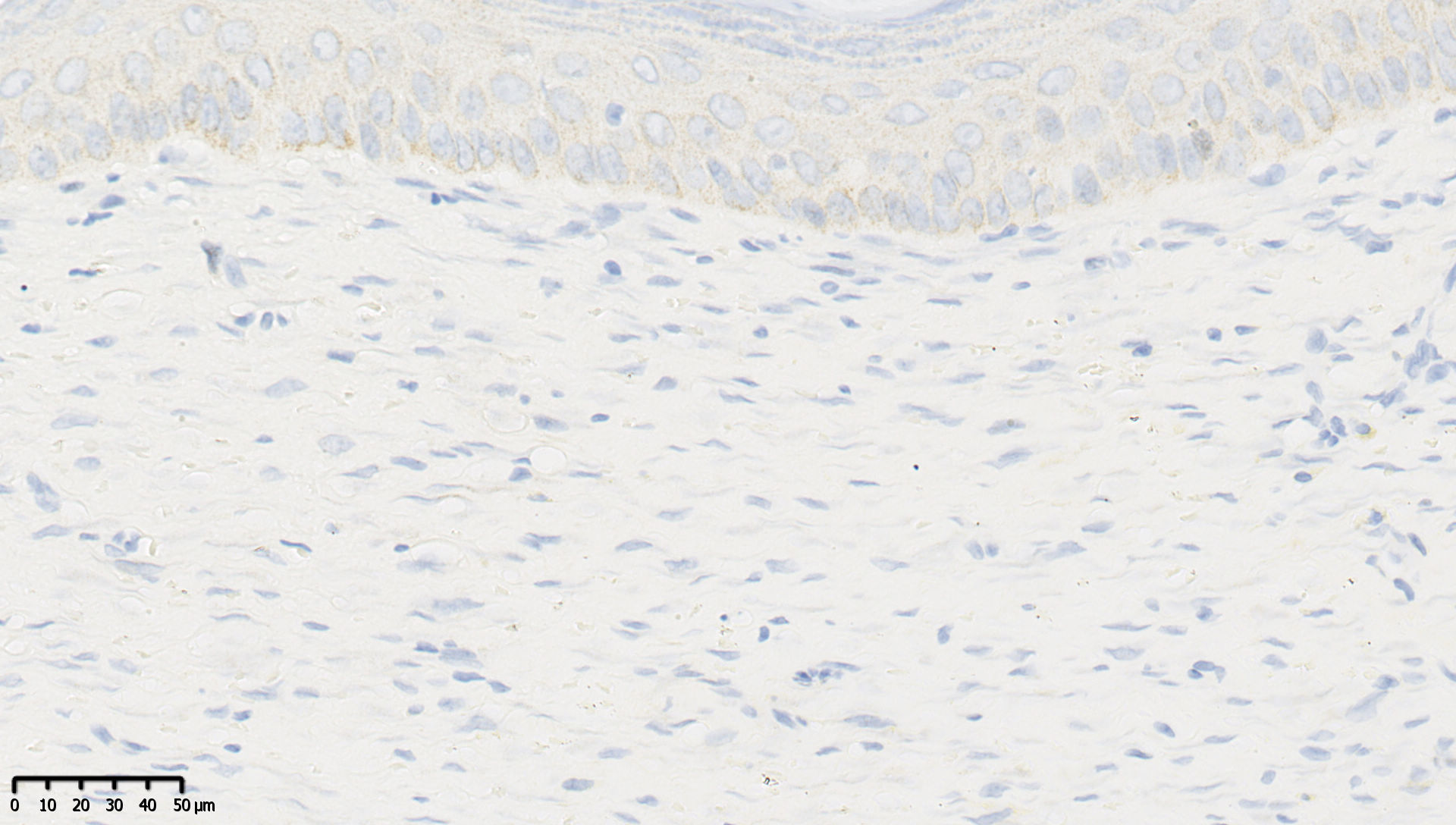

Supplement: S1 File — (ZIP) [file pone.0324264.s001.zip › supplement.material-1/Immunohistochemistry image/KI67/HA-111.jpg]

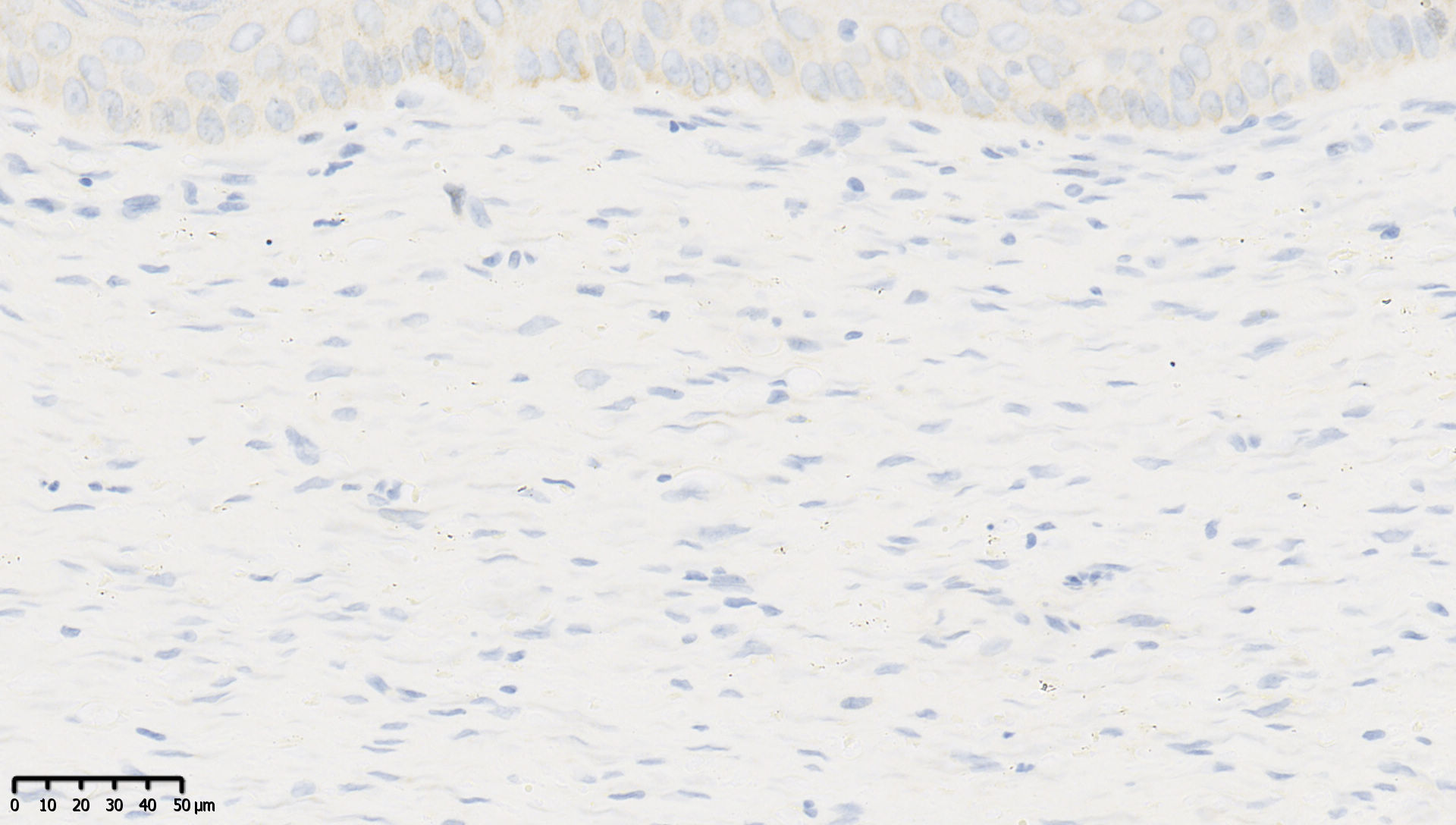

Supplement: S1 File — (ZIP) [file pone.0324264.s001.zip › supplement.material-1/Immunohistochemistry image/KI67/HA-112.jpg]

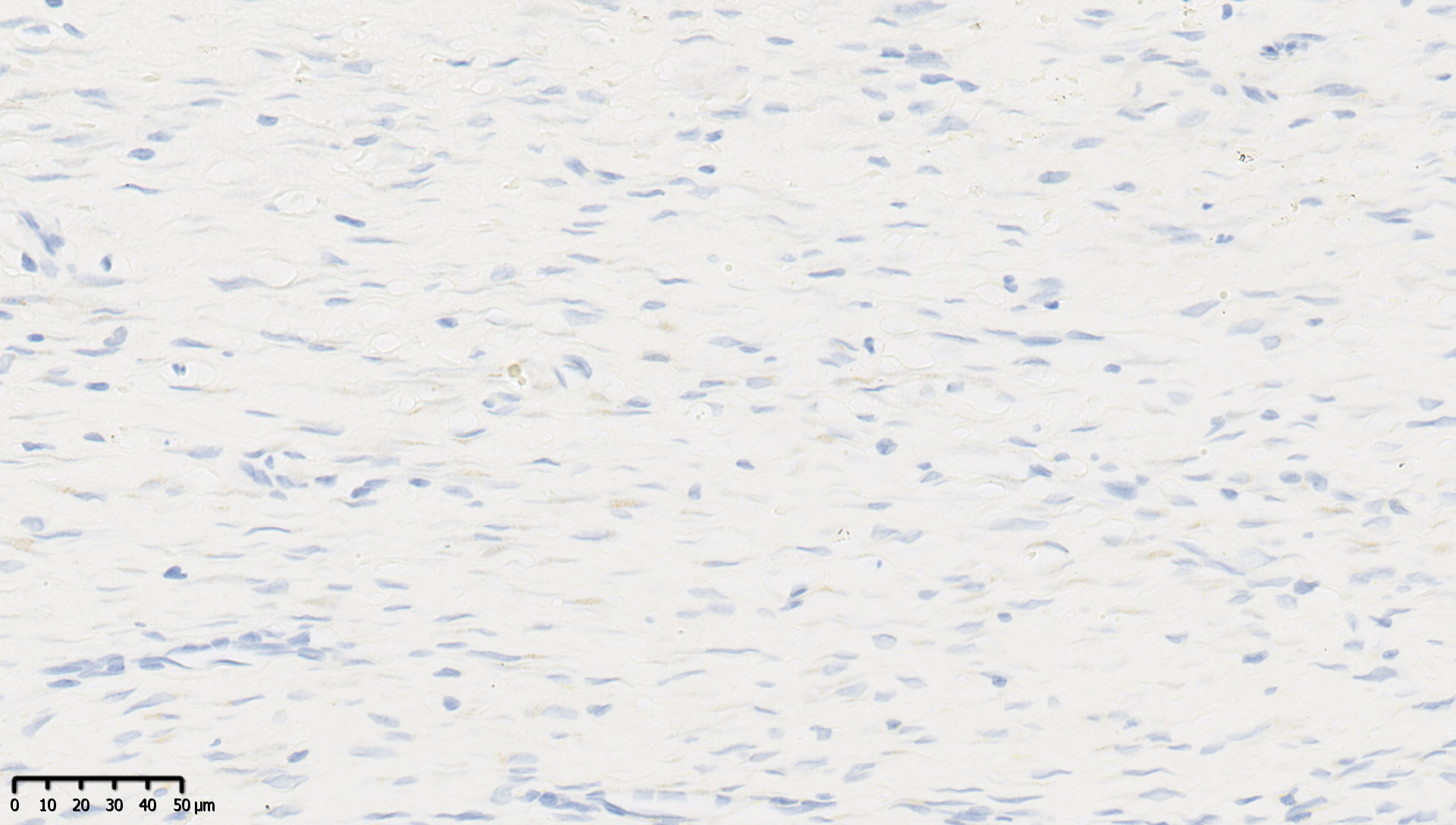

Supplement: S1 File — (ZIP) [file pone.0324264.s001.zip › supplement.material-1/Immunohistochemistry image/KI67/HA-113.jpg]

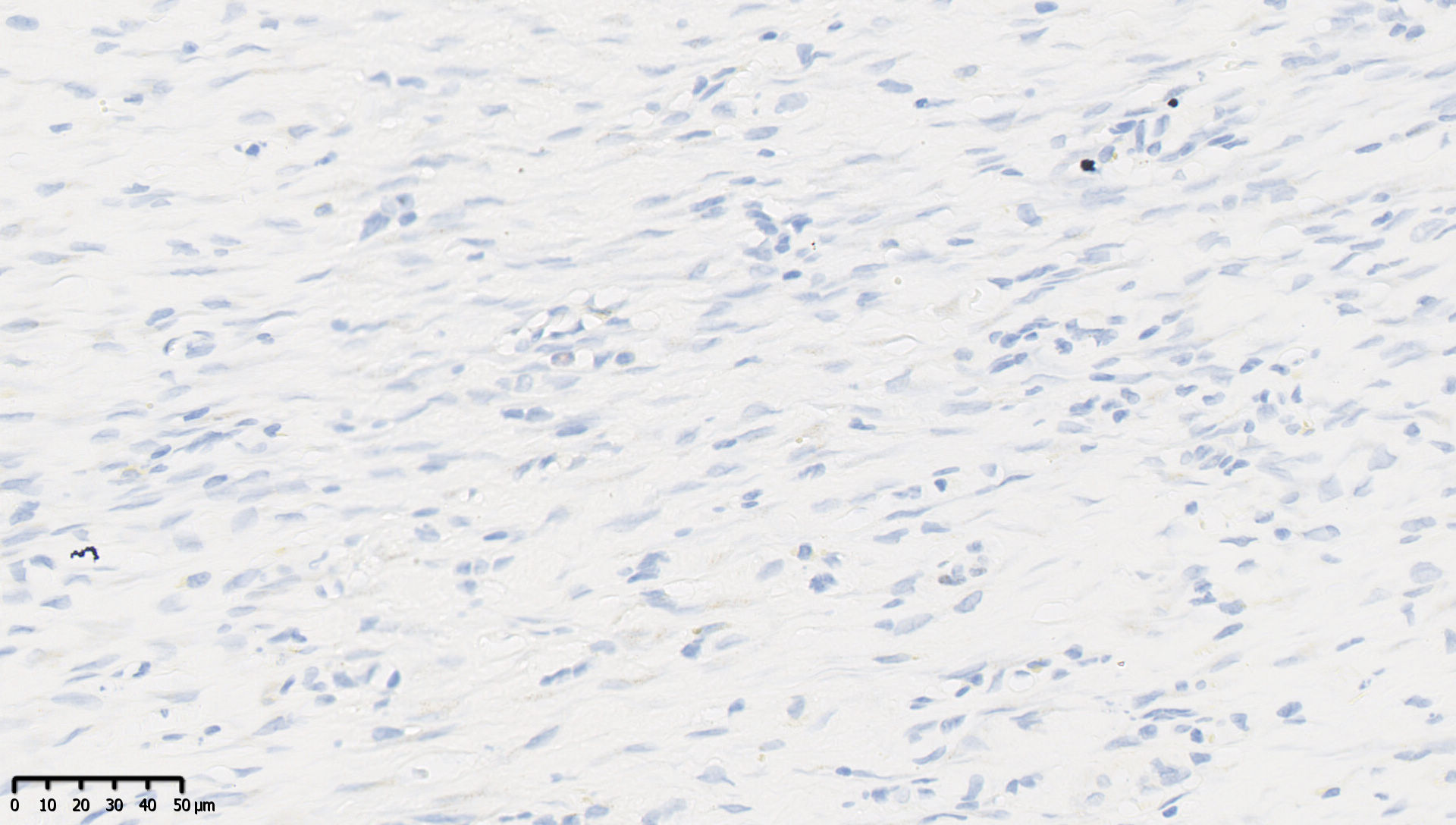

Supplement: S1 File — (ZIP) [file pone.0324264.s001.zip › supplement.material-1/Immunohistochemistry image/KI67/HA-114.jpg]

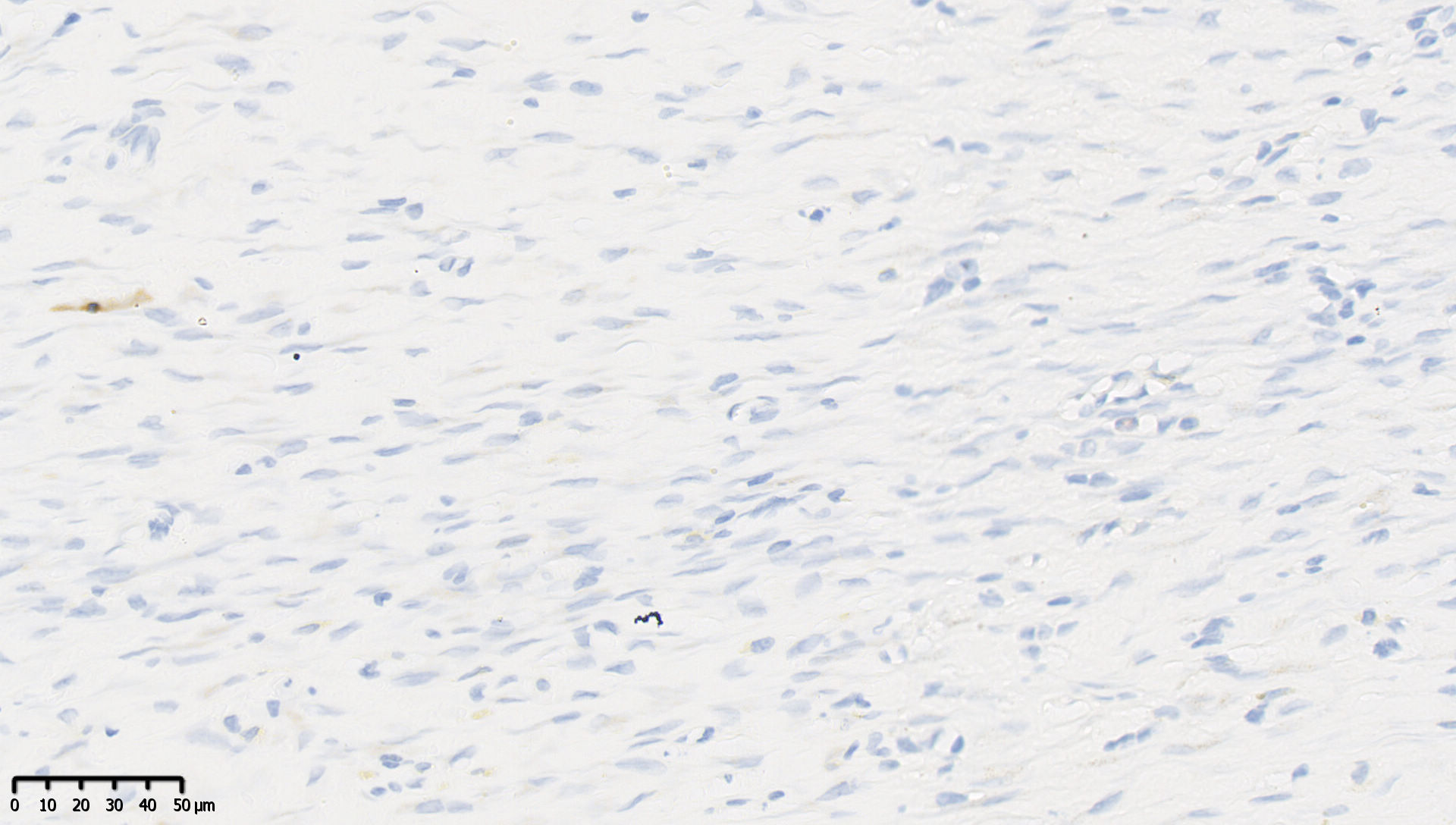

Supplement: S1 File — (ZIP) [file pone.0324264.s001.zip › supplement.material-1/Immunohistochemistry image/KI67/HA-115.jpg]

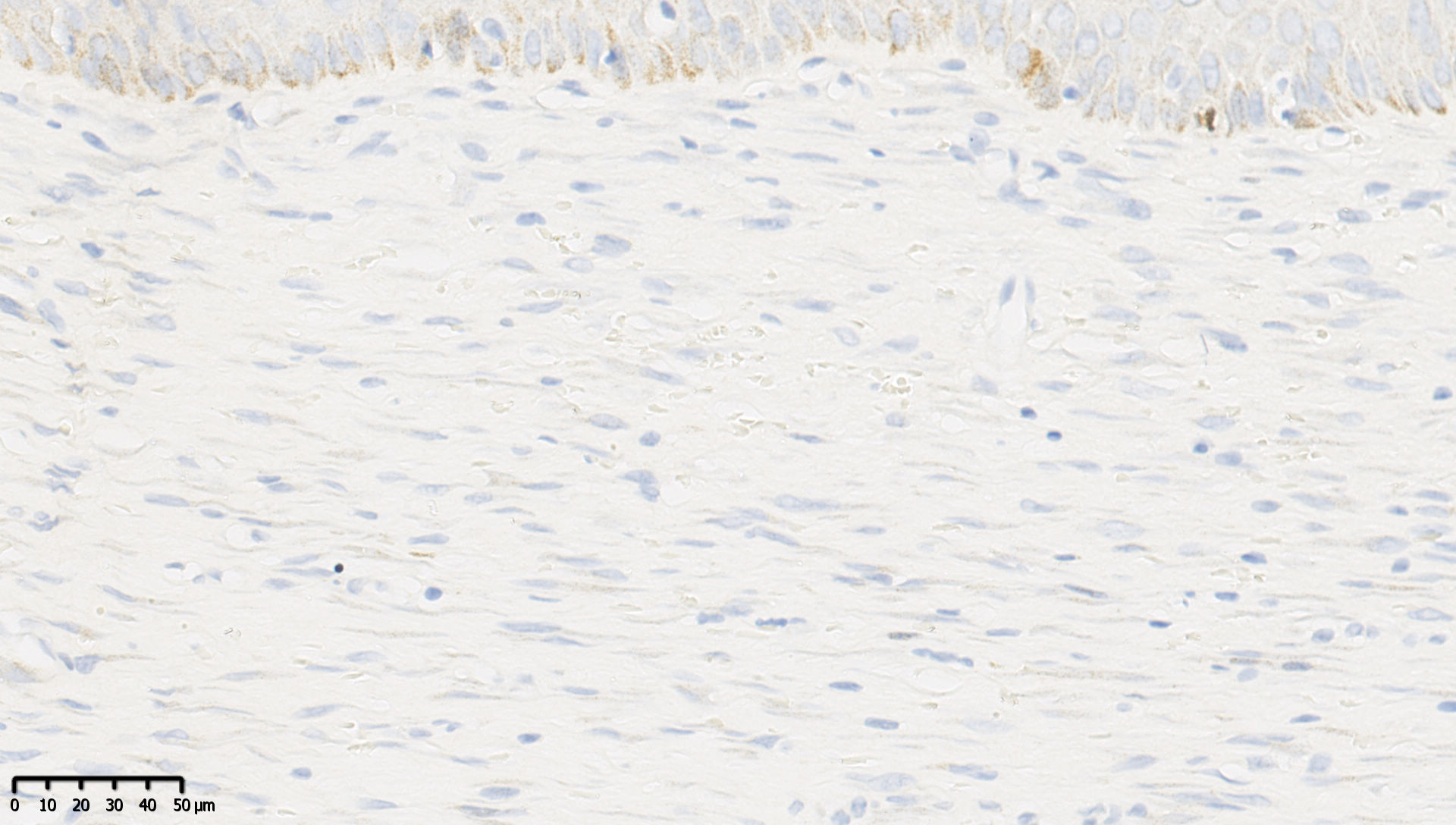

Supplement: S1 File — (ZIP) [file pone.0324264.s001.zip › supplement.material-1/Immunohistochemistry image/KI67/HA-116.jpg]

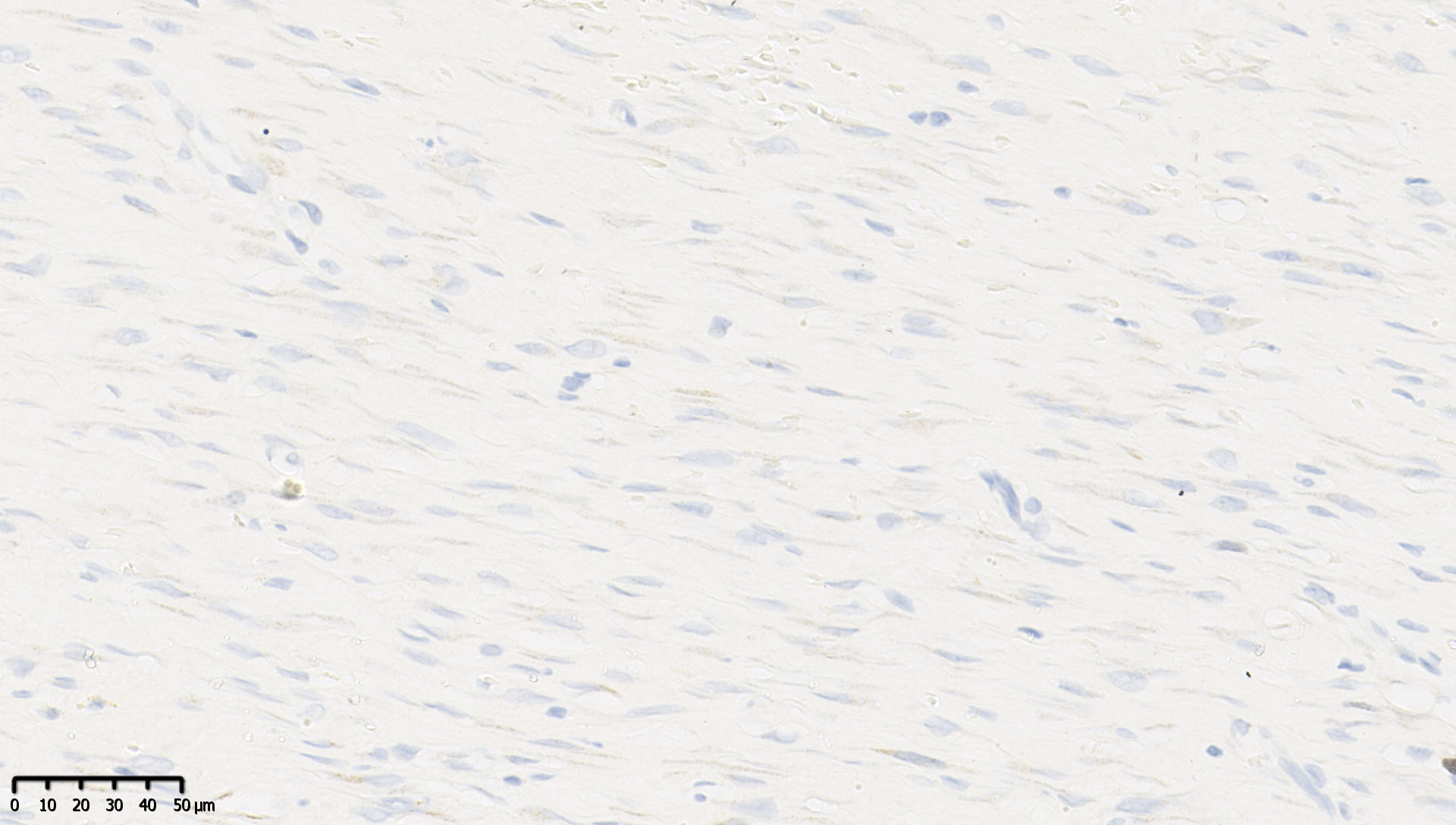

Supplement: S1 File — (ZIP) [file pone.0324264.s001.zip › supplement.material-1/Immunohistochemistry image/KI67/HA-117.jpg]

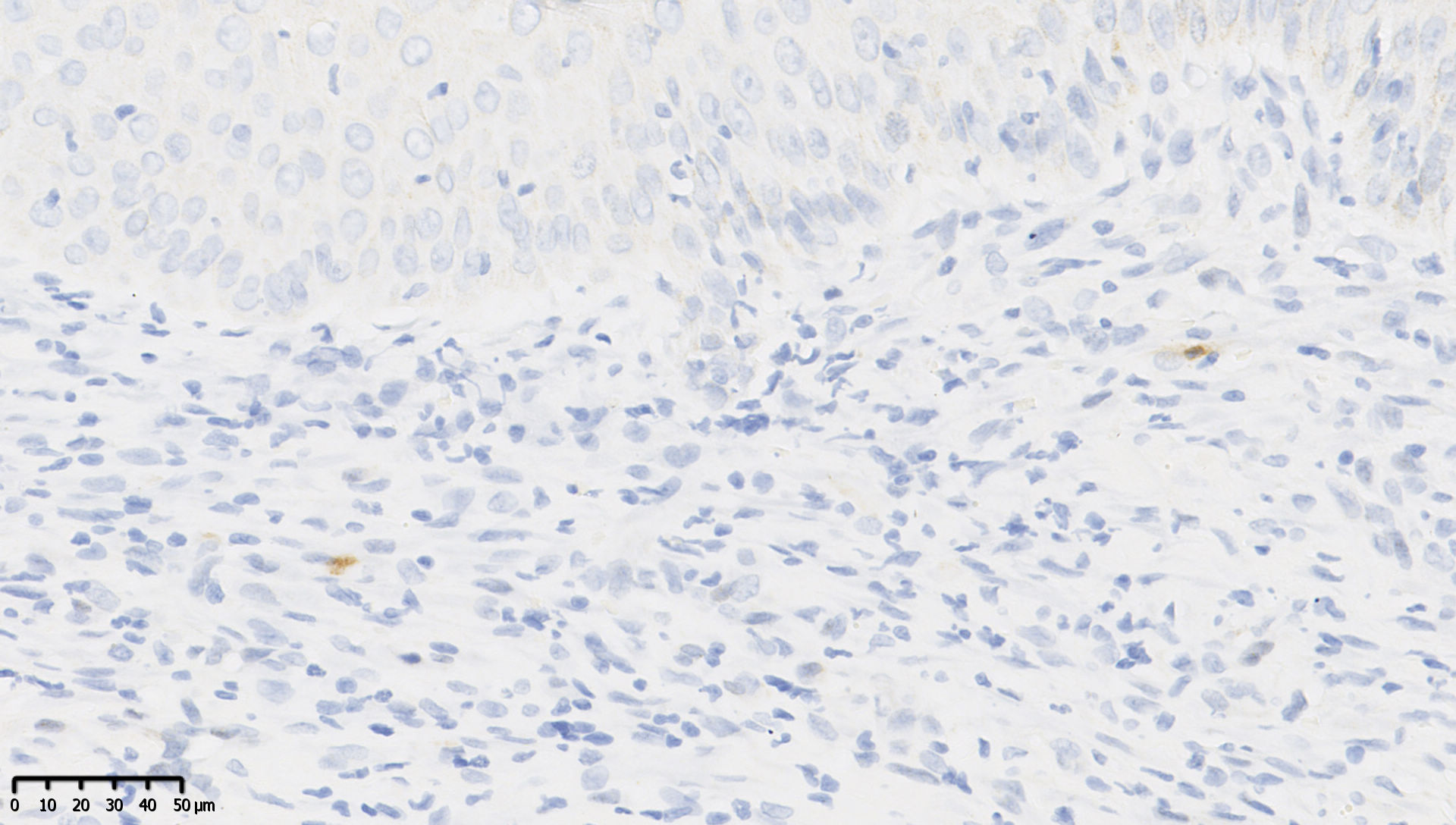

Supplement: S1 File — (ZIP) [file pone.0324264.s001.zip › supplement.material-1/Immunohistochemistry image/KI67/model-111.jpg]

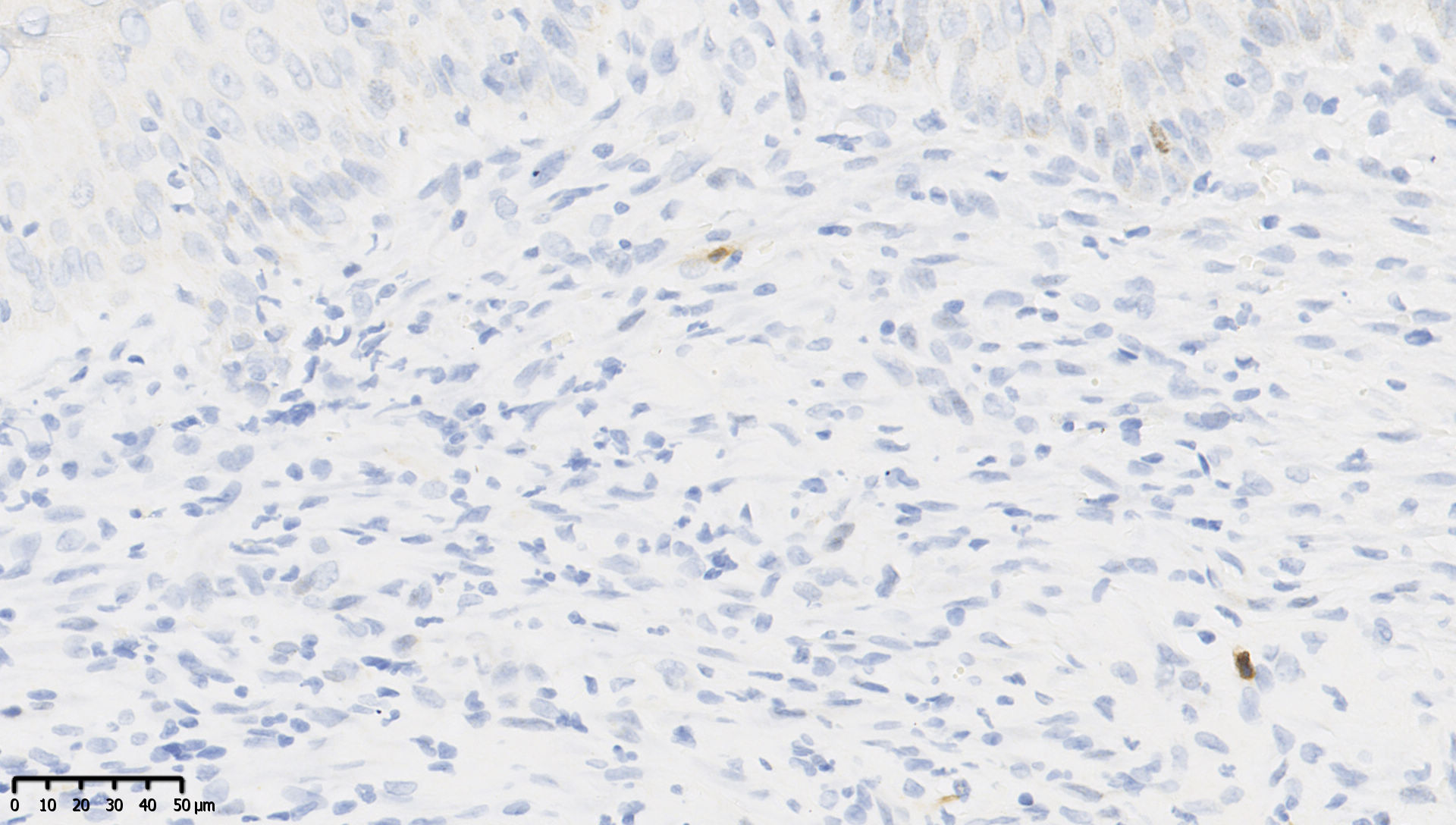

Supplement: S1 File — (ZIP) [file pone.0324264.s001.zip › supplement.material-1/Immunohistochemistry image/KI67/model-112.jpg]

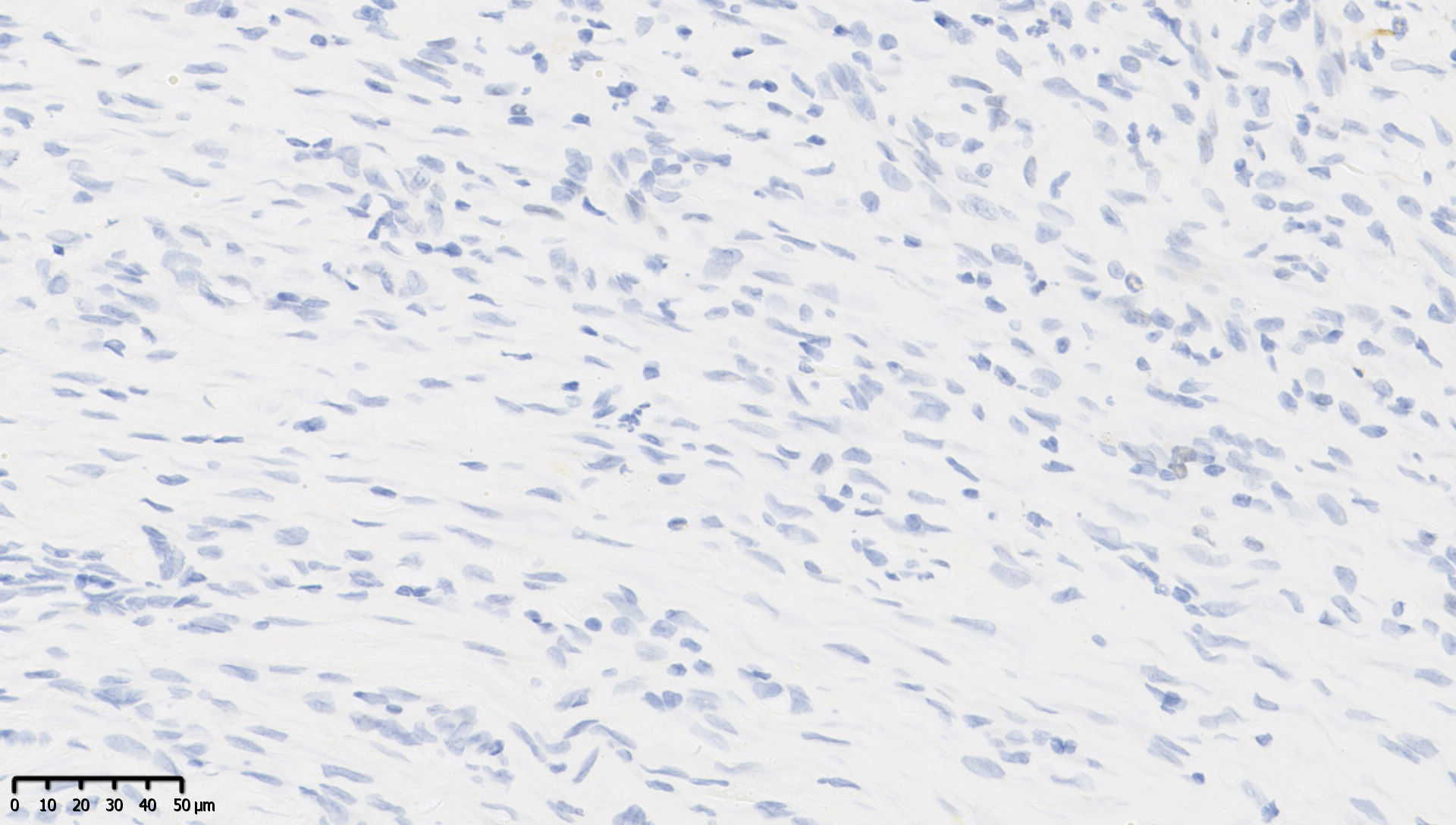

Supplement: S1 File — (ZIP) [file pone.0324264.s001.zip › supplement.material-1/Immunohistochemistry image/KI67/model-113.jpg]

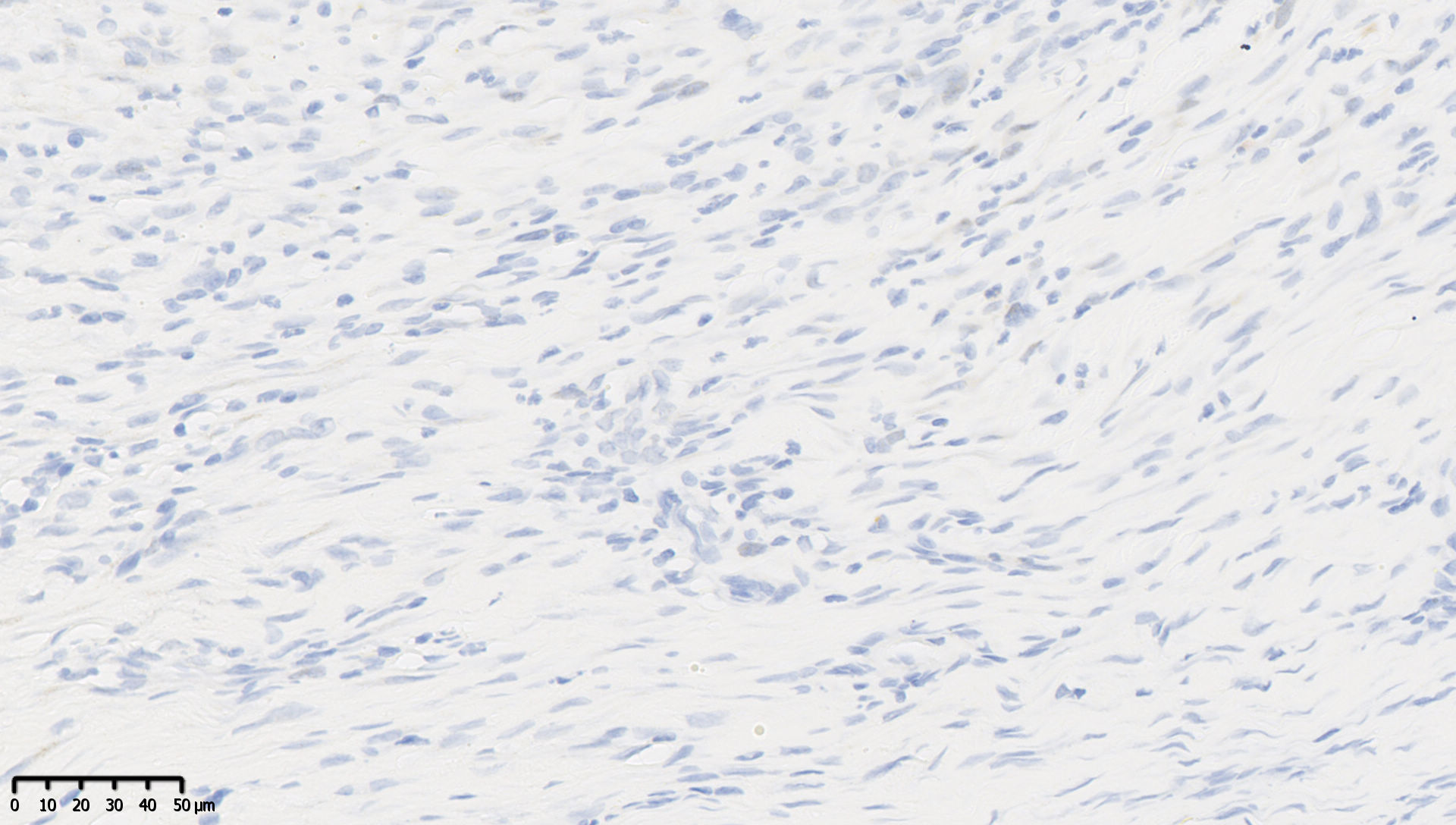

Supplement: S1 File — (ZIP) [file pone.0324264.s001.zip › supplement.material-1/Immunohistochemistry image/KI67/model-114.jpg]

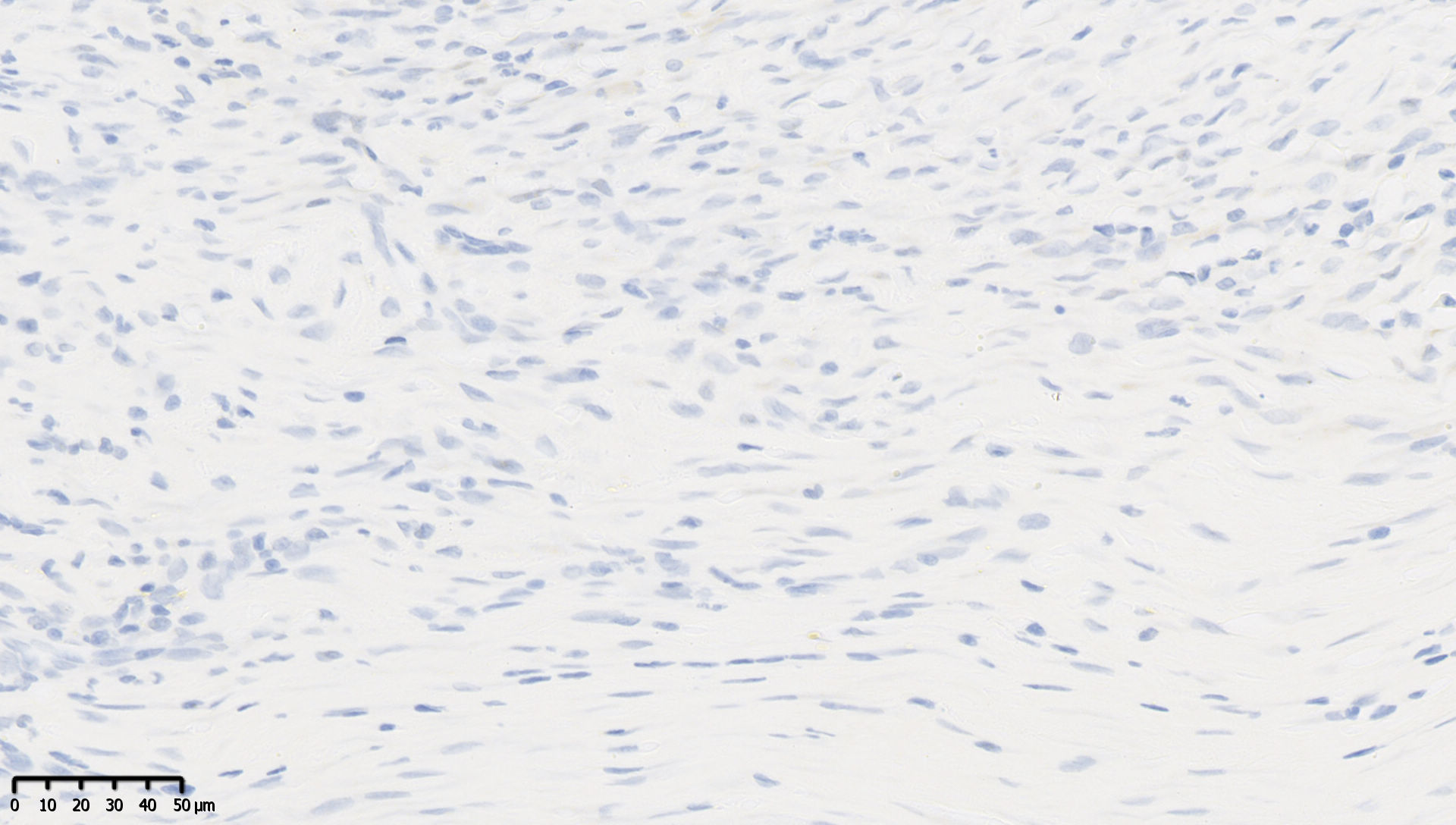

Supplement: S1 File — (ZIP) [file pone.0324264.s001.zip › supplement.material-1/Immunohistochemistry image/KI67/model-115.jpg]

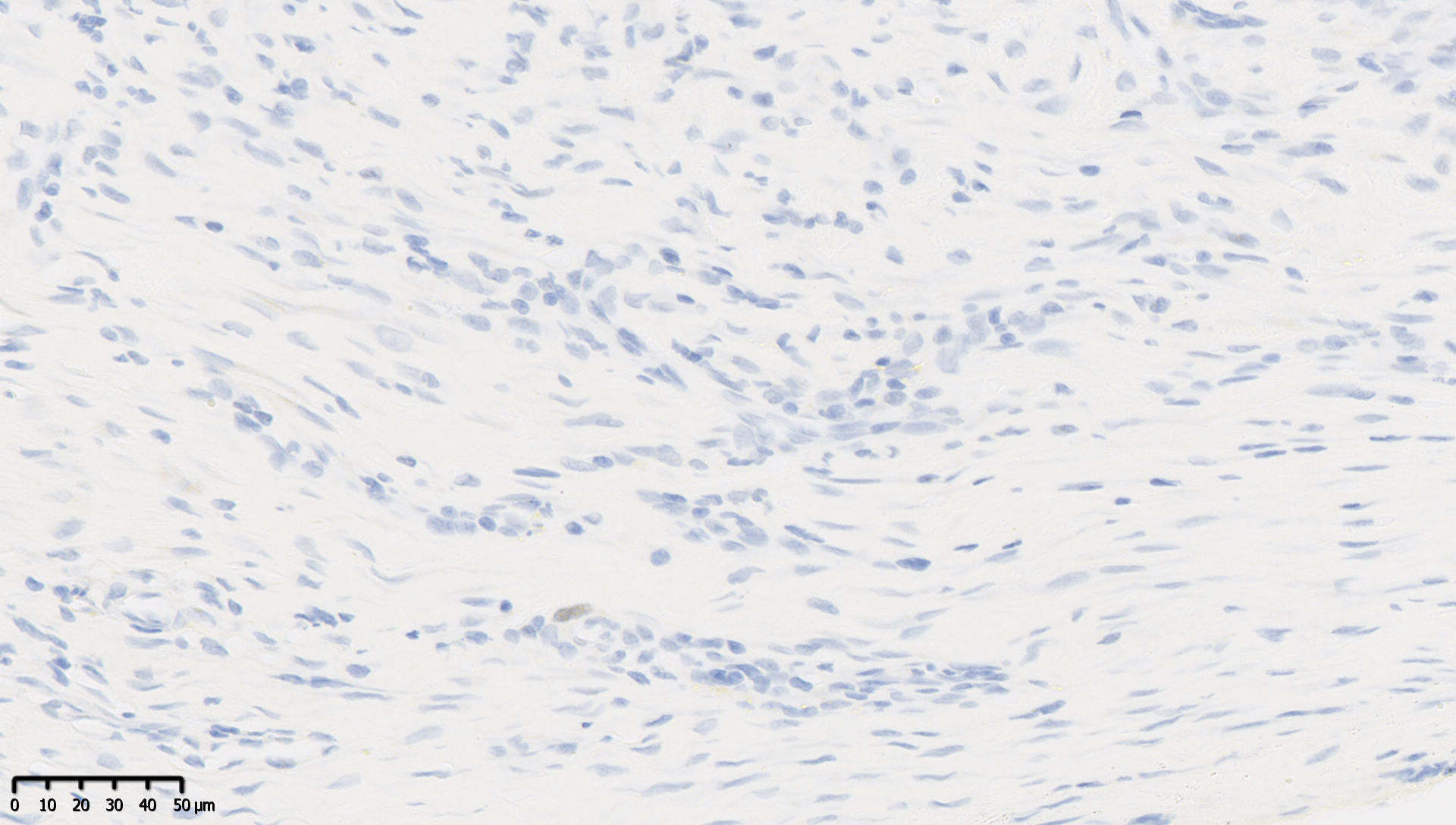

Supplement: S1 File — (ZIP) [file pone.0324264.s001.zip › supplement.material-1/Immunohistochemistry image/KI67/model-116.jpg]

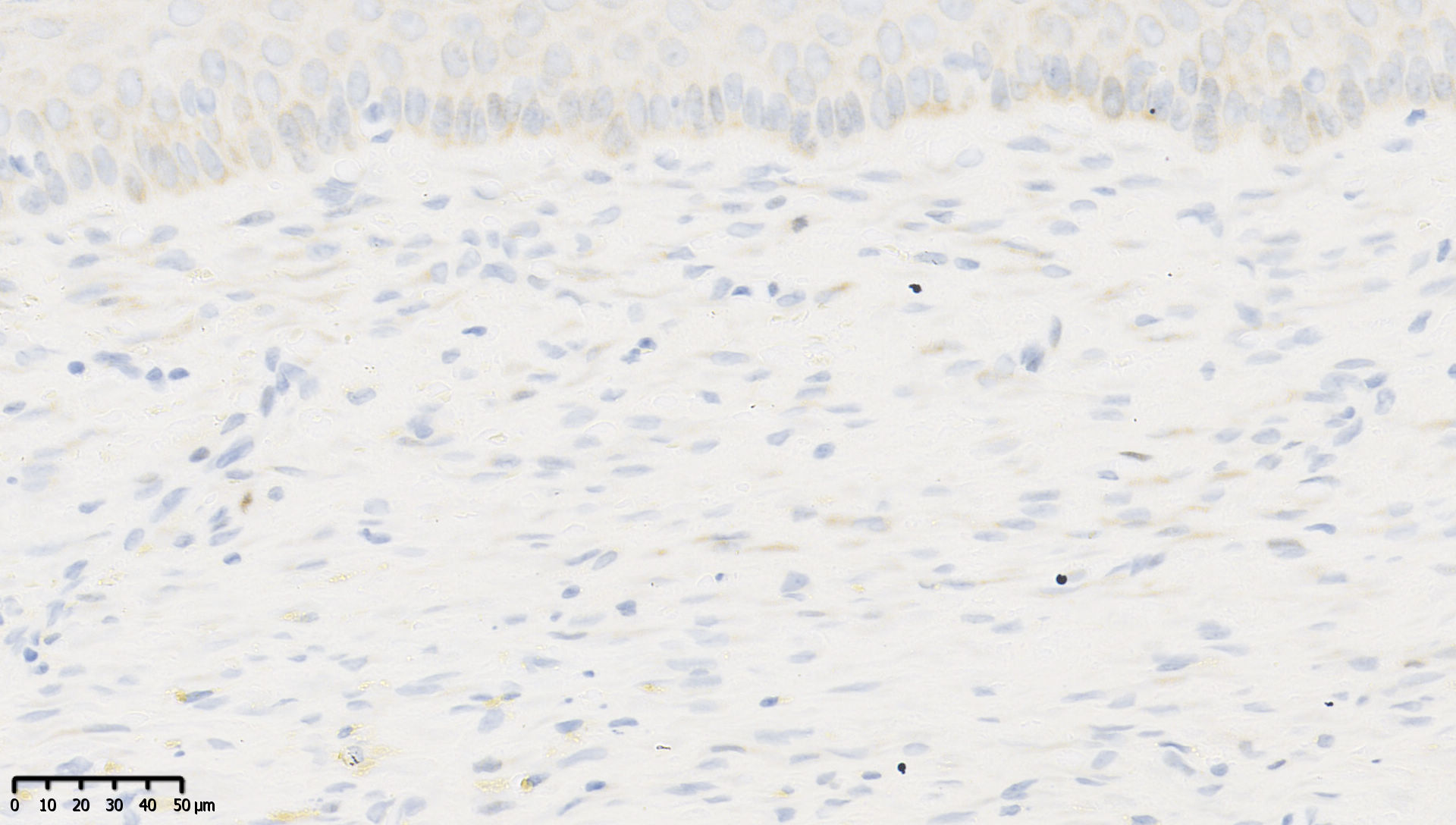

Supplement: S1 File — (ZIP) [file pone.0324264.s001.zip › supplement.material-1/Immunohistochemistry image/KI67/PL-HA-111.jpg]

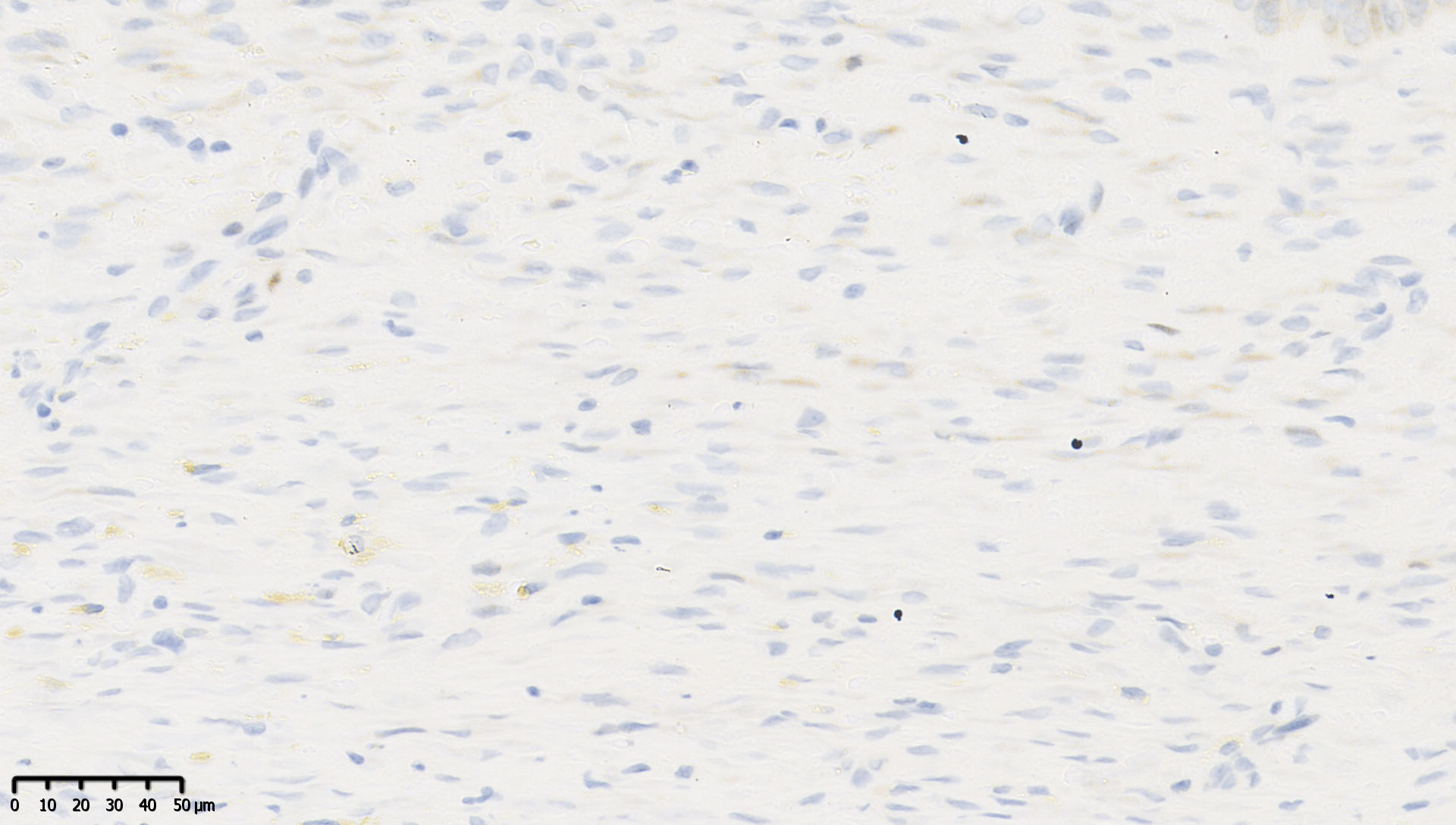

Supplement: S1 File — (ZIP) [file pone.0324264.s001.zip › supplement.material-1/Immunohistochemistry image/KI67/PL-HA-113.jpg]

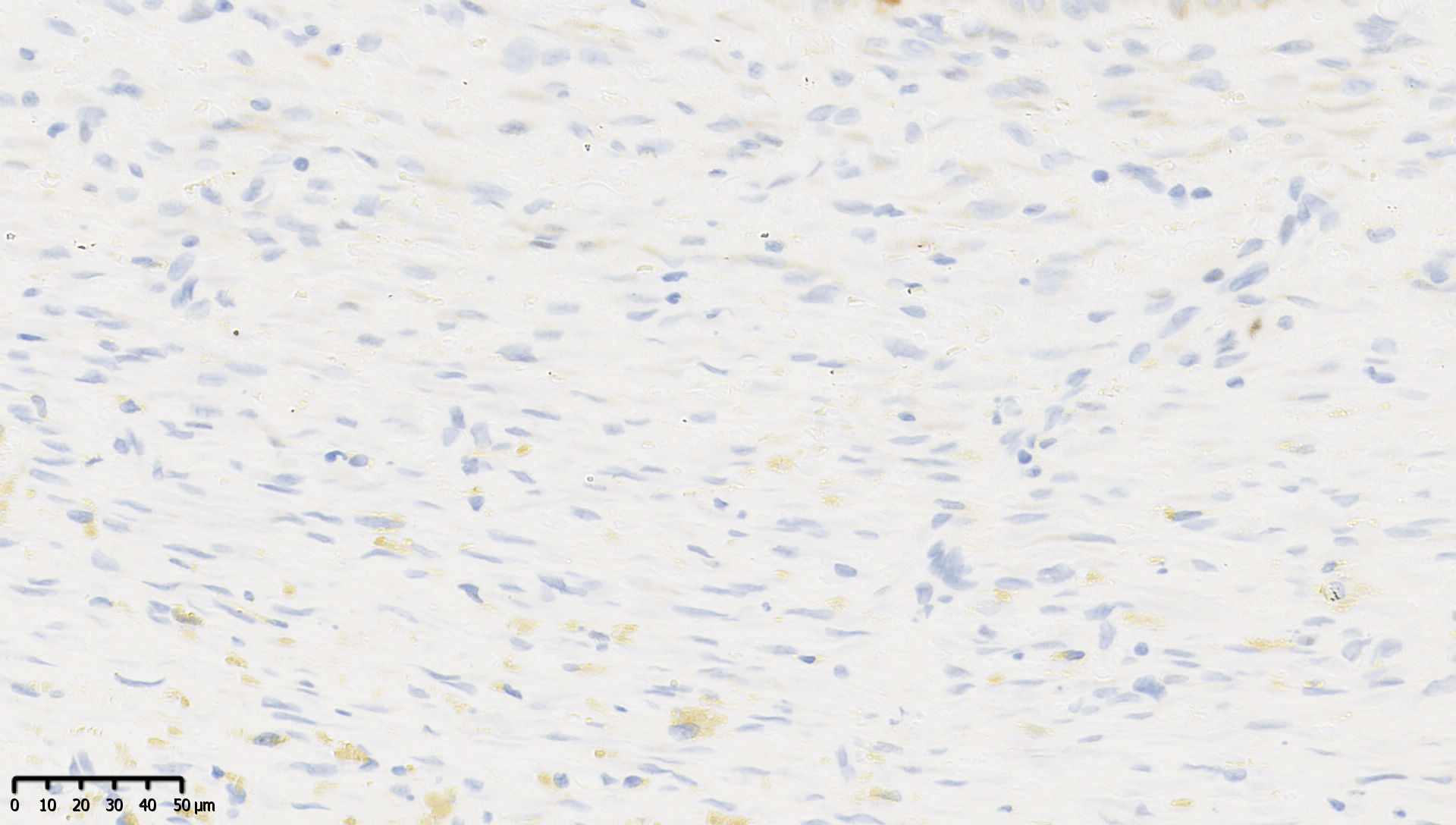

Supplement: S1 File — (ZIP) [file pone.0324264.s001.zip › supplement.material-1/Immunohistochemistry image/KI67/PL-HA-114.jpg]

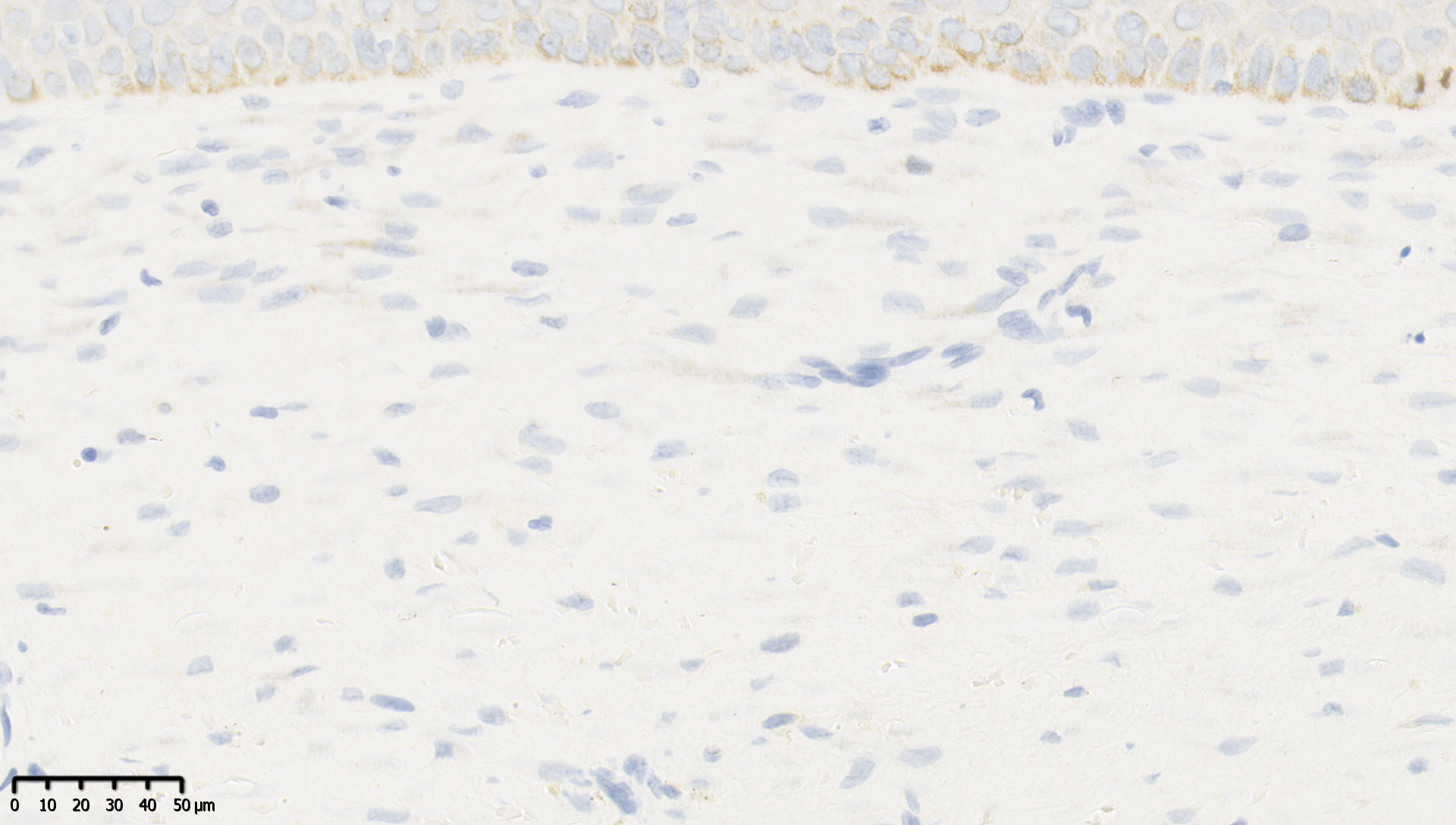

Supplement: S1 File — (ZIP) [file pone.0324264.s001.zip › supplement.material-1/Immunohistochemistry image/KI67/PL-HA-115.jpg]
