# Supplementary figures and images for: Platelet lysate-sodium hyaluronate gel promotes diabetic foot wound healing by regulating oxidative stress and autophagy (part 3 of 4)
Source: PLoS One. 2025 Jun 6;20(6):e0324264. doi: 10.1371/journal.pone.0324264 (PMC12143543; doi:10.1371/journal.pone.0324264)

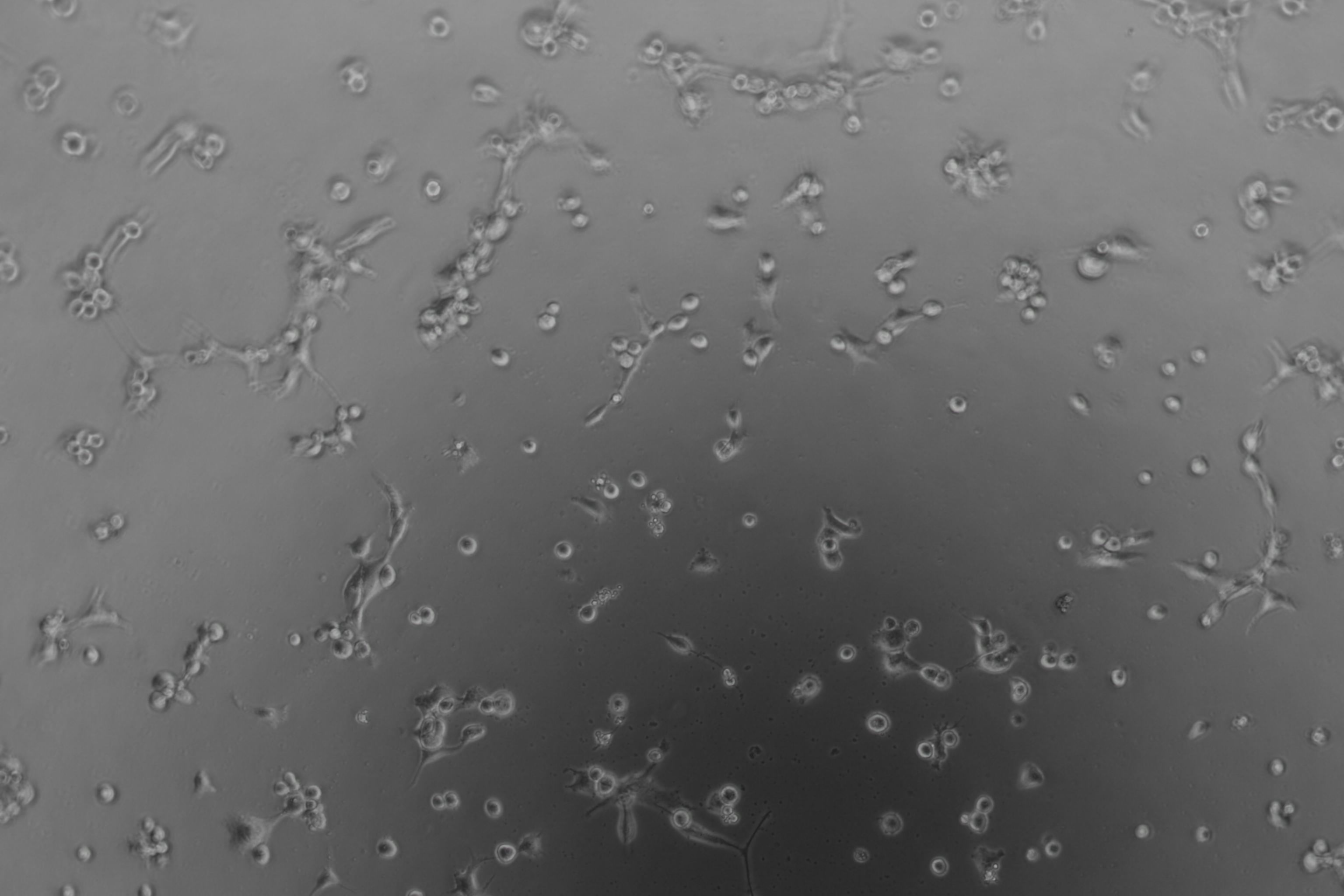

Supplement: S2 File — (ZIP) [file pone.0324264.s002.zip › supplement.material-2/images(tube formation assay)/144-model2.tif]

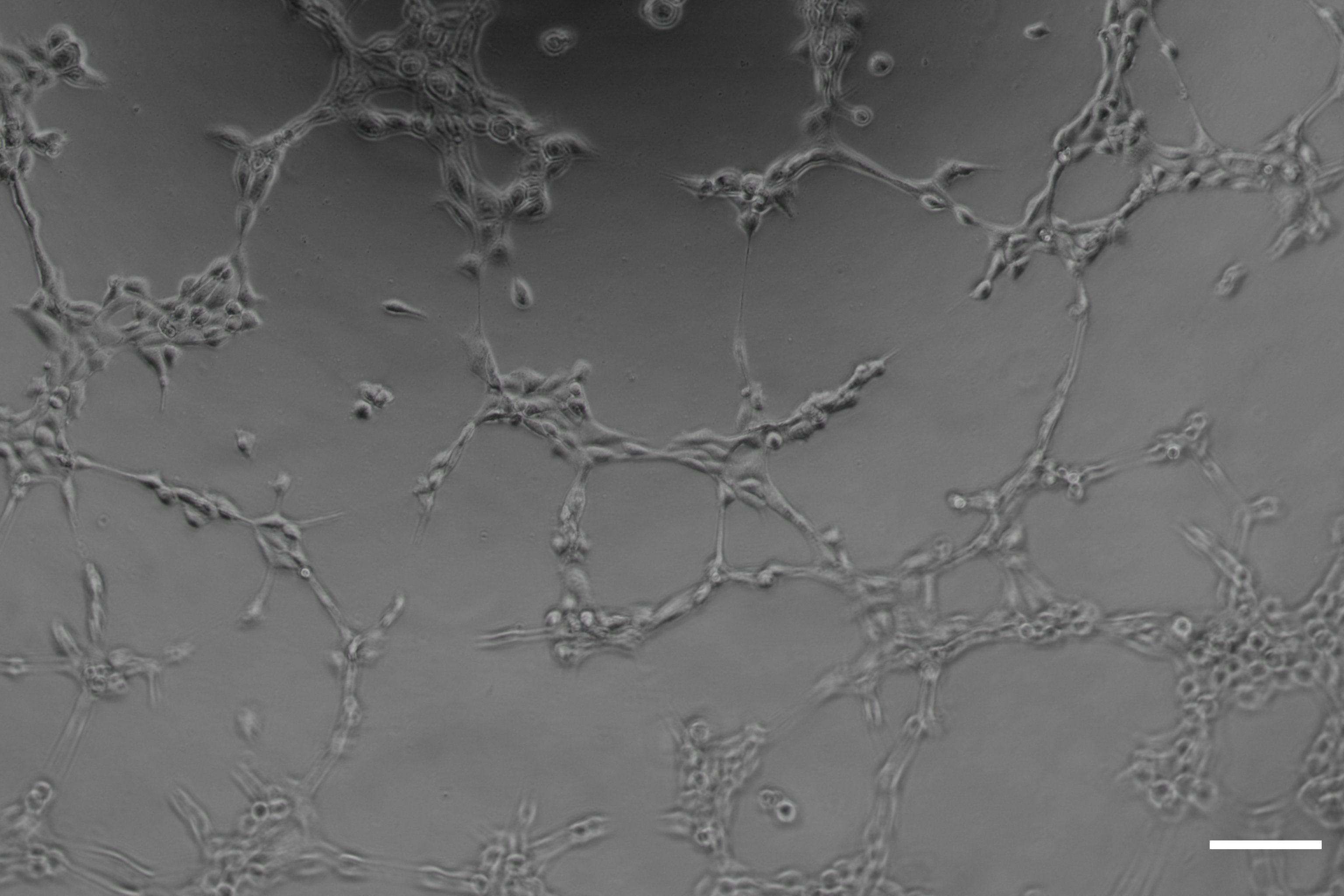

Supplement: S2 File — (ZIP) [file pone.0324264.s002.zip › supplement.material-2/images(tube formation assay)/144-pl1.jpg]

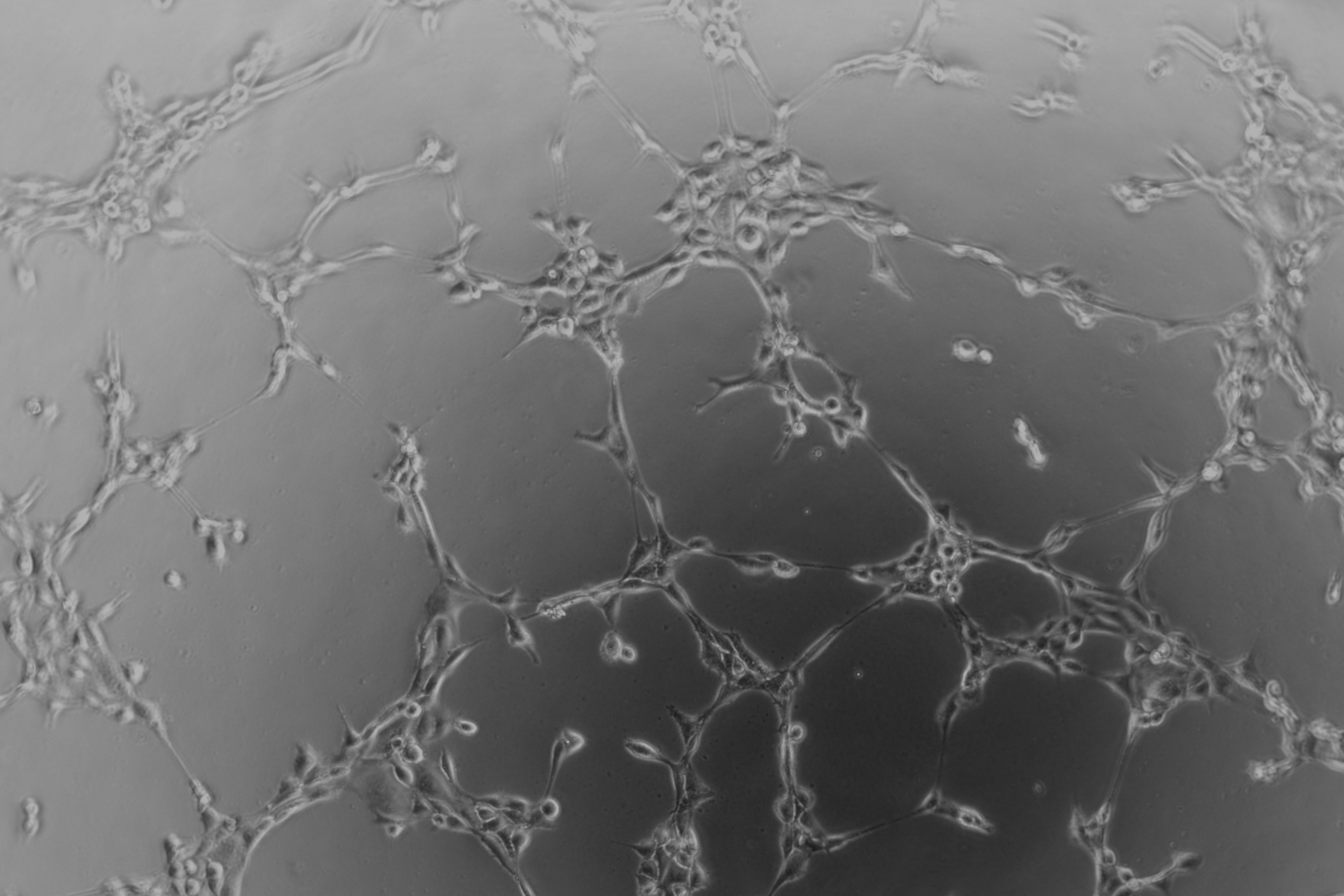

Supplement: S2 File — (ZIP) [file pone.0324264.s002.zip › supplement.material-2/images(tube formation assay)/144-pl2.tif]

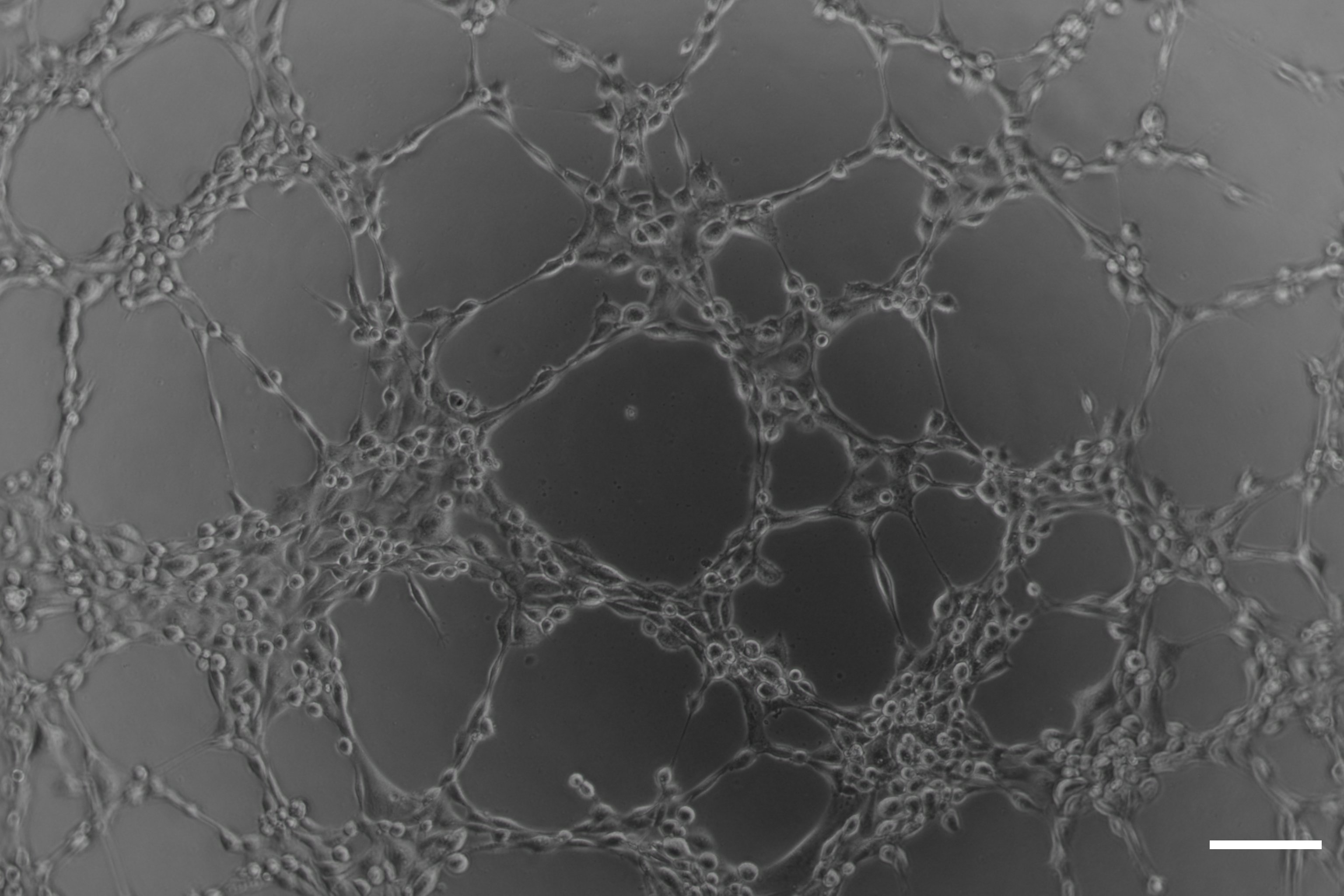

Supplement: S2 File — (ZIP) [file pone.0324264.s002.zip › supplement.material-2/images(tube formation assay)/48-control1.jpg]

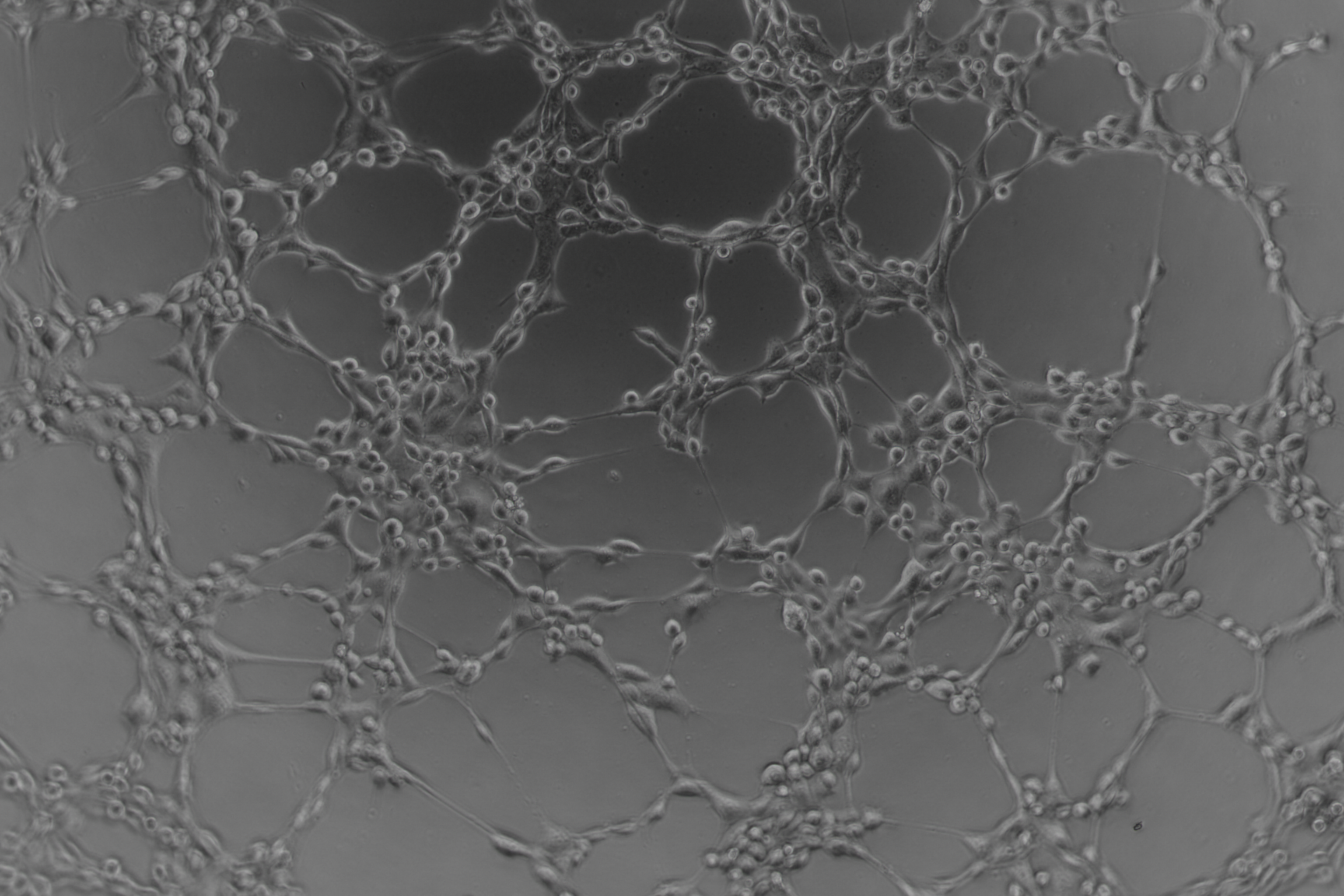

Supplement: S2 File — (ZIP) [file pone.0324264.s002.zip › supplement.material-2/images(tube formation assay)/48-control2.tif]

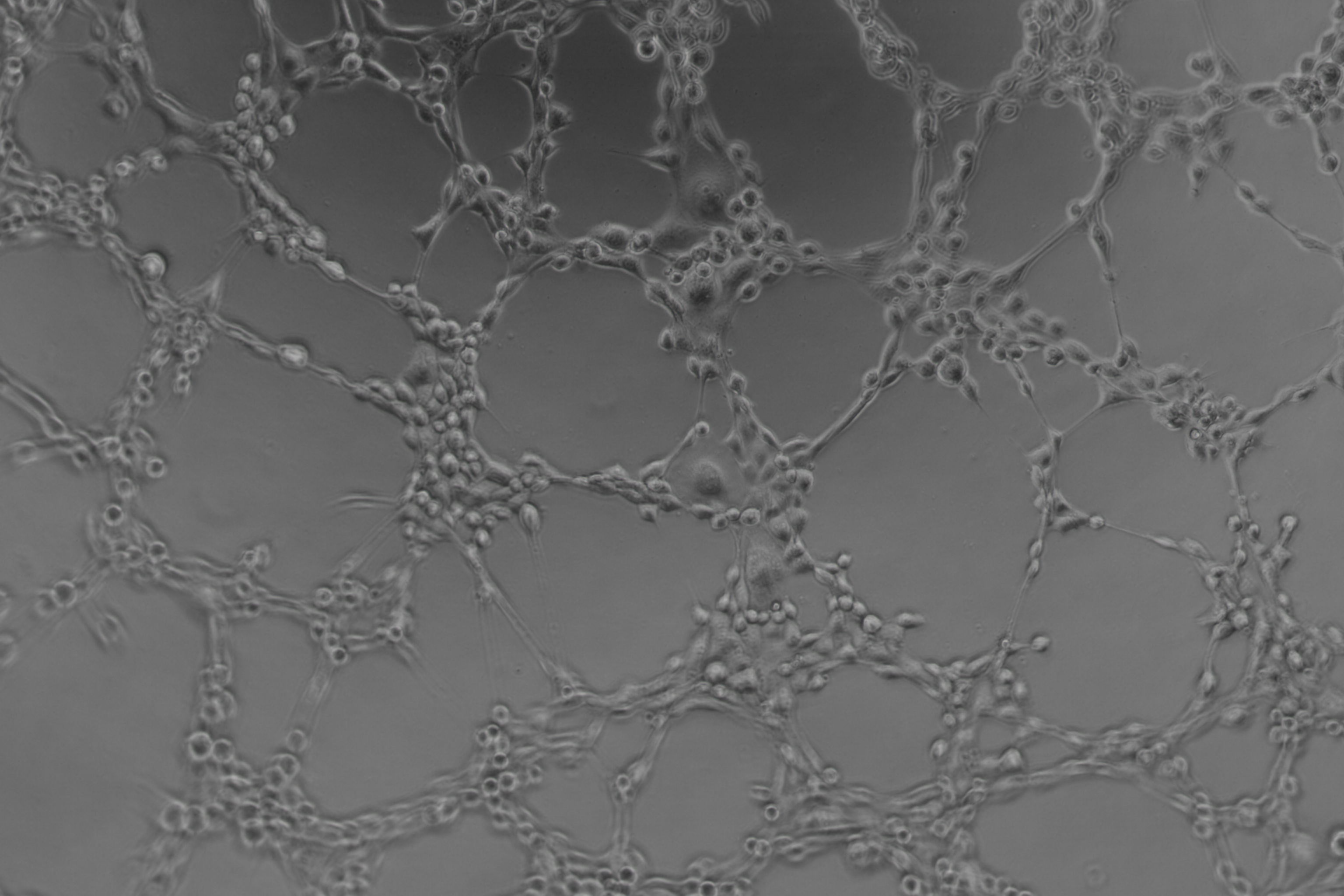

Supplement: S2 File — (ZIP) [file pone.0324264.s002.zip › supplement.material-2/images(tube formation assay)/48-control3.tif]

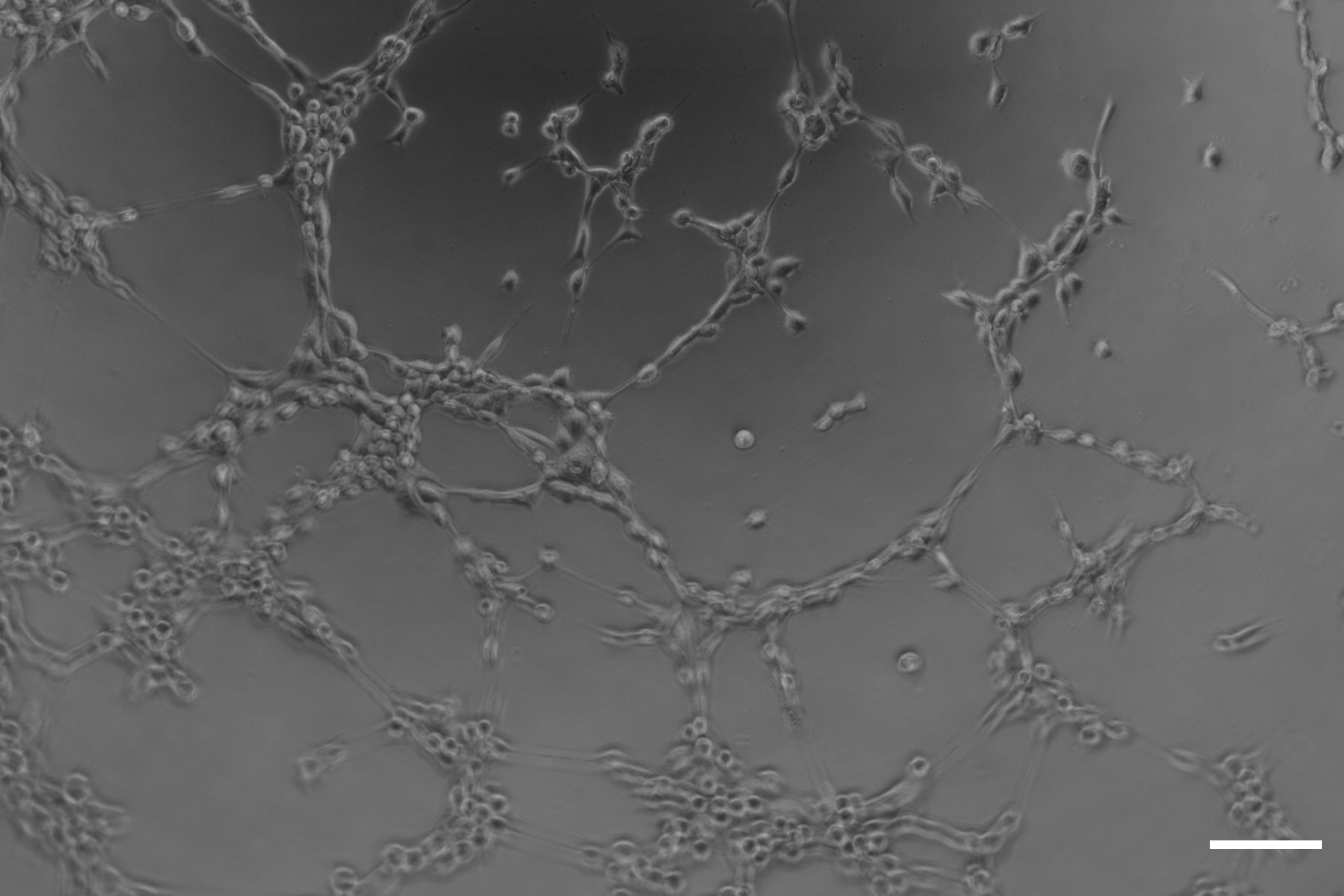

Supplement: S2 File — (ZIP) [file pone.0324264.s002.zip › supplement.material-2/images(tube formation assay)/48-model1.jpg]

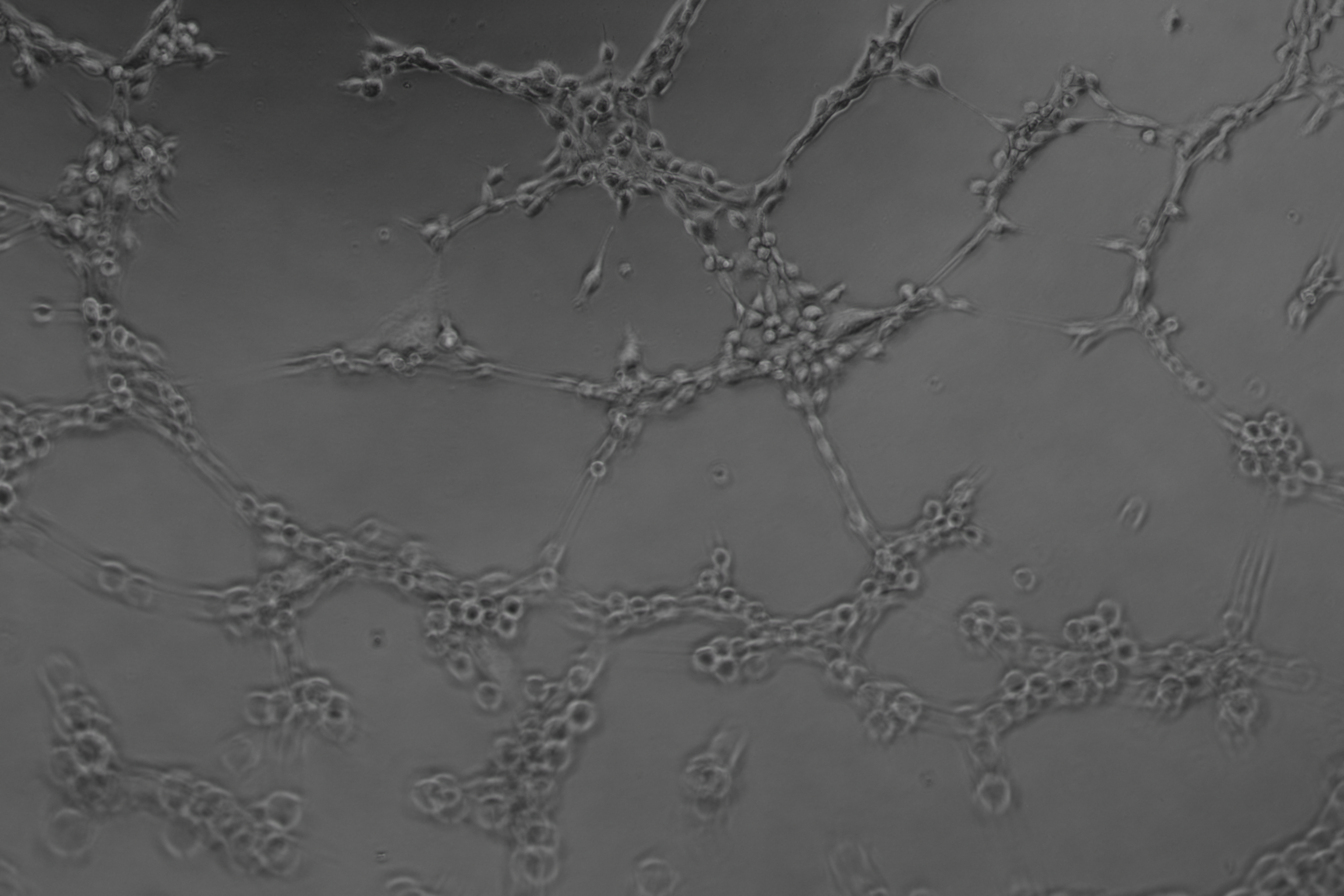

Supplement: S2 File — (ZIP) [file pone.0324264.s002.zip › supplement.material-2/images(tube formation assay)/48-model2.tif]

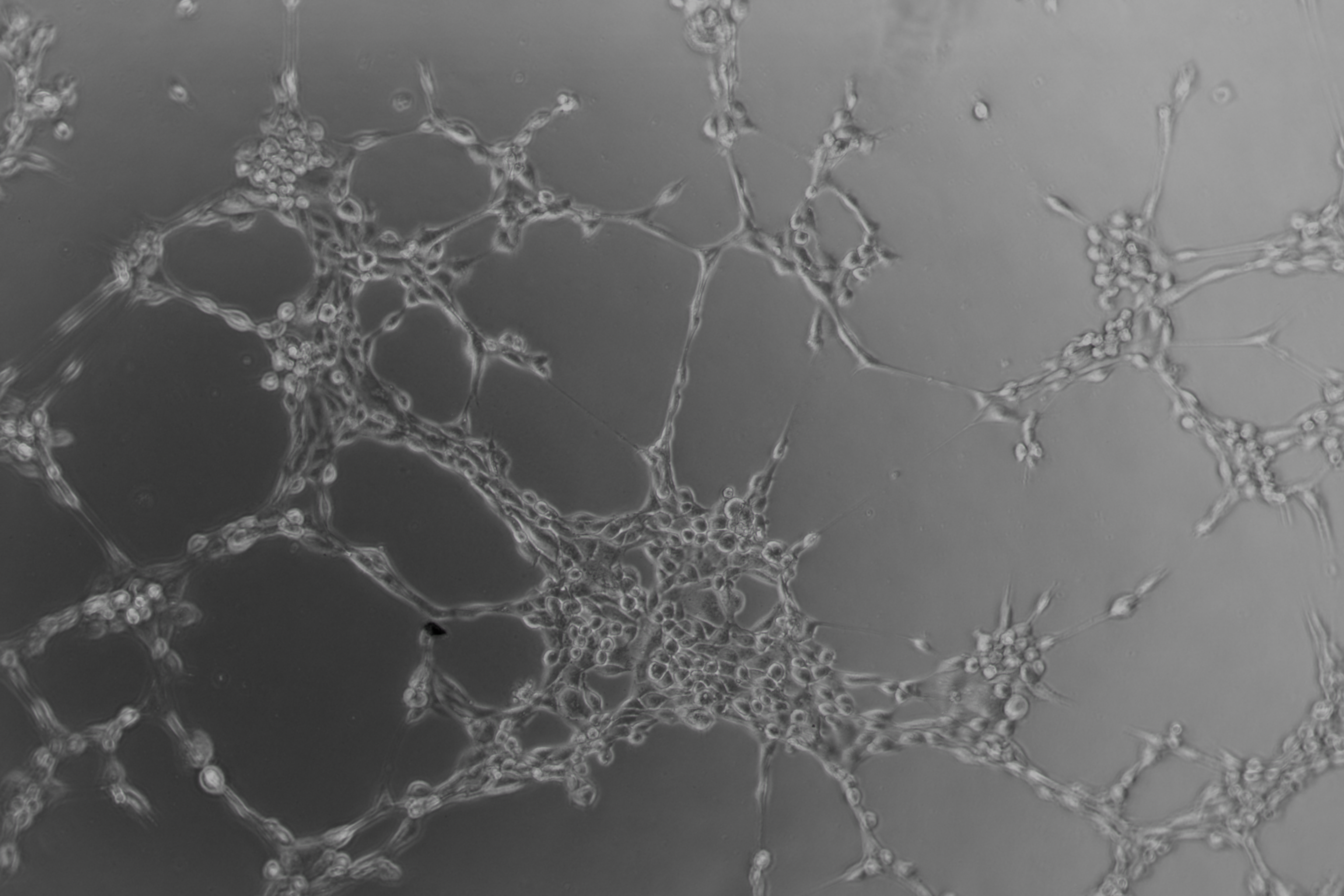

Supplement: S2 File — (ZIP) [file pone.0324264.s002.zip › supplement.material-2/images(tube formation assay)/48-model3.tif]

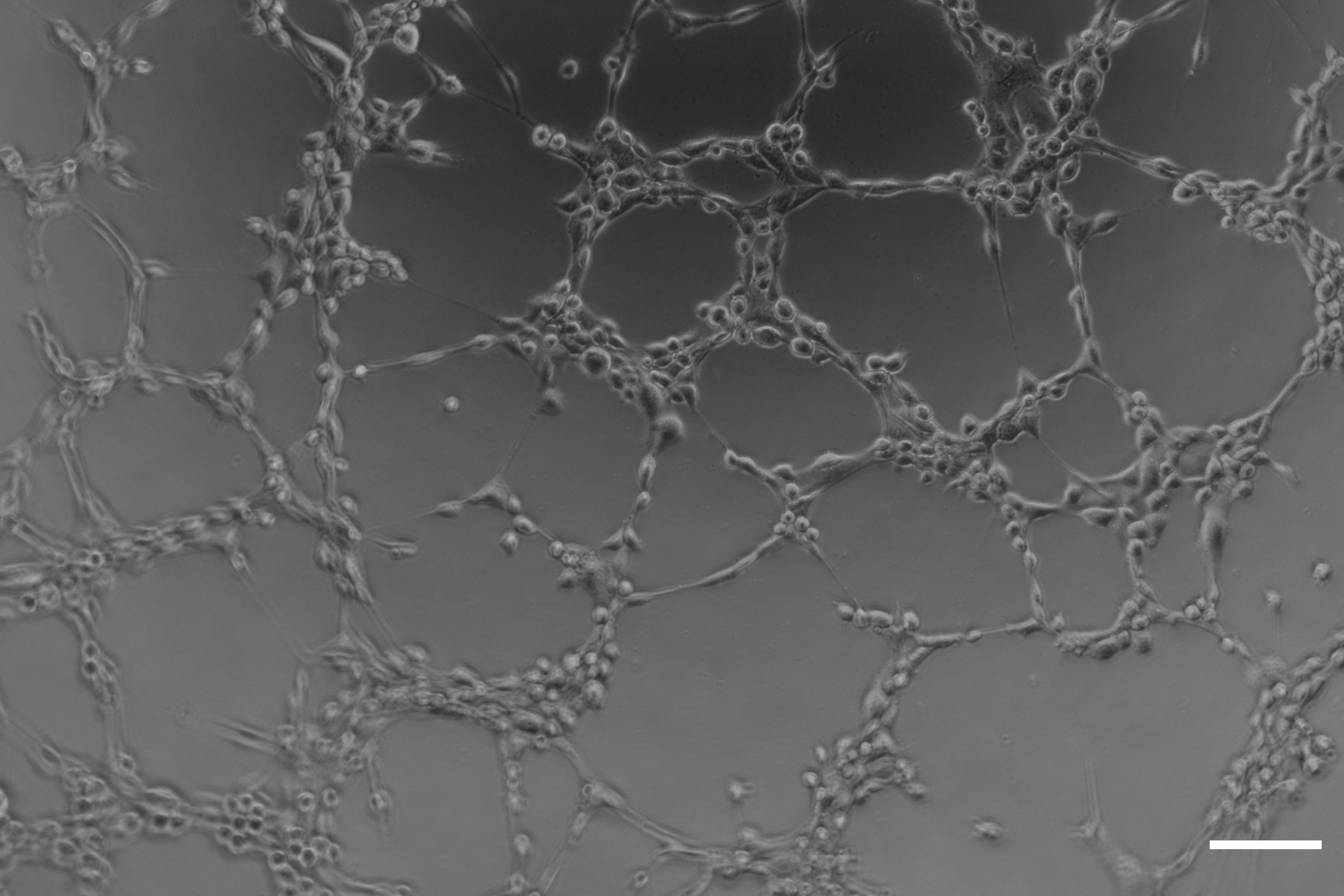

Supplement: S2 File — (ZIP) [file pone.0324264.s002.zip › supplement.material-2/images(tube formation assay)/48-pl1.jpg]

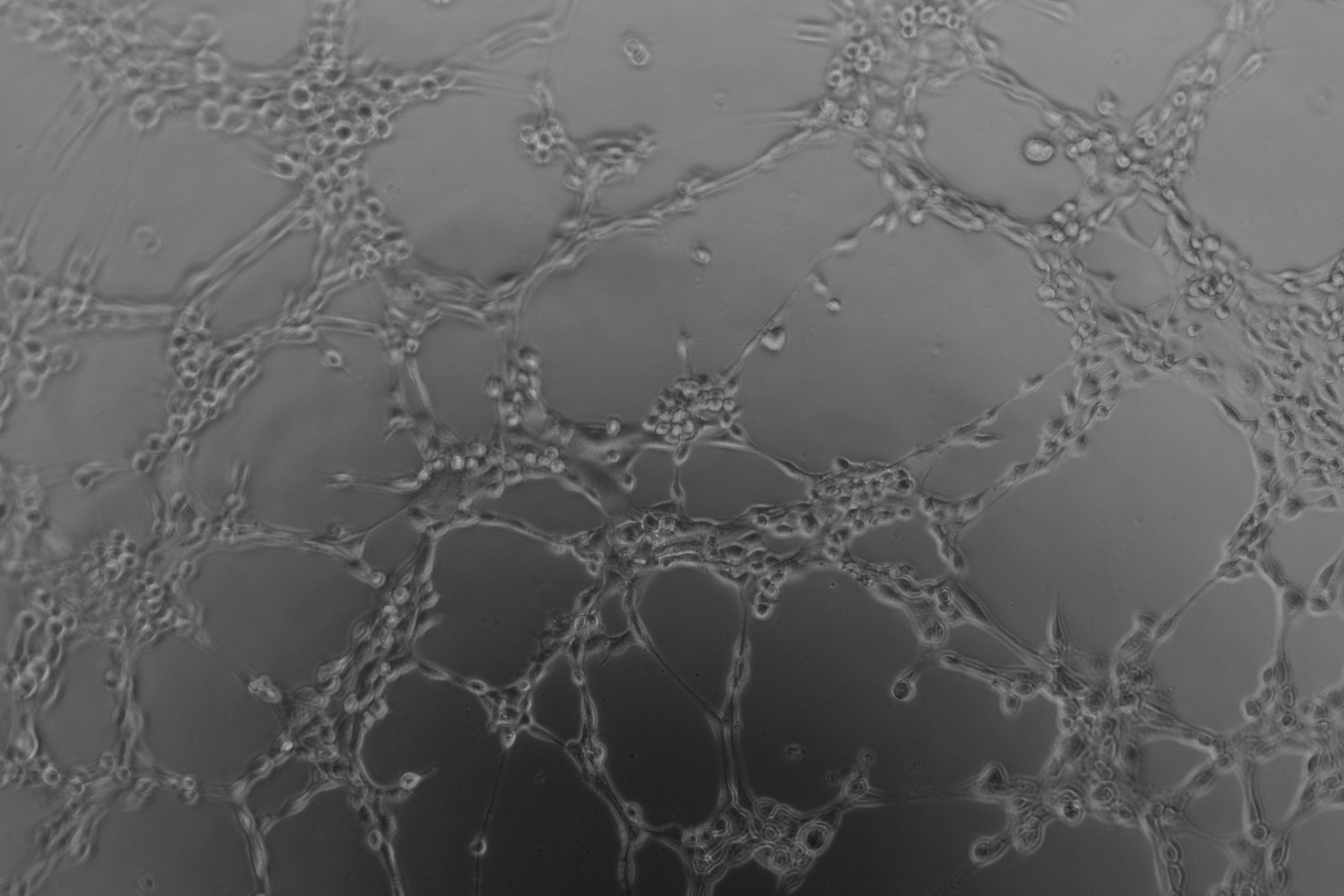

Supplement: S2 File — (ZIP) [file pone.0324264.s002.zip › supplement.material-2/images(tube formation assay)/48-pl2.tif]

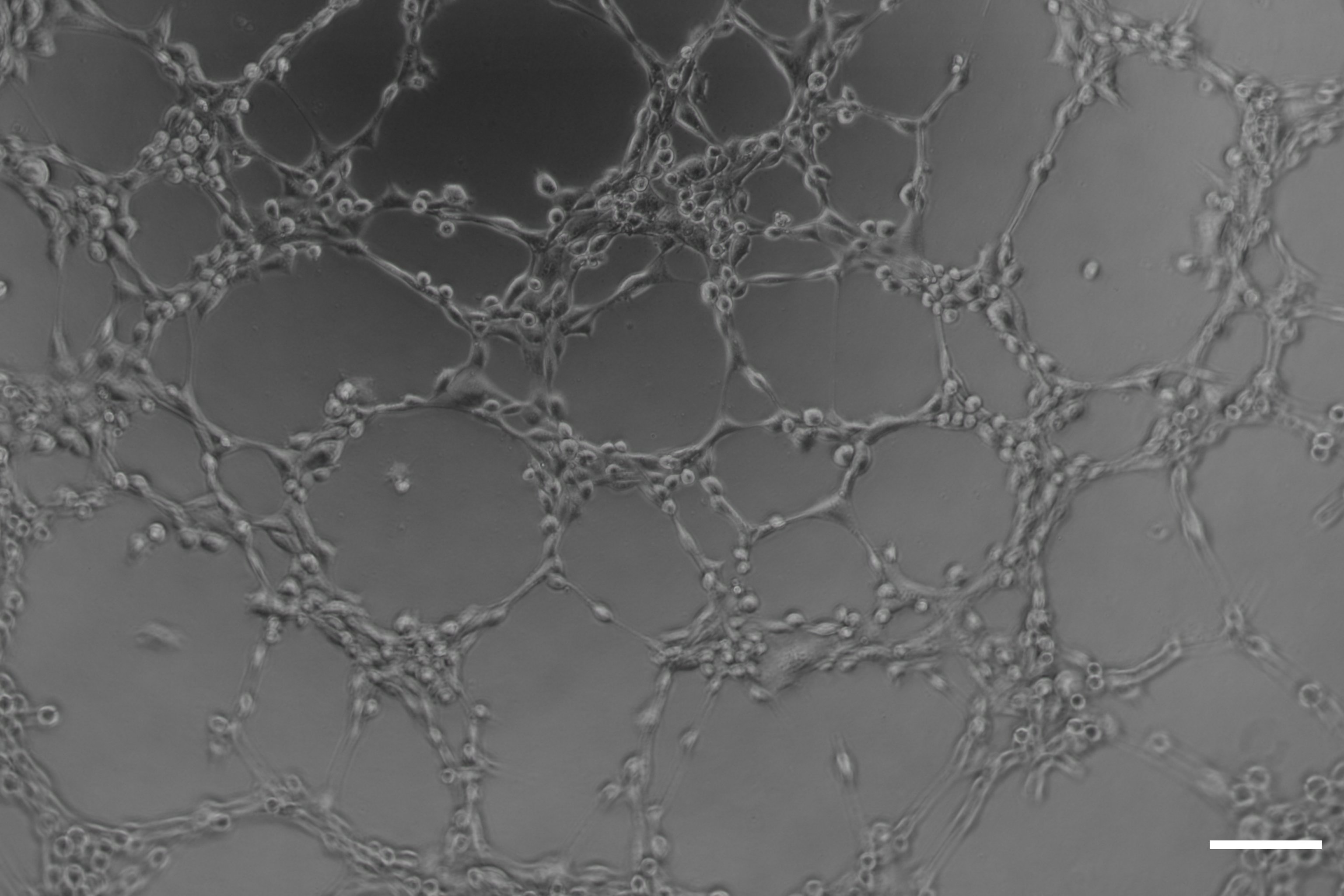

Supplement: S2 File — (ZIP) [file pone.0324264.s002.zip › supplement.material-2/images(tube formation assay)/96-control1.jpg]

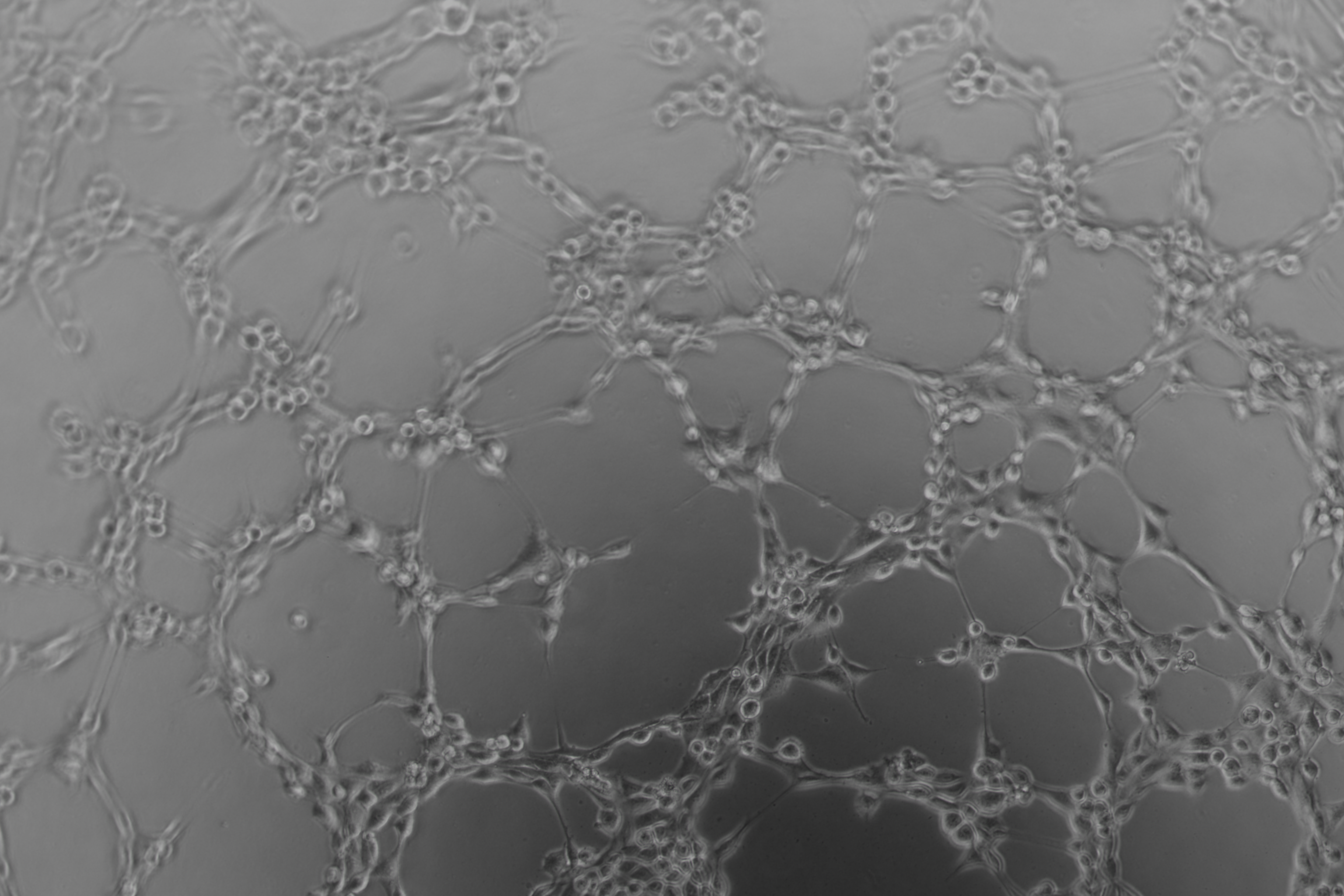

Supplement: S2 File — (ZIP) [file pone.0324264.s002.zip › supplement.material-2/images(tube formation assay)/96-control2.tif]

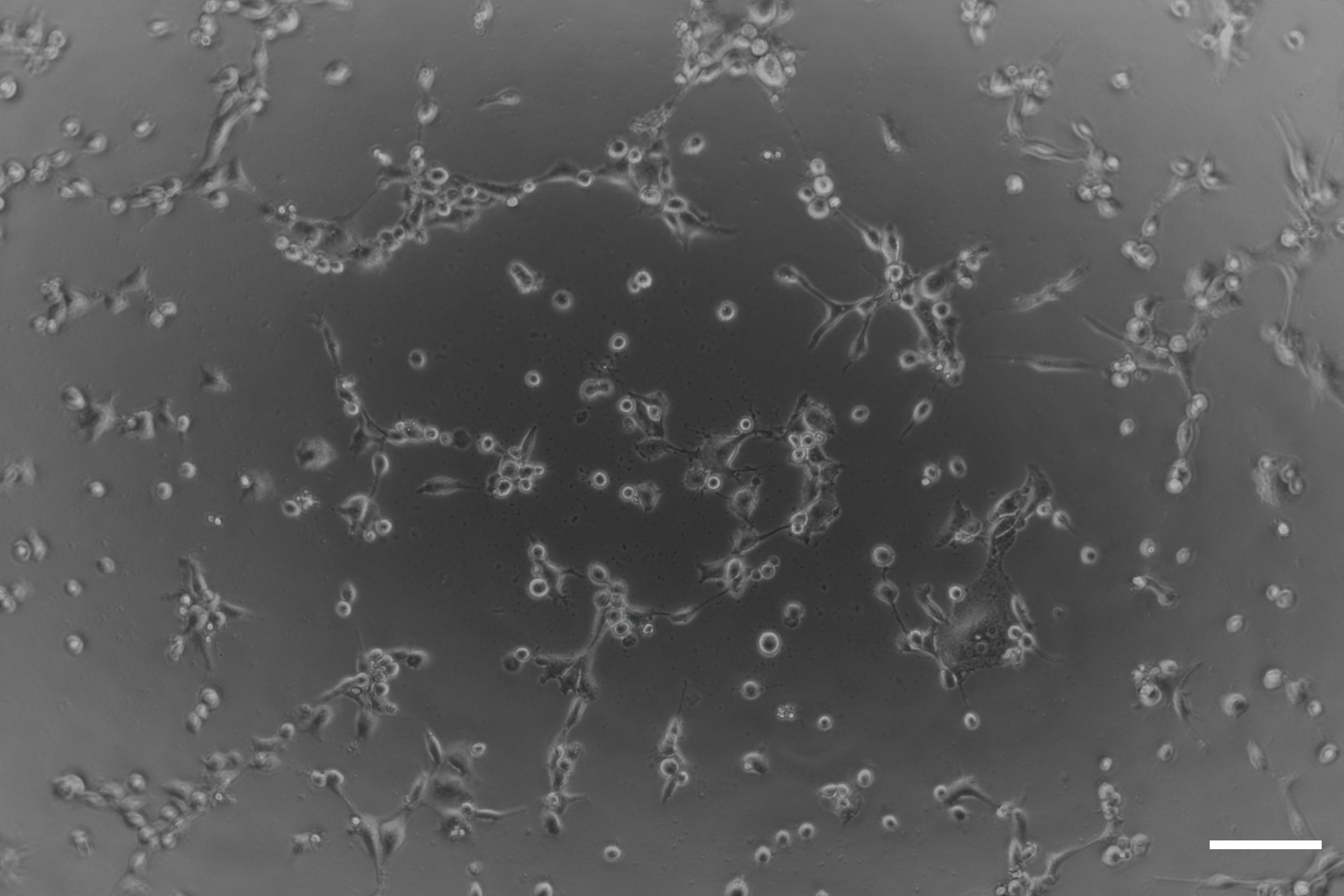

Supplement: S2 File — (ZIP) [file pone.0324264.s002.zip › supplement.material-2/images(tube formation assay)/96-model1.jpg]

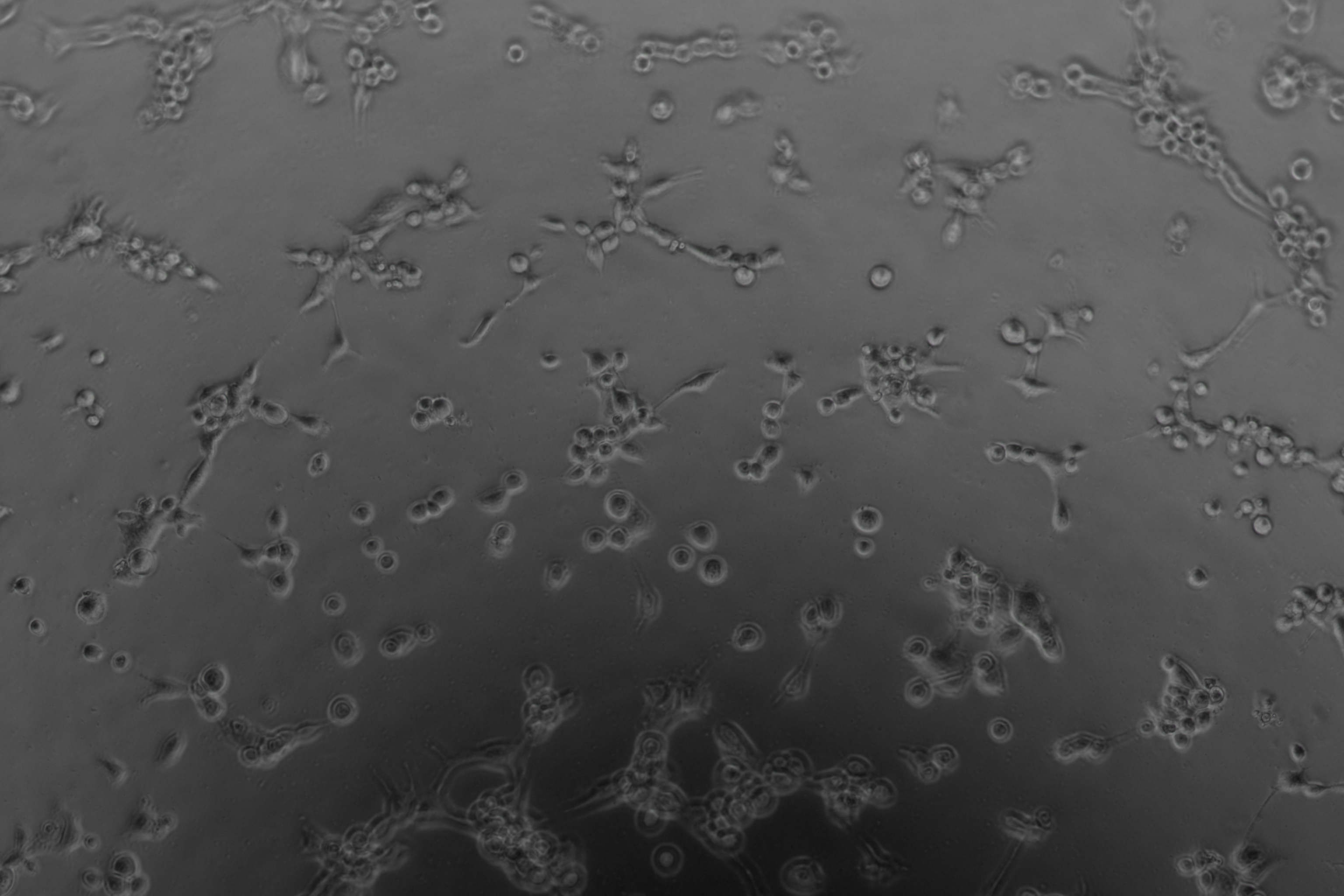

Supplement: S2 File — (ZIP) [file pone.0324264.s002.zip › supplement.material-2/images(tube formation assay)/96-model2.tif]

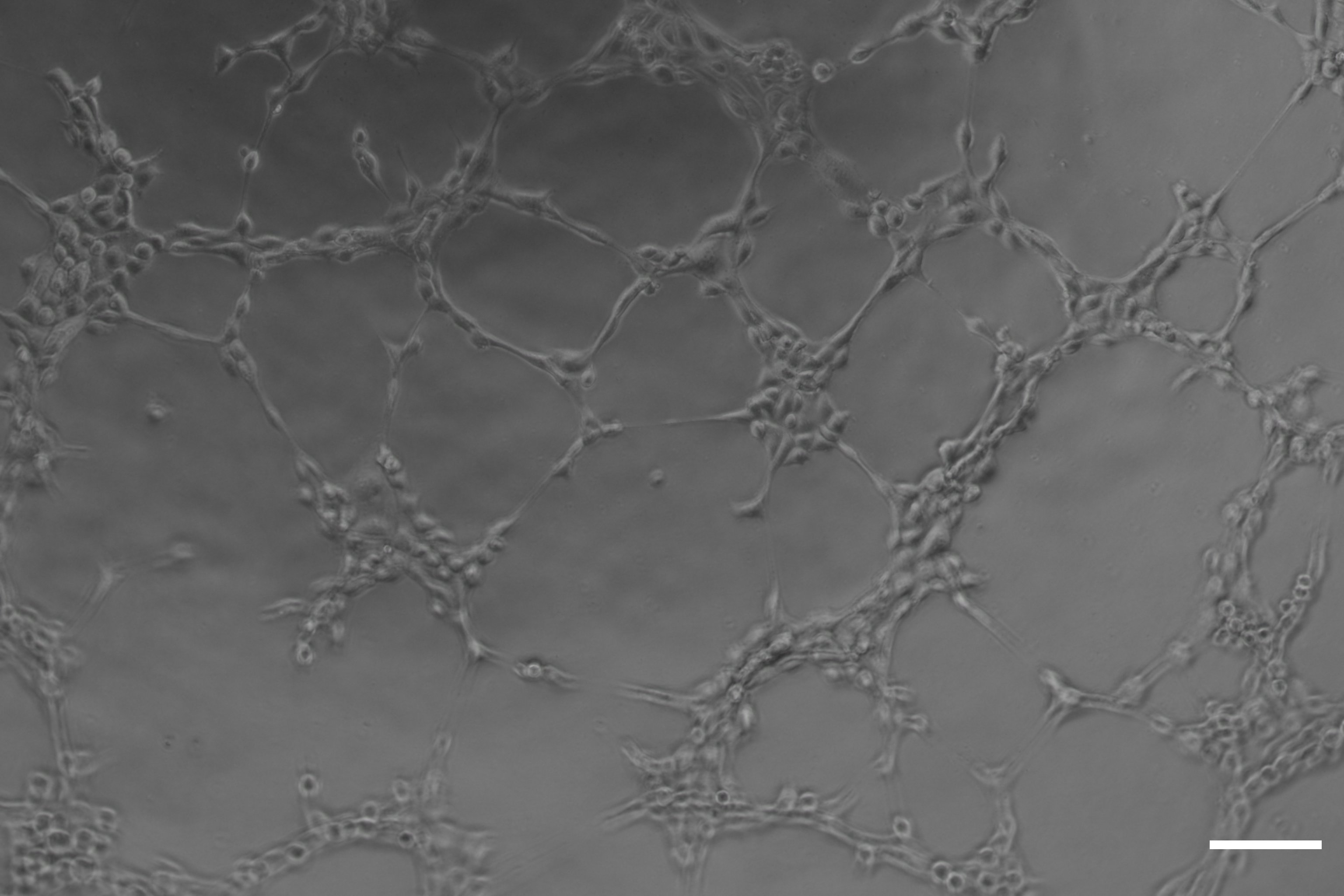

Supplement: S2 File — (ZIP) [file pone.0324264.s002.zip › supplement.material-2/images(tube formation assay)/96-pl1.jpg]

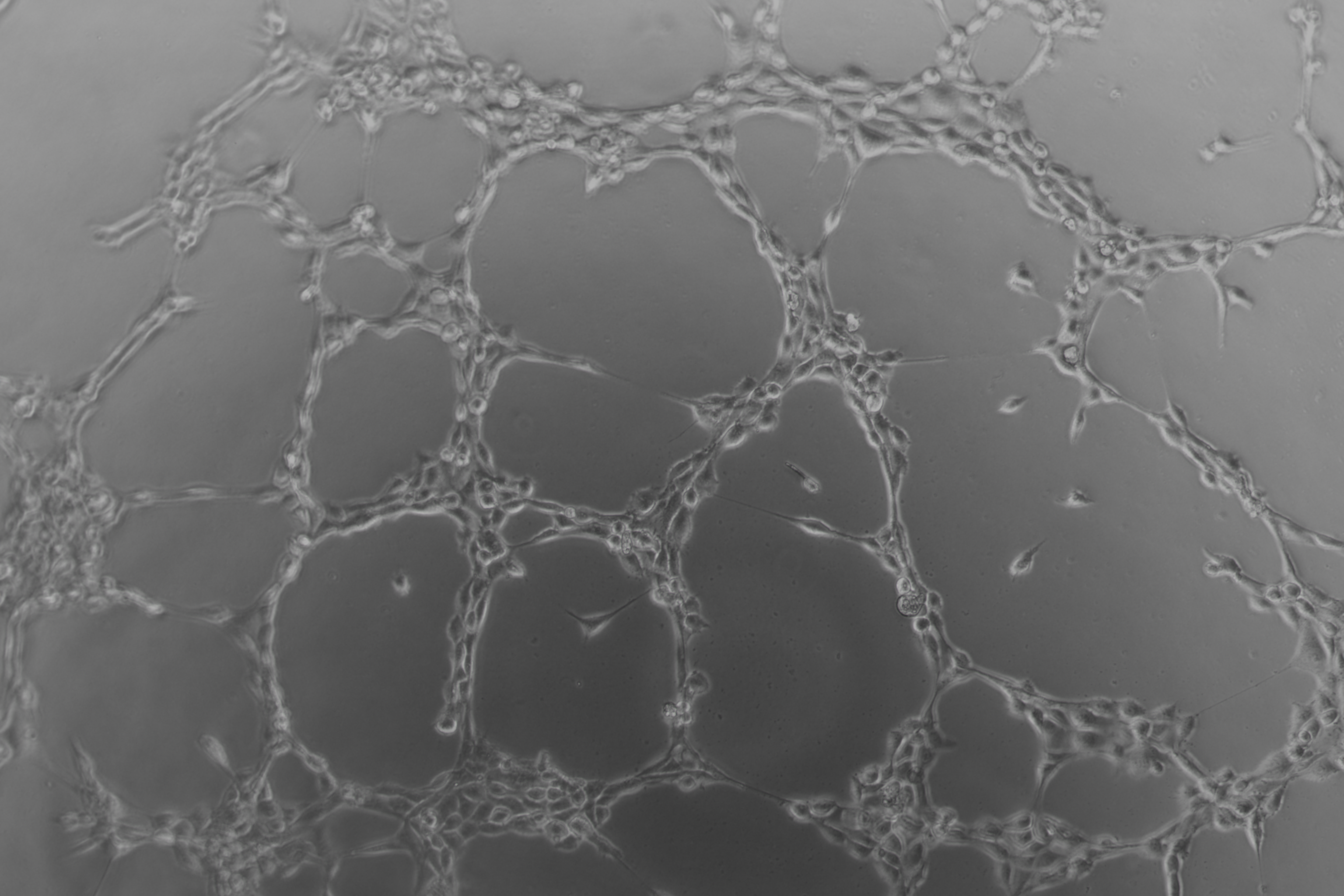

Supplement: S2 File — (ZIP) [file pone.0324264.s002.zip › supplement.material-2/images(tube formation assay)/96-pl2.tif]

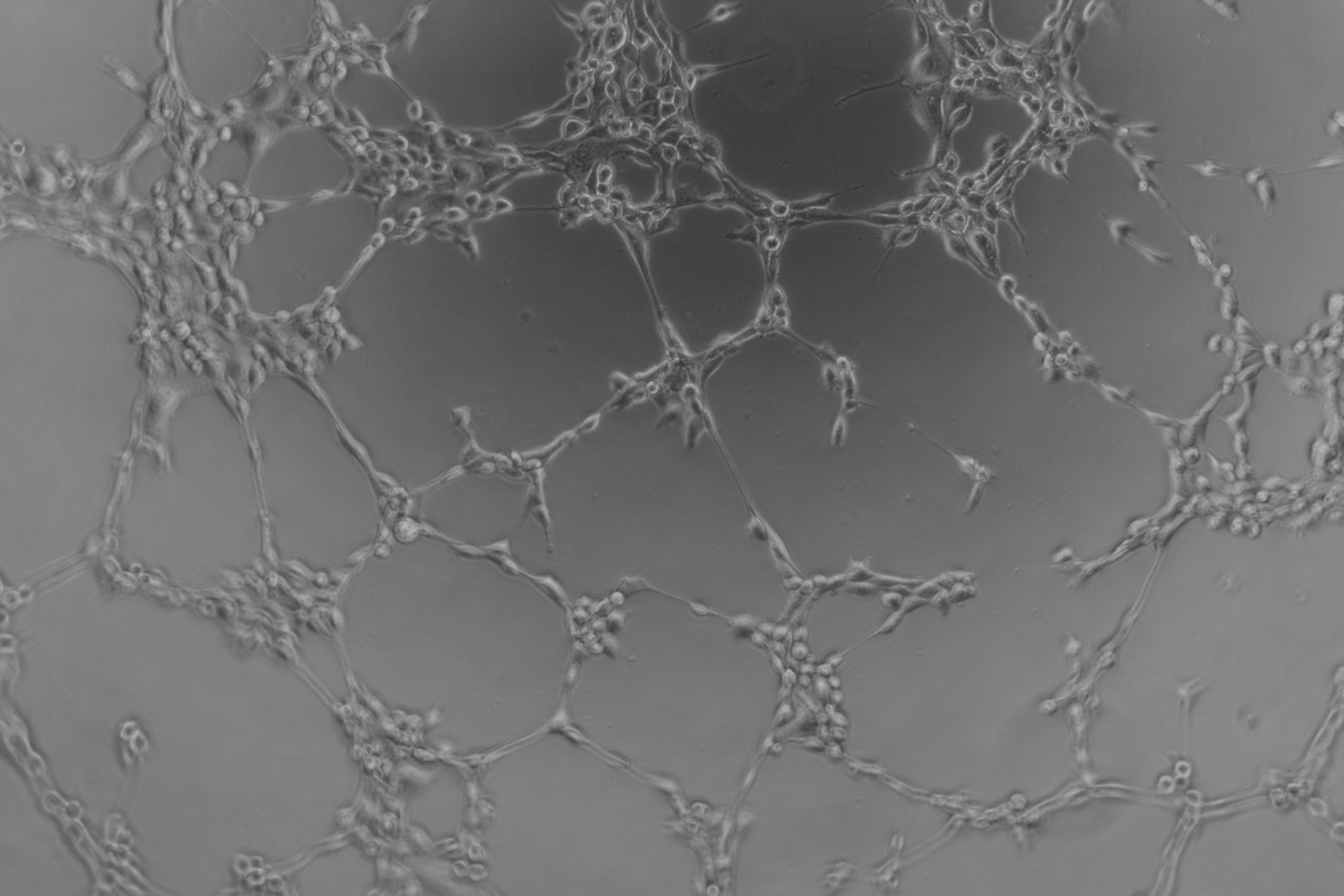

Supplement: S3 File — (ZIP) [file pone.0324264.s003.zip › supplement.material-3/contined images(tube formation assay)/144-control3.tif]

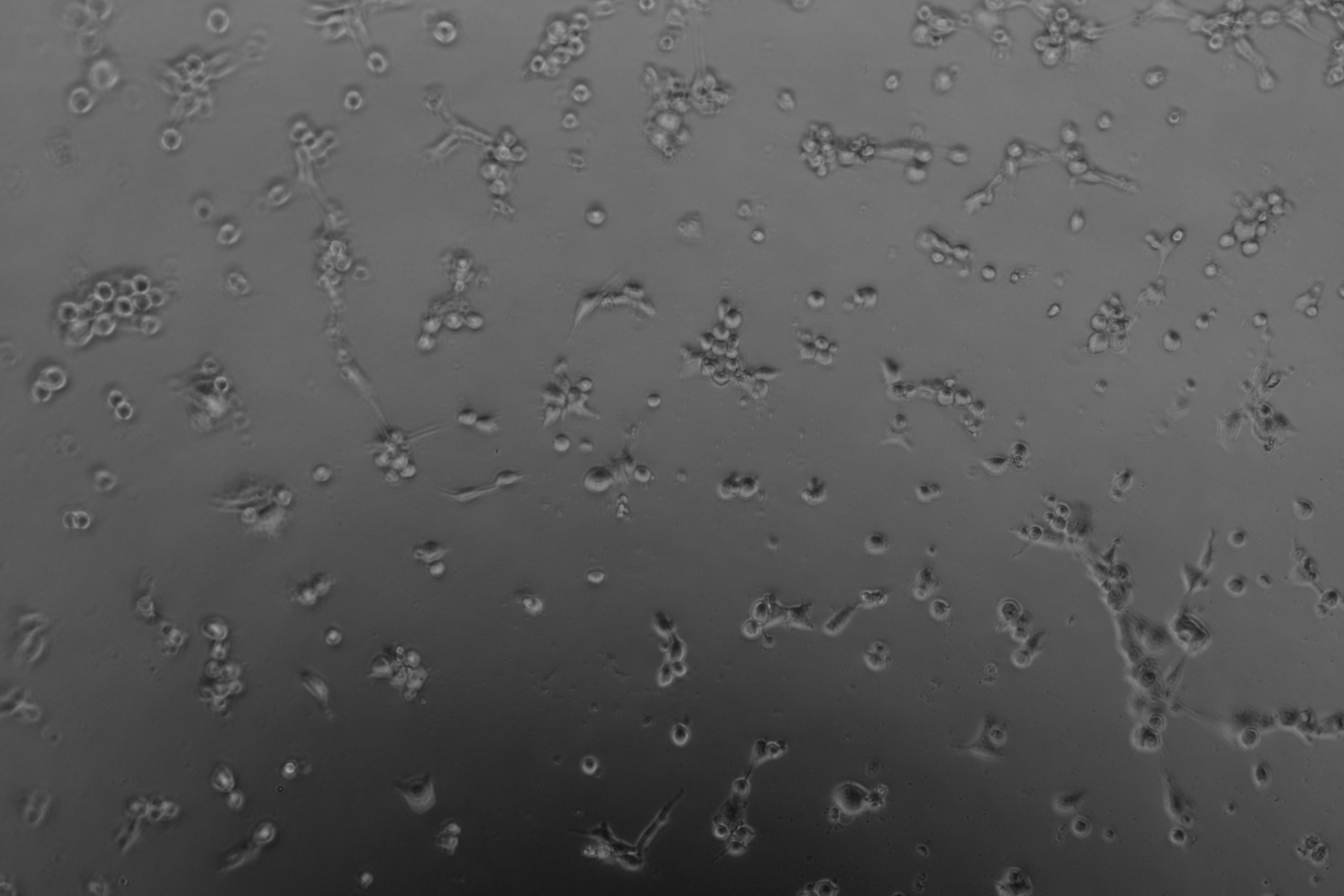

Supplement: S3 File — (ZIP) [file pone.0324264.s003.zip › supplement.material-3/contined images(tube formation assay)/144-model3.tif]

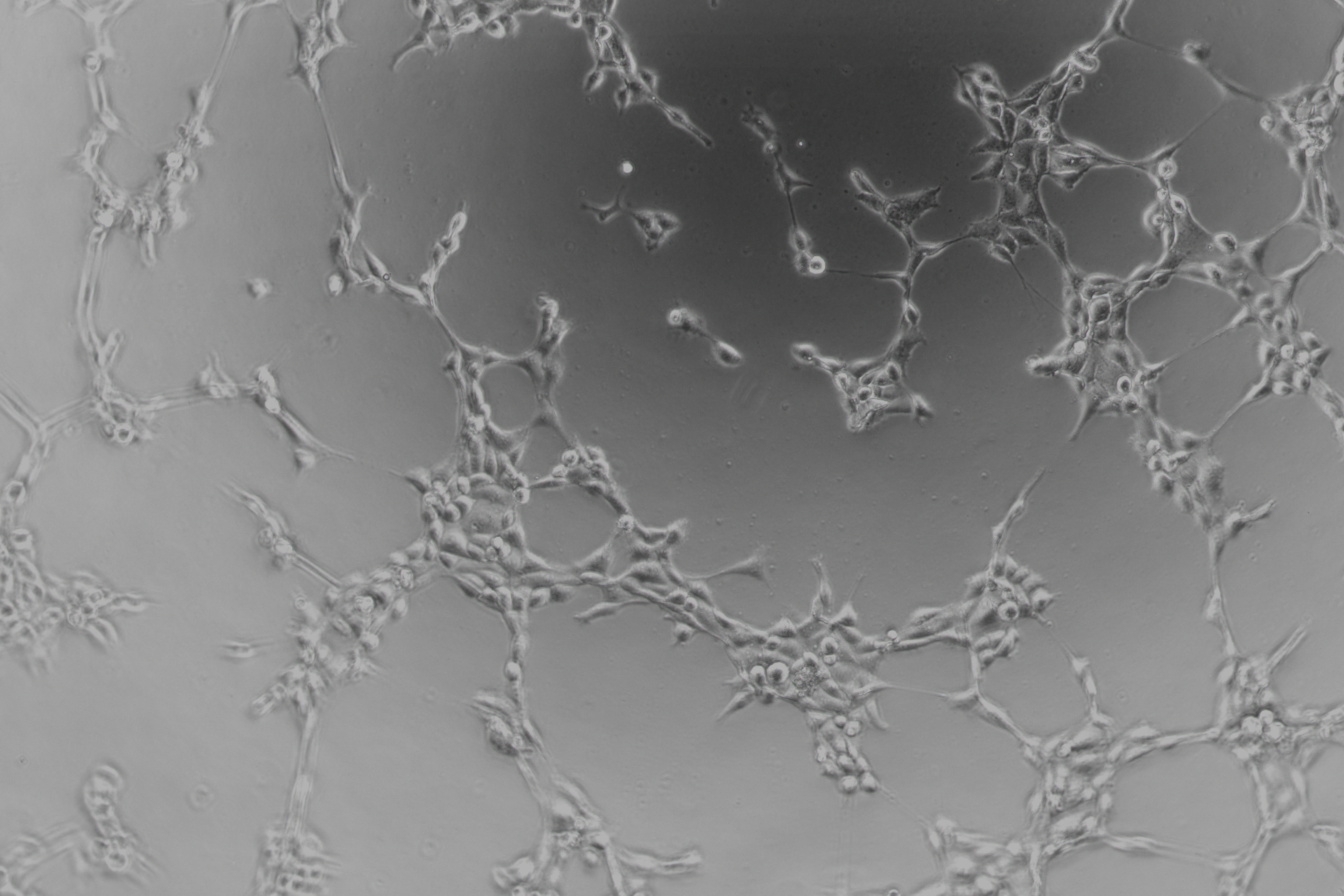

Supplement: S3 File — (ZIP) [file pone.0324264.s003.zip › supplement.material-3/contined images(tube formation assay)/144-pl3.tif]

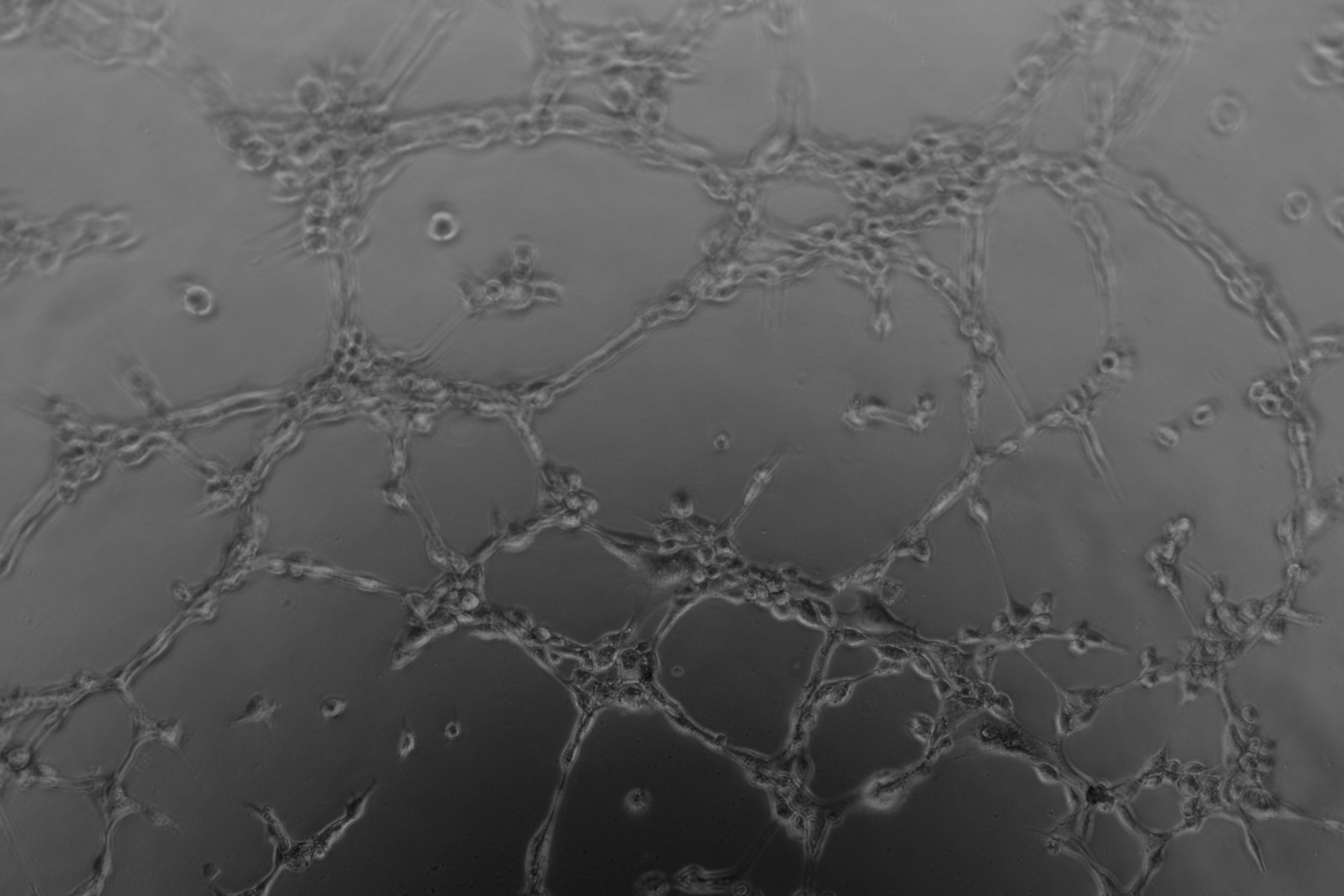

Supplement: S3 File — (ZIP) [file pone.0324264.s003.zip › supplement.material-3/contined images(tube formation assay)/48-pl3.tif]

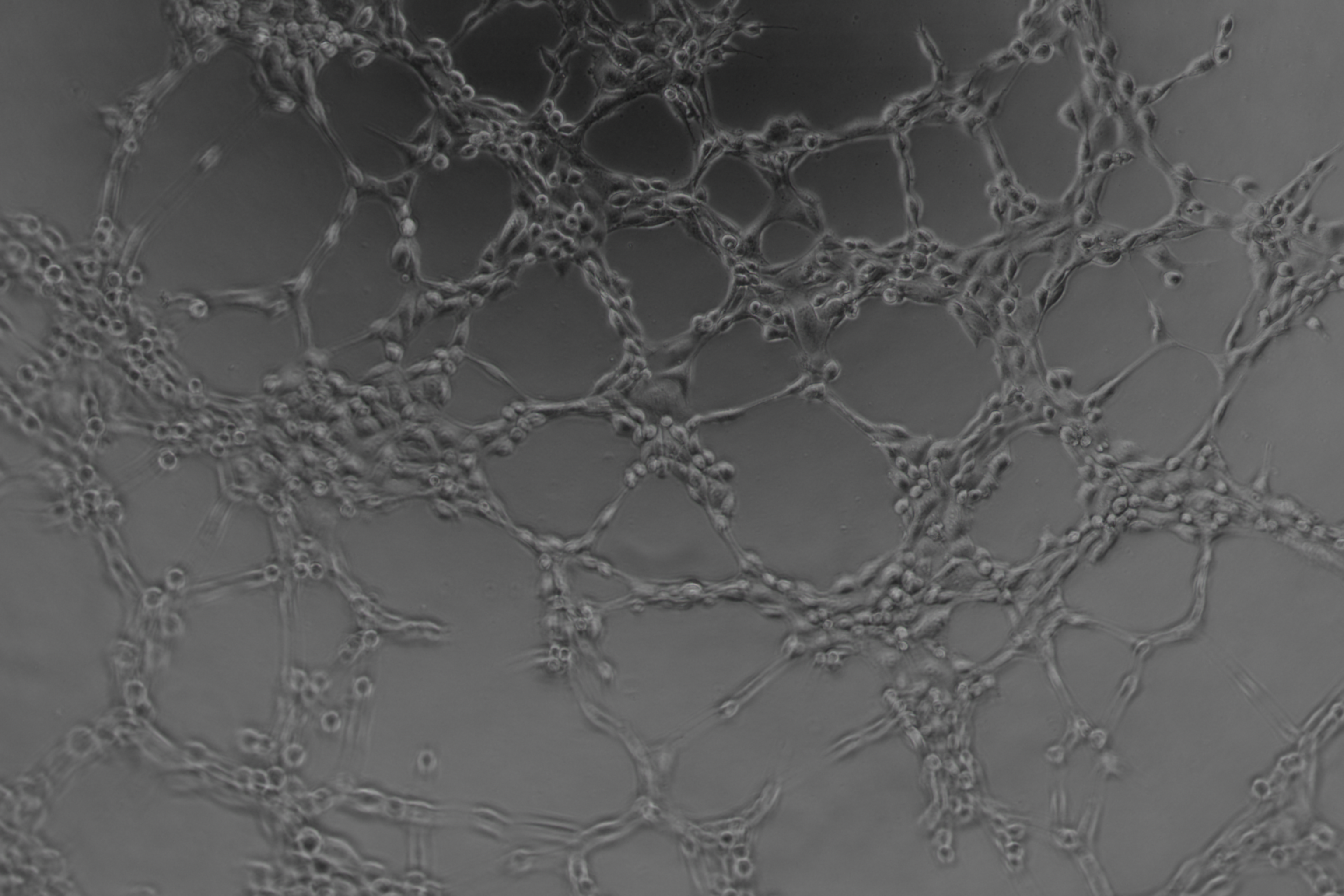

Supplement: S3 File — (ZIP) [file pone.0324264.s003.zip › supplement.material-3/contined images(tube formation assay)/96-control3.tif]

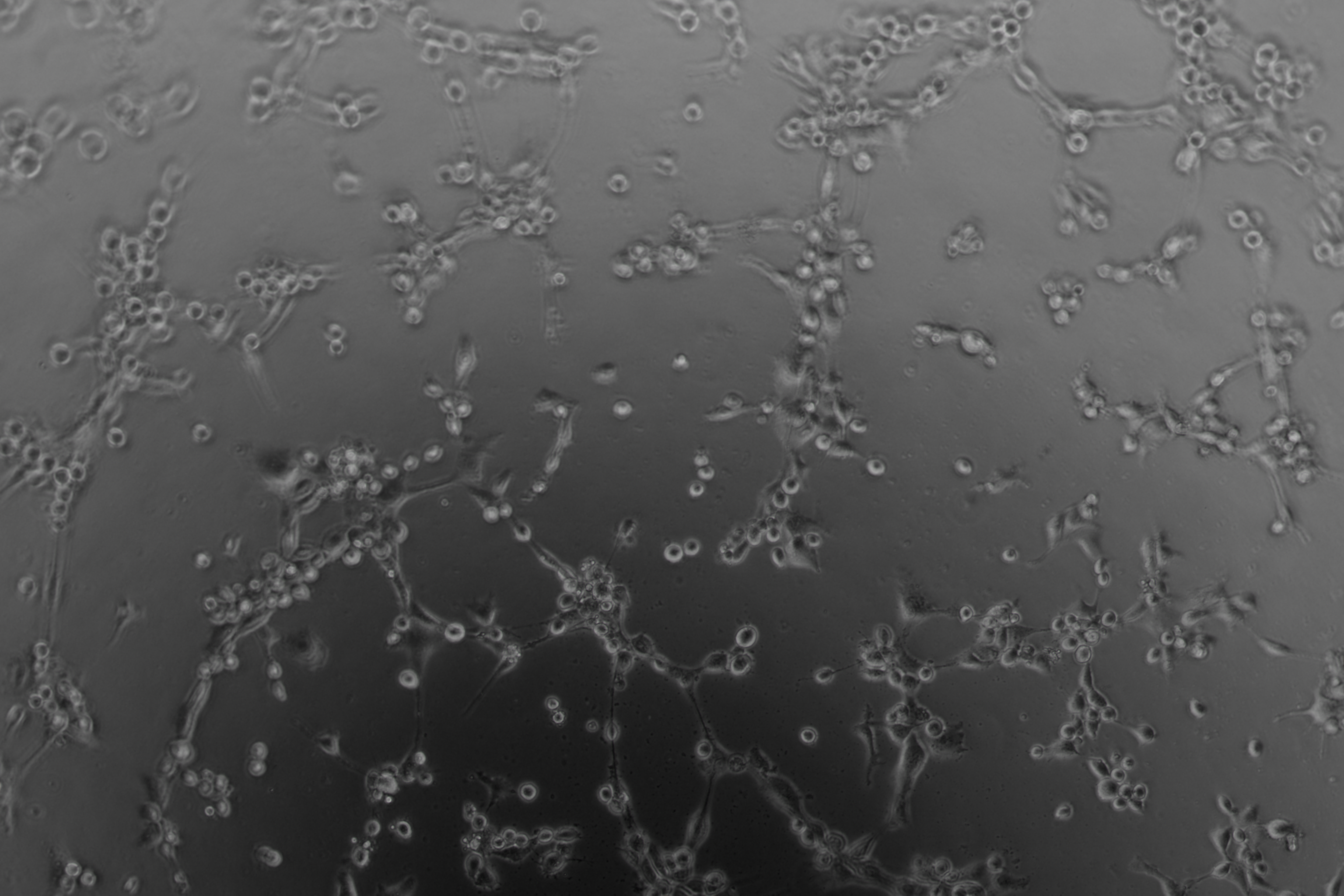

Supplement: S3 File — (ZIP) [file pone.0324264.s003.zip › supplement.material-3/contined images(tube formation assay)/96-model-3.tif]

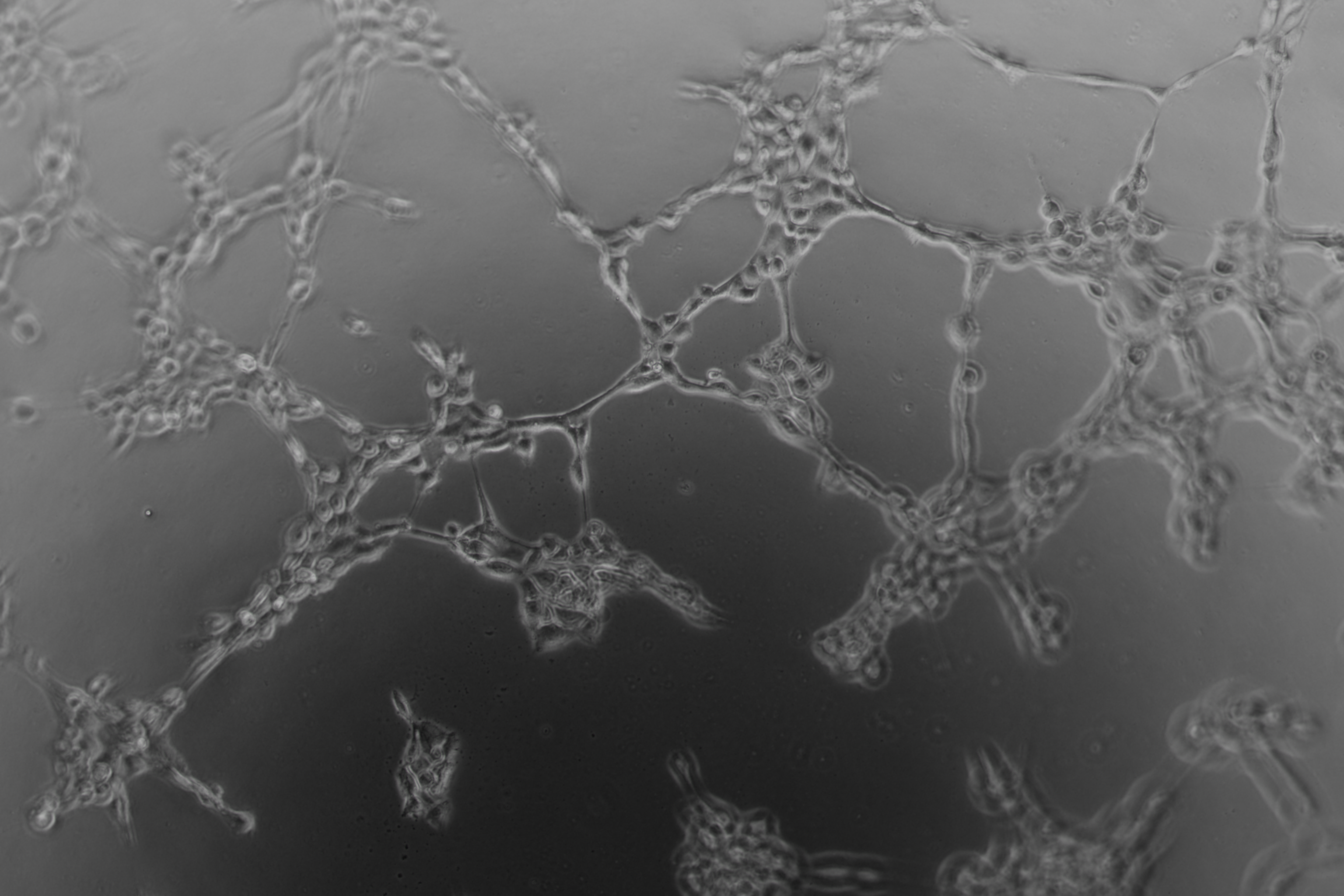

Supplement: S3 File — (ZIP) [file pone.0324264.s003.zip › supplement.material-3/contined images(tube formation assay)/96-pl3.tif]

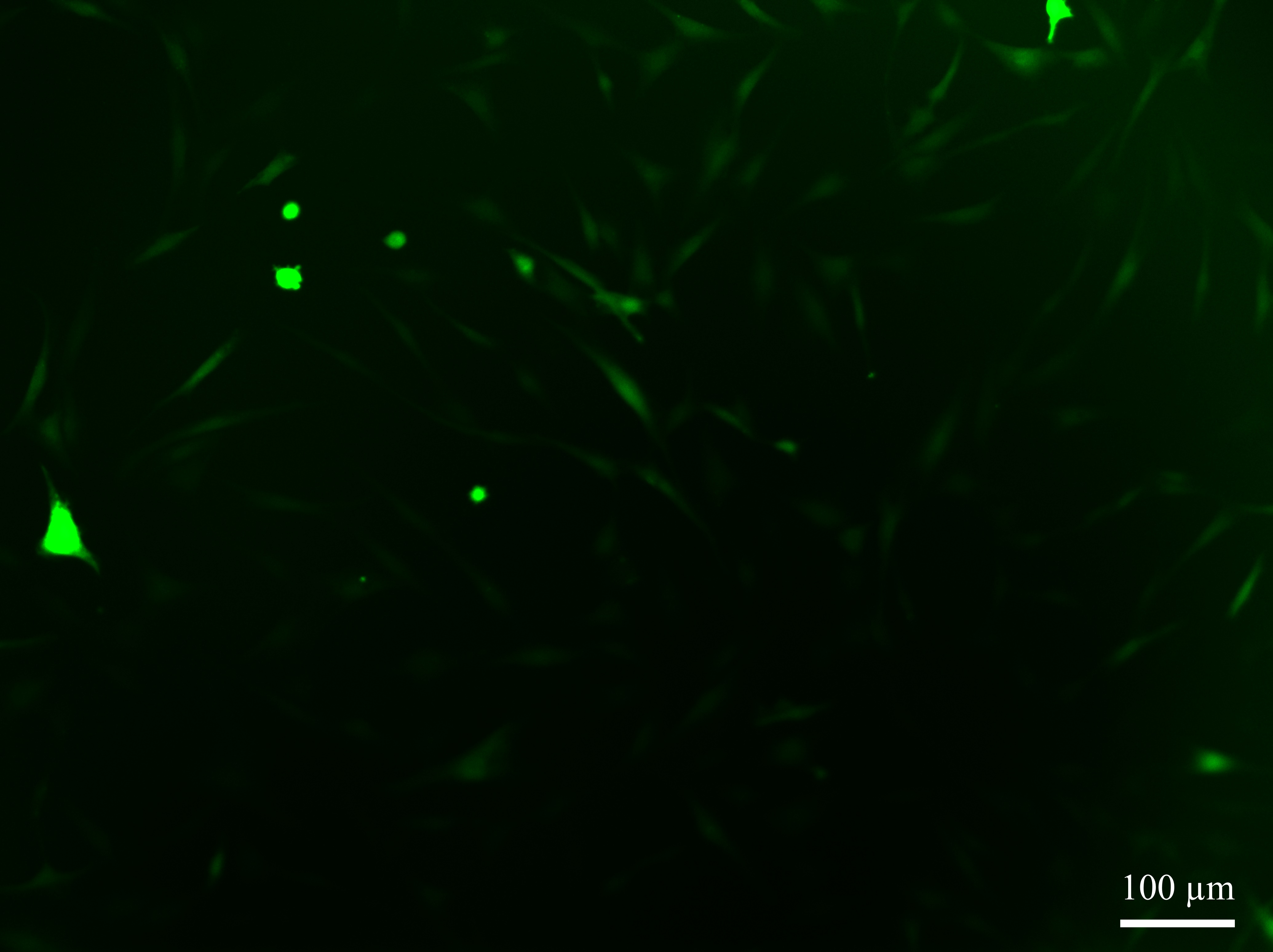

Supplement: S3 File — (ZIP) [file pone.0324264.s003.zip › supplement.material-3/ROS/144-Control1.jpg]

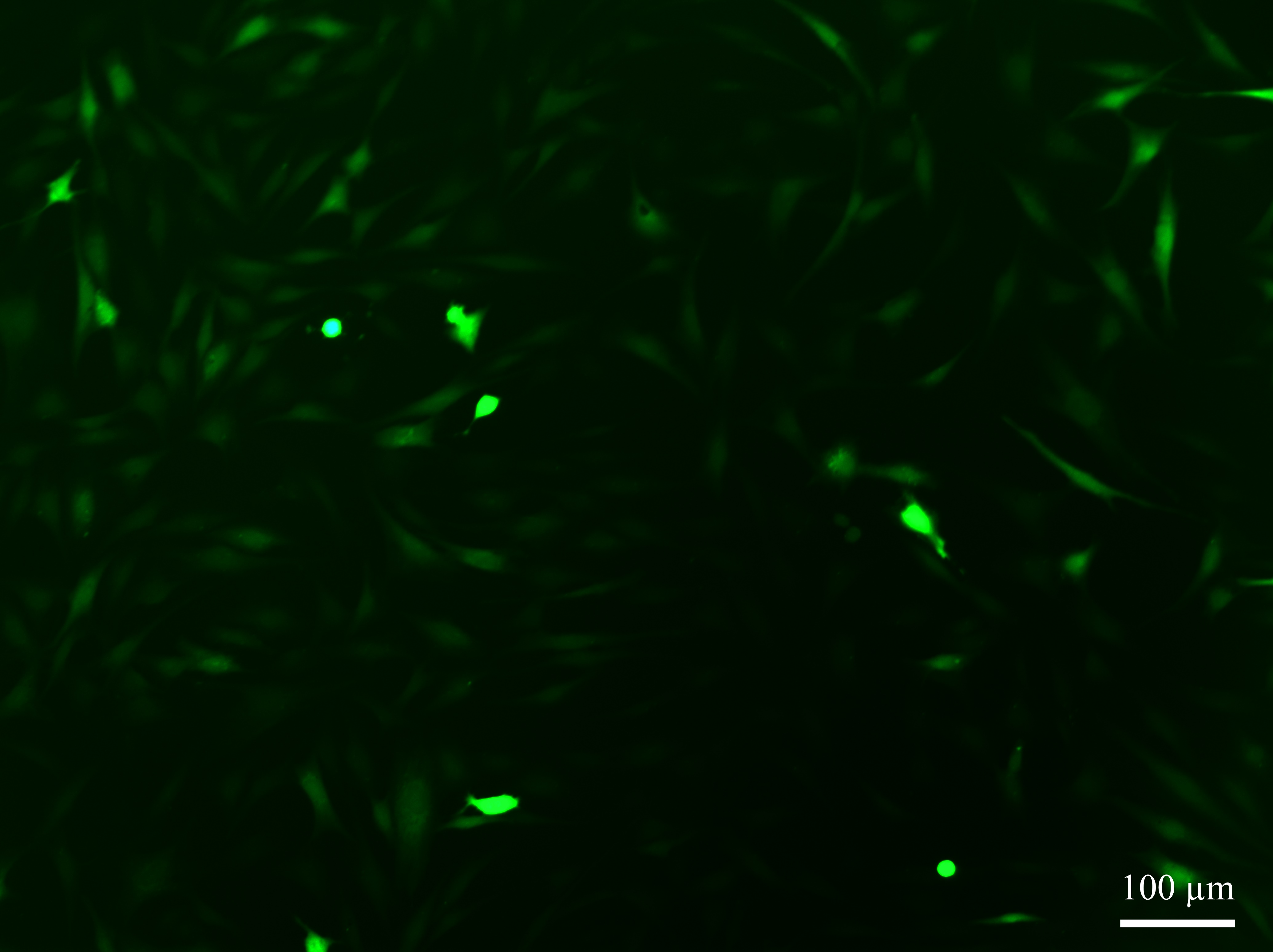

Supplement: S3 File — (ZIP) [file pone.0324264.s003.zip › supplement.material-3/ROS/144-Control2.jpg]

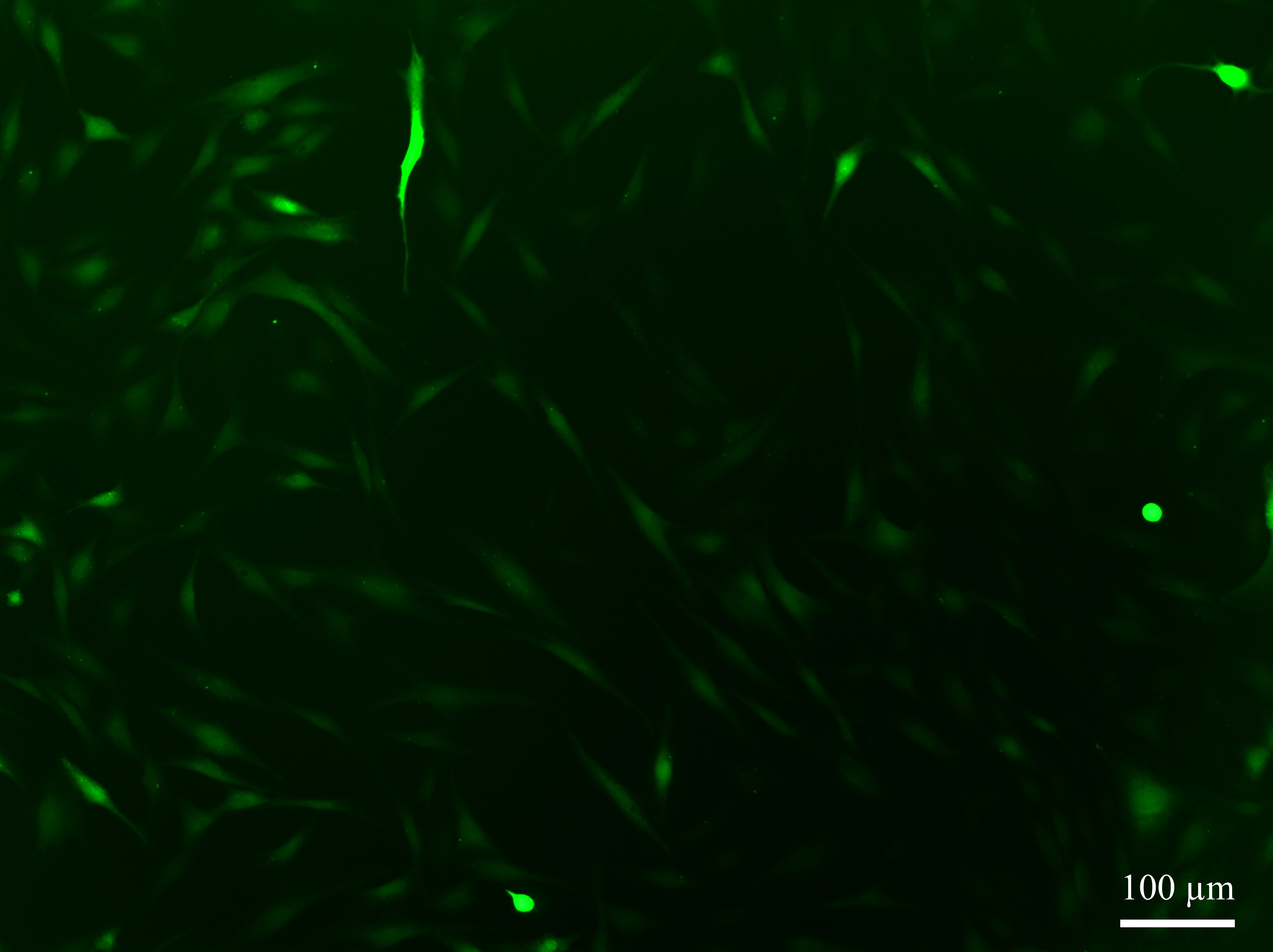

Supplement: S3 File — (ZIP) [file pone.0324264.s003.zip › supplement.material-3/ROS/144-Control3.jpg]

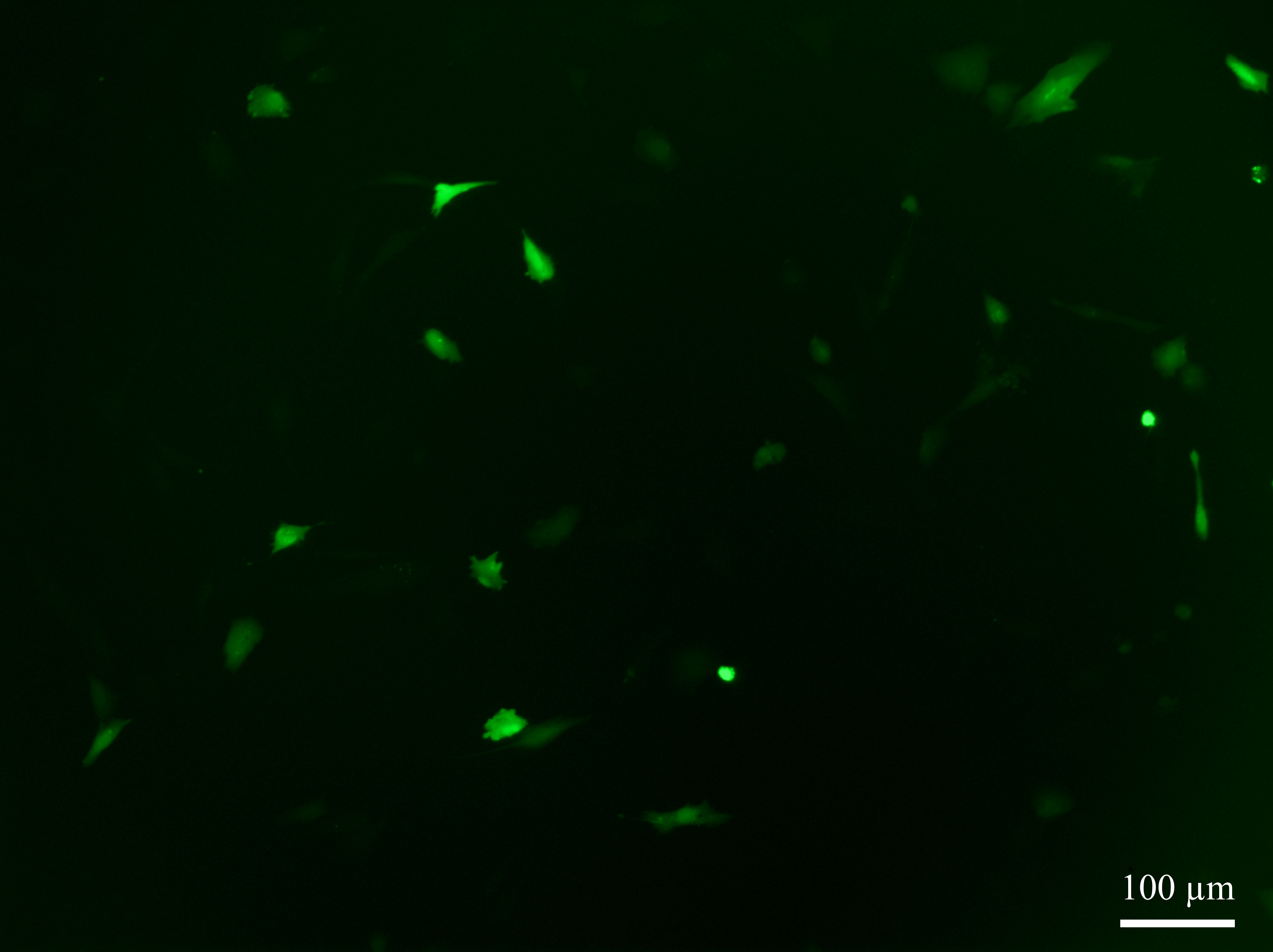

Supplement: S3 File — (ZIP) [file pone.0324264.s003.zip › supplement.material-3/ROS/144-Control4.jpg]

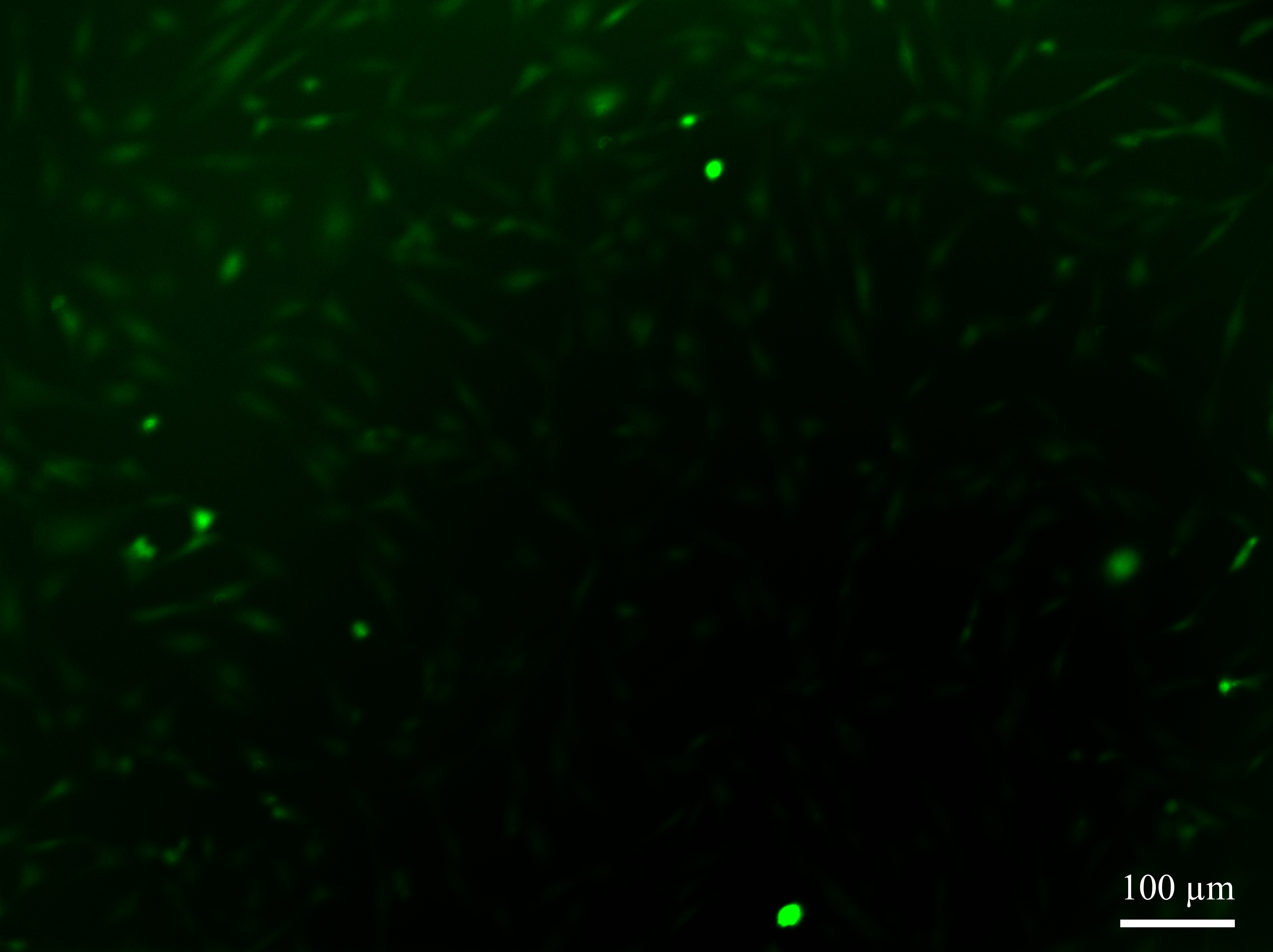

Supplement: S3 File — (ZIP) [file pone.0324264.s003.zip › supplement.material-3/ROS/144-Control5.jpg]

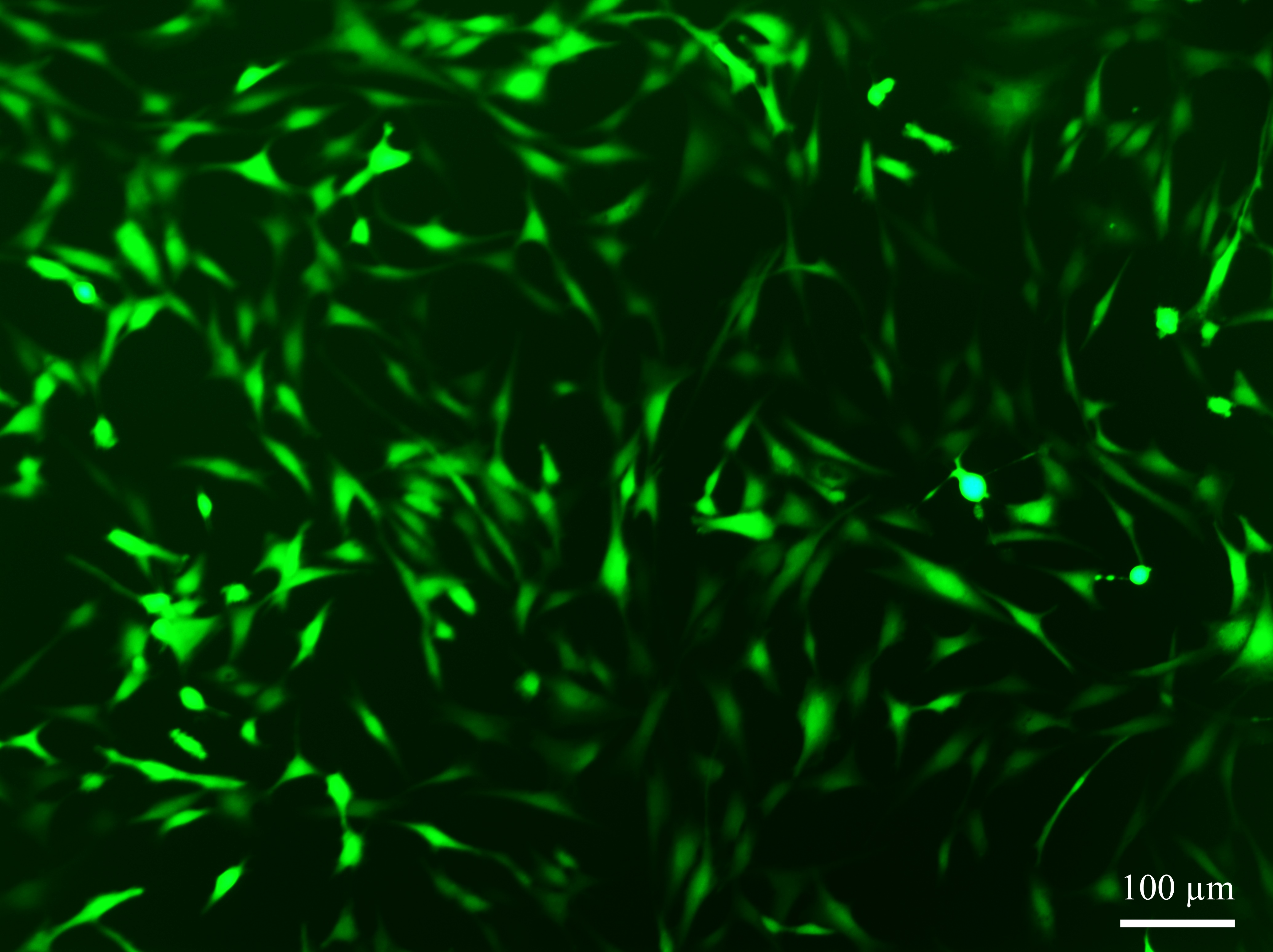

Supplement: S3 File — (ZIP) [file pone.0324264.s003.zip › supplement.material-3/ROS/144-Model1.jpg]

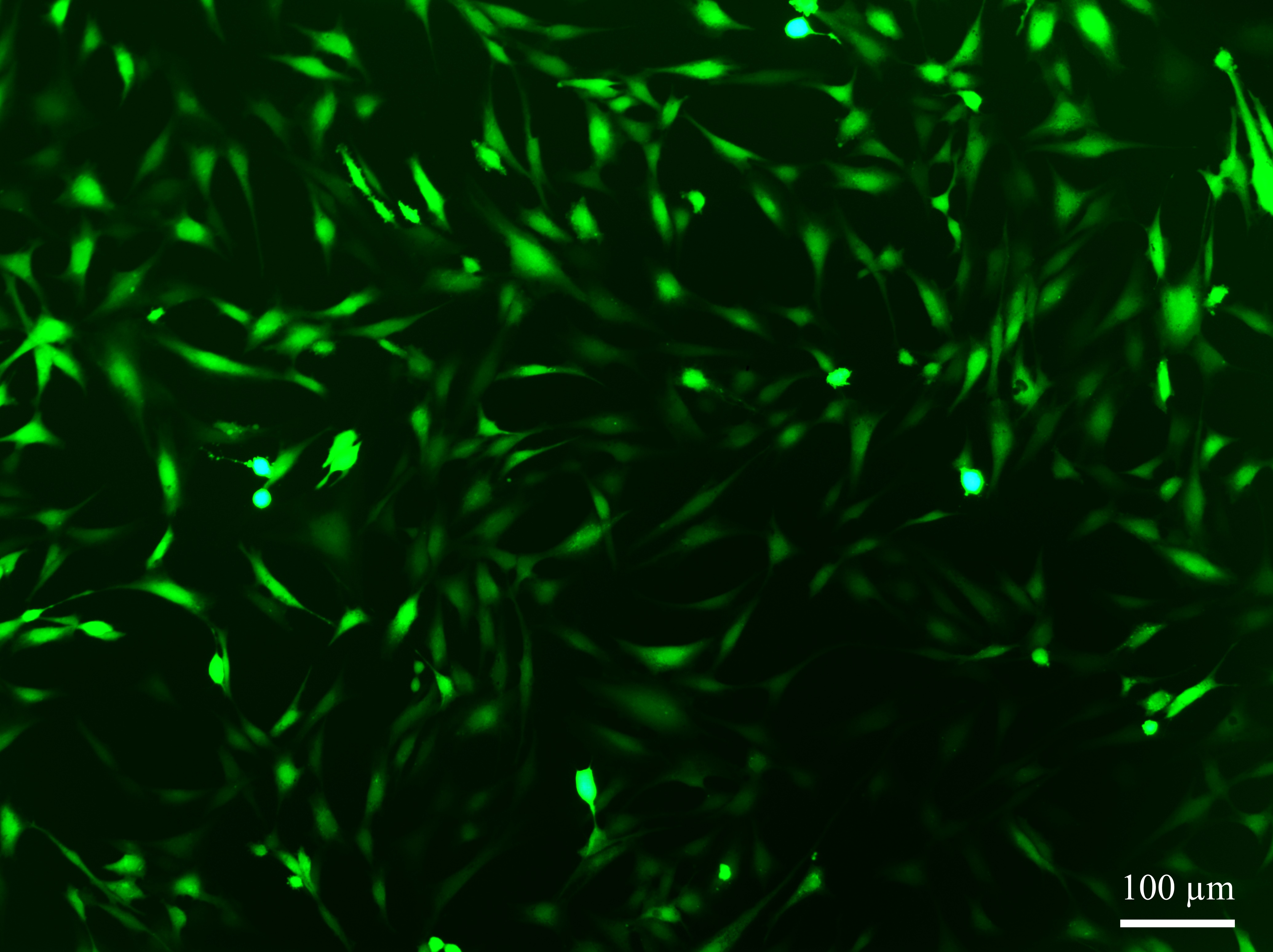

Supplement: S3 File — (ZIP) [file pone.0324264.s003.zip › supplement.material-3/ROS/144-Model2.jpg]

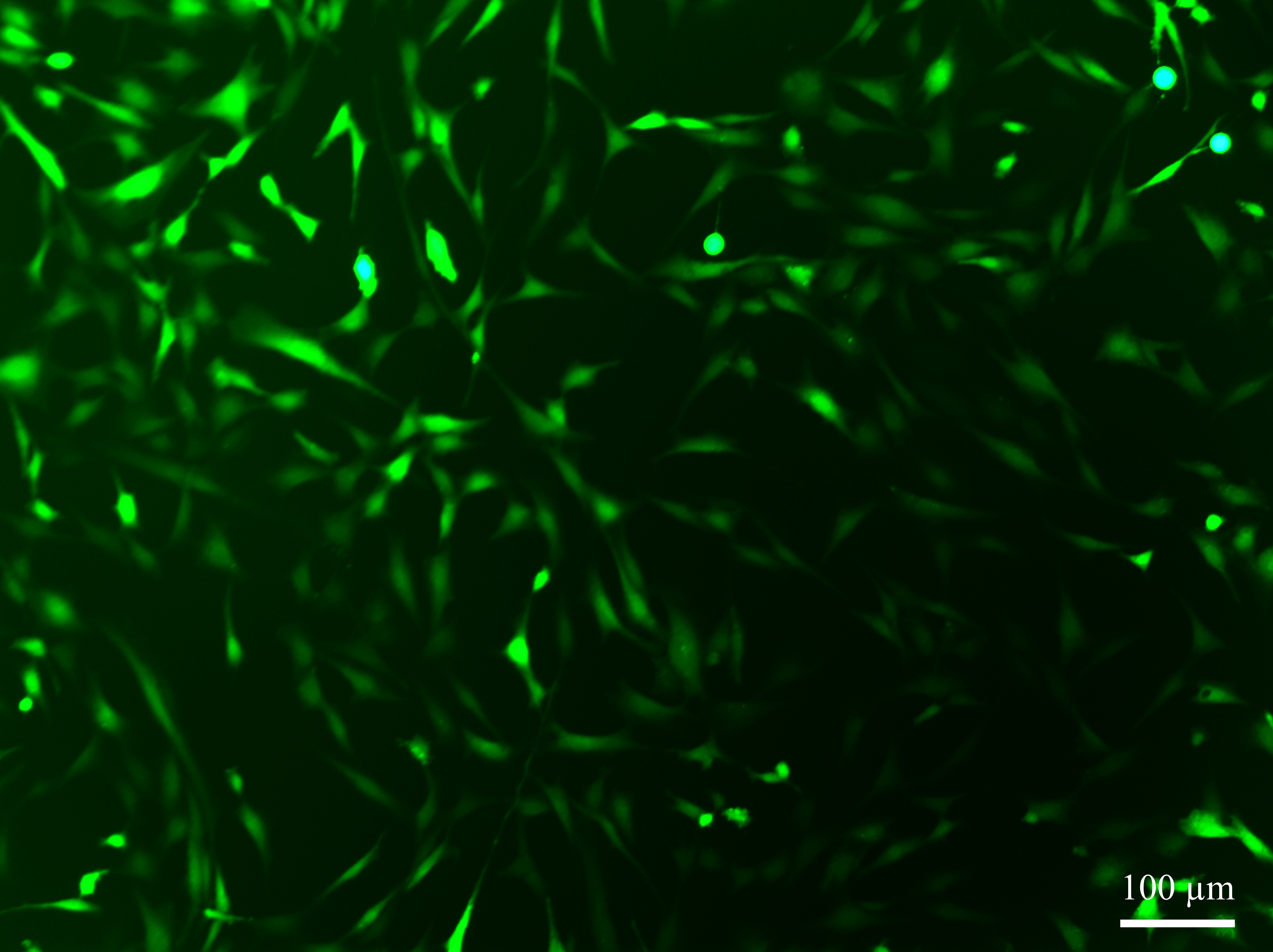

Supplement: S3 File — (ZIP) [file pone.0324264.s003.zip › supplement.material-3/ROS/144-Model3.jpg]

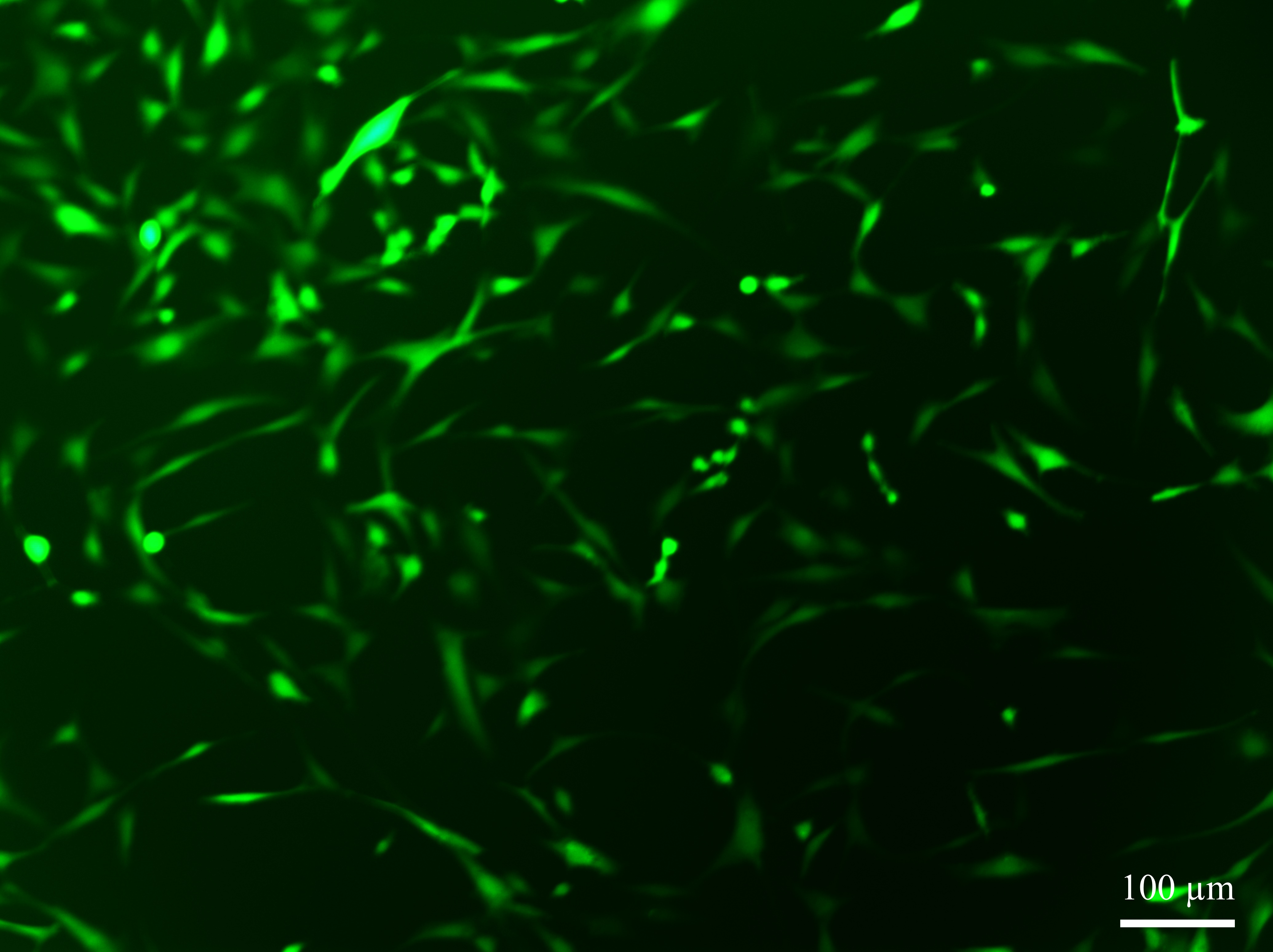

Supplement: S3 File — (ZIP) [file pone.0324264.s003.zip › supplement.material-3/ROS/144-Model4.jpg]

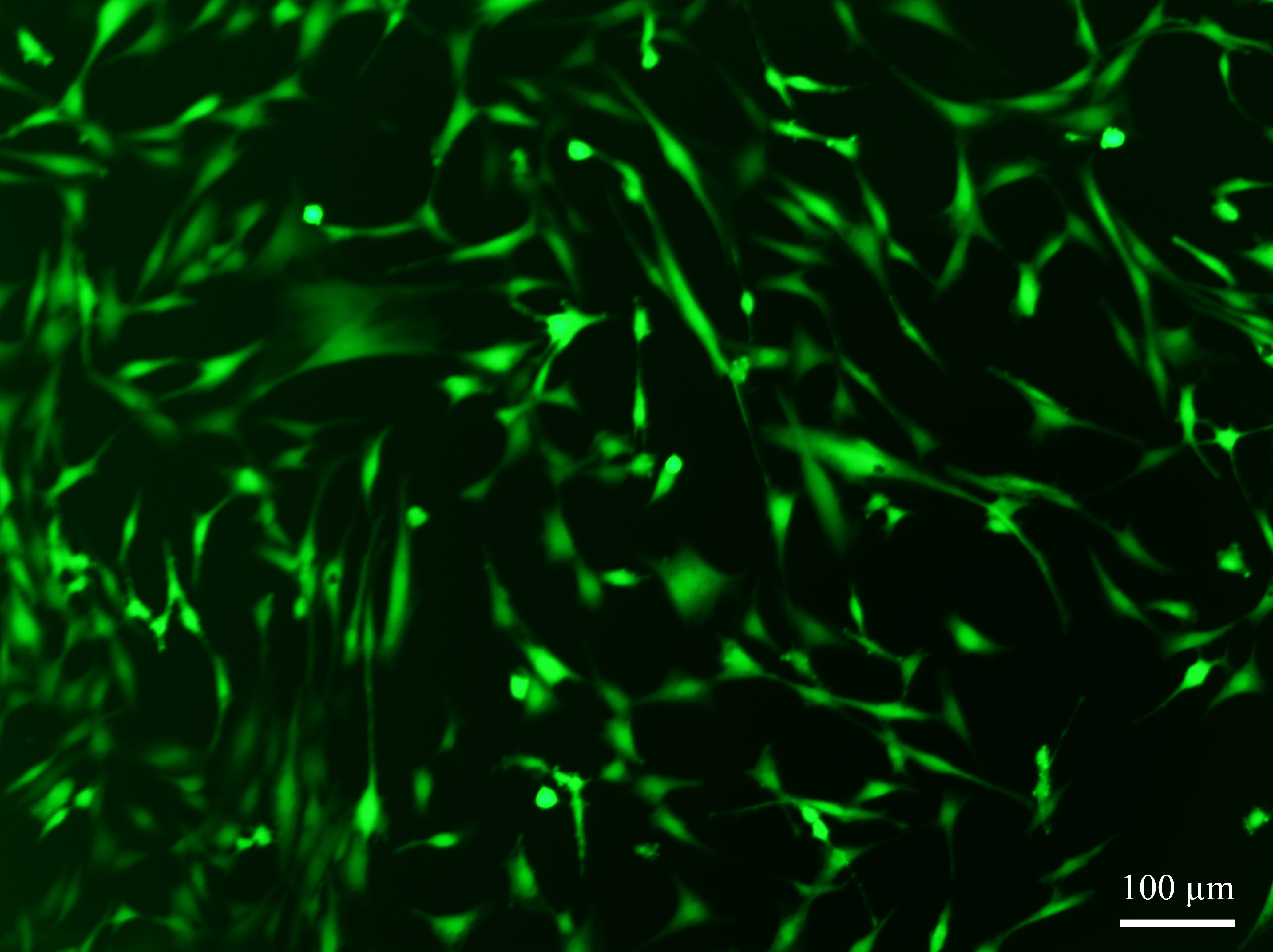

Supplement: S3 File — (ZIP) [file pone.0324264.s003.zip › supplement.material-3/ROS/144-Model5.jpg]

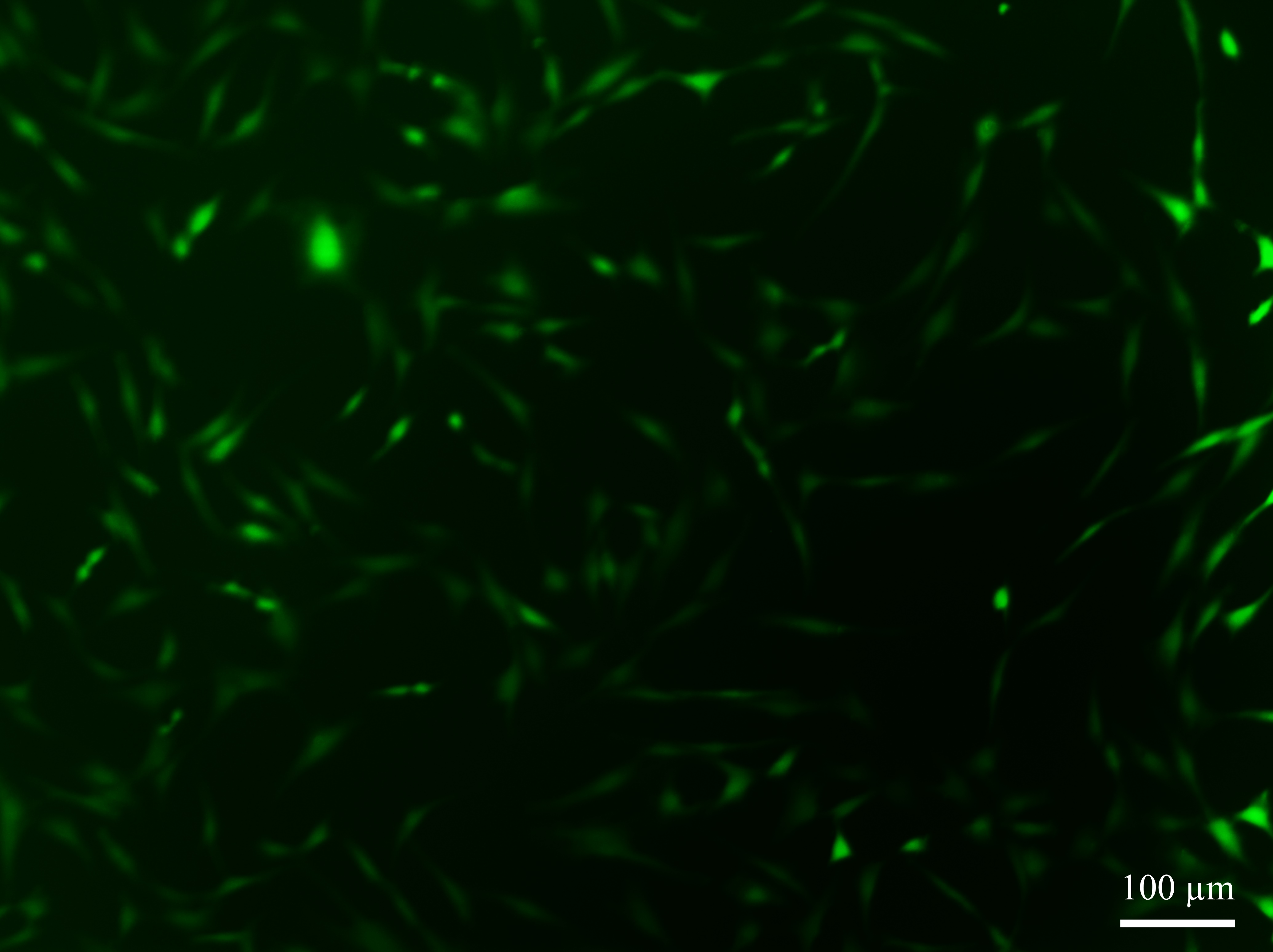

Supplement: S3 File — (ZIP) [file pone.0324264.s003.zip › supplement.material-3/ROS/144-PL1.jpg]

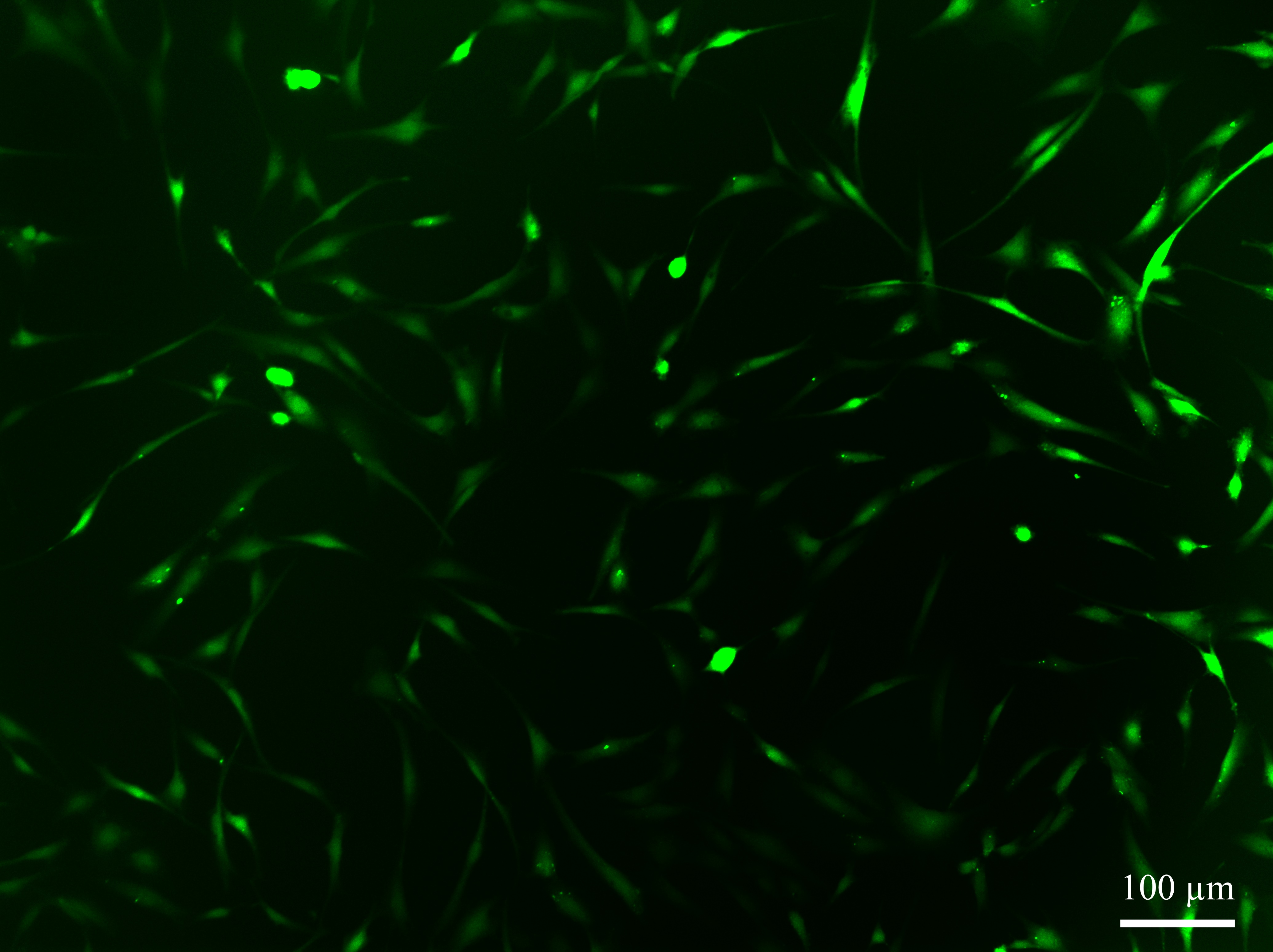

Supplement: S3 File — (ZIP) [file pone.0324264.s003.zip › supplement.material-3/ROS/144-PL2.jpg]

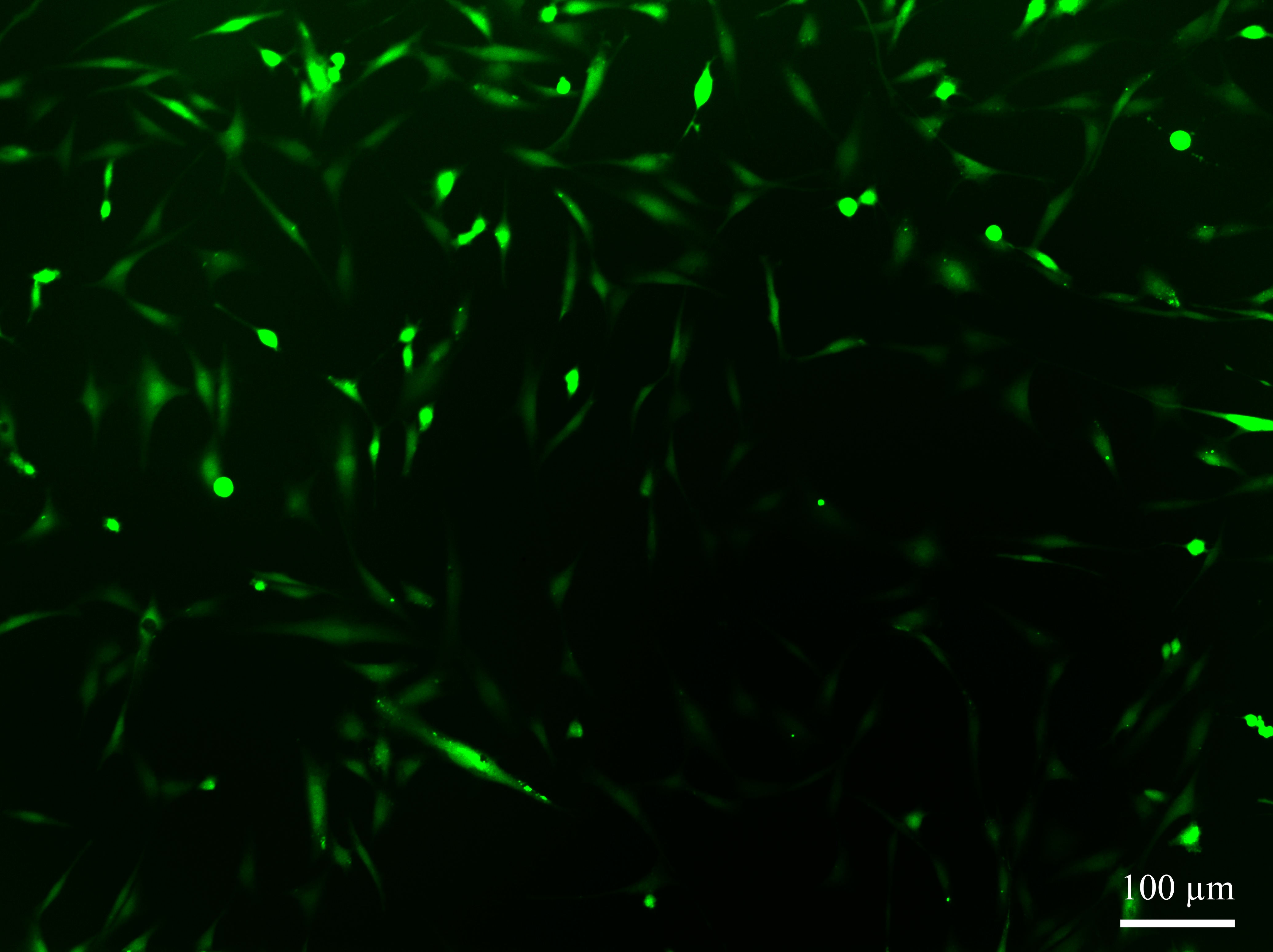

Supplement: S3 File — (ZIP) [file pone.0324264.s003.zip › supplement.material-3/ROS/144-PL3.jpg]

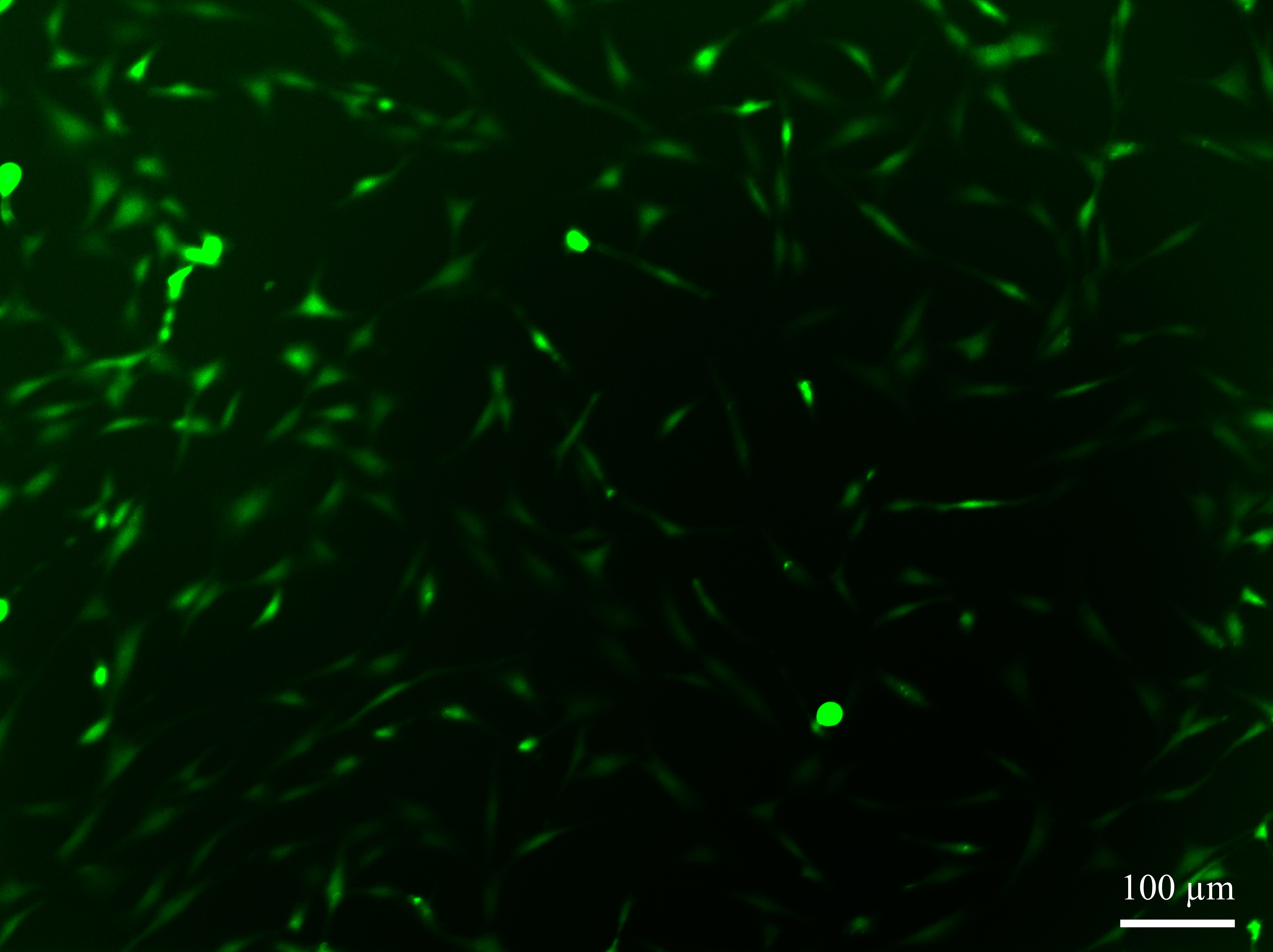

Supplement: S3 File — (ZIP) [file pone.0324264.s003.zip › supplement.material-3/ROS/144-PL4.jpg]

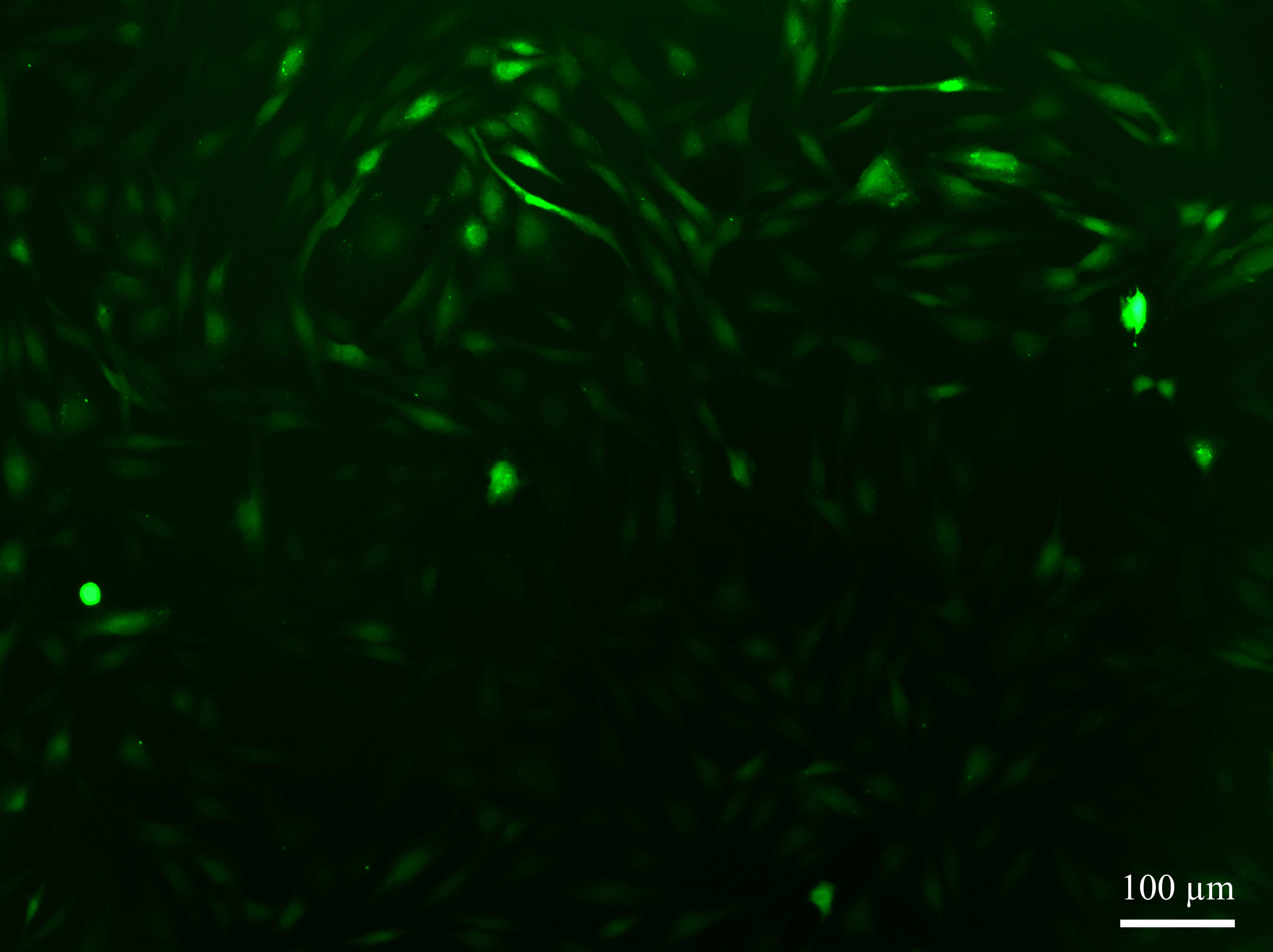

Supplement: S3 File — (ZIP) [file pone.0324264.s003.zip › supplement.material-3/ROS/144-PL5.jpg]

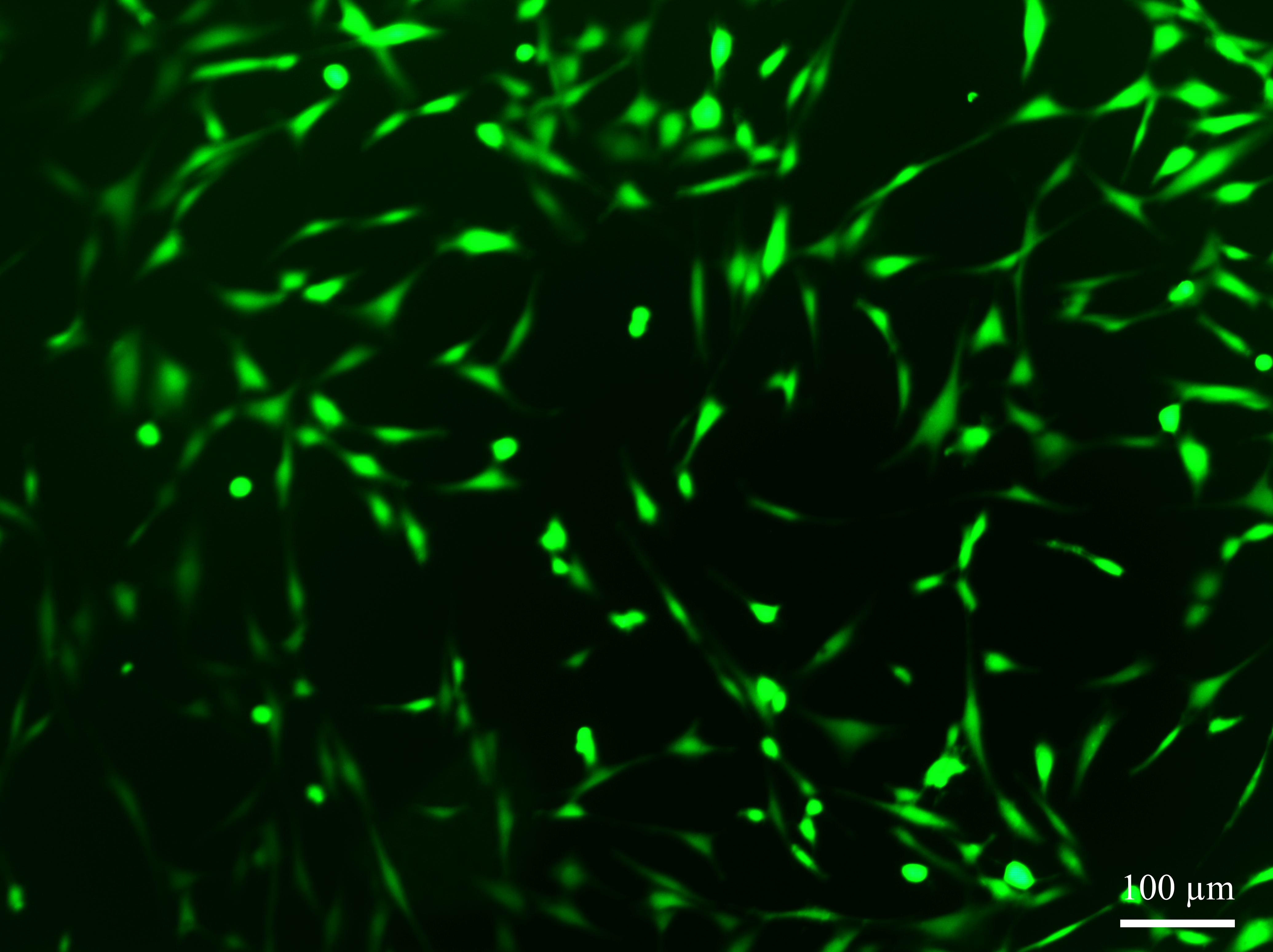

Supplement: S3 File — (ZIP) [file pone.0324264.s003.zip › supplement.material-3/ROS/48--Control1.jpg]

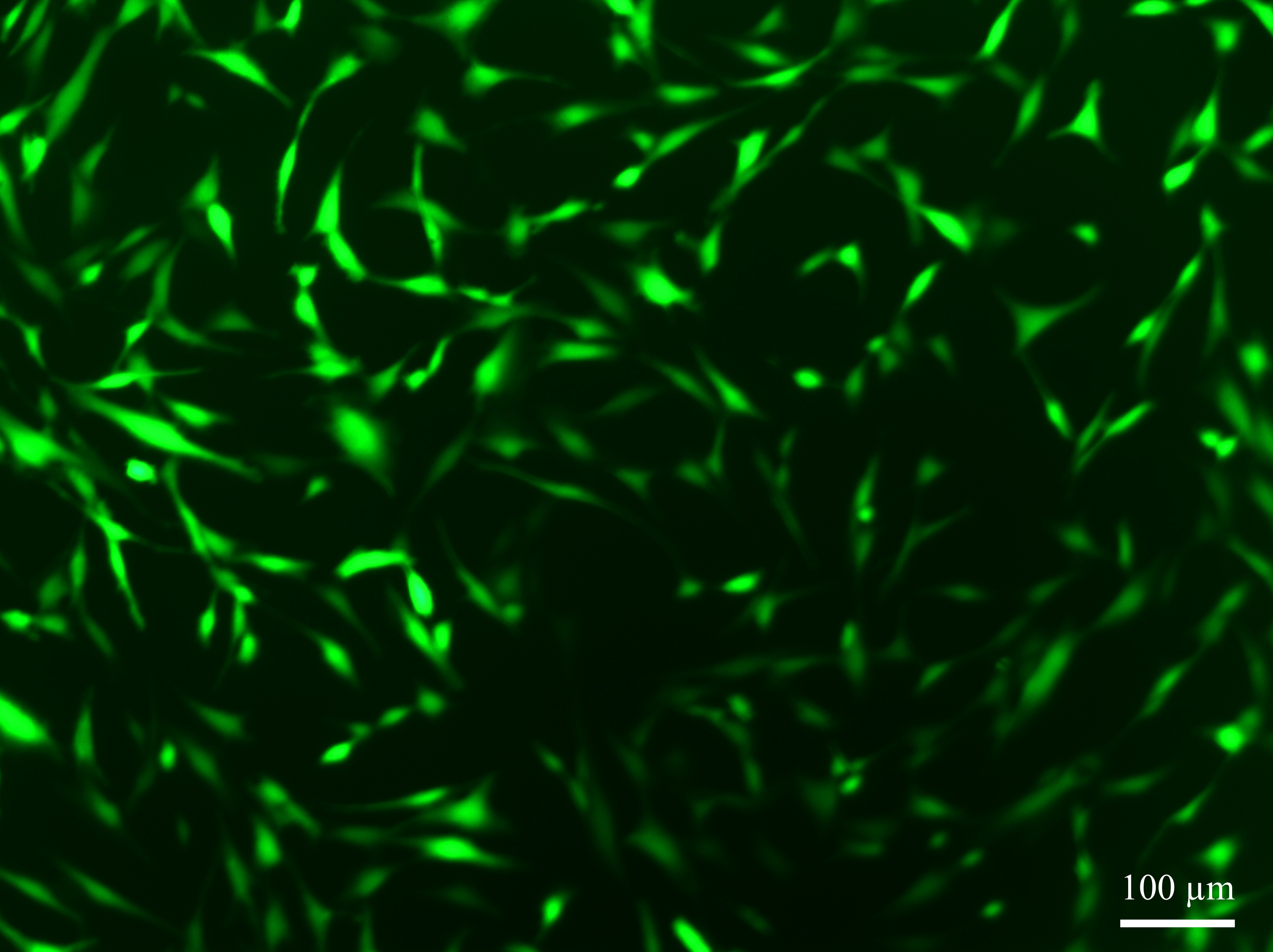

Supplement: S3 File — (ZIP) [file pone.0324264.s003.zip › supplement.material-3/ROS/48--Control2.jpg]

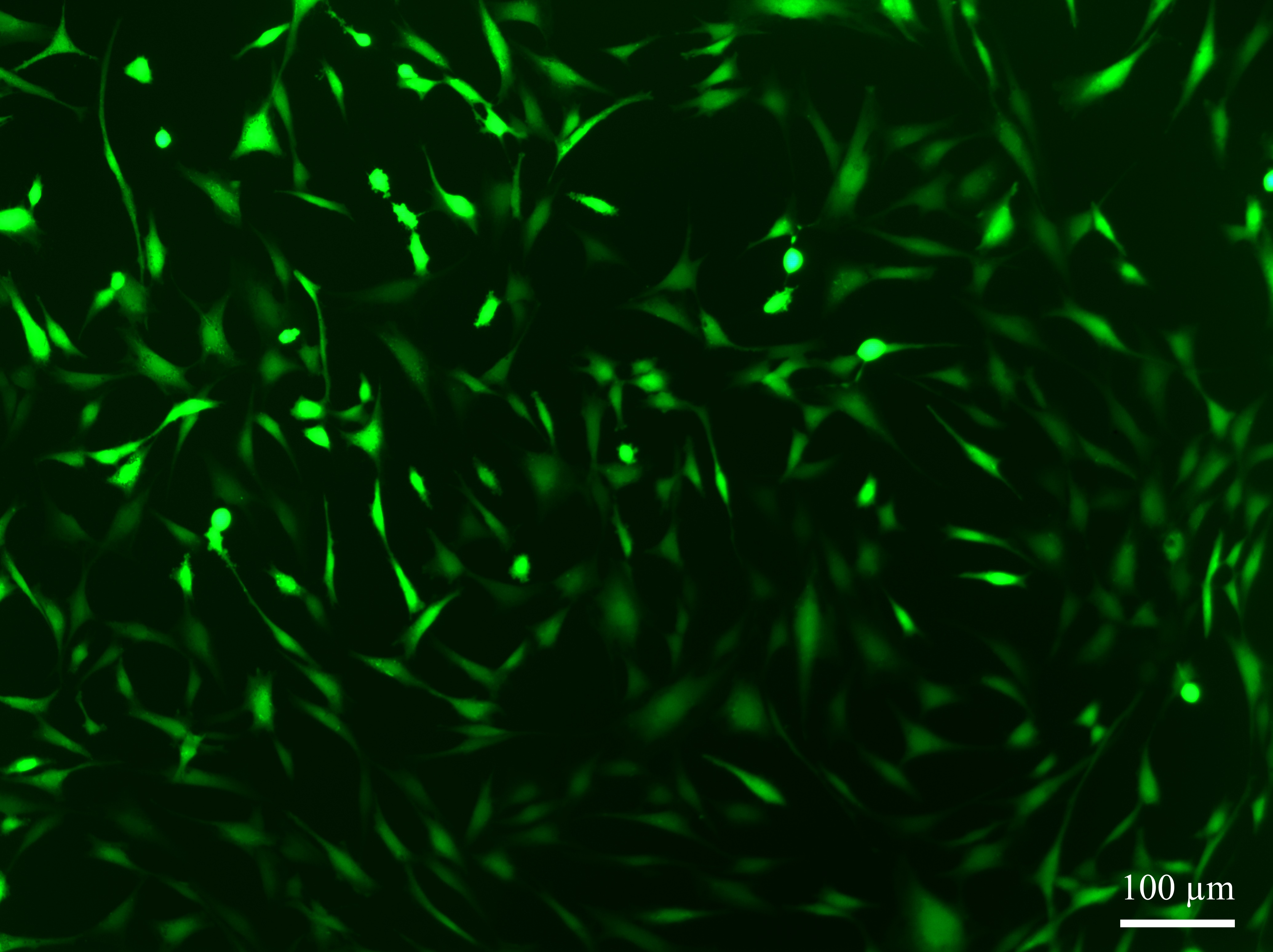

Supplement: S3 File — (ZIP) [file pone.0324264.s003.zip › supplement.material-3/ROS/48--Control3.jpg]

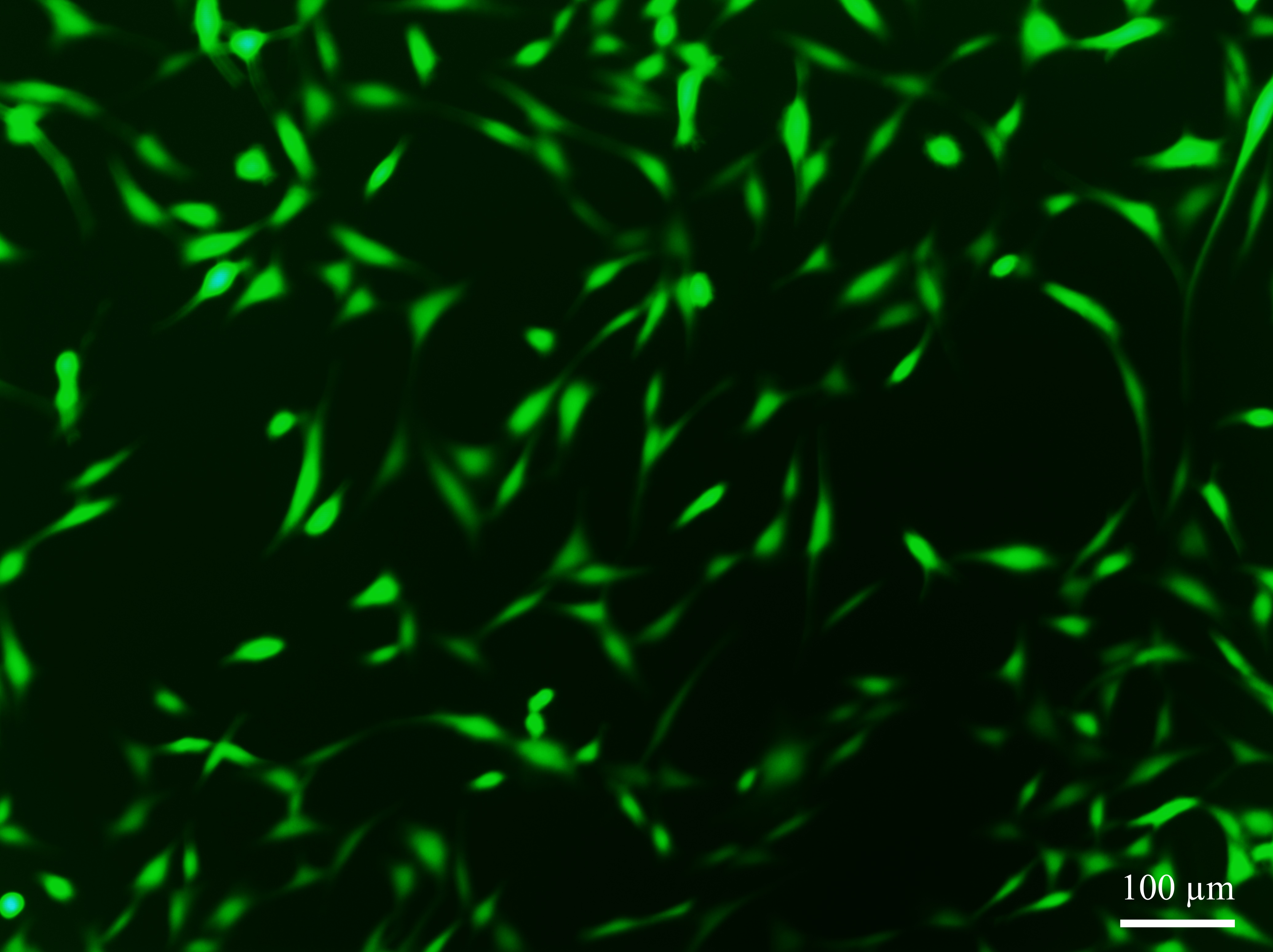

Supplement: S3 File — (ZIP) [file pone.0324264.s003.zip › supplement.material-3/ROS/48--Control4.jpg]

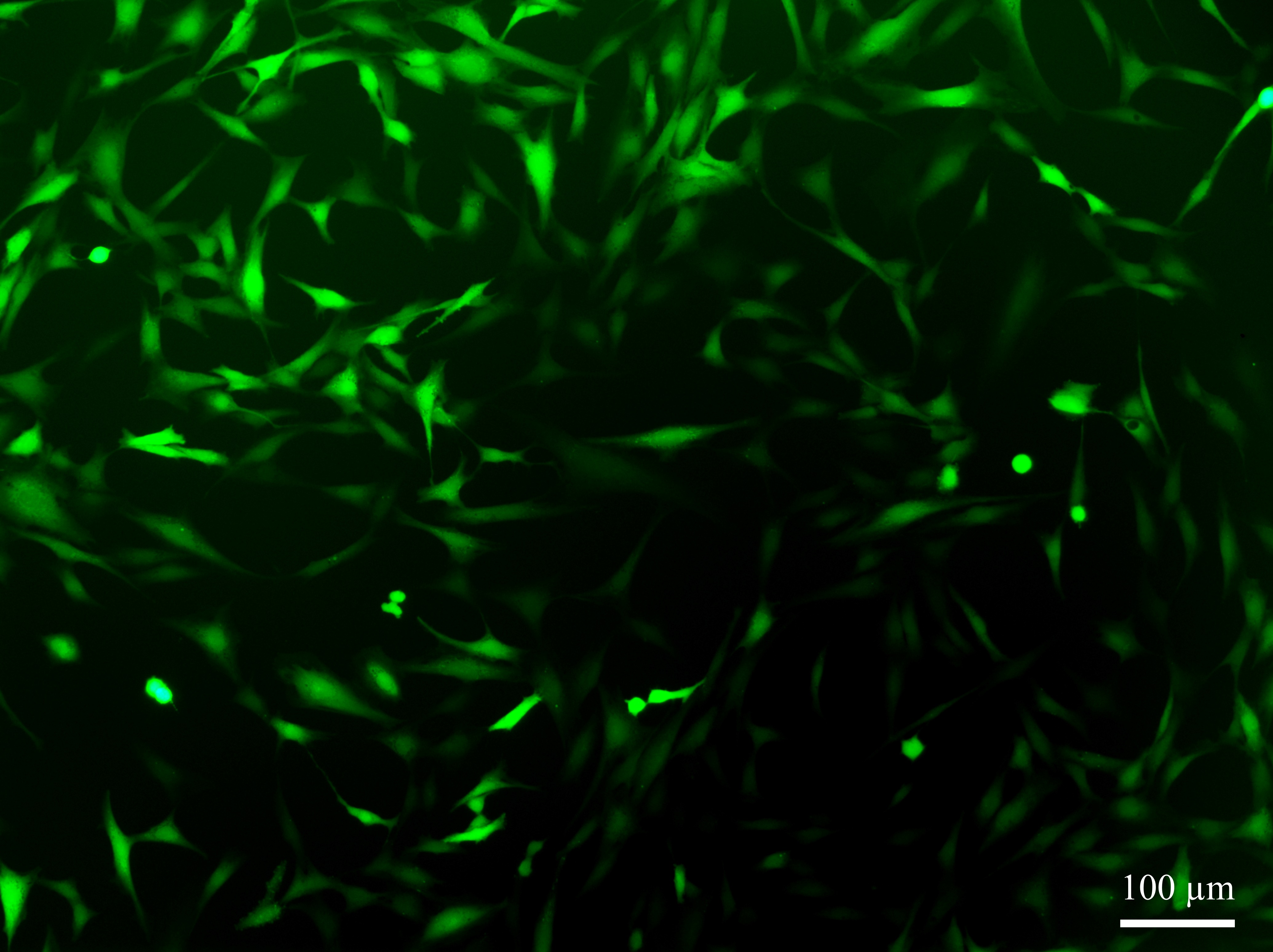

Supplement: S3 File — (ZIP) [file pone.0324264.s003.zip › supplement.material-3/ROS/48--Control5.jpg]

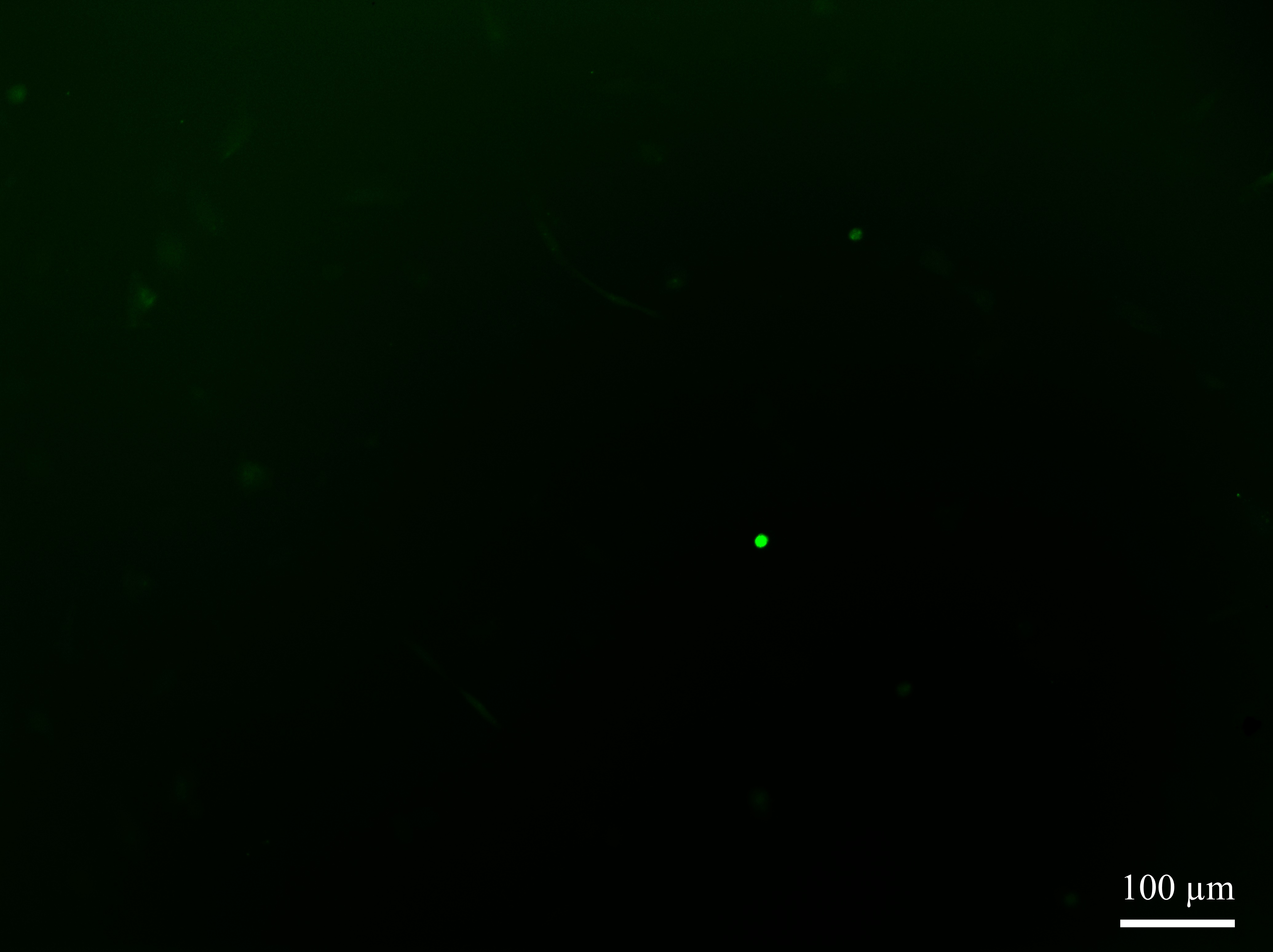

Supplement: S3 File — (ZIP) [file pone.0324264.s003.zip › supplement.material-3/ROS/48--Model1.jpg]

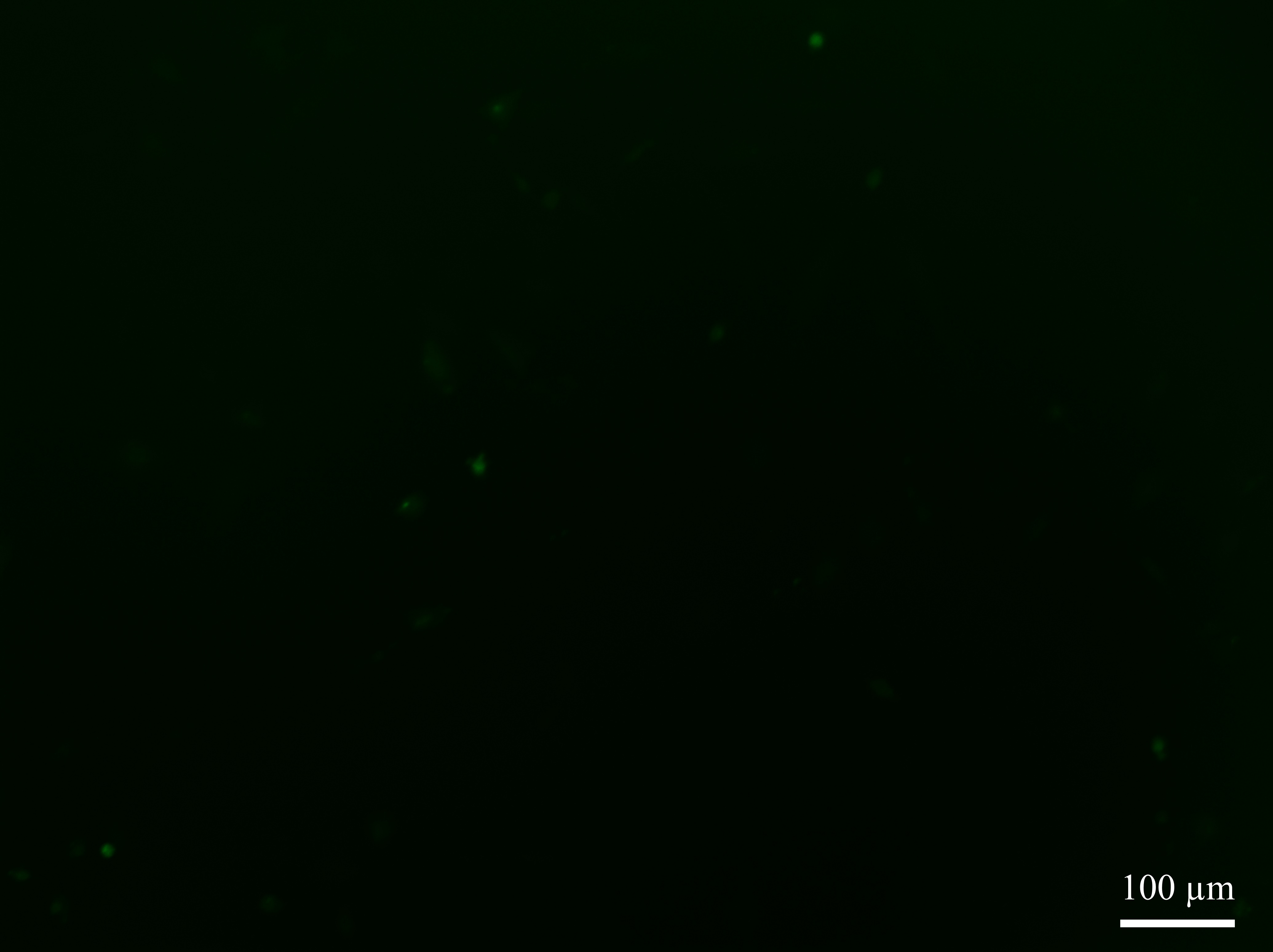

Supplement: S3 File — (ZIP) [file pone.0324264.s003.zip › supplement.material-3/ROS/48--Model2.jpg]

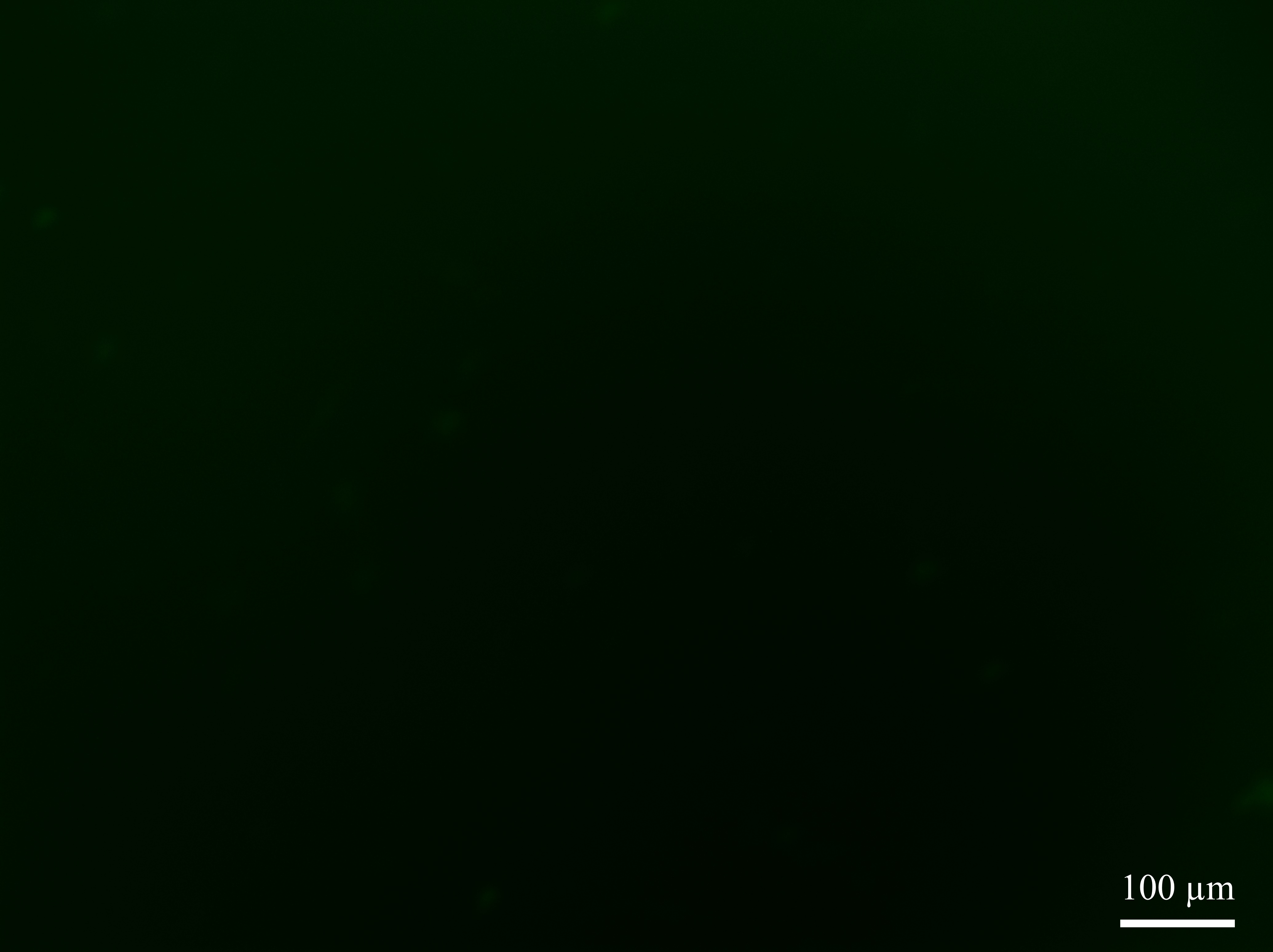

Supplement: S3 File — (ZIP) [file pone.0324264.s003.zip › supplement.material-3/ROS/48--Model3.jpg]

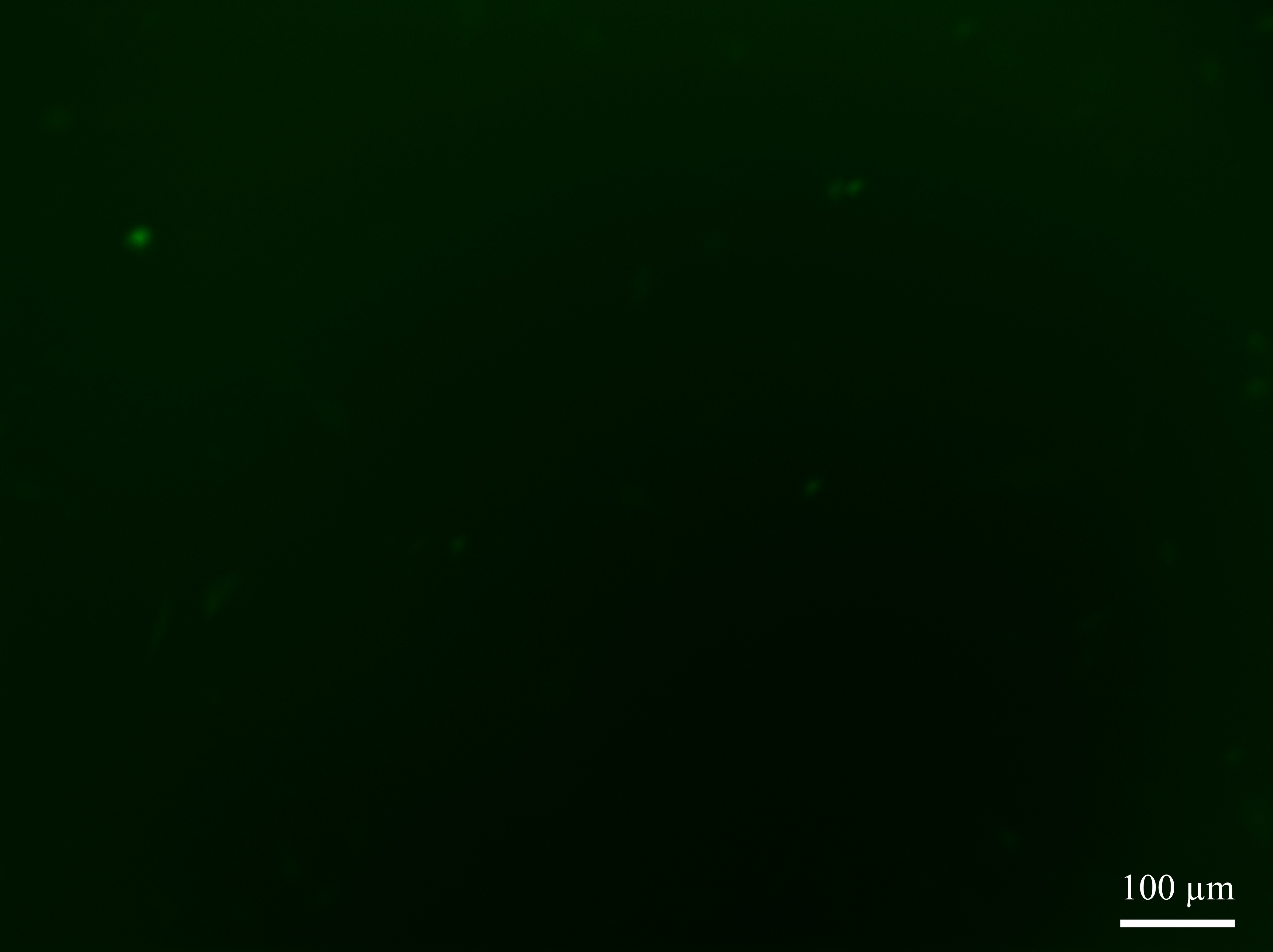

Supplement: S3 File — (ZIP) [file pone.0324264.s003.zip › supplement.material-3/ROS/48--Model4.jpg]

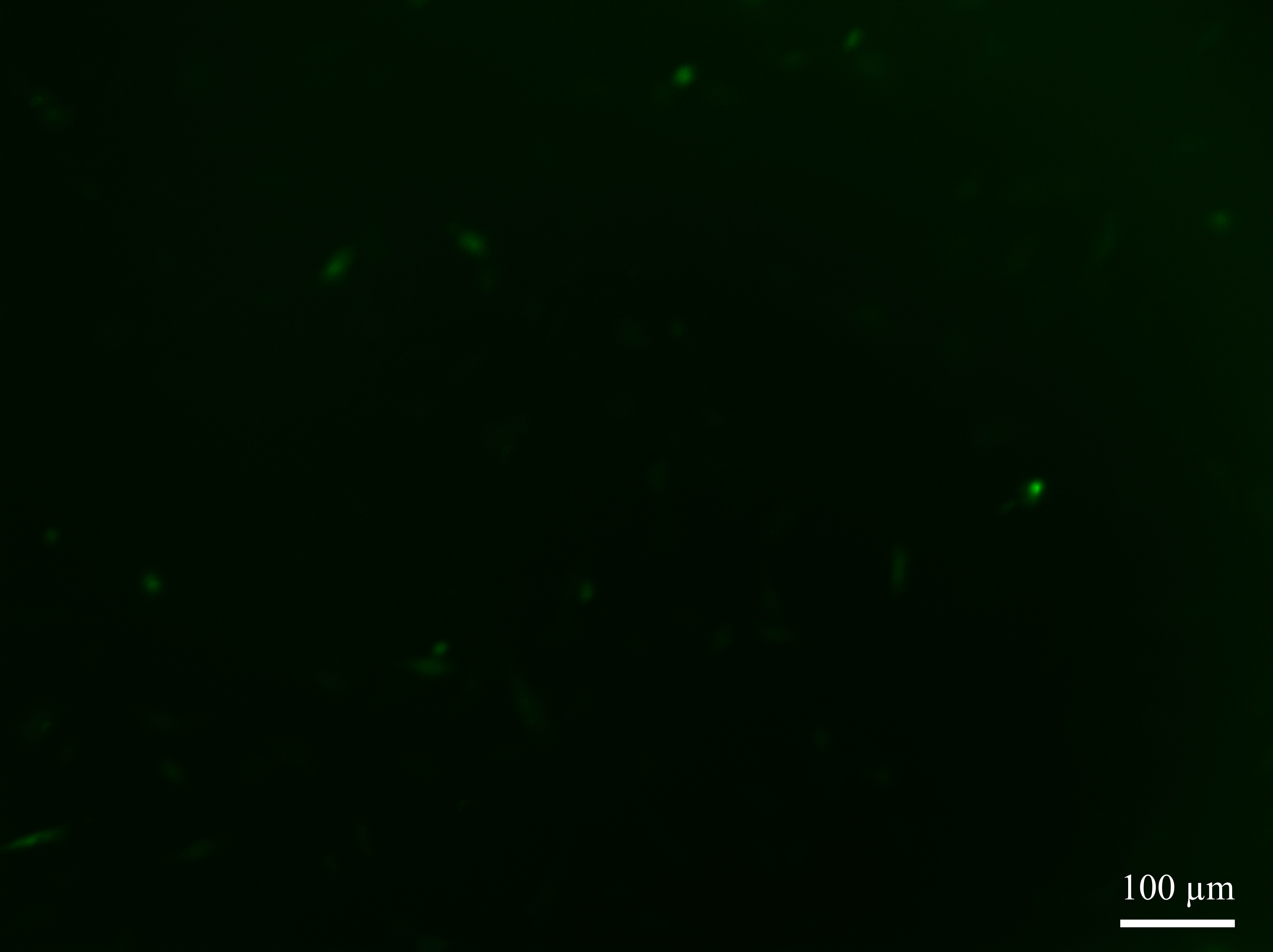

Supplement: S3 File — (ZIP) [file pone.0324264.s003.zip › supplement.material-3/ROS/48--Model5.jpg]

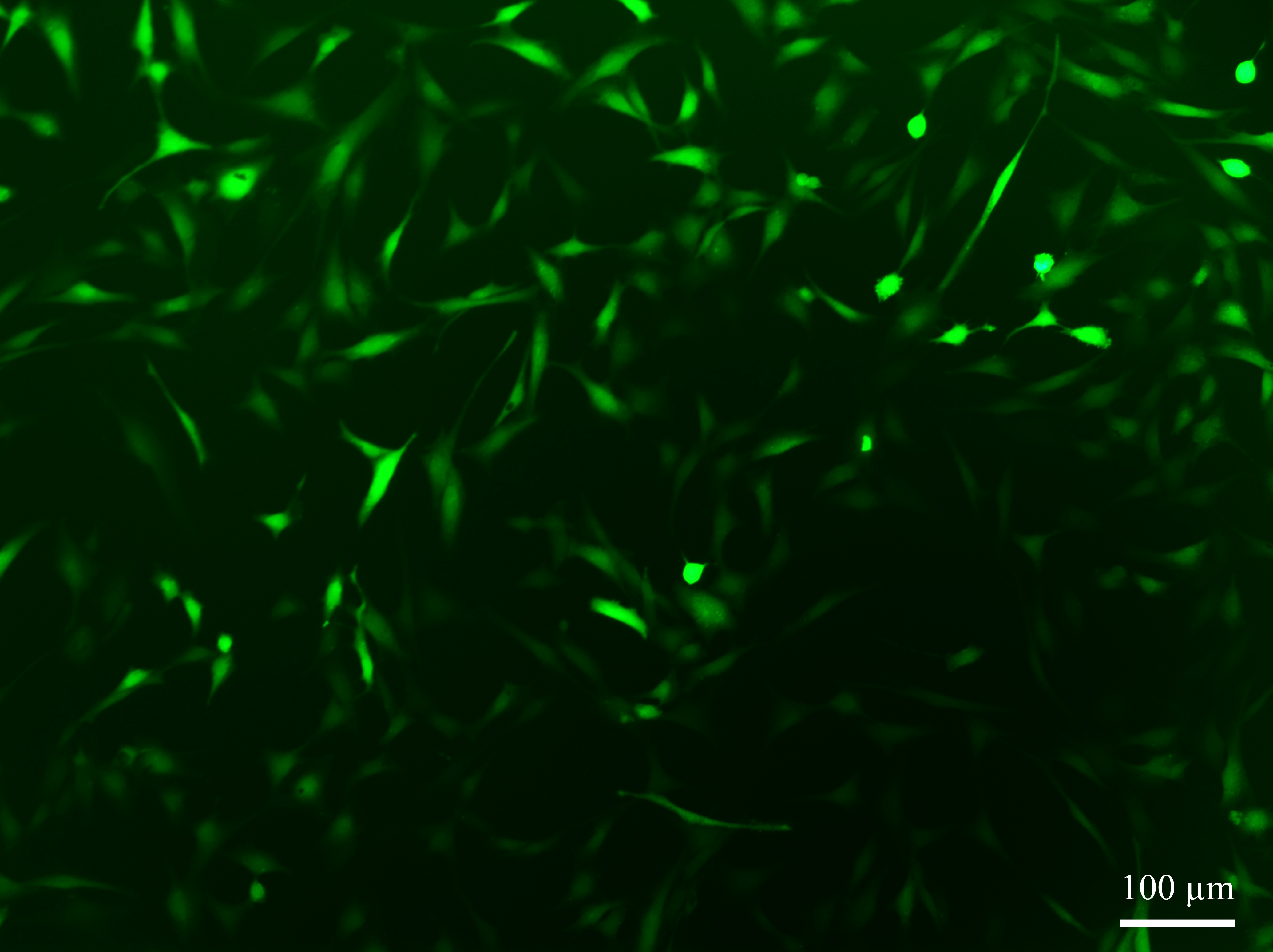

Supplement: S3 File — (ZIP) [file pone.0324264.s003.zip › supplement.material-3/ROS/48--PL1.jpg]

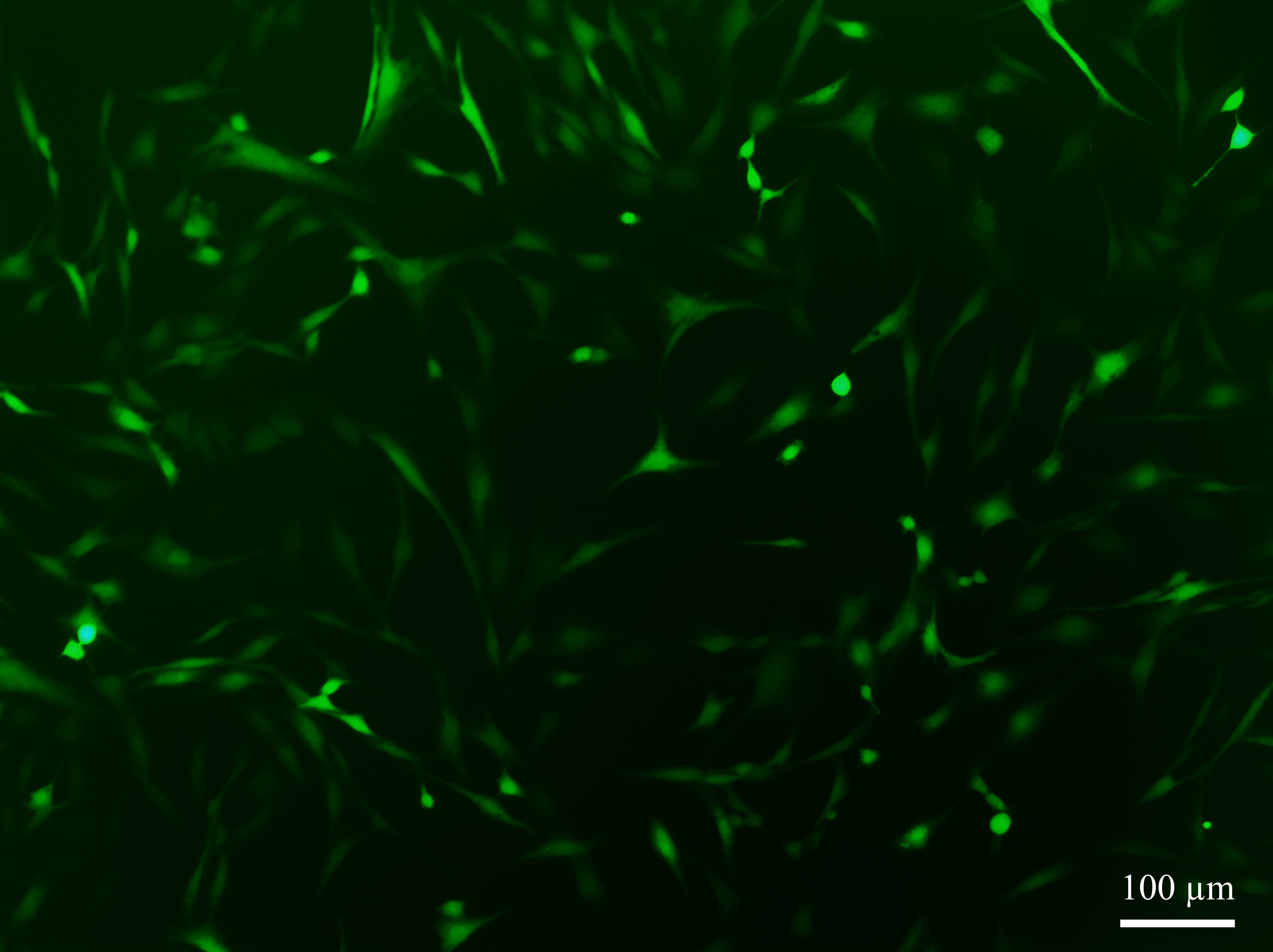

Supplement: S3 File — (ZIP) [file pone.0324264.s003.zip › supplement.material-3/ROS/48--PL2.jpg]

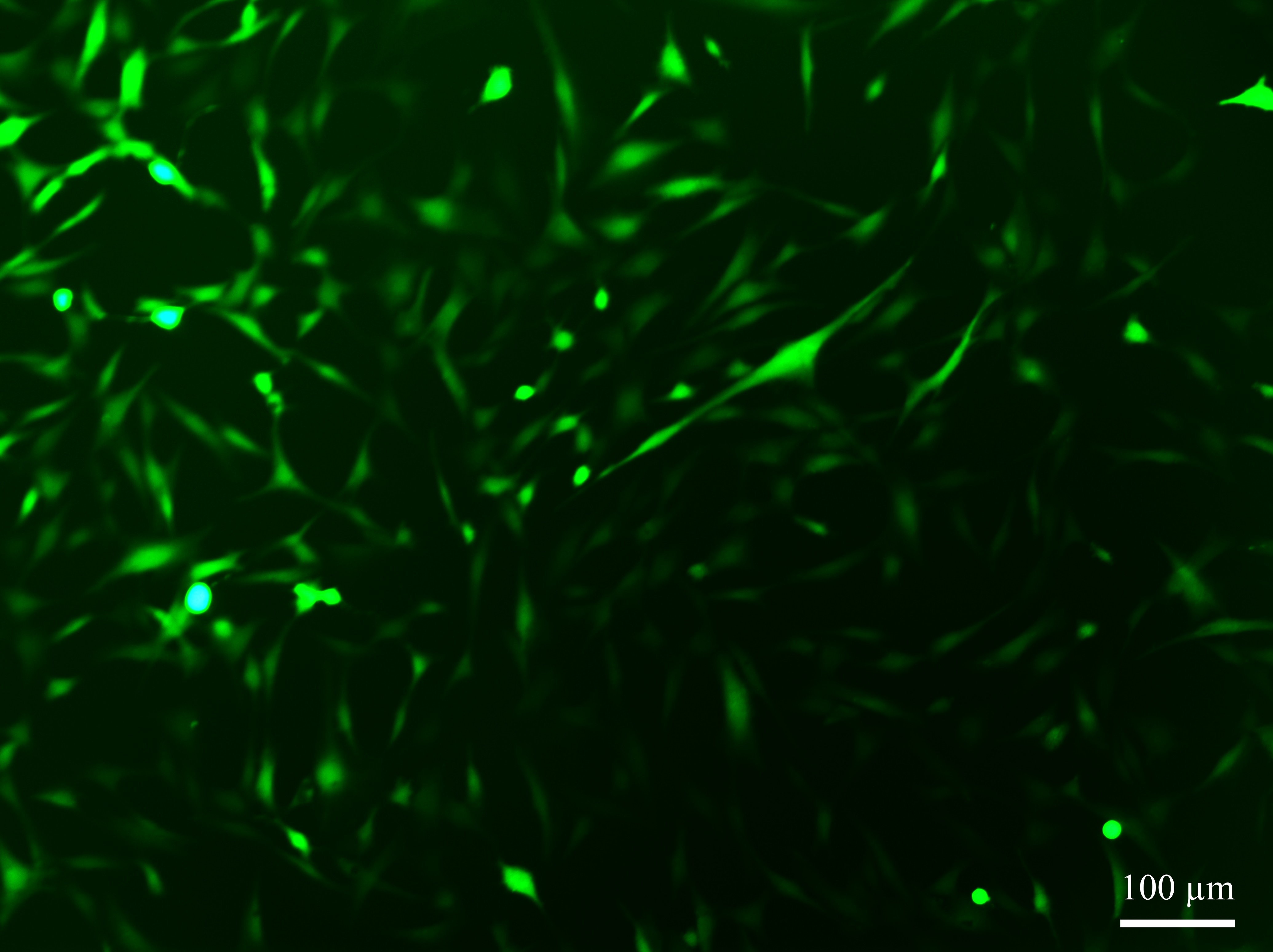

Supplement: S3 File — (ZIP) [file pone.0324264.s003.zip › supplement.material-3/ROS/48--PL3.jpg]

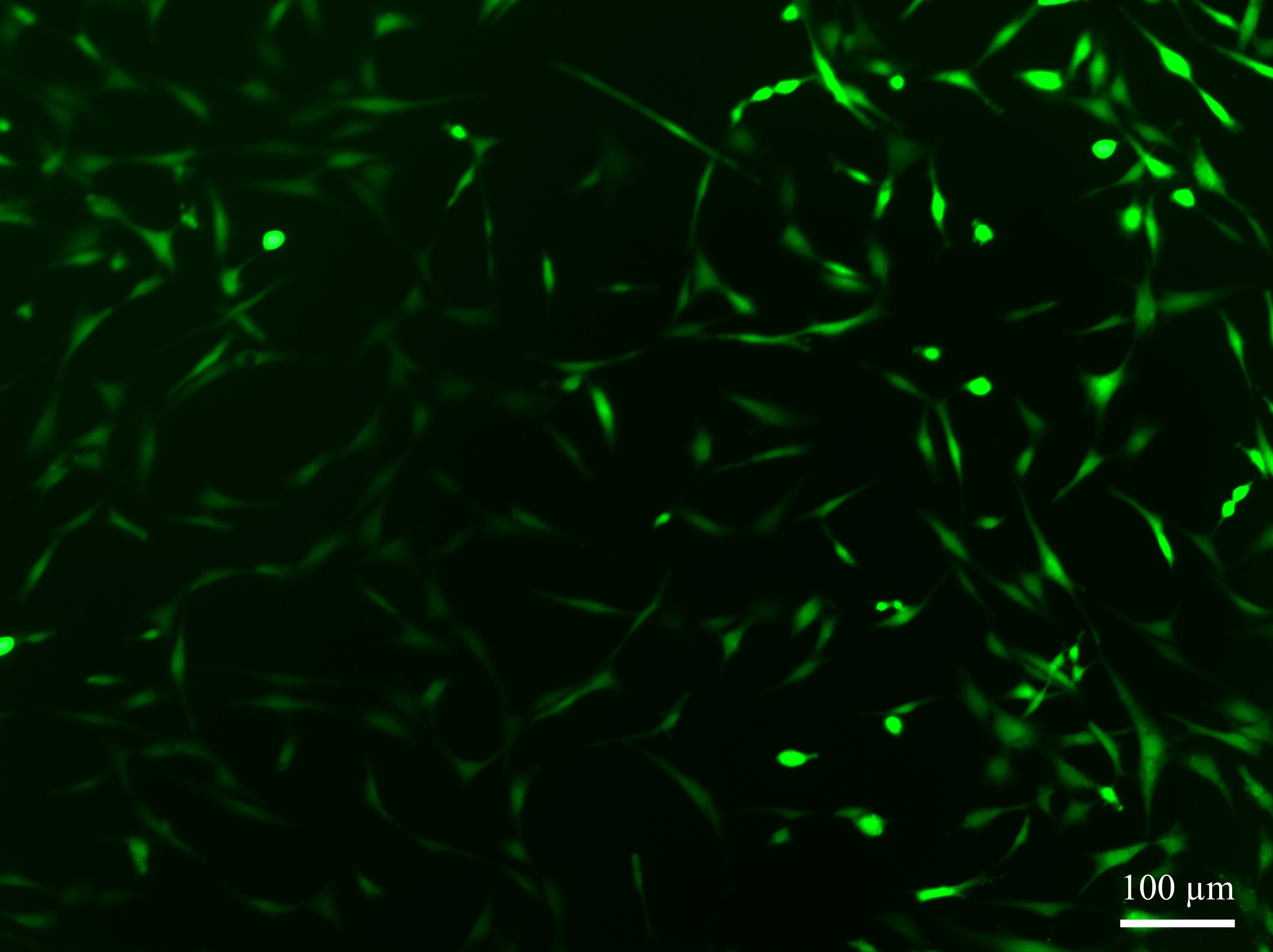

Supplement: S3 File — (ZIP) [file pone.0324264.s003.zip › supplement.material-3/ROS/48--PL4.jpg]

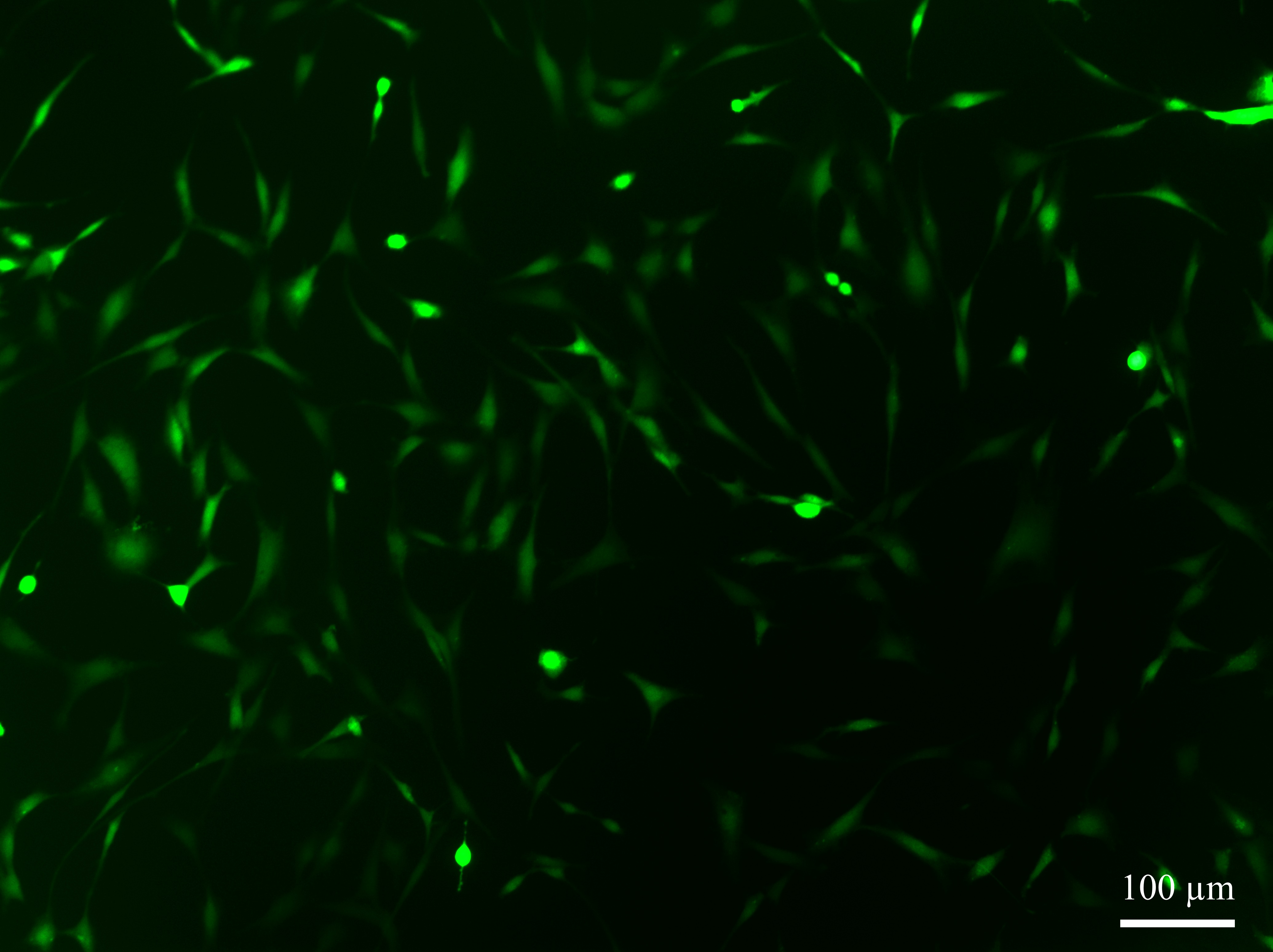

Supplement: S3 File — (ZIP) [file pone.0324264.s003.zip › supplement.material-3/ROS/48--PL5.jpg]

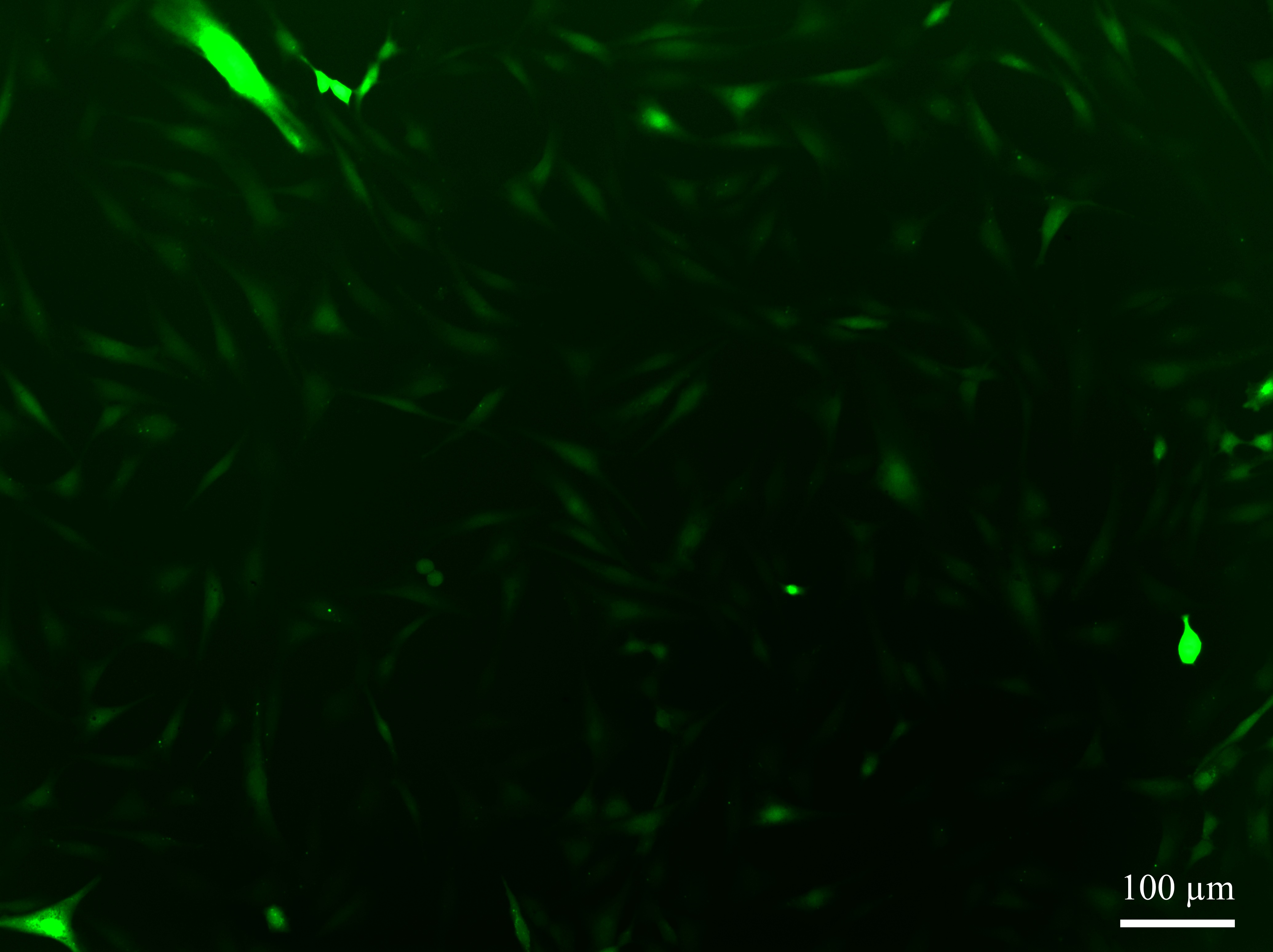

Supplement: S3 File — (ZIP) [file pone.0324264.s003.zip › supplement.material-3/ROS/96-Control1.jpg]

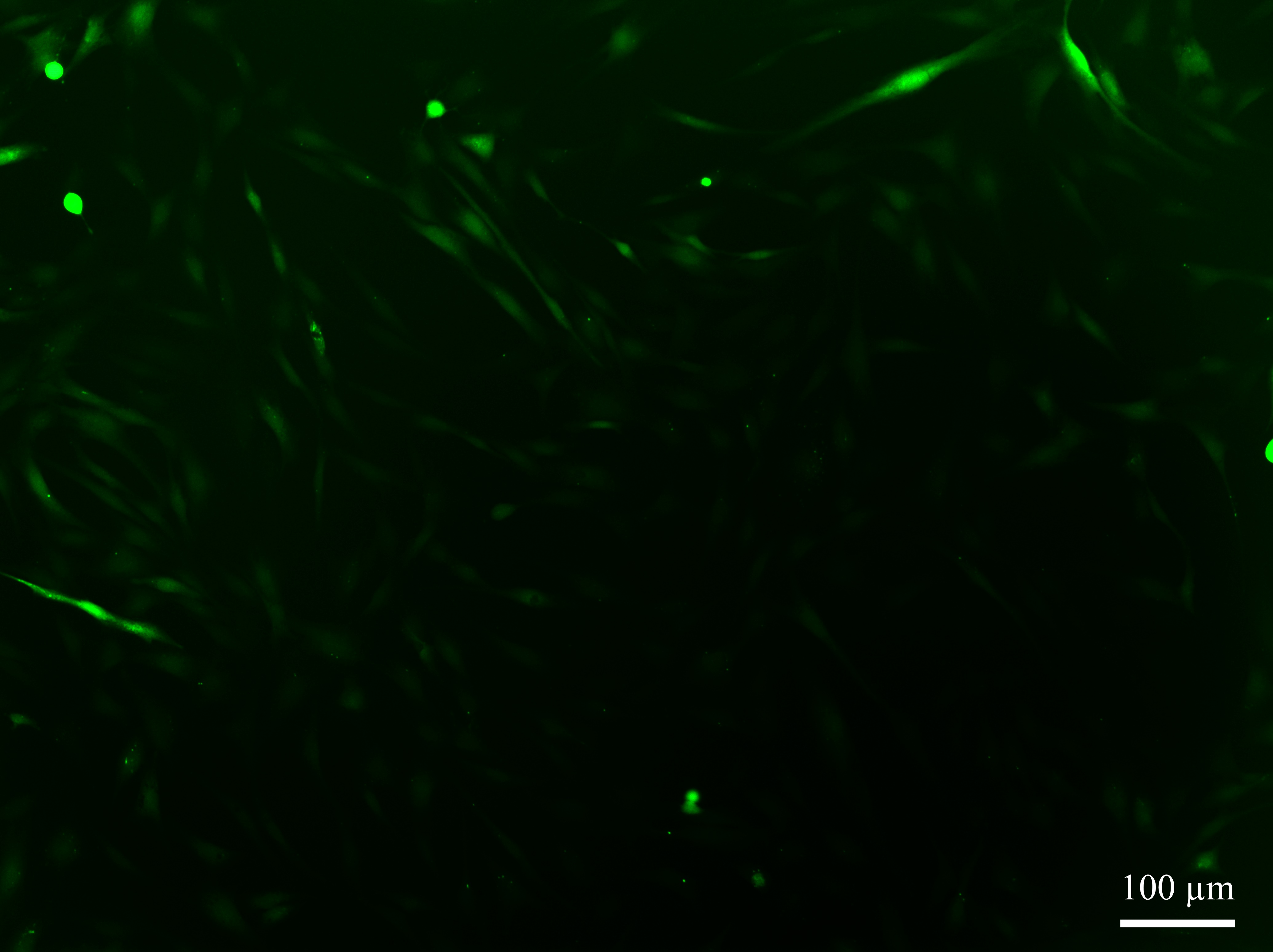

Supplement: S3 File — (ZIP) [file pone.0324264.s003.zip › supplement.material-3/ROS/96-Control2.jpg]

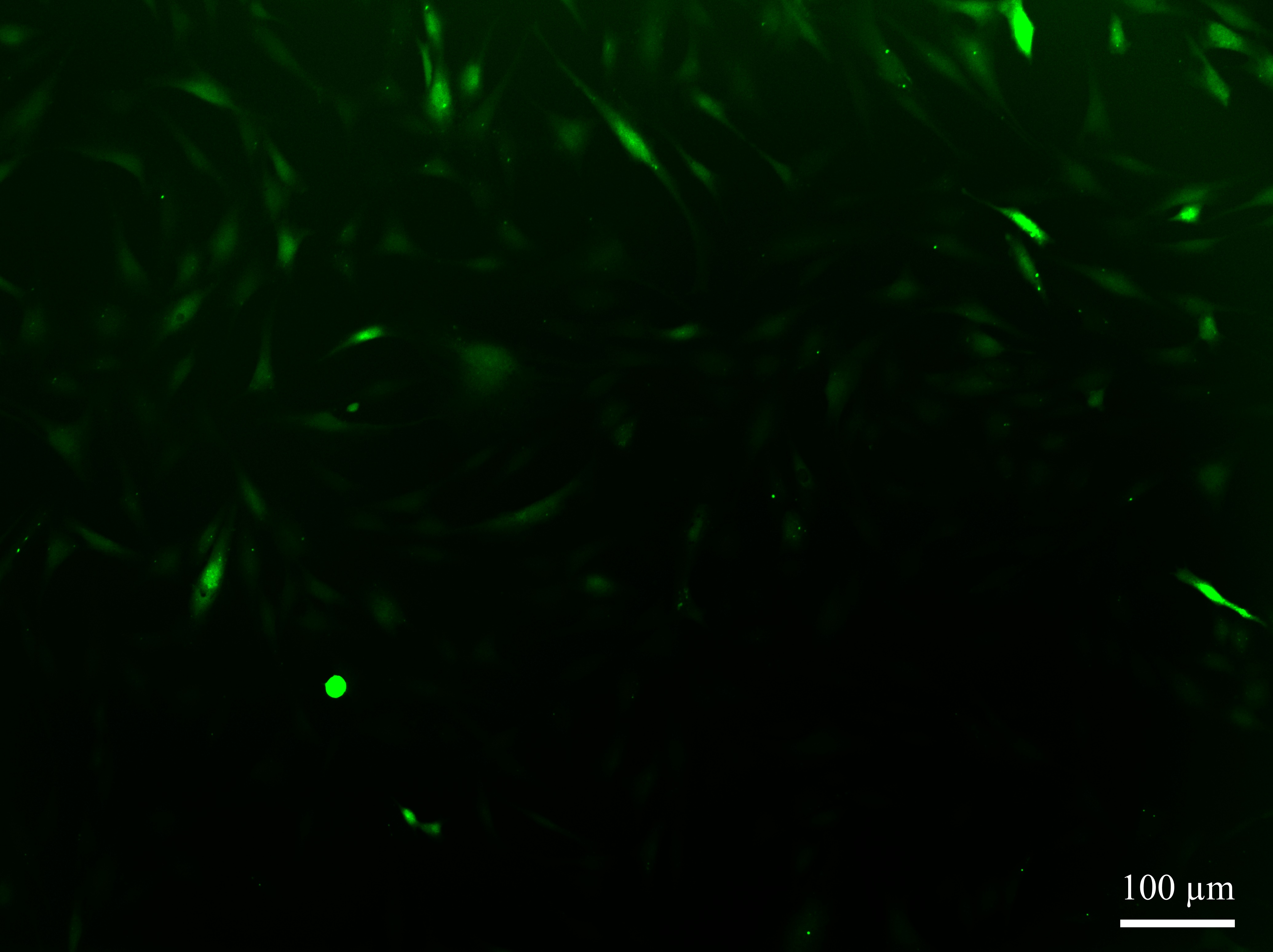

Supplement: S3 File — (ZIP) [file pone.0324264.s003.zip › supplement.material-3/ROS/96-Control3.jpg]

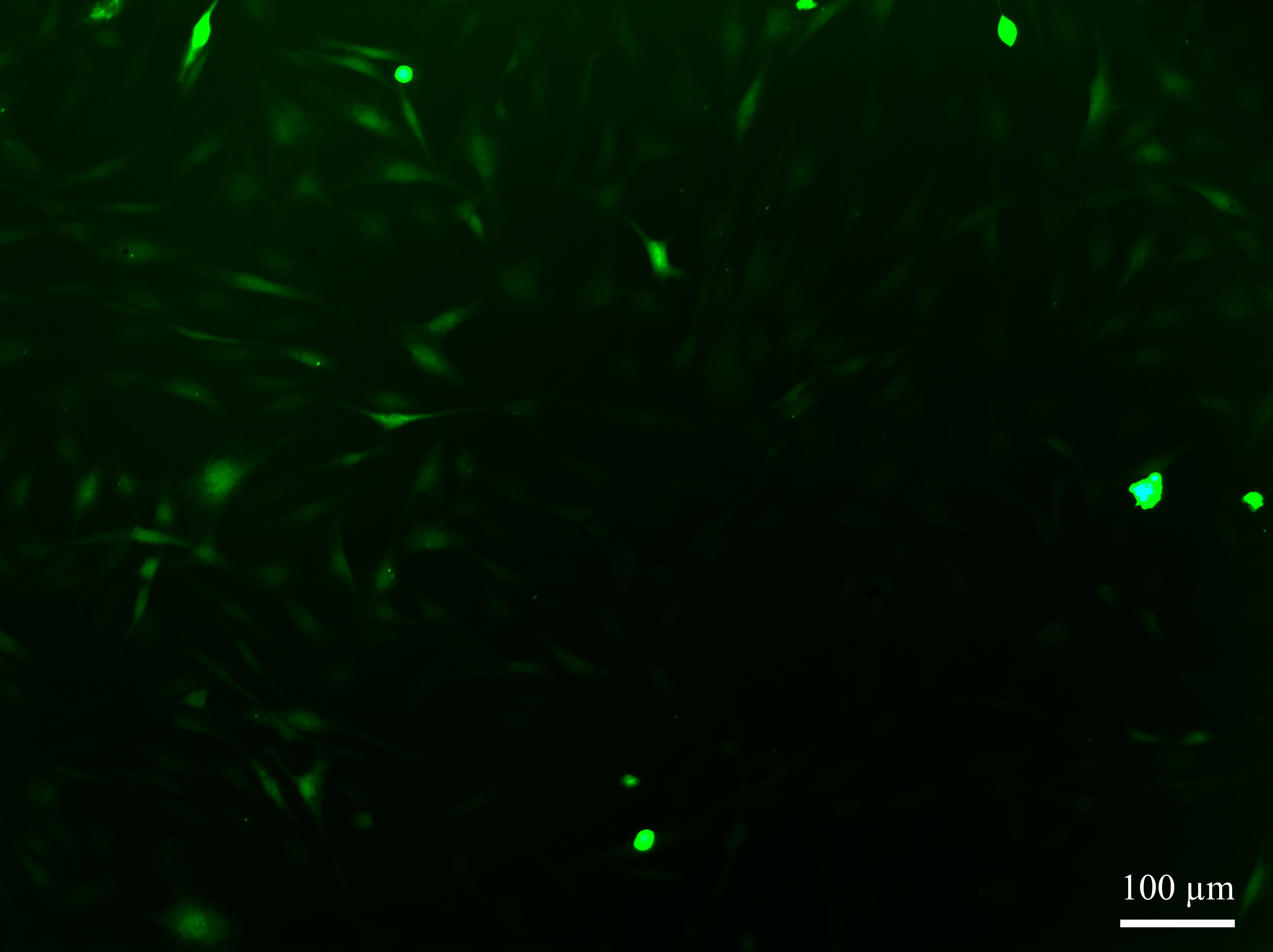

Supplement: S3 File — (ZIP) [file pone.0324264.s003.zip › supplement.material-3/ROS/96-Control4.jpg]

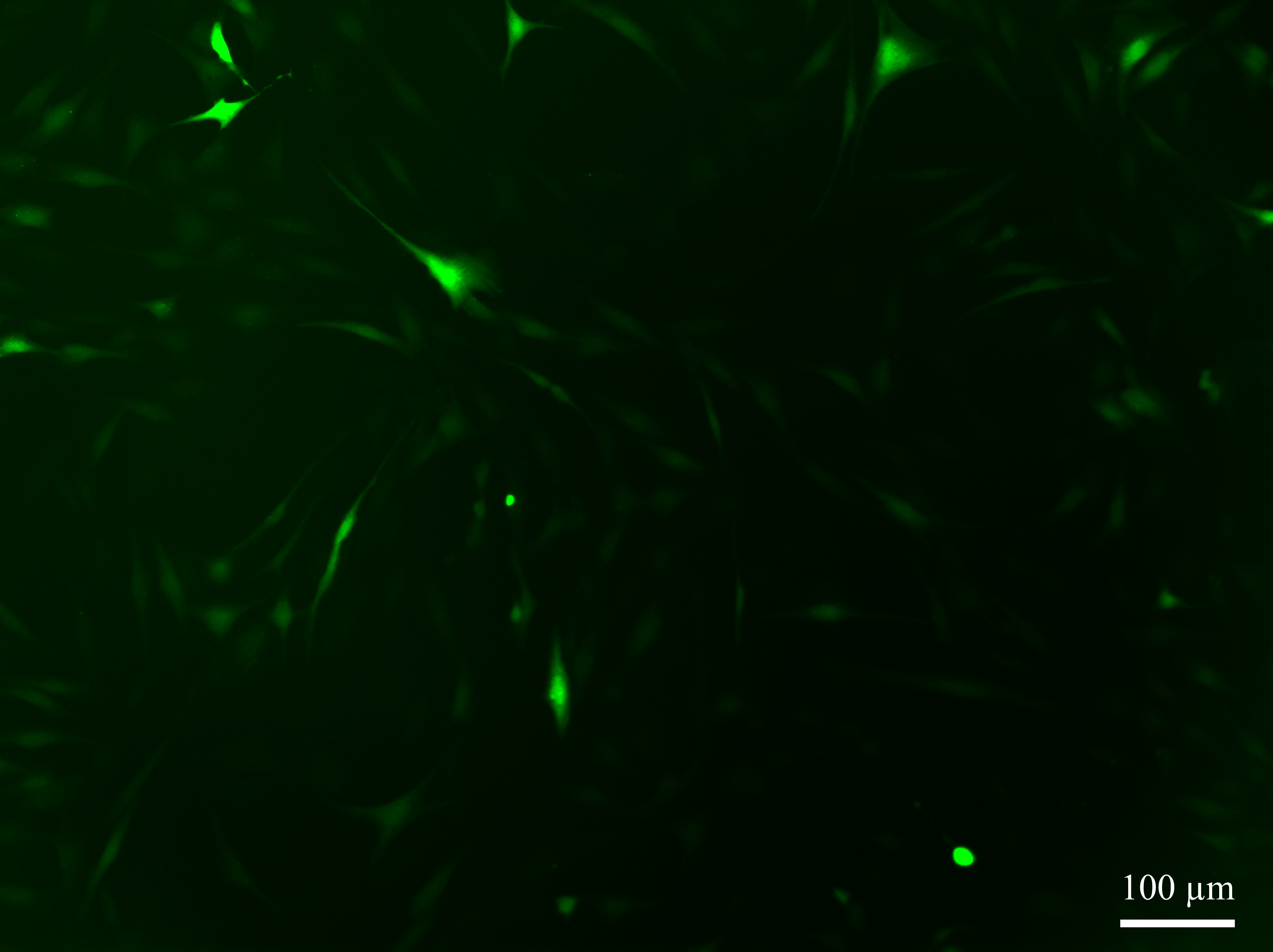

Supplement: S3 File — (ZIP) [file pone.0324264.s003.zip › supplement.material-3/ROS/96-Control5.jpg]

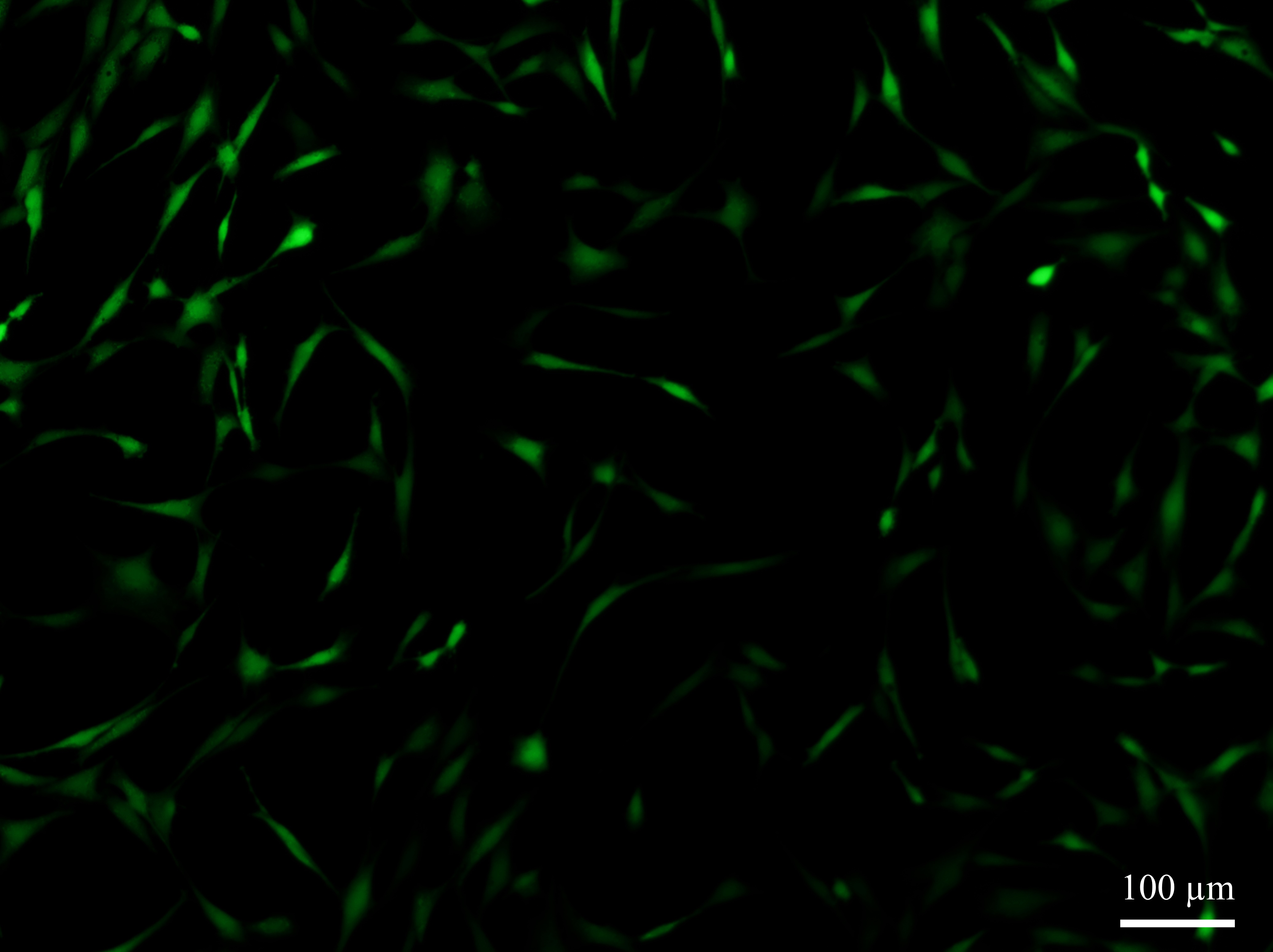

Supplement: S3 File — (ZIP) [file pone.0324264.s003.zip › supplement.material-3/ROS/96-Model1.jpg]

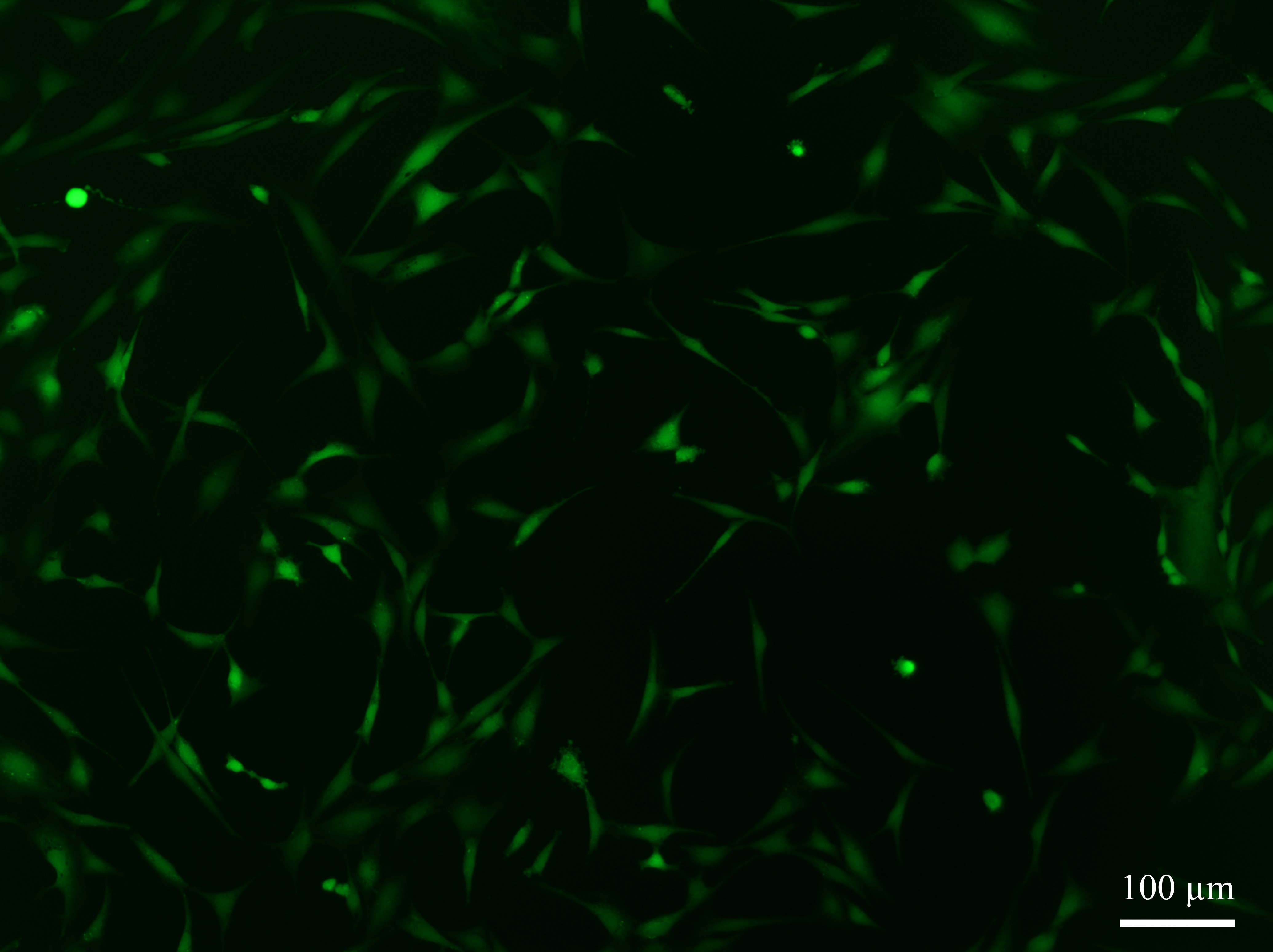

Supplement: S3 File — (ZIP) [file pone.0324264.s003.zip › supplement.material-3/ROS/96-Model2.jpg]

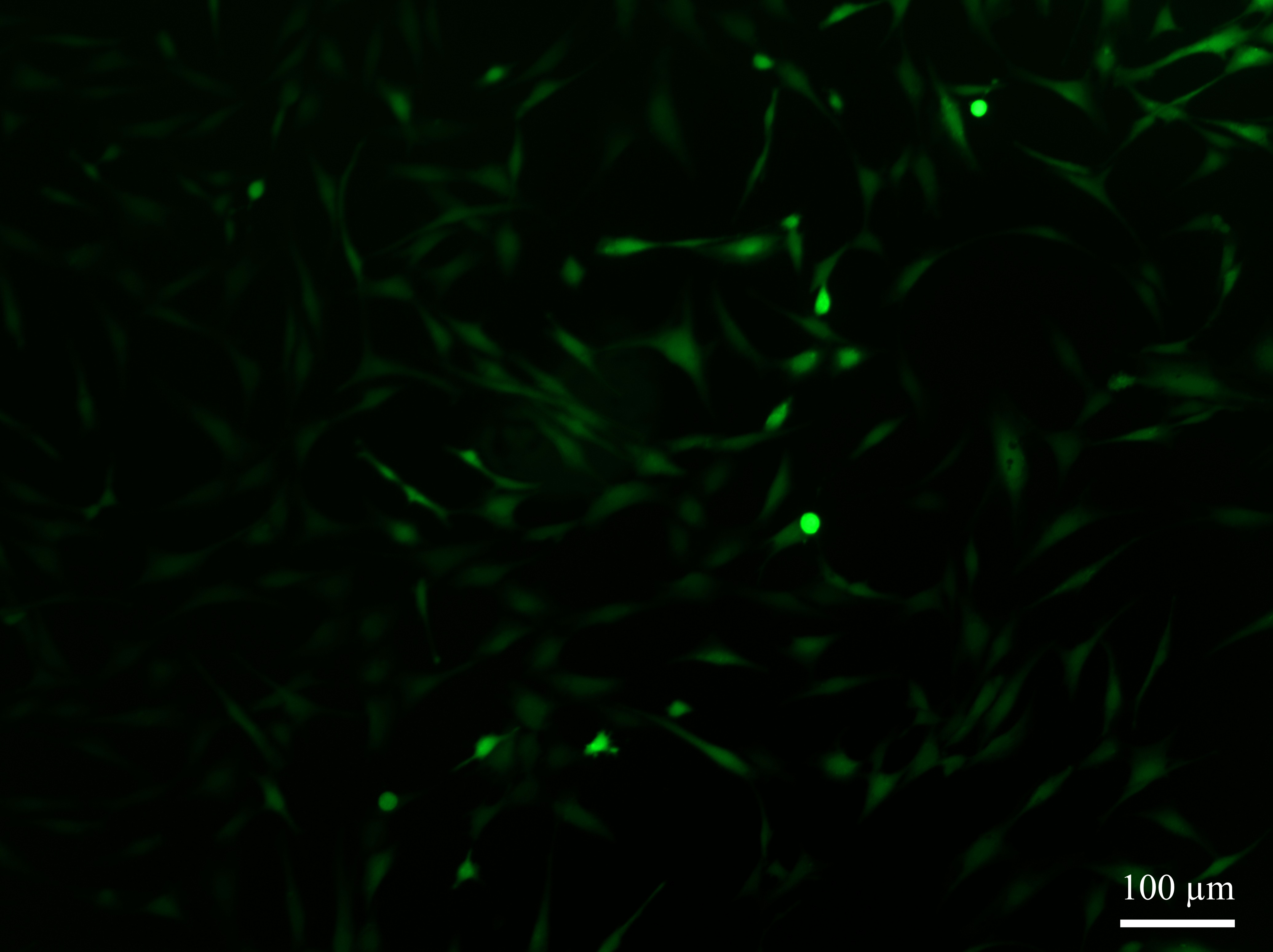

Supplement: S3 File — (ZIP) [file pone.0324264.s003.zip › supplement.material-3/ROS/96-Model3.jpg]

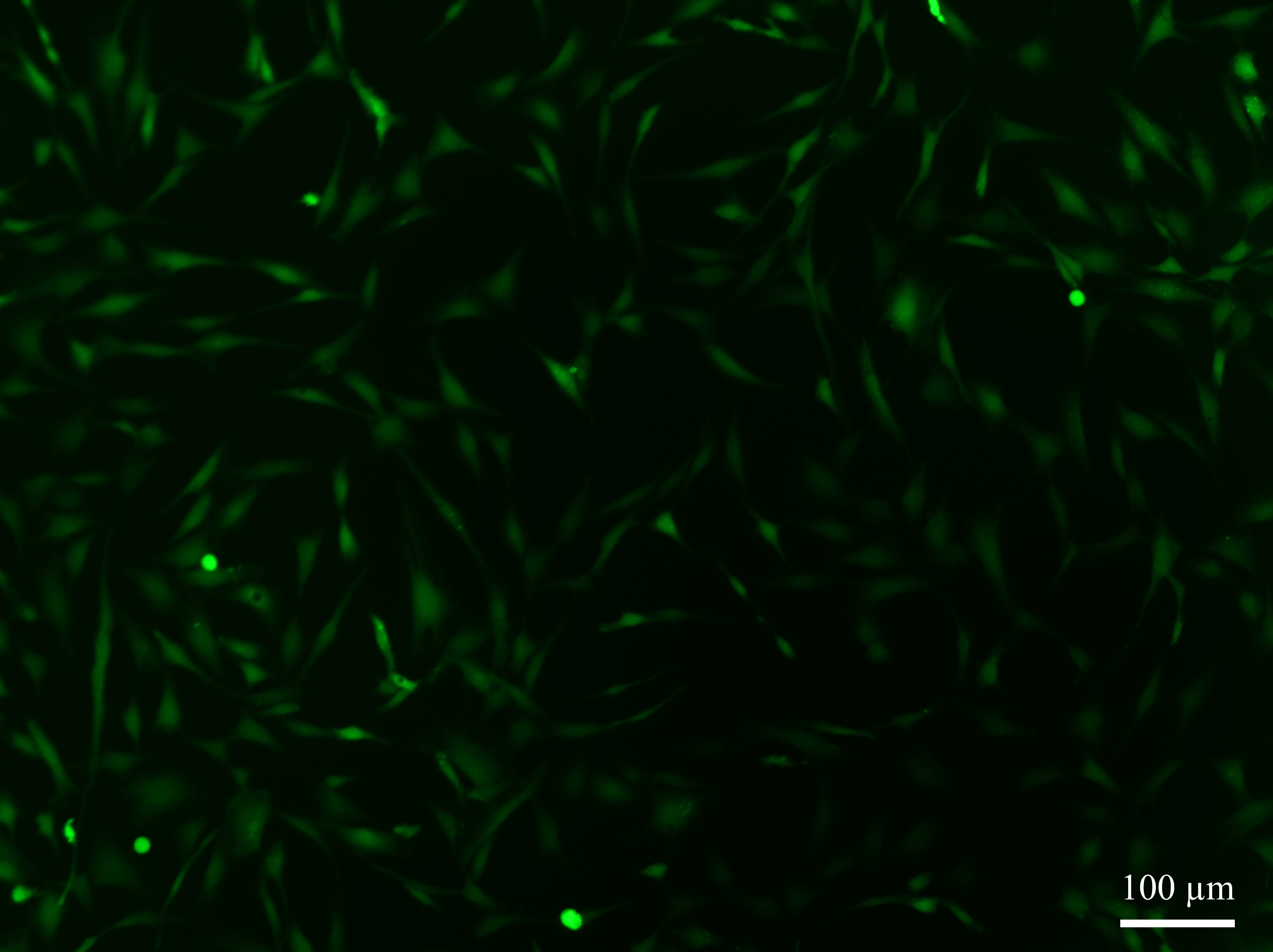

Supplement: S3 File — (ZIP) [file pone.0324264.s003.zip › supplement.material-3/ROS/96-Model4.jpg]

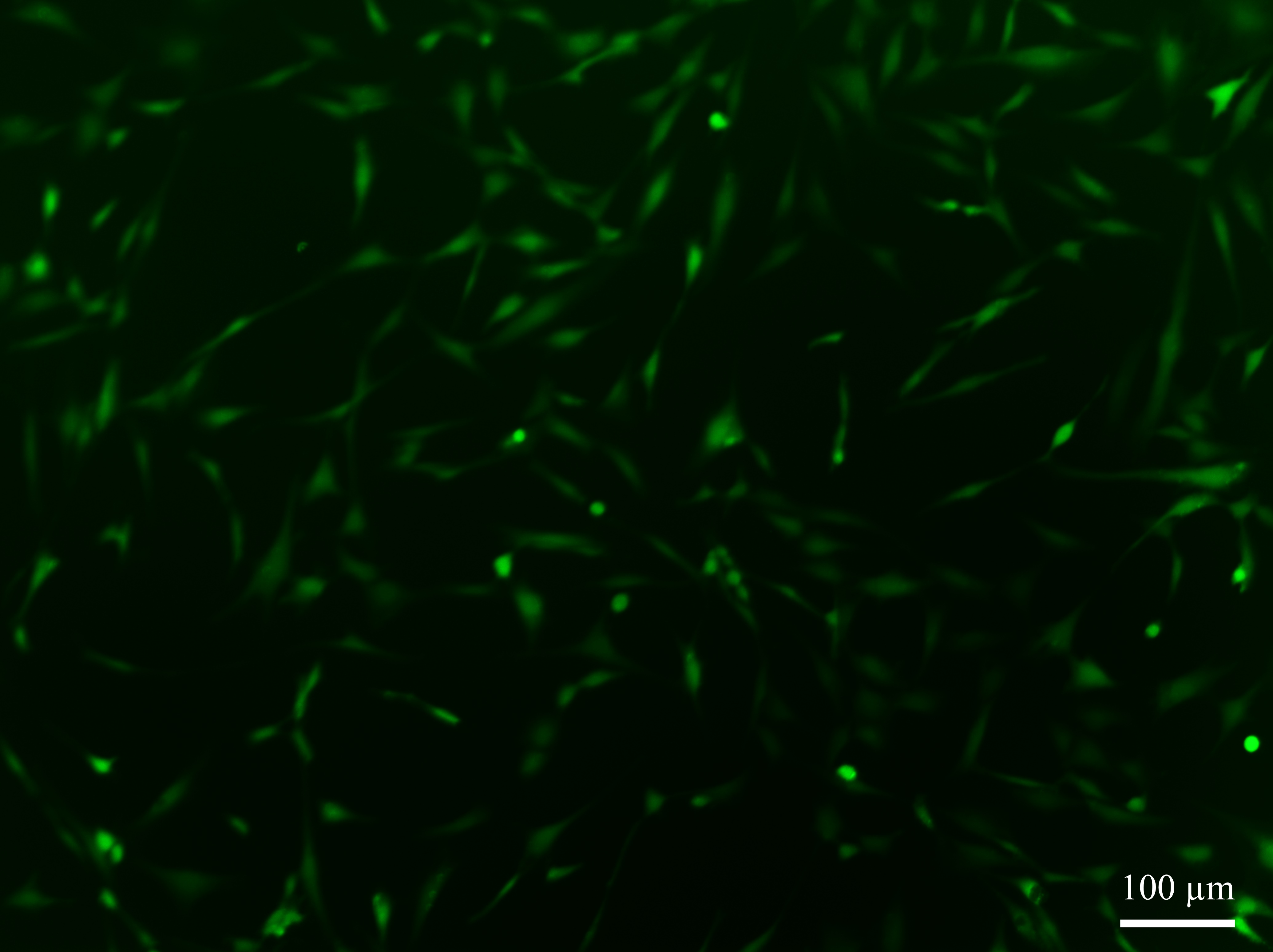

Supplement: S3 File — (ZIP) [file pone.0324264.s003.zip › supplement.material-3/ROS/96-Model5.jpg]

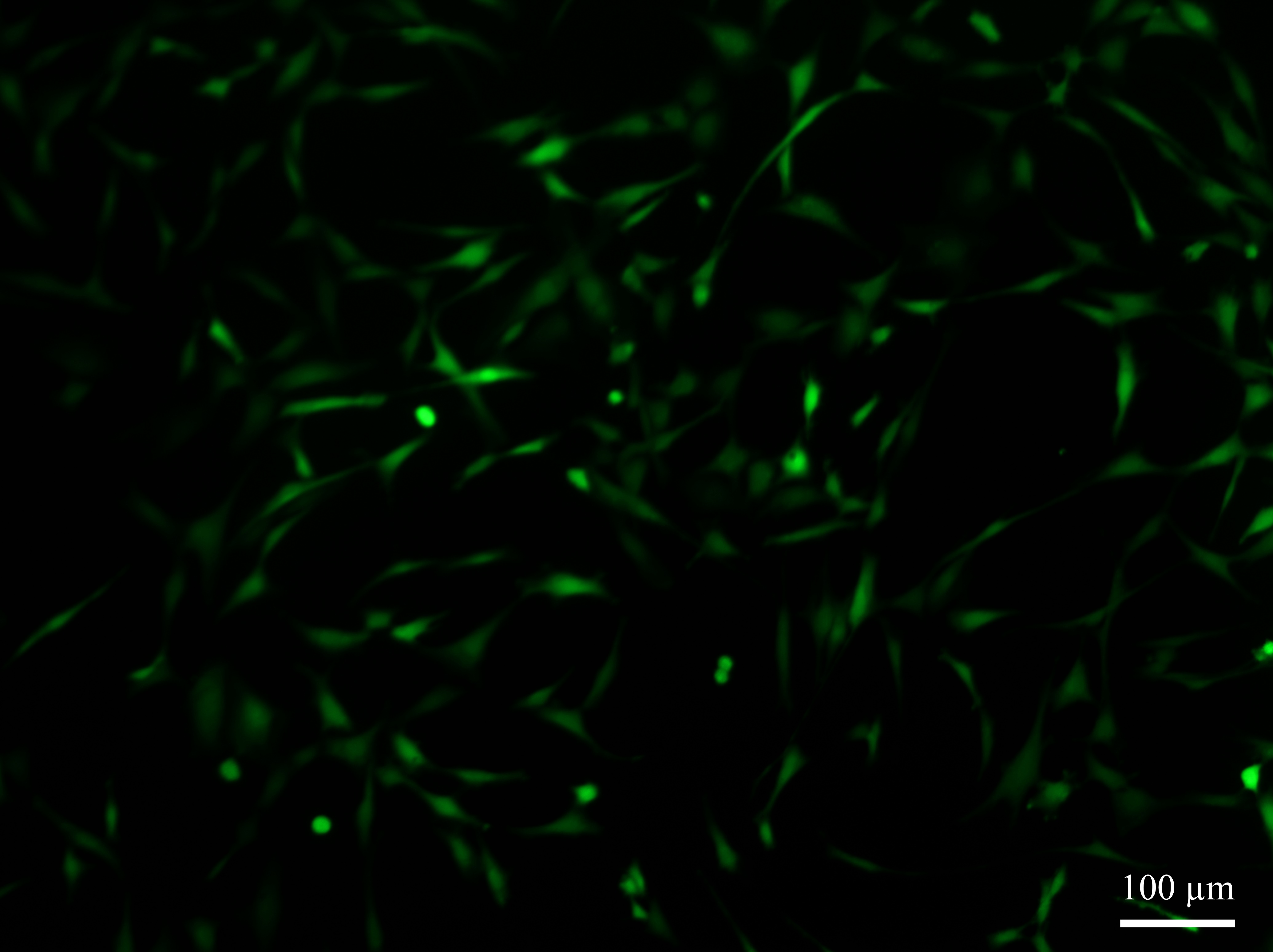

Supplement: S3 File — (ZIP) [file pone.0324264.s003.zip › supplement.material-3/ROS/96-PL1.jpg]

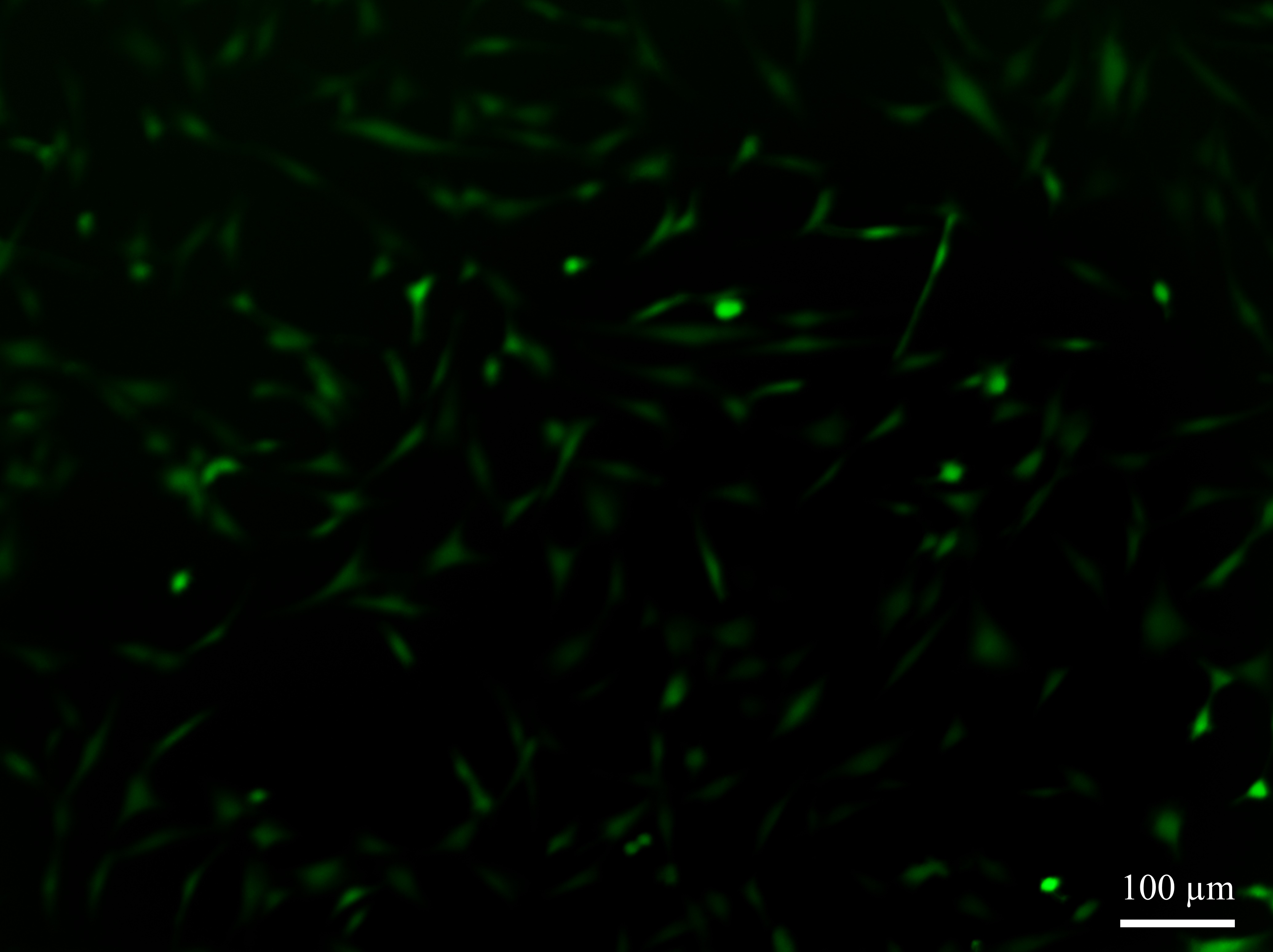

Supplement: S3 File — (ZIP) [file pone.0324264.s003.zip › supplement.material-3/ROS/96-PL2.jpg]

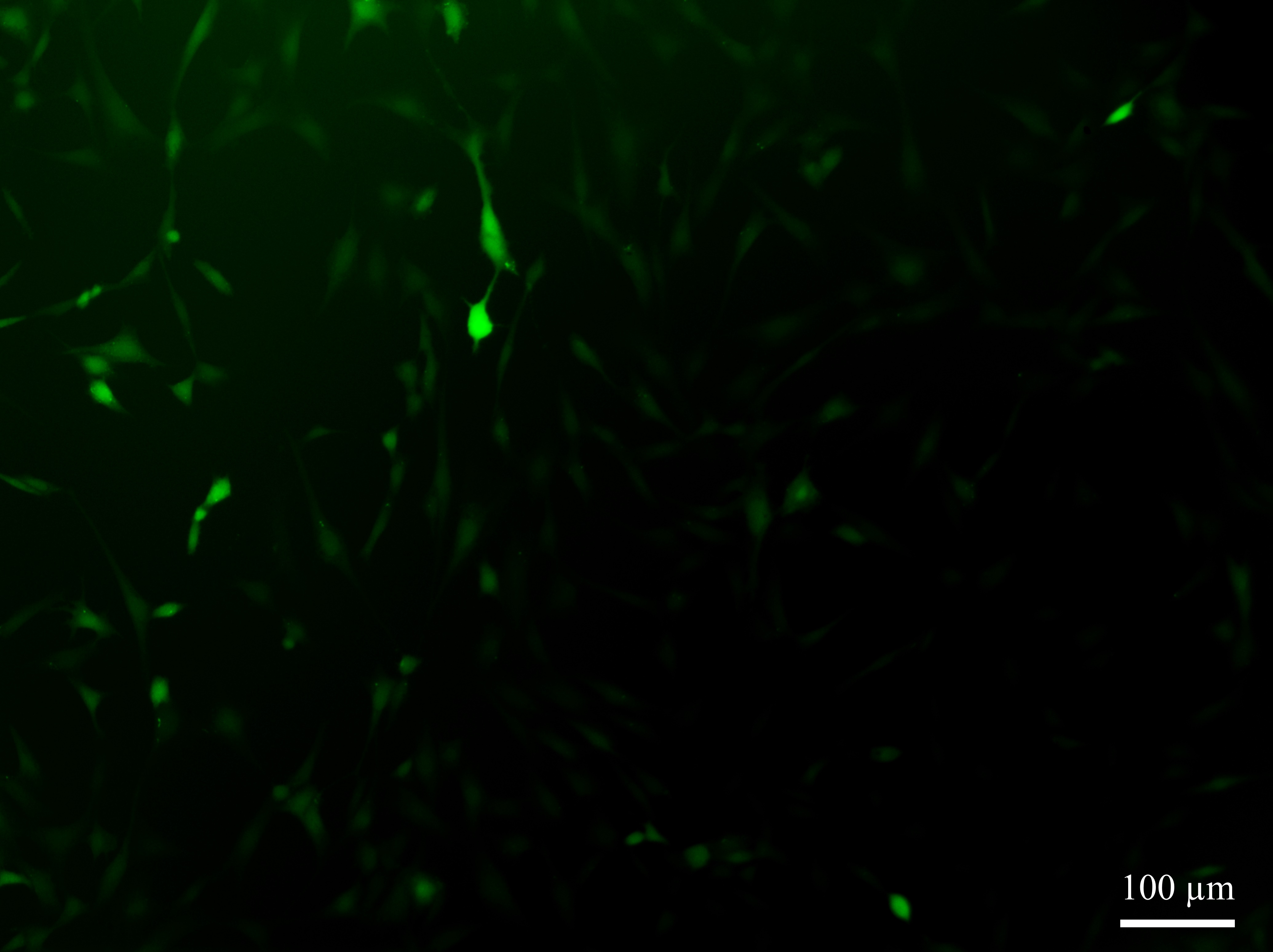

Supplement: S3 File — (ZIP) [file pone.0324264.s003.zip › supplement.material-3/ROS/96-PL3.jpg]

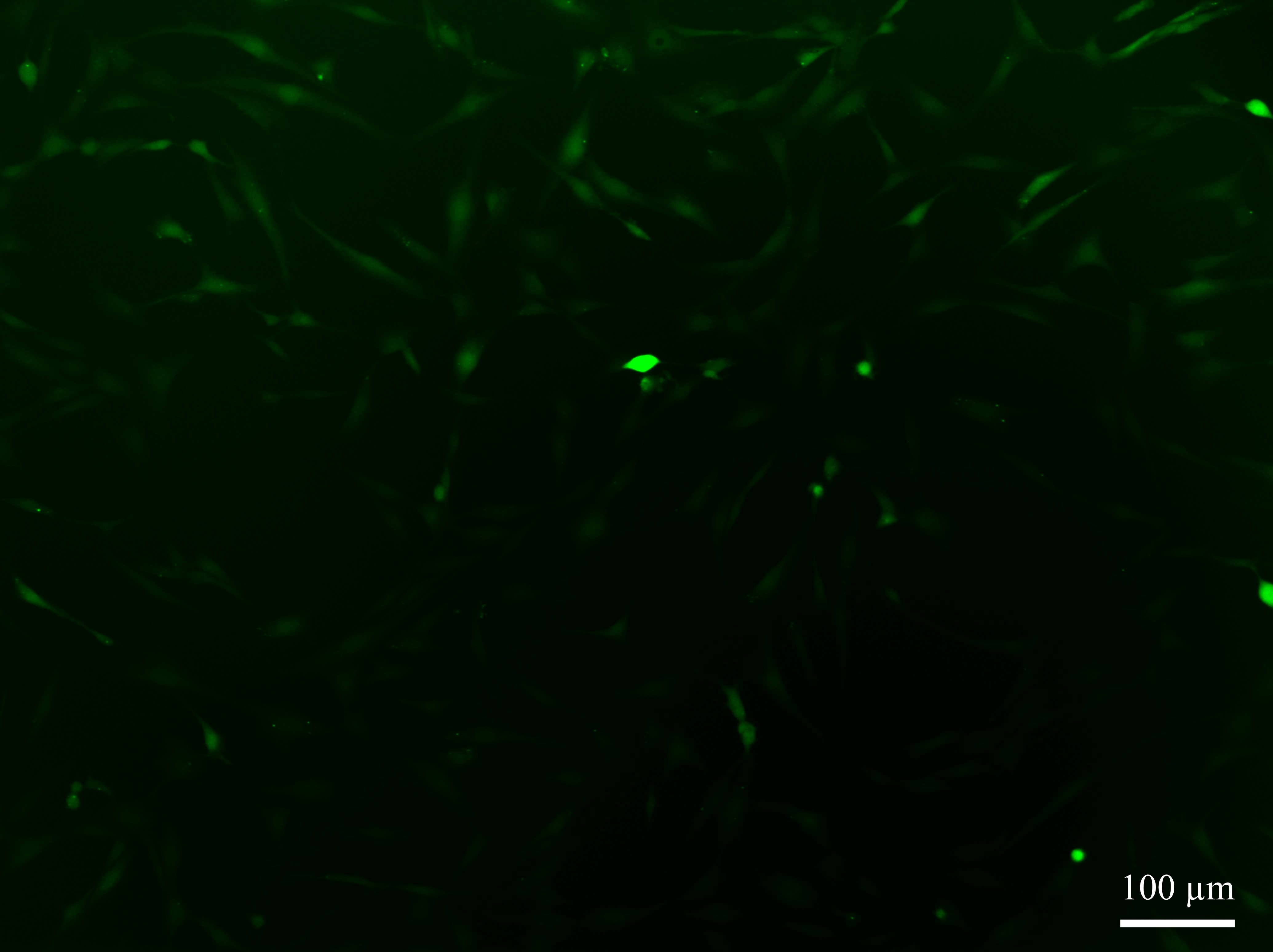

Supplement: S3 File — (ZIP) [file pone.0324264.s003.zip › supplement.material-3/ROS/96-PL4.jpg]

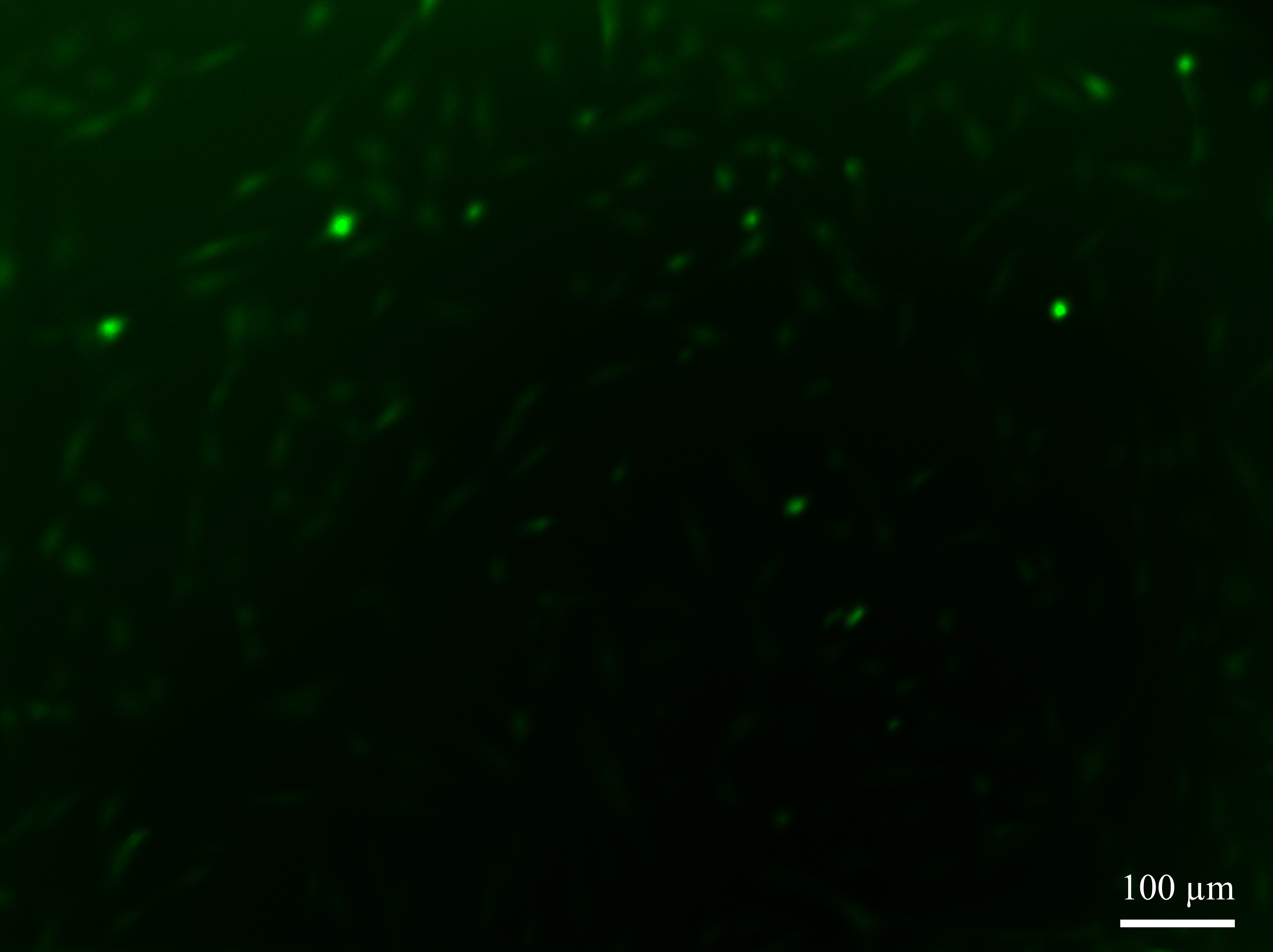

Supplement: S3 File — (ZIP) [file pone.0324264.s003.zip › supplement.material-3/ROS/96-PL5.jpg]

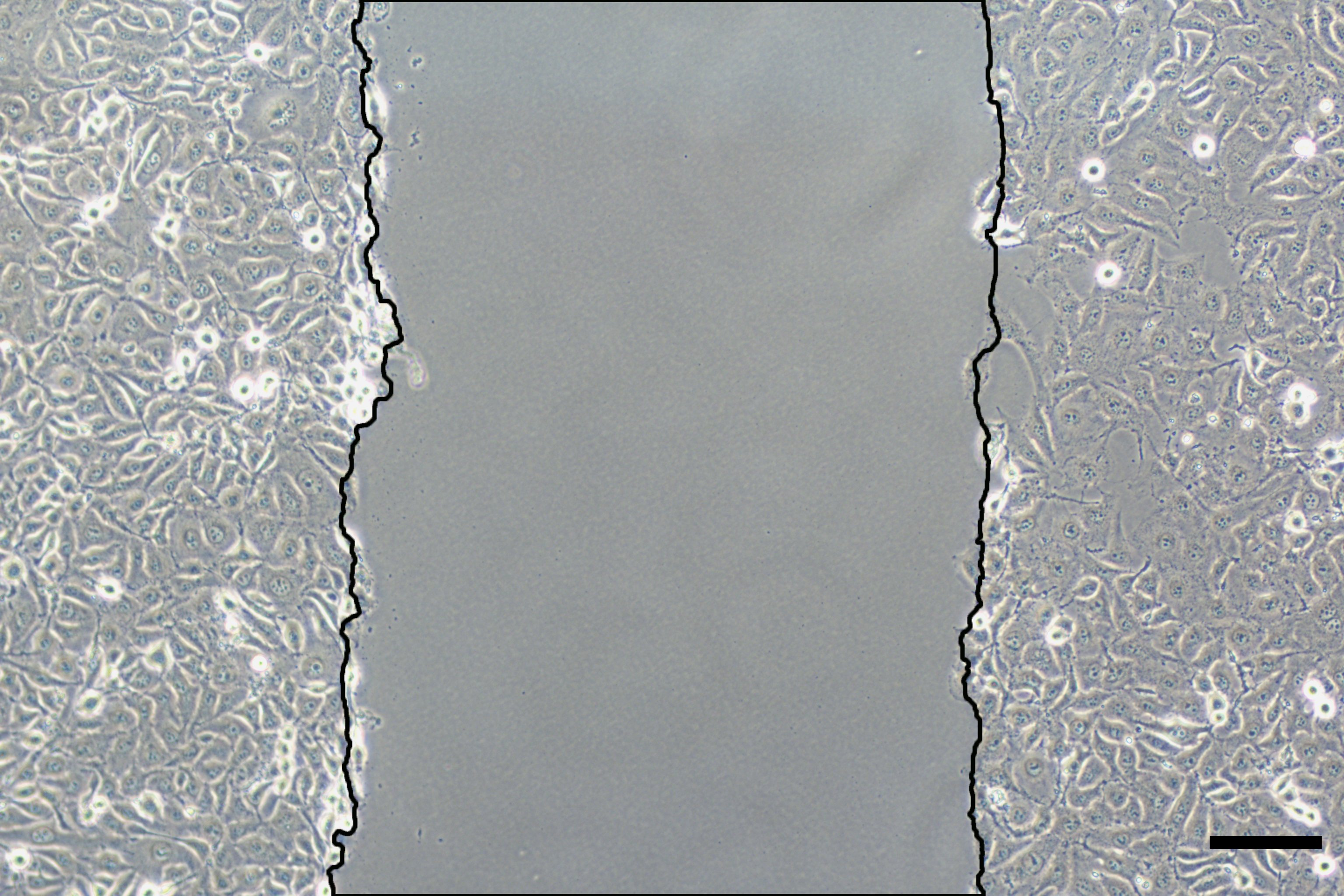

Supplement: S4 File — (ZIP) [file pone.0324264.s004.zip › supplement.material-4/images(Cell Scratch Assay)- HUVEC-0H/0-Model1-.jpg]

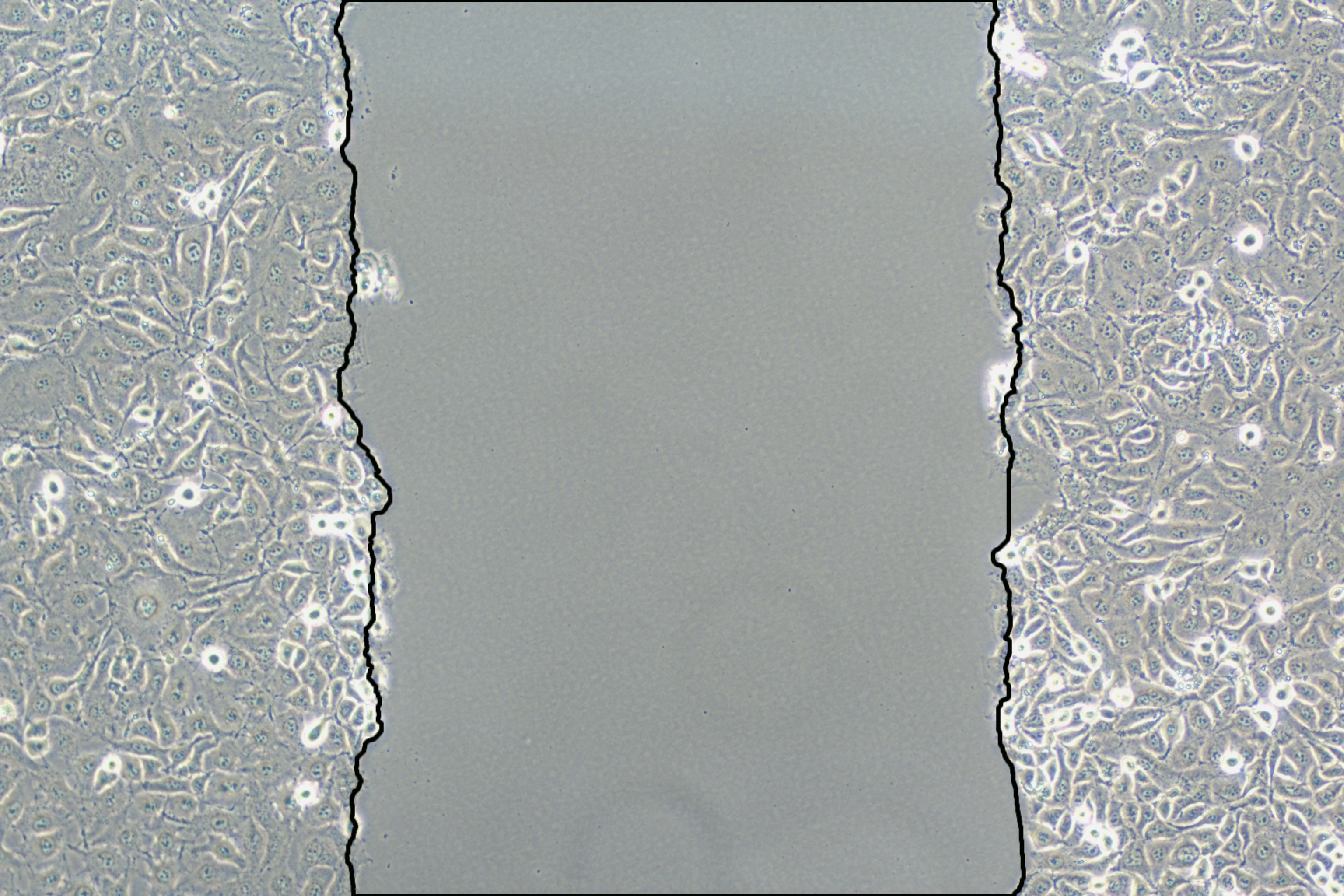

Supplement: S4 File — (ZIP) [file pone.0324264.s004.zip › supplement.material-4/images(Cell Scratch Assay)- HUVEC-0H/0-Model2.jpg]

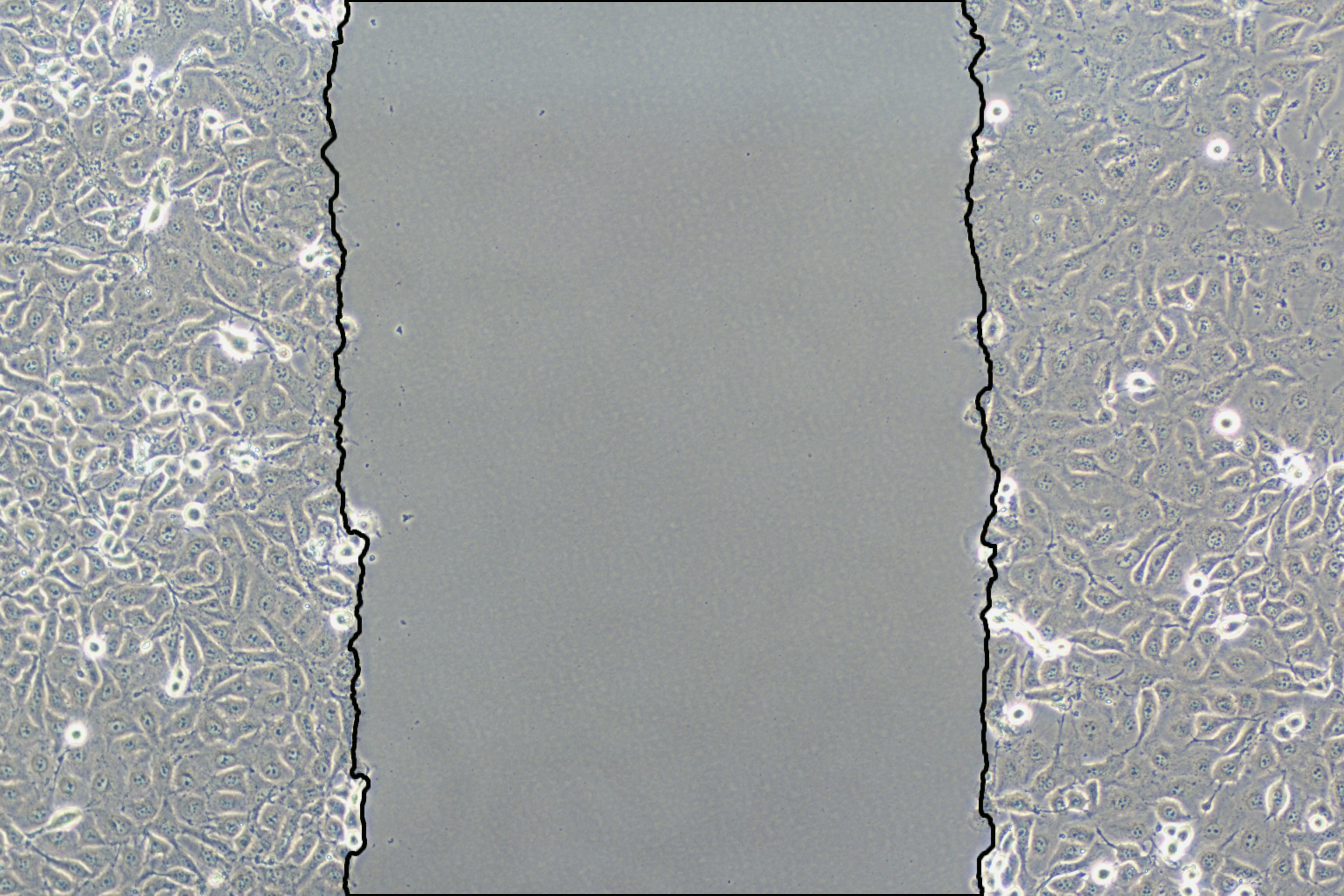

Supplement: S4 File — (ZIP) [file pone.0324264.s004.zip › supplement.material-4/images(Cell Scratch Assay)- HUVEC-0H/0-Model3.jpg]

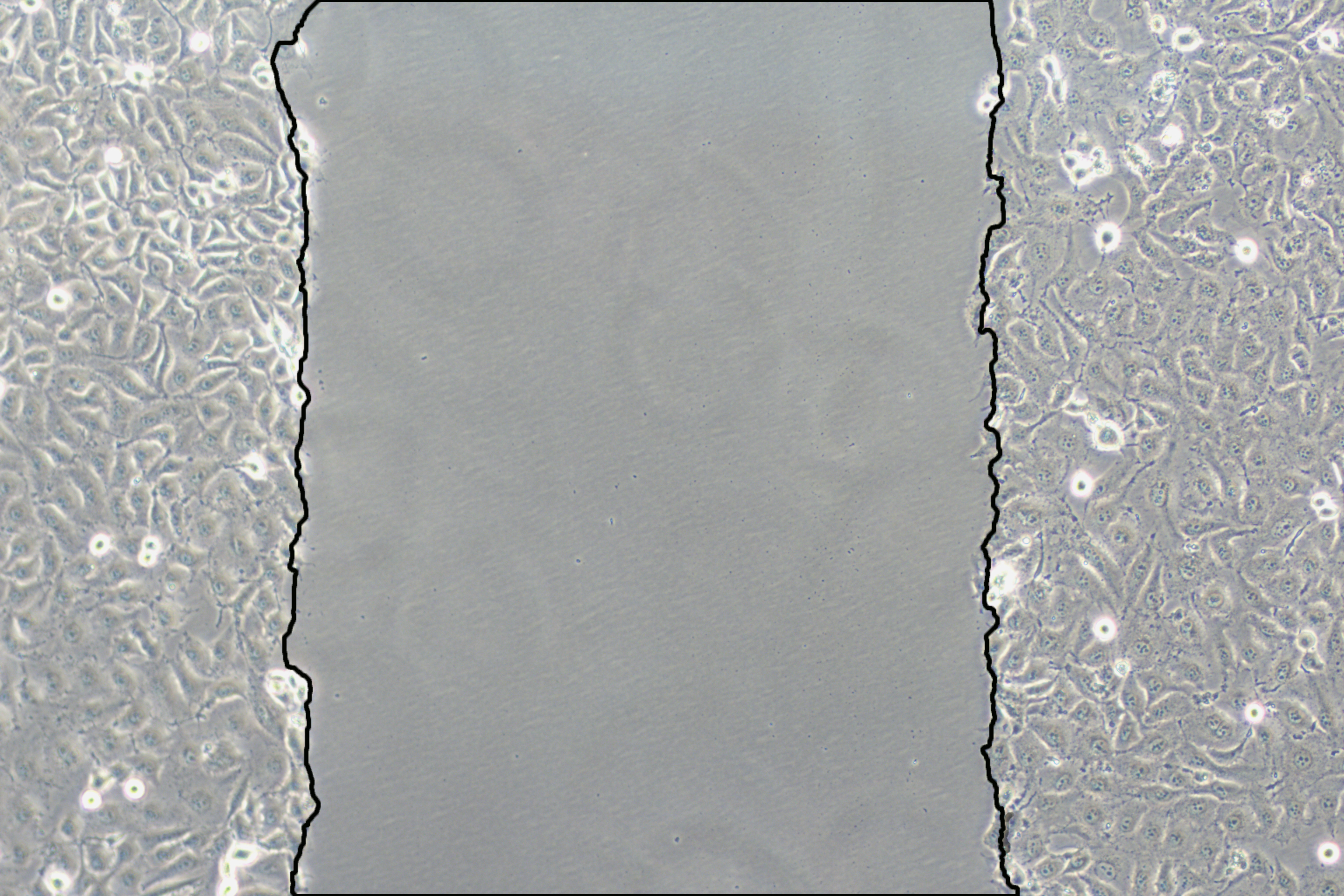

Supplement: S4 File — (ZIP) [file pone.0324264.s004.zip › supplement.material-4/images(Cell Scratch Assay)- HUVEC-0H/0-Model4.jpg]

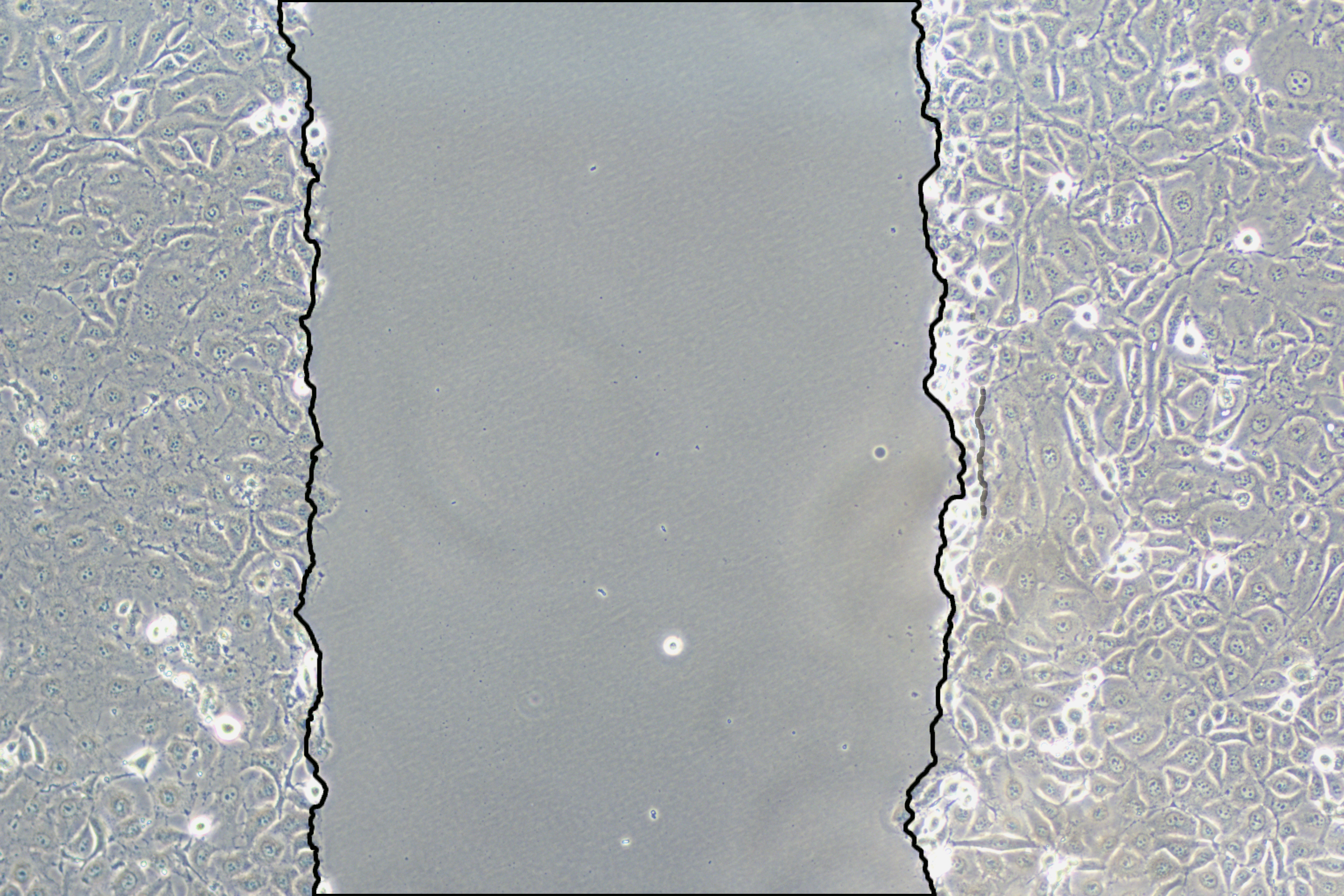

Supplement: S4 File — (ZIP) [file pone.0324264.s004.zip › supplement.material-4/images(Cell Scratch Assay)- HUVEC-0H/0-Model5.jpg]

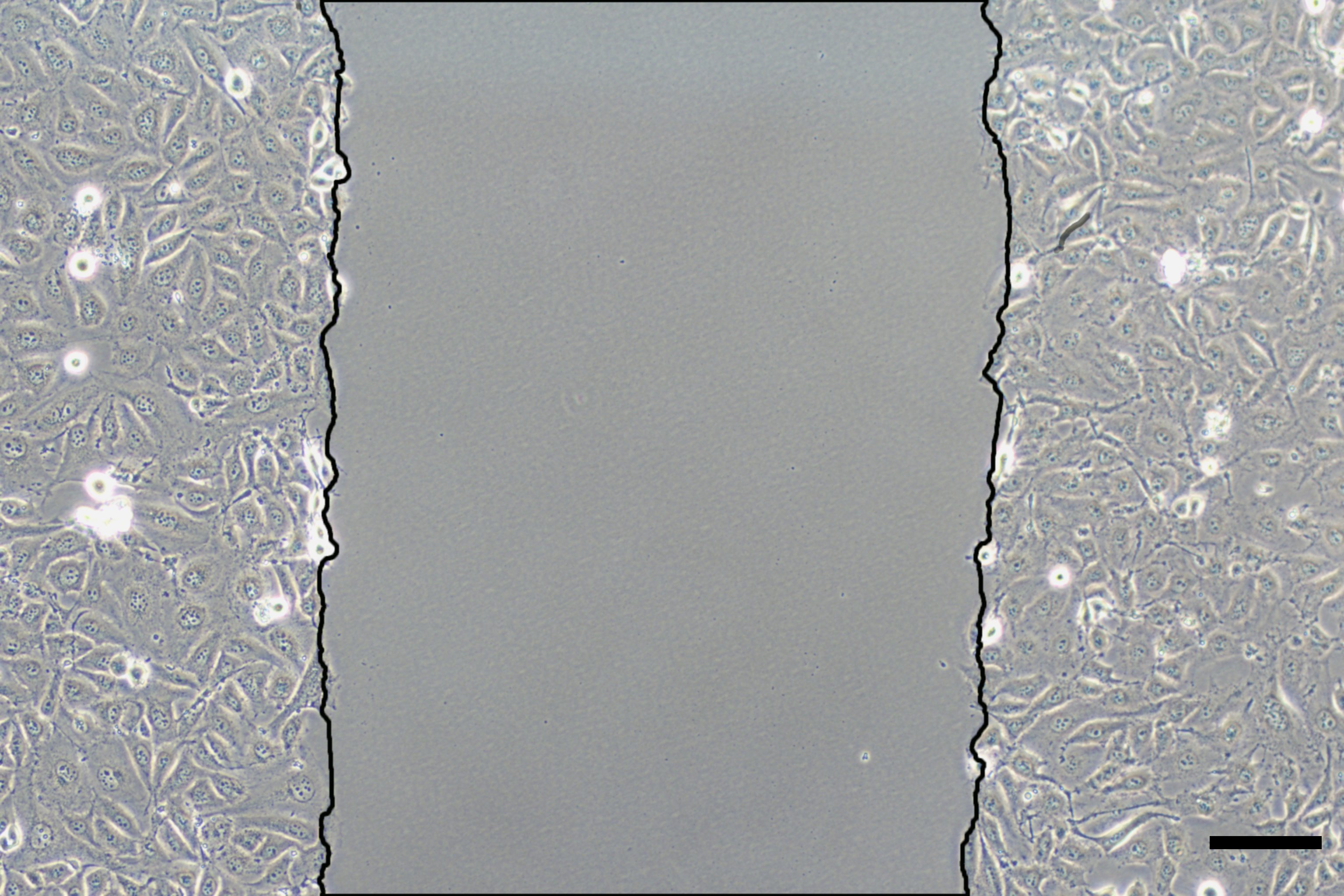

Supplement: S4 File — (ZIP) [file pone.0324264.s004.zip › supplement.material-4/images(Cell Scratch Assay)- HUVEC-0H/0-PL10X1-.jpg]

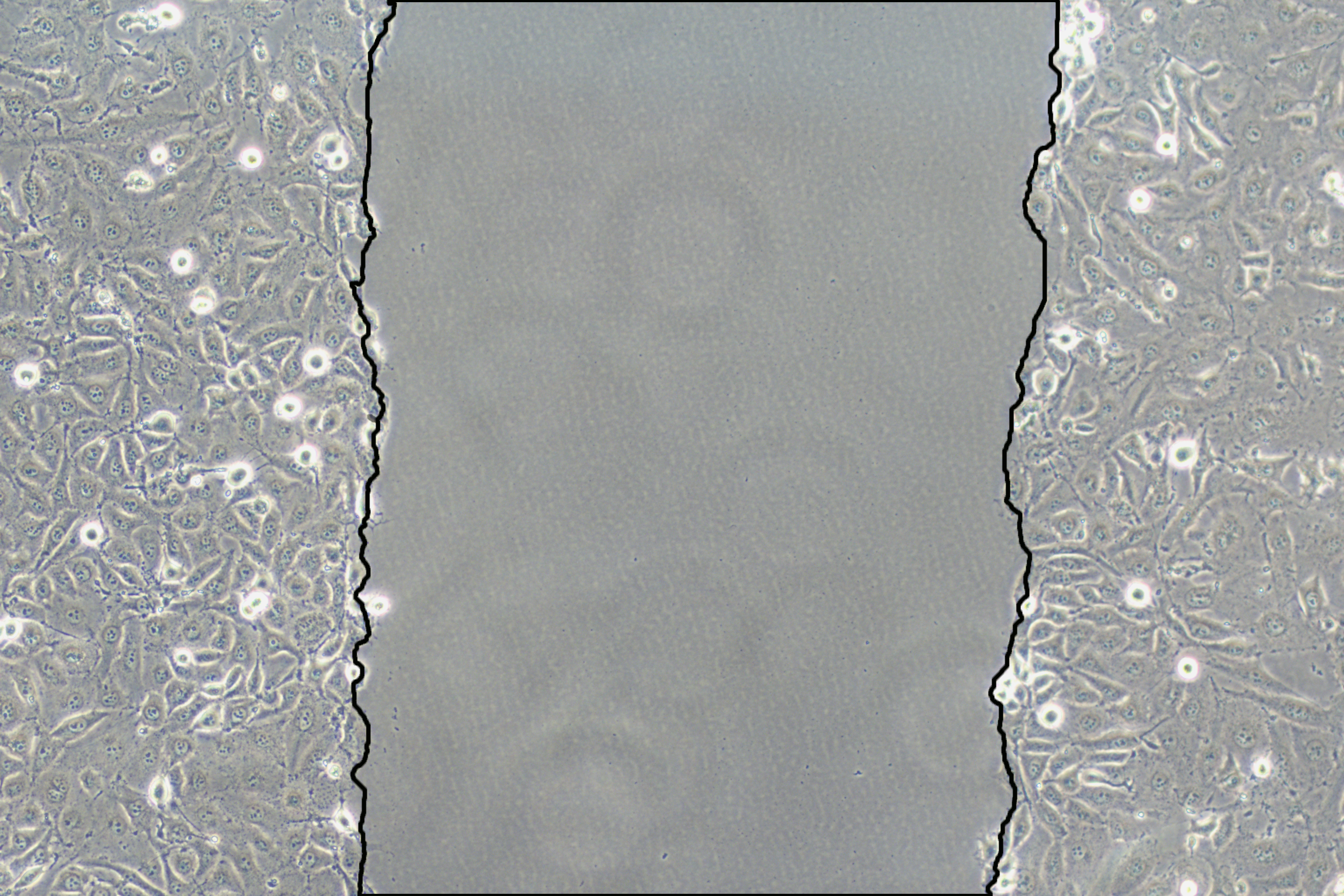

Supplement: S4 File — (ZIP) [file pone.0324264.s004.zip › supplement.material-4/images(Cell Scratch Assay)- HUVEC-0H/0-PL10X2.jpg]

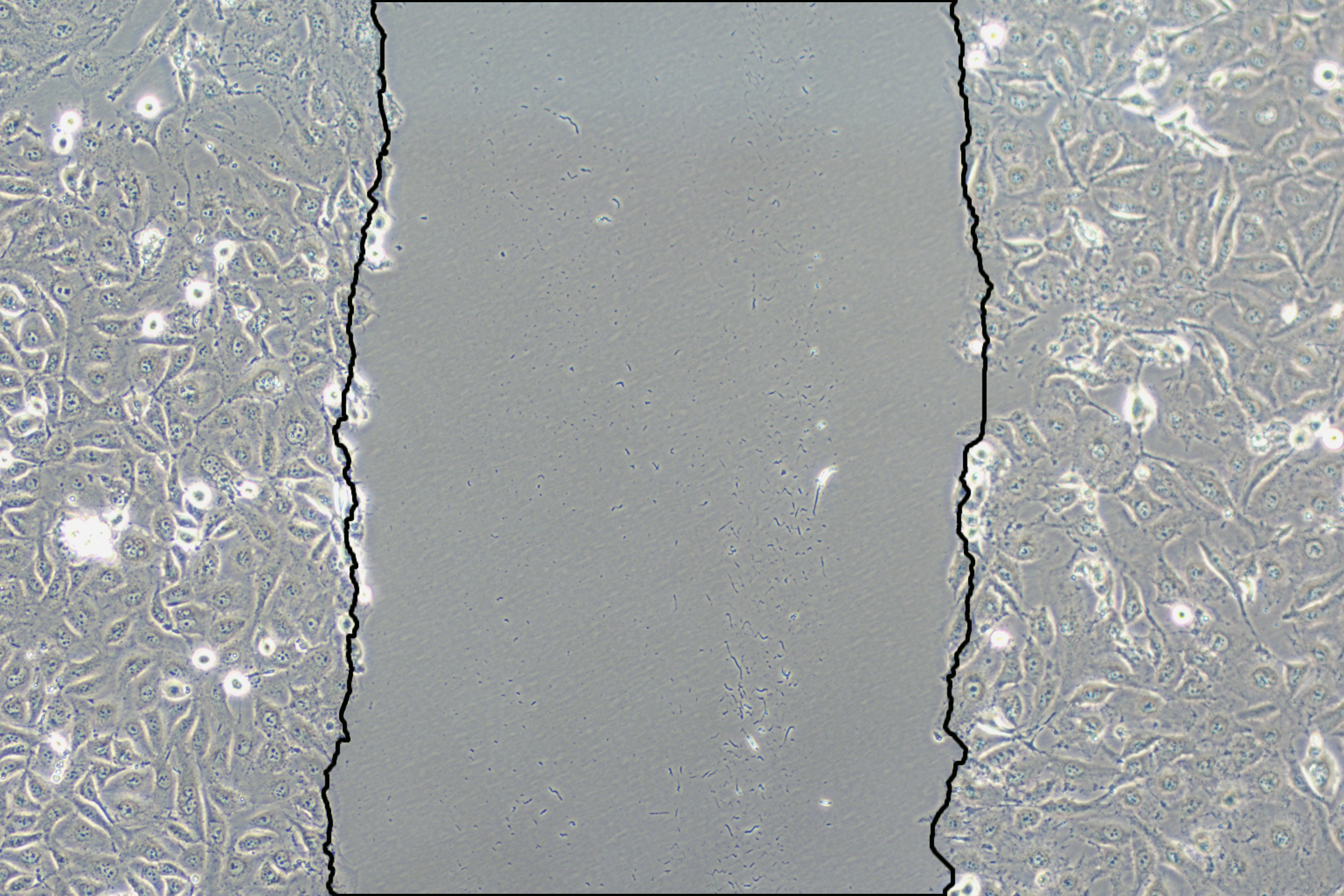

Supplement: S4 File — (ZIP) [file pone.0324264.s004.zip › supplement.material-4/images(Cell Scratch Assay)- HUVEC-0H/0-PL10X3.jpg]

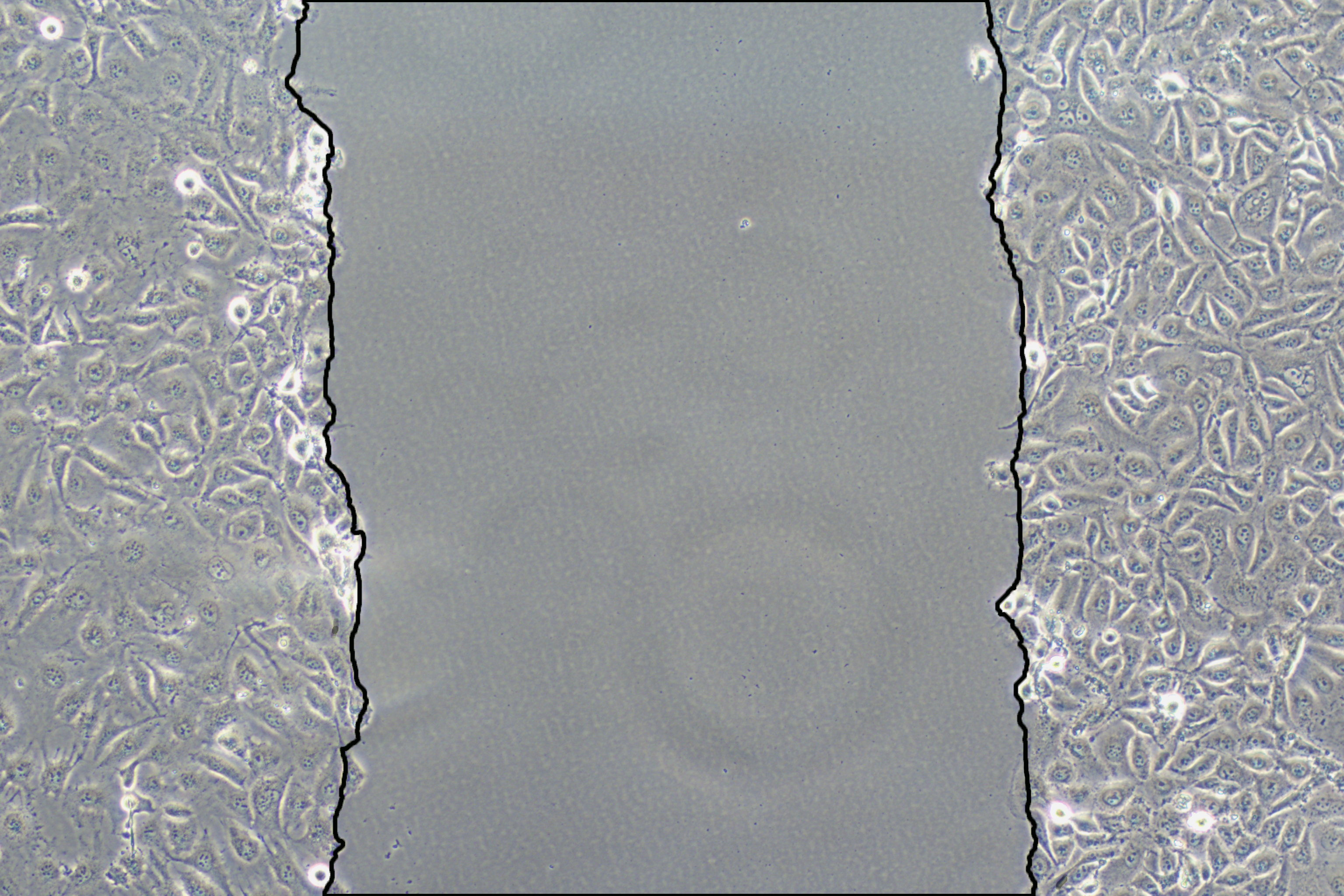

Supplement: S4 File — (ZIP) [file pone.0324264.s004.zip › supplement.material-4/images(Cell Scratch Assay)- HUVEC-0H/0-PL10X4.jpg]

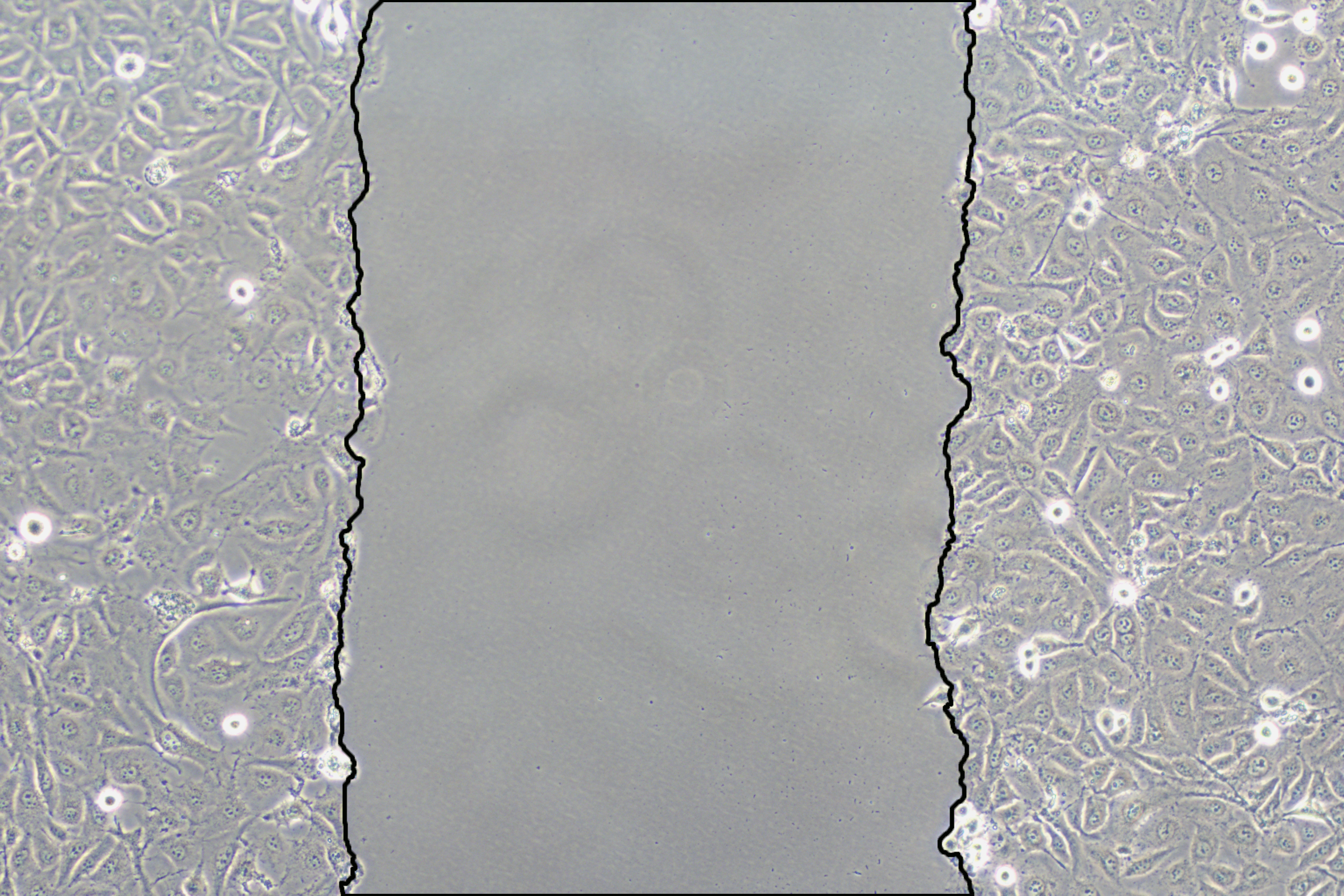

Supplement: S4 File — (ZIP) [file pone.0324264.s004.zip › supplement.material-4/images(Cell Scratch Assay)- HUVEC-0H/0-PL10X5.jpg]

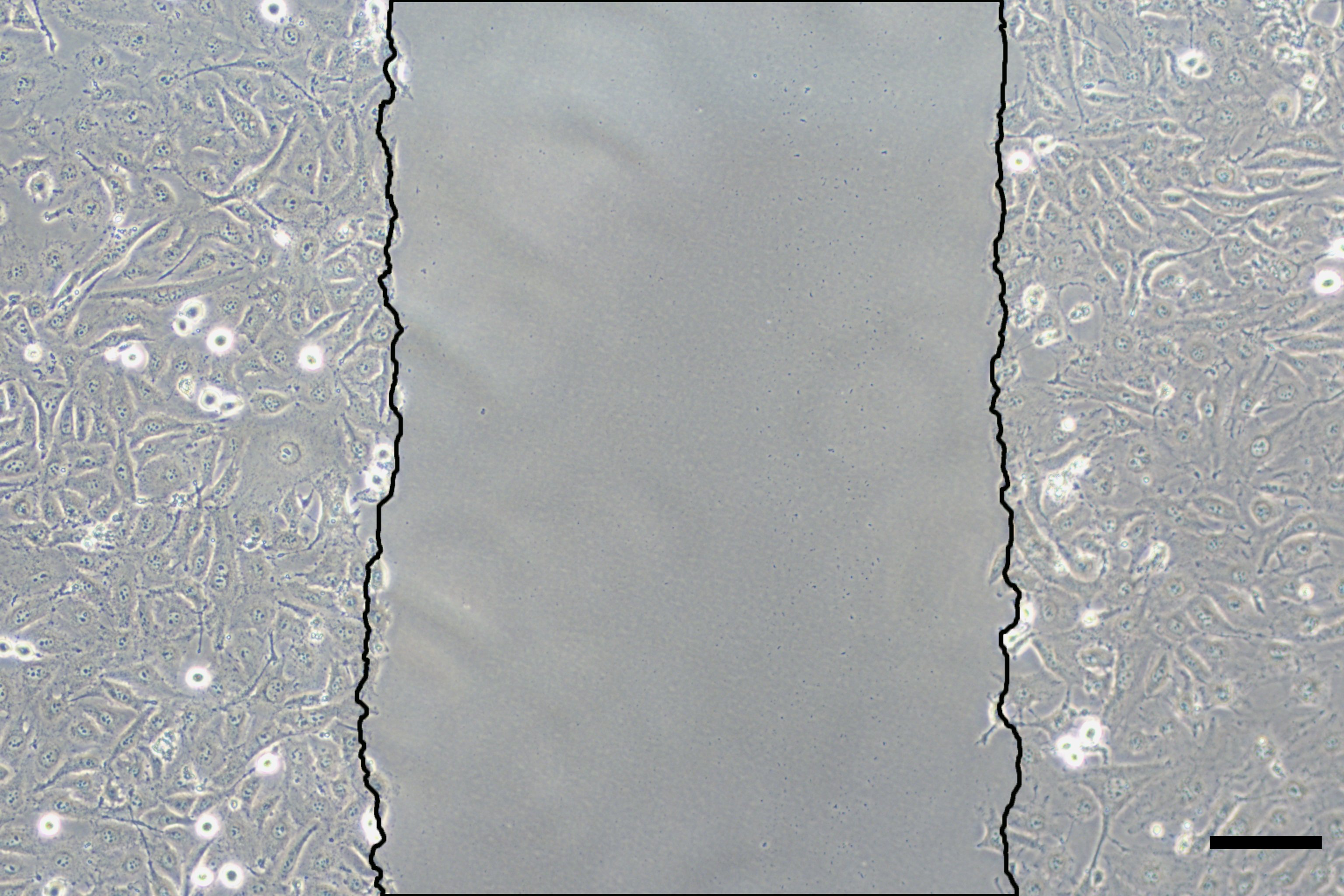

Supplement: S4 File — (ZIP) [file pone.0324264.s004.zip › supplement.material-4/images(Cell Scratch Assay)- HUVEC-0H/0-PL20X1-.jpg]

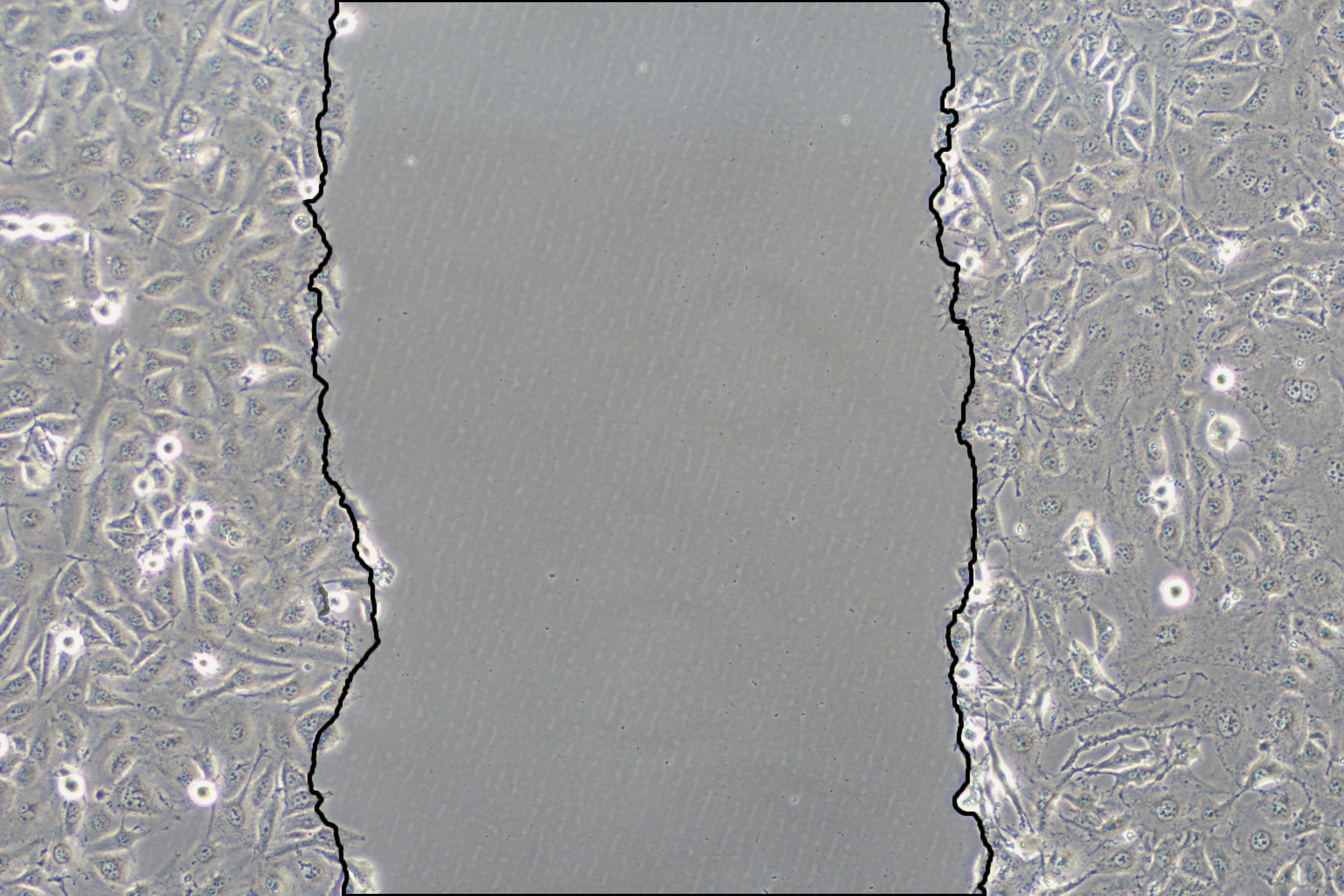

Supplement: S4 File — (ZIP) [file pone.0324264.s004.zip › supplement.material-4/images(Cell Scratch Assay)- HUVEC-0H/0-PL20X2.jpg]

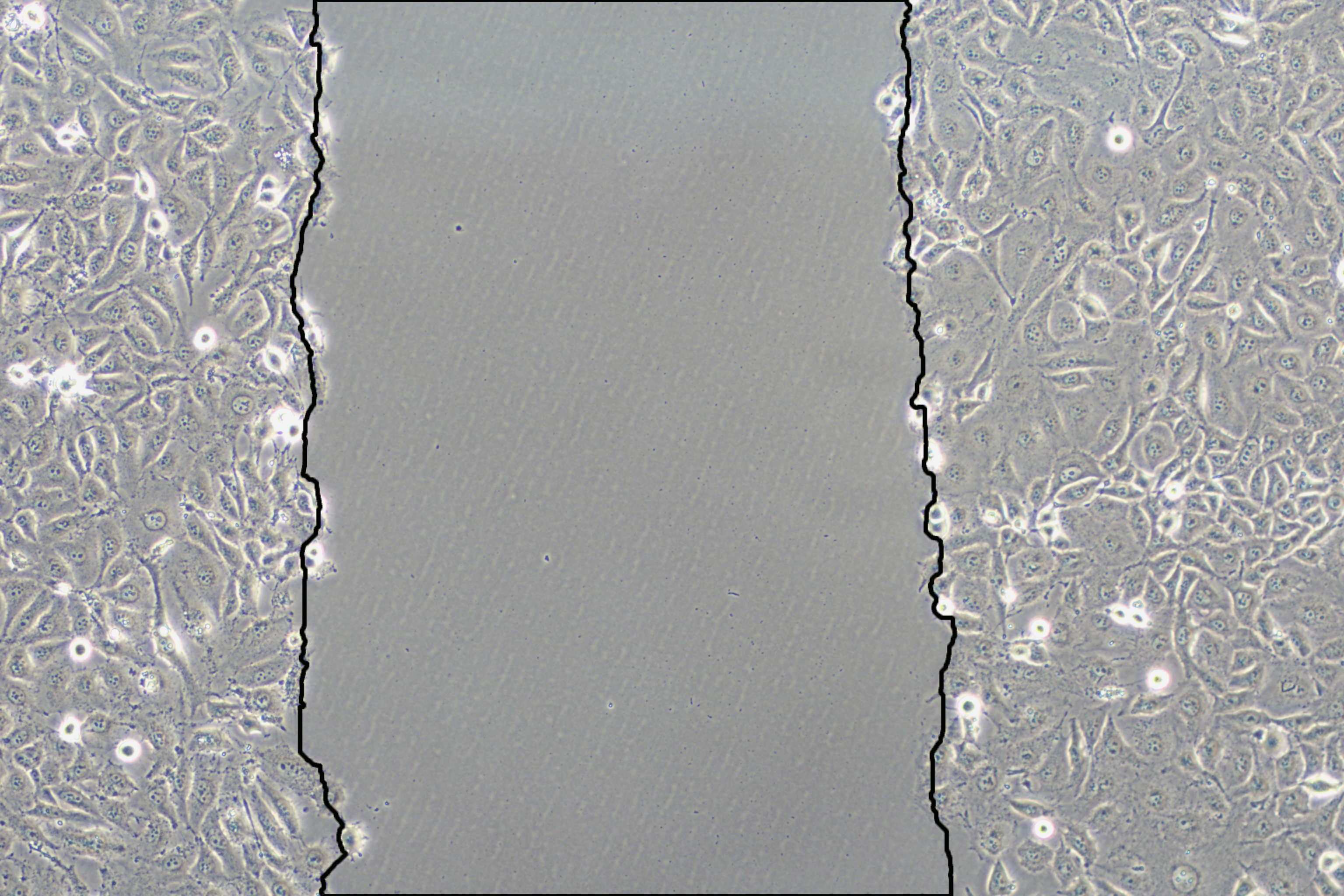

Supplement: S4 File — (ZIP) [file pone.0324264.s004.zip › supplement.material-4/images(Cell Scratch Assay)- HUVEC-0H/0-PL20X3.jpg]

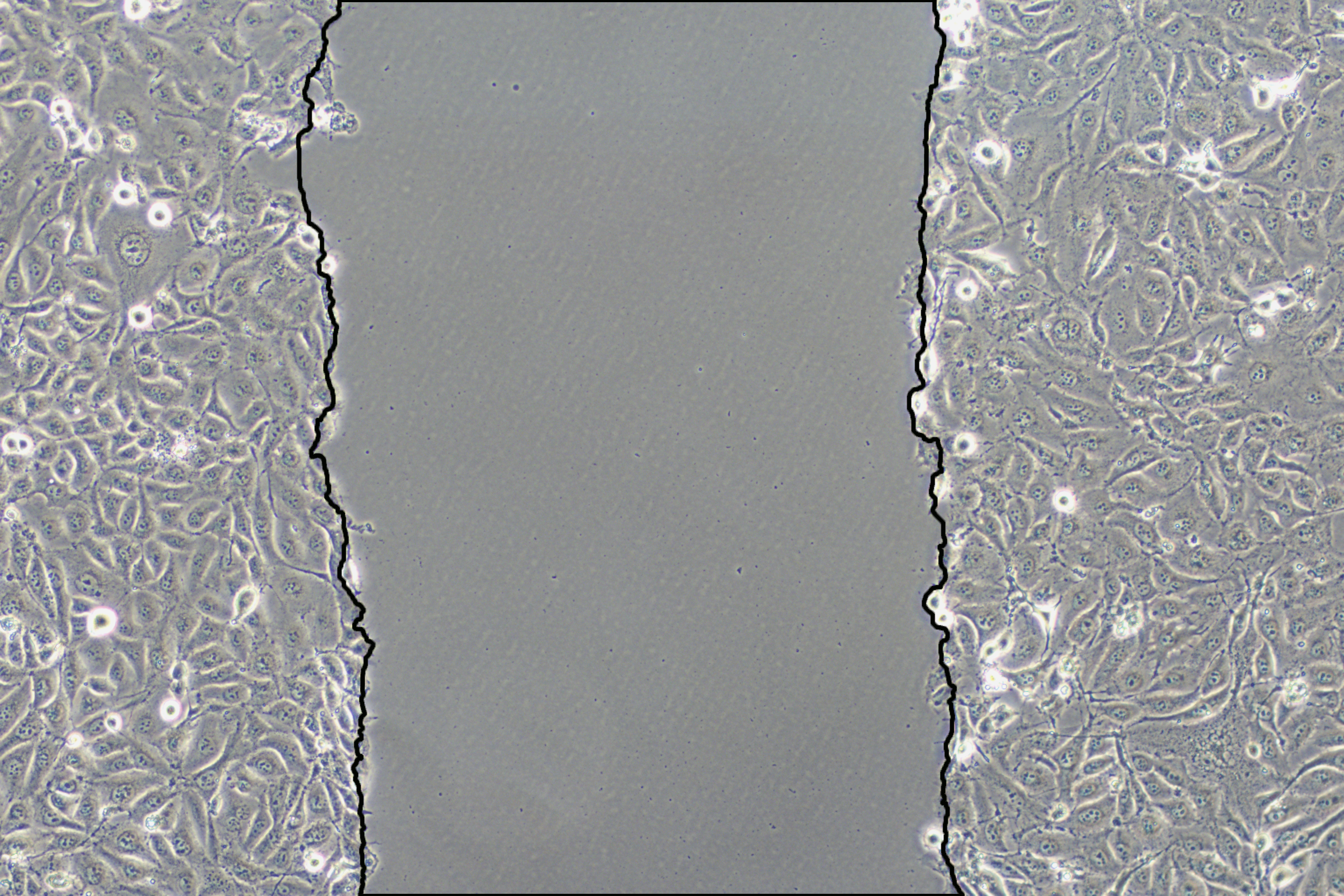

Supplement: S4 File — (ZIP) [file pone.0324264.s004.zip › supplement.material-4/images(Cell Scratch Assay)- HUVEC-0H/0-PL20X4.jpg]

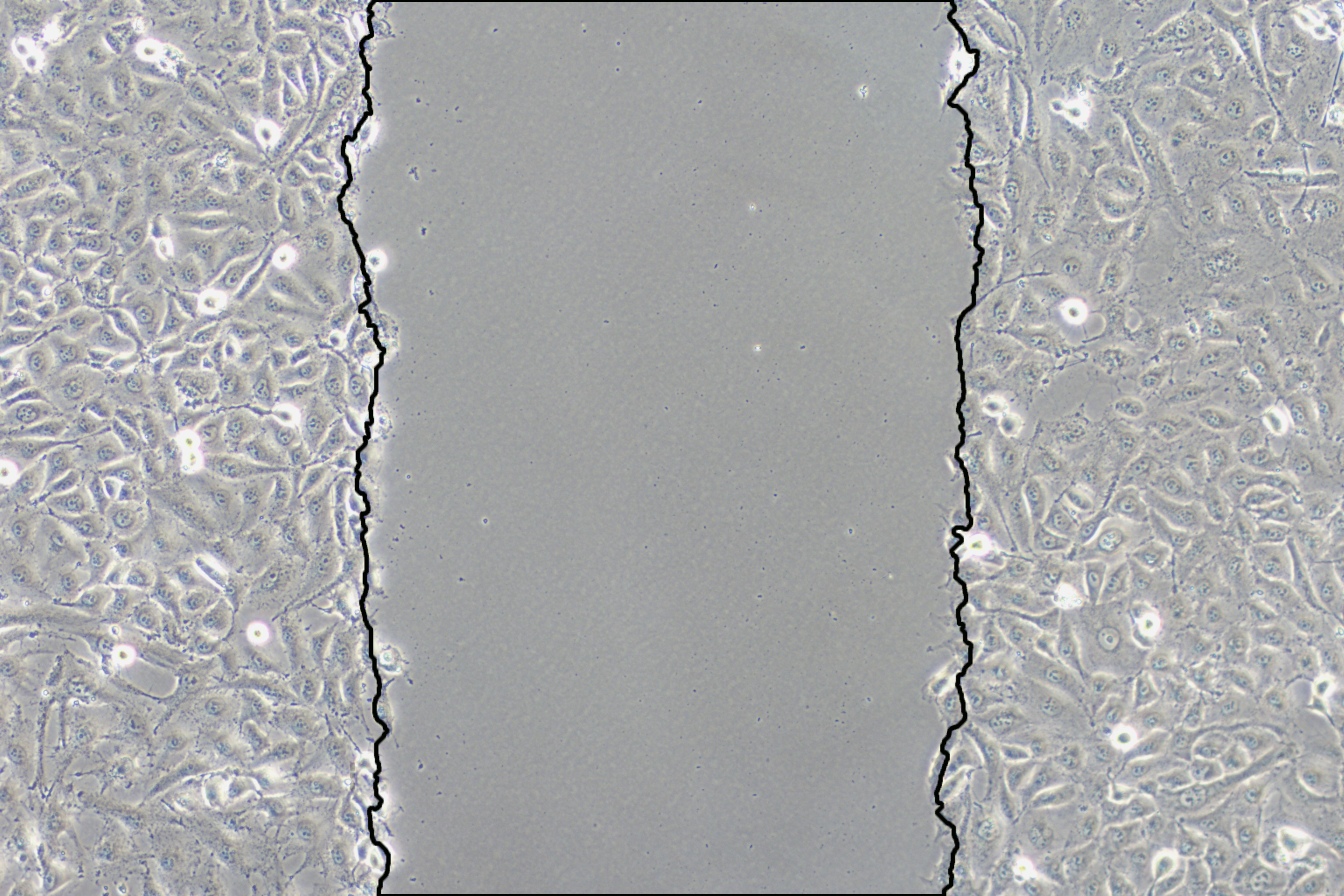

Supplement: S4 File — (ZIP) [file pone.0324264.s004.zip › supplement.material-4/images(Cell Scratch Assay)- HUVEC-0H/0-PL20X5.jpg]

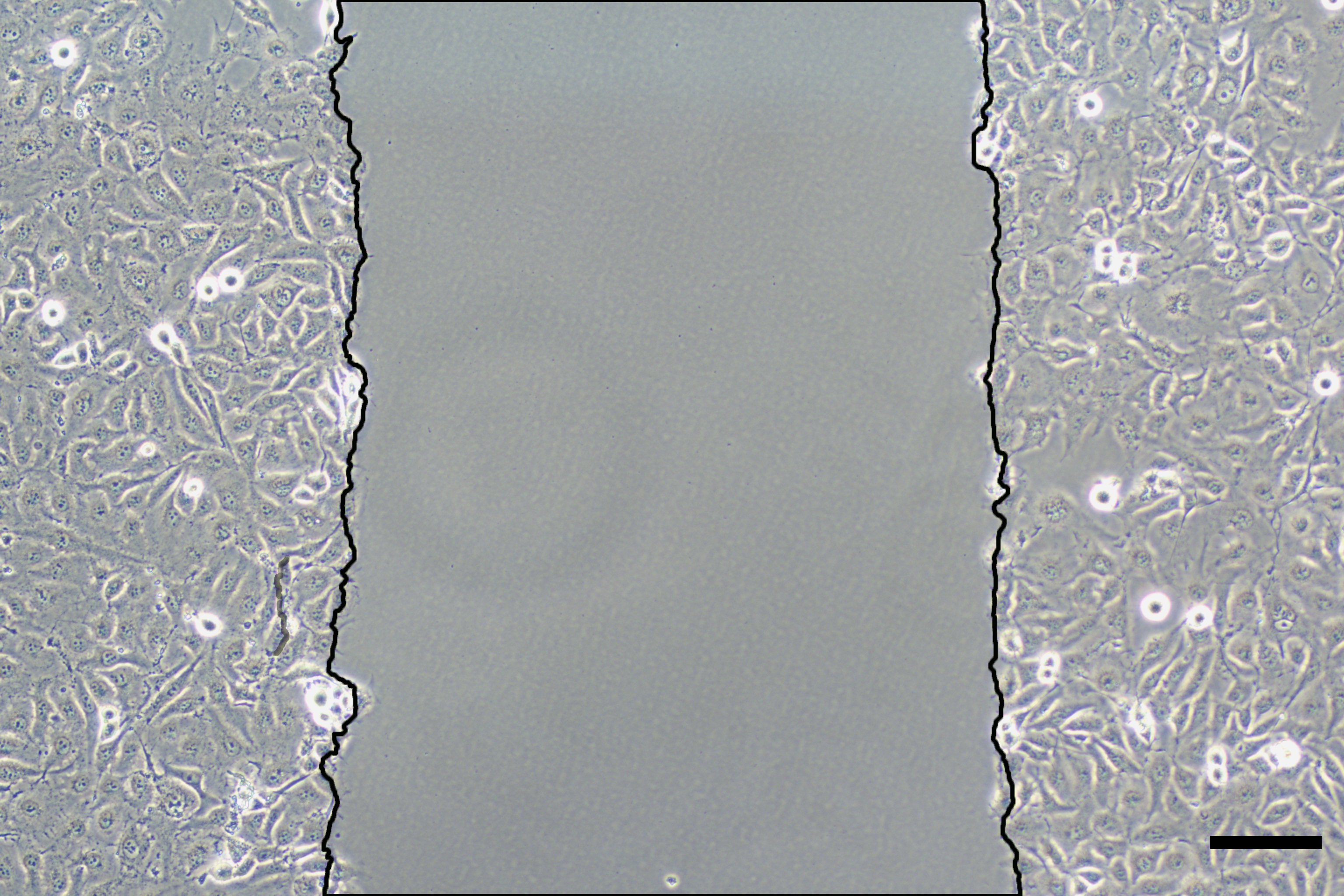

Supplement: S4 File — (ZIP) [file pone.0324264.s004.zip › supplement.material-4/images(Cell Scratch Assay)- HUVEC-0H/0h-Control1-.jpg]

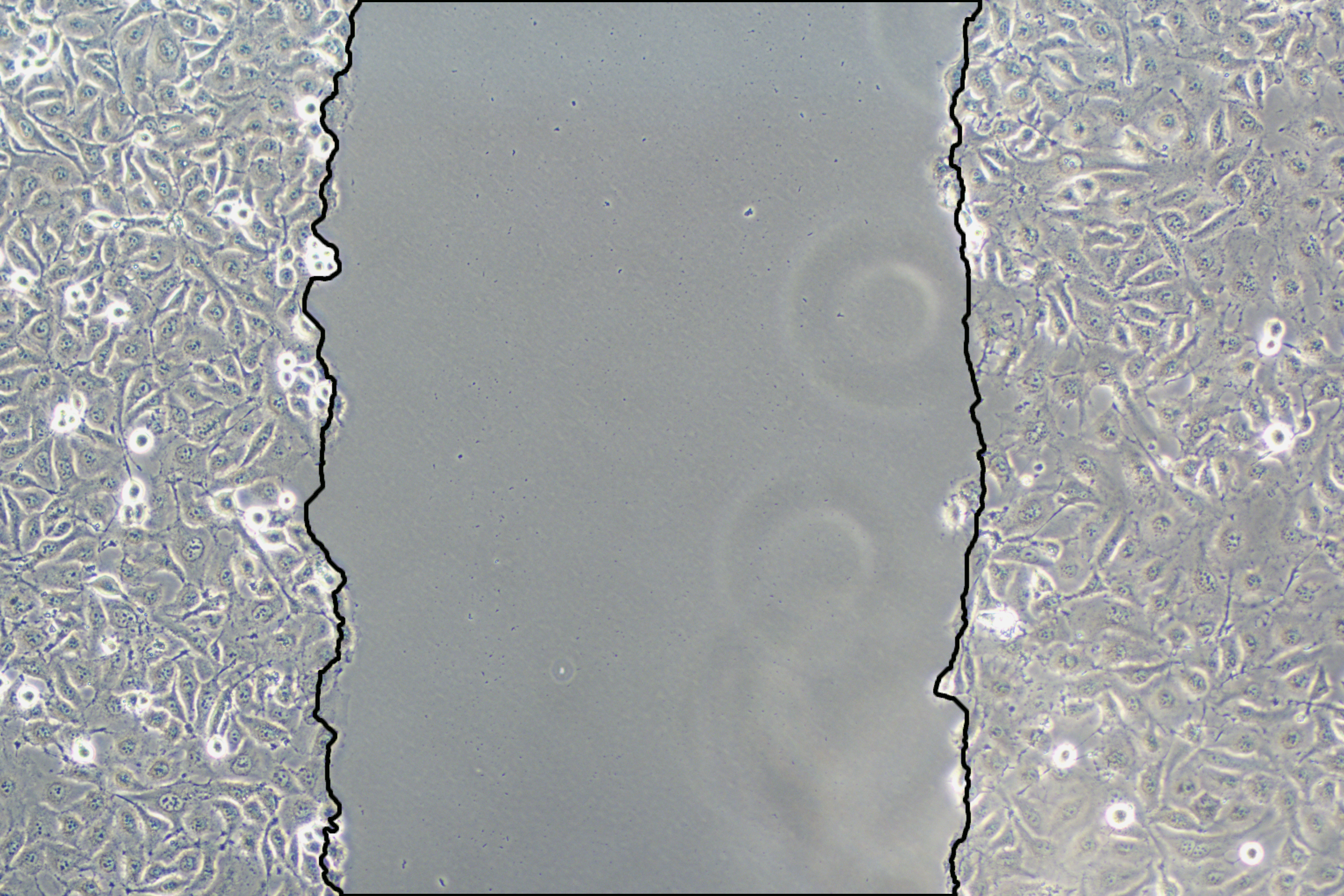

Supplement: S4 File — (ZIP) [file pone.0324264.s004.zip › supplement.material-4/images(Cell Scratch Assay)- HUVEC-0H/0h-Control2.jpg]

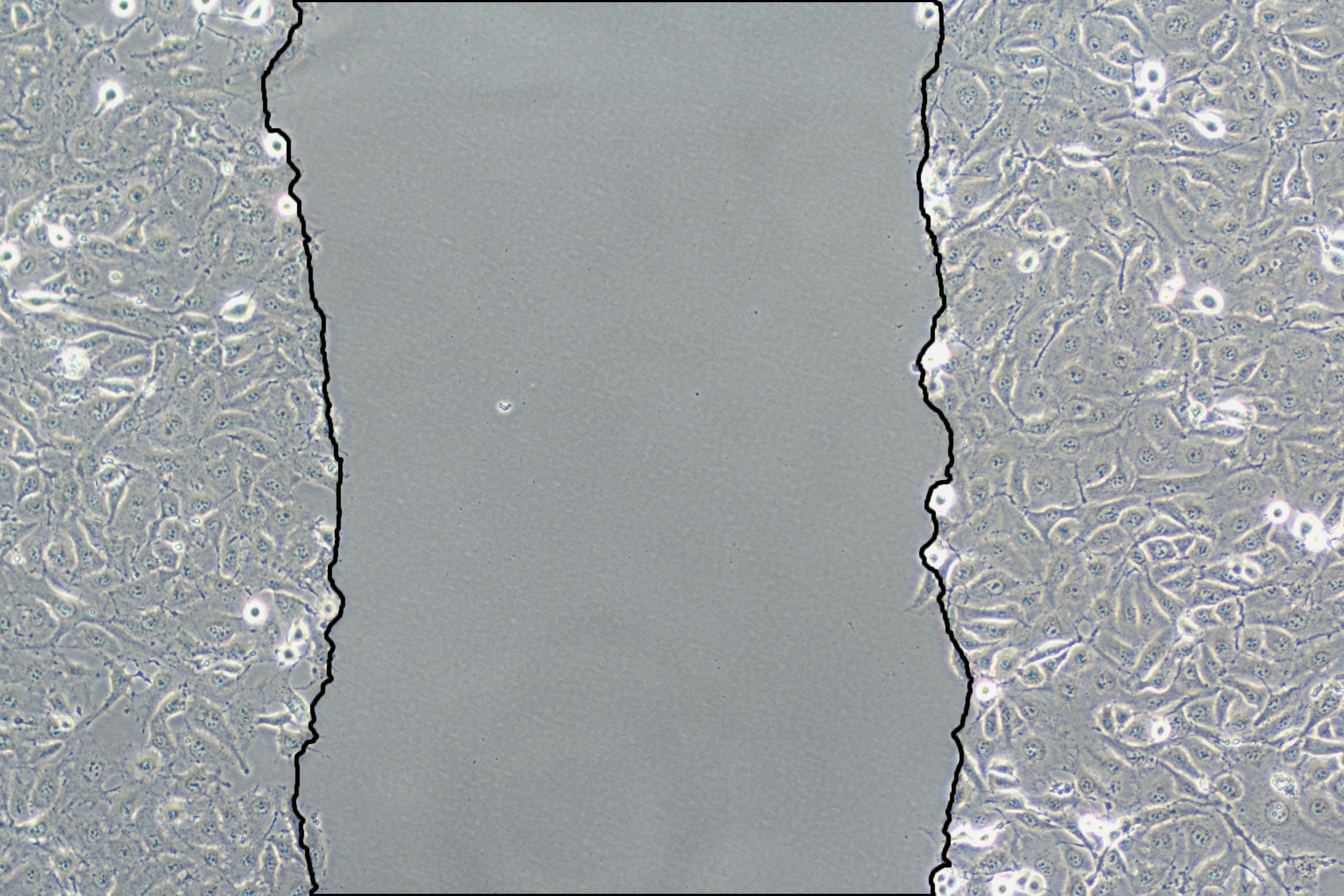

Supplement: S4 File — (ZIP) [file pone.0324264.s004.zip › supplement.material-4/images(Cell Scratch Assay)- HUVEC-0H/0h-Control3.jpg]

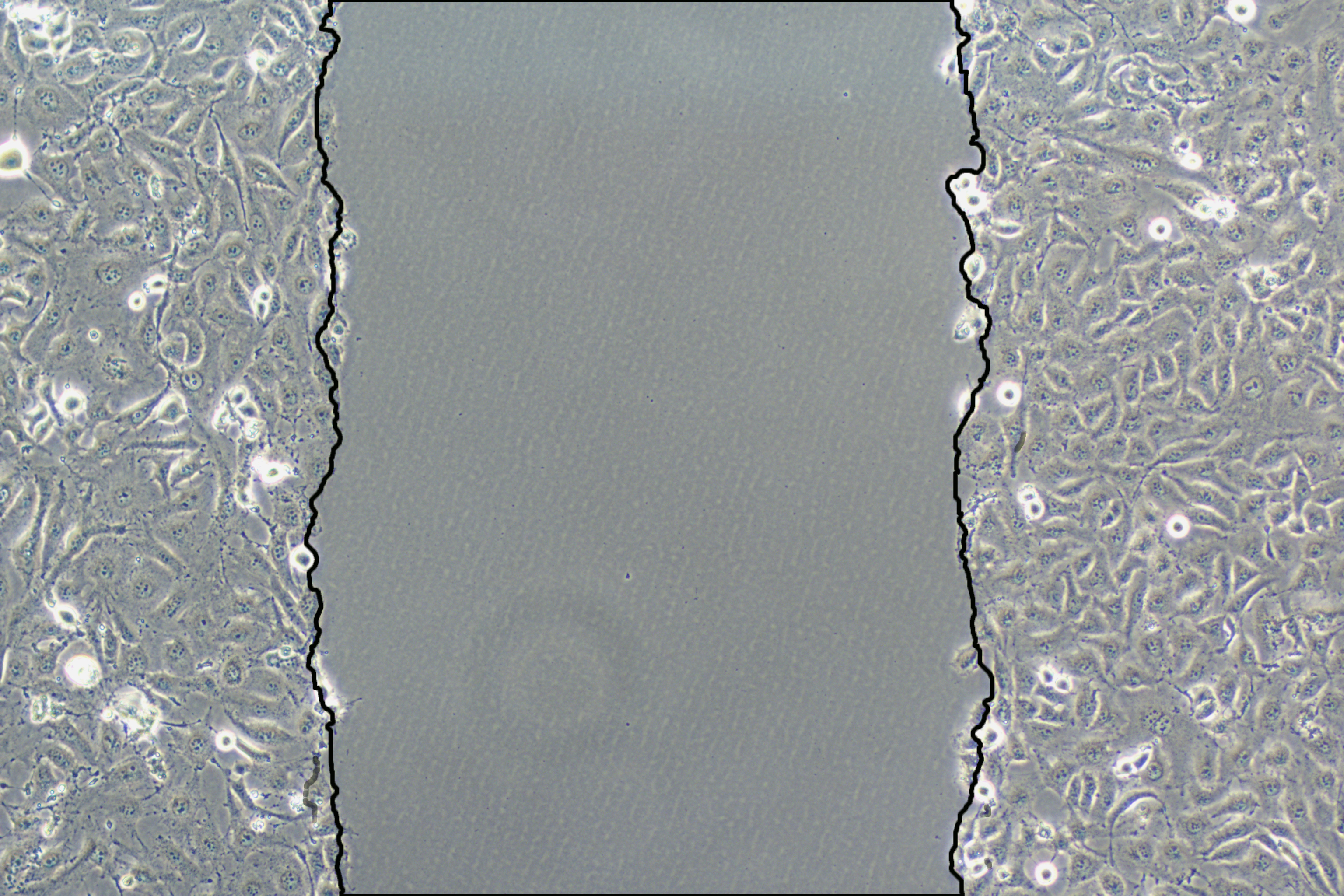

Supplement: S4 File — (ZIP) [file pone.0324264.s004.zip › supplement.material-4/images(Cell Scratch Assay)- HUVEC-0H/0h-Control4.jpg]

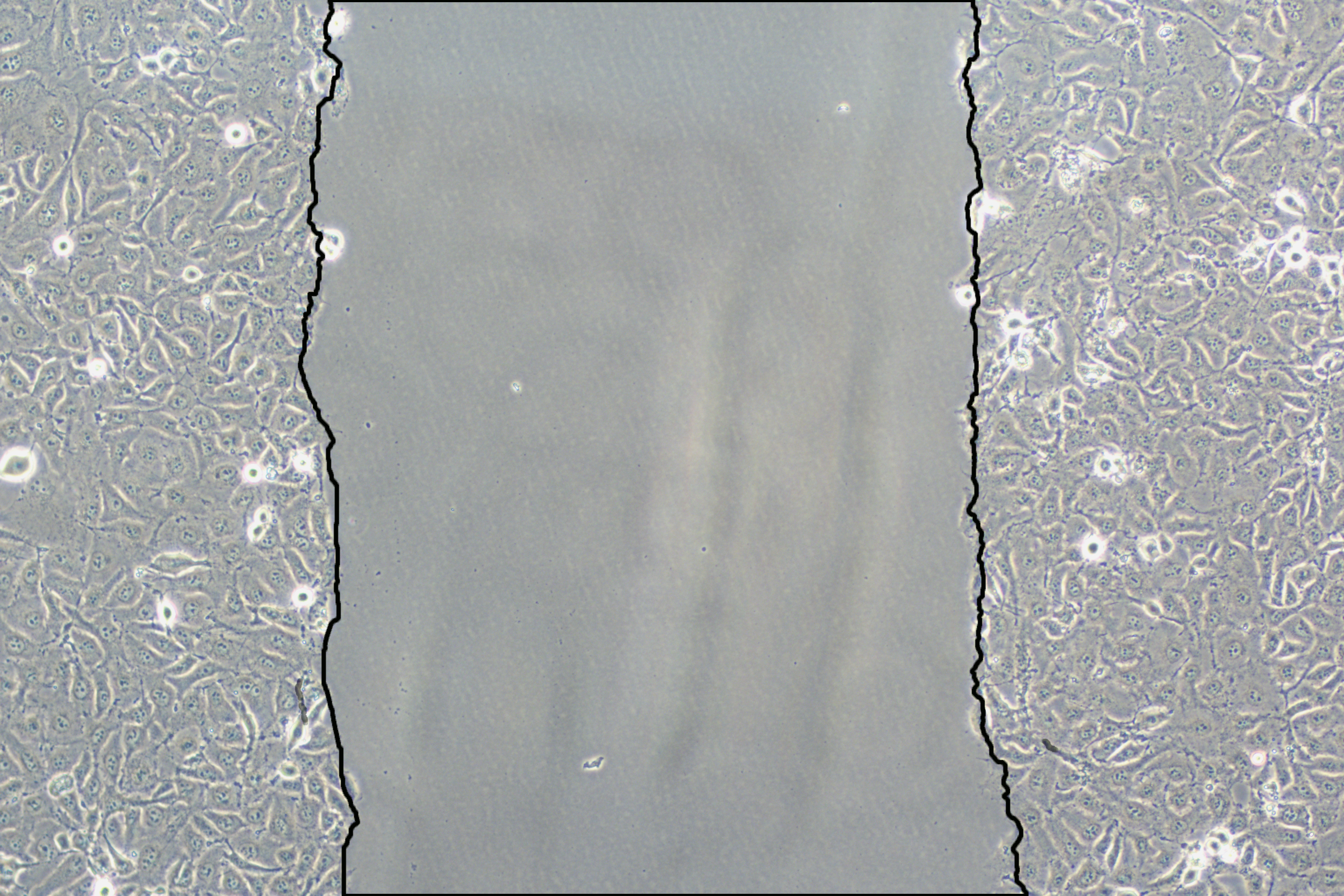

Supplement: S4 File — (ZIP) [file pone.0324264.s004.zip › supplement.material-4/images(Cell Scratch Assay)- HUVEC-0H/0h-Control5.jpg]

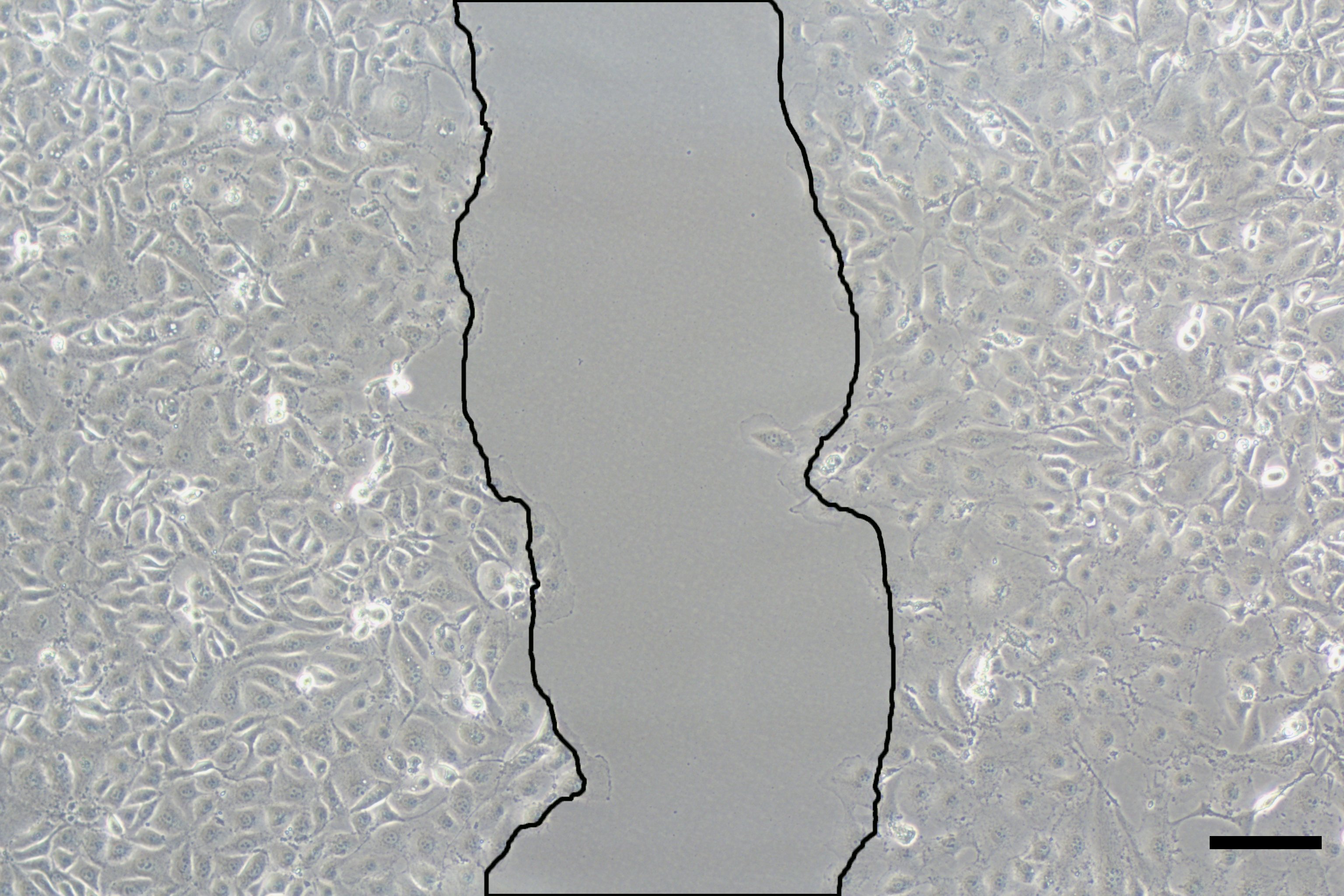

Supplement: S5 File — (ZIP) [file pone.0324264.s005.zip › supplement.material-5/images(Cell Scratch Assay)- HUVEC-12H/12-control1-.jpg]

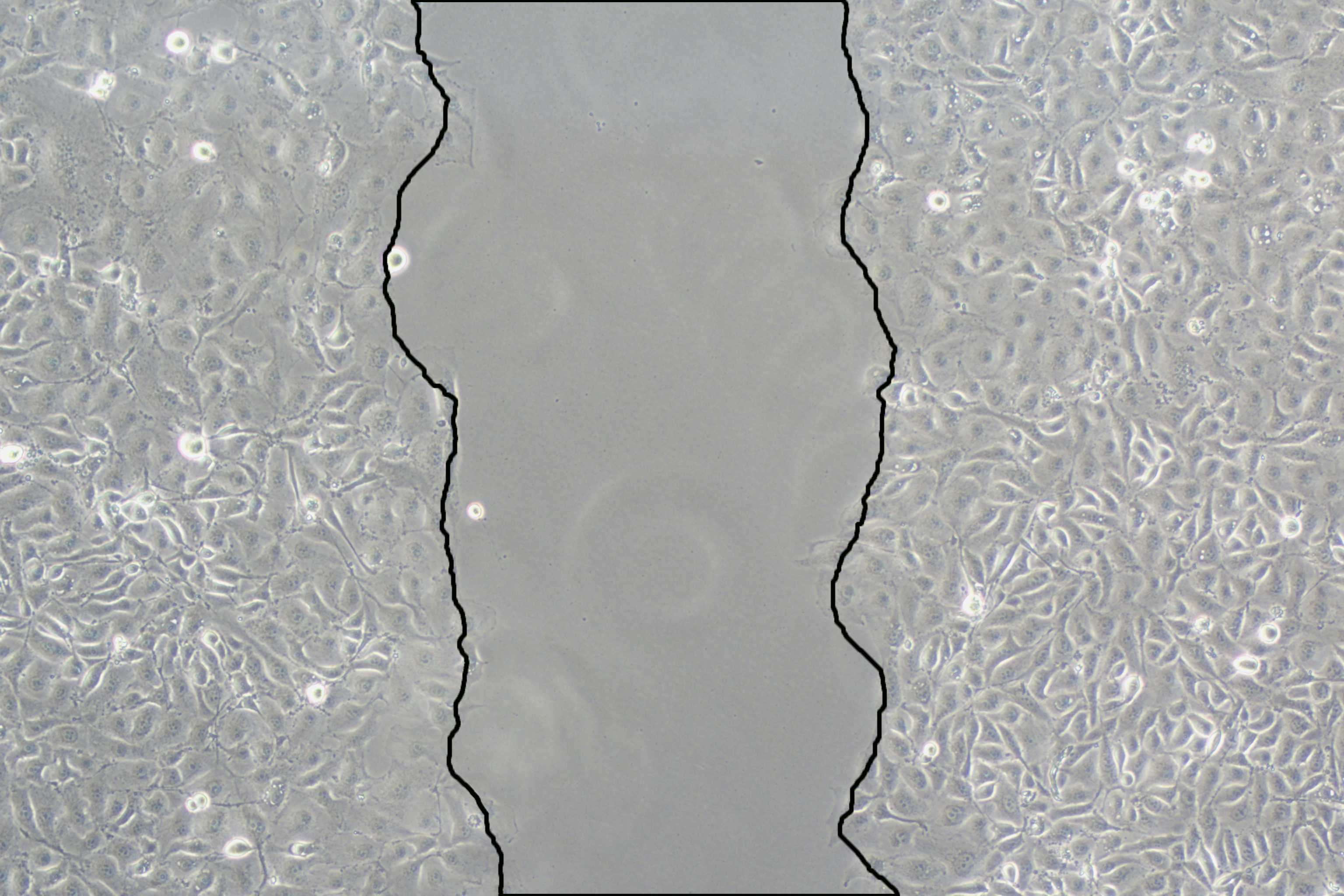

Supplement: S5 File — (ZIP) [file pone.0324264.s005.zip › supplement.material-5/images(Cell Scratch Assay)- HUVEC-12H/12-control2.jpg]

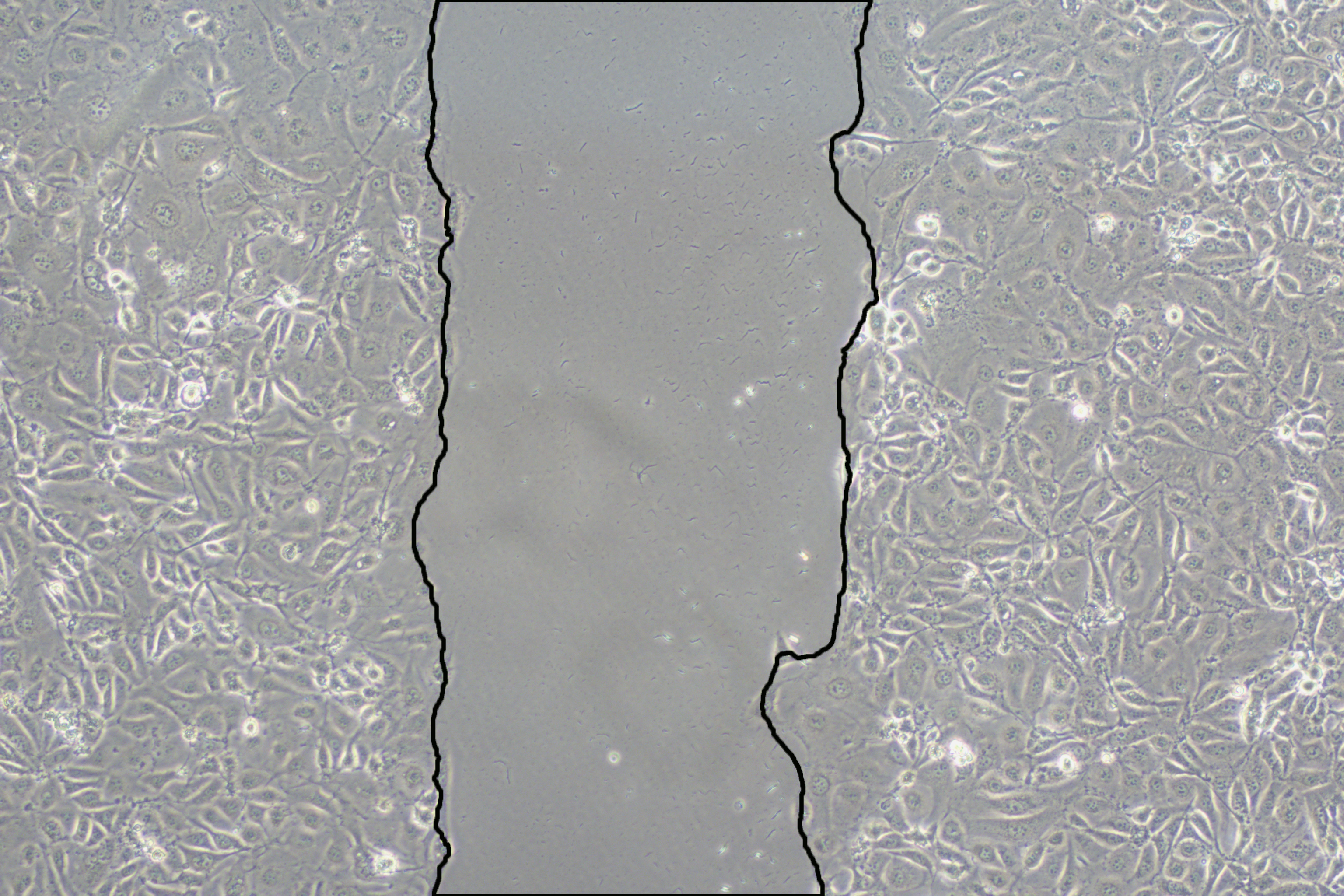

Supplement: S5 File — (ZIP) [file pone.0324264.s005.zip › supplement.material-5/images(Cell Scratch Assay)- HUVEC-12H/12-control3.jpg]

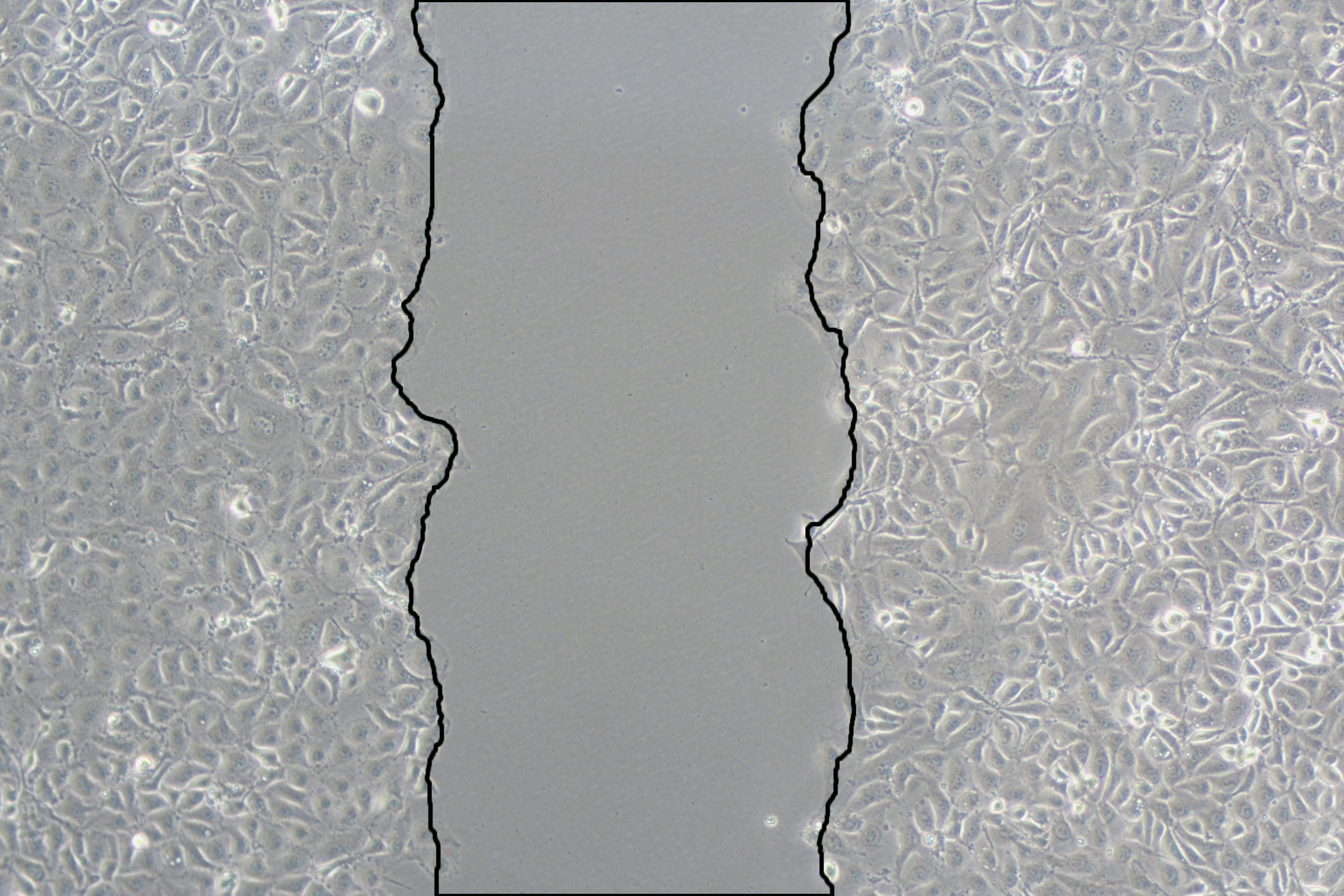

Supplement: S5 File — (ZIP) [file pone.0324264.s005.zip › supplement.material-5/images(Cell Scratch Assay)- HUVEC-12H/12-control4.jpg]

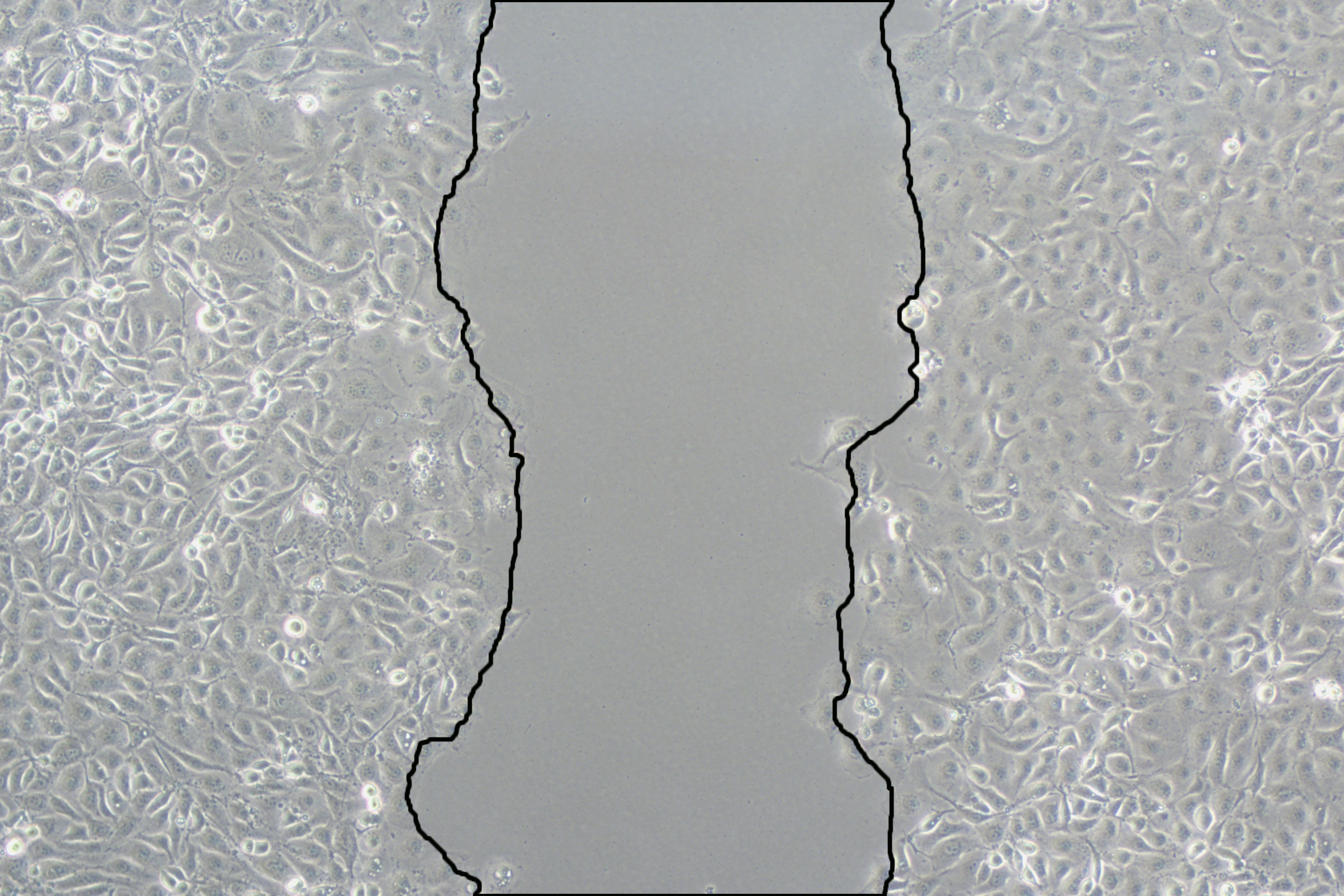

Supplement: S5 File — (ZIP) [file pone.0324264.s005.zip › supplement.material-5/images(Cell Scratch Assay)- HUVEC-12H/12-control5.jpg]

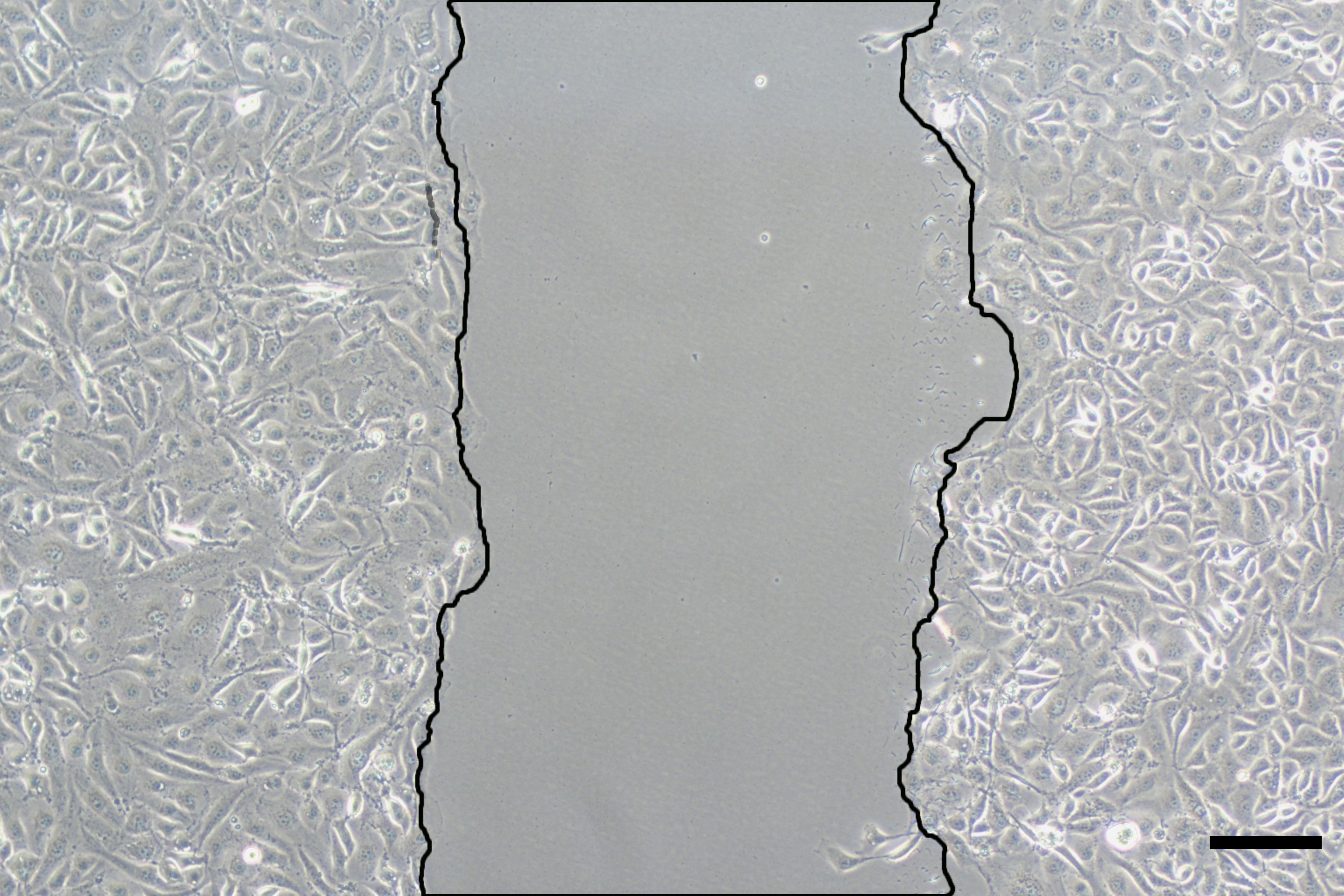

Supplement: S5 File — (ZIP) [file pone.0324264.s005.zip › supplement.material-5/images(Cell Scratch Assay)- HUVEC-12H/12-Model1-.jpg]

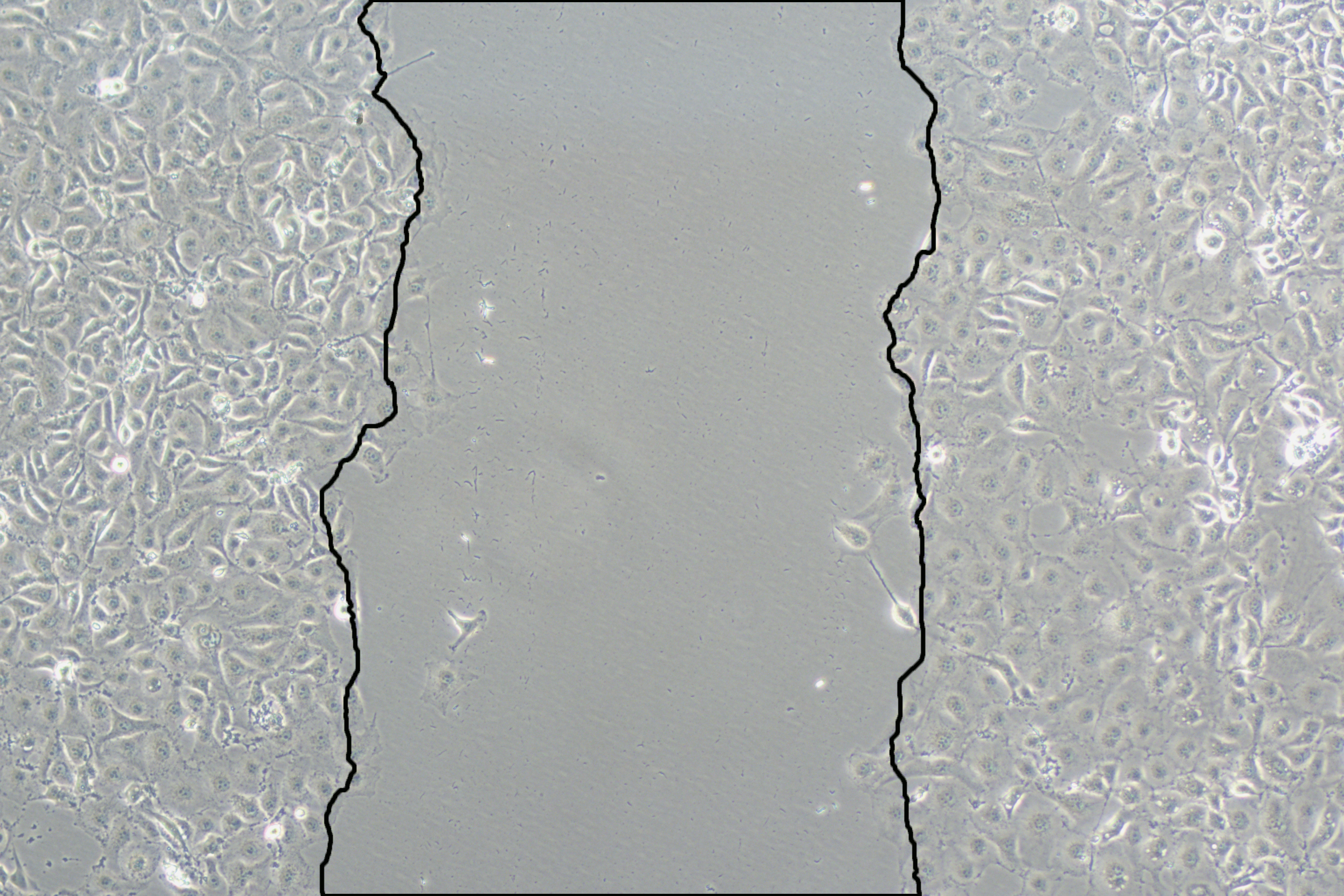

Supplement: S5 File — (ZIP) [file pone.0324264.s005.zip › supplement.material-5/images(Cell Scratch Assay)- HUVEC-12H/12-Model2.jpg]

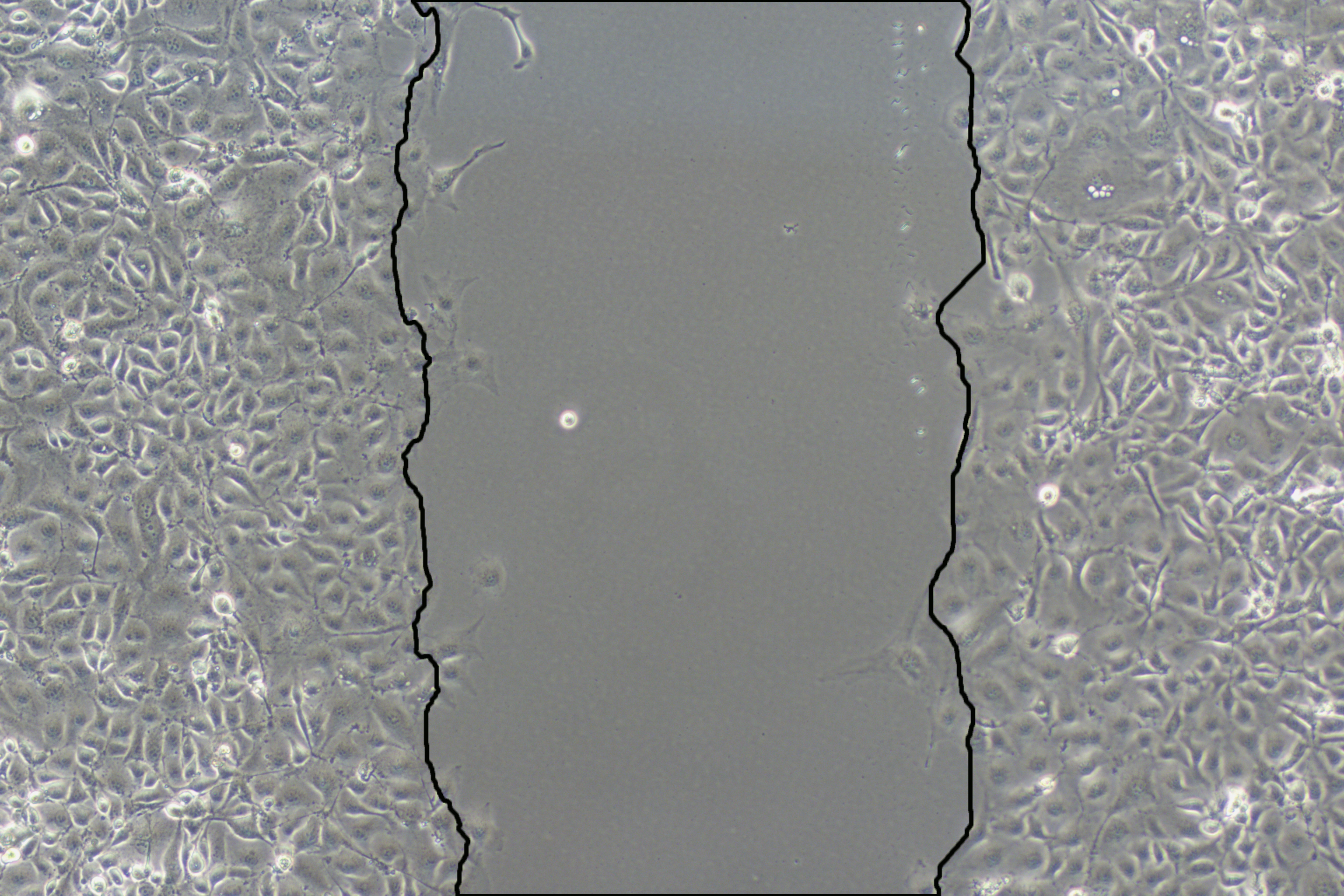

Supplement: S5 File — (ZIP) [file pone.0324264.s005.zip › supplement.material-5/images(Cell Scratch Assay)- HUVEC-12H/12-Model3.jpg]

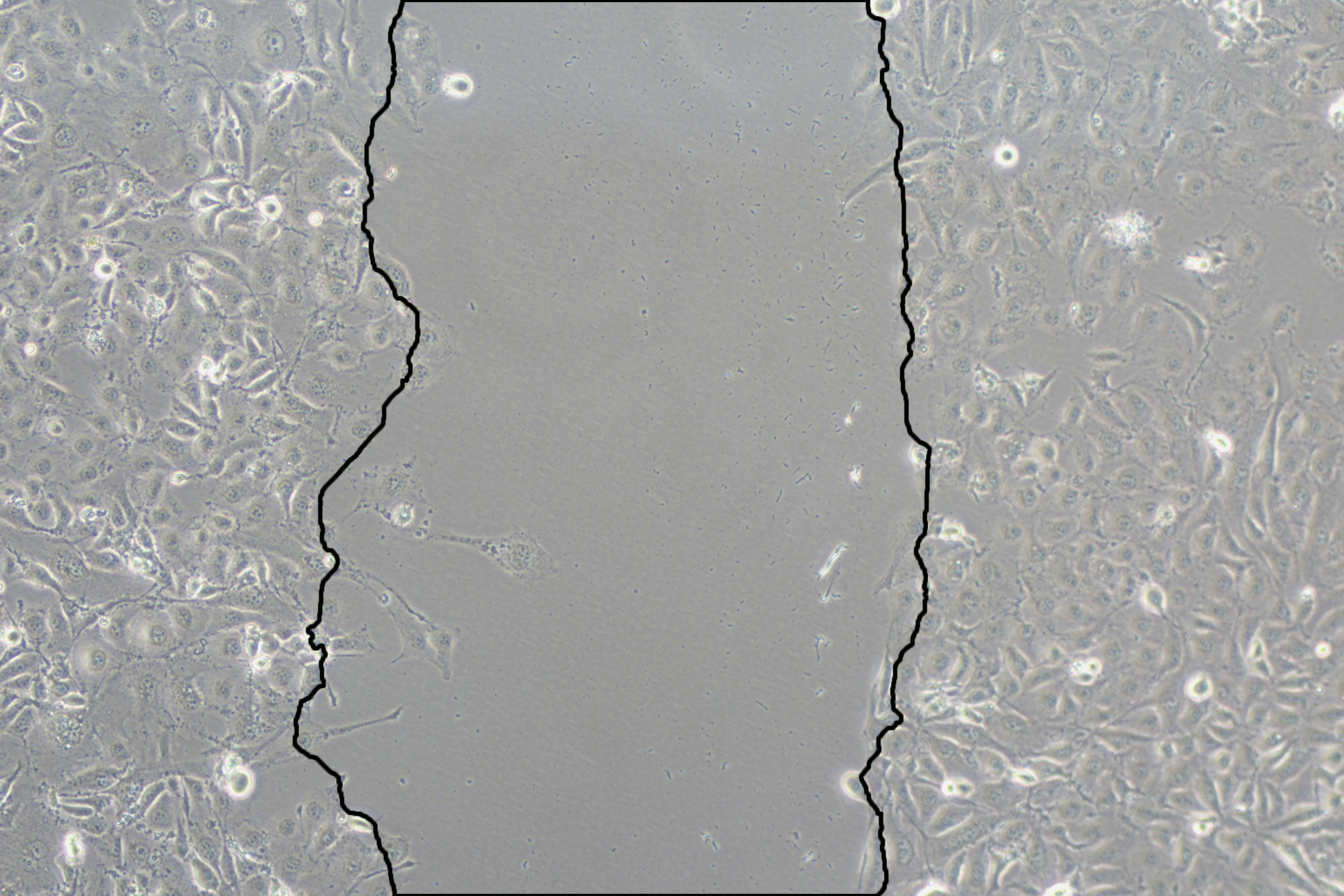

Supplement: S5 File — (ZIP) [file pone.0324264.s005.zip › supplement.material-5/images(Cell Scratch Assay)- HUVEC-12H/12-Model4.jpg]

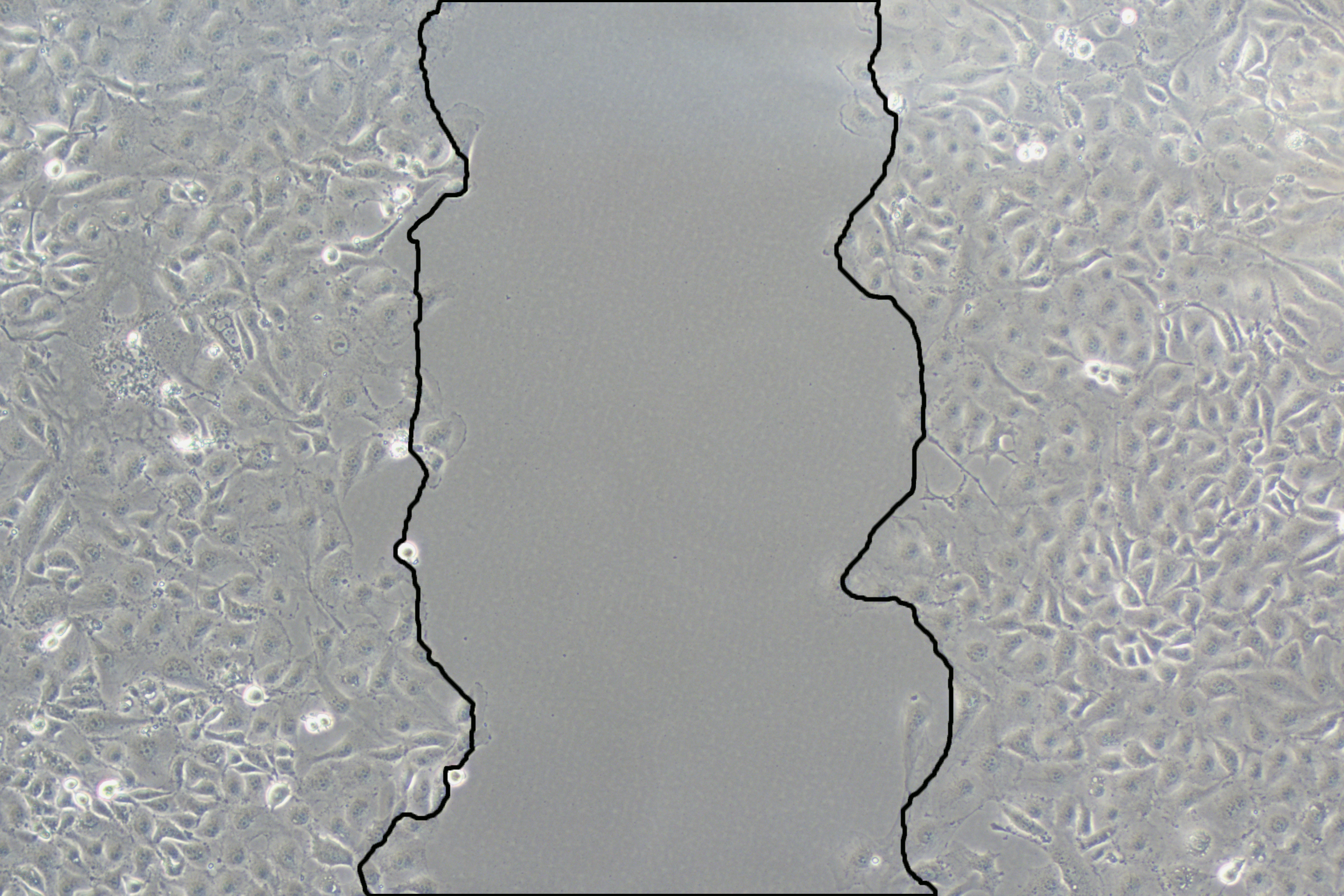

Supplement: S5 File — (ZIP) [file pone.0324264.s005.zip › supplement.material-5/images(Cell Scratch Assay)- HUVEC-12H/12-Model5.jpg]

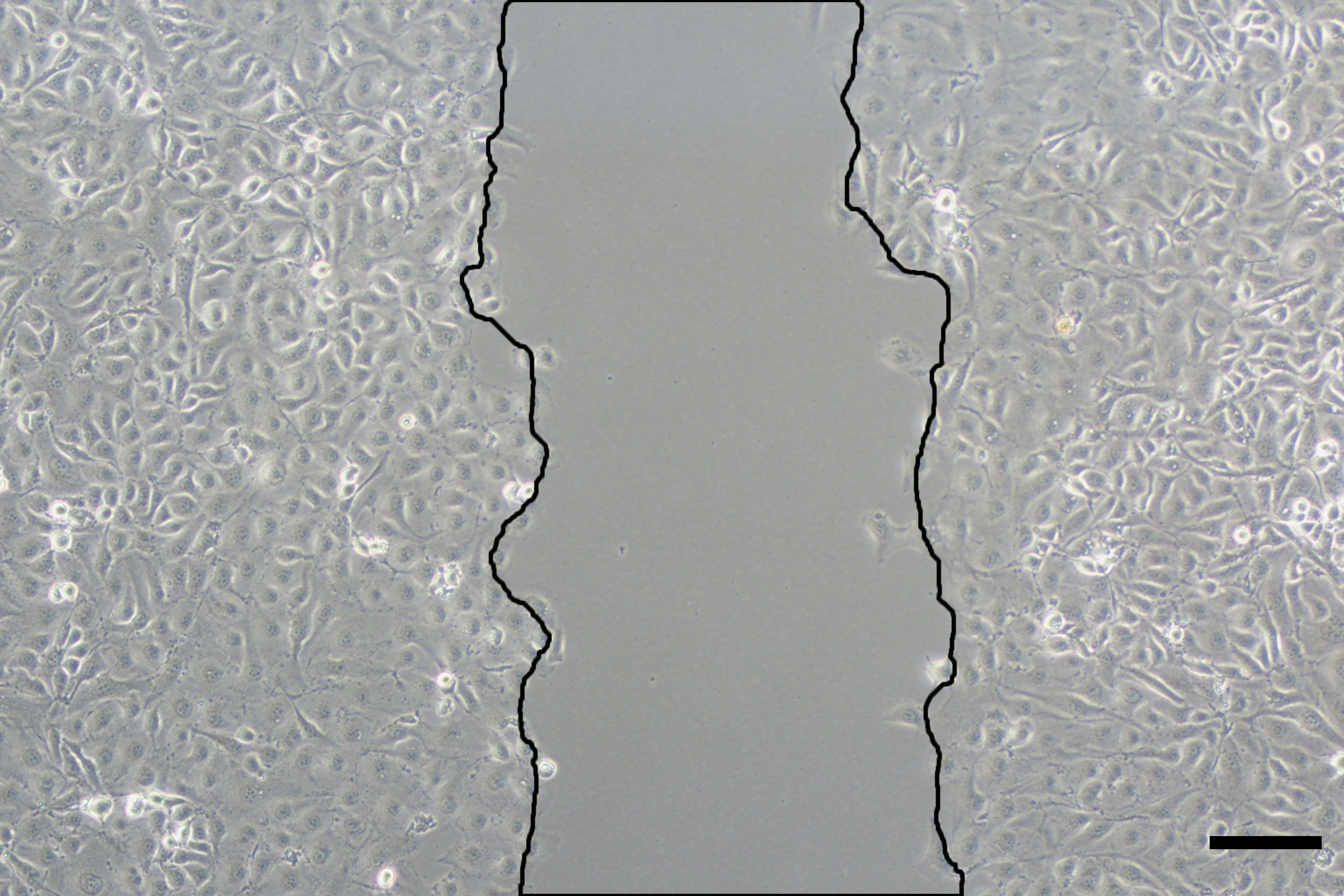

Supplement: S5 File — (ZIP) [file pone.0324264.s005.zip › supplement.material-5/images(Cell Scratch Assay)- HUVEC-12H/12-PL10X1-.jpg]
