# Supplementary figures and images for: Platelet lysate-sodium hyaluronate gel promotes diabetic foot wound healing by regulating oxidative stress and autophagy (part 4 of 4)
Source: PLoS One. 2025 Jun 6;20(6):e0324264. doi: 10.1371/journal.pone.0324264 (PMC12143543; doi:10.1371/journal.pone.0324264)

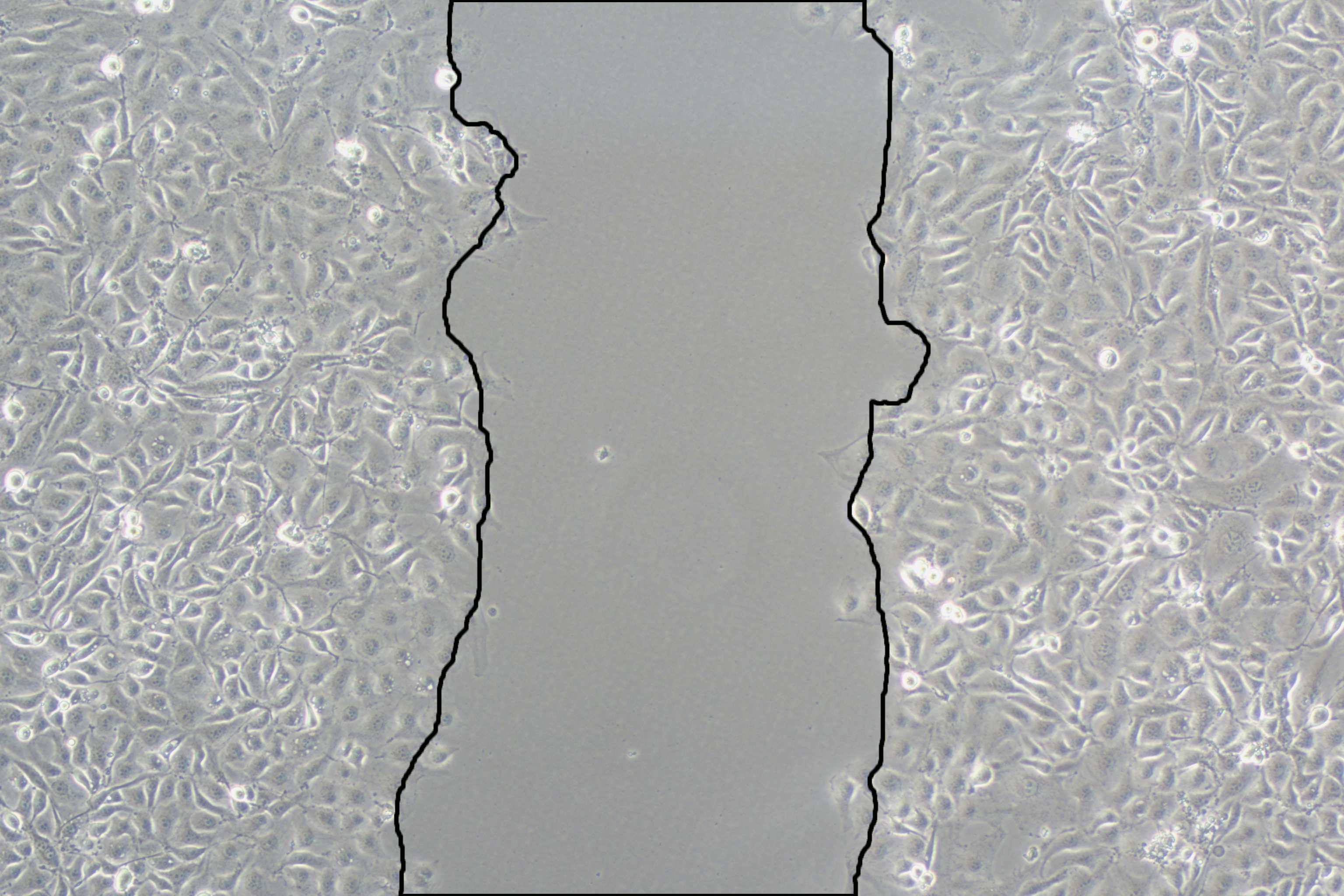

Supplement: S5 File — (ZIP) [file pone.0324264.s005.zip › supplement.material-5/images(Cell Scratch Assay)- HUVEC-12H/12-PL10X2.jpg]

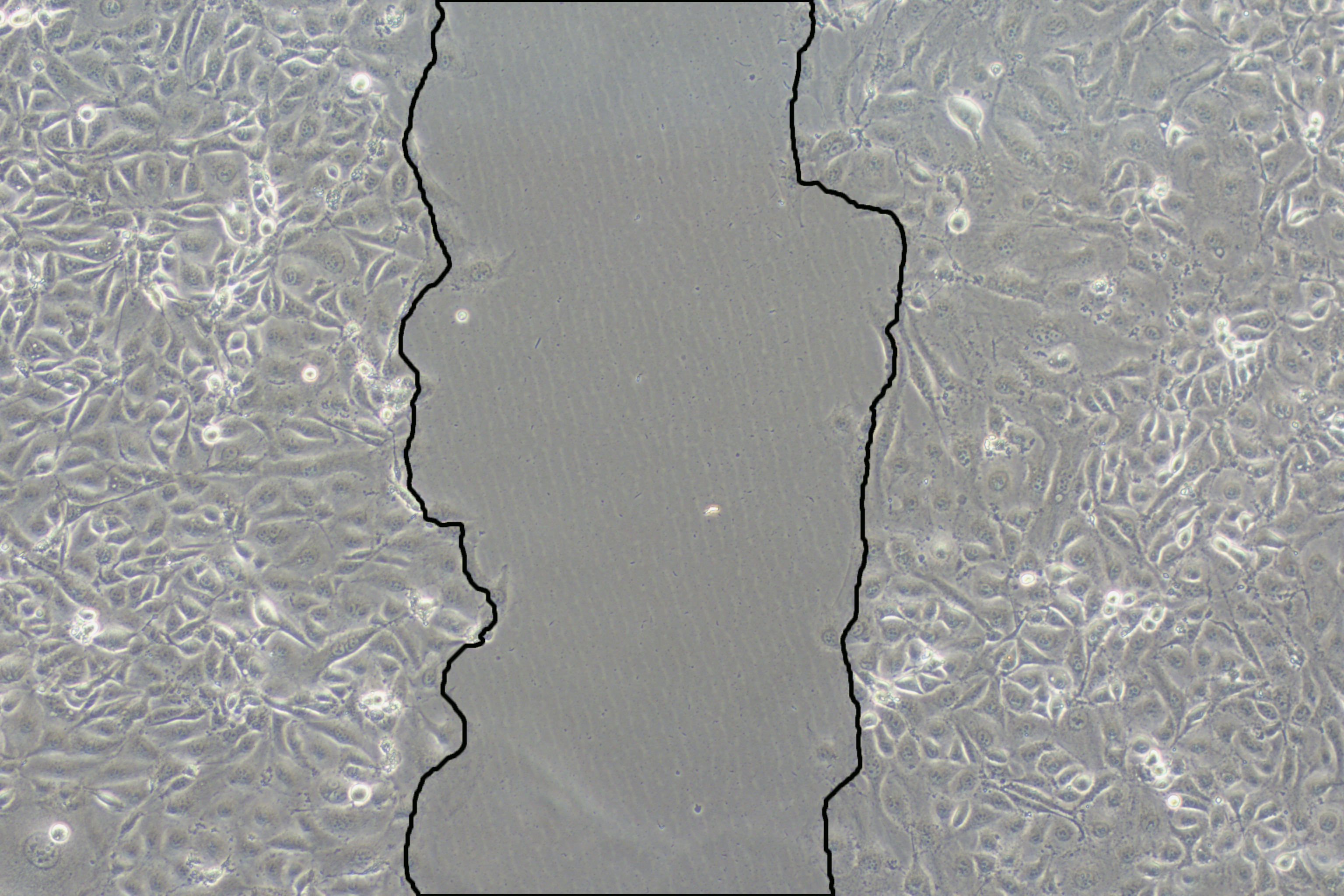

Supplement: S5 File — (ZIP) [file pone.0324264.s005.zip › supplement.material-5/images(Cell Scratch Assay)- HUVEC-12H/12-PL10X3.jpg]

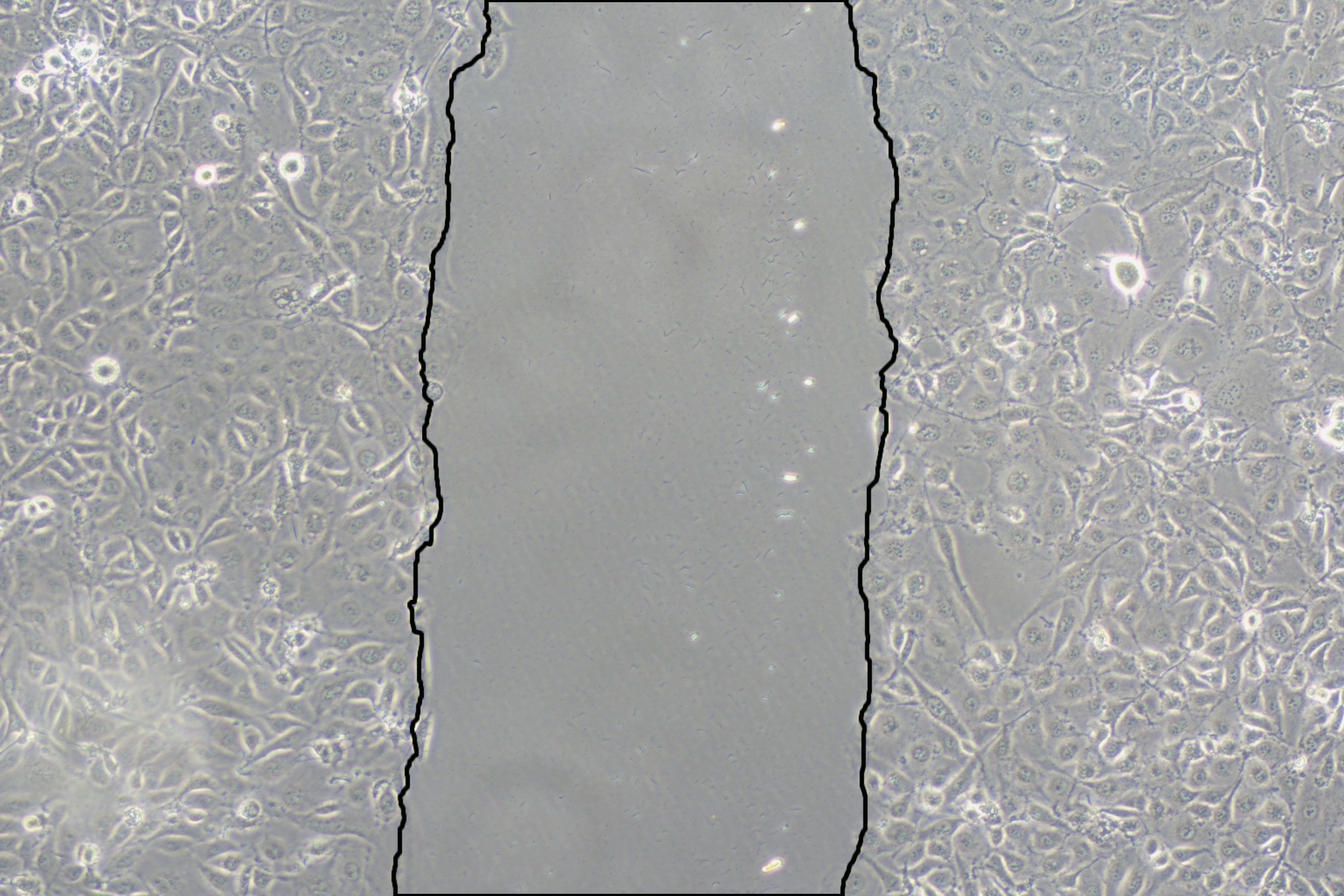

Supplement: S5 File — (ZIP) [file pone.0324264.s005.zip › supplement.material-5/images(Cell Scratch Assay)- HUVEC-12H/12-PL10X4.jpg]

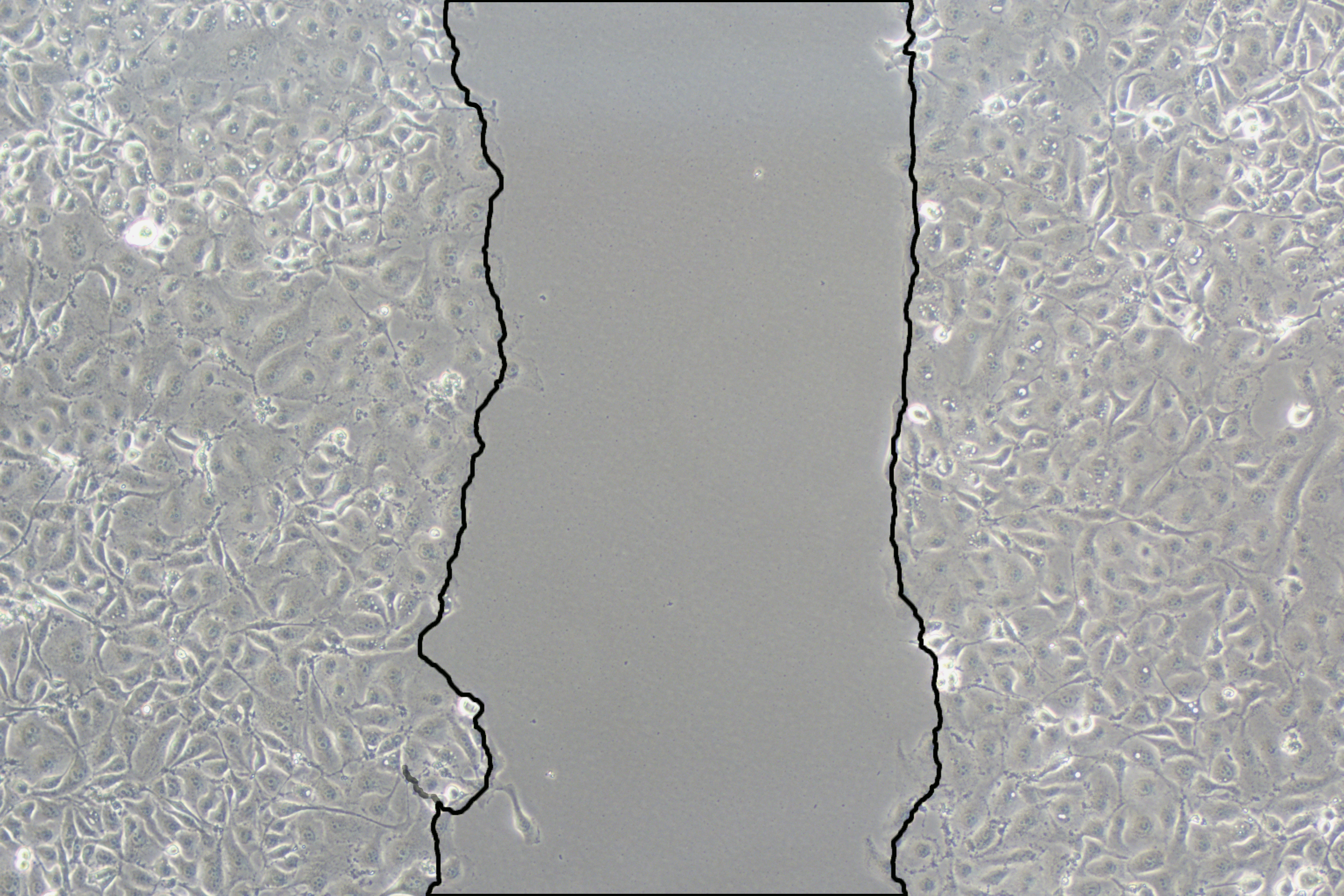

Supplement: S5 File — (ZIP) [file pone.0324264.s005.zip › supplement.material-5/images(Cell Scratch Assay)- HUVEC-12H/12-PL10X5.jpg]

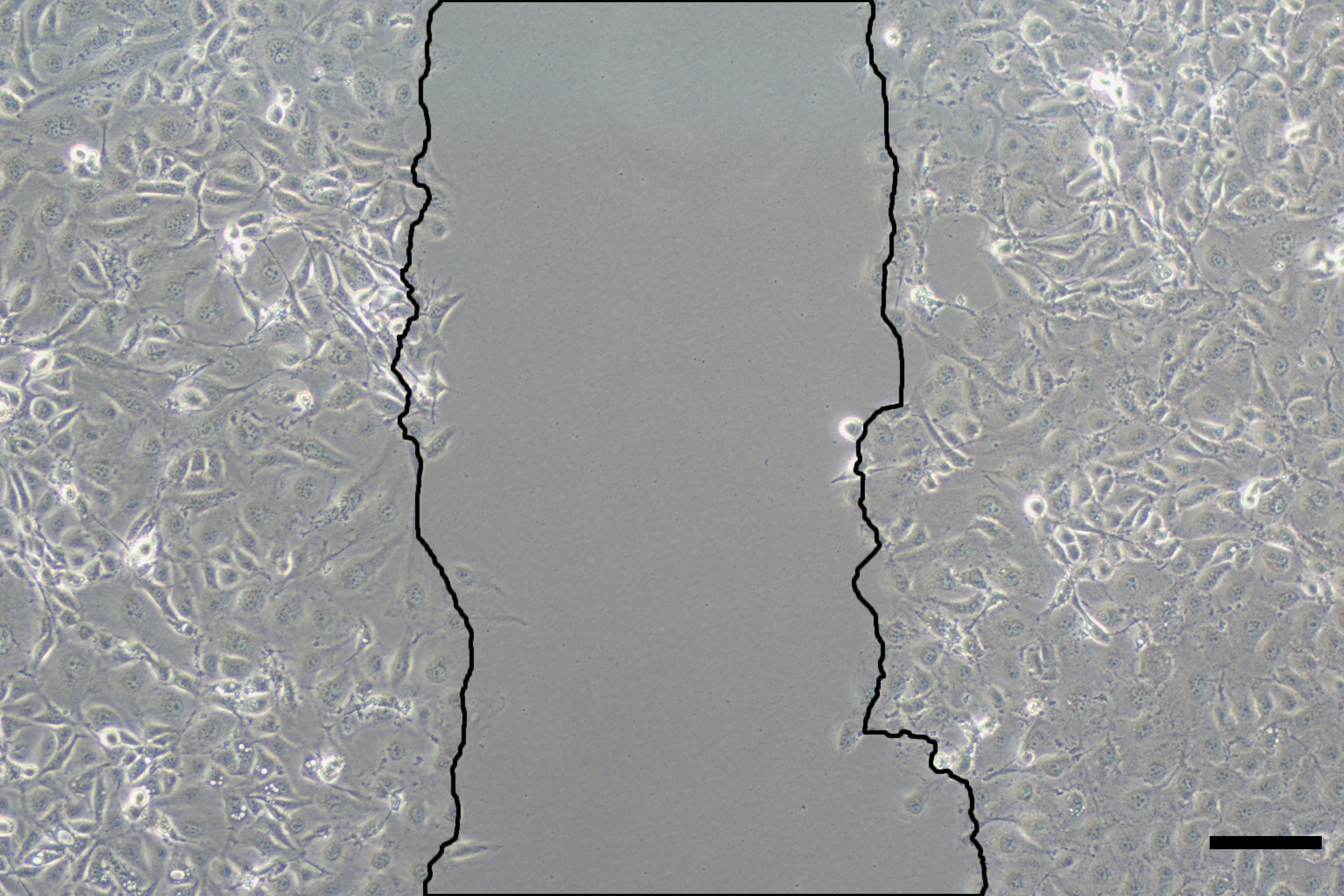

Supplement: S5 File — (ZIP) [file pone.0324264.s005.zip › supplement.material-5/images(Cell Scratch Assay)- HUVEC-12H/12-PL20X1-.jpg]

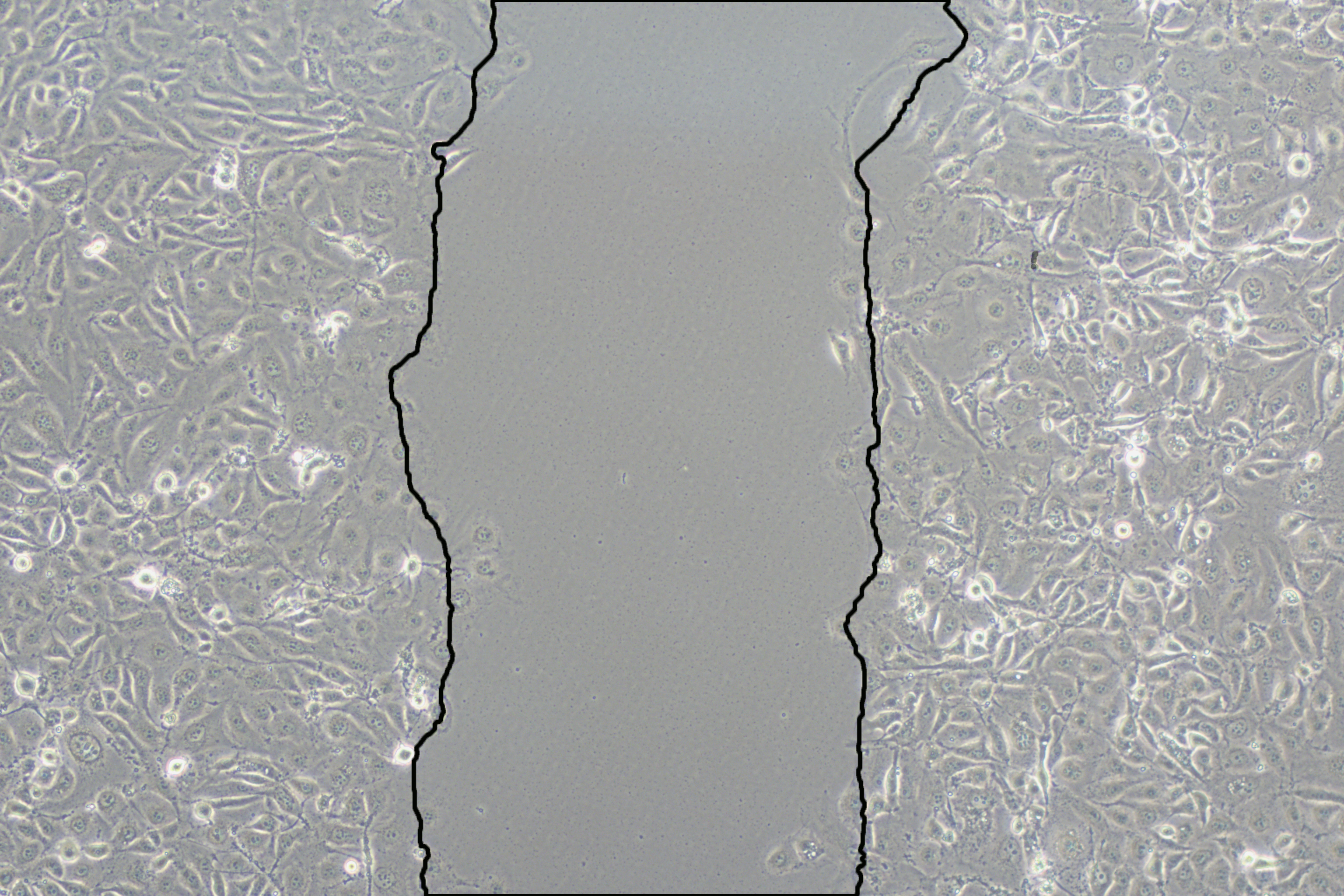

Supplement: S5 File — (ZIP) [file pone.0324264.s005.zip › supplement.material-5/images(Cell Scratch Assay)- HUVEC-12H/12-PL20X2.jpg]

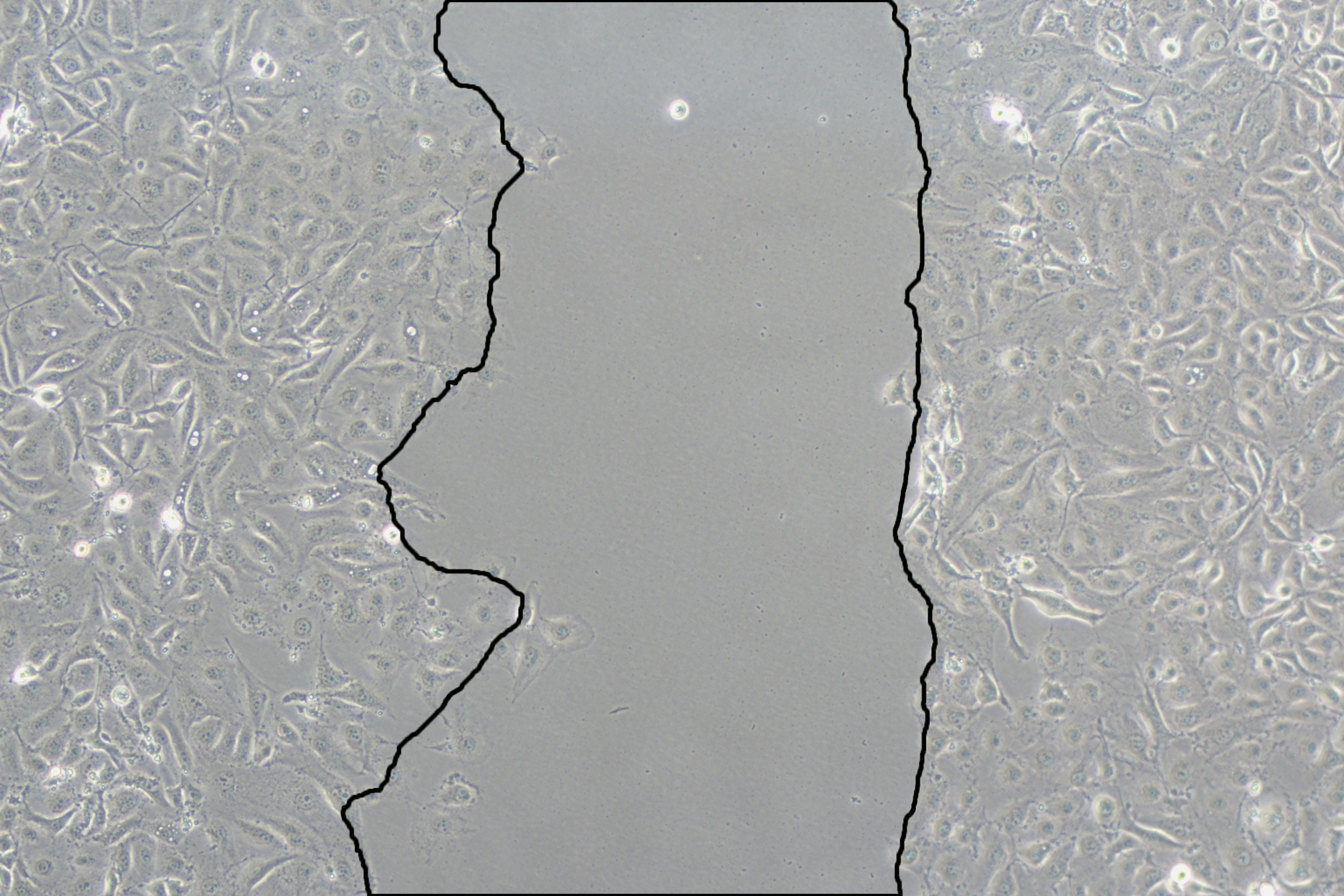

Supplement: S5 File — (ZIP) [file pone.0324264.s005.zip › supplement.material-5/images(Cell Scratch Assay)- HUVEC-12H/12-PL20X3.jpg]

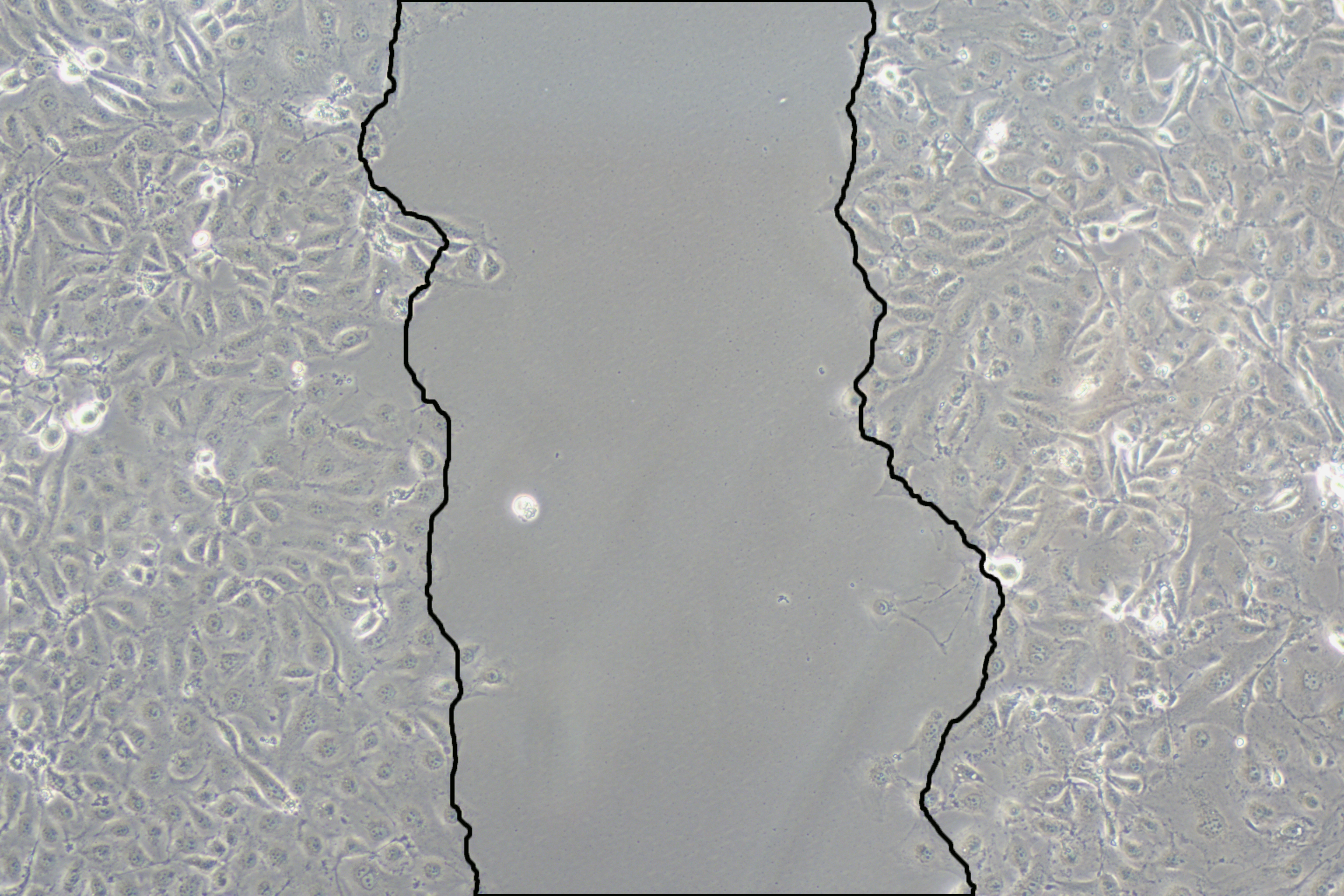

Supplement: S5 File — (ZIP) [file pone.0324264.s005.zip › supplement.material-5/images(Cell Scratch Assay)- HUVEC-12H/12-PL20X4.jpg]

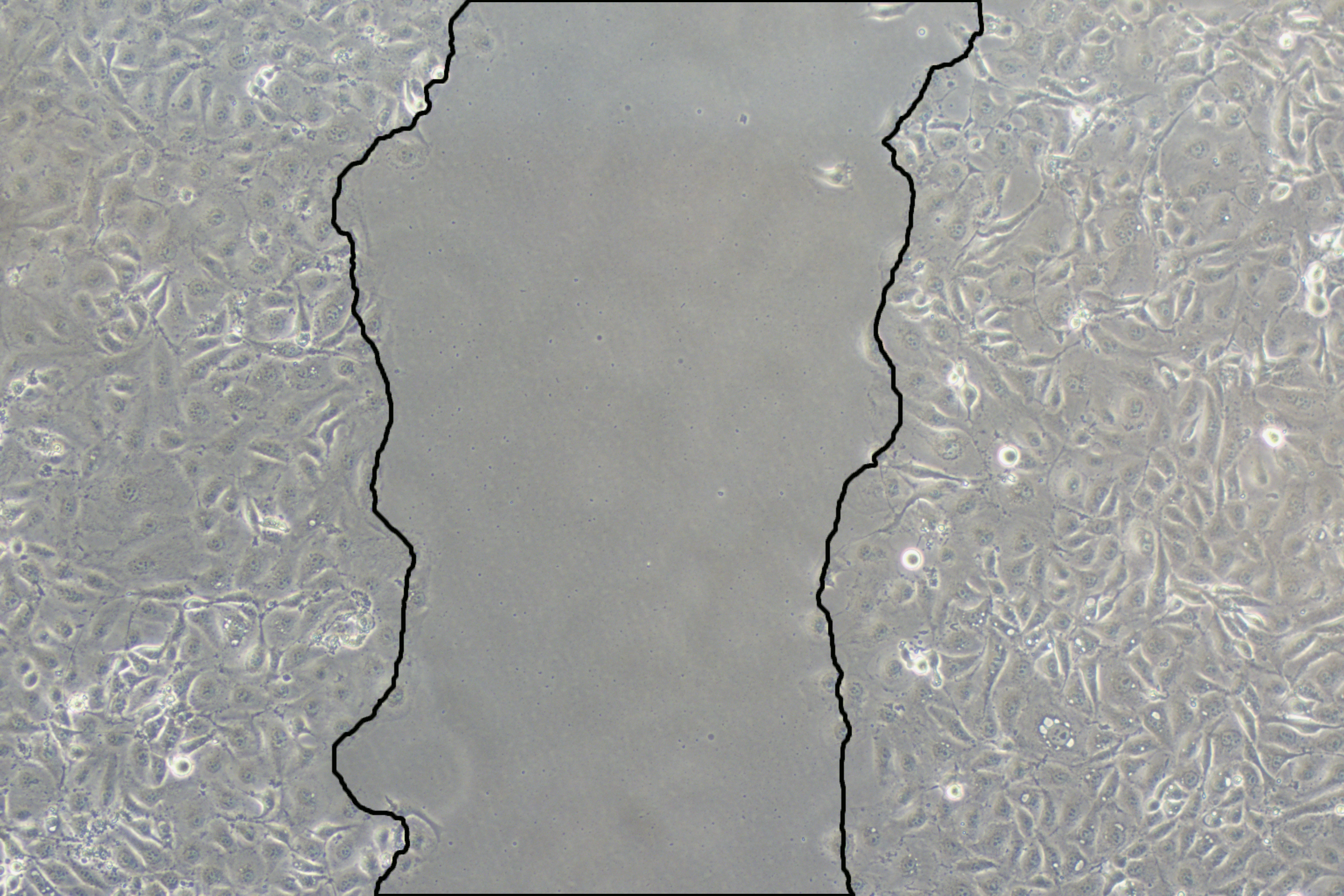

Supplement: S5 File — (ZIP) [file pone.0324264.s005.zip › supplement.material-5/images(Cell Scratch Assay)- HUVEC-12H/12-PL20X5.jpg]

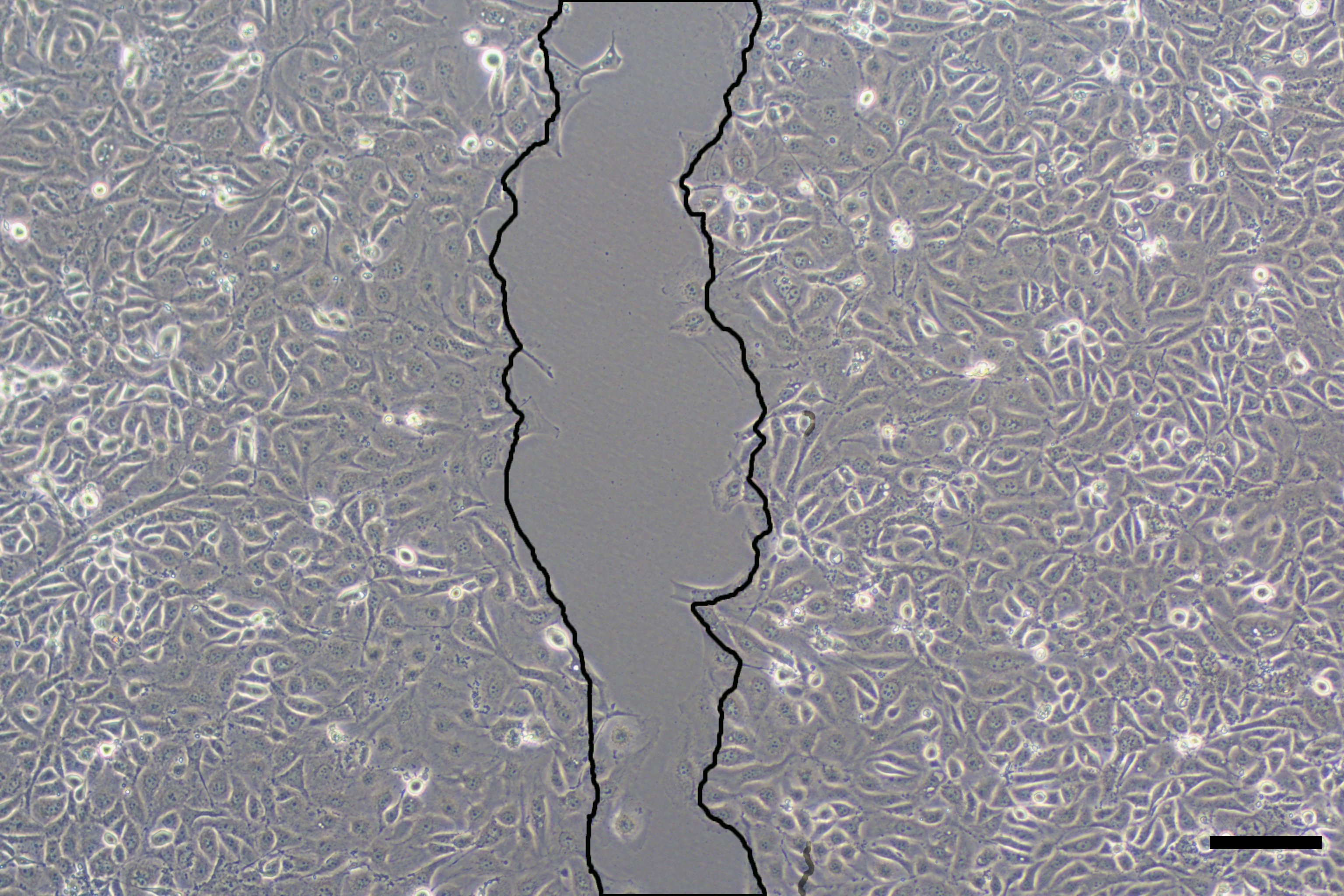

Supplement: S6 File — (ZIP) [file pone.0324264.s006.zip › supplement.material-6/images(Cell Scratch Assay)- HUVEC-24H/24-Control1-.jpg]

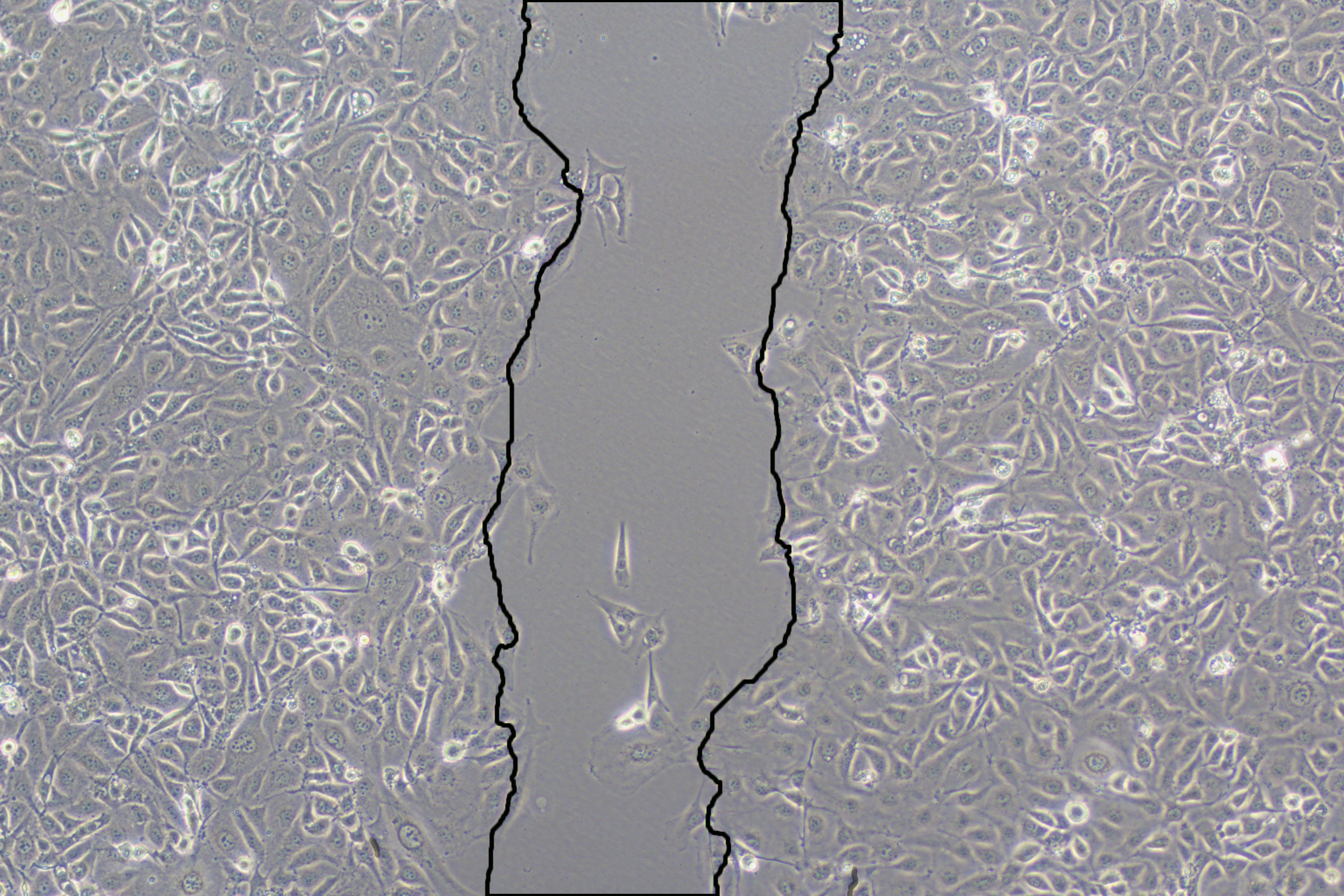

Supplement: S6 File — (ZIP) [file pone.0324264.s006.zip › supplement.material-6/images(Cell Scratch Assay)- HUVEC-24H/24-Control2.jpg]

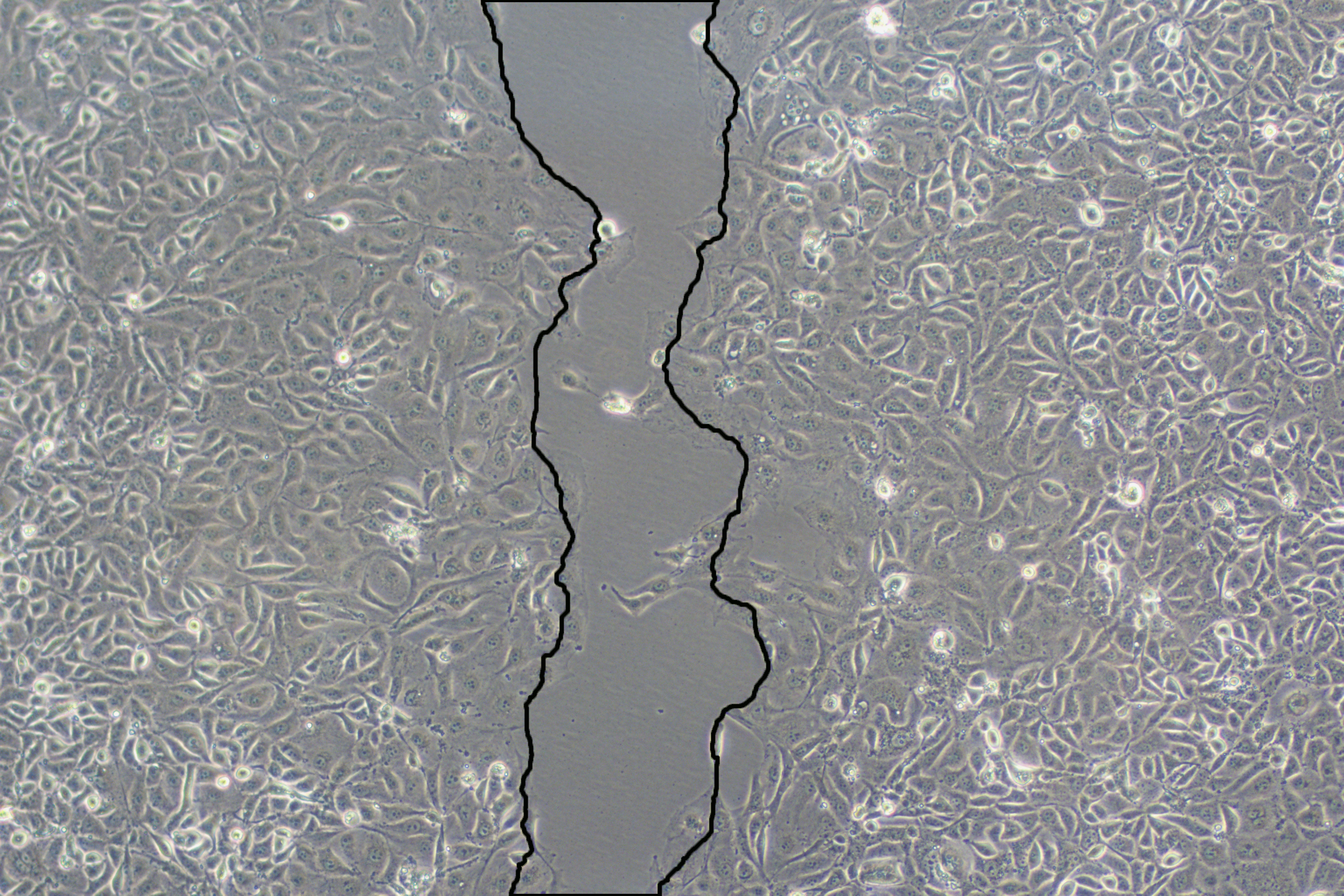

Supplement: S6 File — (ZIP) [file pone.0324264.s006.zip › supplement.material-6/images(Cell Scratch Assay)- HUVEC-24H/24-Control3.jpg]

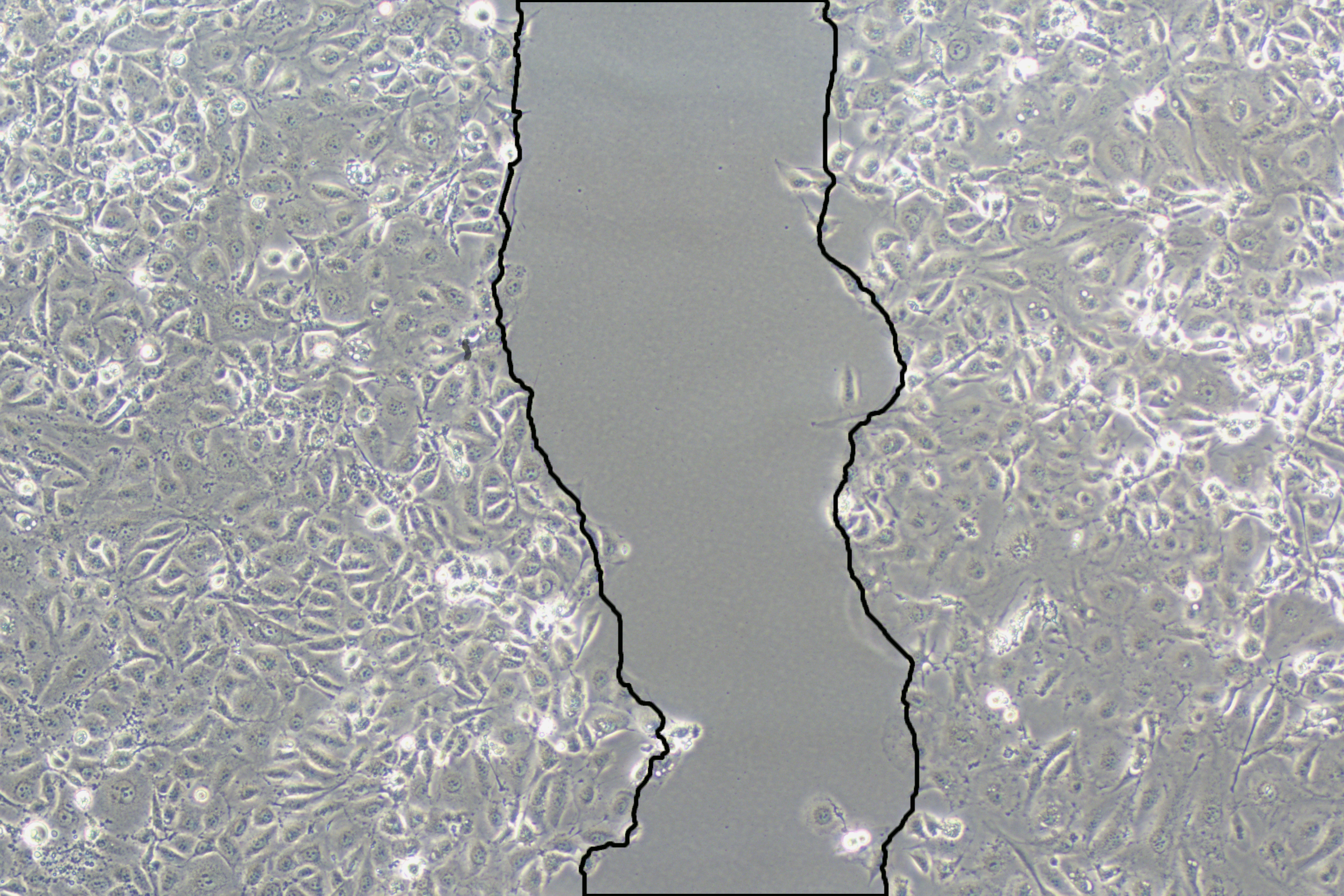

Supplement: S6 File — (ZIP) [file pone.0324264.s006.zip › supplement.material-6/images(Cell Scratch Assay)- HUVEC-24H/24-Control4.jpg]

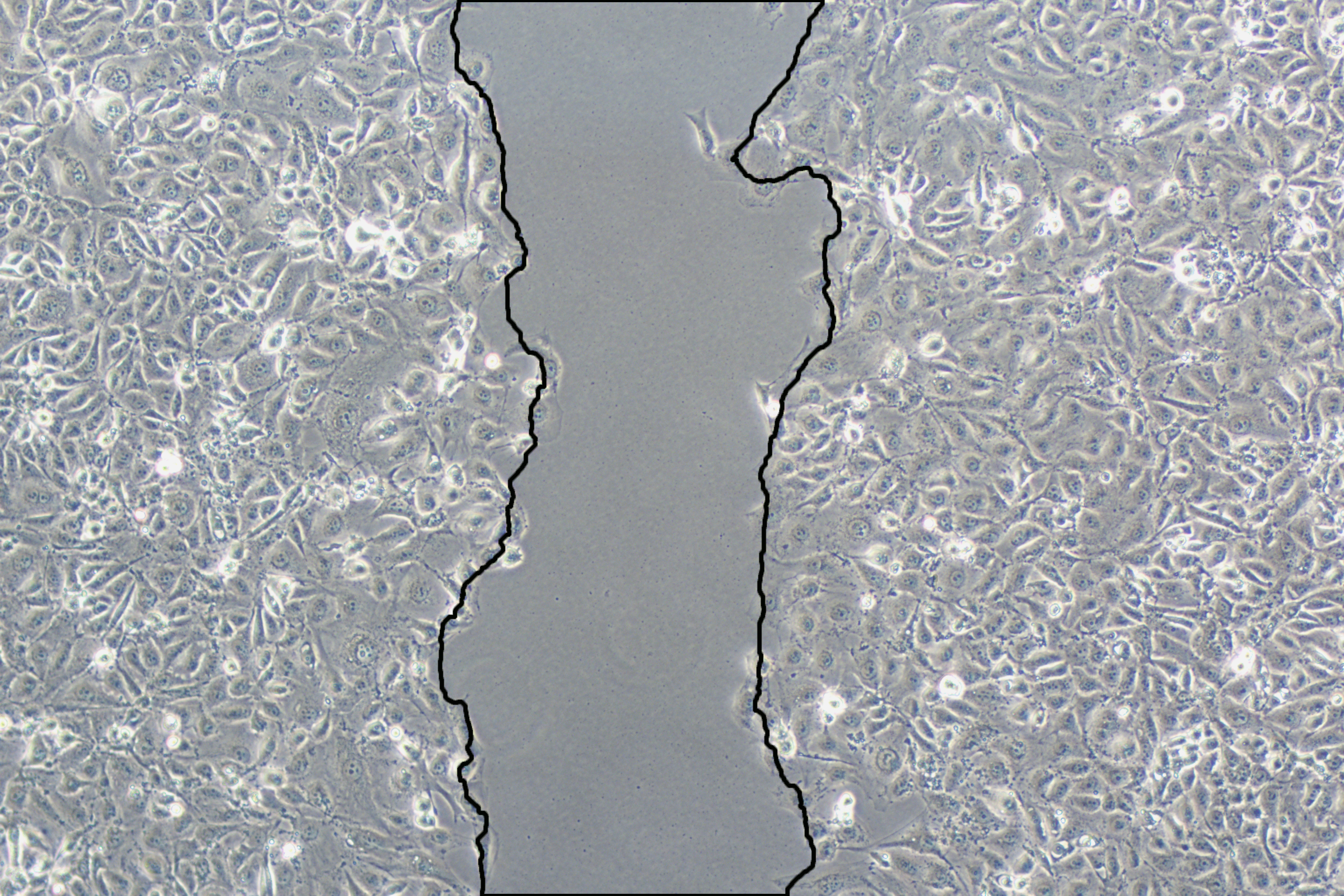

Supplement: S6 File — (ZIP) [file pone.0324264.s006.zip › supplement.material-6/images(Cell Scratch Assay)- HUVEC-24H/24-Control5.jpg]

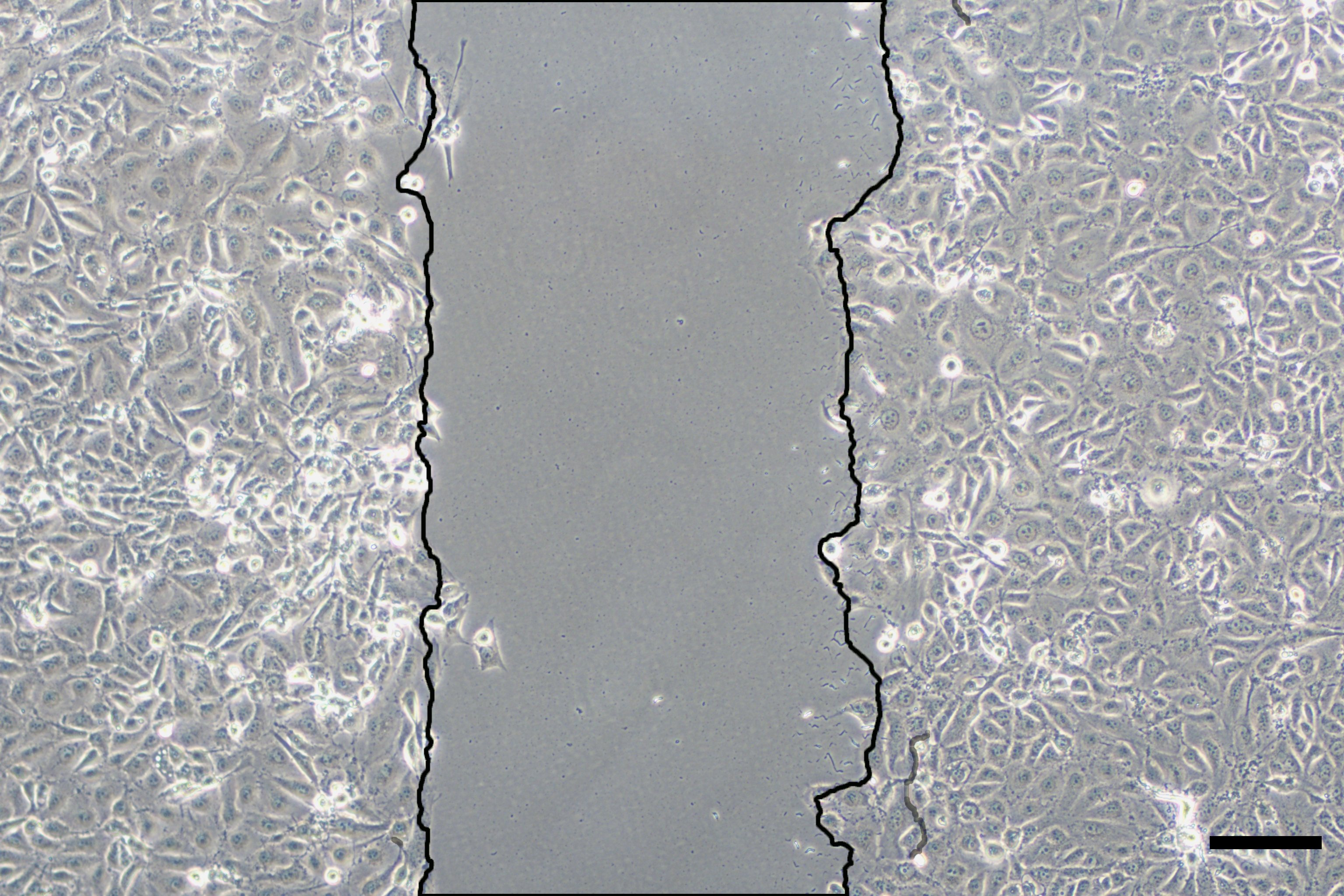

Supplement: S6 File — (ZIP) [file pone.0324264.s006.zip › supplement.material-6/images(Cell Scratch Assay)- HUVEC-24H/24-Model1-.jpg]

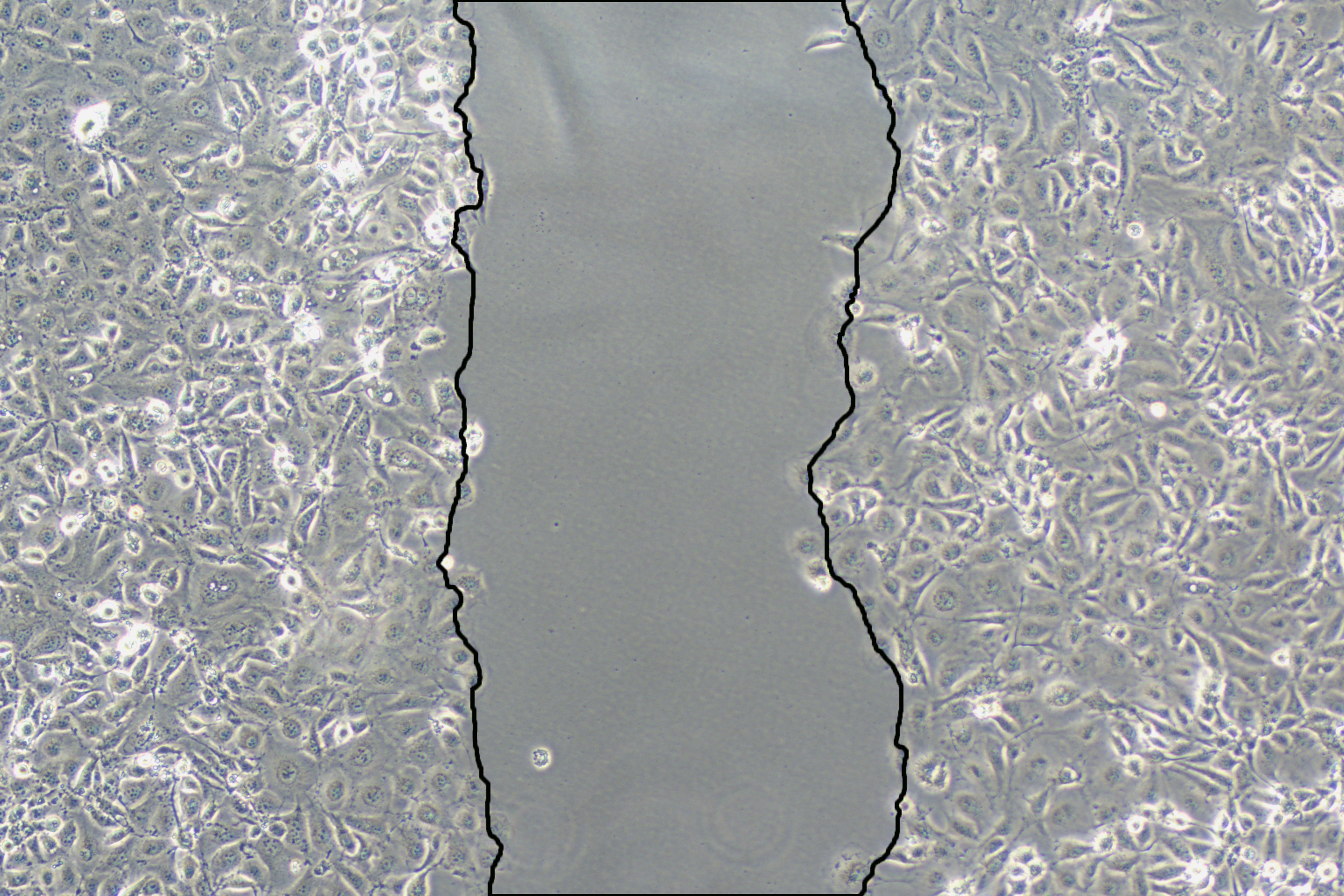

Supplement: S6 File — (ZIP) [file pone.0324264.s006.zip › supplement.material-6/images(Cell Scratch Assay)- HUVEC-24H/24-Model2.jpg]

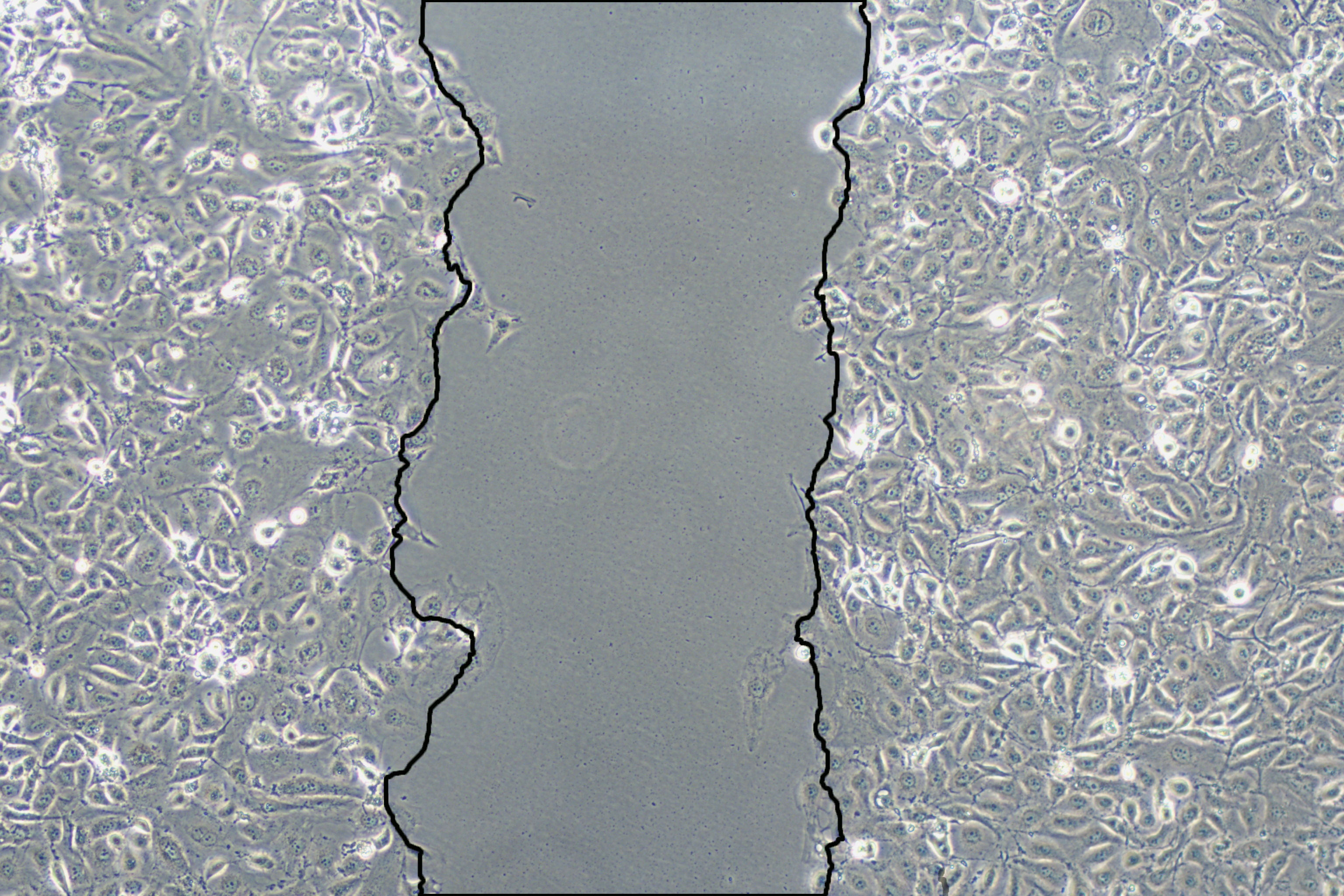

Supplement: S6 File — (ZIP) [file pone.0324264.s006.zip › supplement.material-6/images(Cell Scratch Assay)- HUVEC-24H/24-Model3.jpg]

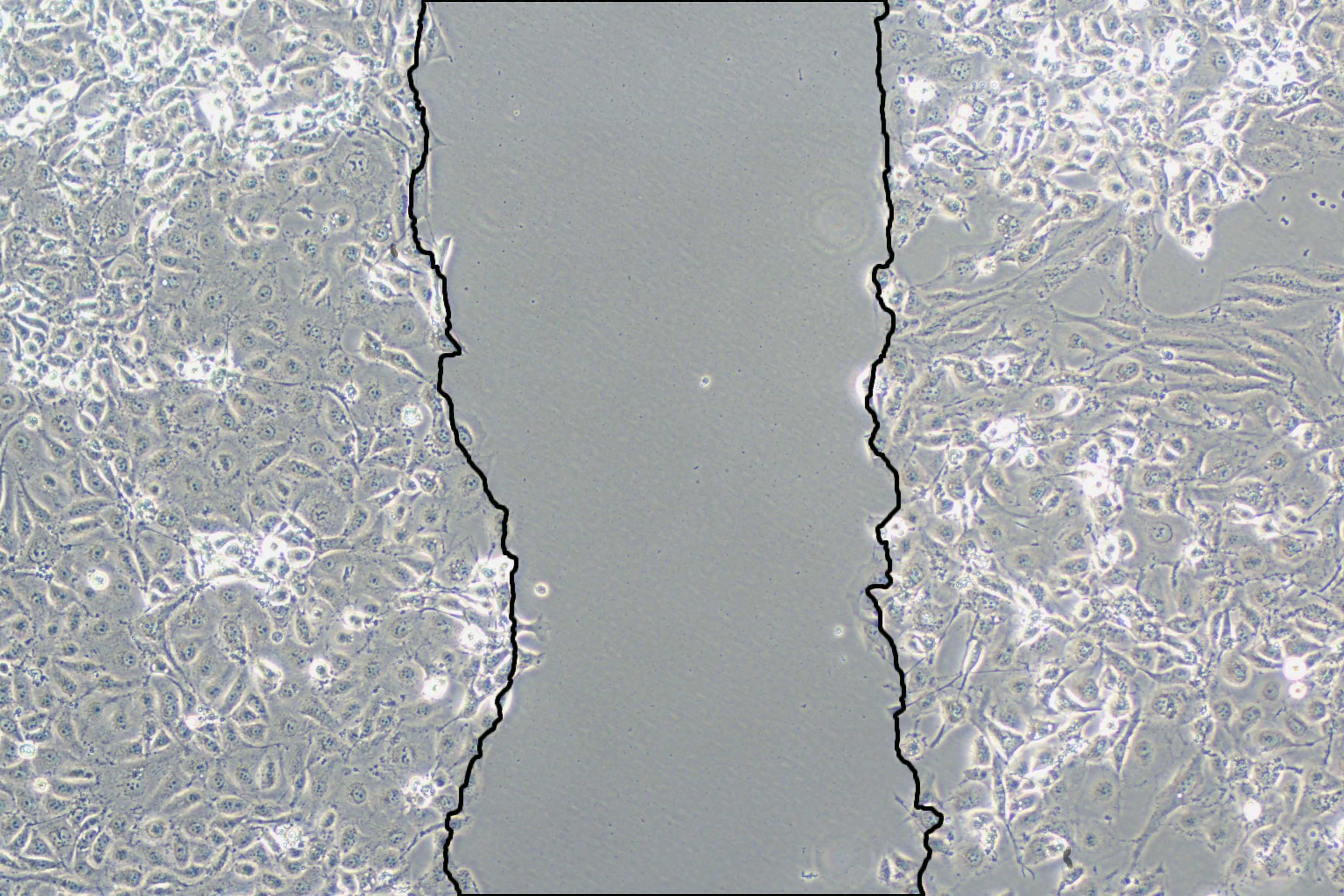

Supplement: S6 File — (ZIP) [file pone.0324264.s006.zip › supplement.material-6/images(Cell Scratch Assay)- HUVEC-24H/24-Model4.jpg]

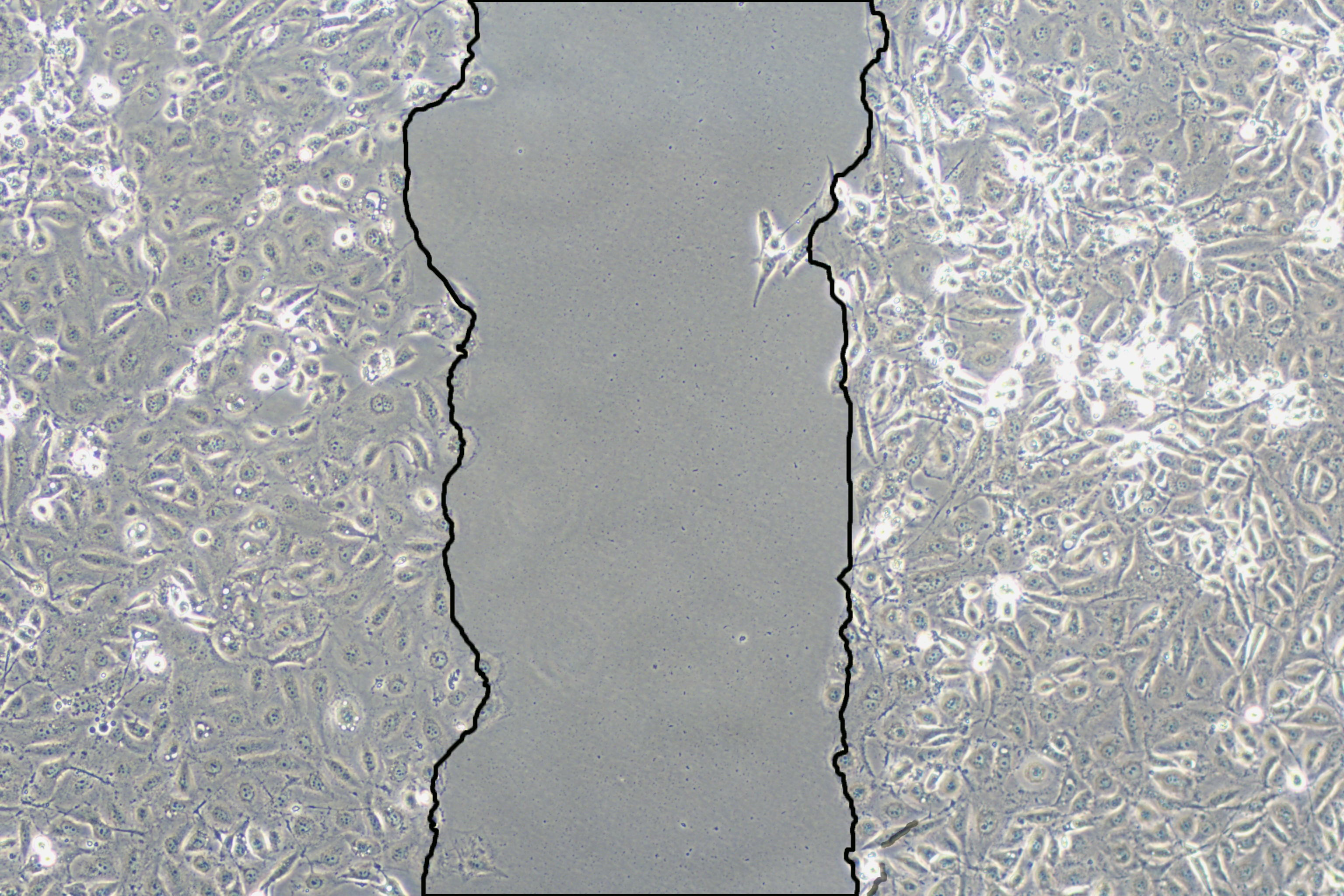

Supplement: S6 File — (ZIP) [file pone.0324264.s006.zip › supplement.material-6/images(Cell Scratch Assay)- HUVEC-24H/24-Model5.jpg]

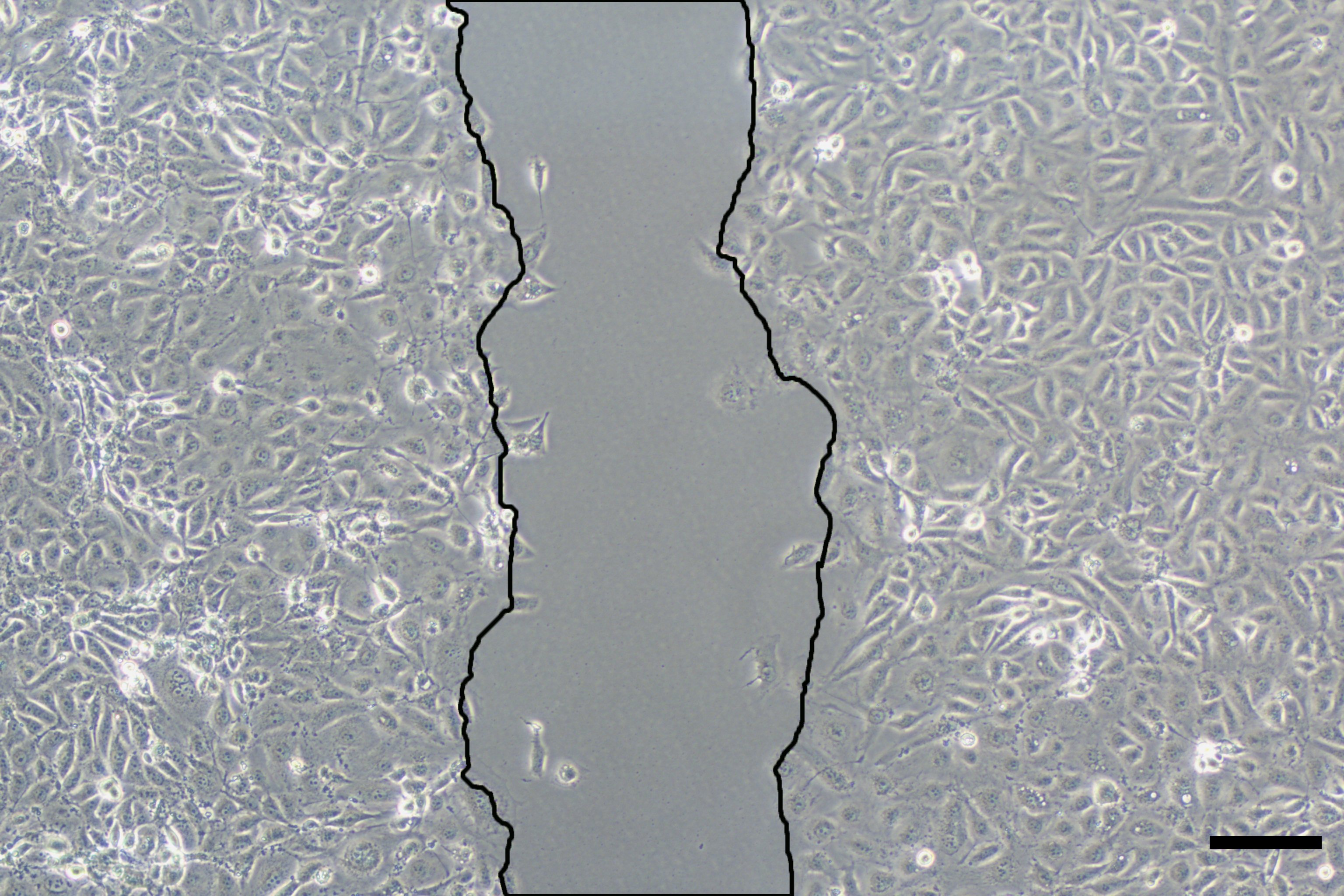

Supplement: S6 File — (ZIP) [file pone.0324264.s006.zip › supplement.material-6/images(Cell Scratch Assay)- HUVEC-24H/24-PL10X1-.jpg]

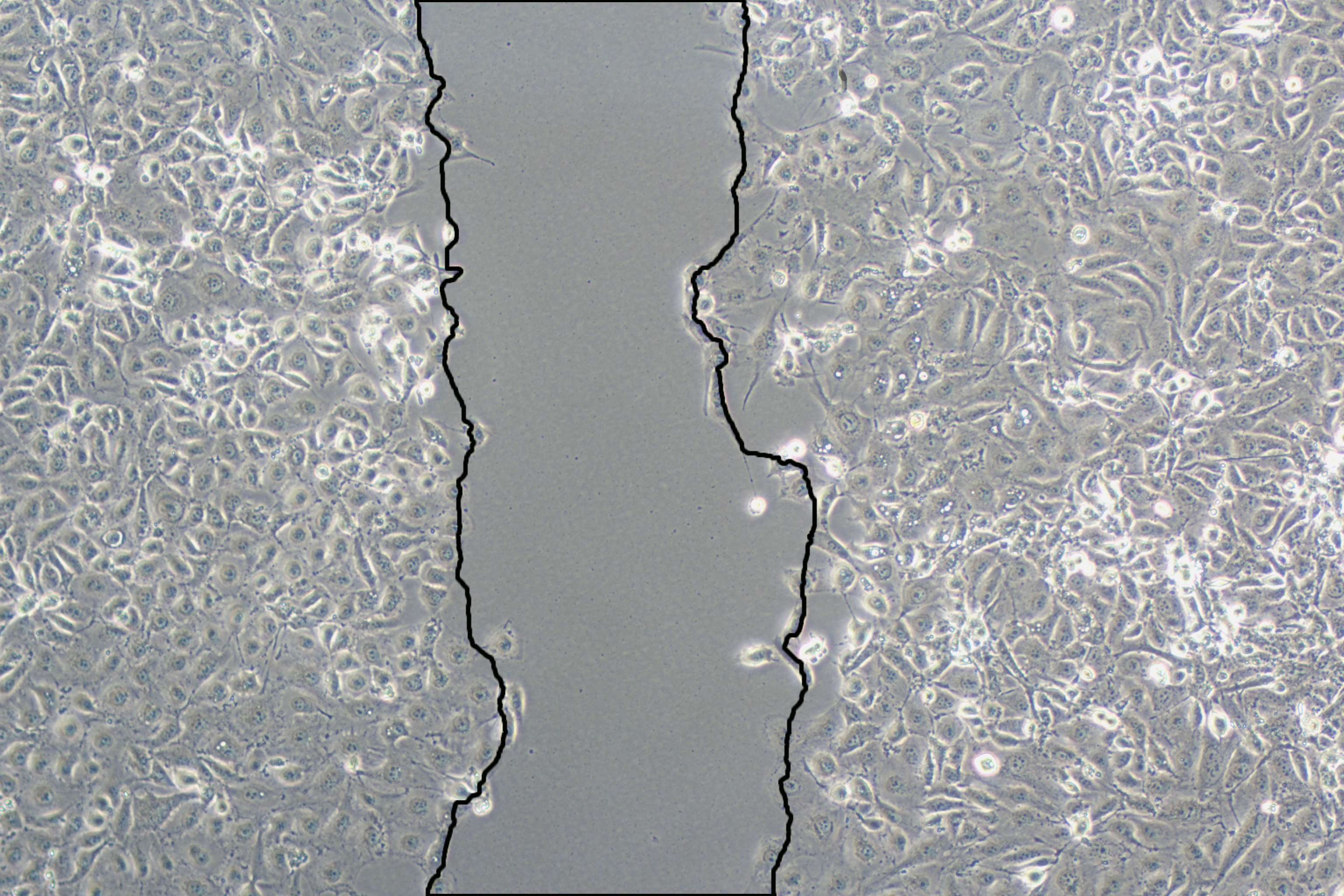

Supplement: S6 File — (ZIP) [file pone.0324264.s006.zip › supplement.material-6/images(Cell Scratch Assay)- HUVEC-24H/24-PL10X2.jpg]

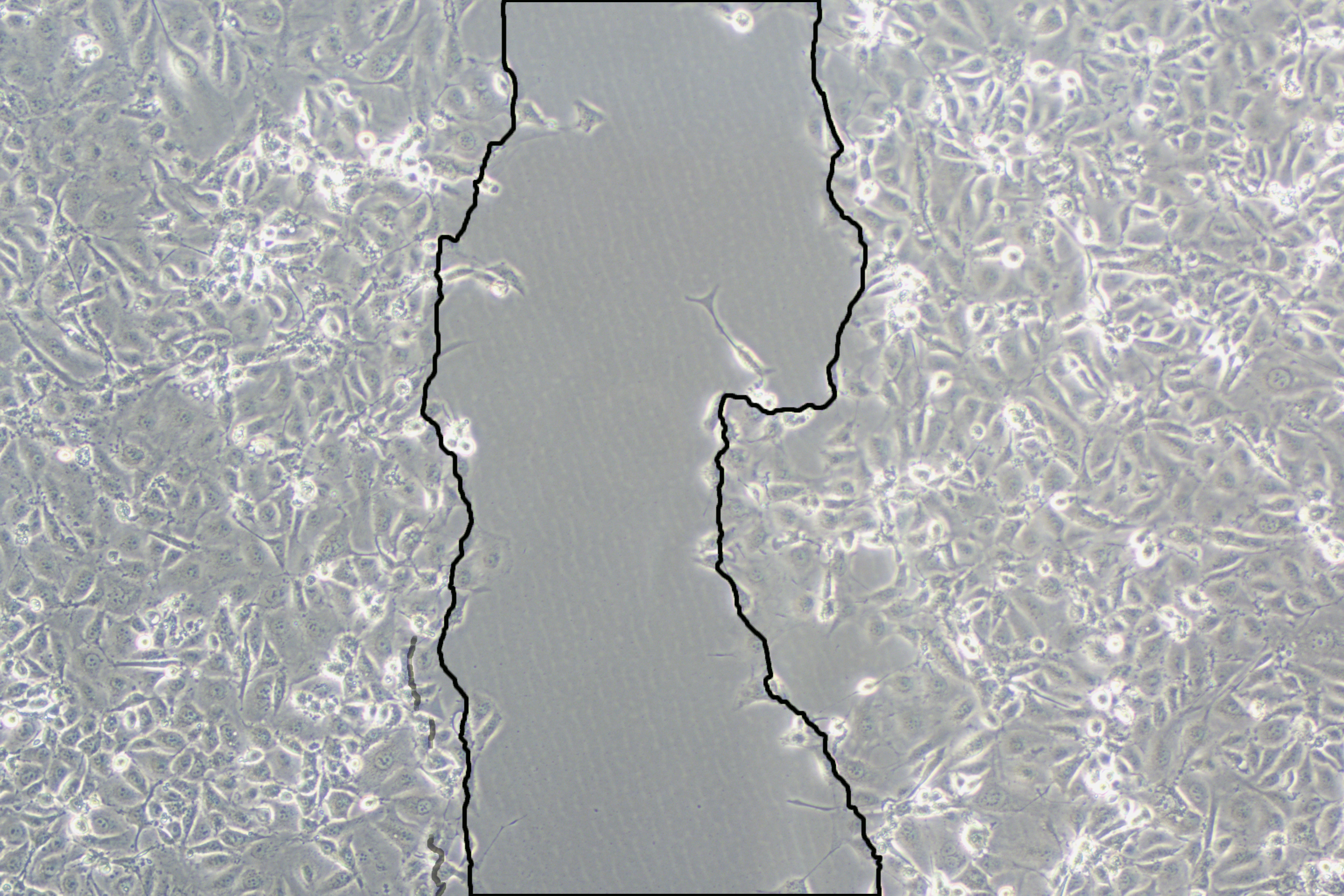

Supplement: S6 File — (ZIP) [file pone.0324264.s006.zip › supplement.material-6/images(Cell Scratch Assay)- HUVEC-24H/24-PL10X3.jpg]

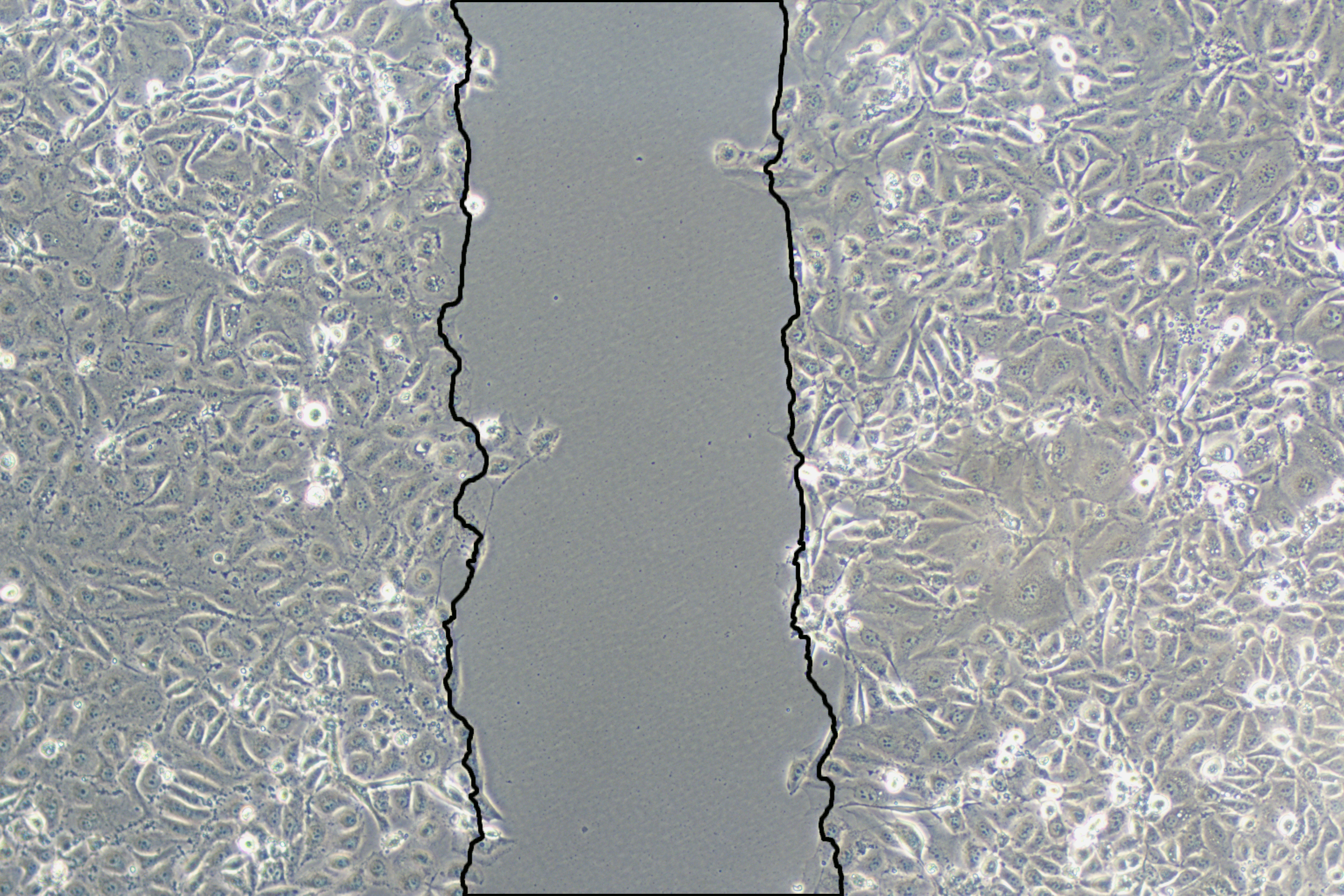

Supplement: S6 File — (ZIP) [file pone.0324264.s006.zip › supplement.material-6/images(Cell Scratch Assay)- HUVEC-24H/24-PL10X4.jpg]

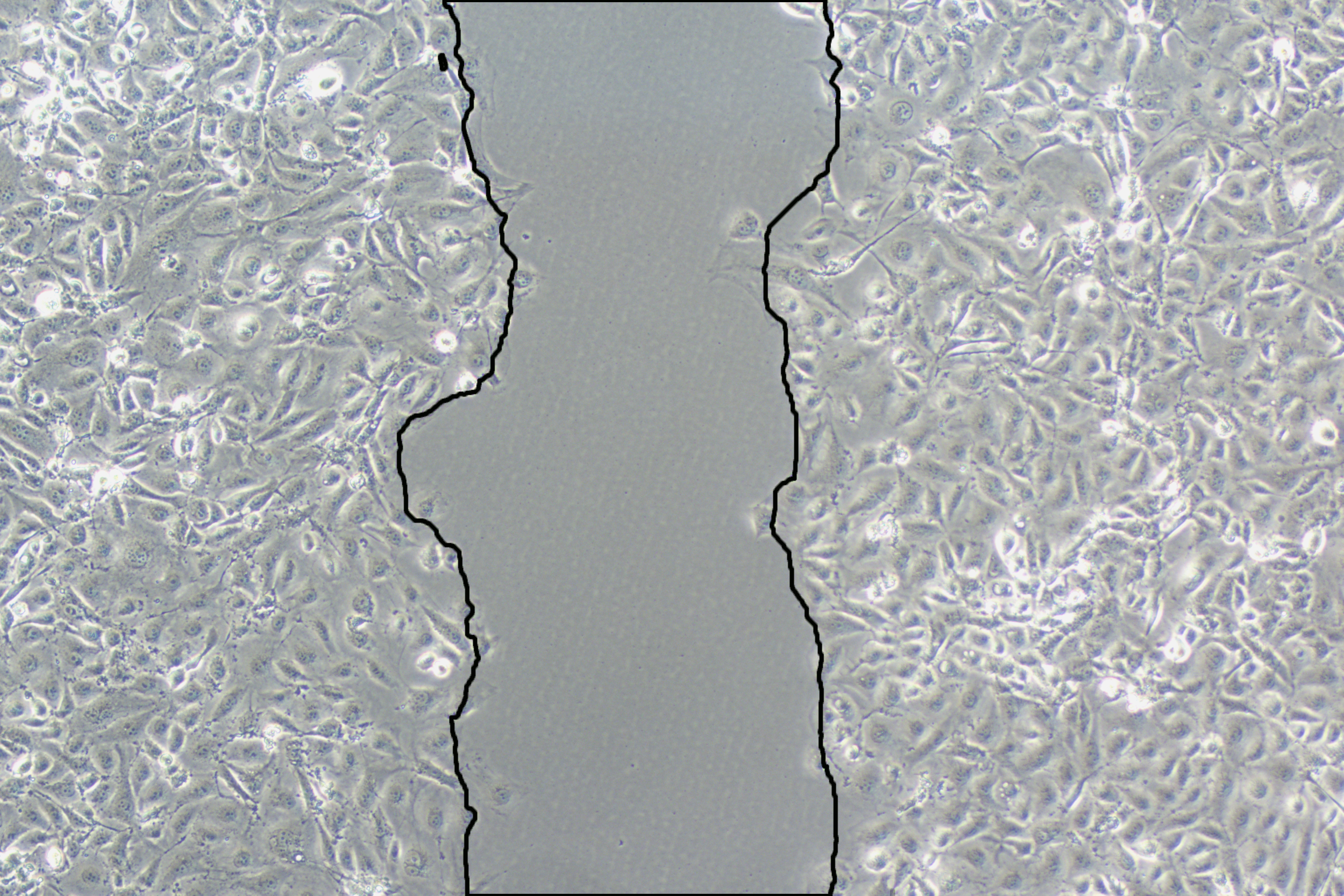

Supplement: S6 File — (ZIP) [file pone.0324264.s006.zip › supplement.material-6/images(Cell Scratch Assay)- HUVEC-24H/24-PL10X5.jpg]

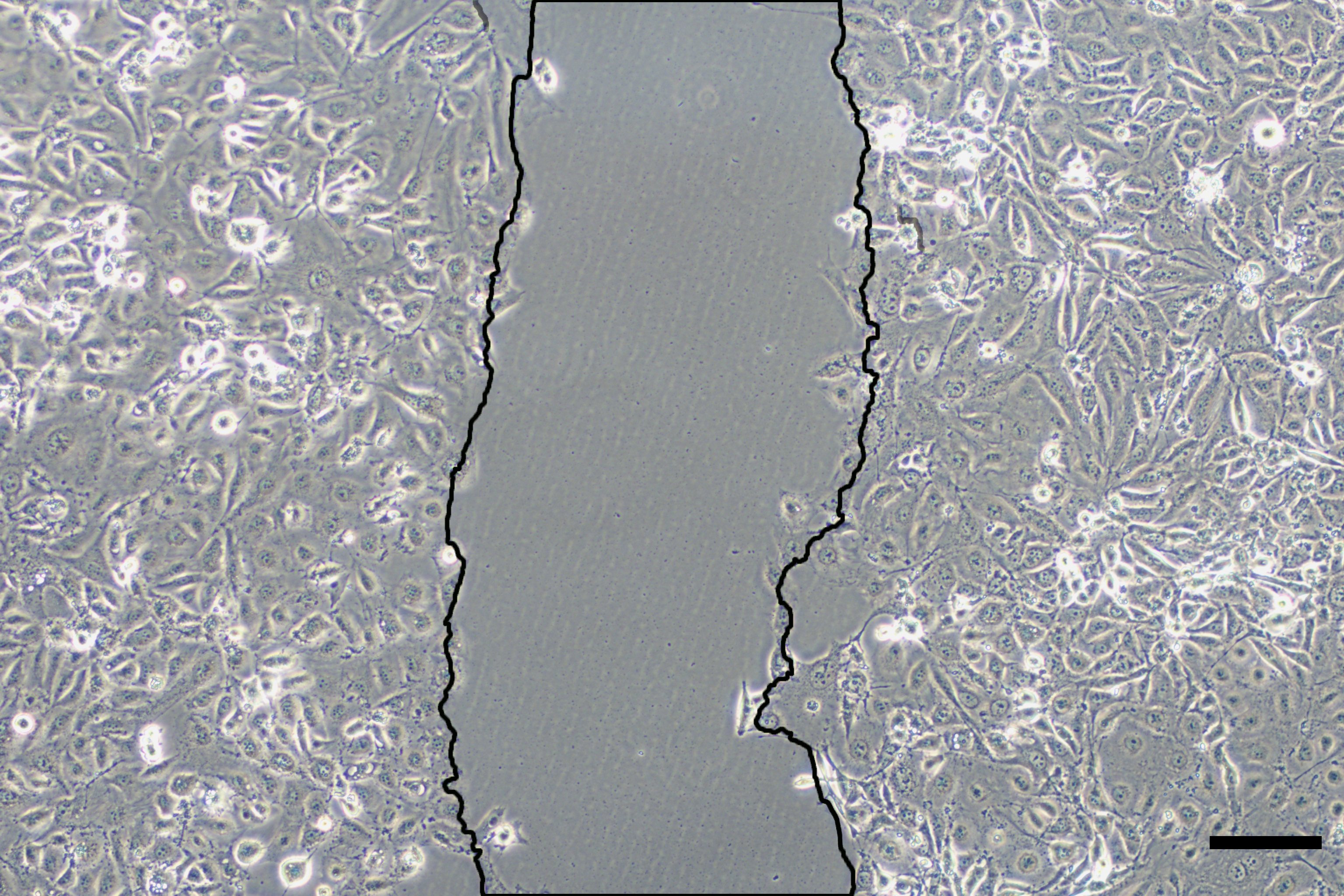

Supplement: S6 File — (ZIP) [file pone.0324264.s006.zip › supplement.material-6/images(Cell Scratch Assay)- HUVEC-24H/24-PL20X1-.jpg]

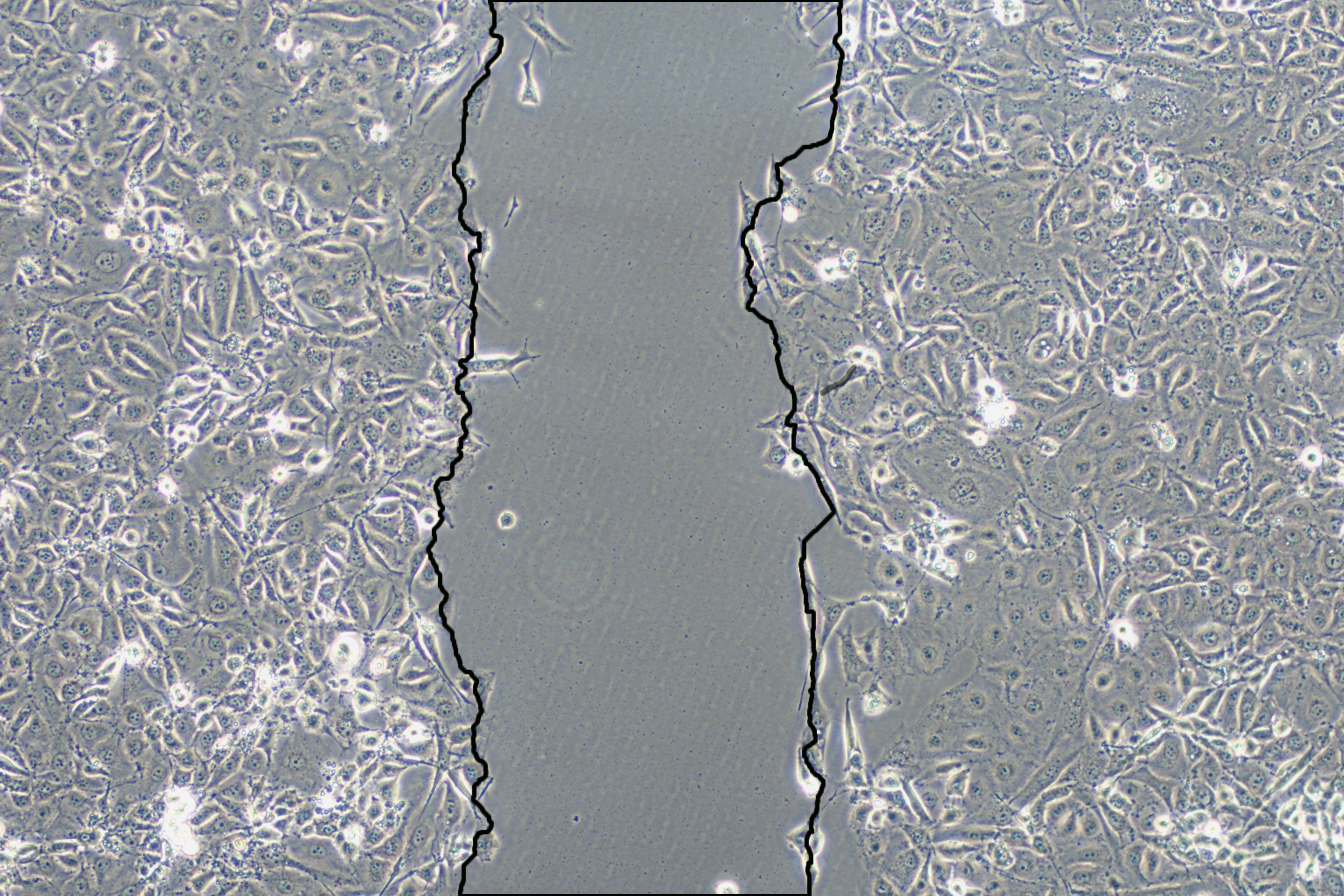

Supplement: S6 File — (ZIP) [file pone.0324264.s006.zip › supplement.material-6/images(Cell Scratch Assay)- HUVEC-24H/24-PL20X2.jpg]

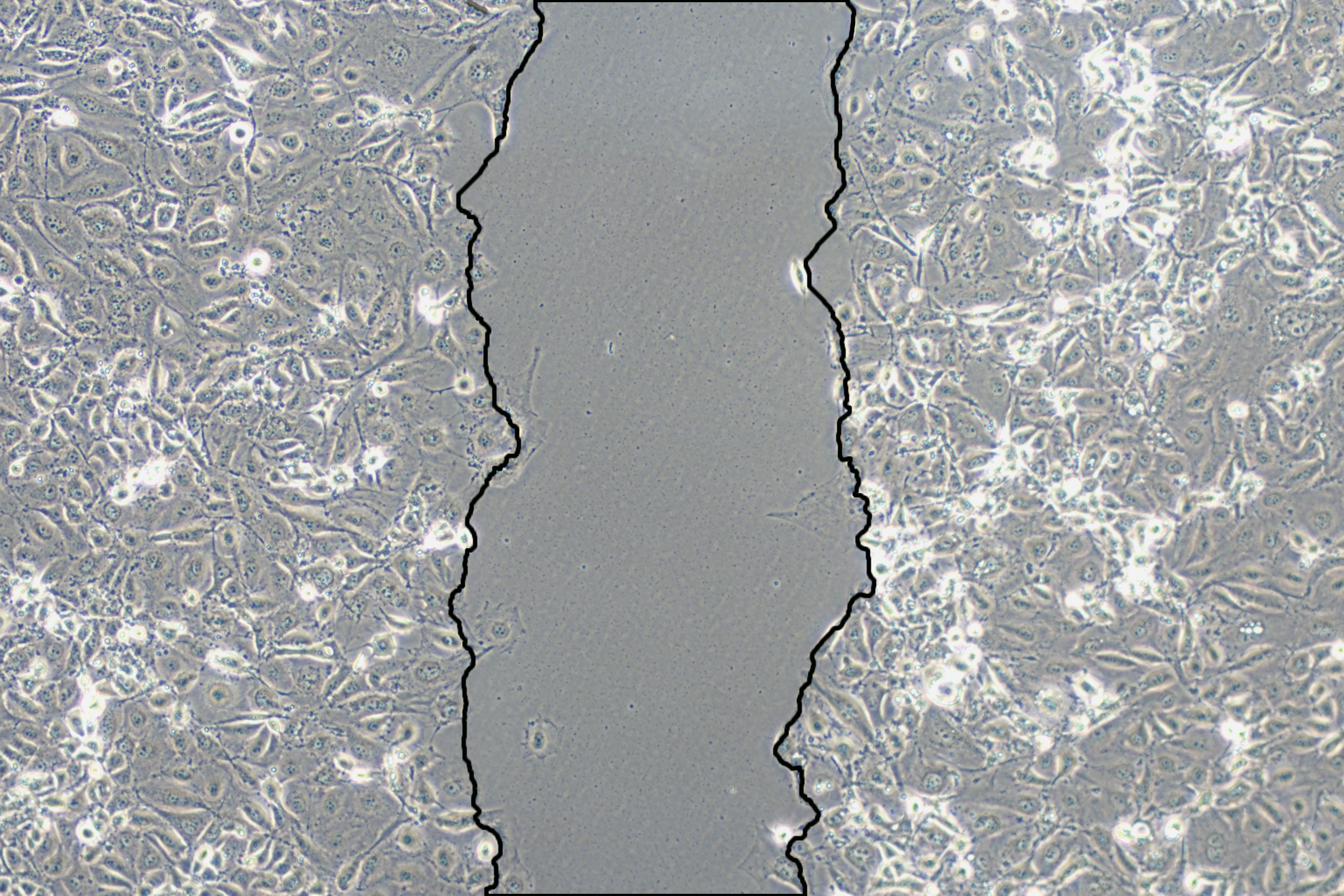

Supplement: S6 File — (ZIP) [file pone.0324264.s006.zip › supplement.material-6/images(Cell Scratch Assay)- HUVEC-24H/24-PL20X3.jpg]

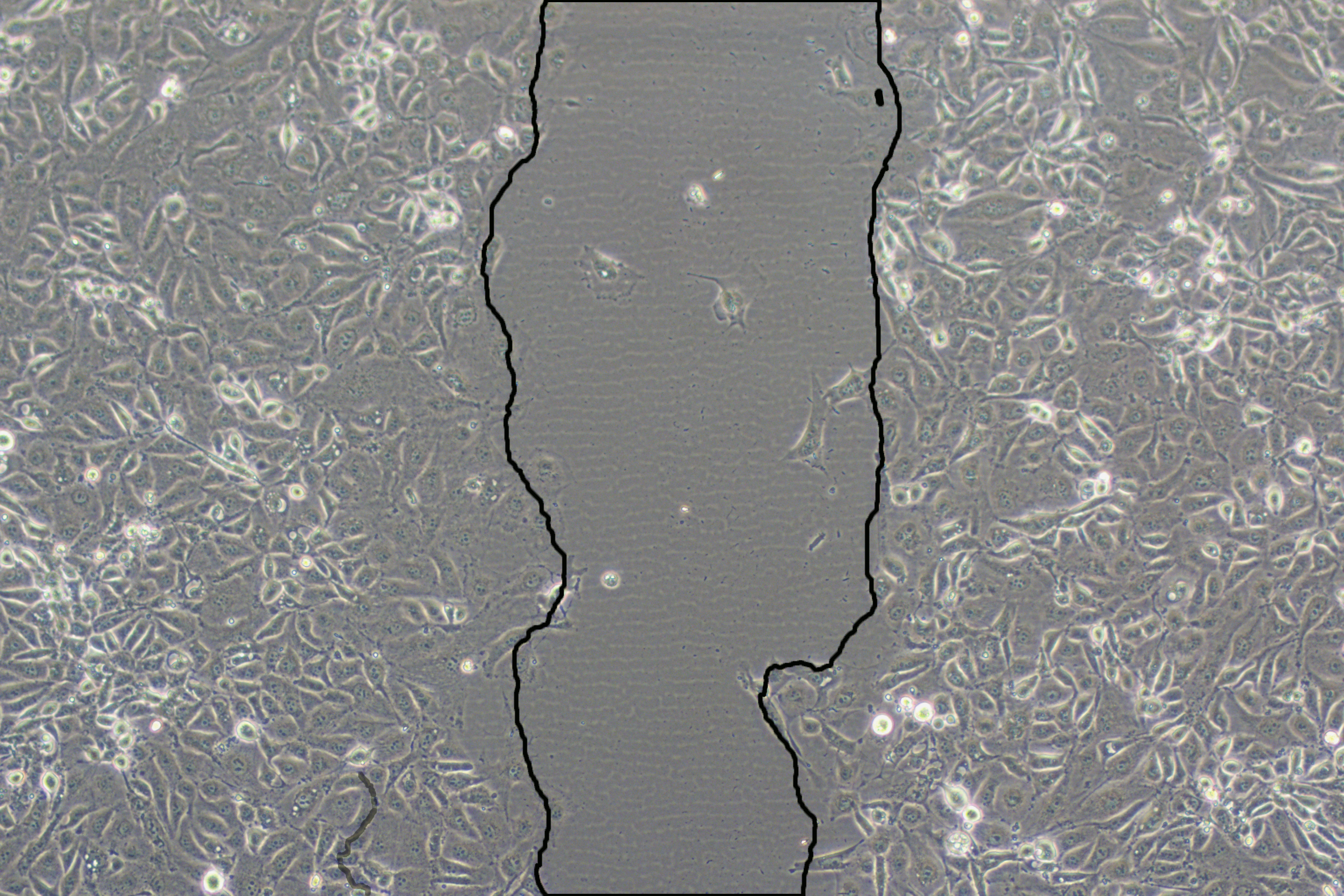

Supplement: S6 File — (ZIP) [file pone.0324264.s006.zip › supplement.material-6/images(Cell Scratch Assay)- HUVEC-24H/24-PL20X4.jpg]

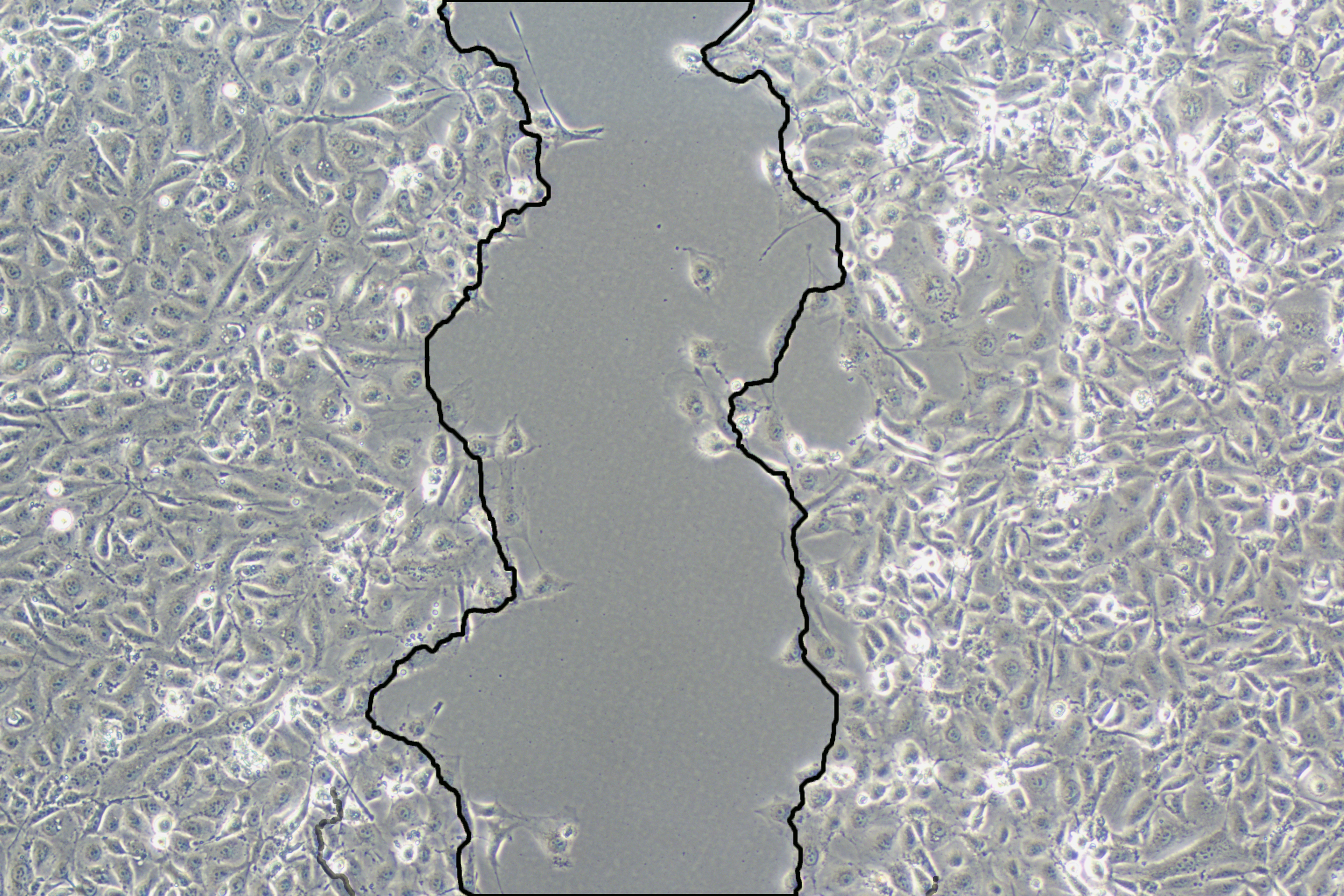

Supplement: S6 File — (ZIP) [file pone.0324264.s006.zip › supplement.material-6/images(Cell Scratch Assay)- HUVEC-24H/24-PL20X5.jpg]

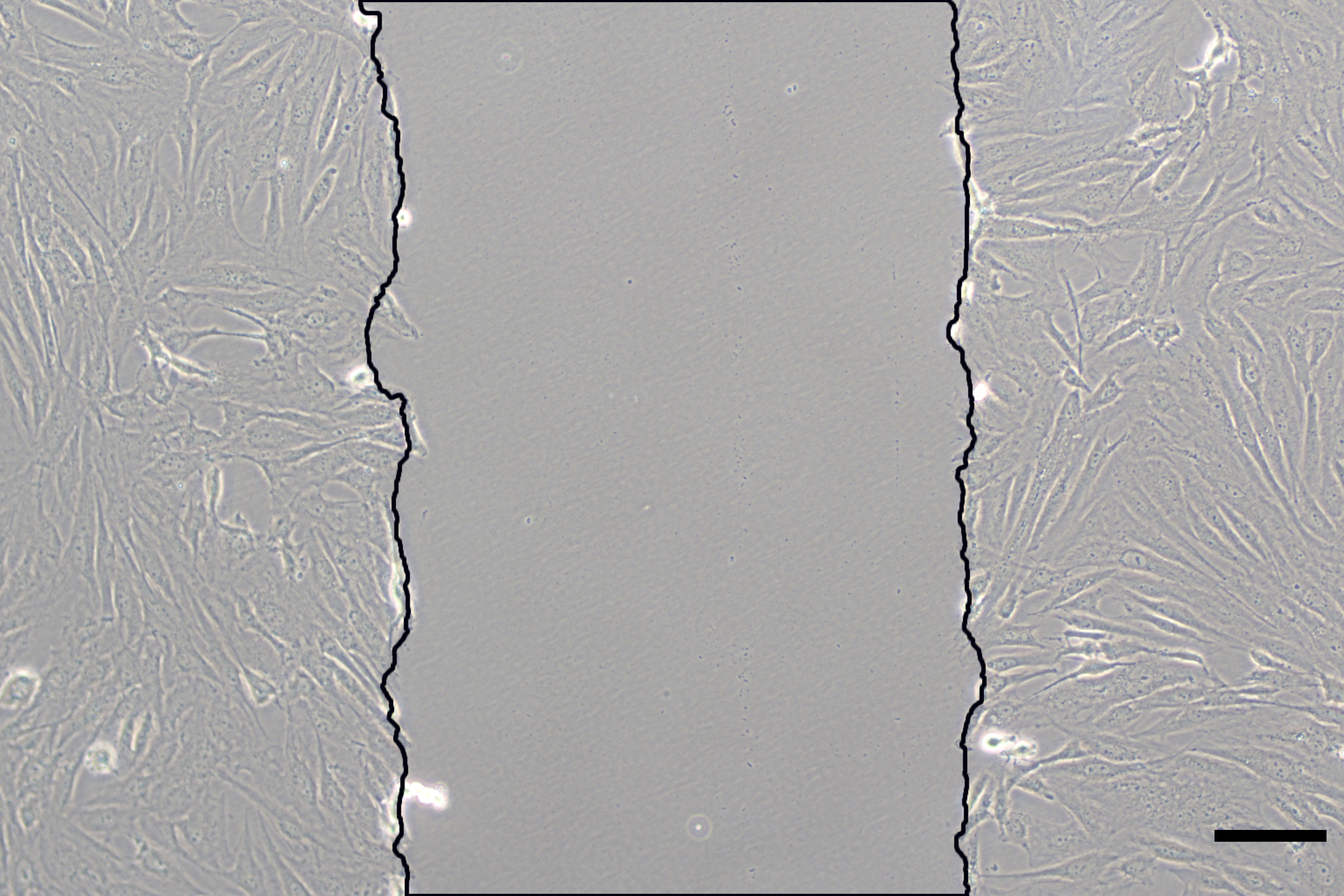

Supplement: S7 File — (ZIP) [file pone.0324264.s007.zip › supplement.material-7/images(Cell Scratch Assay)-HSF-0h/Control1.png]

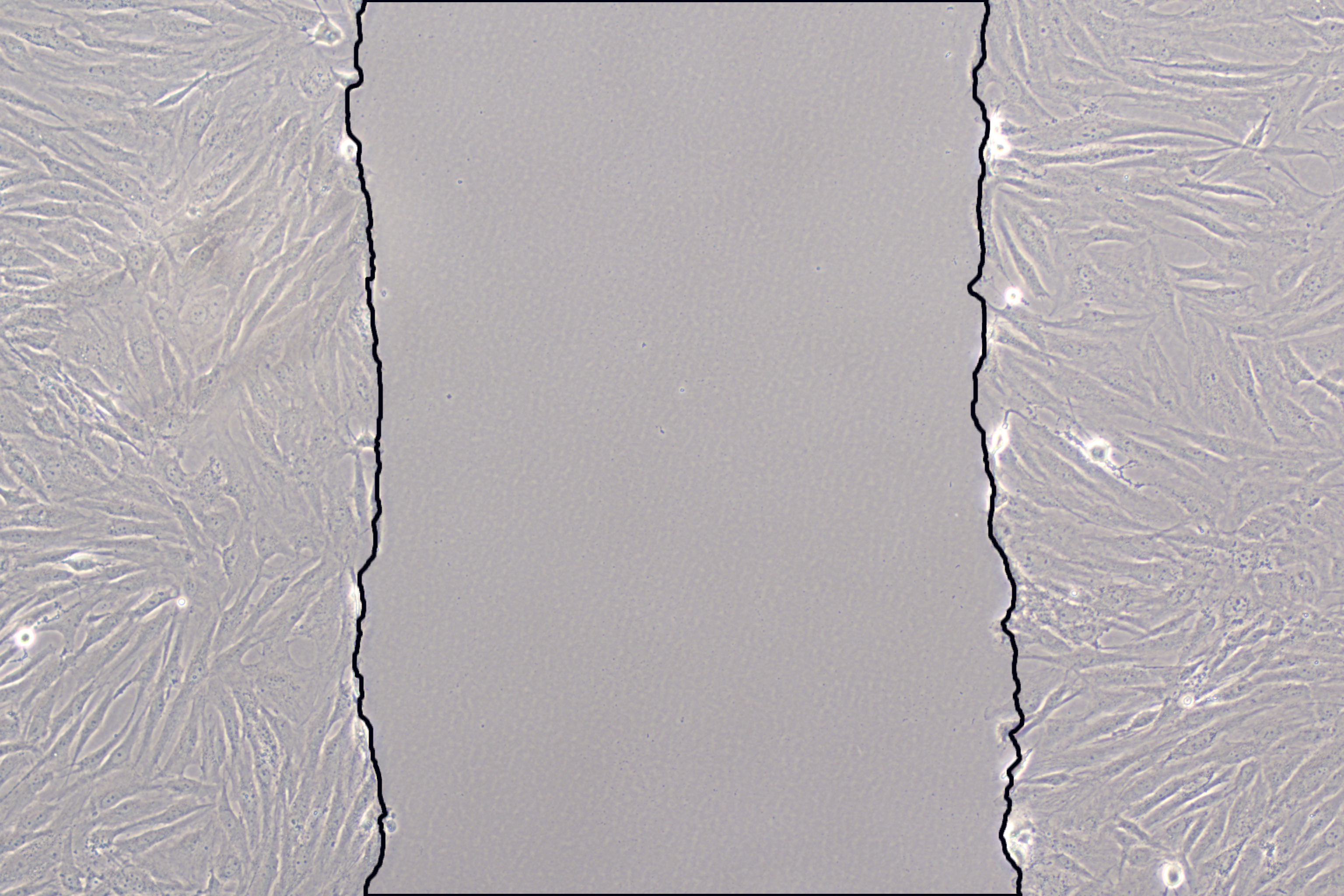

Supplement: S7 File — (ZIP) [file pone.0324264.s007.zip › supplement.material-7/images(Cell Scratch Assay)-HSF-0h/Control2.jpg]

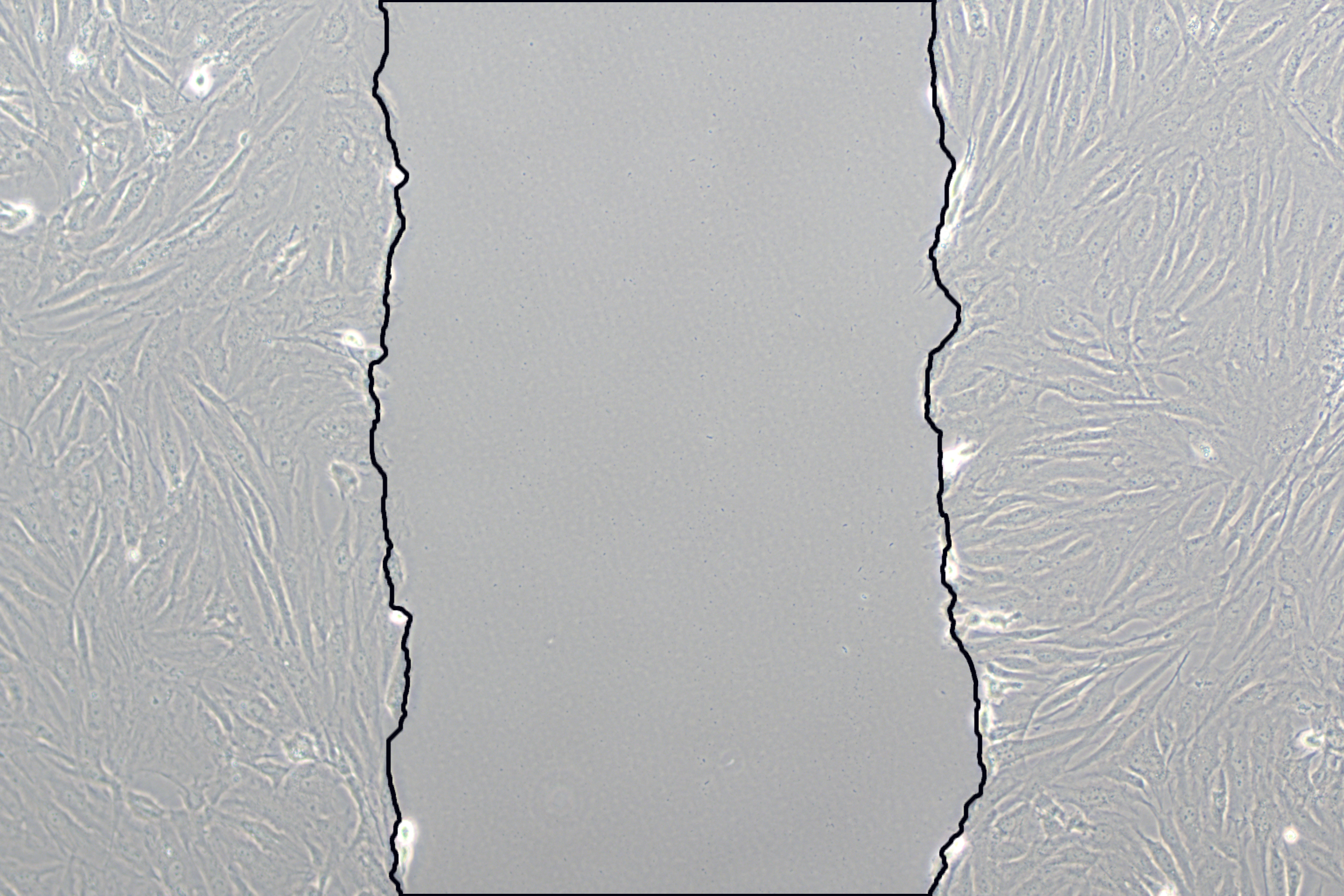

Supplement: S7 File — (ZIP) [file pone.0324264.s007.zip › supplement.material-7/images(Cell Scratch Assay)-HSF-0h/Control3.jpg]

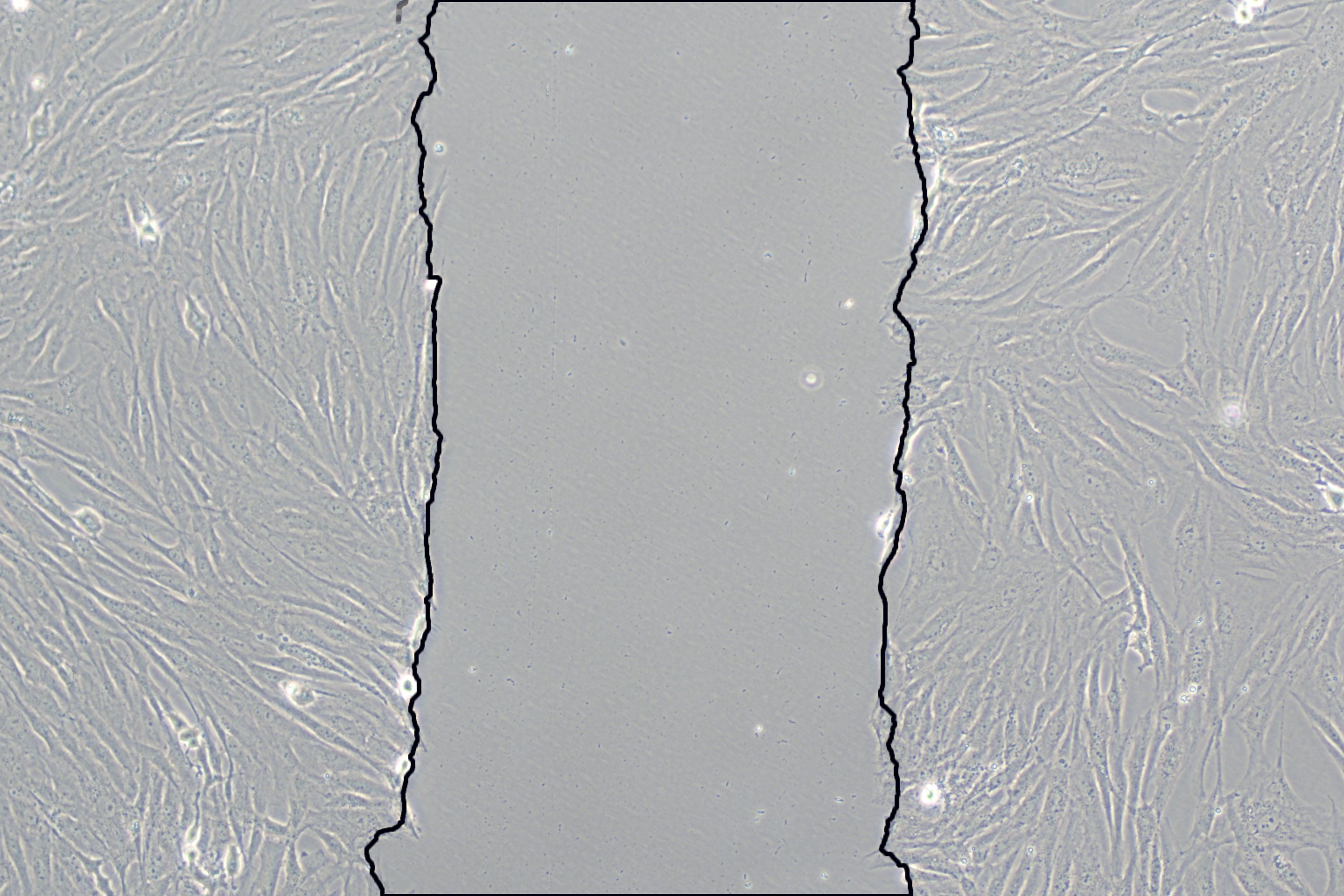

Supplement: S7 File — (ZIP) [file pone.0324264.s007.zip › supplement.material-7/images(Cell Scratch Assay)-HSF-0h/Control4.jpg]

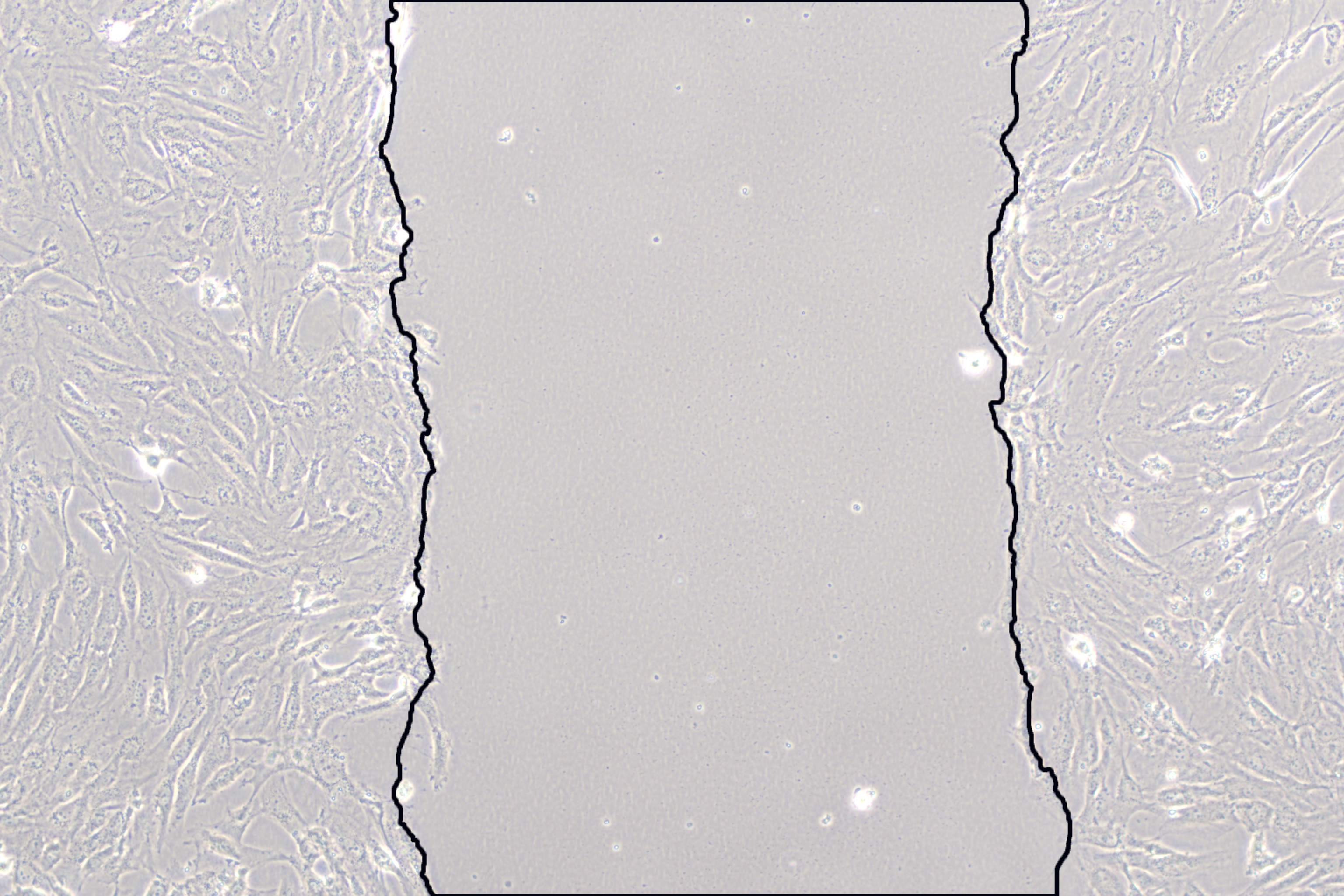

Supplement: S7 File — (ZIP) [file pone.0324264.s007.zip › supplement.material-7/images(Cell Scratch Assay)-HSF-0h/Control5.jpg]

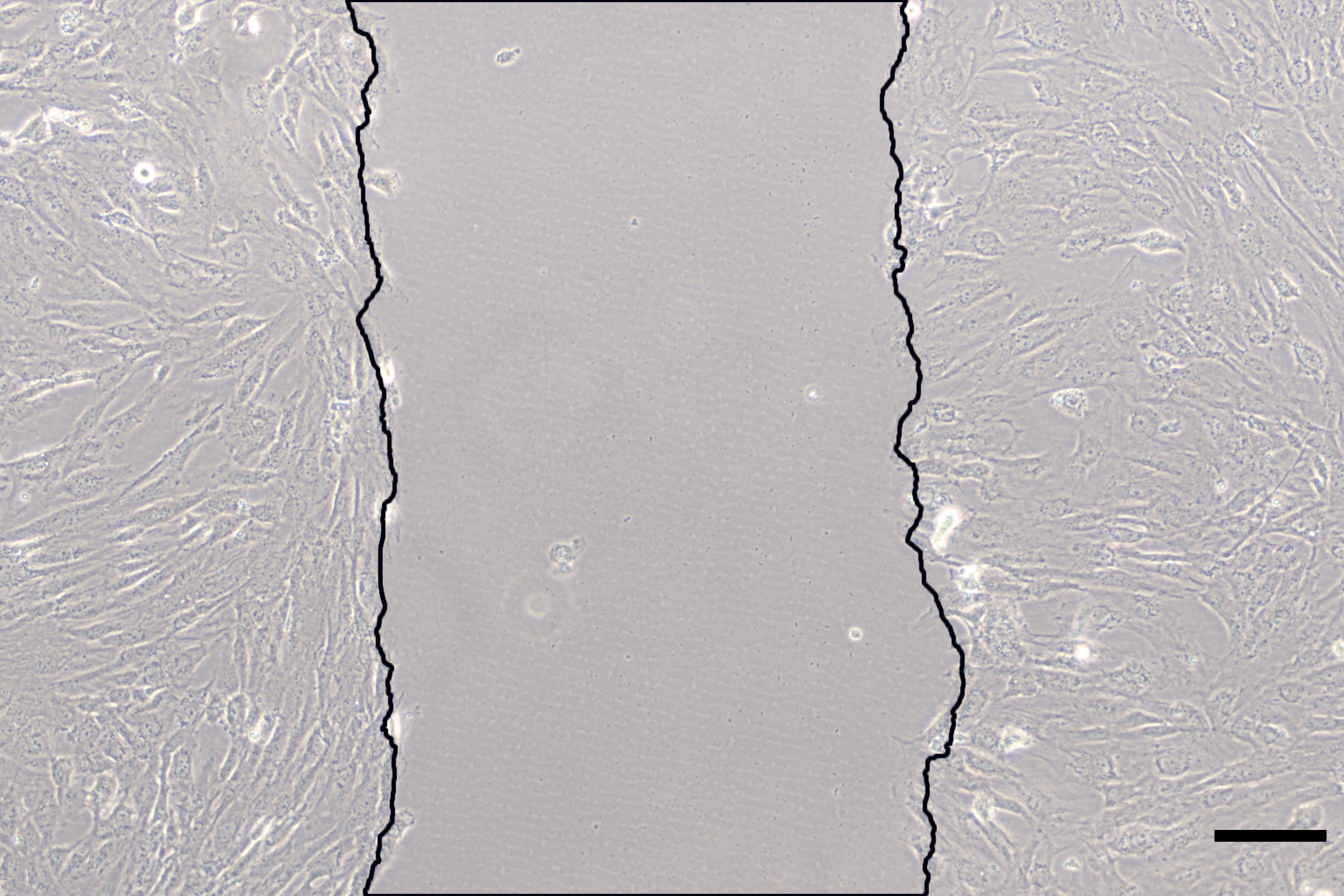

Supplement: S7 File — (ZIP) [file pone.0324264.s007.zip › supplement.material-7/images(Cell Scratch Assay)-HSF-0h/Model1.png]

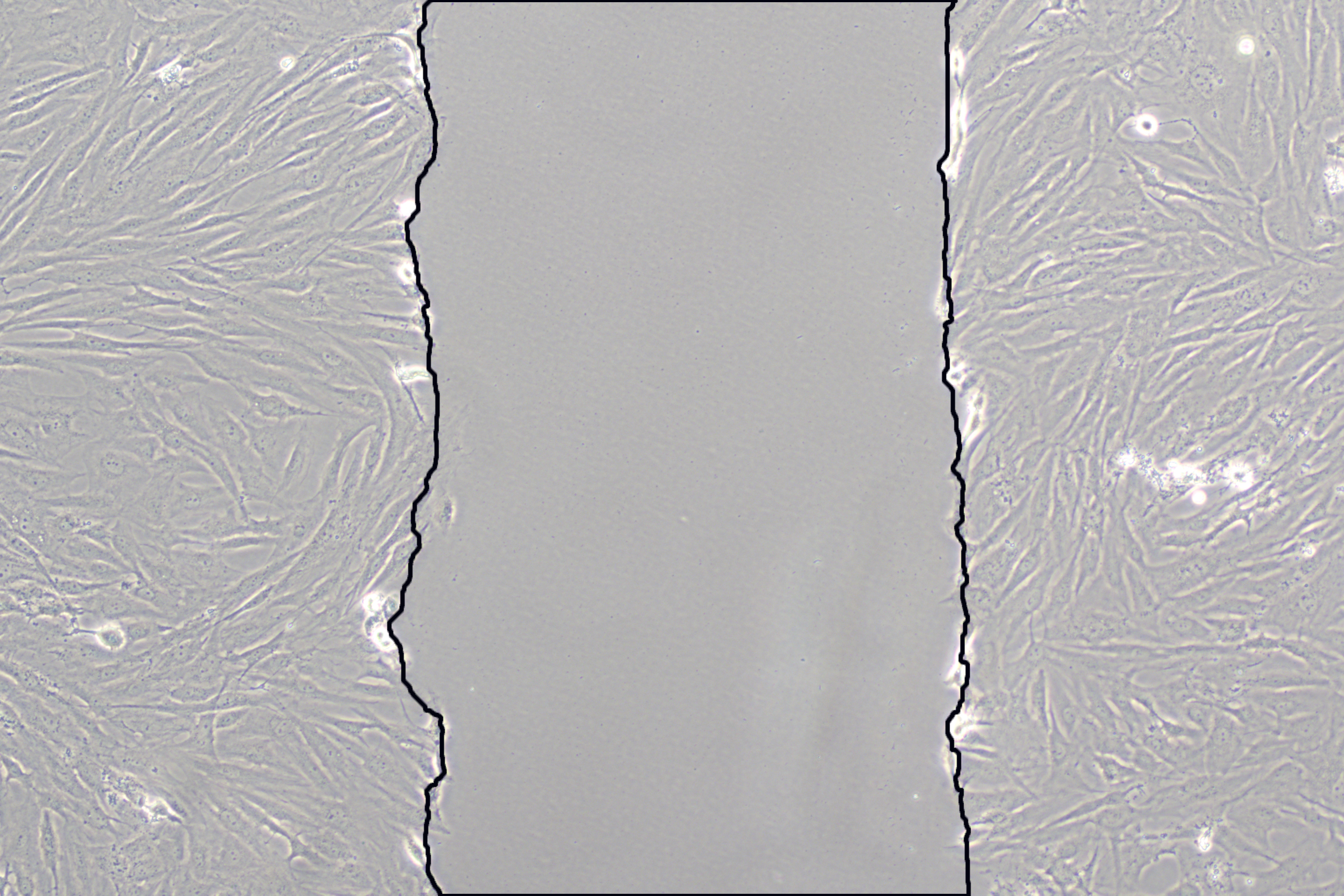

Supplement: S7 File — (ZIP) [file pone.0324264.s007.zip › supplement.material-7/images(Cell Scratch Assay)-HSF-0h/Model2.jpg]

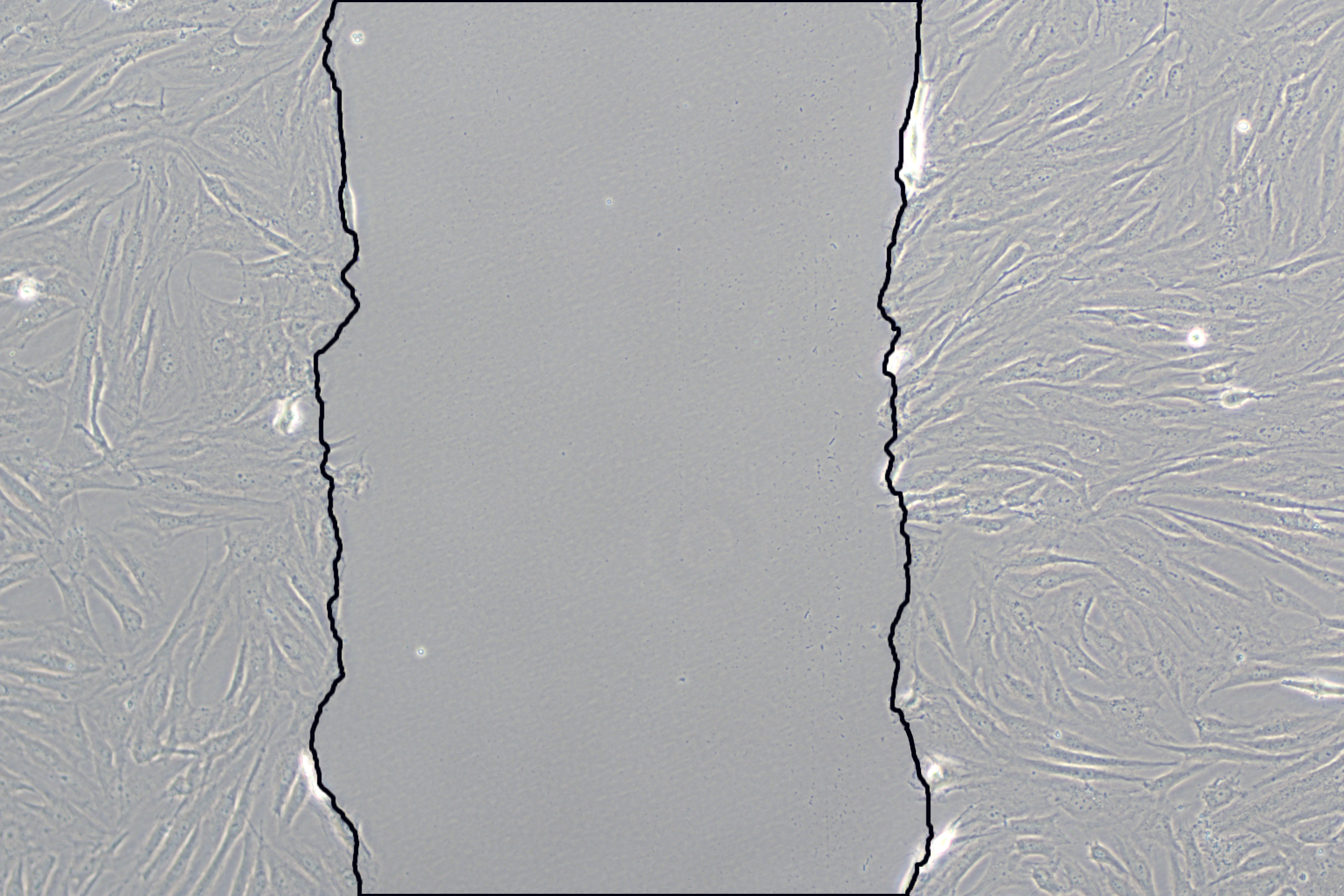

Supplement: S7 File — (ZIP) [file pone.0324264.s007.zip › supplement.material-7/images(Cell Scratch Assay)-HSF-0h/Model3.jpg]

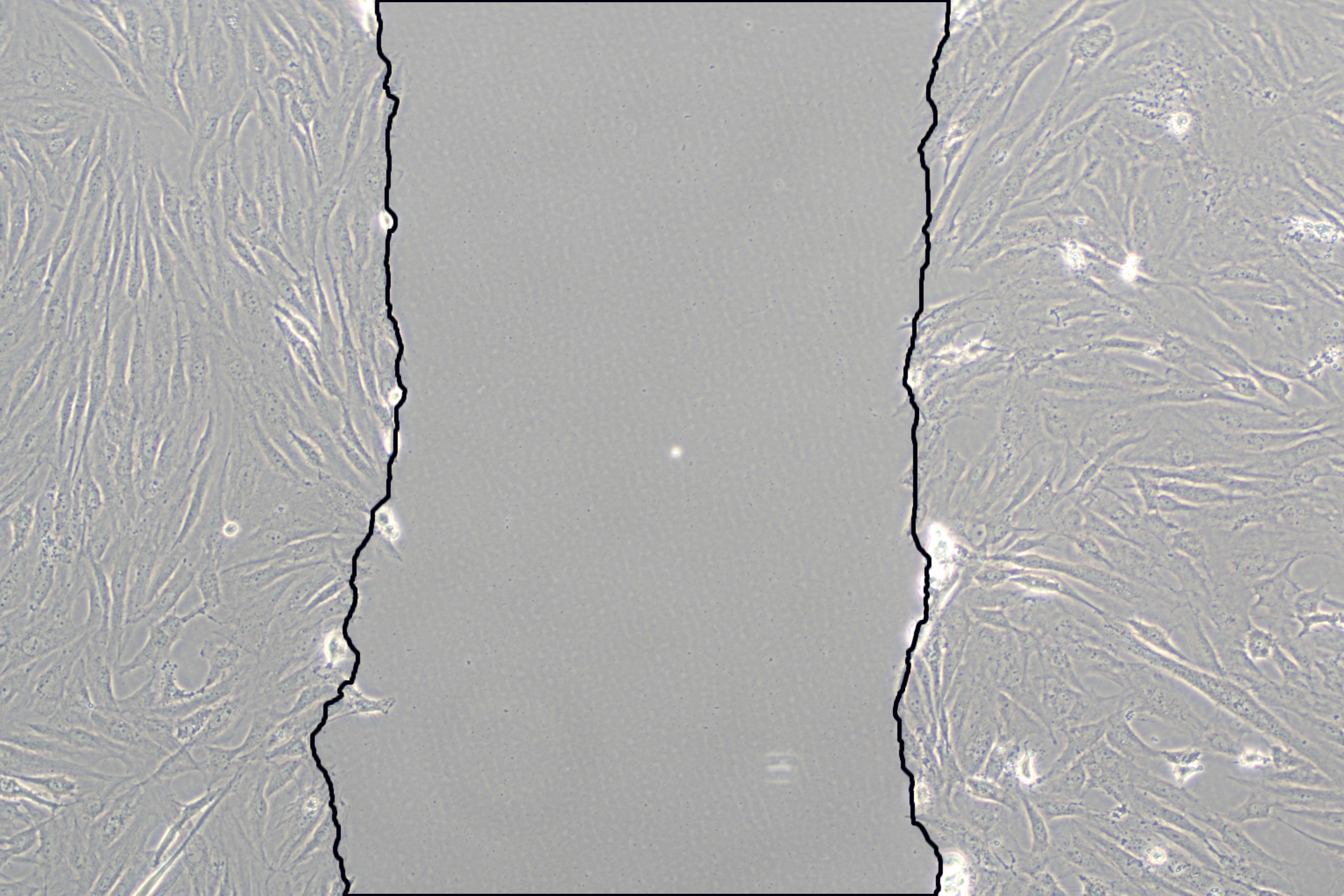

Supplement: S7 File — (ZIP) [file pone.0324264.s007.zip › supplement.material-7/images(Cell Scratch Assay)-HSF-0h/Model4.jpg]

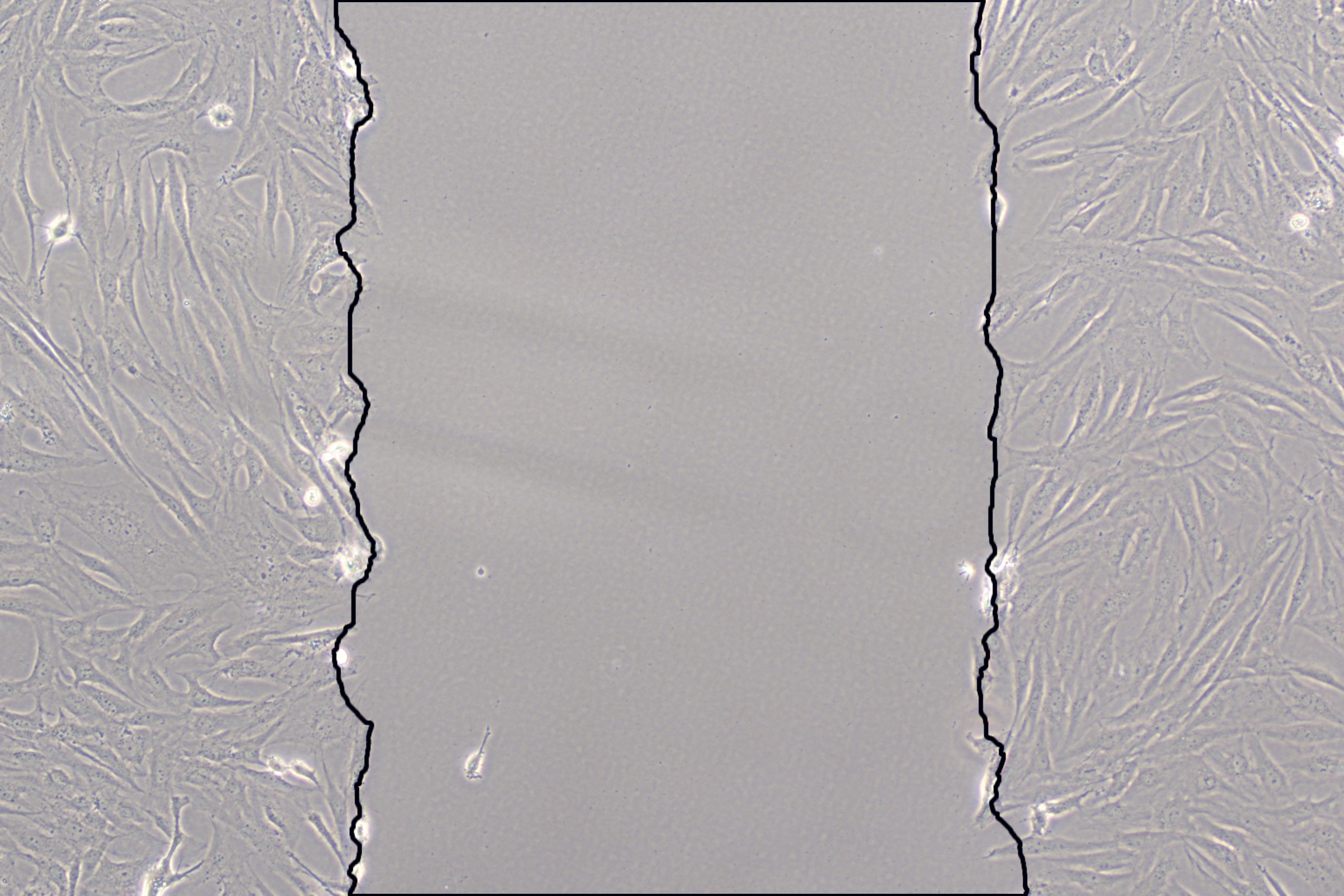

Supplement: S7 File — (ZIP) [file pone.0324264.s007.zip › supplement.material-7/images(Cell Scratch Assay)-HSF-0h/Model5.jpg]

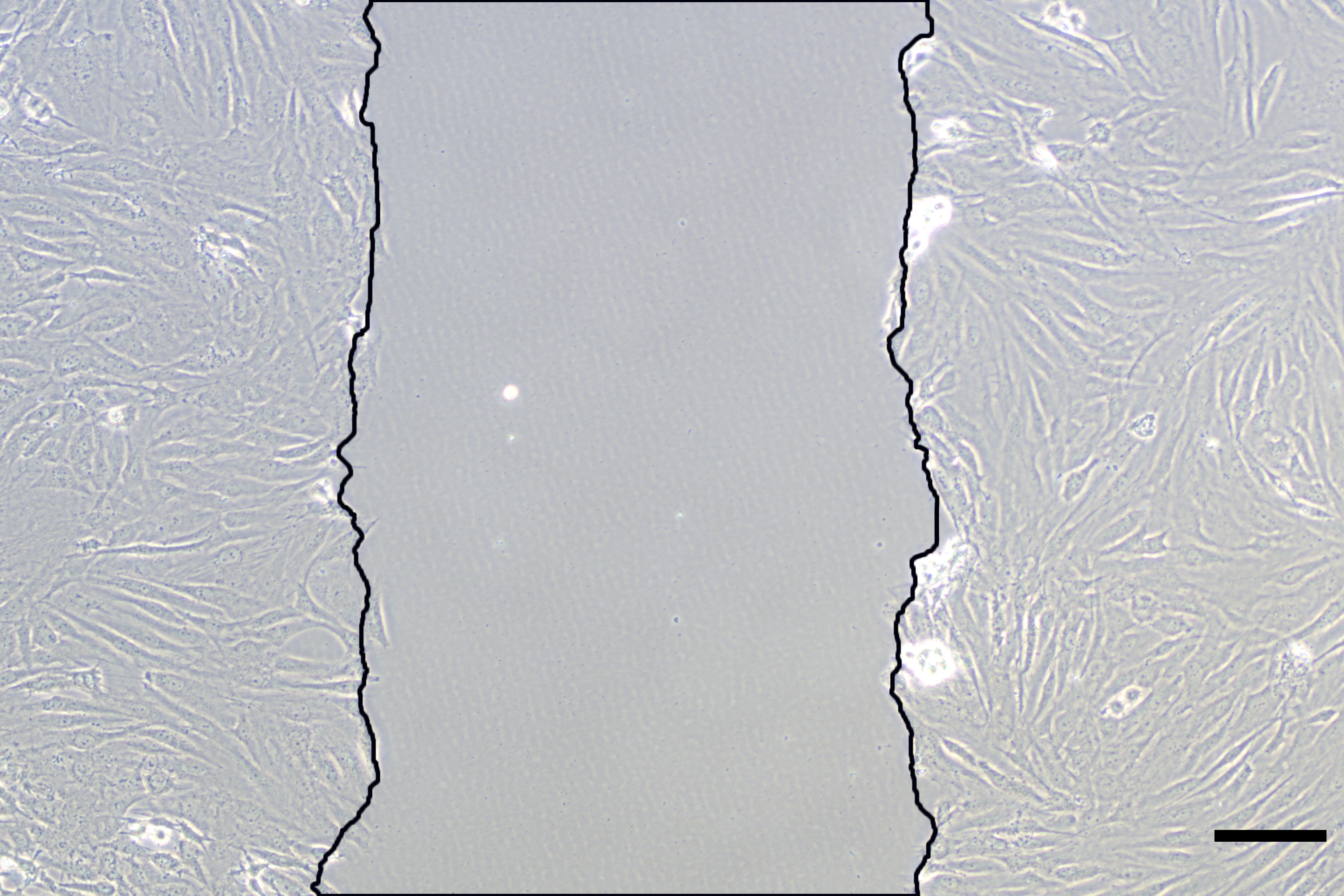

Supplement: S7 File — (ZIP) [file pone.0324264.s007.zip › supplement.material-7/images(Cell Scratch Assay)-HSF-0h/PL10X1.png]

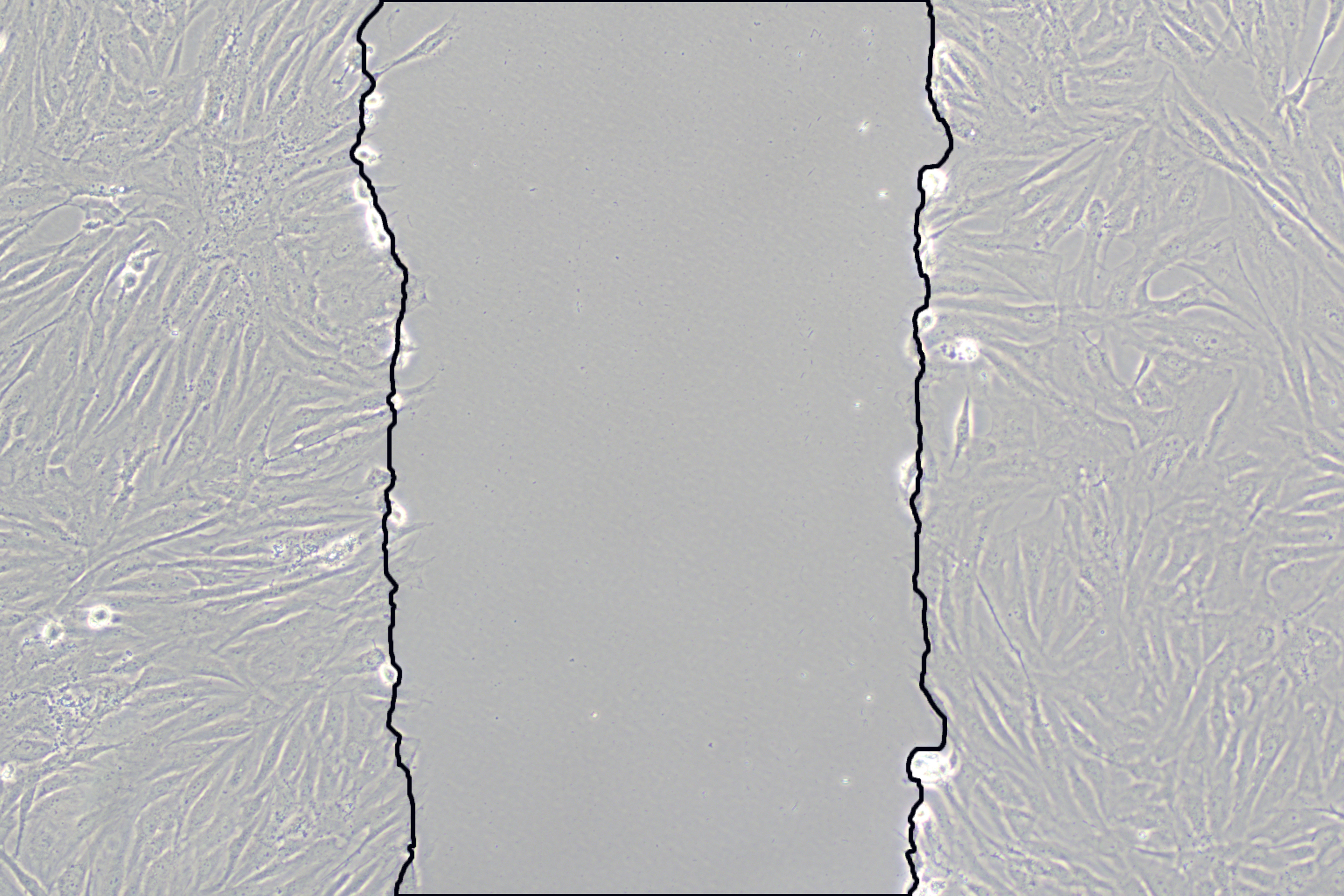

Supplement: S7 File — (ZIP) [file pone.0324264.s007.zip › supplement.material-7/images(Cell Scratch Assay)-HSF-0h/PL10x2.jpg]

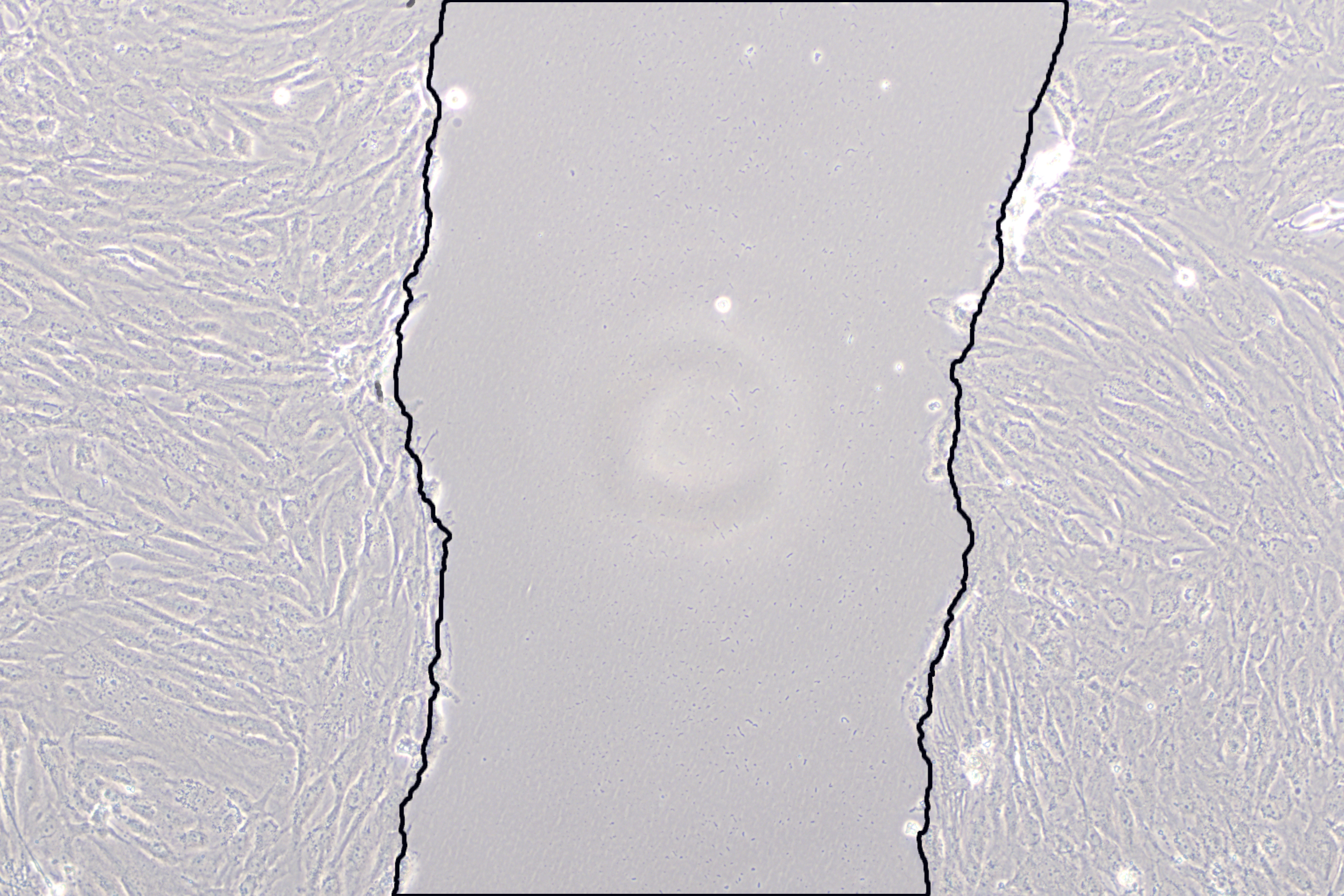

Supplement: S7 File — (ZIP) [file pone.0324264.s007.zip › supplement.material-7/images(Cell Scratch Assay)-HSF-0h/PL10X3.jpg]

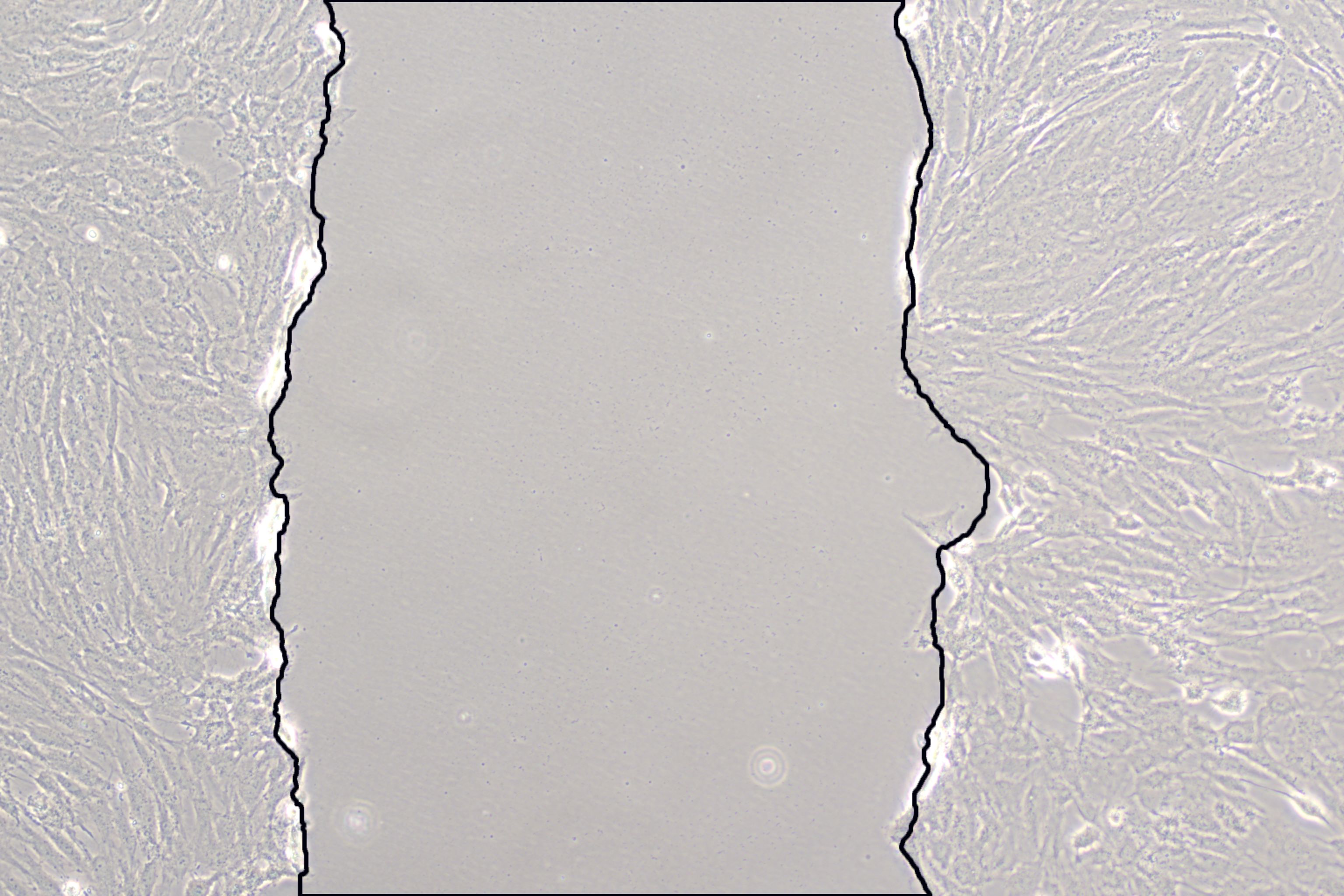

Supplement: S7 File — (ZIP) [file pone.0324264.s007.zip › supplement.material-7/images(Cell Scratch Assay)-HSF-0h/PL10X4.jpg]

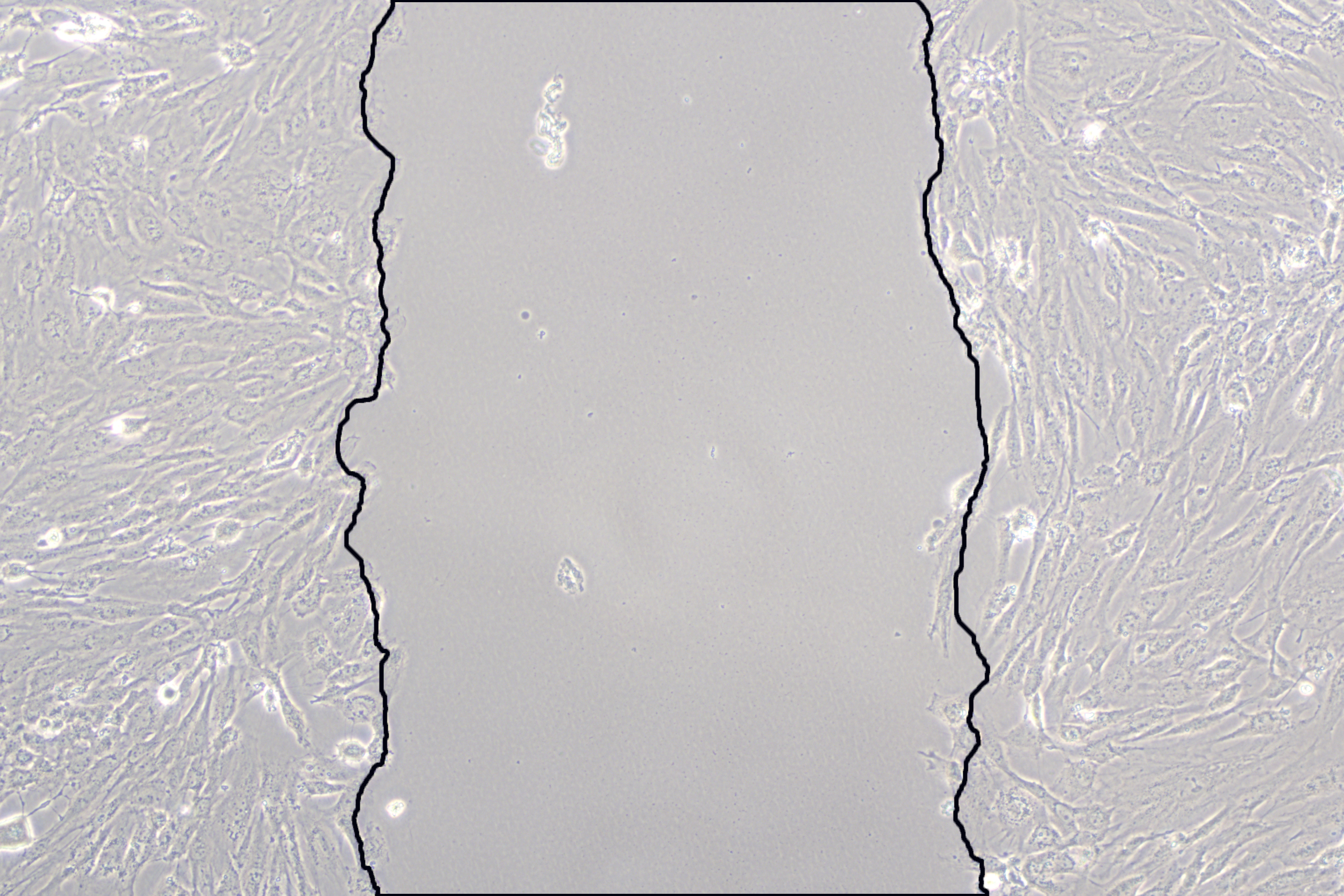

Supplement: S7 File — (ZIP) [file pone.0324264.s007.zip › supplement.material-7/images(Cell Scratch Assay)-HSF-0h/PL10X5.jpg]

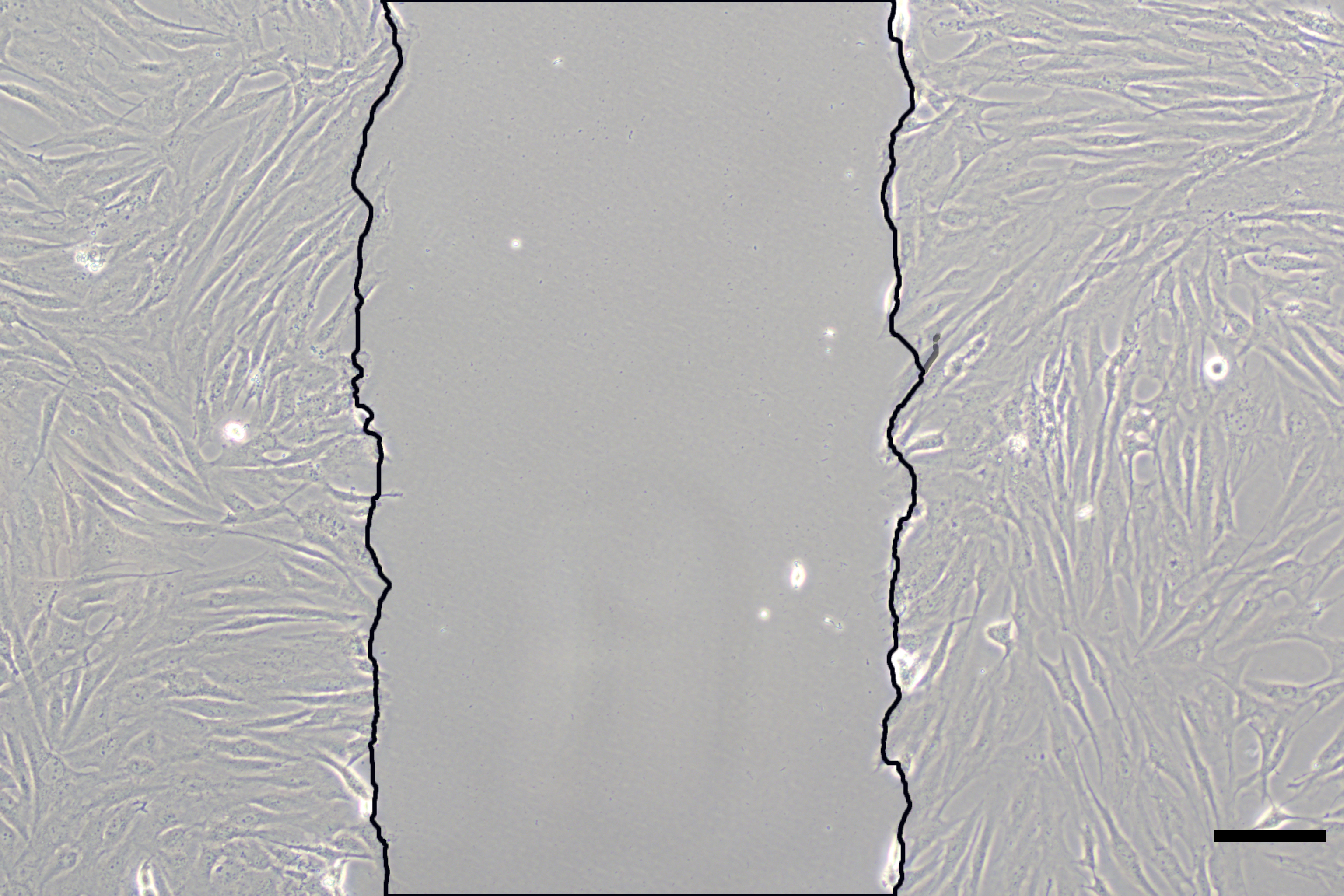

Supplement: S7 File — (ZIP) [file pone.0324264.s007.zip › supplement.material-7/images(Cell Scratch Assay)-HSF-0h/PL20x1.png]

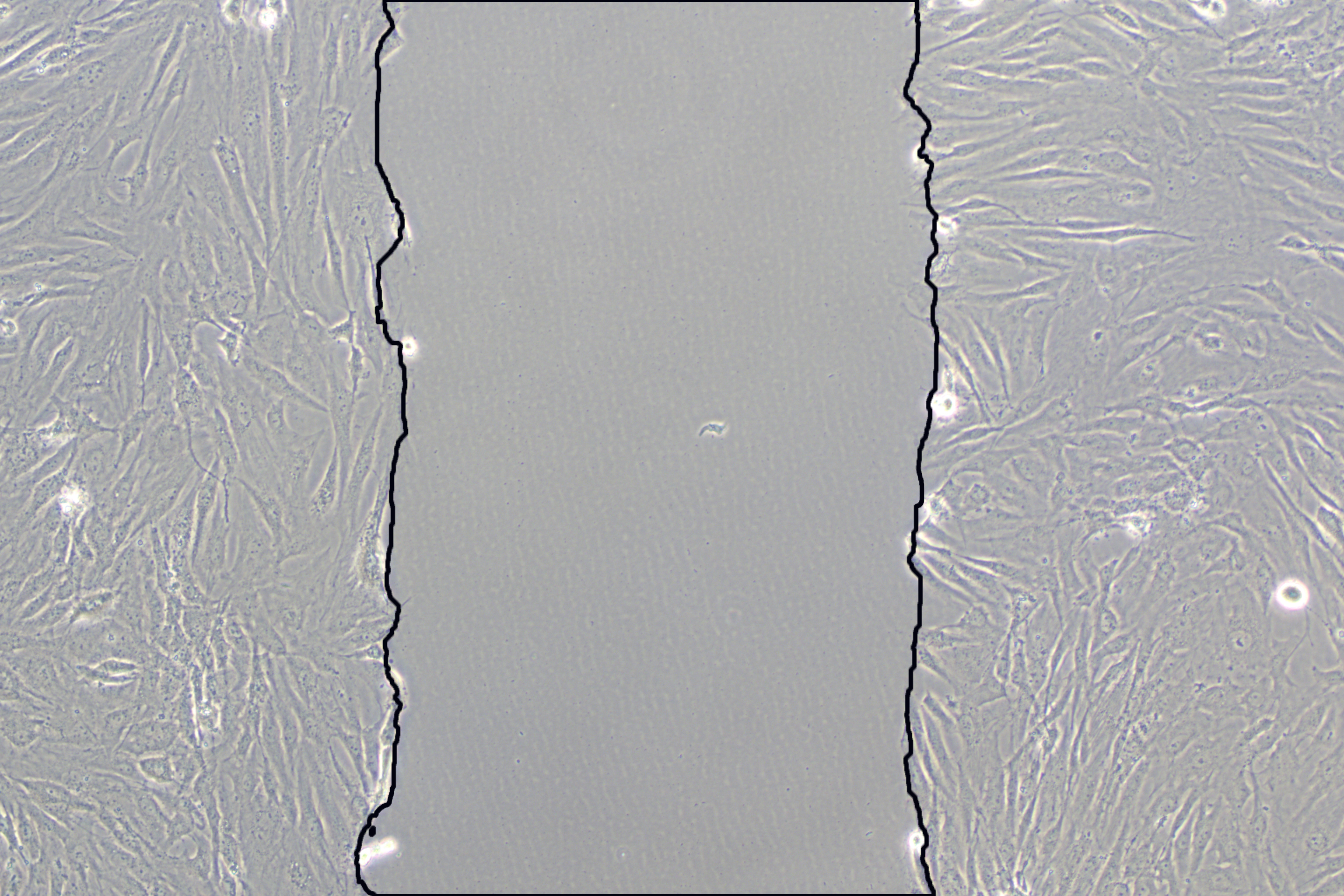

Supplement: S7 File — (ZIP) [file pone.0324264.s007.zip › supplement.material-7/images(Cell Scratch Assay)-HSF-0h/PL20X2.jpg]

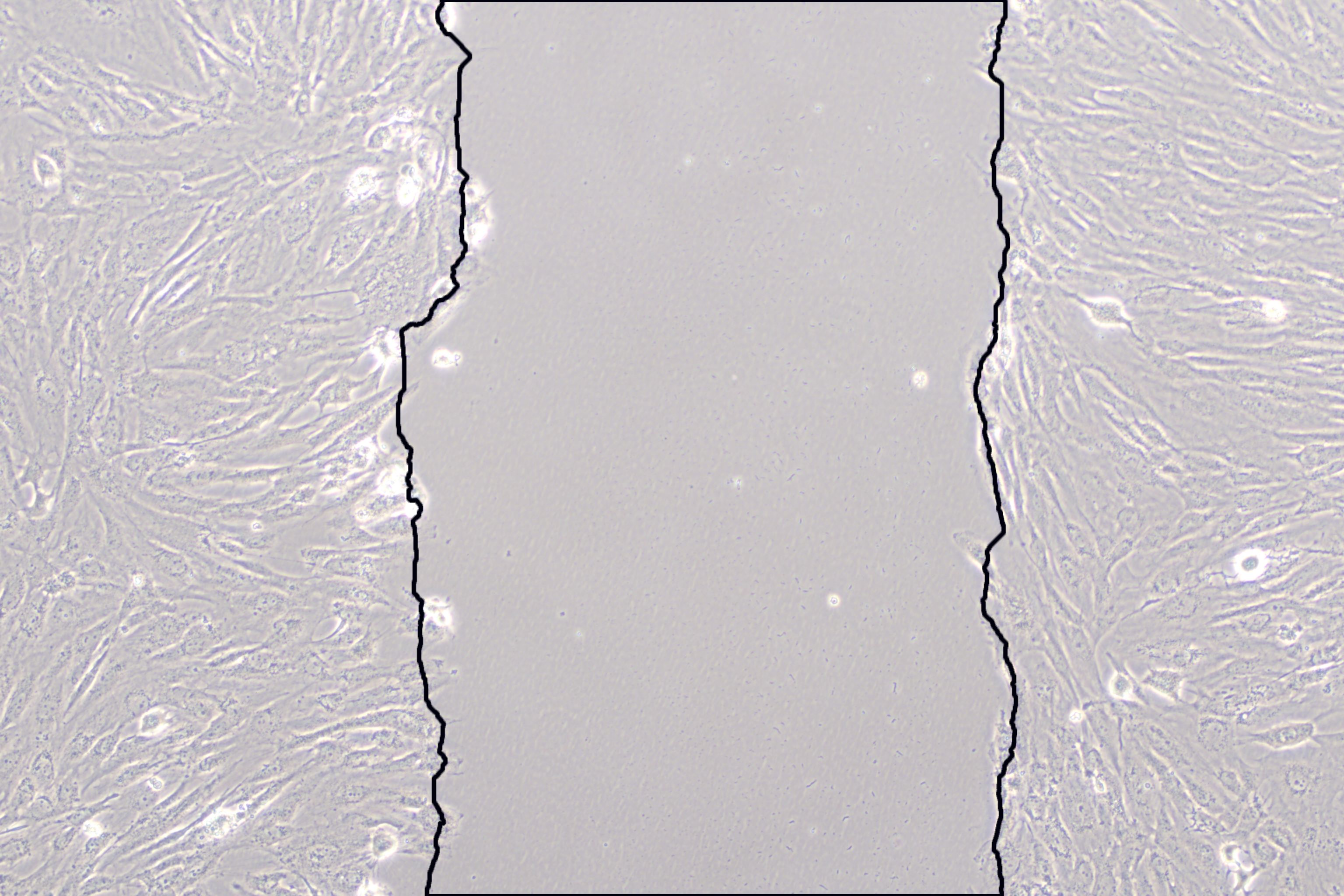

Supplement: S7 File — (ZIP) [file pone.0324264.s007.zip › supplement.material-7/images(Cell Scratch Assay)-HSF-0h/PL20X3.jpg]

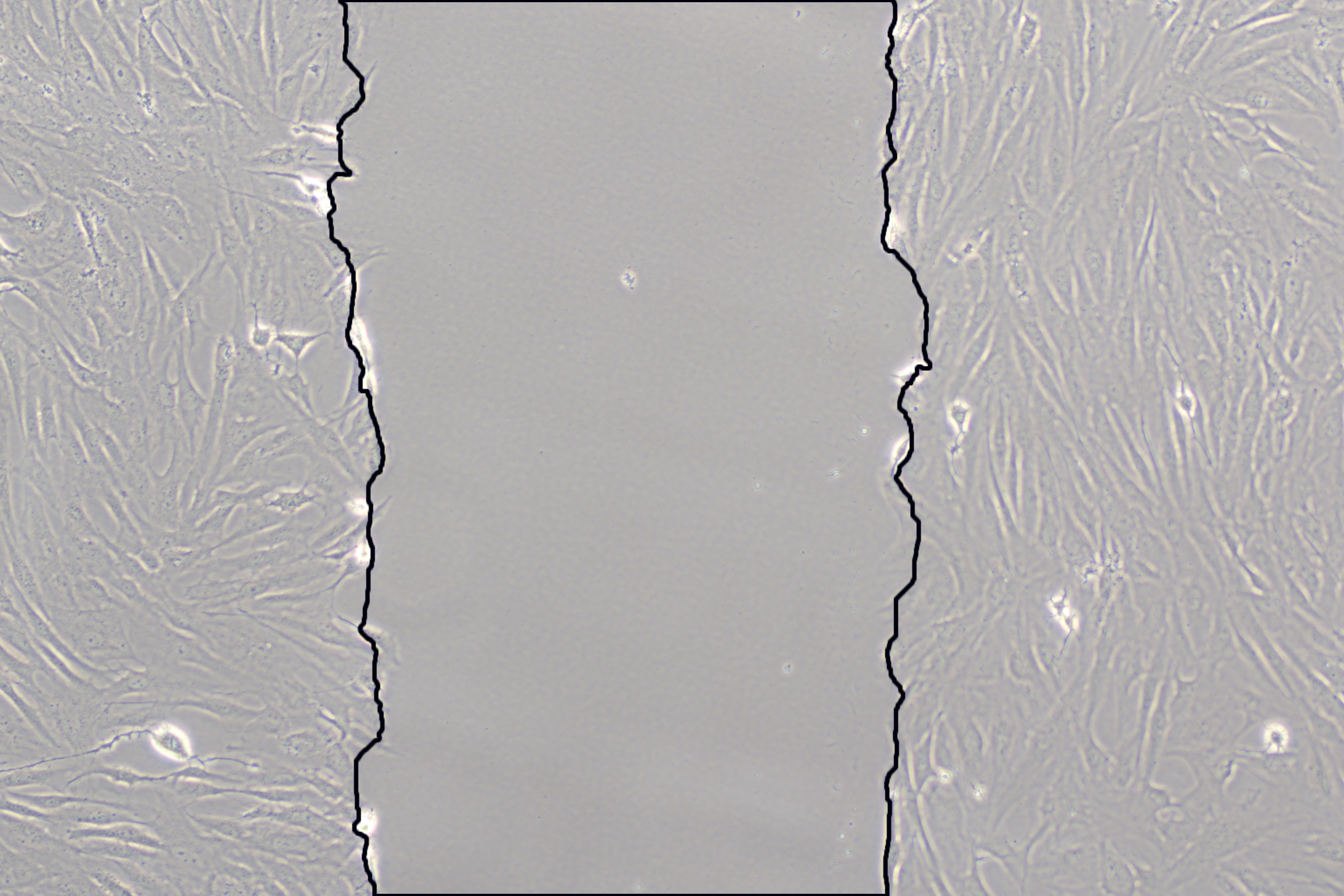

Supplement: S7 File — (ZIP) [file pone.0324264.s007.zip › supplement.material-7/images(Cell Scratch Assay)-HSF-0h/PL20X4.jpg]

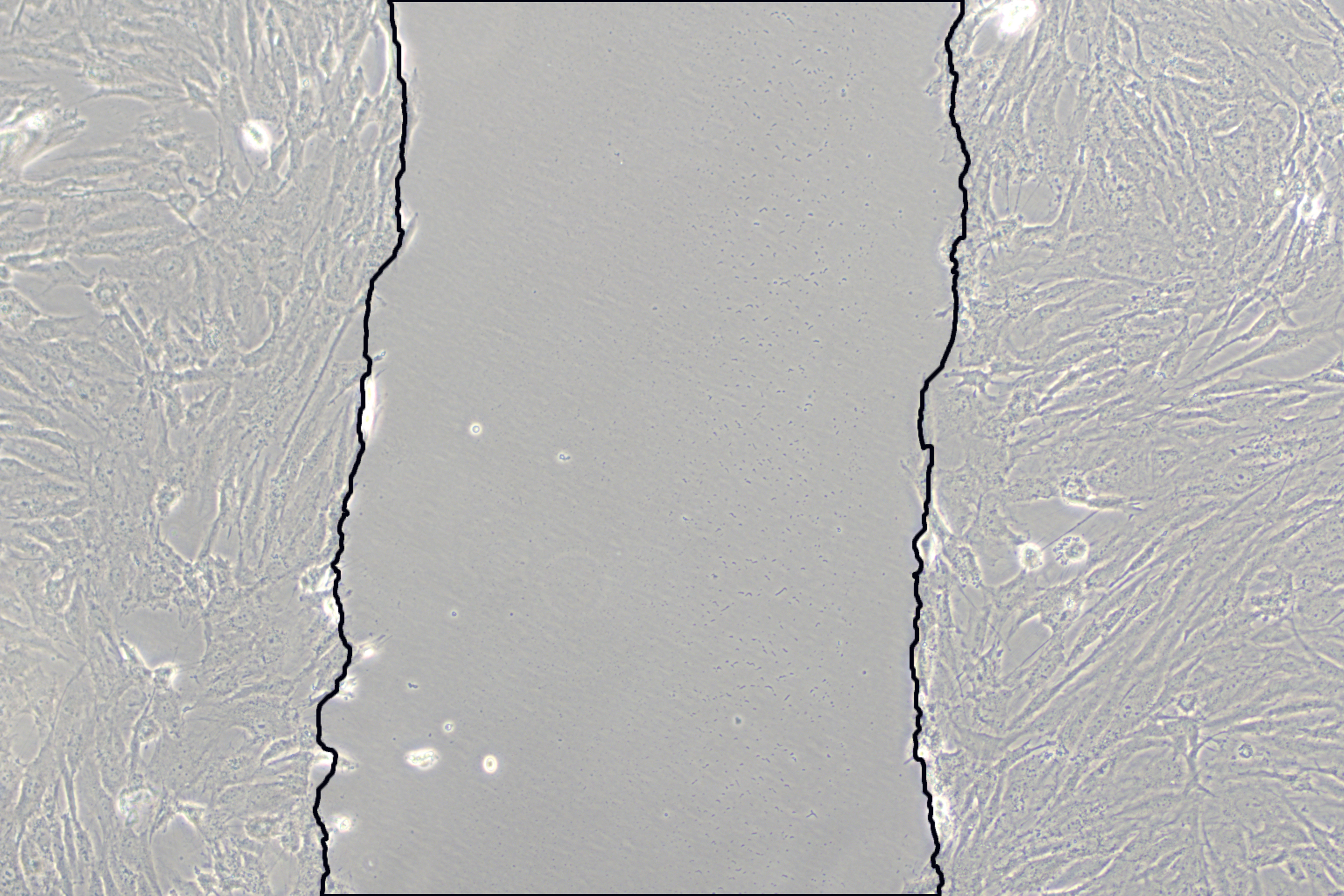

Supplement: S7 File — (ZIP) [file pone.0324264.s007.zip › supplement.material-7/images(Cell Scratch Assay)-HSF-0h/PL20X5.jpg]

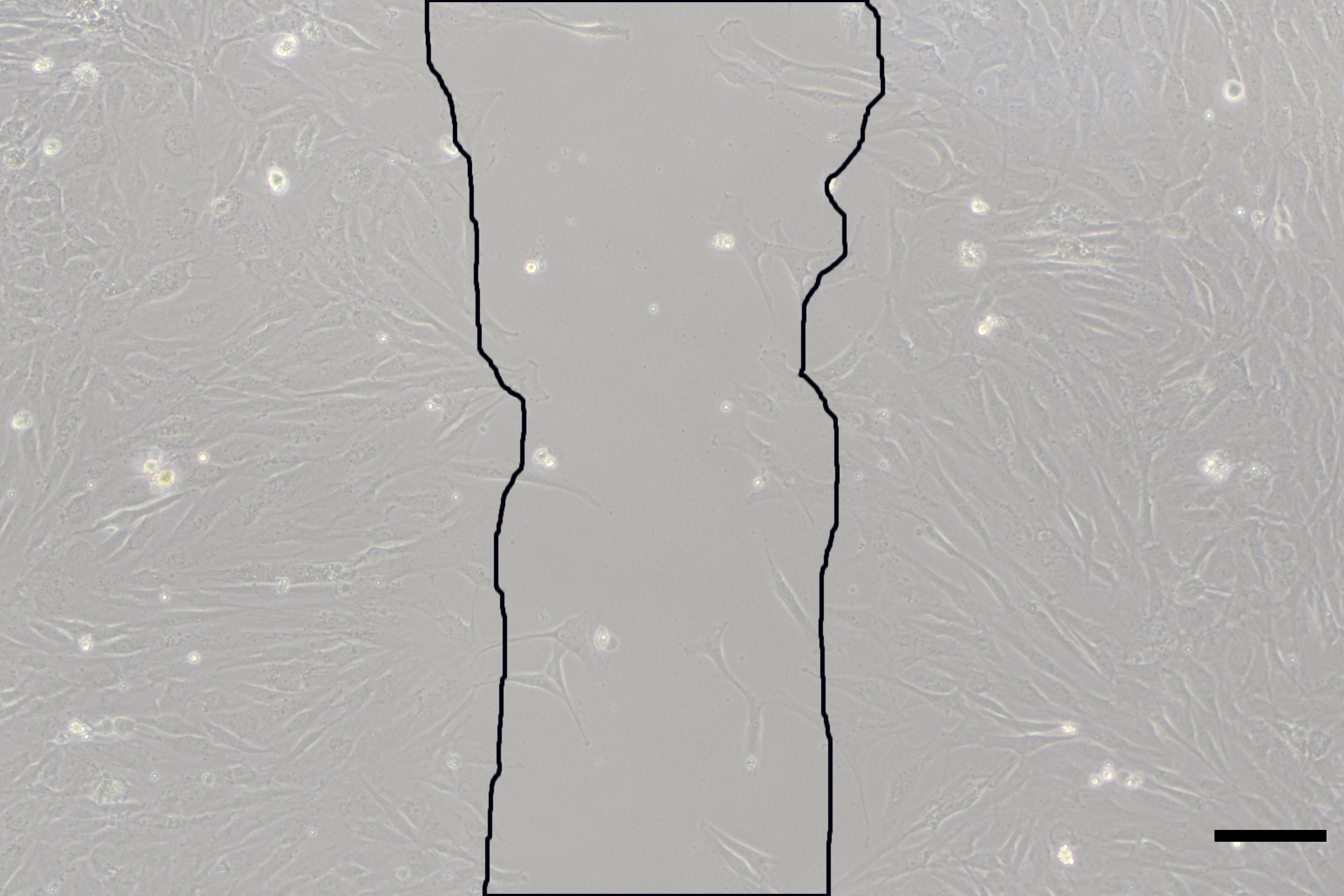

Supplement: S8 File — (ZIP) [file pone.0324264.s008.zip › supplement.material-8/images(Cell Scratch Assay)-HSF-12h/Control1.png]

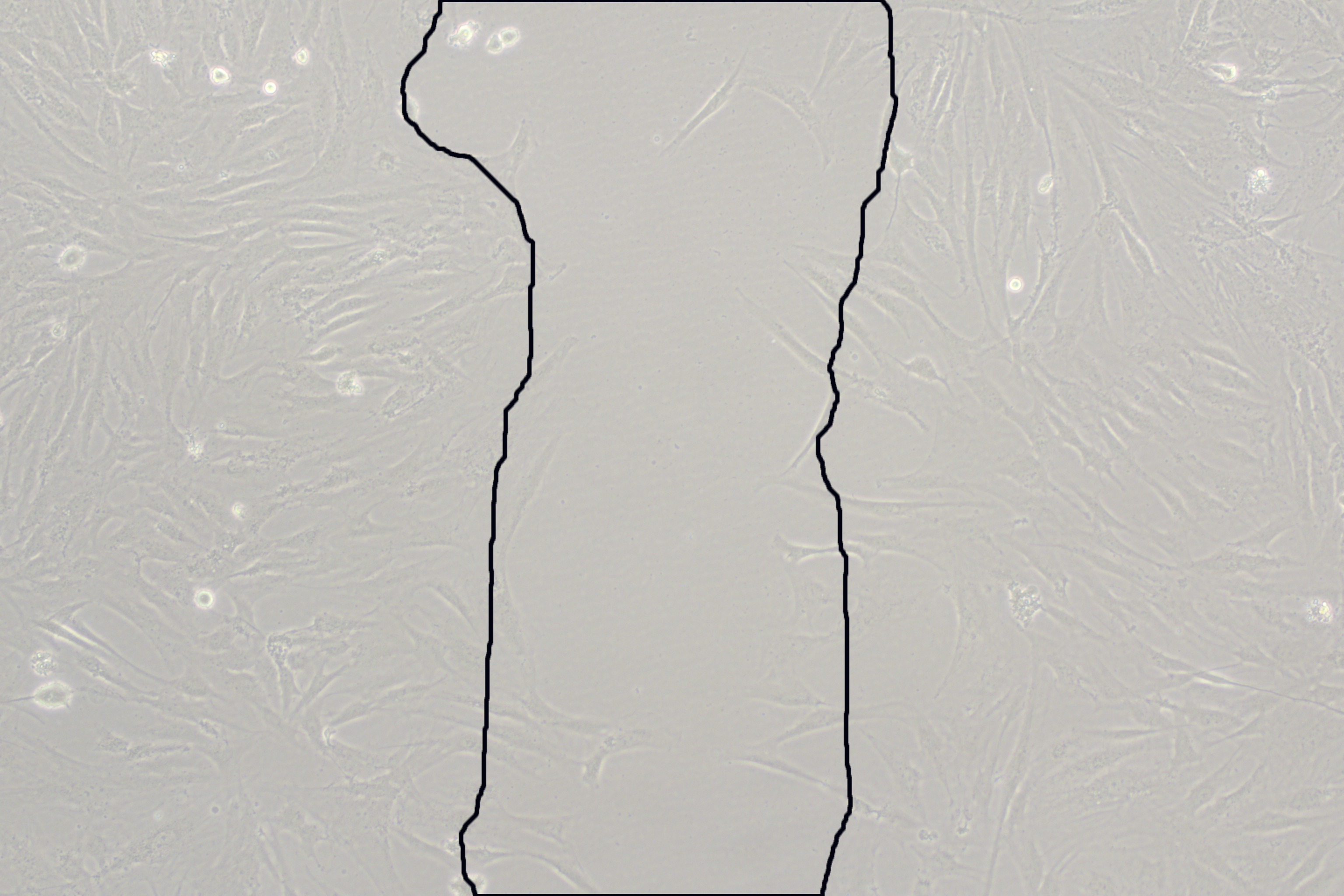

Supplement: S8 File — (ZIP) [file pone.0324264.s008.zip › supplement.material-8/images(Cell Scratch Assay)-HSF-12h/Control2.jpg]

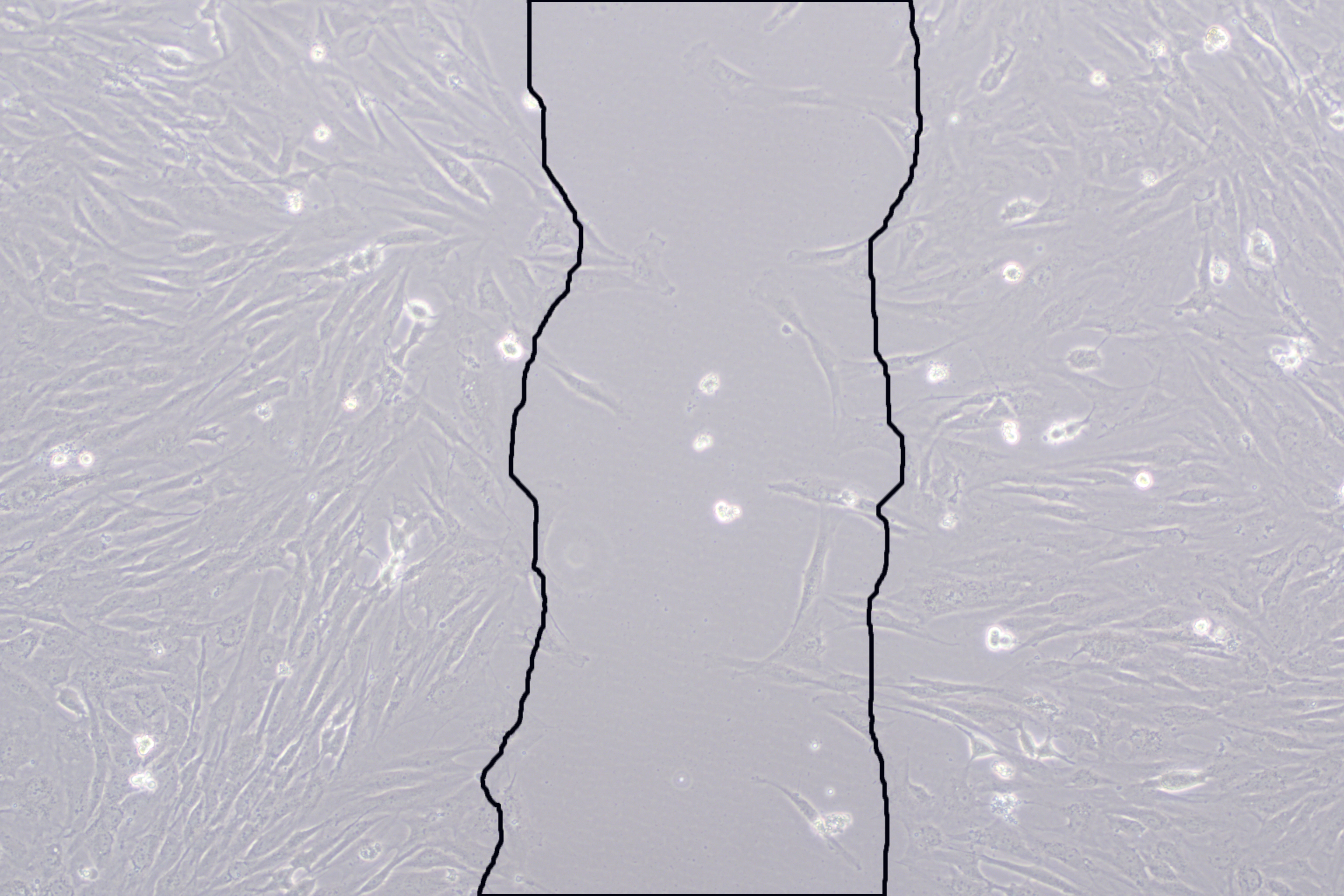

Supplement: S8 File — (ZIP) [file pone.0324264.s008.zip › supplement.material-8/images(Cell Scratch Assay)-HSF-12h/Control3.jpg]

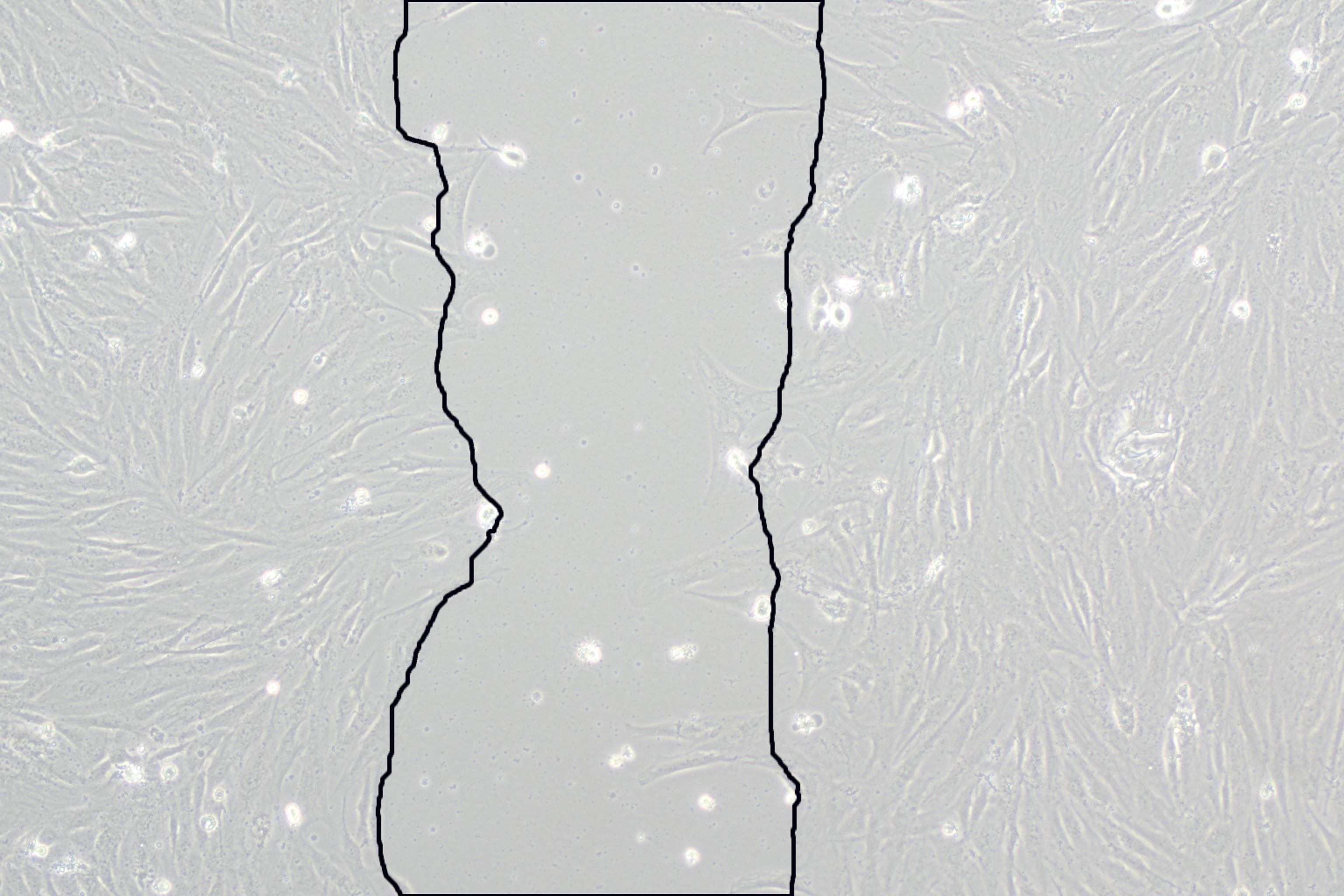

Supplement: S8 File — (ZIP) [file pone.0324264.s008.zip › supplement.material-8/images(Cell Scratch Assay)-HSF-12h/Control4.jpg]

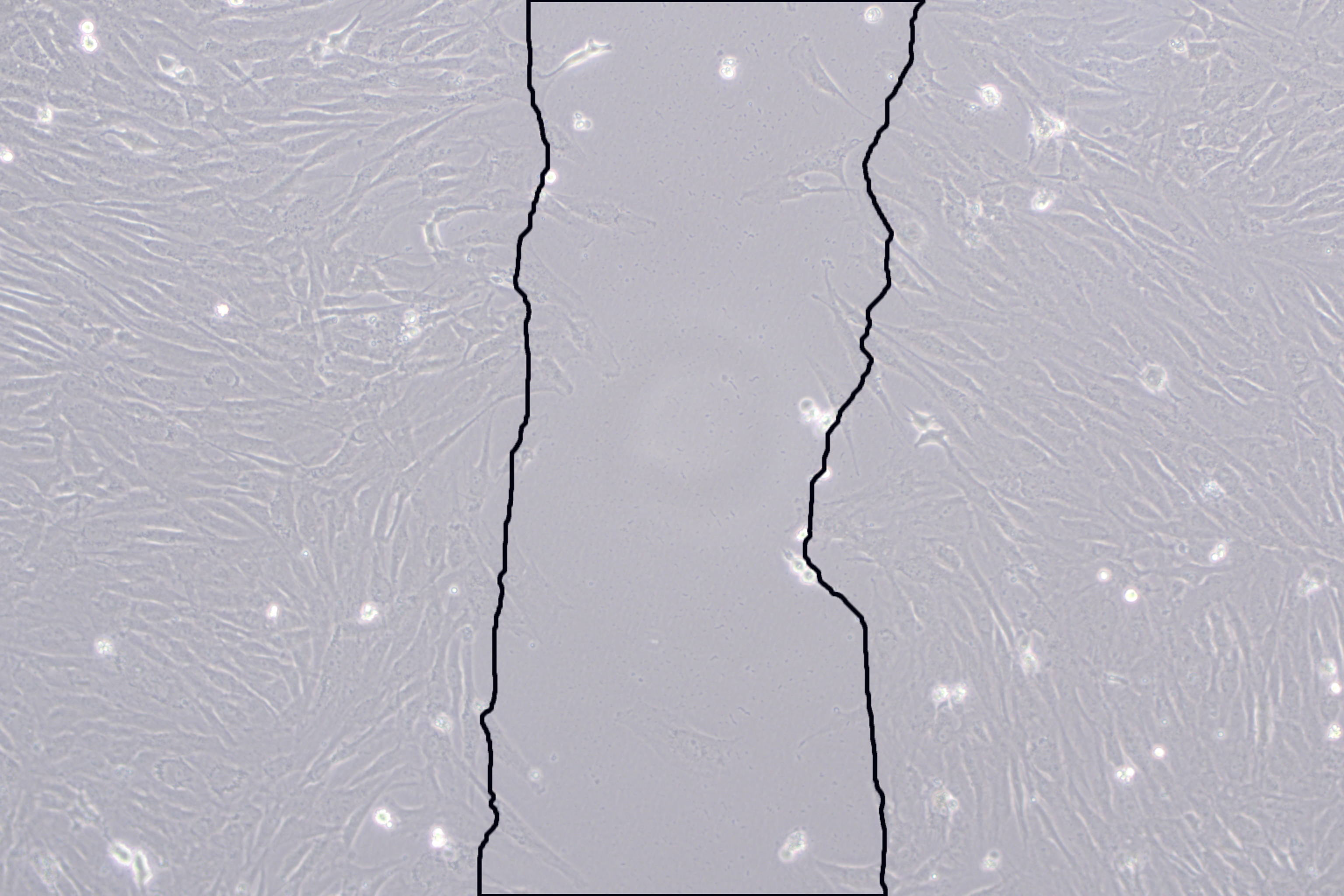

Supplement: S8 File — (ZIP) [file pone.0324264.s008.zip › supplement.material-8/images(Cell Scratch Assay)-HSF-12h/Control5.jpg]

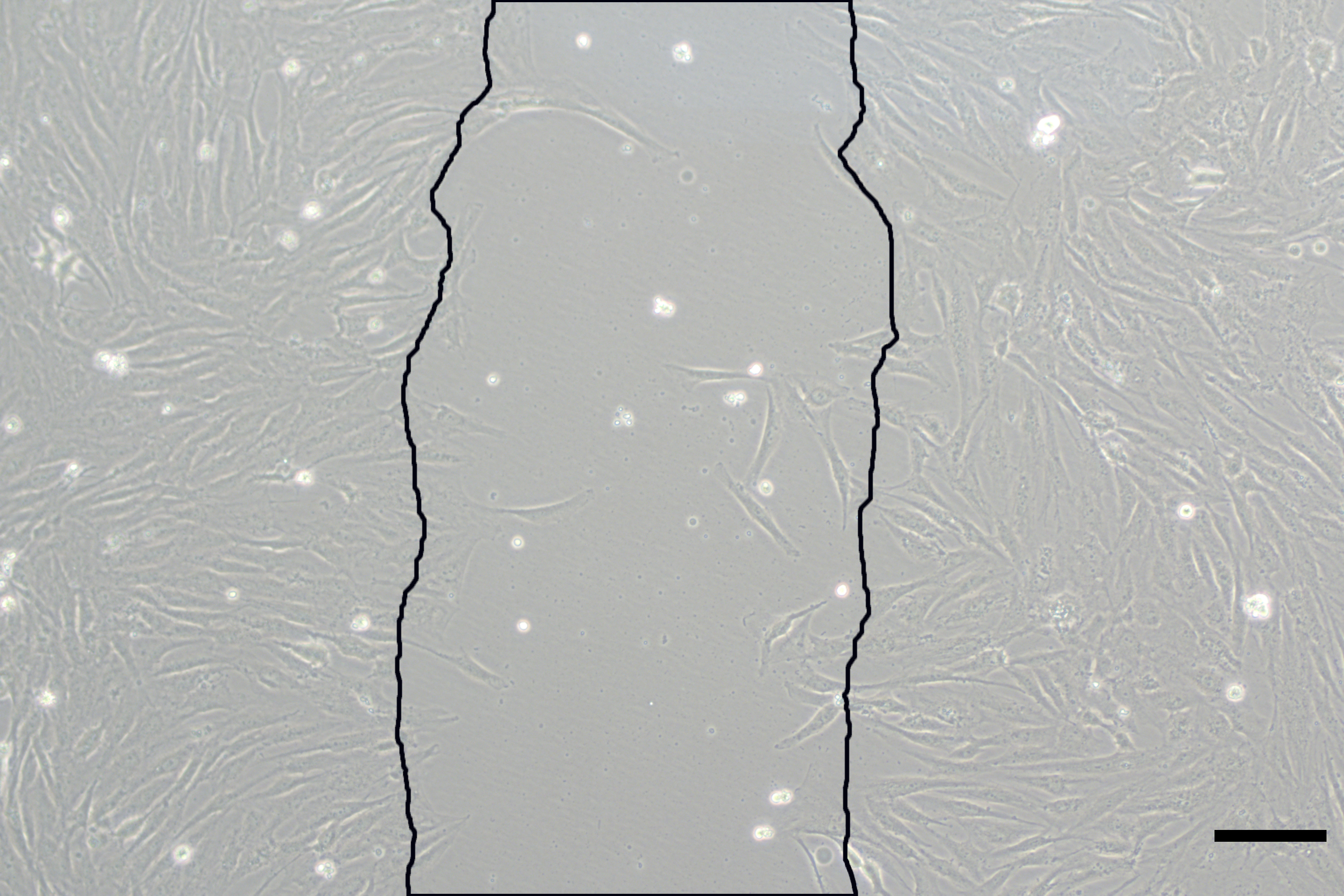

Supplement: S8 File — (ZIP) [file pone.0324264.s008.zip › supplement.material-8/images(Cell Scratch Assay)-HSF-12h/Model1.png]

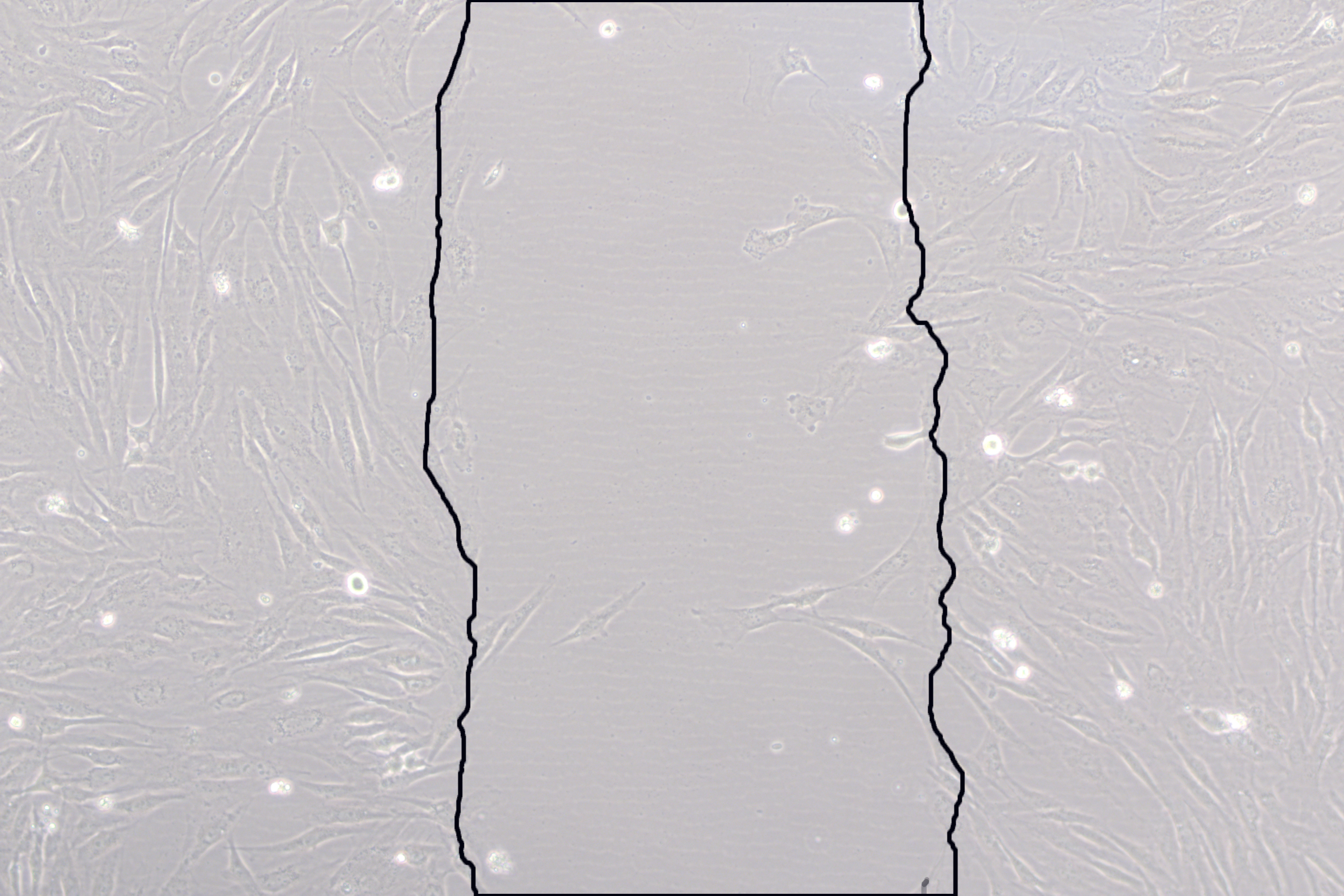

Supplement: S8 File — (ZIP) [file pone.0324264.s008.zip › supplement.material-8/images(Cell Scratch Assay)-HSF-12h/Model2.jpg]

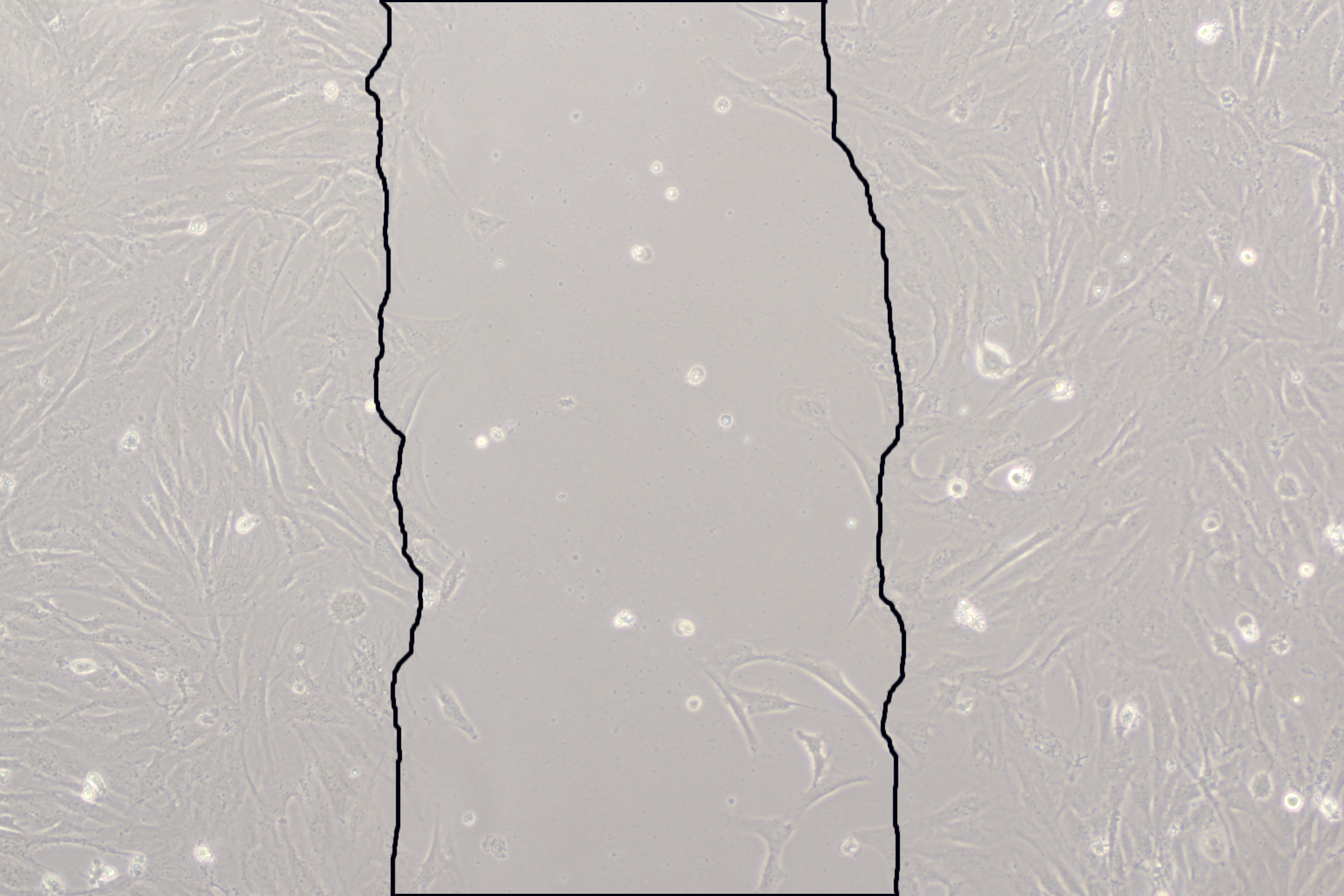

Supplement: S8 File — (ZIP) [file pone.0324264.s008.zip › supplement.material-8/images(Cell Scratch Assay)-HSF-12h/Model3.jpg]

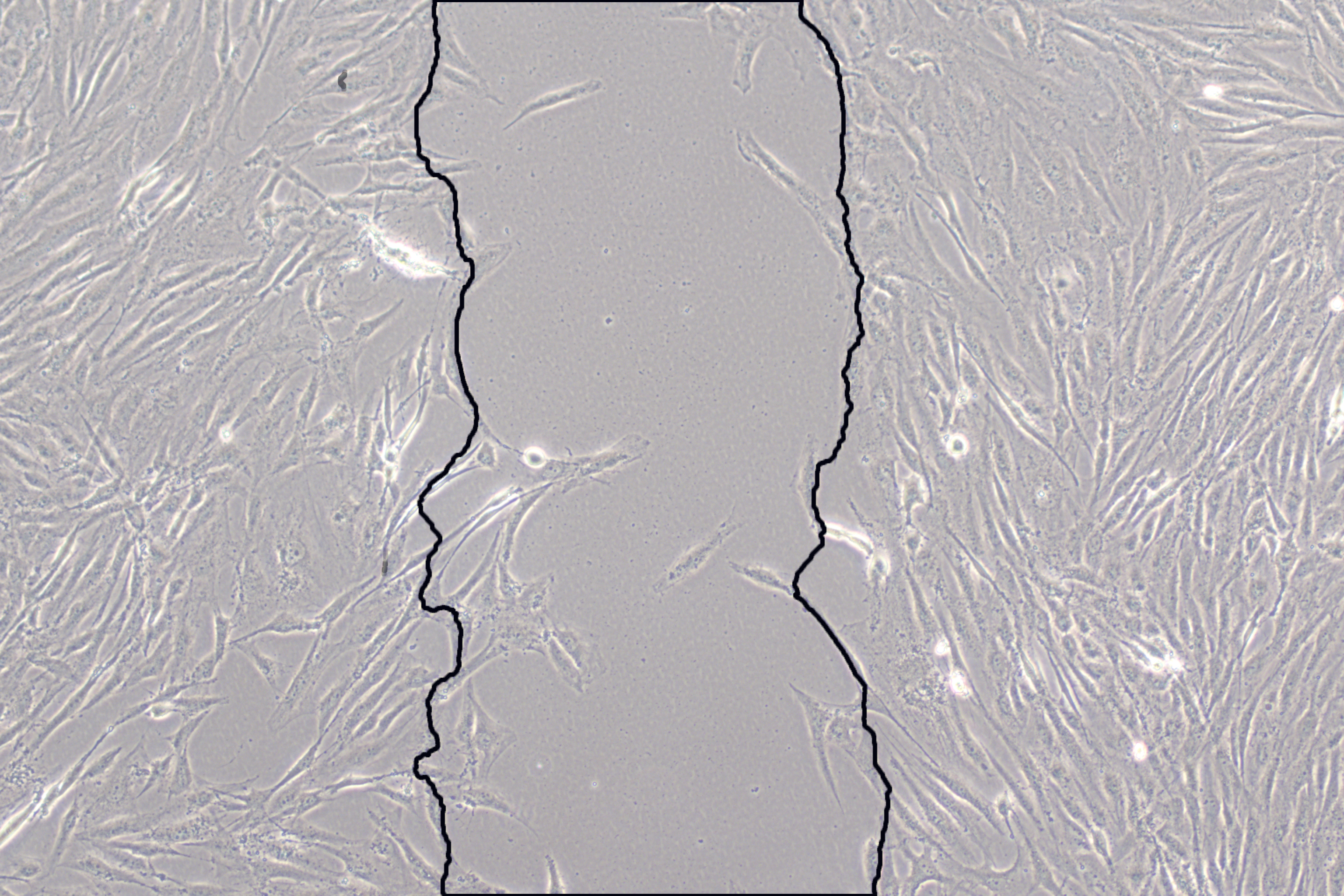

Supplement: S8 File — (ZIP) [file pone.0324264.s008.zip › supplement.material-8/images(Cell Scratch Assay)-HSF-12h/Model4.jpg]

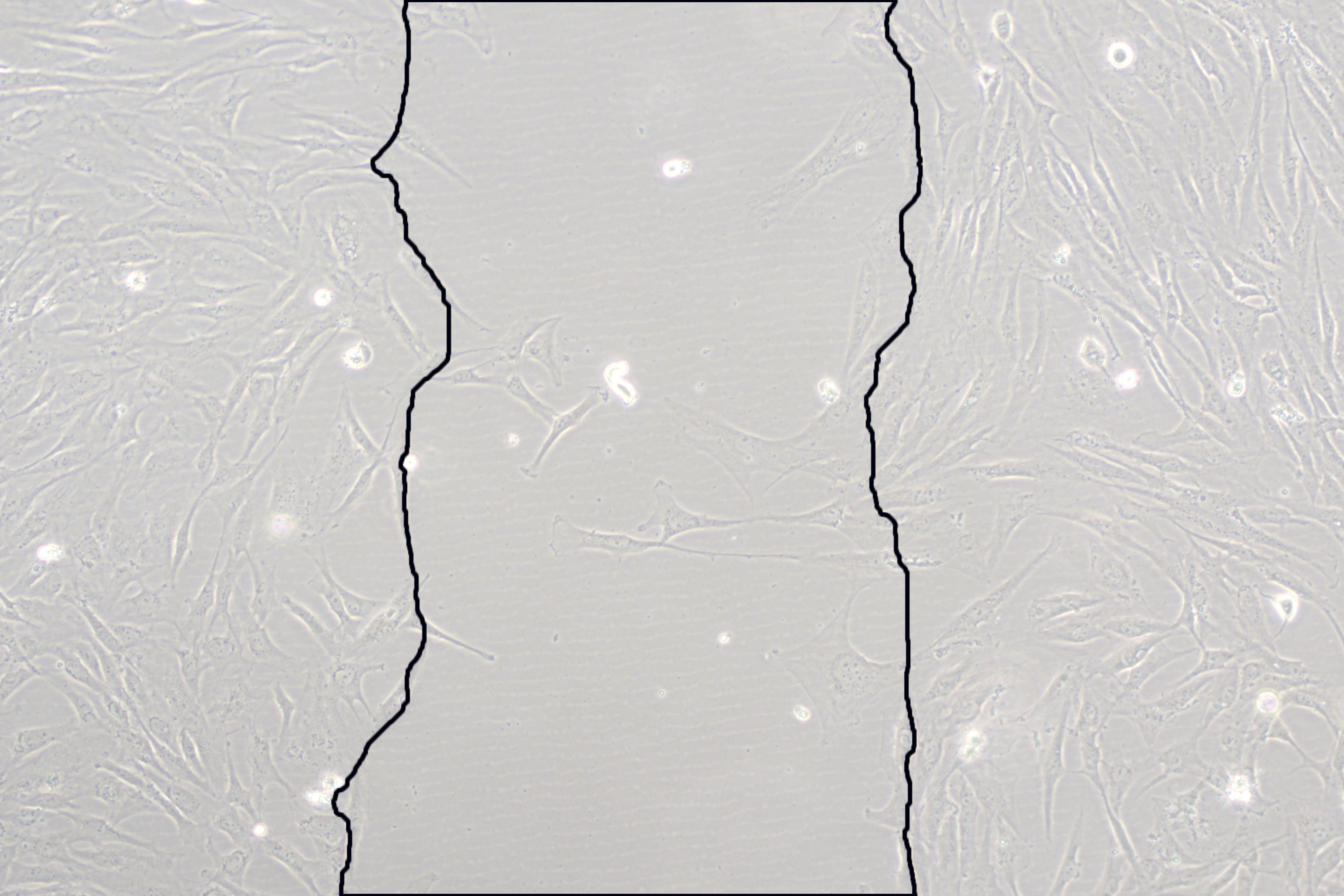

Supplement: S8 File — (ZIP) [file pone.0324264.s008.zip › supplement.material-8/images(Cell Scratch Assay)-HSF-12h/Model5.jpg]

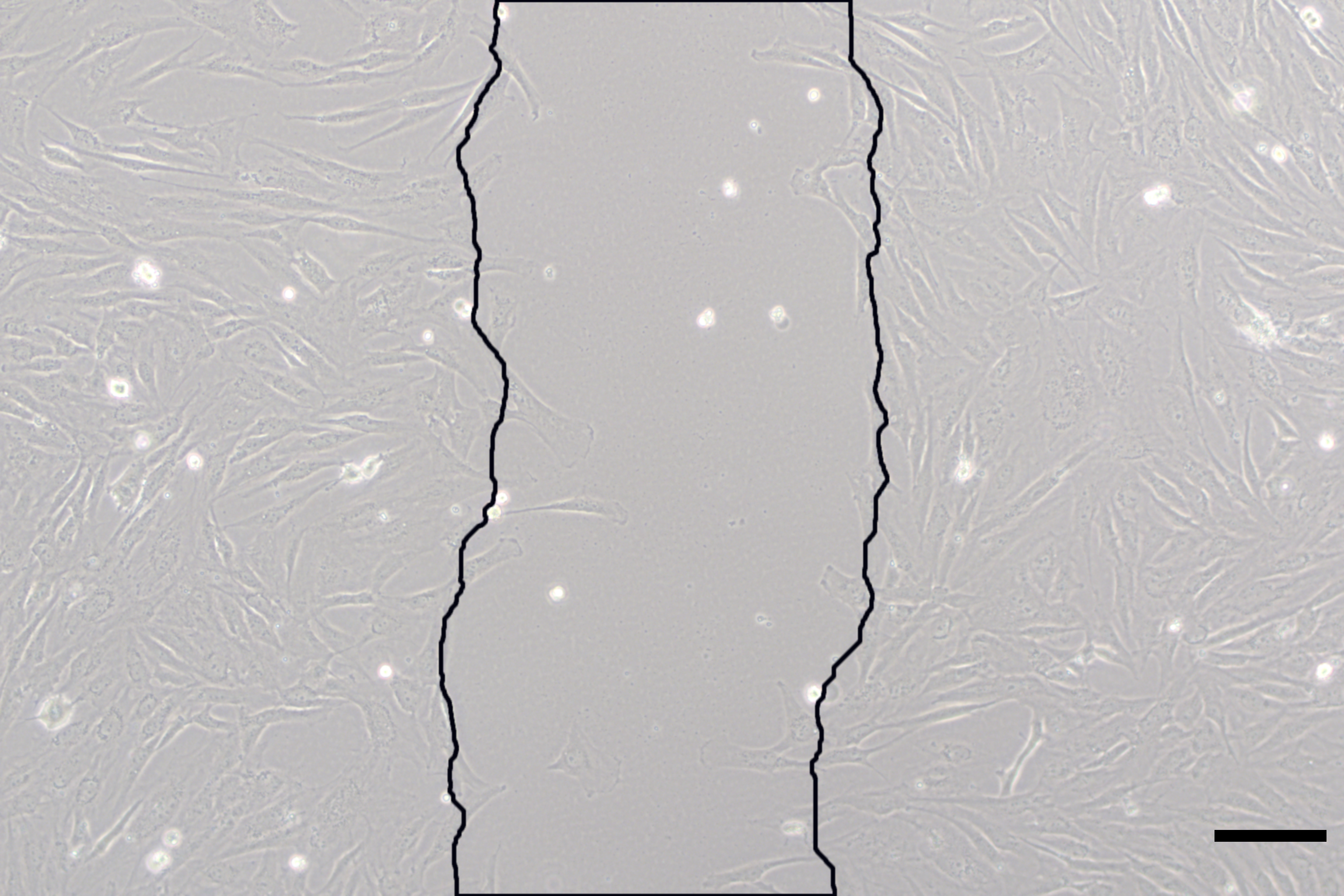

Supplement: S8 File — (ZIP) [file pone.0324264.s008.zip › supplement.material-8/images(Cell Scratch Assay)-HSF-12h/PL10X1.png]

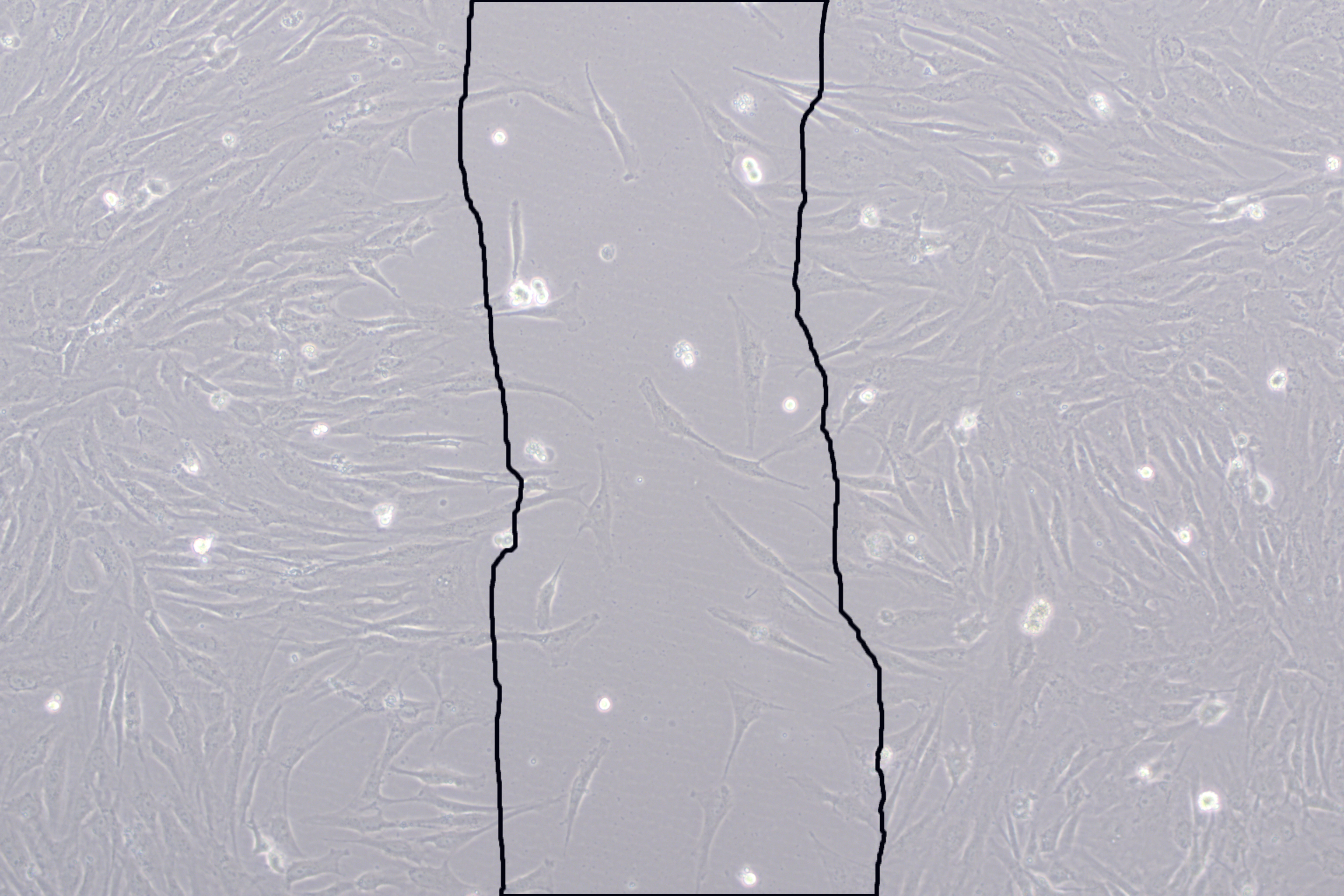

Supplement: S8 File — (ZIP) [file pone.0324264.s008.zip › supplement.material-8/images(Cell Scratch Assay)-HSF-12h/PL10X2.jpg]

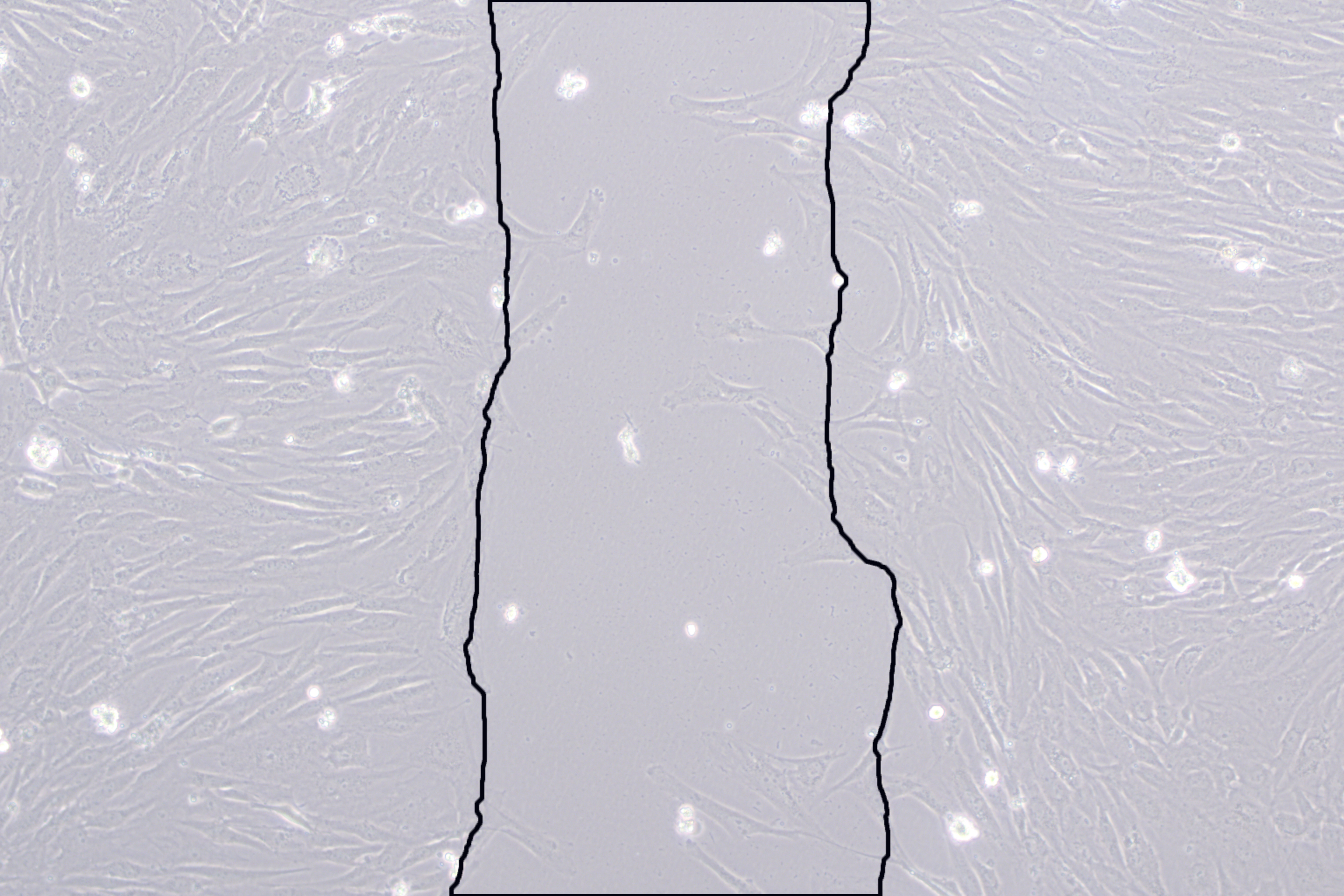

Supplement: S8 File — (ZIP) [file pone.0324264.s008.zip › supplement.material-8/images(Cell Scratch Assay)-HSF-12h/PL10X3.jpg]

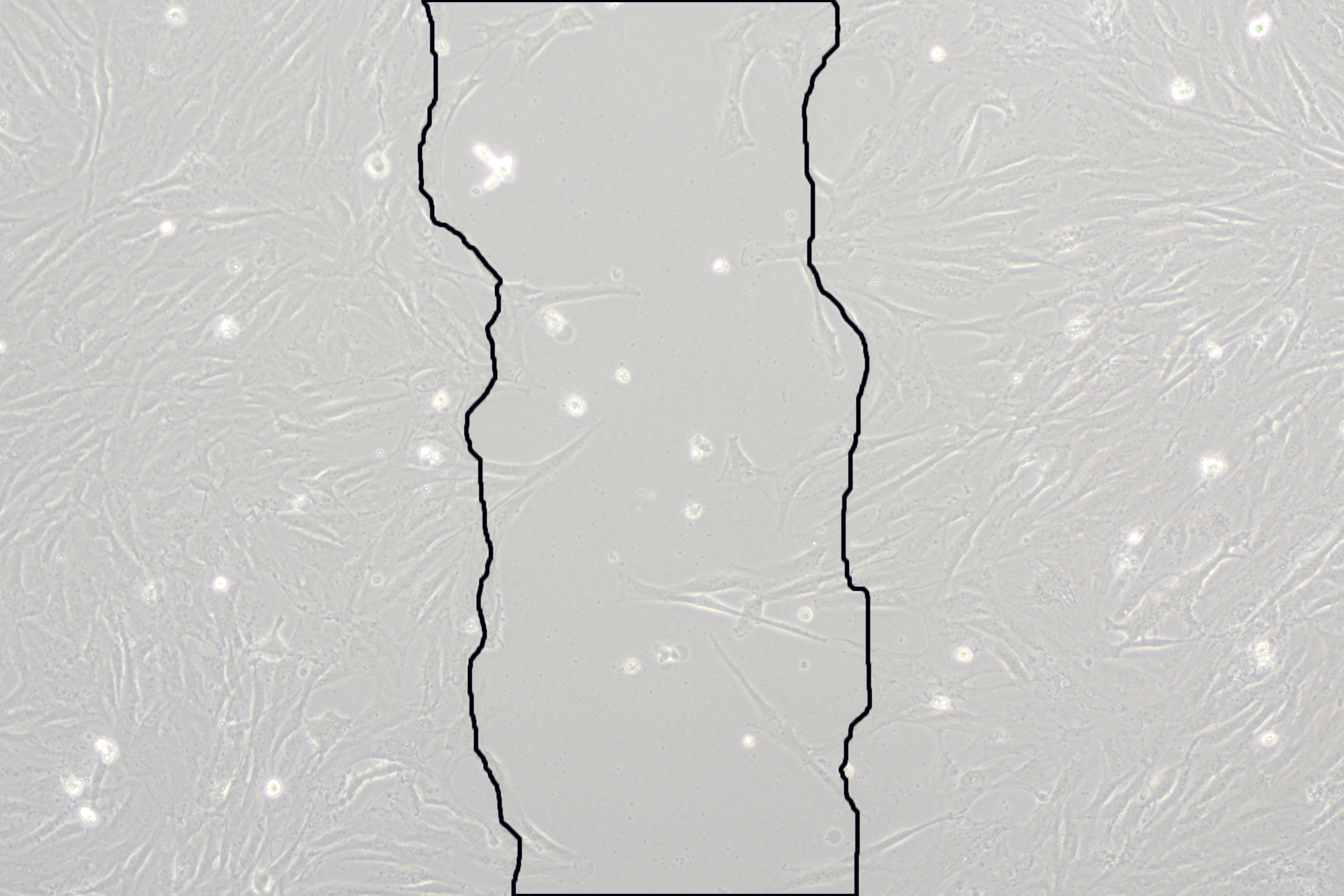

Supplement: S8 File — (ZIP) [file pone.0324264.s008.zip › supplement.material-8/images(Cell Scratch Assay)-HSF-12h/PL10X4.jpg]

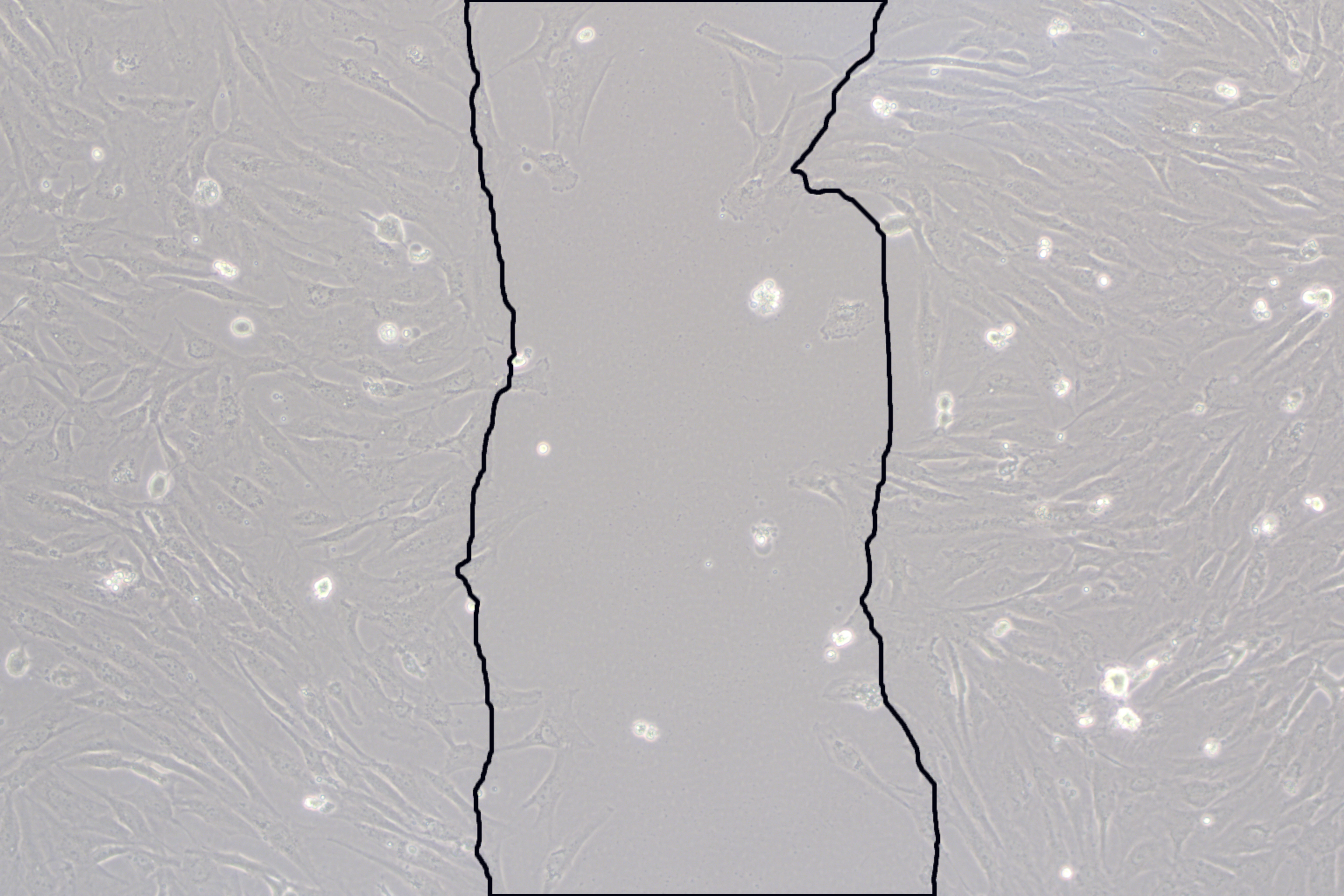

Supplement: S8 File — (ZIP) [file pone.0324264.s008.zip › supplement.material-8/images(Cell Scratch Assay)-HSF-12h/PL10X5.jpg]

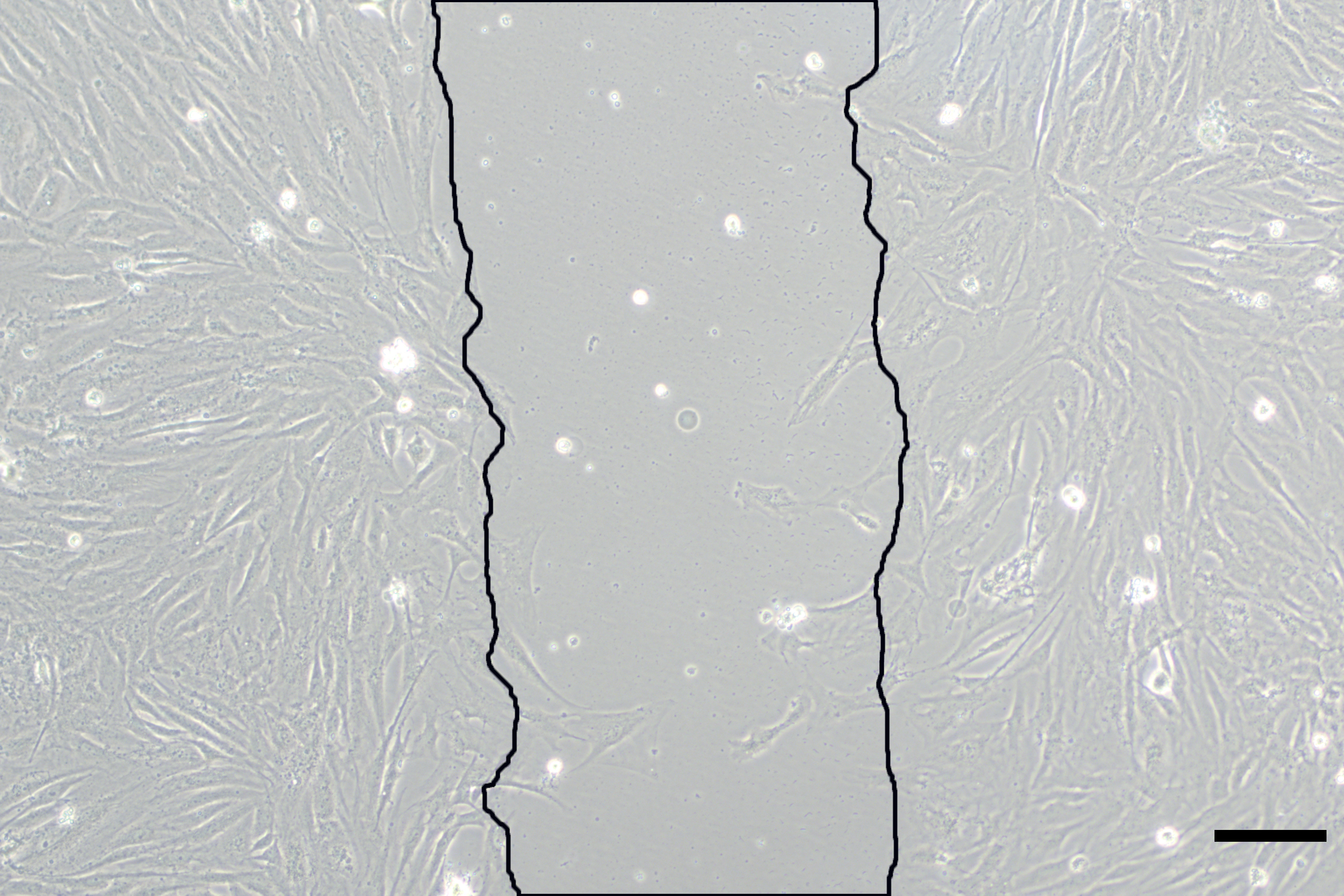

Supplement: S8 File — (ZIP) [file pone.0324264.s008.zip › supplement.material-8/images(Cell Scratch Assay)-HSF-12h/PL20X1.png]

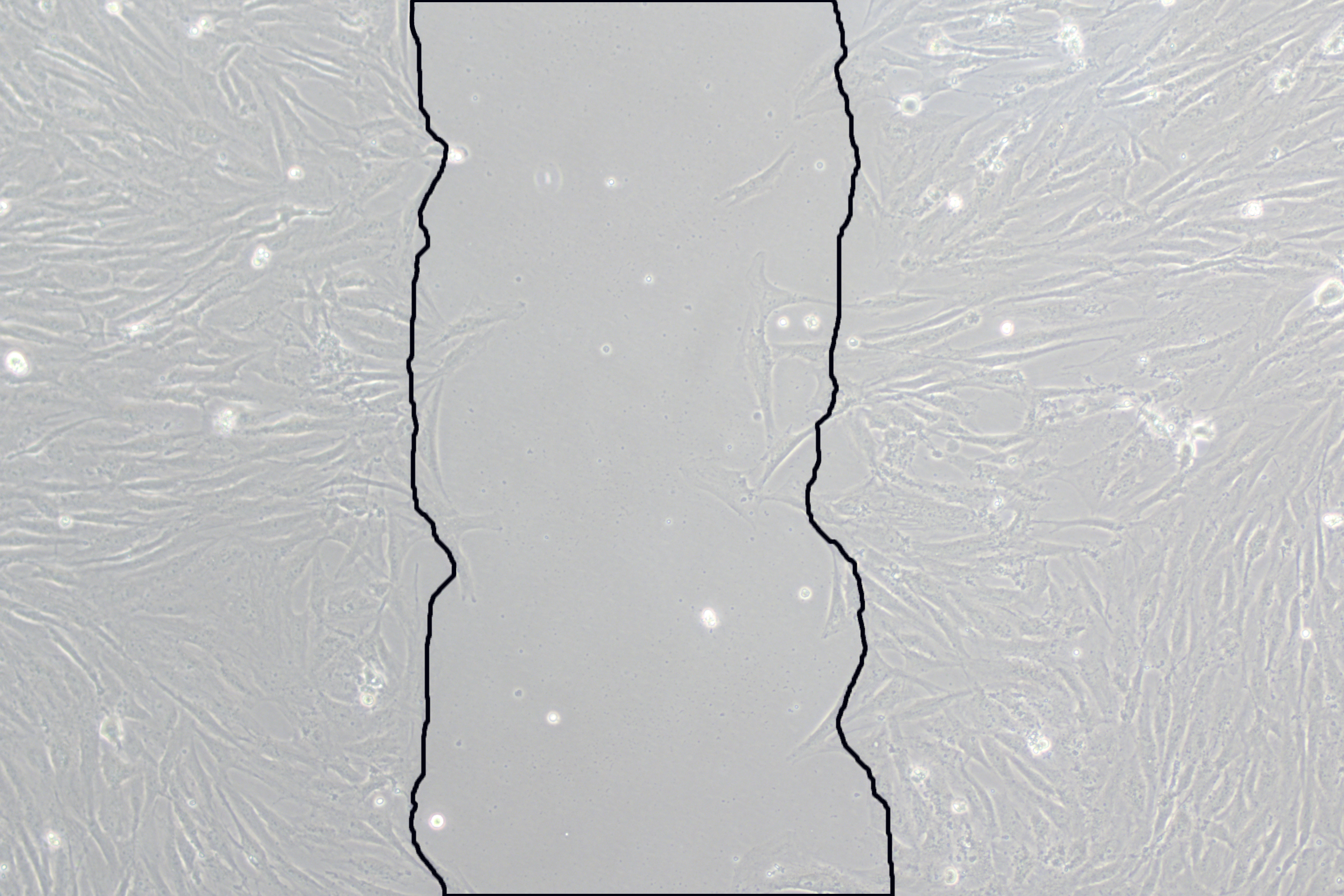

Supplement: S8 File — (ZIP) [file pone.0324264.s008.zip › supplement.material-8/images(Cell Scratch Assay)-HSF-12h/PL20X2.jpg]

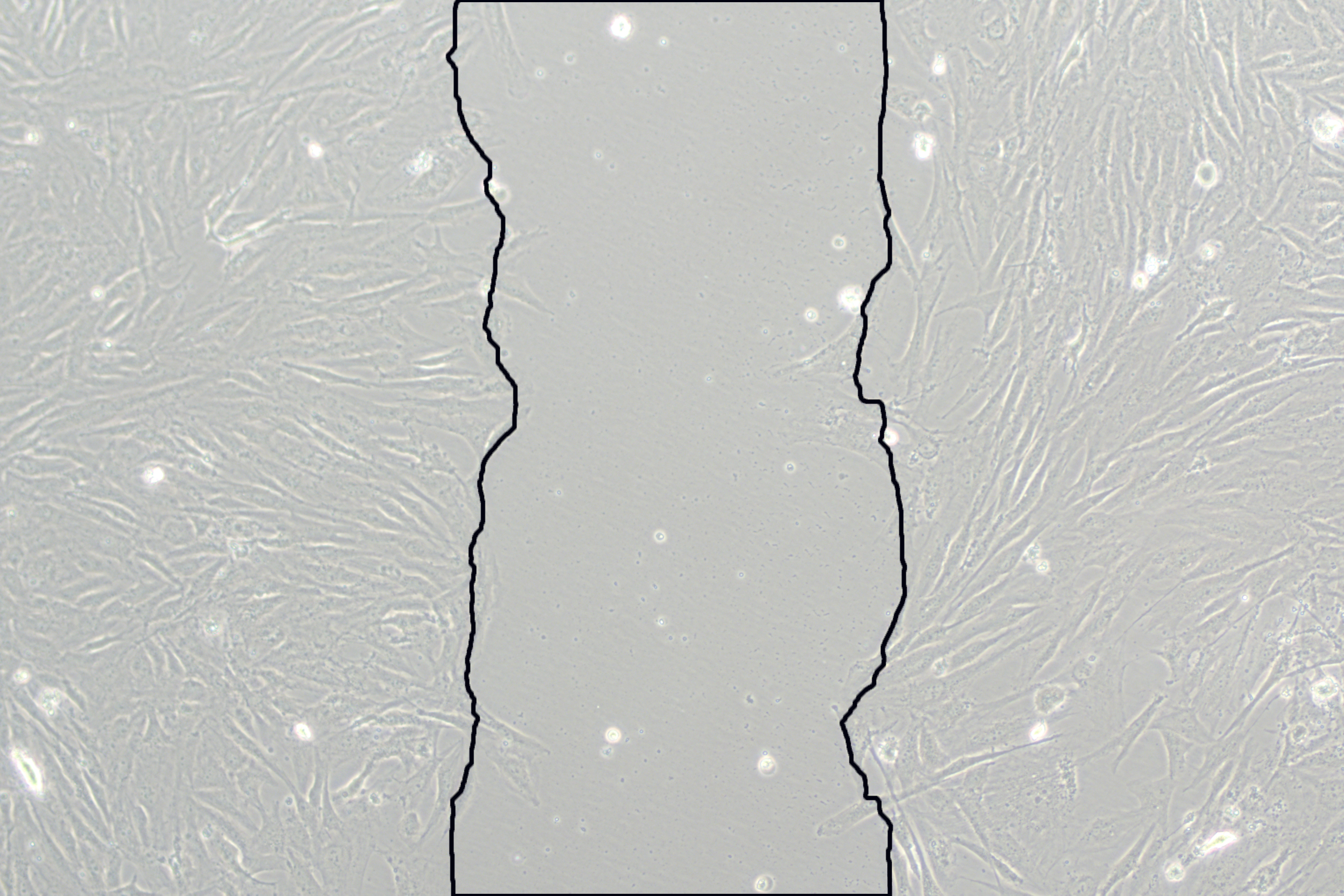

Supplement: S8 File — (ZIP) [file pone.0324264.s008.zip › supplement.material-8/images(Cell Scratch Assay)-HSF-12h/PL20X3.jpg]

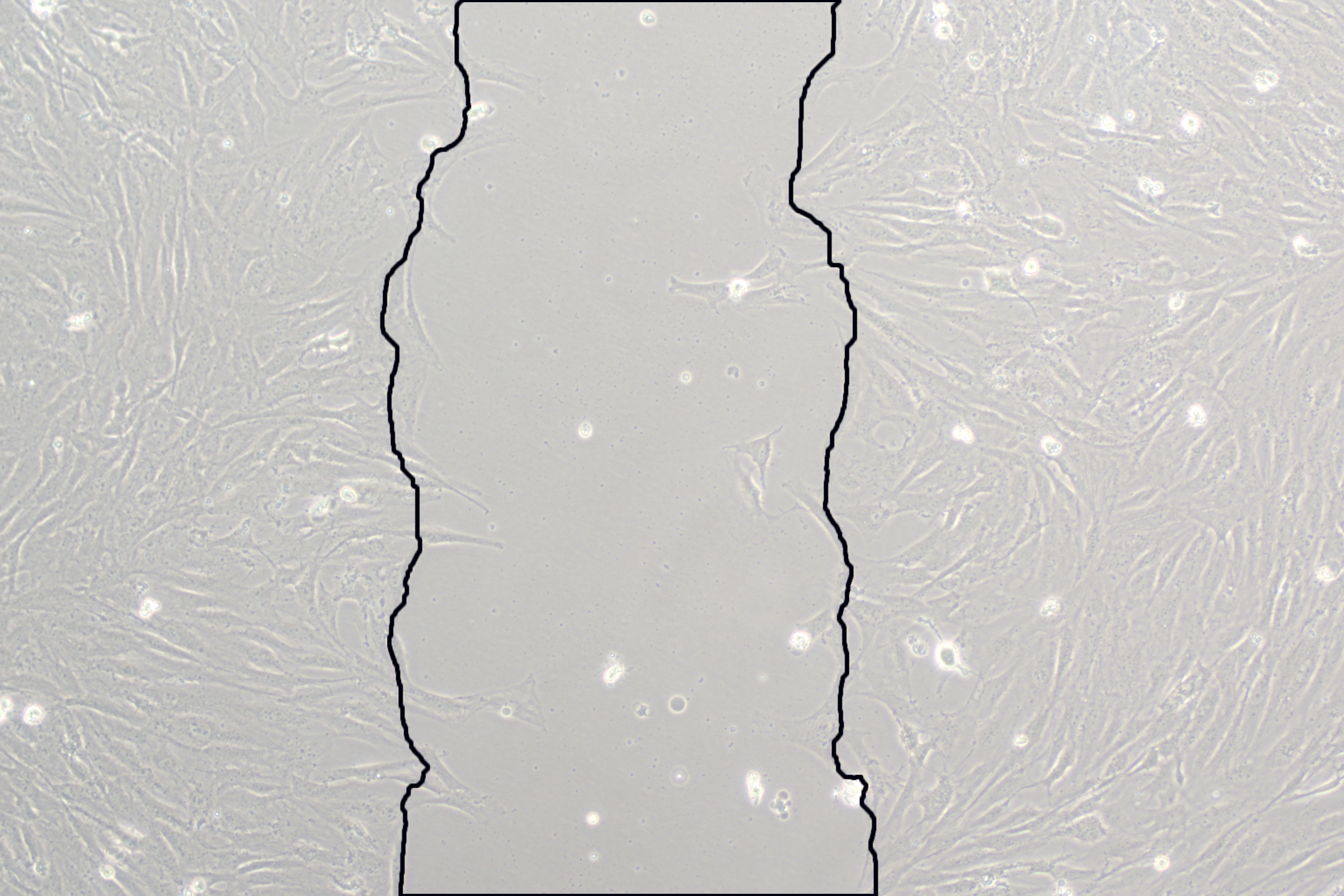

Supplement: S8 File — (ZIP) [file pone.0324264.s008.zip › supplement.material-8/images(Cell Scratch Assay)-HSF-12h/PL20X4.jpg]

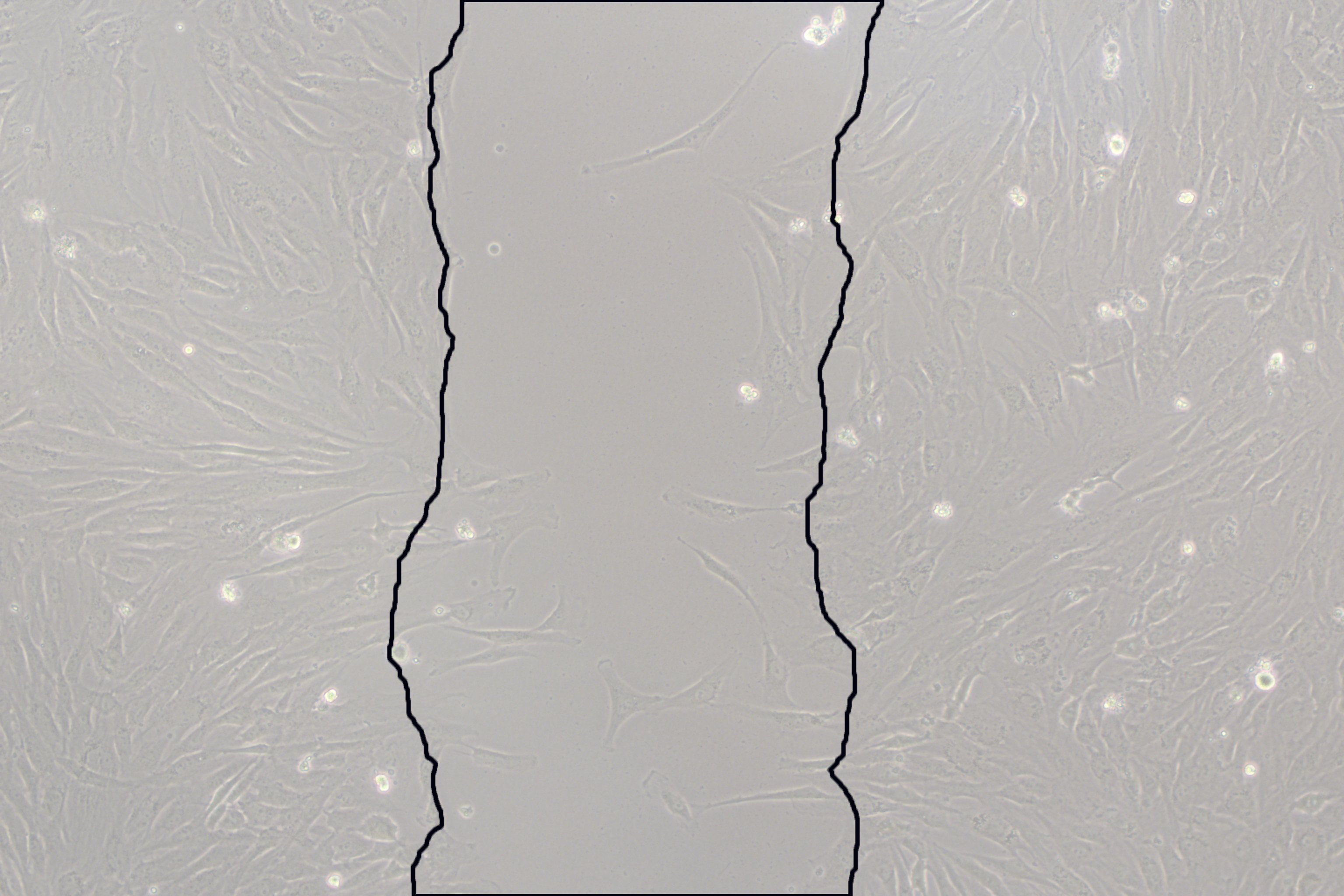

Supplement: S8 File — (ZIP) [file pone.0324264.s008.zip › supplement.material-8/images(Cell Scratch Assay)-HSF-12h/PL20X5.jpg]

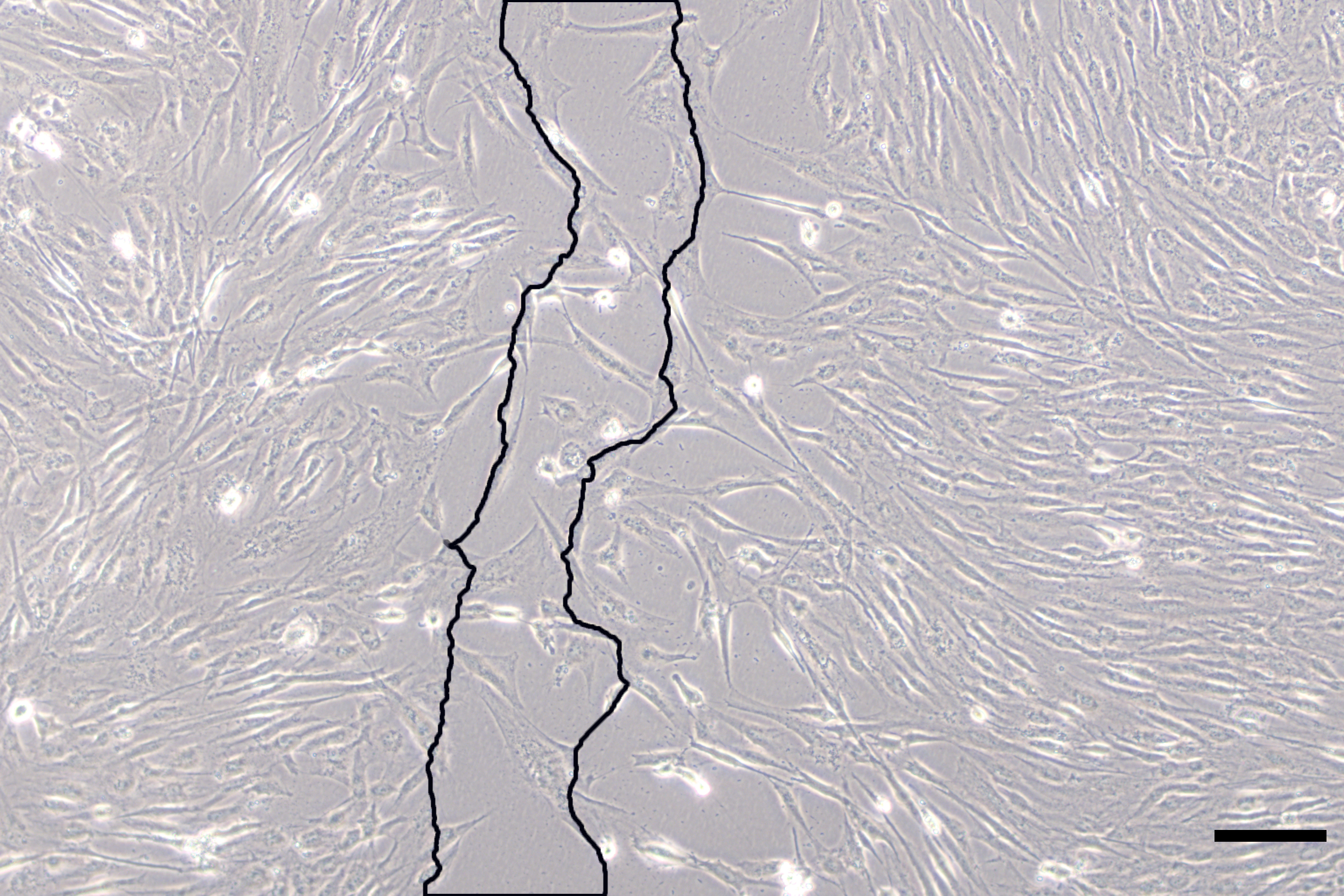

Supplement: S9 File — (ZIP) [file pone.0324264.s009.zip › supplement.material-9/images(Cell Scratch Assay)-HSF-24h/Control1.png]

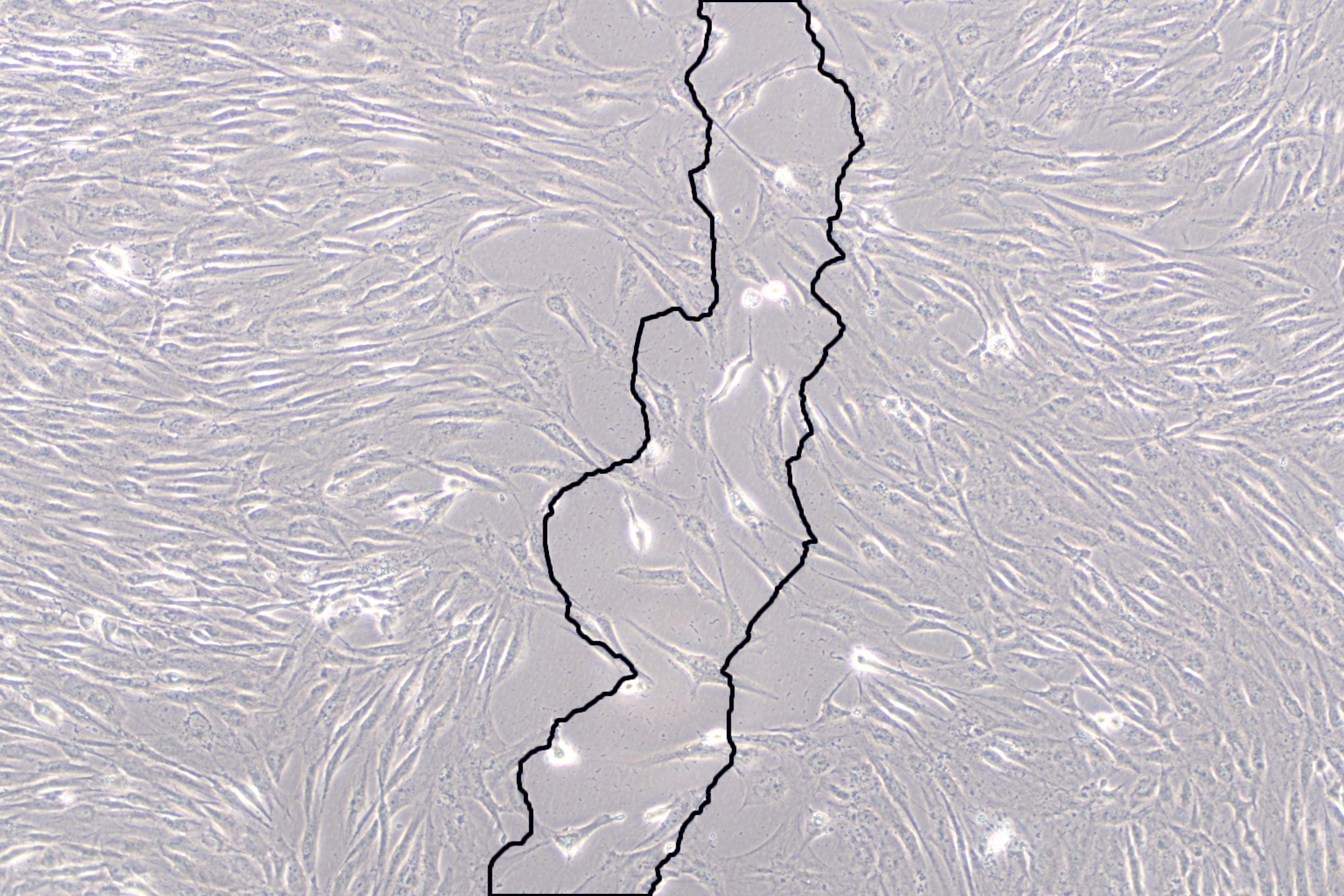

Supplement: S9 File — (ZIP) [file pone.0324264.s009.zip › supplement.material-9/images(Cell Scratch Assay)-HSF-24h/Control2.jpg]

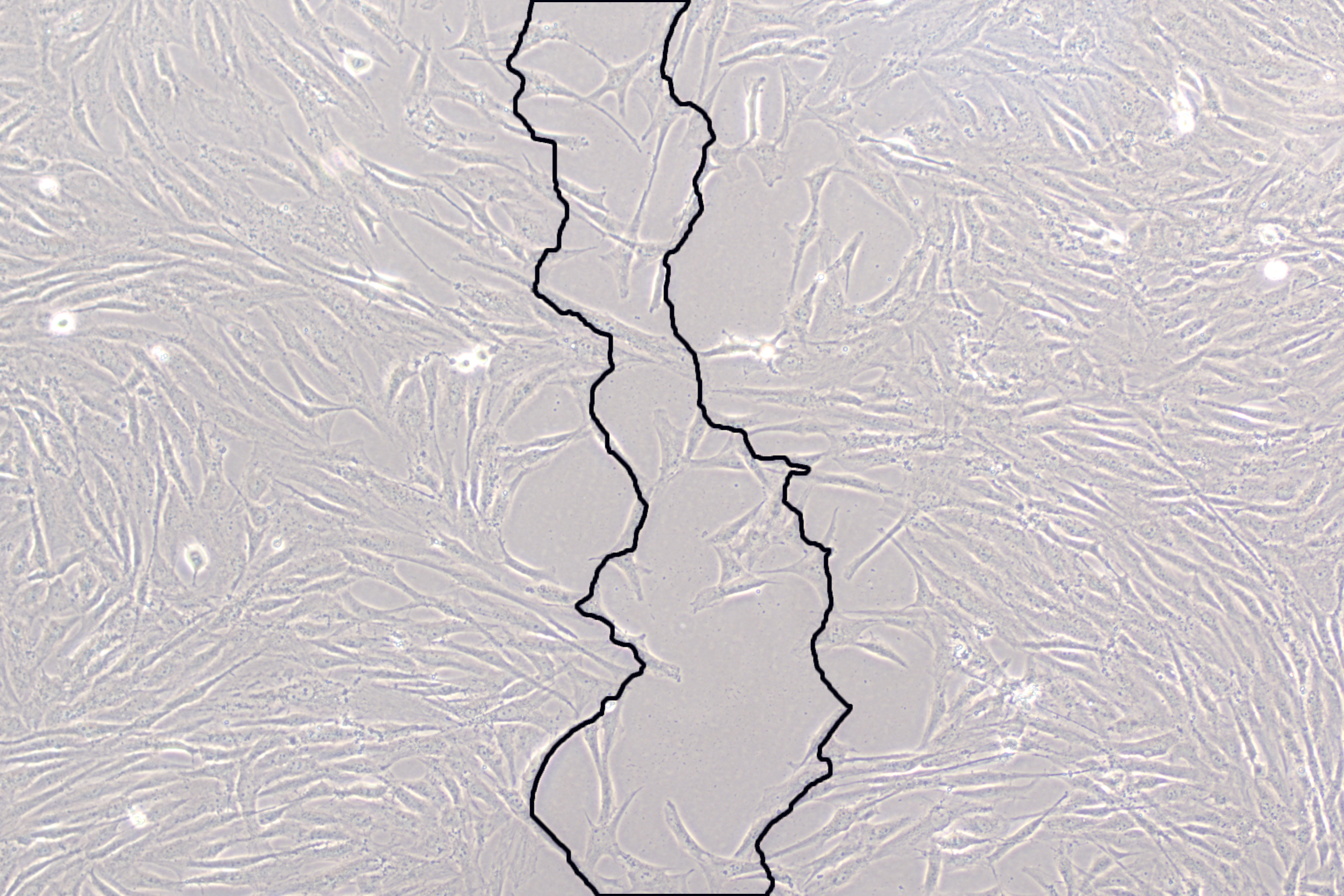

Supplement: S9 File — (ZIP) [file pone.0324264.s009.zip › supplement.material-9/images(Cell Scratch Assay)-HSF-24h/Control3.jpg]

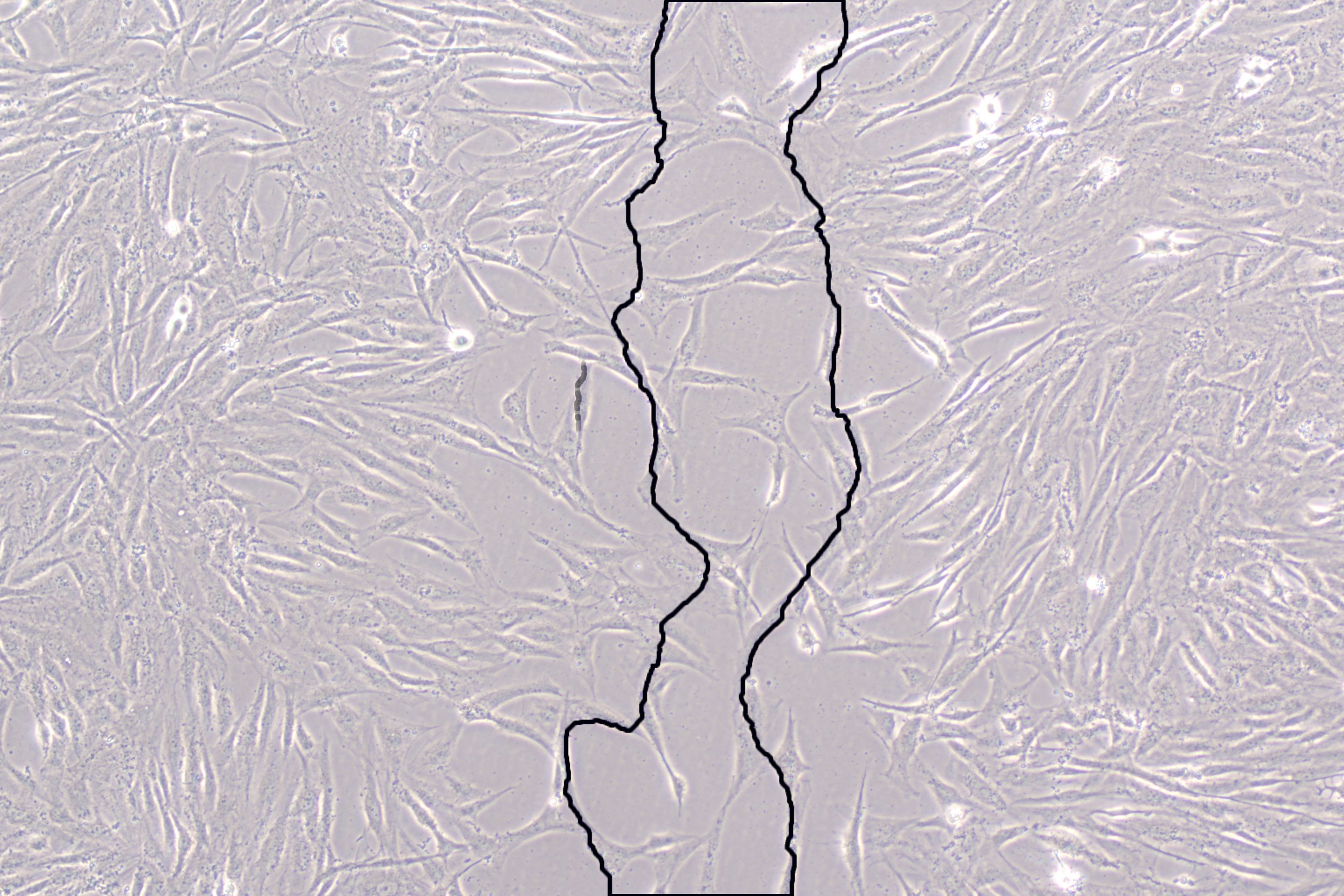

Supplement: S9 File — (ZIP) [file pone.0324264.s009.zip › supplement.material-9/images(Cell Scratch Assay)-HSF-24h/Control4.jpg]

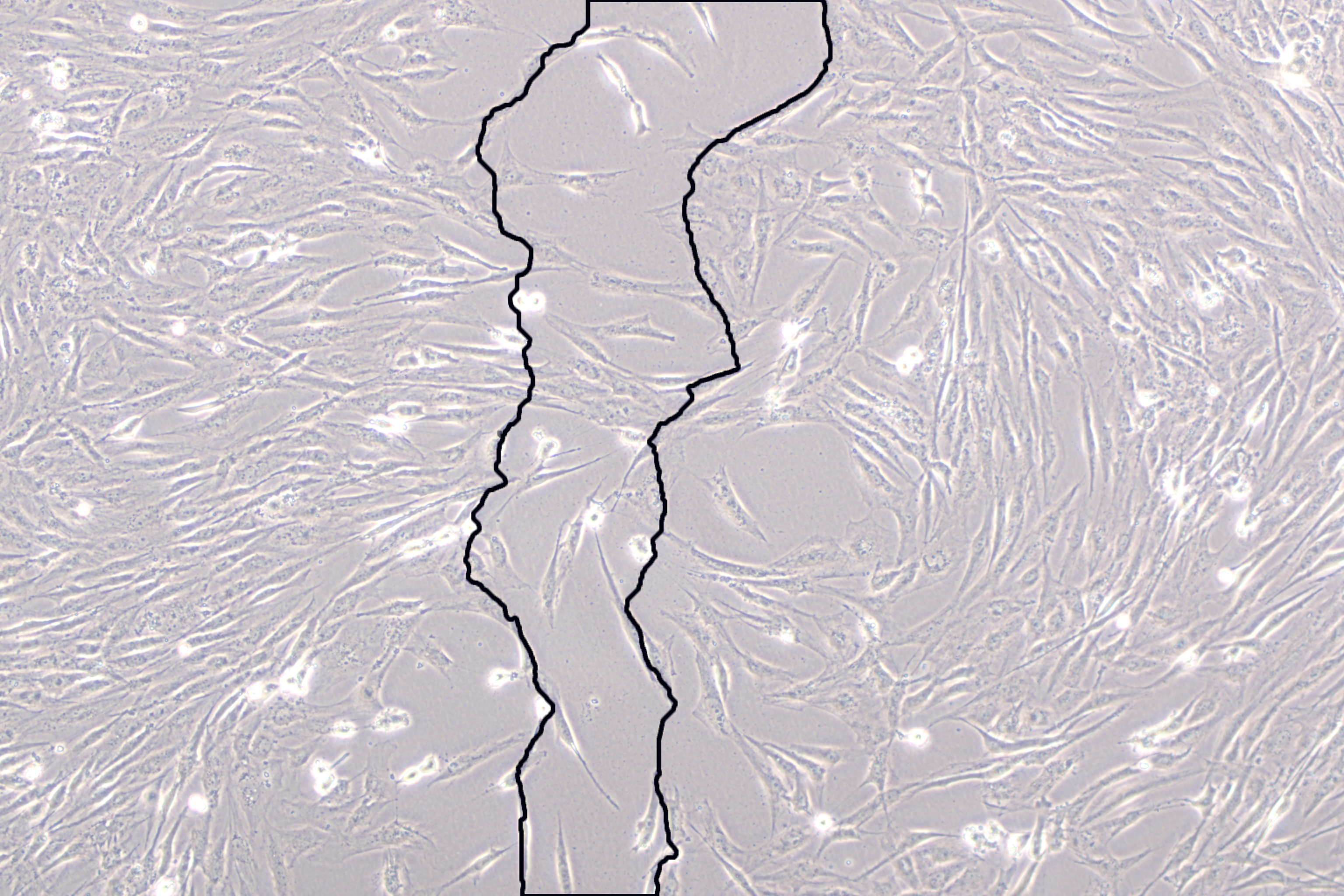

Supplement: S9 File — (ZIP) [file pone.0324264.s009.zip › supplement.material-9/images(Cell Scratch Assay)-HSF-24h/Control5.jpg]

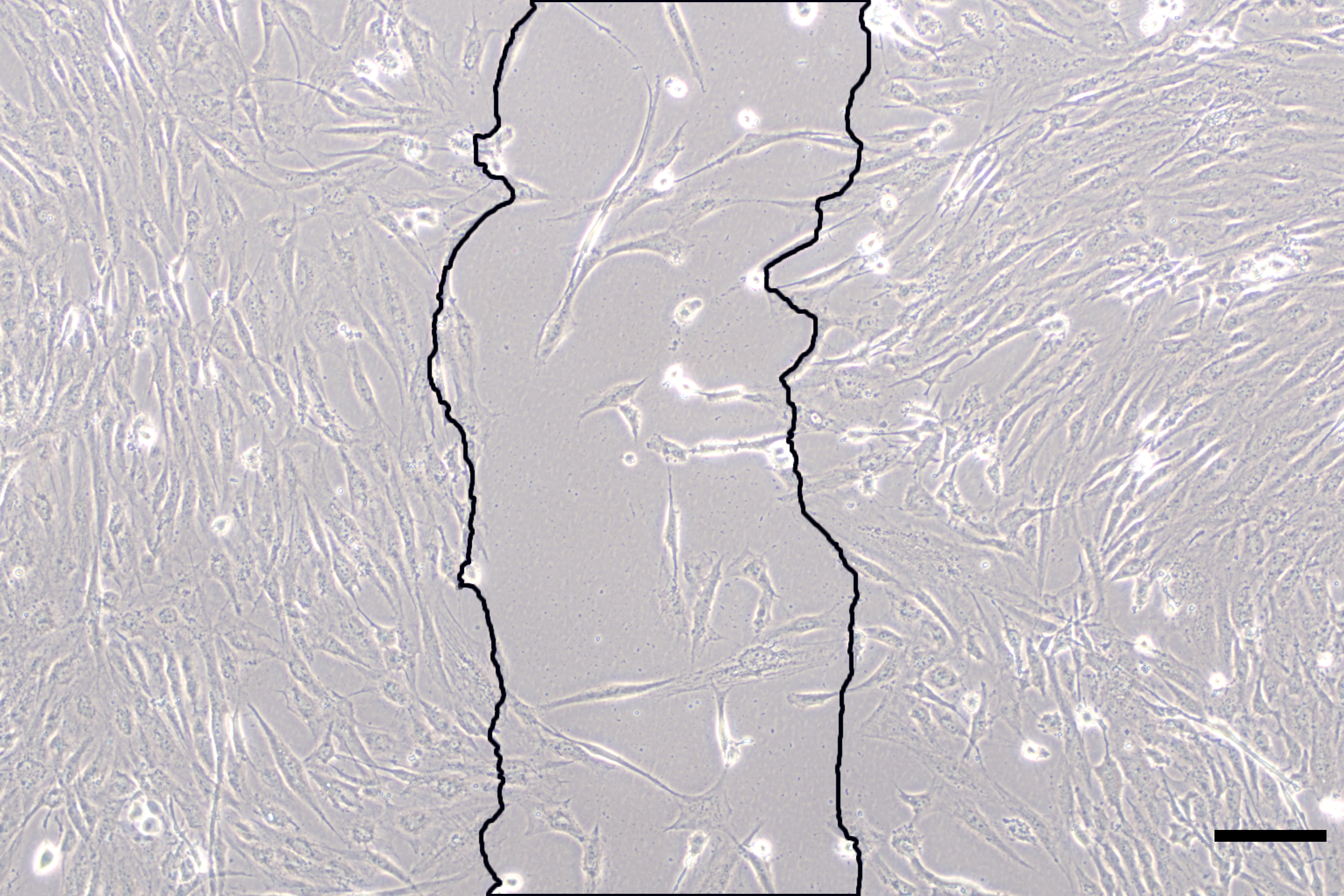

Supplement: S9 File — (ZIP) [file pone.0324264.s009.zip › supplement.material-9/images(Cell Scratch Assay)-HSF-24h/Model1.png]

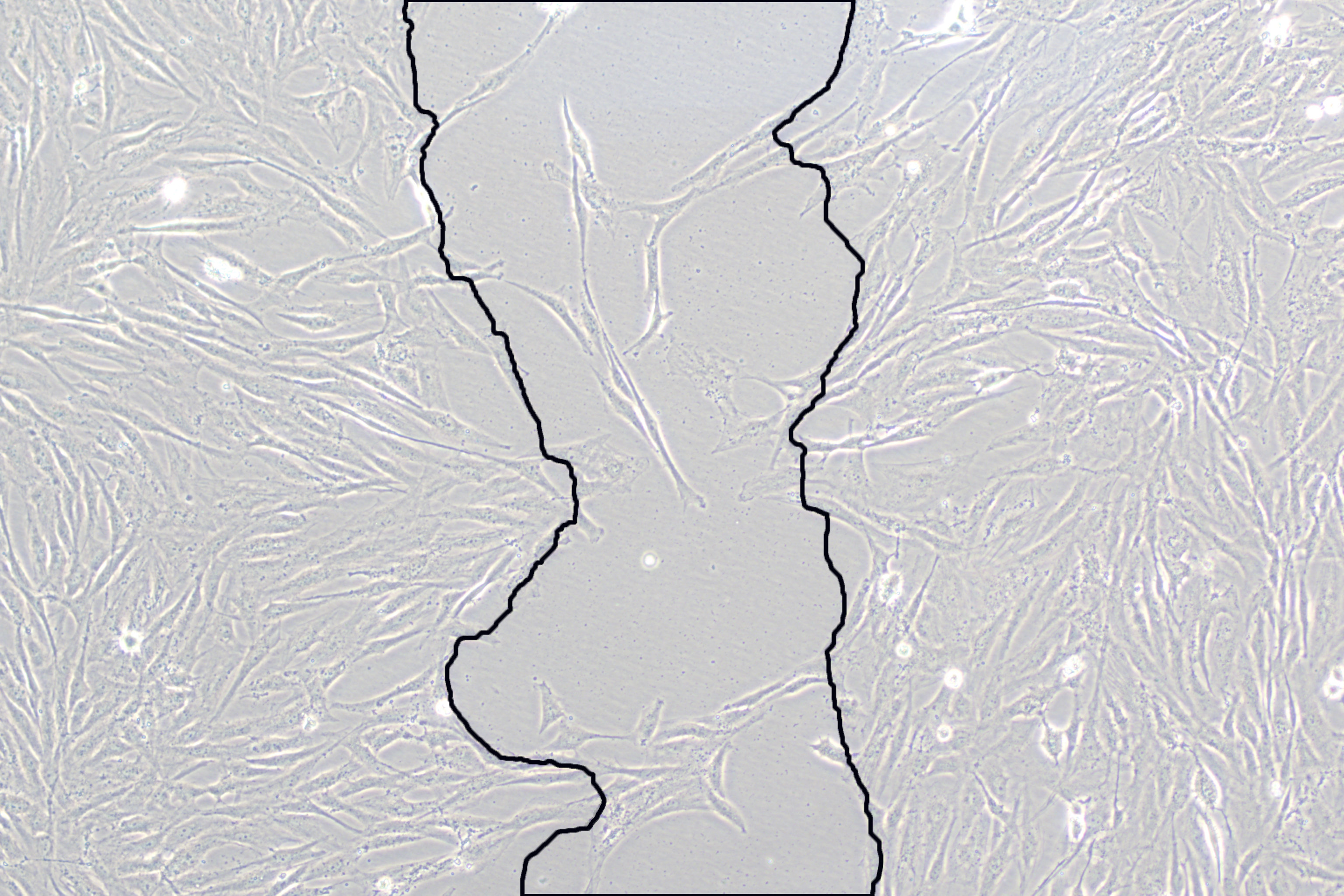

Supplement: S9 File — (ZIP) [file pone.0324264.s009.zip › supplement.material-9/images(Cell Scratch Assay)-HSF-24h/Model2.jpg]

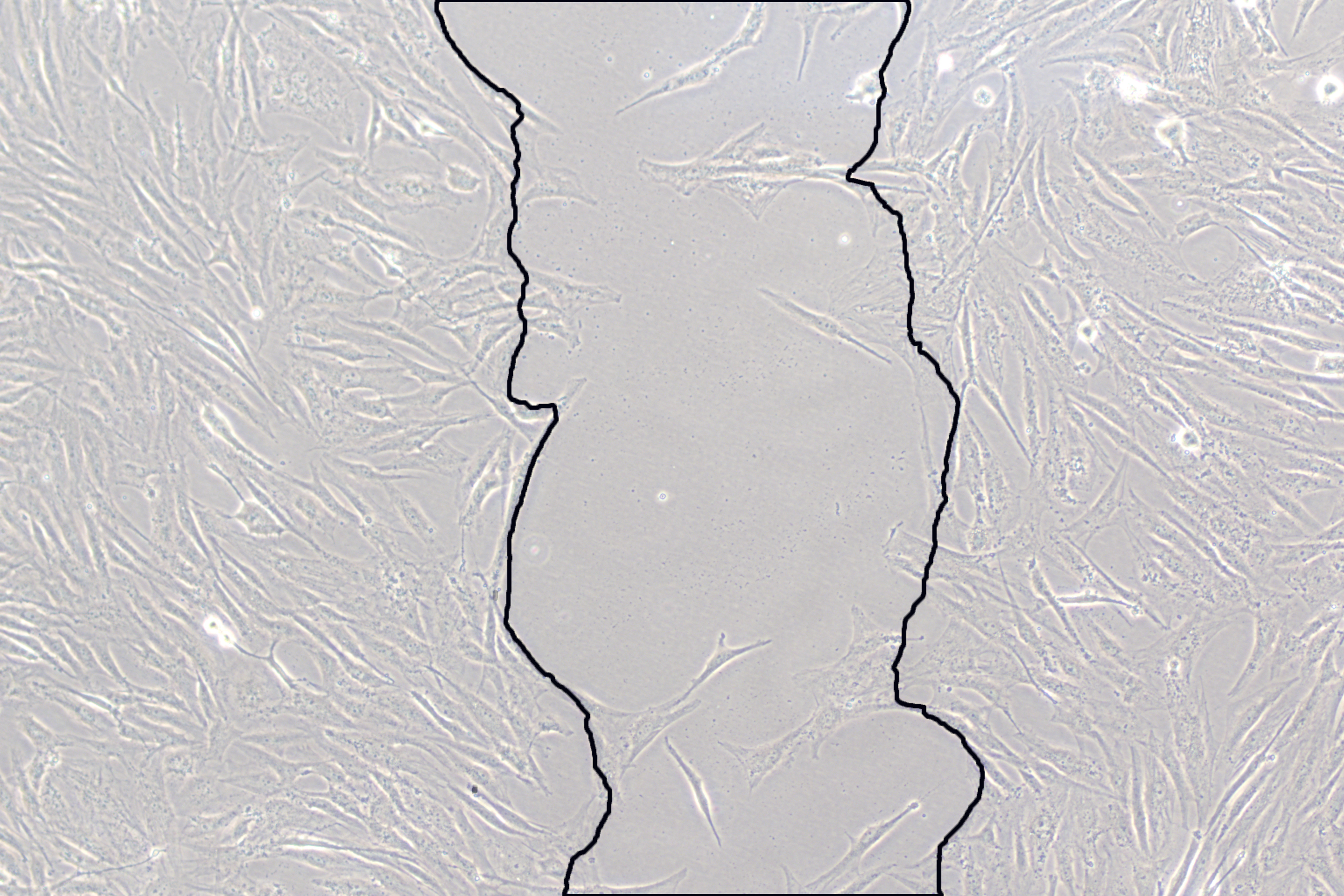

Supplement: S9 File — (ZIP) [file pone.0324264.s009.zip › supplement.material-9/images(Cell Scratch Assay)-HSF-24h/Model3.jpg]

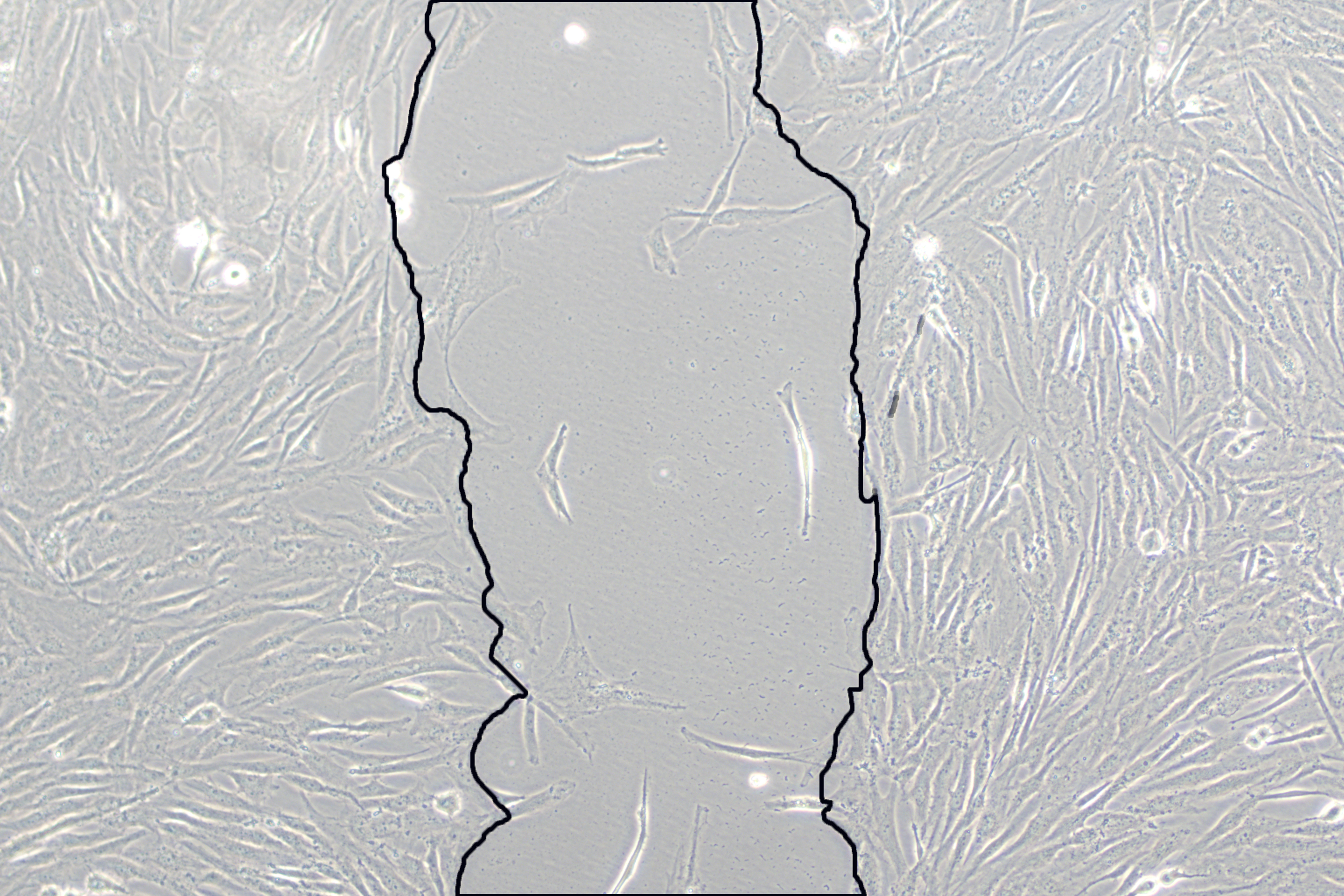

Supplement: S9 File — (ZIP) [file pone.0324264.s009.zip › supplement.material-9/images(Cell Scratch Assay)-HSF-24h/Model4.jpg]

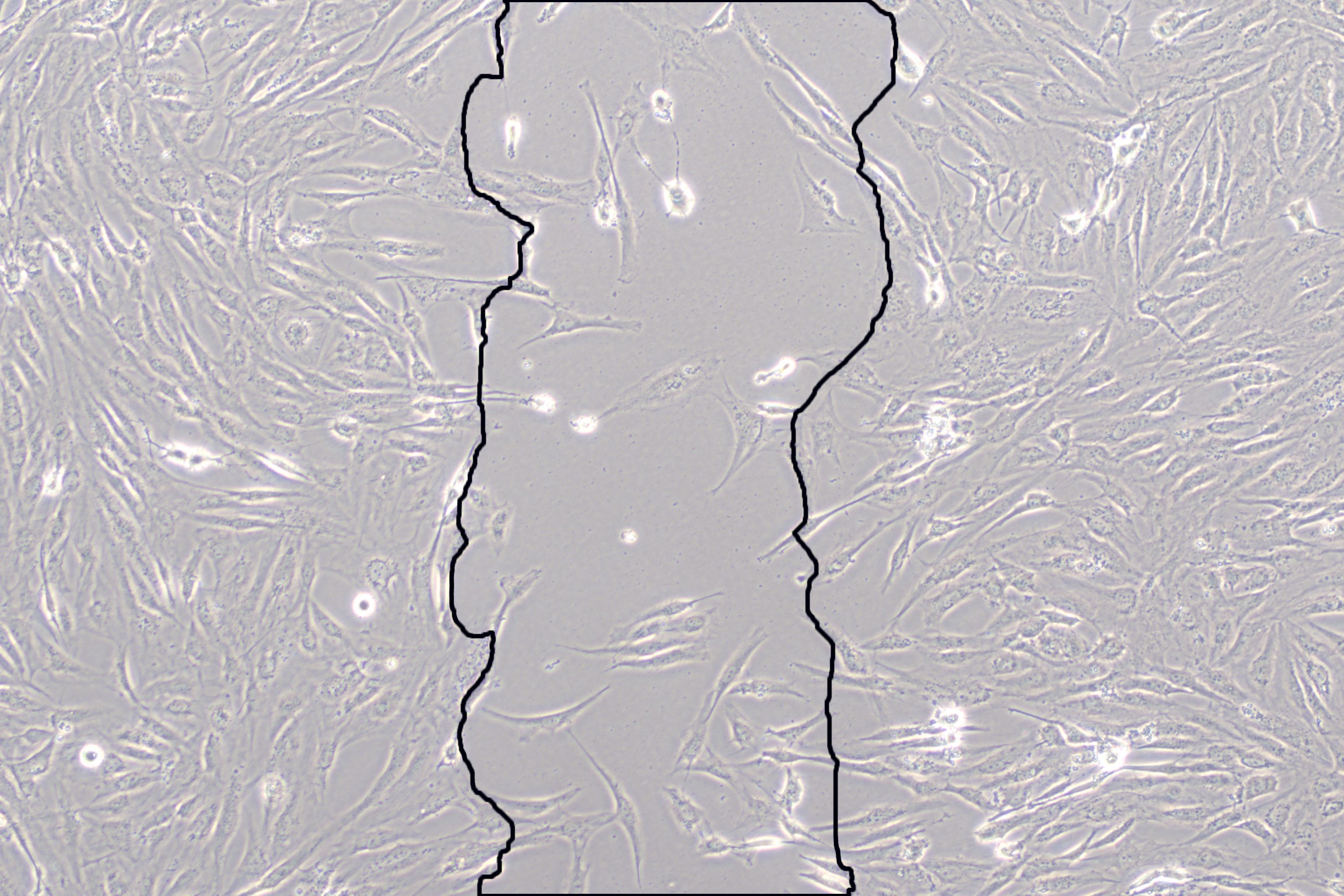

Supplement: S9 File — (ZIP) [file pone.0324264.s009.zip › supplement.material-9/images(Cell Scratch Assay)-HSF-24h/Model5.jpg]

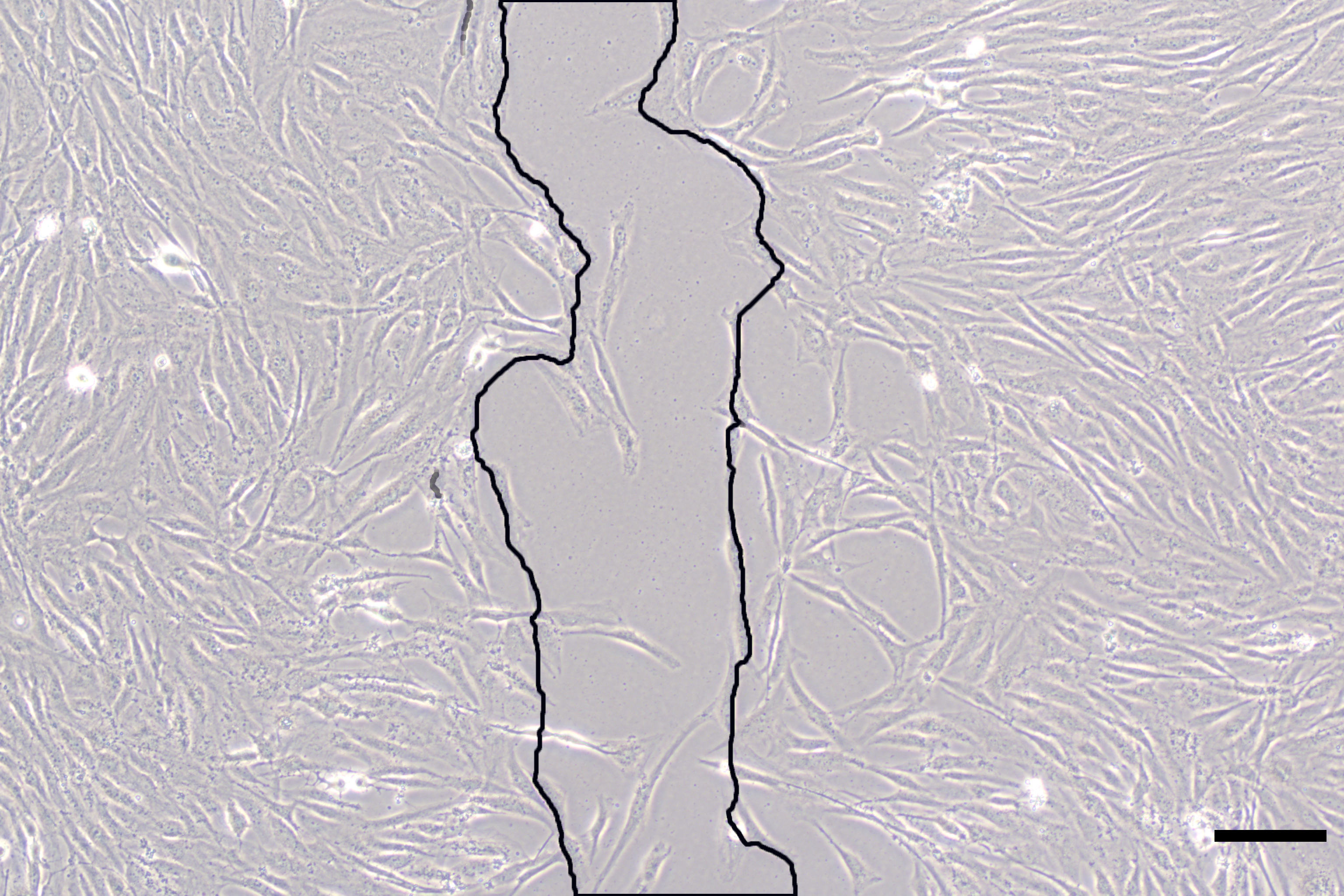

Supplement: S9 File — (ZIP) [file pone.0324264.s009.zip › supplement.material-9/images(Cell Scratch Assay)-HSF-24h/PL10X1.png]

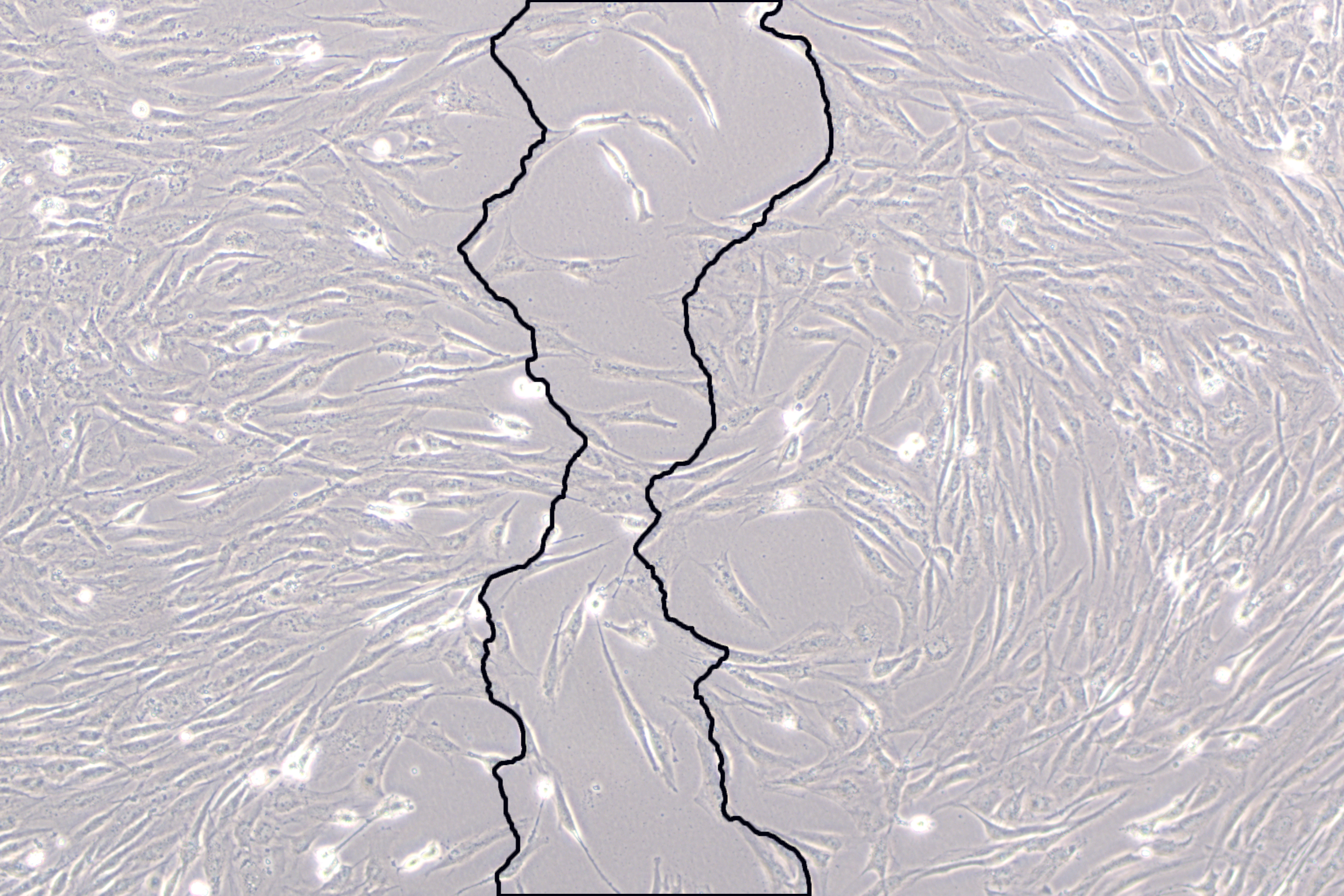

Supplement: S9 File — (ZIP) [file pone.0324264.s009.zip › supplement.material-9/images(Cell Scratch Assay)-HSF-24h/PL10X2.jpg]

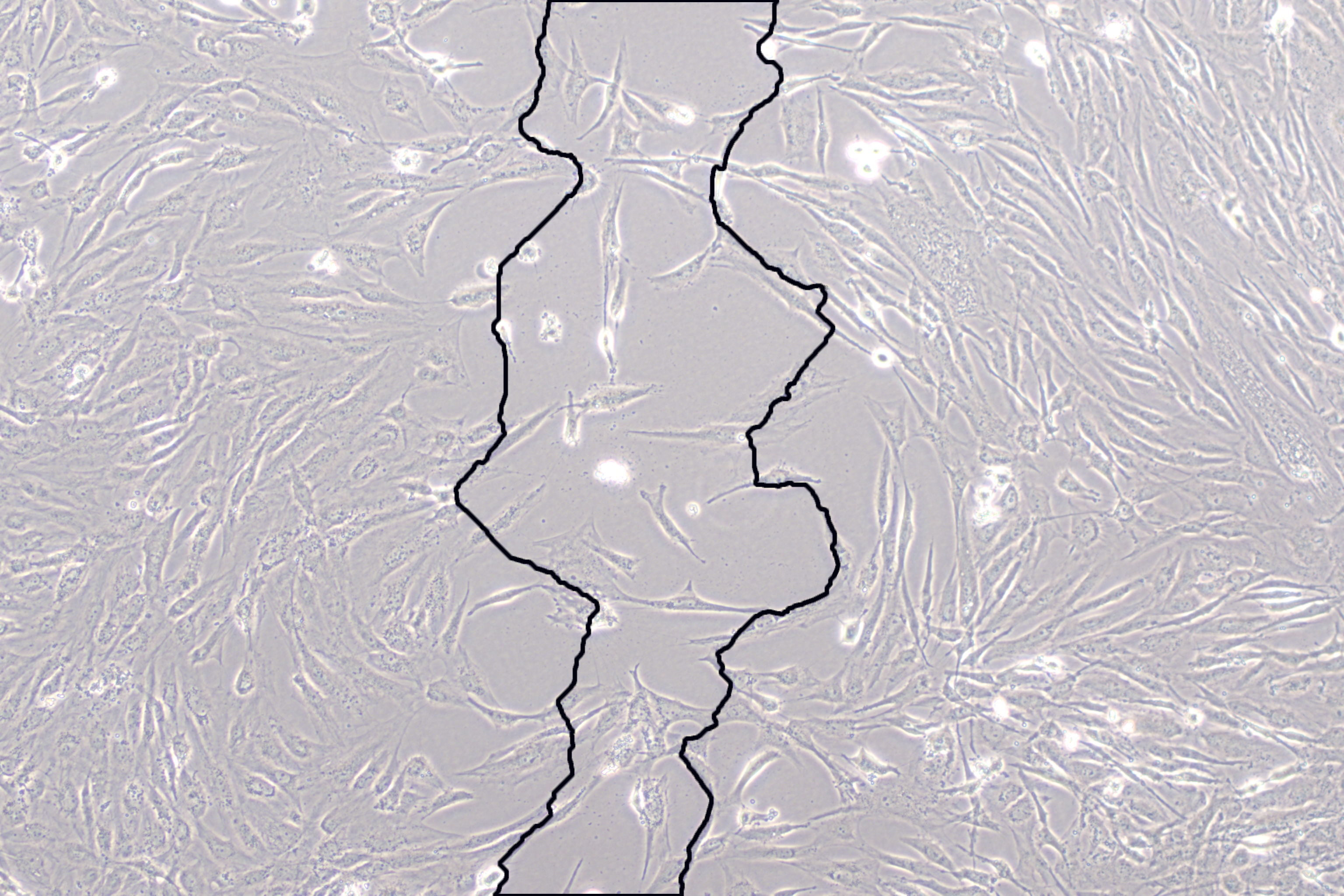

Supplement: S9 File — (ZIP) [file pone.0324264.s009.zip › supplement.material-9/images(Cell Scratch Assay)-HSF-24h/PL10X3.jpg]

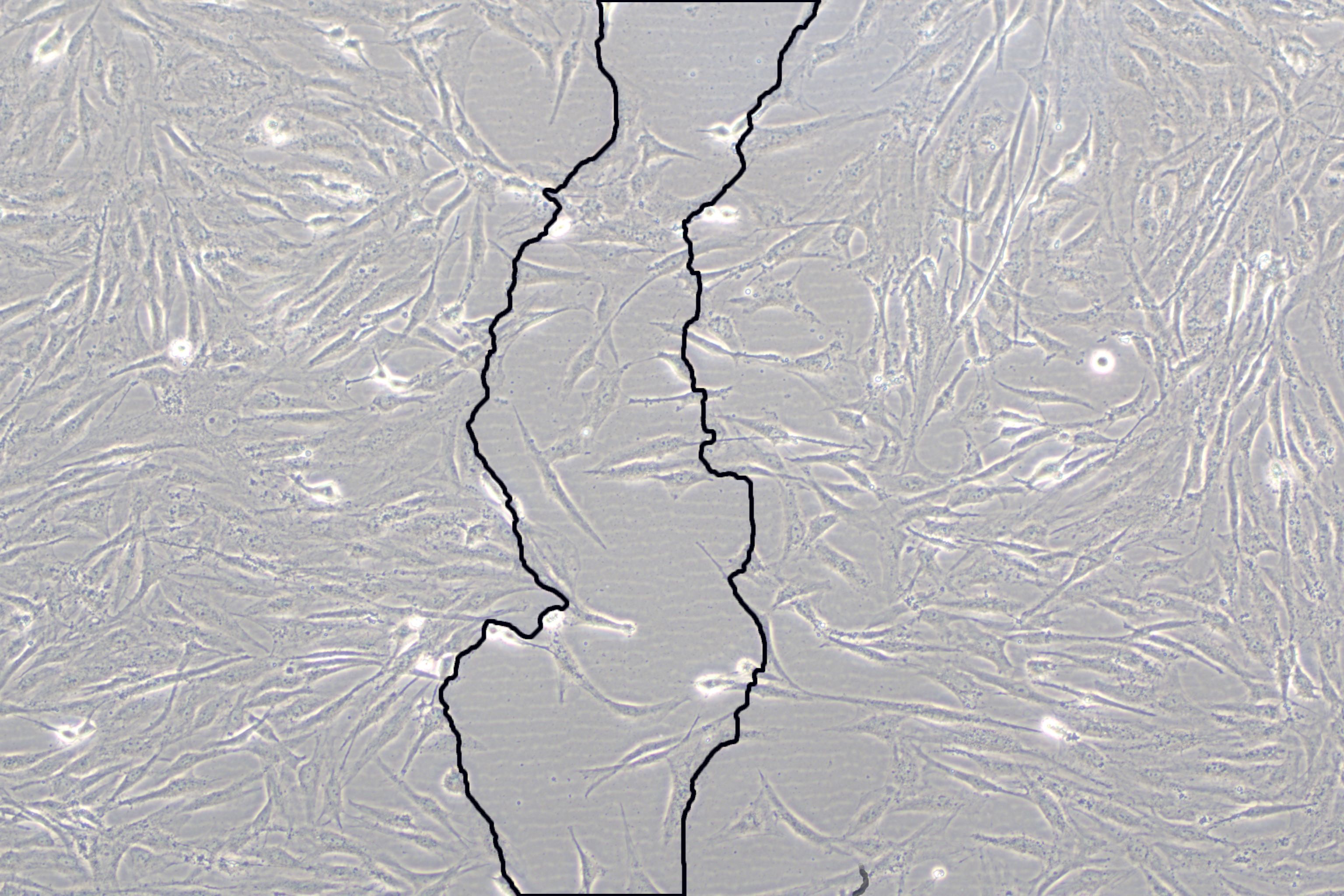

Supplement: S9 File — (ZIP) [file pone.0324264.s009.zip › supplement.material-9/images(Cell Scratch Assay)-HSF-24h/PL10X4.jpg]

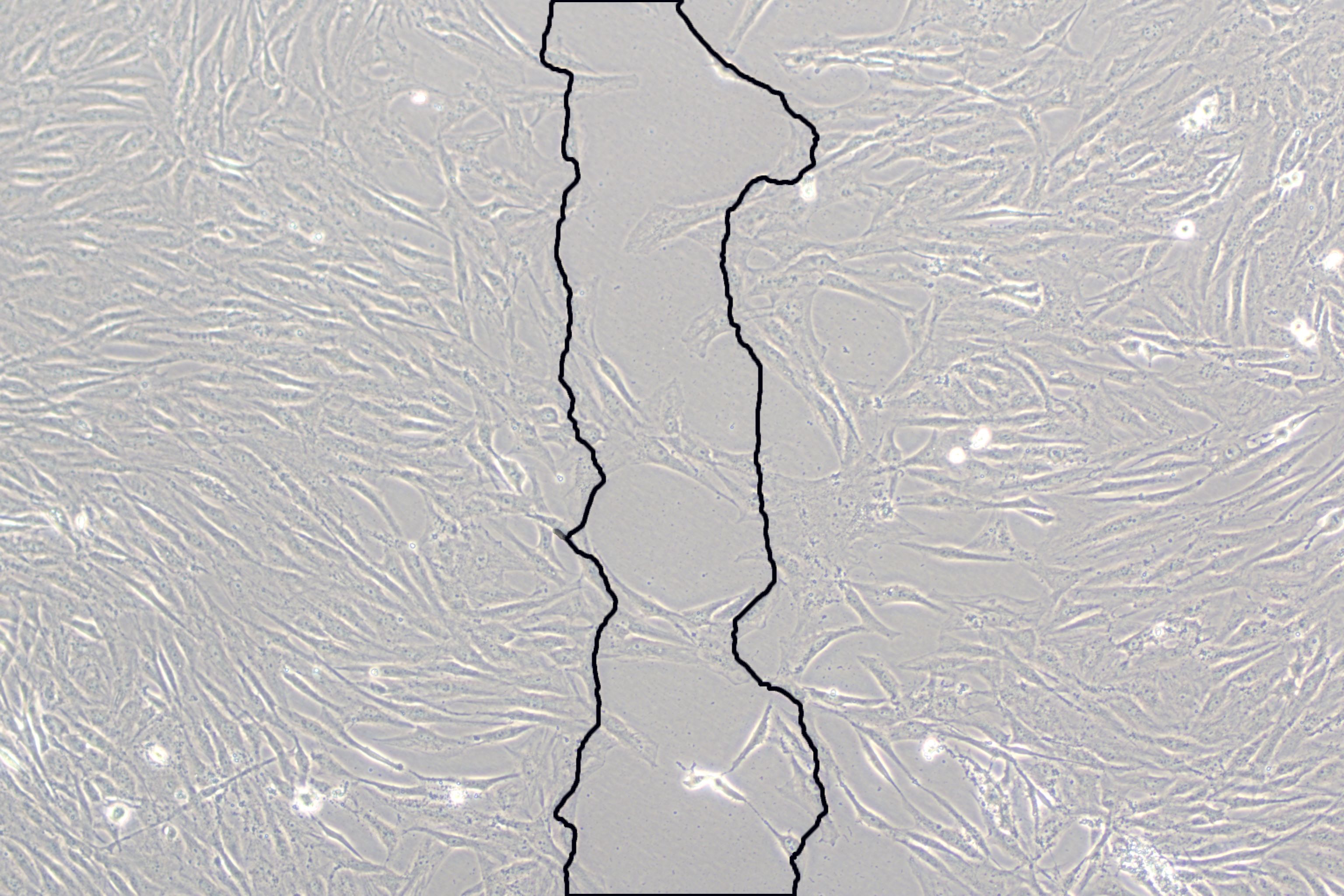

Supplement: S9 File — (ZIP) [file pone.0324264.s009.zip › supplement.material-9/images(Cell Scratch Assay)-HSF-24h/PL10X5.jpg]

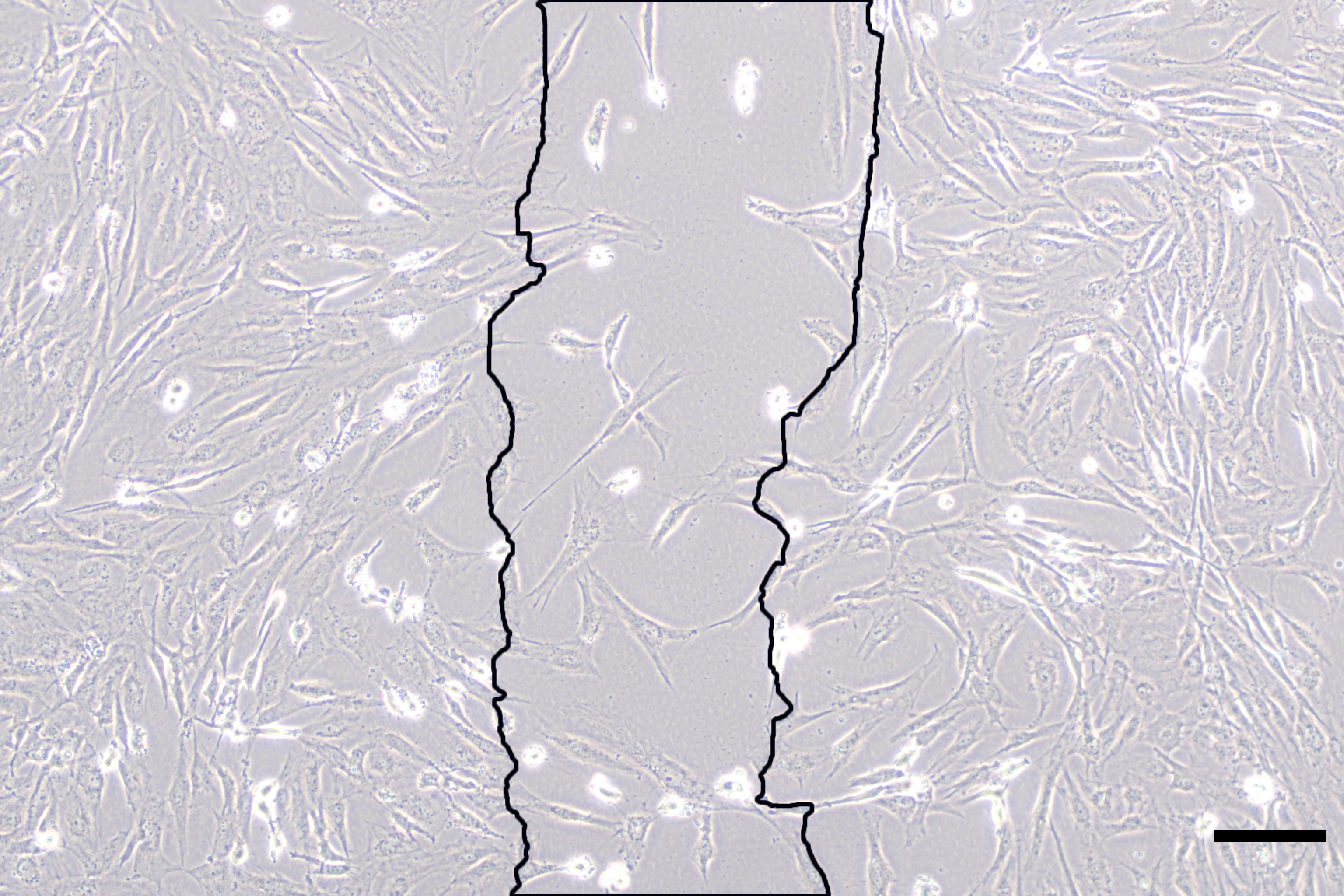

Supplement: S9 File — (ZIP) [file pone.0324264.s009.zip › supplement.material-9/images(Cell Scratch Assay)-HSF-24h/PL20X1.png]

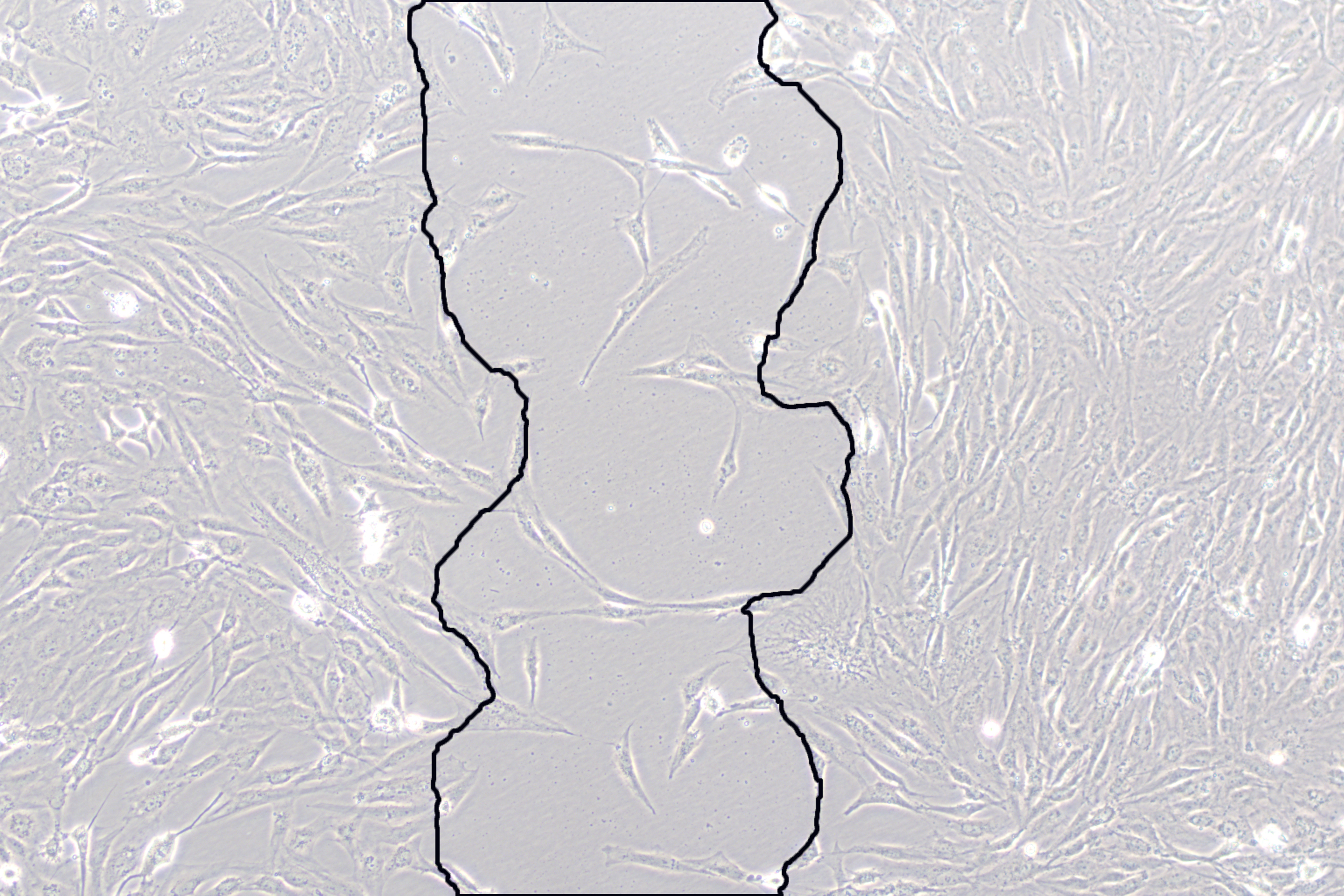

Supplement: S9 File — (ZIP) [file pone.0324264.s009.zip › supplement.material-9/images(Cell Scratch Assay)-HSF-24h/PL20X2.jpg]

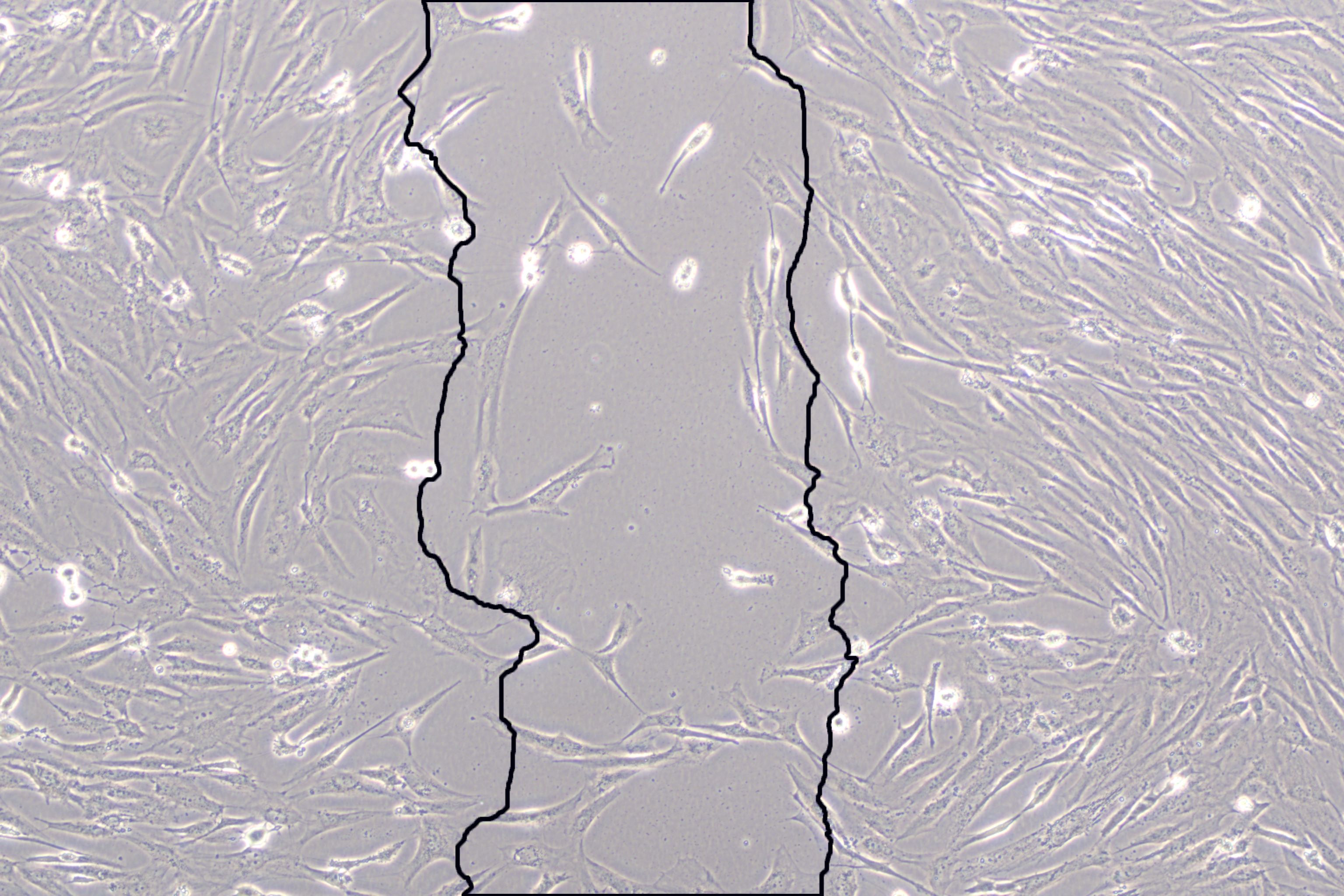

Supplement: S9 File — (ZIP) [file pone.0324264.s009.zip › supplement.material-9/images(Cell Scratch Assay)-HSF-24h/PL20X3.jpg]

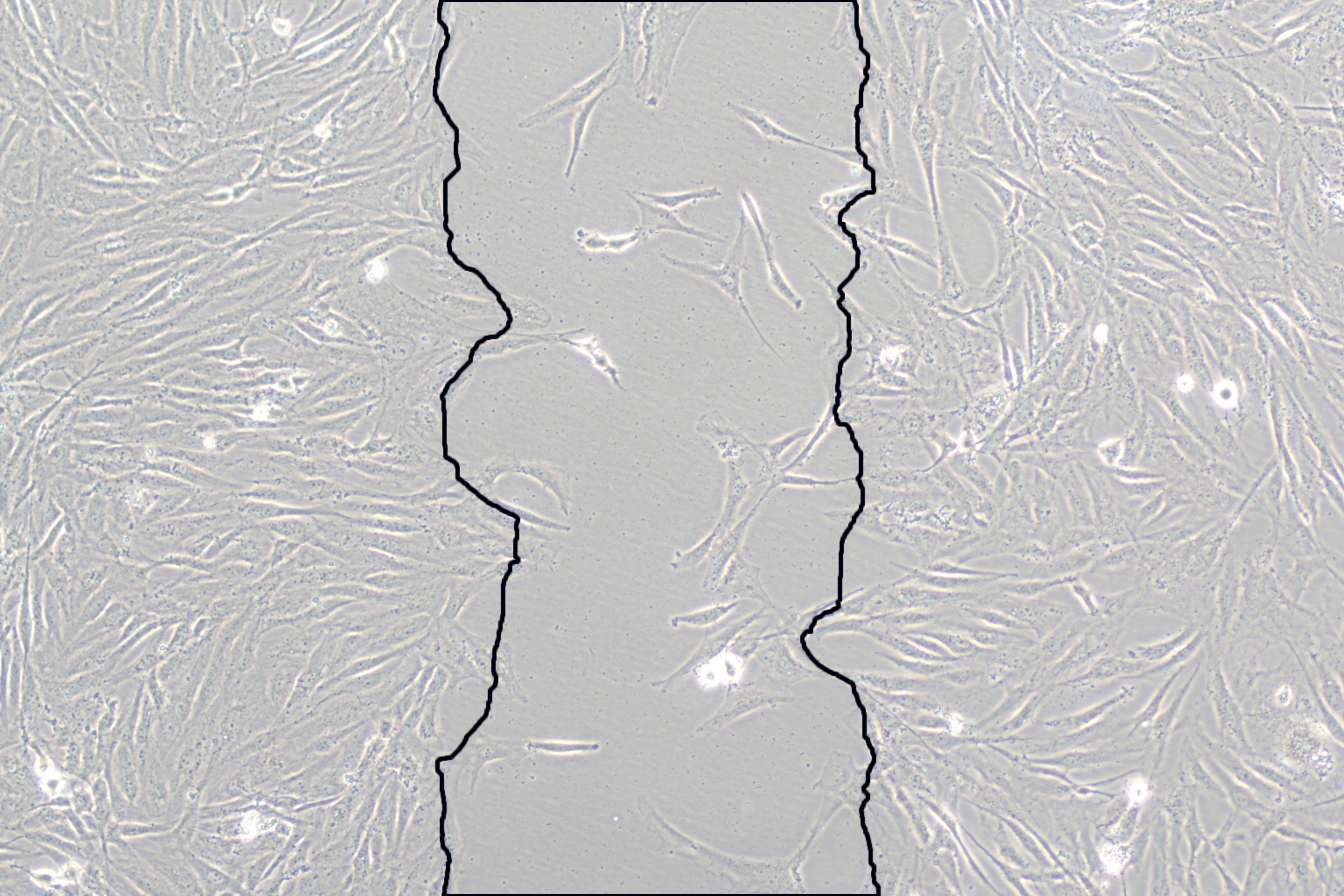

Supplement: S9 File — (ZIP) [file pone.0324264.s009.zip › supplement.material-9/images(Cell Scratch Assay)-HSF-24h/PL20X4.jpg]

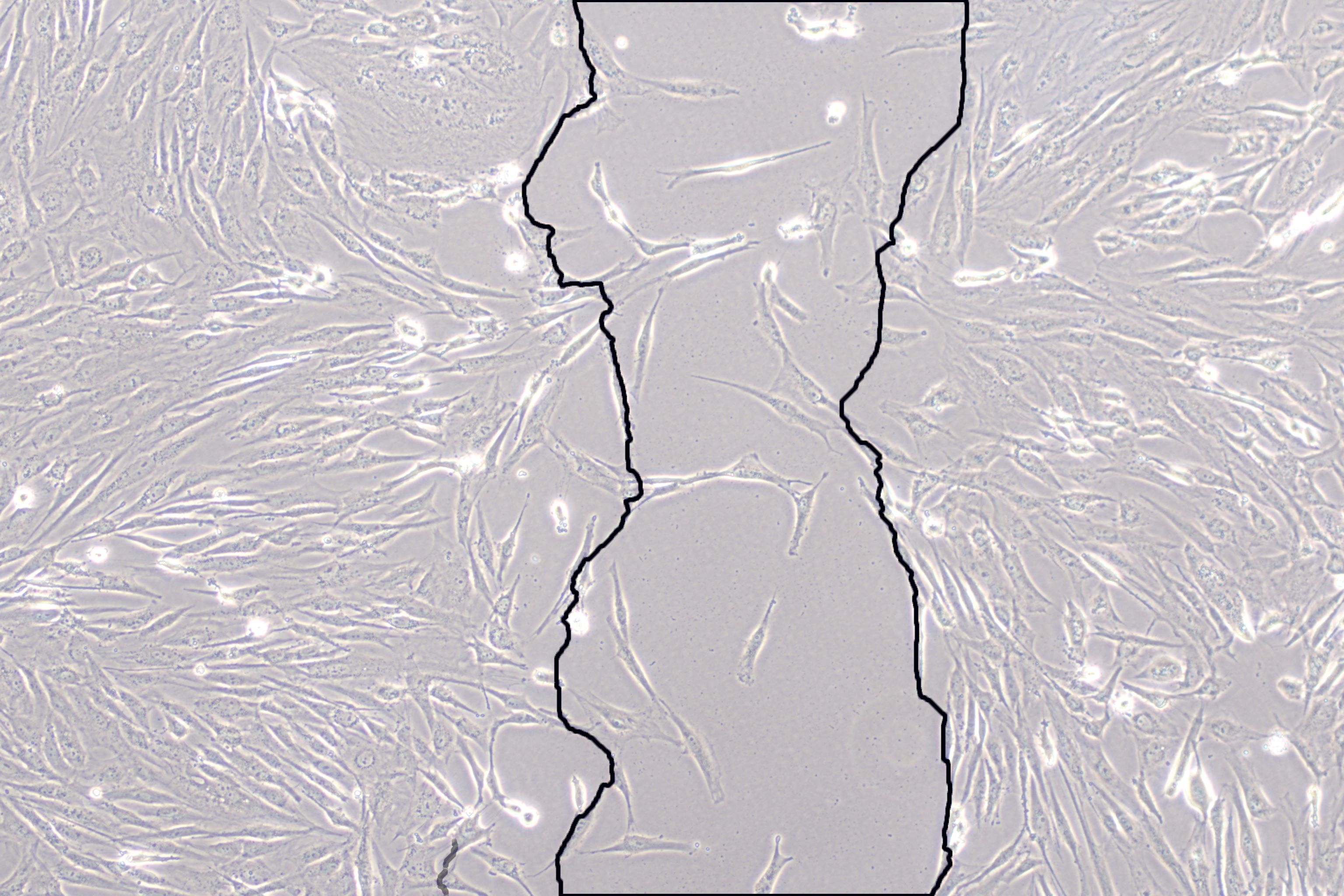

Supplement: S9 File — (ZIP) [file pone.0324264.s009.zip › supplement.material-9/images(Cell Scratch Assay)-HSF-24h/PL20X5.jpg]
