# Supplementary material for: Platelet lysate-sodium hyaluronate gel promotes diabetic foot wound healing by regulating oxidative stress and autophagy
Source: PLoS One. 2025 Jun 6;20(6):e0324264. doi: 10.1371/journal.pone.0324264 (PMC12143543; doi:10.1371/journal.pone.0324264)

Original strips——1

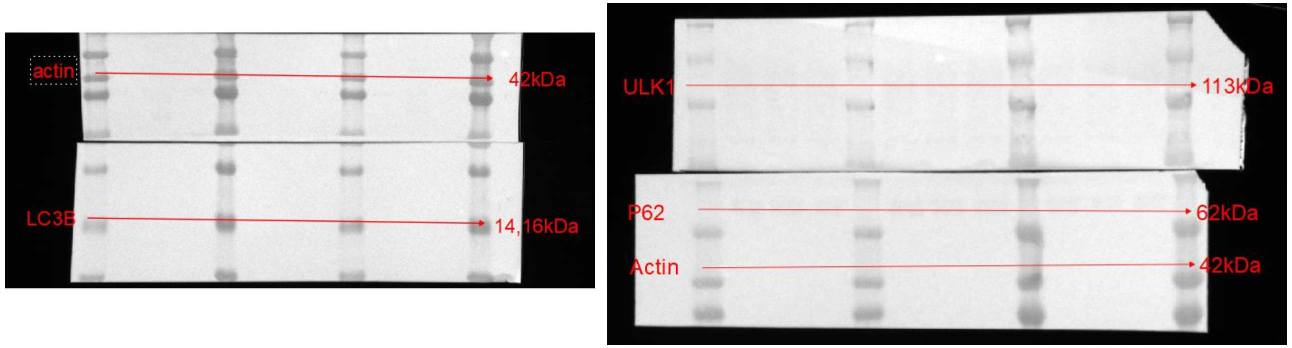

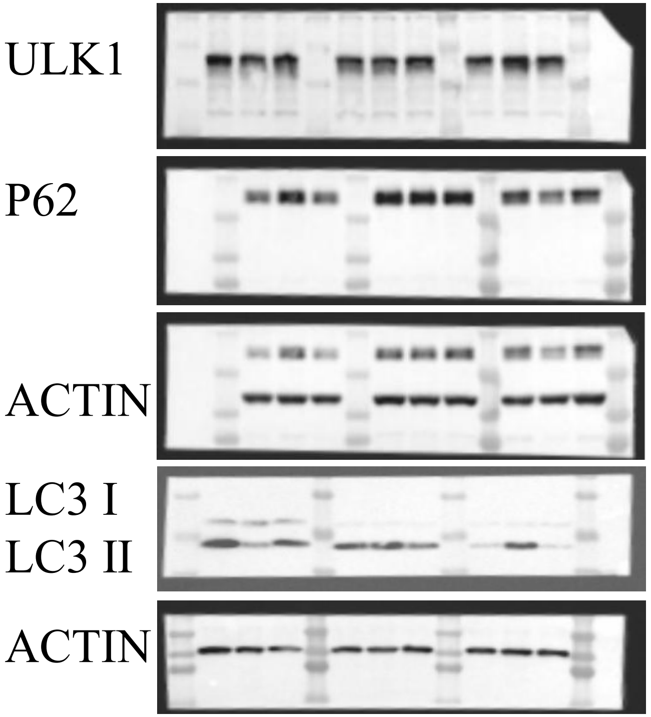

Processing strips——1

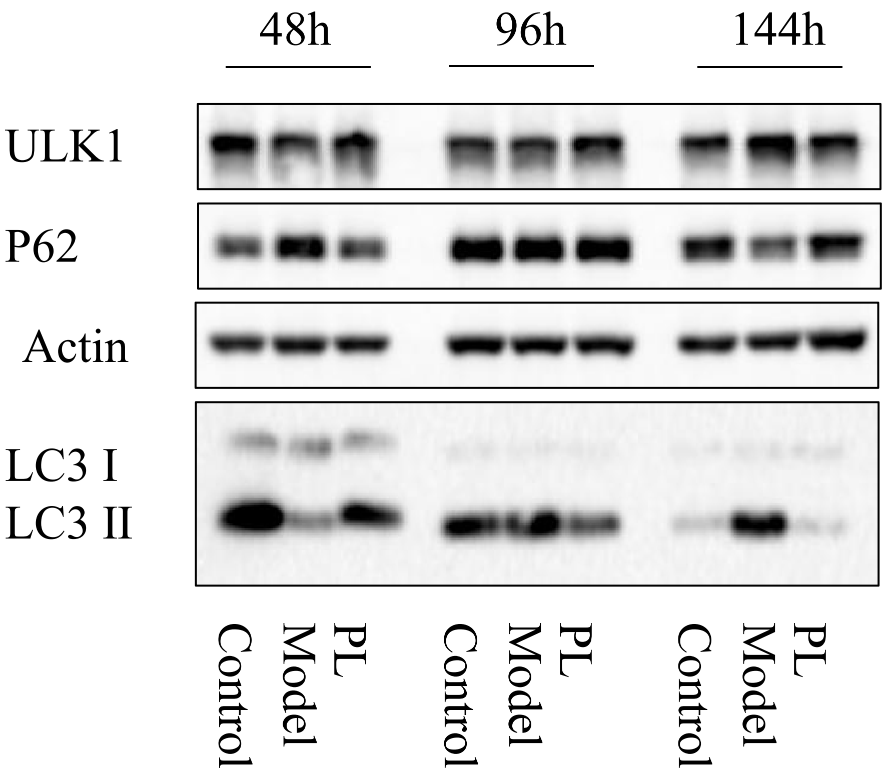

Original strips——2

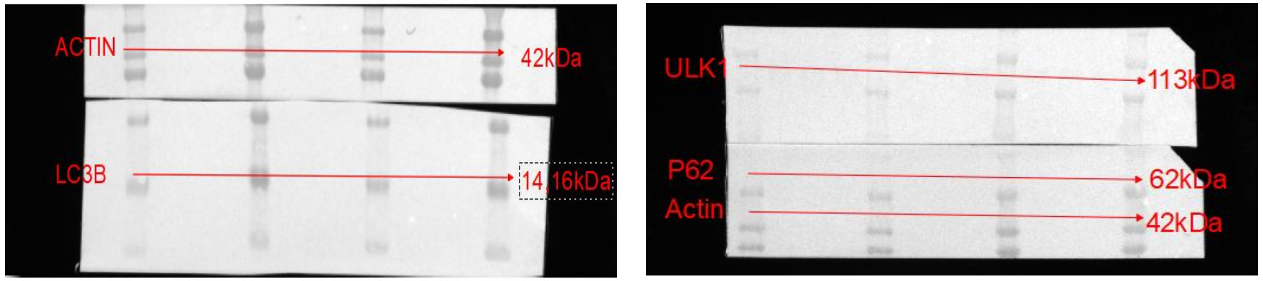

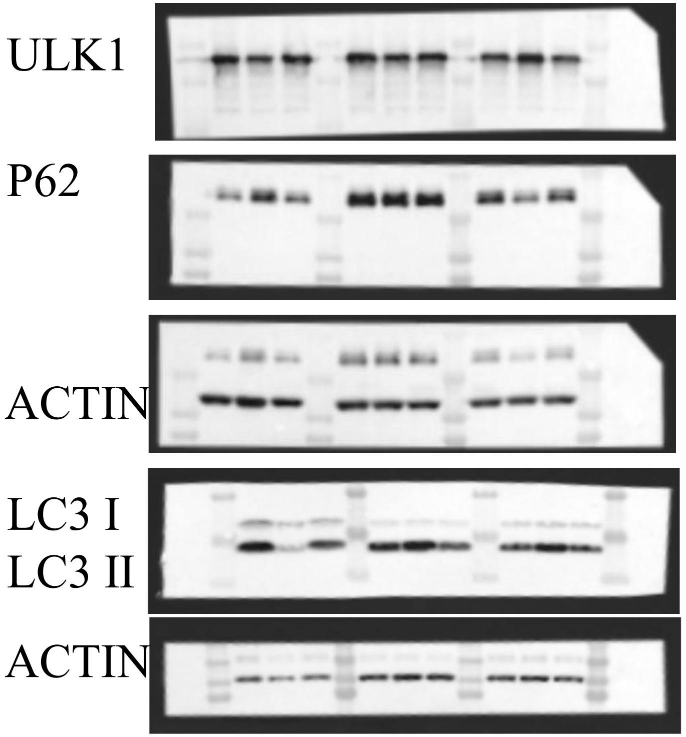

Processing strips——2

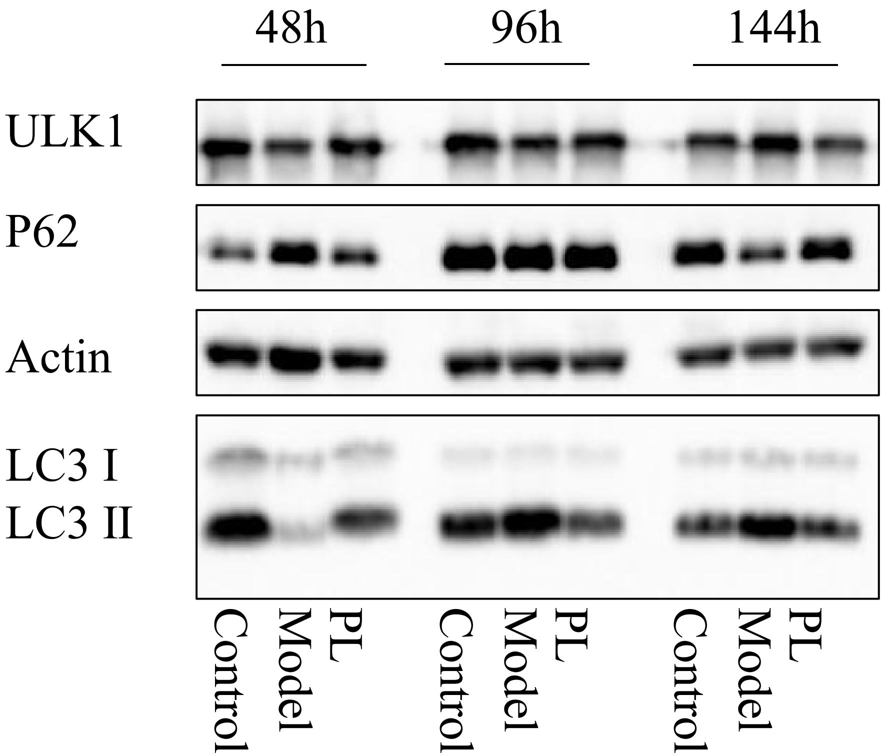

Original strips——3

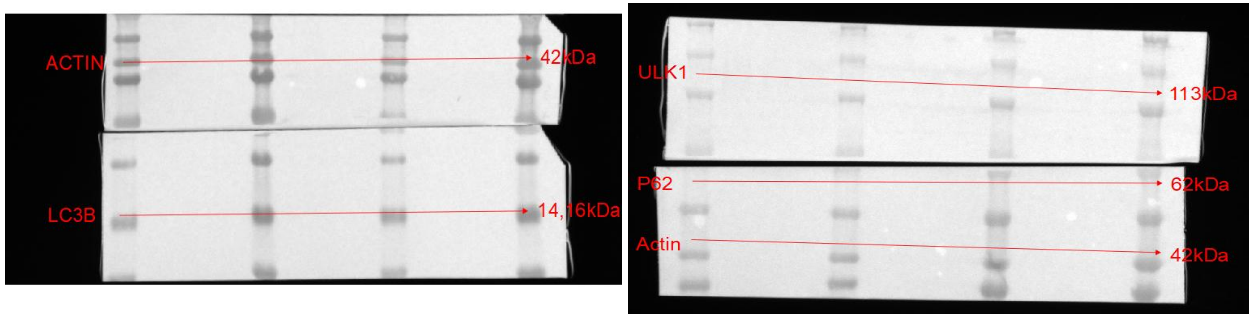

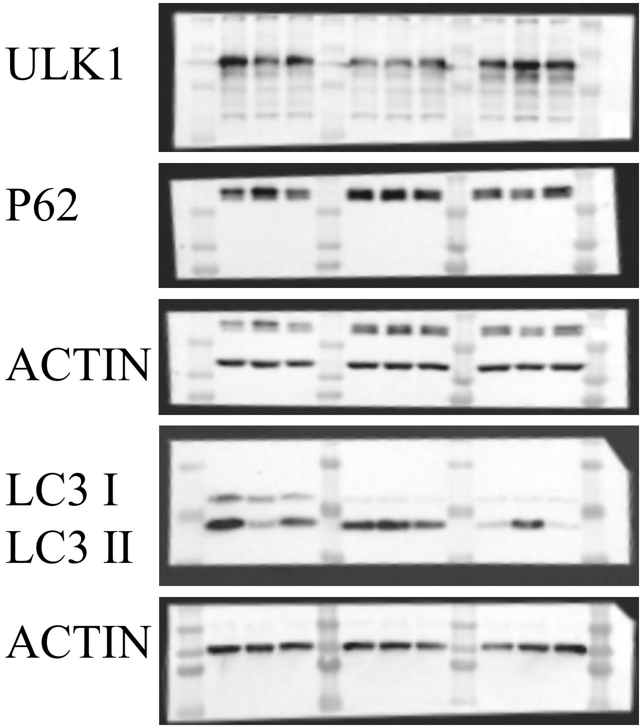

Processing strips——3

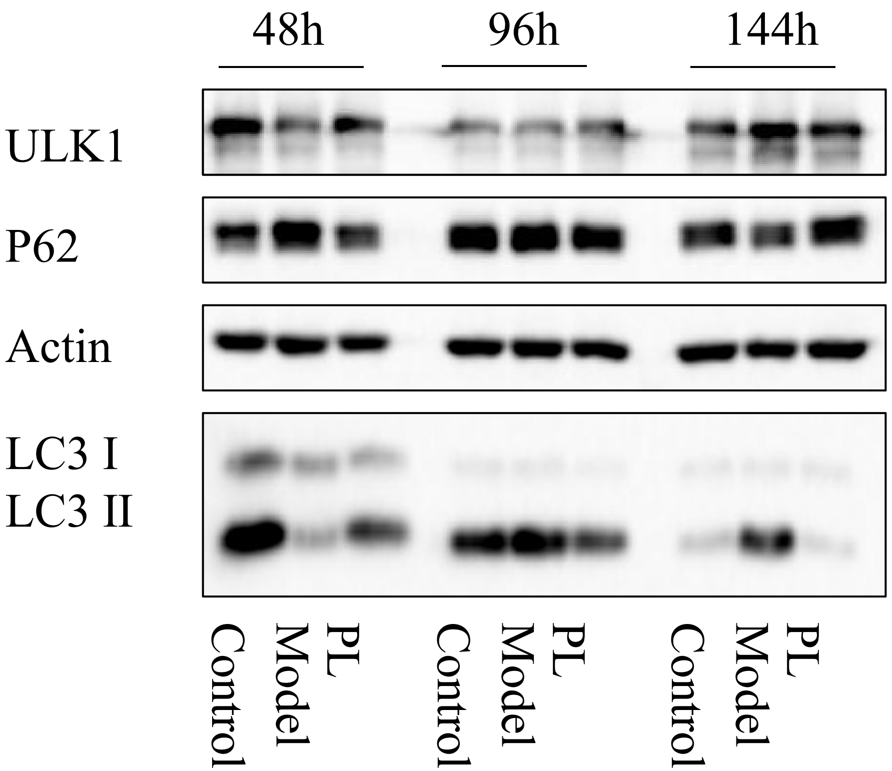

Supplement: S1 File — (ZIP) [file pone.0324264.s001.zip › supplement.material-1/western blot/cell/western blot.pdf]
